# Design and Biological Evaluation of Small-Molecule PET-Tracers for Imaging of Programmed Death Ligand 1

**DOI:** 10.3390/cancers15092638

**Published:** 2023-05-06

**Authors:** Fabian Krutzek, Cornelius K. Donat, Martin Ullrich, Kristof Zarschler, Marie-Charlotte Ludik, Anja Feldmann, Liliana R. Loureiro, Klaus Kopka, Sven Stadlbauer

**Affiliations:** 1 Helmholtz-Zentrum Dresden-Rossendorf, Institute of Radiopharmaceutical Cancer Research, Bautzner Landstraße 400, 01328 Dresden, Germany; f.krutzek@hzdr.de (F.K.); c.donat@hzdr.de (C.K.D.); m.ullrich@hzdr.de (M.U.); k.zarschler@hzdr.de (K.Z.); m.ludik@gmx.de (M.-C.L.); a.feldmann@hzdr.de (A.F.); l.loureiro@hzdr.de (L.R.L.); k.kopka@hzdr.de (K.K.); 2 School of Science, Faculty of Chemistry and Food Chemistry, Technische Universität Dresden, Mommsenstraße 4, 01069 Dresden, Germany; 3 German Cancer Consortium (DKTK), Partner Site Dresden, Fetscherstraße 74, 01307 Dresden, Germany; 4 National Center for Tumor Diseases (NCT) Dresden, University Hospital Carl Gustav Carus, Fetscherstraße 74, 01307 Dresden, Germany

**Keywords:** radiotracer, PET imaging, PD-L1, immune checkpoint inhibitors, small molecules, organic synthesis

## Abstract

**Simple Summary:**

PD-L1 plays a crucial role in the immune responses against cancer. Only around 30% of cancer patients respond to an anti-PD-L1 immune checkpoint inhibitor therapy. Noninvasive molecular imaging techniques such as positron emission tomography (PET) would allow identification of patients likely to respond. Here we report on the synthesis of nine PET radioligands targeting PD-L1, based on small-molecule inhibitors. We introduced a chelator for radiolabeling and water-soluble groups to aim for clearance through the kidneys. The compounds showed binding affinities toward PD-L1 in the lower nanomolar range and stability in vitro. Mouse experiments showed moderate accumulation in tumor tissue but mainly clearance through the liver. Additionally, the compounds showed unexpected long circulation times due to strong binding to albumin in blood. Nevertheless, our compounds are a starting point for further development of PD-L1 small molecule PET radiotracer to support therapy decisions.

**Abstract:**

Noninvasive molecular imaging of the PD-1/PD-L1 immune checkpoint is of high clinical relevance for patient stratification and therapy monitoring in cancer patients. Here we report nine small-molecule PD-L1 radiotracers with solubilizing sulfonic acids and a linker–chelator system, designed by molecular docking experiments and synthesized according to a new, convergent synthetic strategy. Binding affinities were determined both in cellular saturation and real-time binding assay (LigandTracer), revealing dissociation constants in the single digit nanomolar range. Incubation in human serum and liver microsomes proved in vitro stability of these compounds. Small animal PET/CT imaging, in mice bearing PD-L1 overexpressing and PD-L1 negative tumors, showed moderate to low uptake. All compounds were cleared primarily through the hepatobiliary excretion route and showed a long circulation time. The latter was attributed to strong blood albumin binding effects, discovered during our binding experiments. Taken together, these compounds are a promising starting point for further development of a new class of PD-L1 targeting radiotracers.

## 1. Introduction

The tumor microenvironment is a very dynamic immunosuppressive network, comprising a range of T, B, and NK cells which, together with endothelia, associate with the extracellular matrix and cancer cells. Within that network, adipocytes, regulatory T (Treg) cells, fibroblasts, macrophages, and cytokines promote cellular proliferation in all stages of cancer [1]. Therefore, these cellular and extracellular components represent a multitude of potential imaging biomarkers for early detection of tumor disease and monitoring of treatment response. Furthermore, they also provide a repertoire of targets for cancer therapy. Immune checkpoint inhibitor therapy addressing the programmed death ligand 1 (PD-L1, CD274) is a promising therapeutic strategy for cancer patients. However, only an average of 30% of patients respond to a checkpoint inhibitor monotherapy [2,3,4]. In order to identify these responders, noninvasive molecular imaging techniques such as positron emission tomography (PET) and single-photon emission computed tomography (SPECT) are ideal. These techniques provide advantages over currently employed immunohistochemistry methods in biopsies because they can fully address the issue of heterogeneous expression of PD-L1 over time and tissue. In addition, PET and SPECT allow for a noninvasive whole-body monitoring, thus avoiding repeated biopsies, which are burdensome for patients. Providing clinicians with a diagnostic tool for supporting therapy decisions is therefore an unmet need in the field of immune checkpoint inhibitor therapy [5]. So far, radiotracers targeting PD-L1 based on antibodies [6,7,8,9,10,11], nanobodies [12,13,14,15], affibodies [16], or adnectines [17] have been reported and are partly undergoing clinical trials [18,19,20]. These exhibit advantageously high accumulation in tumor tissues [21]. However, most of these high molecular weight molecules possess long circulation times requiring long-living radionuclides, which cause an additional radiation burden for the patient. Another important point is their relatively high immunogenicity, potentially causing adverse immunological effects, which are difficult to manage [22]. The manufacturing and healthcare costs of these compounds are high [23] and they are more difficult to modify, as compared to peptides or small molecules.

Hence, peptides and nonpeptide small molecules are favorable imaging agents because they possess higher tissue and tumor penetration, a relatively low immunogenicity, and short clearance times, therefore producing higher imaging contrast within minutes to hours and are synthetically easily accessible [21,24]. In recent years, a number of peptide-based PD-L1 radiotracers have been developed and preclinically evaluated [25,26,27,28,29]. A cyclic peptide WL12, labeled with copper-64, has been recently evaluated in a first clinical study [30]. Small molecules have been reported as inhibitors for the treatment of PD-L1 positive cancers [31,32,33]; however, so far only two papers report on the development of a radiolabeled small-molecule radiotracer [34,35].

The introduction of a radiolabel can potentially alter the physicochemical and biological properties of small molecules. Therefore, its introduction site must be carefully selected. To achieve reasonable biodistribution of the resulting radioligand, the pharmacokinetic properties have to be adjusted by introducing hydrophilic moieties into lipophilic lead structures to achieve favorable renal secretion rather than undesirable hepatobiliary secretion [36,37,38]. One elegant way is the introduction of a hydrophilic chelator such as DOTA, which can counterbalance the lipophilicity of a small molecule. The clinically used FAP-targeting ligands are successful examples of this concept, which obtain their water solubility from a hydrophilic piperazine linker and a DOTA or NOTA chelator [39,40,41,42].

Here we report on the synthesis and radiochemistry, as well as the in vitro and in vivo evaluation, of a new series of PD-L1-targeting radiotracers based on biphenyl small-molecule inhibitors. We implemented a new convergent synthetic strategy, offering advantages compared to the reported linear synthesis of PD-L1 inhibitors. Existing PD-L1 inhibitors were modified through introduction of a DOTA chelator via a hydrophilic linker to allow labeling with the positron emitters gallium-68 and copper-64. These new radioligands were tested using in vitro cell binding assays to determine the corresponding binding affinities. The preclinical evaluation was complemented by comprehensive stability and albumin binding studies, as well as small animal-PET/CT imaging and ex vivo biodistribution experiments in PD-L1-positive tumor-bearing mice.

## 2. Results

### 2.1. Organic Synthesis

#### 2.1.1. Synthetic Strategy Based on PD-L1 Cocrystal Structures

Based on the cocrystallization of the PD-L1 protein with the inhibitor BMS-1166 by the Holak group in 2021 [43], we rationalized our radioligand design on these findings.

**Figure 1:**
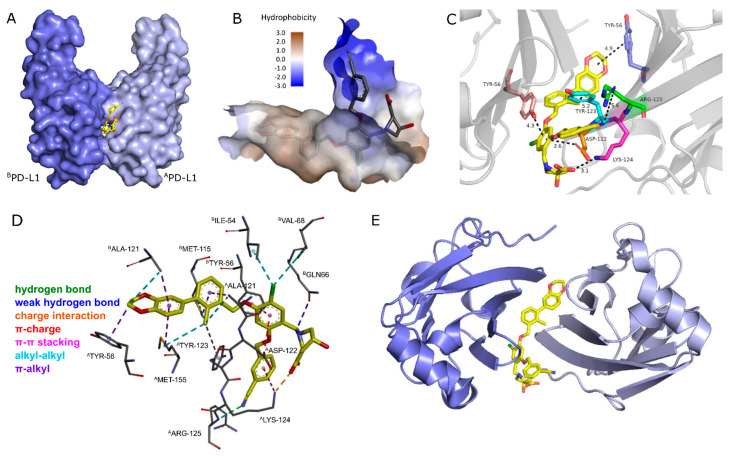
(**A**) Surface model of dimeric PD-L1 proteins with BMS-1166 as inhibitor. (**B**) Surface model displaying hydrophobicity in cavity and solvent region. (**C**) Zoomed-in view of BMS-1166 in PD-L1 dimer cavity displayed in cartoon model with a few important protein–ligand interactions displayed. (**D**) Detailed protein–ligand interactions in stick model with color visualization of interaction types. (**E**) Top view of PD-L1 dimer in cartoon. Distances are given in angstrom. The crystal structure of the dimeric PD-L1 protein with BMS-1166 inhibitor was taken from the PDB databank (PDB code: 6R3K).

The small-molecule inhibitors containing the well-known biphenyl core induce dimerization of two PD-L1 proteins with a deep cylindrical and hydrophobic cavity, which is mainly occupied by the biaryl and tetrasubstituted chlor-aryl (Figure 1A). The dioxane ring forms alkyl-alkyl and alkyl-*π* interactions with ^B^Ala-121 and ^A^Tyr-56, respectively. ^B^Ala-121 also interacts with the *π*-system of the adjacent phenyl ring, which additionally builds alkyl-interactions with ^A^Met-155. The second ring of the biaryl also interacts in a *π*-alkyl manner with two residues from different PD-L1 monomers, ^A^Ala-121 and ^B^Met-155. The ortho-substituted methyl group enhances the binding to PD-L1 with several hydrophobic alkyl-alkyl and π-alkyl interactions (Figure 1D). The central aryl with chlorine substitution forms π-π-stacking with ^B^Tyr-56 and builds π-charge interactions with the carboxylic group from ^A^Asp-122. The chlorine atom forms hydrophobic interactions with ^B^Ile-54 and ^B^Val-68. It has been shown that the pyridine ring with its nitrile moiety contributes with a hydrogen bond to ^A^Arg-125 and a π-charge interaction with ^A^Lys-124, which is also responsible for the strongly hydrophilic binding with the carboxylic group from the hydroxy-proline moiety (Figure 1C,D).

Based on these findings, we aimed to maintain as many interactions as possible, while transforming the PD-L1 inhibitors into radioligands. It has been shown that the dioxane ring induces a tyrosine folding (^A^Tyr-56) [44,45], which we wanted to mimic by a triazole unit, also enabling simple and fast modifications at this position through copper-catalyzed CuAAC reaction. The biaryl core, known to be crucial for binding to the protein in the narrow hydrophobic tunnel, (Figure 1B) was preserved in our radioligand design [46]. Since a bromobiphenyl unit was claimed to exhibit even better binding affinities than the dimethyl biphenyl unit, we hoped that the former one would benefit our radioligands, too. It has been reported that replacement of the chlorine at the central aryl with the larger and more hydrophobic bromine atom is beneficial to the affinity [47]. To extend this trend, we replaced it by the even larger iodine to further improve the binding affinity. By substituting the nitrile group with a methyl sulfone moiety at the pyridine ring, we aimed to maintain the affinity [48] and at the same time increase the hydrophilicity of the molecule. As indicated in Figure 1B, the hydrophobic tunnel ends in a hydrophilic region, which was occupied with a hydrophilic amino acid. To overcome the hydrophobic nature of these biphenyl-based PD-L1 inhibitors, we decided to substitute the cyclic amino acid with a strongly hydrophilic bis(sulfonic acid) moiety, attached via a sarcosine spacer to the central aryl and thus ensure that the sulfonate groups are exposed in the hydrophilic region and to the solvent outside the cavity. This was expected to provide the necessary hydrophilicity to the radioligand for being sufficiently soluble in the blood pool, resulting in fast pharmacokinetics with reduced hepatobiliary excretion.

**Figure 2:**
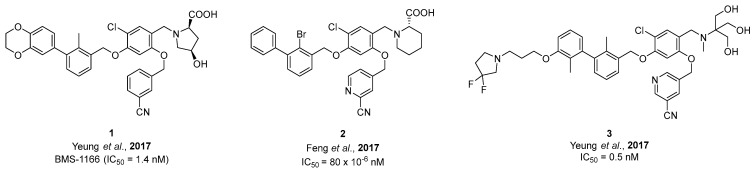
Structures and reported IC_50_ values of three selected PD-L1 inhibitors from the literature [48,51].

The chelator introduces additional water solubility and was attached to the biaryl of the molecule. The top view of the PD-L1 dimer (Figure 1E) indicates that the end of the hydrophobic tunnel is beyond the dioxane ring. This has been shown for dimeric PD-L1 inhibitors, too, which extend out of the cavity [49]. Among a number of reported inhibitors, molecule **3** (Figure 2) is an example of extensions at this position being tolerated [45,50]. Therefore, we introduced the chelator at this position via a linker to ensure spatial distance to the binding site, thus preventing negative influence on the binding affinity. We chose DOTA as a chelator because of its versatility, allowing complexation of different diagnostic and therapeutic radionuclides, such as ^68^Ga, ^64^Cu and ^177^Lu. As a structural basis for the development of PD-L1 targeting radioligands, we selected inhibitors **1**, **2**, and **3**, as these molecules are reported to possess high affinities toward PD-L1 (Figure 2).

**Figure 3:**
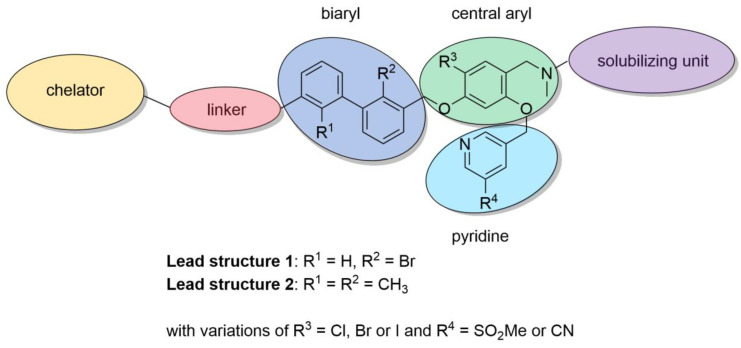
Structural modifications for PD-L1 ligand development.

**Scheme 1:**
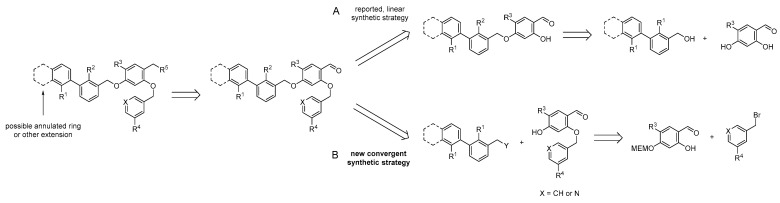
Retrosynthetic comparison of the reported synthetic routes for PD-L1 inhibitors (**A**) with our convergent synthetic strategy toward PD-L1 radiotracers (**B**).

As a result of the aforementioned considerations, we merged the structural elements of those three inhibitors into two main lead structures, containing either a bromobiphenyl or a dimethyl biphenyl core (Figure 3). For structure activity relationship (SAR) studies, we synthesized a library of PD-L1-targeting ligands by developing a new synthetic route for accessing these compounds. Our synthetic route is convergent, allowing faster synthesis through modular building blocks (see Scheme 1). The previously reported inhibitor syntheses are based on a linear synthesis. In contrast, our route introduces first the pyridine at the central aryl moiety followed by attaching the biaryl. This approach enables the rapid exchange of R^3^ and R^4^ to obtain a series of PD-L1 ligands with less synthetic demand. In a first series, we synthesized six PD-L1 ligands derived from two binding motifs with three different linkers.

#### 2.1.2. Synthesis of First Series of PD-L1 Ligands

**Scheme 2:**
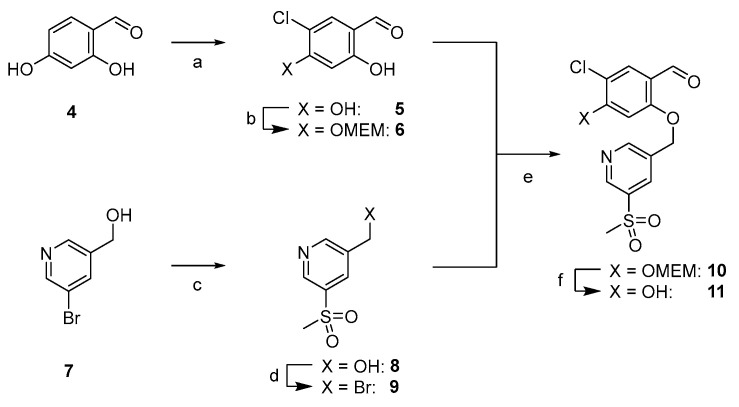
Synthesis of central ether-bridged arylic unit 11. (**a**) NCS, CHCl_3_, reflux, and 16 h, then HCl_conc_, CHCl_3_, reflux, 16 h, and 54%. (**b**) MEMCl, abs. DIPEA, abs. THF, 0 °C to rt, 40 h, and 60%. (**c**) NaCH_3_SO_2_, CuI, d-proline, NaOH, argon, DMSO, 90 °C, 60 h, and 61%. (**d**) PBr_3_, abs. DCM, 0 °C to rt, 16 h, and quant. (**e**) K_2_CO_3_, abs. DMF, rt, 16 h, and 84%. (**f**) TFA:DCM (1:1), 0 °C to rt, 16 h, and 82% as TFA salt.

The synthesis starts with the preparation of the tetrasubstituted chloro aryl building block bearing the pyridine moiety (see Scheme 2). Starting from commercially available 2,4-dihydroxybenzaldehyde **4**, chlorination at the 5-position was performed with NCS according to the literature [52]. The 4-hydroxy group was selectively MEM-protected, leading to **6**. Alkylation at the 2-OH was not observed, unless catalytic amounts of tetrabutylammonium iodide were added, which led to double alkylation. The second building block **9** was obtained by sulfination [48] with sulfinic acid sodium salt followed by bromination with phosphorus tribromide. Nucleophilic substitution reaction between **6** and **9** was performed in K_2_CO_3_/DMF providing compound **10,** which was deprotected with DCM/TFA (1:1).

**Scheme 3:**
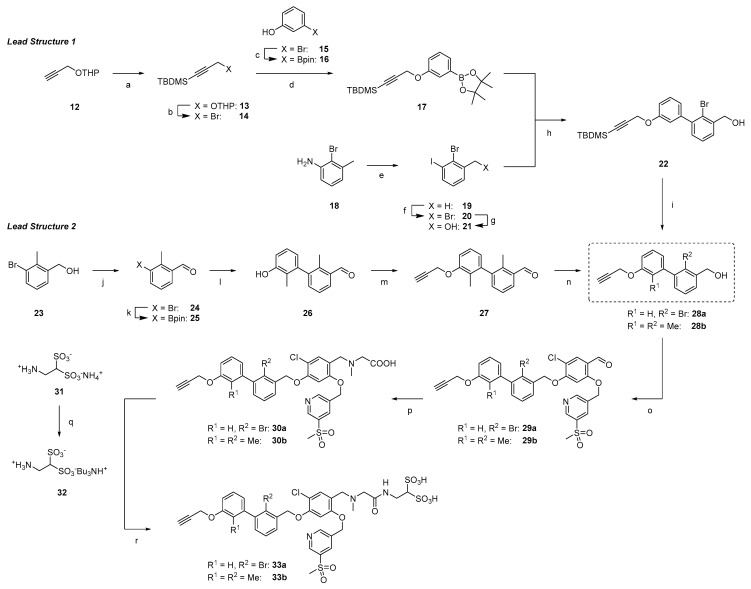
Synthetic pathways to key alkyne intermediates **33a** and **33b** for lead structure **1** and **2**. (**a**) TBDMSCl, *n*-BuLi, abs. THF, −78 °C to rt, 16 h, and 97%. (**b**) PPh_3_Br_2_, DCM, rt, 3 h, and 69%. (**c**) B_2_pin_2_, Pd(dppf)Cl_2_, argon, KOAc, 1,4-dioxane, 90 °C, 16 h, and 86%. (**d**) K_2_CO_3_, abs. DMF, rt, 16 h, and 68%. (**e**) NaNO_2_, KI, 1 M HCl_(aq)_, 0 °C to rt, 3 h, and 76%. (**f**) NBS, AIBN, argon, CCl_4_, reflux, 16 h, and 51%. (**g**) DMF/H_2_O (8:2), 80 °C, 16 h, and 75%. (**h**) Pd(PPh_3_)_4_, K_2_CO_3_, argon, toluene/EtOH/H_2_O (20:0.5:0.1), 80 °C, 16 h, and 63%. (**i**) TBAF (1 M in THF), abs. THF, 0 °C to rt, 2 h, and 85%. (**j**) MnO_2_, DCM, rt, 16 h, and 65%. (**k**) B_2_pin_2_, Pd(dppf)Cl_2_, NaOAc, argon, DMF, 110 °C, 16 h, and 67%. (**l**) 3-bromo-2-methylphenol, Pd(PPh_3_)_4_, K_2_CO_3_, argon, toluene/EtOH/H_2_O (20:0.5:0.1), 100 °C, 16 h, and 90%. (**m**) propargyl bromide, K_2_CO_3_, abs. DMF, rt, 16 h and 91%. (**n**) NaBH_4_, abs. MeOH/DCM (1:1), 0 °C to rt, 3 h, and 86%. (**o**) DEAD, PPh_3_, abs. DMF, 0 °C to rt, 16 h, and 80% (**29a**), 86% (**29b**). (**p**) sarcosine, NaBH_3_CN, abs. MeOH/DMF (1:1), 0 °C to rt, 16 h, and 56% (**30a**) 38% (**30b**). (**q**) Amberlyst™ WET 15H, H_2_O, rt, 16 h, then tributylamine, H_2_O, rt, 24 h, and quant. (**r**) HBTU, DIPEA, abs. DMF, rt, 16 h, and 60% (**33a**), 37% (**33b**).

Scheme 3 shows the synthetic route toward alkyne key intermediates for both lead structures. THP-protected propargyl alcohol **12** was TBDMS-protected [53] and the OTHP group directly transformed into the corresponding bromide using PPh_3_Br_2_ [54], thereby shortening a two-step procedure reported in the literature [53]. The bromide **14** was then reacted with borylated 2-bromo phenol [55] **16**, providing **17**. The Sandmeyer reaction of commercially available 2-bromo-3-methyl aniline (**18**) followed by radical bromination led to **20,** which was further converted to benzylic alcohol **21** [56]. Suzuki coupling of fragments **17** and **21** provided biaryl **22**, followed by TBDMS deprotection, yielding **28a**.

The synthesis of the analogous compounds based on lead structure 2 started with oxidation of **23** with manganese (II) oxide and subsequent borylation, leading to aldehyde **25** [57]. Suzuki coupling with 3-bromo-2-methylphenol followed by *O*-alkylation with propargyl bromide yielded **27**. After reduction of the aldehyde with sodium borohydride, the second key building block **28b** was obtained.

Both biarylic cores **28a** and **28b** underwent Mitsunobu reactions with phenol **11** in good yields (80% and 86% for **29a** and **29b**, respectively). Reductive amination with sarcosine provided carboxylic acids **30a** and **30b** in moderate yields (56% and 38%, respectively). Amide bond formation with 2-aminoethane-1,1-disulfonic acid **32** (as TBA salt [58]) was achieved through standard HTBU/DIPEA/DMF coupling. After reverse phase HPLC separation and desalting with Amberlyst™ WET15, bis(sulfonic acid) derivatives **33a** and **33b** were obtained in their H^+^ form, which served as key intermediates for the synthesis of different PD-L1 ligands.

**Scheme 4:**
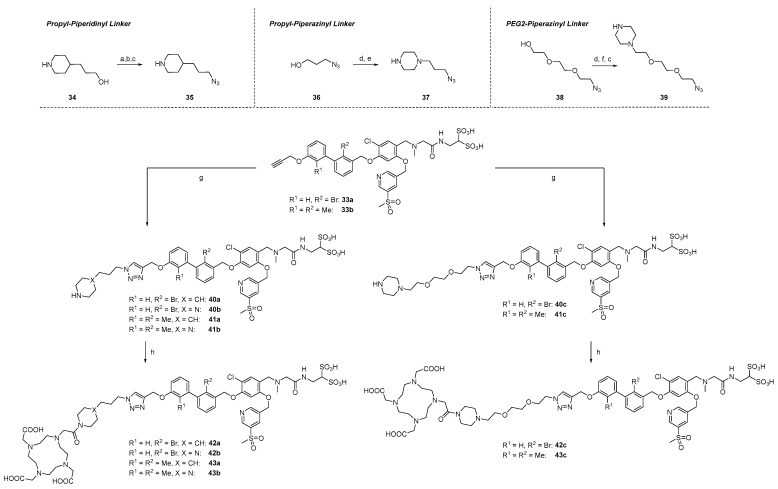
Synthesis of linker structures, their attachment to alkynes and DOTA-conjugation. (**a**) Boc_2_O, 1,4-dioxane/1 M NaOH (1:1), 0 °C to rt, 16 h, and 63%. (**b**) ADMP, DBU, abs. THF, rt, 16 h, and 85%. (**c**) TFA/DCM (1:1), 0 °C to rt, 16 h, and quant. for **35** and **39**. (**d**) TsCl, pyridine, abs. DCM, 0 °C to rt, 16 h, and 81% (**37**), 93% (**39**). (**e**) piperazine, THF, 50 °C, 16 h, and 50%. (**f**) *N*-Boc-piperazine, K_2_CO_3_, abs. DMF, rt, 16 h, and 73%. (**g**) [Cu(MeCN)_4_]PF_6_, TBTA, argon, DMF, rt, 16 h, and 66% (**40a**), 29% (**40b**), 68% (**40c**), 61% (**41a**), 45% (**41b**), 54% (**41c**). (**h**) DOTA-PNP, abs. DIPEA, abs, DMF, rt to 80 °C, 44 h, and 36% (**42a**), 75% (**42b**), 42% (**42c**), 37% (**43a**), 75% (**43b**), and 33% (**43c**).

Three different linker structures were synthesized (Scheme 4) to serve as a spacer between the binding motif and chelator. The piperazine-propyl linker was adapted from the development of FAP radiotracers due to its hydrophilicity and wide presence in biological active compounds [59,60]. By replacing the piperazine ring with a piperidine ring or the propyl chain with a PEG2 chain, we aimed to modify the hydrophilicity. For the propyl-piperidinyl linker building block, the synthesis started with commercially available 3-(piperidin-4-yl)propan-1-ol **34**, which was Boc-protected, then transformed into its azide with ADMP/DBU [61] and finally deprotected with TFA/DCM (1:1), providing linker **35**. The propyl-piperazinyl linker **37** was accessed from 3-azidopropanol **36** and a subsequent nucleophilic substitution with piperazine, according to the literature [62]. In a similar manner, a PEG2 piperazinyl linker was synthesized by tosylation of alcohol **38** and conjugation to 1-Boc-piperazine. Deprotection under acidic conditions provided the most hydrophilic linker **39** as a free base. These linker structures were coupled with alkynes **33a** and **33b** in a Copper(I)-catalyzed Azide-Alkyne Cycloaddition (CuAAC), leading to compounds **40a**–**41c** in 29–68% yield. Because the secondary amines in piperazine amines are less reactive, the conjugation with commercially available DOTA-OSu failed. Therefore, we employed the more reactive DOTA-*p*-nitrophenylester [63] for the conjugation reaction. After stirring for 48 h, the starting material was consumed and a complex mixture via analytical HPLC was observed. Side products were identified as final compounds with several (*n* = 1, 2, 3) *p*-nitrophenyl moieties conjugated to the chelator. Stirring at 70 °C for 3 h cleaved the thermally instable *p*-nitrophenyl multimers and after HPLC chromatography, the final compounds **42a**–**43c** were obtained in 33 to 75% yield.

#### 2.1.3. Exploration of Larger Halogens at the Central Aryl Moiety

**Scheme 5:**
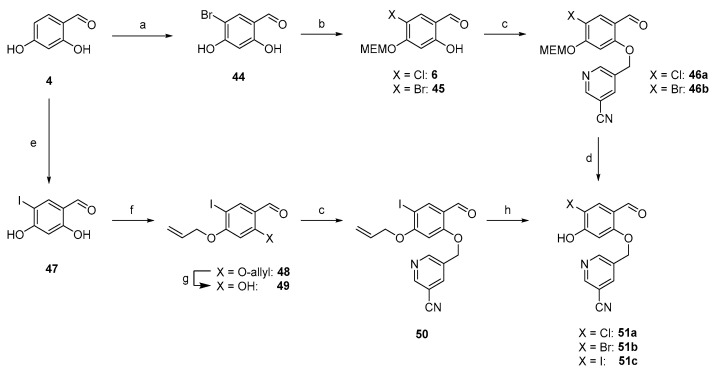
Synthesis of central building block with Cl, Br, and I at 4-position. (**a**) Br_2_, AcOH, rt, 3 h, and 36%. (**b**) MEMCl, abs. DIPEA, abs. THF, 0 °C to rt, 16 h, and 49%. (**c**) 5-(bromomethyl)-3-pyridinecarbonitrile, K_2_CO_3_, abs. DMF, rt, 16 h, and 93% (**46a**), 87% (**46b**), 74% (**50**). (**d**) TFA:DCM (1:1), 0 °C to rt, 16 h, and 92% (**51a**), 82% (**51b**) as TFA salts. (**e**) ICl, AcOH, 0 °C to rt, 5 h, 19%. (**f**) allyl bromide, K_2_CO_3_, NaI, abs. DMF, 50 °C, 1 h, and 96%. (**g**) TiCl_4_, TBAI, abs. DCM, rt, 16 h, 61%. (**h**) Pd(PPh_3_)_4_, K_2_CO_3_, MeOH, rt, 16 h, and 33%.

Based on the results obtained in our in vitro studies (see below), we focused on the dimethylbiphenyl biaryl core (R^1^ = R^2^ = Me). To study the effect of the SO_2_Me versus the CN group, we replaced the methylsulfonyl moiety at the pyridinylmethyl ether with a nitrile, which is present in the parent inhibitor molecules (Scheme 5). Additionally, we replaced the chloride on the central aromatic core with a bromide, as that modification was reported to improve binding affinities [47]. To extend this concept even further, we replaced the chlorine by the large iodine, too. To the best of our knowledge, this has not been reported for any PD-L1 inhibitor. These modifications led to three additional PD-L1 ligands. Synthesis started with 2,4-dihydroxybenzaldehyde (**4**), which was on the one hand brominated with elemental bromine [64] and on the other hand iodinated with iodine chloride [65]. Both reactions were performed in acetic acid leading to **44** and **47**. In analogy to **6**, the bromine derivative **44** was selectively MEM-protected at position 4 in 49% yield. However, for the iodine derivate **47,** double protection of both hydroxy groups was observed under the same conditions. Therefore, we adopted a route reported elsewhere [65]: Alkylation of both hydroxyl groups with allyl bromide and selective deprotection of the 2-allylic group with TiCl_4_. This route provided **49** in 59% yield over two steps. All three derivatives, **6**, **45** and **49,** were alkylated with commercially available 5-(bromomethyl)-3-pyridinecarbonitrile leading to chlorine derivative **46a**, bromine derivative **46b,** and iodine derivative **50**, in yields between 74 and 93%. MEM deprotection of **46a** and **46b** proceeded smoothly, yielding central building blocks **51a** and **51b** as TFA salts in excellent yields, whereas the allylic deprotection with Pd(PPh_3_)_4_/K_2_CO_3_ resulted only in a moderate yield (33%).

**Scheme 6:**
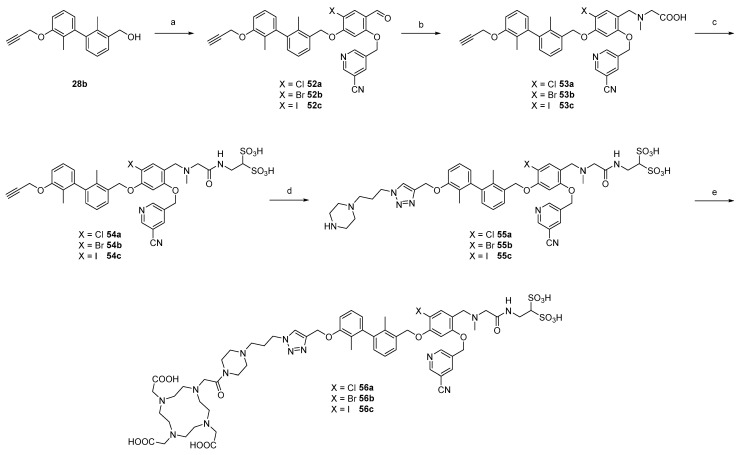
Synthesis of 2^nd^ series radiotracers with different halogens at 4-position of central arylic core. (**a**) **51a**, **51b** or **51c**, DEAD, PPh_3_, abs. DMF, 0 °C to rt, 16 h, and 65% (**52a**), 59% (**52b**), 57% (**52c**). (**b**) sarcosine, NaBH_3_CN, abs. DMF/MeOH (1:1), 0 °C to rt, 16 h, and 59% (**53a**), 65% (**53b**), and 68% (**53c**). (**c**) HBTU, abs. DIPEA, abs. DMF, rt, 16 h, and 81% (**54a**), 63% (**54b**), and 56% (**54c**). (**d**) **37**, sodium ascorbate, CuSO_4_, THPTA, H_2_O/*t*-BuOH (1:1), rt, 3 h, and 46% (**55a**), 63% (**55b**), and 67% (**55c**). (**e**) DOTA-PNP, abs. DIPEA, abs, DMF, rt to 80 °C, 44 h, and 59% (**56a**), 50% (**56b**), and 52% (**56c**).

With these three central building blocks in hand, the synthetic route proceeded like the one described above (Scheme 6). After Mitsunobu reaction of these phenols with **28b** (57–65% yield), reductive amination proceeded with good yields of 59 to 68%. Amide bond formation was achieved under standard conditions to yield key intermediates **54a**–**c**. Since the in vitro results showed the highest affinity for compound **42b** bearing the piperazine propyl-linker, we selected that linker for CuAAC reaction, which provided **55a**–**c** in yields between 46 and 67% after HPLC purification. DOTA conjugation was performed as described above, providing three 1-Boc-piperazine PD ligands **56a**–**c** in 50 to 59% yield.

### 2.2. Radiochemistry

#### 2.2.1. Radiolabeling with ^64^Cu, ^68^Ga, and ^177^Lu

Radiolabeling with ^64^Cu, ^68^Ga, and ^177^Lu was performed at pH 4 in a 1 M HEPES solution at 95 °C for 15 min. These conditions resulted in a labeling efficiency of 95% for all three radionuclides.

#### 2.2.2. Determination of logD_7.4_ Values

Partition coefficients log*D*_7.4_ between *n*-octanol and PBS-buffer pH 7.4 were determined for the ^64^Cu-labeled compounds, using the shake flask method, summarized in Table 1. The obtained values ranged between −2.73 for **42a** and −3.50 for **43c**, indicating acceptable water solubilities for an in vivo application.

### 2.3. Stability Studies

The kinetic stability of the compounds was studied in different buffer systems, whereas the proteolytic stability was investigated in the presence of human serum. Finally, their metabolic stability was analyzed using human liver microsomes. To allow data acquisition for up to seven days, all experiments were performed with ^177^Lu-derivatives of the radiotracers due to the physical half-life (t_1/2_ = 6.65 d).

#### 2.3.1. Kinetic Stability in Buffer Solutions

The potential degradation of the ^177^Lu-labeled compounds **42c**, **43,** and **56a**–**c** in aqueous HEPES as well as PBS solution was monitored by radio-HPLC. The corresponding chromatograms in Supplementary Figures S234 and S235 consistently show a major peak with a retention time of ~5.5 min and a minor peak at ~1.3 min, originating from the intact radiolabeled compounds and uncomplexed ^177^Lu, respectively. No radiotracer decomposition was observed upon incubation in HEPES solution at room temperature over a period of seven days, resulting in >96% of intact compounds present at all time points evaluated. Comparable results were obtained for the incubation in PBS, except for [^177^Lu]Lu-**56c,** which showed a slightly reduced stability of approximately 94.5% at the last time point (7 d).

#### 2.3.2. Proteolytic Stability in Human Serum

The susceptibility of the ^177^Lu-labeled compounds to proteolysis and associated therewith, the generation of degradation products, was investigated by exposing the radiotracers to human serum for up to seven days. For the ligands **42c**, **43c**, and **56a,b**, radio-HPLC analyses (Supplementary Figure S236) showed that radiotracers remain intact (>90%) in human serum throughout the duration of the study, whereas the percentage of intact [^177^Lu]Lu-**56c** was slightly reduced after seven days of incubation (~84%). 

#### 2.3.3. Metabolic Stability

**Figure 4:**
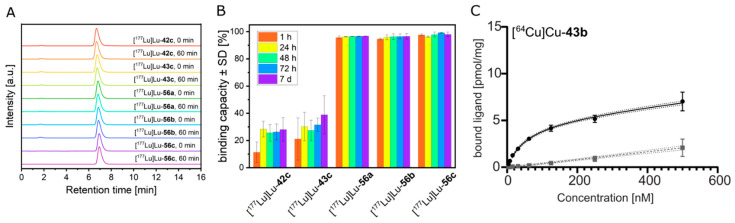
(**A**) Radio-HPLC chromatograms of [^177^Lu]Lu-tracers of **42c**, **43c**,and **56a**–**c** after incubation with liver microsomes. (**B**) Serum protein binding capacity of ^177^Lu-labeled compounds **42c**, **43c,** and **56a**–**c**. The radiotracers were incubated with human serum for up to seven days and serum samples were analyzed by size exclusion chromatography at indicated time points. The percentage of serum-associated radiotracer was calculated using the integrated peak areas of corresponding size-exclusion chromatograms. (**C**) Nonlinear iterative curve fitting of saturation binding experimental data for [^64^Cu]Cu-**43b** (*n* = 3 independent experiments). Total binding is represented by the solid black curve (with dotted line showing the 95% confidence interval), while the grey line (dotted line showing the 95% confidence interval) represents nonspecific binding.

The time-dependent microsomal transformation of the radiolabeled ligands was evaluated to obtain basic information about their metabolic stability. Therefore, ^177^Lu-labeled compounds **42c**, **43c**, and **56a**–**c** were incubated with human liver microsomes, and at defined time points (until 60 min), the fractions of remaining intact radioligands were determined by radio-HPLC (Figure 4A). No difference in the peak number, shape, or retention time was observed between the chromatograms obtained for each individual ligand before and at the end of the incubation (0 vs. 60 min). In other words, in vitro radiometabolites of the investigated ^177^Lu-labeled compounds were not detectable.

### 2.4. Albumin Binding Experiments

Before studying the pharmacokinetic profile of the novel PD-L1 ligands by in vivo and organ distribution experiments, their interaction with serum proteins was investigated. ^177^Lu-labeled compounds **42c**, **43c**, and **56a**–**c** were incubated with human serum, and at defined time points (until seven days), the serum samples were separated by size exclusion chromatography. The resulting radio-HPLC chromatograms (Supplementary Figure S238) for each investigated radiotracer show two peaks with retention times of ~6.9 min and ~8.7 min, corresponding to the serum-associated and the unbound ligands, respectively (Supplementary Figure S237, Supplementary Table S1). Integration of the individual peaks allowed the quantification of serum protein binding capacity (Figure 4B). After 1 h of incubation, the vast majority of the ^177^Lu-labeled compounds **56a**–**c** already existed as serum-associated fractions (95.7%, 94.6% and 97.6%, respectively), whereas [^177^Lu]Lu-**42c** and [^177^Lu]Lu-**43c** bind to serum proteins to a much lesser extent. These values increased over time to ~28% for [^177^Lu]Lu-**42c** and 37% for [^177^Lu]Lu-**43c**, respectively, indicating an overall lower binding to human serum proteins.

To identify the particular serum proteins interacting with the ligands **42c**, **43c**, and **56a**–**c**, the serum samples were separated by native gel electrophoresis (Supplementary Figure S239). The autoradiographic image of the obtained polyacrylamide gel showed for each ^177^Lu-labeled compound a single band corresponding to human serum albumin. Its intensity is substantially higher in the serum samples of the ^177^Lu-labeled compounds **56a**–**c,** confirming their increased serum protein binding capacity compared to [^177^Lu]Lu-**42c** and [^177^Lu]Lu-**43c**. Interestingly, incubation of uncomplexed ^177^Lu with human serum resulted in a single band corresponding to serum transferrin, which apparently complexes the trivalent radiometal through its metal-binding sites [66,67]. The absence of the transferrin band in the serum samples containing ^177^Lu-labeled compounds **42c**, **43c**, and **56a**–**c** shows the absence of demetalation or transchelation and thus indicates the stability of the radioligands.

### 2.5. In Vitro Evaluation

#### 2.5.1. Saturation Binding

Equilibrium dissociation constants (K_D_) and maximum number of binding sites (B_max_) were derived from saturation binding in the presence of BSA. Data of first (**42a**–**c**, **43a**–**c**) and second series of compounds **56a**–**c**, along with the cyclic peptide WL12, are reported in Table 1 as their ^64^Cu complexes. Supplementary Figure S240 shows iterative curve fitting for first (A) and second series of compounds (C), along with the cyclic peptide WL12 (B).

Based on affinity derived from saturation binding, the compounds can be broadly classified into three groups: low, medium, and high affinity. Under identical conditions, compounds [^64^Cu]Cu-**42a**, [^64^Cu]Cu-**42b**, [^64^Cu]Cu-**42c**, and [^64^Cu]Cu-**56c** were found to express low affinity (>300 nM;), while compounds [^64^Cu]Cu-**43a**, [^64^Cu]Cu-**56a**, and [^64^Cu]Cu-**56b** showed medium affinity (100–300 nM). Lastly, compounds [^64^Cu]Cu-**43b** and [^64^Cu]Cu-**43c**, along with [^64^Cu]Cu-DOTAGA-WL12, showed a higher affinity (<100 nM). The maximum number of binding sites were found to differ as well, with compounds of the first series (**42a**–**c**, **43a**–**c**) showing lower B_max_ values as compounds of the second series (**56a**–**c**), especially [^64^Cu]Cu-**56c** and the cyclic peptide [^64^Cu]Cu-DOTAGA-WL12. [^64^Cu]Cu-DOTAGA-WL12 was assessed using the same conditions as the small molecules, yielding the best affinity of all compounds. However, this is approximately 15 times worse than previously reported using a SPR assay [68], probably reflecting the wider range of concentrations in our assay.

#### 2.5.2. Real-Time Radioligand Binding

**Figure 5:**
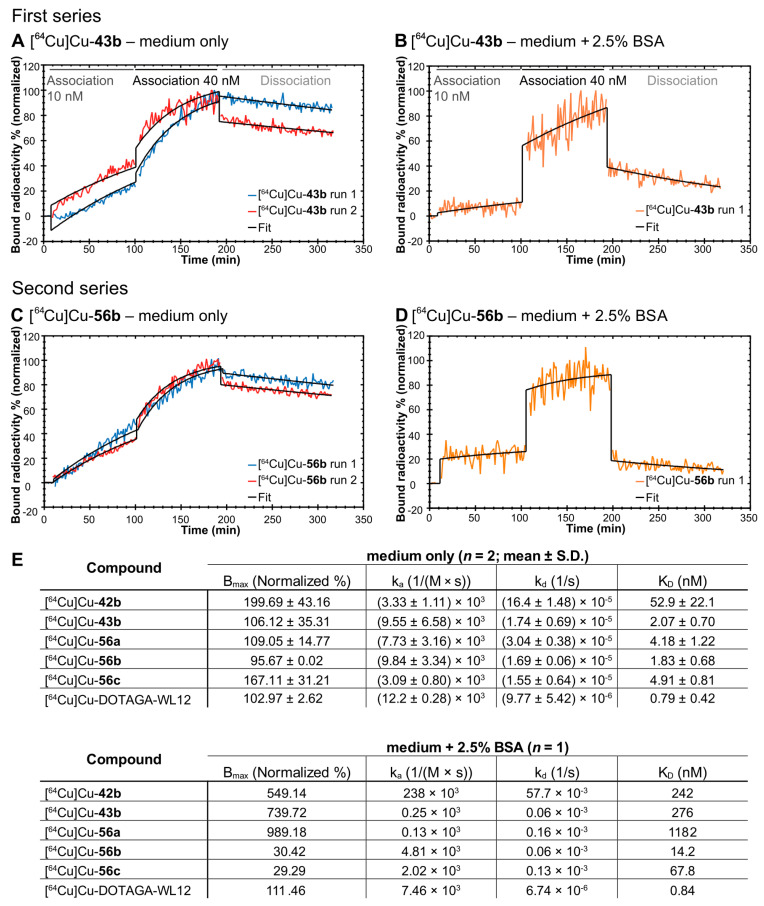
Real-time radioligand binding (trace) of [^64^Cu]Cu-**43b** and [^64^Cu]Cu-**56b** in the absence (**A**,**C**) and presence (**B**,**D**) of 2.5% bovine serum albumin (BSA). (**E**). Kinetic parameters (normalized maximum number of binding sites, association rate constant k_a_, dissociation rate constant k_d_, and dissociation constant K_D_) for compounds [^64^Cu]Cu-**42b** and [^64^Cu]Cu-**43b** (first series), complete second series along with [^64^Cu]Cu-DOTAGA-WL12.

As indicated by the albumin binding results, we suspected our ^64^Cu-labeled compounds to have some albumin binding capacity. As this could potentially interfere with the endpoint saturation measurements, we tested this hypothesis by employing another method: real-time radioligand binding [69]. Using the same cells and compounds, real-time binding data revealed a significant difference in kinetics between PD-L1 binding of our compounds, depending on whether bovine serum albumin was present or absent. As exemplified by [^64^Cu]Cu-**43b** and [^64^Cu]Cu-**56c** (Figure 5), incubation of cells with radioligand and medium only, yielded a constant concentration-dependent increase in binding (Figure 5A,C; Supplementary Figure S241A,C,E). This pattern indicates mainly association (10 nM, constant increase) followed by association and approaching equilibrium (40 nM, increase followed by flattening of the curve). When bovine serum albumin was present in the medium, the increase in signal was almost instantaneous and during the association phases, only a marginal increase was observed over time (Figure 5B,D; Supplementary Figure S241B,D,F). In contrast, binding of [^64^Cu]Cu-DOTAGA-WL12 to PD-L1 overexpressing cells was not affected by BSA in the medium and affinity was similar between both experimental conditions (Supplementary Figure S241G,H). In the presence of BSA, the binding pattern is likely a compounded signal of both the albumin binding in the liquid phase and the PD-L1 binding on the cellular surface. Hence, the binding curve without BSA likely reflects the actual binding of our compounds toward PD-L1. Under these conditions, all of our compounds, except [^64^Cu]Cu-**42b**, yielded binding affinities between 1.83–4.92 nM (Figure 5E), putting it in a similar range as [^64^Cu]Cu-DOTAGA-WL12.

### 2.6. PET Imaging and Biodistribution

Imaging of PD-L1 small-molecule tracer candidates was initially performed after ^68^Ga labeling. However, for all compounds reported in this study, the tracer uptake in tumor tissue did not reach a plateau within 4 h, making ^68^Ga unsuitable for PET imaging. Therefore, we changed to ^64^Cu with its longer half-life of 12.7 h for the imaging studies. All ligands showed different distribution behavior in vivo as expected from in vitro determined affinities. [^64^Cu]Cu-**43b** showed the most promising affinity (Table 1 and Figure 4C) and was therefore investigated in detail, using PET and biodistribution.

**Figure 6:**
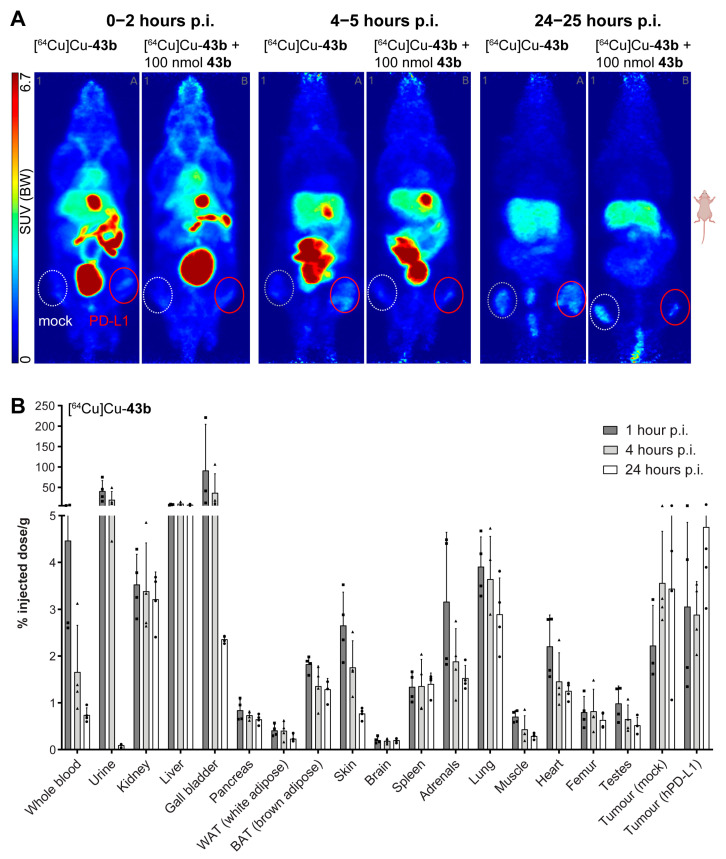
(**A**) Maximum intensity projections (prone view) of [^64^Cu]Cu-**43b** distribution in tumor-bearing (PC3 + mock left, white dotted line and PC3 + PD-L1 overexpressing right, red solid line) NMRI-Foxn1 nu/nu mice, at different time points after injections. (**B**) Biodistribution (% injected dose/g of [^64^Cu]Cu-**43b** in tumor-bearing NMRI-Foxn1 nu/nu mice (*n* = 4) at 1, 4, and 24 h postinjection (p.i.). Rectangles, triangles and circles represent individual datapoints (animals) of the respective timepoint.

Small-animal PET revealed a primarily hepatobiliary clearance of all radiotracers, as indicated by liver and intestinal activity distribution between 1 and 4.5 h post injection (Figure 6A and Figure S242–S244). By 24 h, the liver still showed substantial accumulation, especially for compounds [^64^Cu]Cu-**42b** and [^64^Cu]Cu-**56c**. Compounds [^64^Cu]Cu-**43b**, [^64^Cu]Cu-**43c**, [^64^Cu]Cu-**56b,** and [^64^Cu]Cu-**56c** appeared to have a longer blood retention time, as indicated by heart and vascular distribution within the first two hours. These findings were confirmed for compound [^64^Cu]Cu-**43b** through biodistribution data (Figure 6B). The albumin binding of our compound certainly provides a convincing explanation for these observed in vivo effects. An early (1–2 h) tumor uptake was only visible for compounds [^64^Cu]Cu-**56b** and [^64^Cu]Cu-**56c**, as indicated by SUV_max_ values >2 (Supplementary Tables S2 and S3). By 4 h, most compounds except [^64^Cu]Cu-**42a** reached an SUV_max_ of 2 and above in PD-L1 positive tumors. The contrast between PD-L1 overexpressing and mock construct tumors was found to be low for compounds [^64^Cu]Cu-**42a**–**b** and [^64^Cu]Cu-**43a**–**b** for the whole observation period, with SUV_max_ showing either no difference or a maximum increase of 67% (Supplementary Tables S2 and S3) over mock construct tumors. Compounds [^64^Cu]Cu-**42c** and [^64^Cu]Cu-**43c** showed a consistently fair contrast between PD-L1 and mock tumors up to 24 h post injection, with SUV_max_ between 60% and 470% higher in PD-L1 tumors. Tracer candidates of the second series ([^64^Cu]Cu-**56a**–**c**) showed a slightly faster tumor uptake in the first hours, however, without apparent decrease toward 24 h. The contrast between PD-L1 and mock tumors was more in line with compounds [^64^Cu]Cu-**42a**–**b** and [^64^Cu]Cu-**43a**–**b**, again with SUV_max_ showing no differences and a maximum increase of 114% over mock tumors. Interestingly, by 24 h p.i., the already low contrast between PD-L1 and mock tumors was lost for most compounds ([^64^Cu]Cu-56**b**–**c**), as SUVs did not differ. Blocking experiments were performed with 100 nmol of the respective unlabeled compounds **42a**–**b** and **43a**–**b**. However, only a low to moderate blocking effect was observed for all compounds, with slightly better results for [^64^Cu]Cu-**43b**. Again, for compound [^64^Cu]Cu-**43b**, biodistribution data (Figure 6B) confirmed the slightly higher SUV for PD-L1 overexpressing tumors early on (1–2 h p.i.), which was later lost (24 h p.i.). Again, the low SUV, slow kinetics and low efficacy of blocking can likely be explained by the high albumin binding of our compounds, preventing a suitably high free fraction of tracer available to bind to the target.

To confirm PD-L1 expression of injected cells, tumors were excised from animals not subjected to PET and random specimens stained using a monoclonal antibody against PD-L1. As expected, immunostaining confirmed target overexpression in PD-L1 tumors as compared to the ones formed from cells carrying a mock construct (Supplementary Figure S245).

## 3. Discussion

The discovery of the immune checkpoint inhibitors, such as cytotoxic T-lymphocyte antigen-4, PD-1, and its ligand PD-L1 as part of the same B7-CD28-CTLA4 family, has provided oncology with novel biomarkers and treatment options [70]. Clinical success of mobilizing the immune system via immune checkpoint therapies suggests further promising potential. However, the decision of whether patients would benefit from PD-1 pathway-targeting drugs relies primarily on biopsies and subsequent immuno(histochemical) assays. Both methods come with a number of caveats, such as disregarding temporospatial heterogeneity across the tumor volume [71] and varying diagnostic accuracy [72,73], attributed partially to assay differences [74]. Furthermore, only 30% of patients are estimated responders to PD-L1 targeted therapy [75], along with dynamic in vivo changes and possible treatment-related resistance [76], thus requiring better diagnostic tools. This provides an opportunity for noninvasive imaging using PET [77], SPECT, and optical methods, for patient stratification, therapy control, and surgical guidance. This has resulted in a number of radiolabeled complete antibodies and fragments, peptides, and few small molecules [5]. In comparison to larger constructs, small-molecule tracers offer some advantages, such as faster uptake and clearance and lower production costs. However, only a few PD-L1 peptidic and small-molecule tracers have been described so far.

For meeting the clinical need to predict the outcome of immune checkpoint inhibitor therapy, we developed novel PET radiotracers based on biphenyl small-molecule inhibitors. Through rational design, we identified two lead structures for the synthesis of PD-L1 targeting radiotracers. To meet the ADME criteria, the highly lipophilic parent biphenyl molecules had to be modified with water-soluble groups. We choose the bis(sulfonic acid) moiety, bearing two sulfonic acid groups in a geminal fashion, which we introduced in the solubilizer unit of the molecule, thereby ensuring interaction with the hydrophilic surrounding and the solvent-exposed region. So far, PD-L1 inhibitors modified with taurine and homotaurine have been reported with increased potency and together with additional amino side chains, improved water solubility [78]. For generating a radiotracer, we introduced a DOTA chelator, fulfilling a dual role: as a cage for hosting the radiometal and for providing extra hydrophilicity to the molecule. Furthermore, DOTA is a versatile chelator, which allows labeling with different PET and therapeutic radionuclides, e.g., ^68^Ga or ^177^Lu. To prevent negative impact on the binding affinity, the chelator was kept from the binding site through a linker. For accessing the designed molecules, we developed a new convergent route, which has the advantage of rapid synthesis and use of versatile building blocks. As a result, we obtained six different PD-L1 ligands in a first series, bearing either a bromobiphenyl or a dimethyl biphenyl core and a bis(sulfonic acid) moiety as the solubilizer unit of the molecule. Furthermore, different linkers were synthesized and incorporated between the chelator and biphenyl core. Our work using ^64^Cu-labeled biphenyl small-molecule inhibitors of PD-L1 shows the principal suitability of these compounds as radioligands and starters for further modification. In vitro evaluation, using saturation binding in PC3 cells stably transduced with human PD-L1, showed saturable binding, which could be displaced by several PD-L1 inhibitors described in the literature. Nonspecific binding, assessed with a 300-fold excess of BMS1166 (**1**), was very low, further supporting in vitro suitability and in vivo testing. Affinities across derivates showed a variation of approximately factor 10 (59–585 nM), which was also observed for B_max_. The latter could indicate slight differences in binding mode, e.g., some molecules providing better recruitment/stabilization of the PD-L1 dimer, as described by molecular docking simulations [79]. However, all of our nine radioligands still exhibit a relatively high lipophilicity, which was also observable in vitro, as exemplified by increased retention on tubes and plates. This is in contrast to the obtained log*D*_7.4_ values, which ranged between −2.73 and −3.50 for the ^64^Cu-labeled radioligands. The highest affinities in saturation binding (with BSA) were determined to be below 100 nM. Structural differences apparently affect affinity, as demonstrated by the contrast between compound [^64^Cu]Cu-**42a** (585 nM) and [^64^Cu]Cu-**43b** (59.9 nM). Generally, the data show that all dimethyl biphenyl derived radioligands ([^64^Cu]Cu-**43a**–**c**) exhibited significantly higher binding affinities as compared to the bromobiphenyl derivatives ([^64^Cu]Cu-**42a**–**c**). Interestingly, the affinity determined for compound [^64^Cu]Cu-**43b** was comparable to previously described small-molecule biphenyl active structure radioligands, e.g., [^18^F]FLN with 65 nM [34] and [^18^F]LG-1 with 63 nM [35], using A375 cells transfected with PD-L1. Furthermore, labeled peptides were found to exhibit similar affinities, e.g., TPP with 95 nM [29] and very low specificity [80]. We also performed a comparison to the previously described cyclic peptide WL12 [25,27,81], with IC_50_ values of 2.9 to 23 nM, reported using fluorescence resonance energy transfer and a K_D_ of 3 nM using SPR [68]. While assay differences complicate direct comparison, the in vitro affinity of ^64^Cu-labeled DOTAGA-WL12 was similar to compound [^64^Cu]Cu-**43b** in our hands. However, B_max_ was approximately 10 times lower for [^64^Cu]Cu-**43b**, once again pointing toward potentially different binding modes. As the range of the employed concentrations in our saturation assay is rather wide, a certain bias toward a worse affinity could explain this. Compound [^64^Cu]Cu-**43b** with a K_D_ value of 60 nM served as the basis for further modifications by replacing the SO_2_Me group in the pyridine moiety with a CN group and by replacing the chlorine atom at the central aryl with bromine or iodine, resulting in three additional ligands. The water solubility in this series decreased from chlorine derivative [^64^Cu]Cu-**56a** over bromine derivative [^64^Cu]Cu-**56b** to iodine derivative [^64^Cu]Cu-**56c**. In the same manner, the binding affinities dropped from 128 nM for [^64^Cu]Cu-**56a** to 285 nM for [^64^Cu]Cu-**56c**. This is in contrast to the findings of Konieczny et al., who claimed that bromine at the central aryl moiety improves the affinities [47]. However, the receptor density values (B_max_) are significantly higher for the iodinated ligand [^64^Cu]Cu-**56c** compared to the bromine and chlorine radioligand. The structure of our radioligands included sulfonic acids as well as iodine, which are both known to possess albumin binding properties [82,83]. Therefore, we performed albumin binding experiments with the ^177^Lu-labeled ligands in human serum. The results clearly confirmed that all compounds bind to human serum albumin, presumably due to the presence of the sulfonic acids. Especially the bromine and iodine-containing compounds showed almost complete binding to albumin within one hour of incubation. These findings are corroborated by the real-time radioligand binding derived from our compounds. Here, the binding curve in the presence of BSA did not reflect the expected pattern of ligand–target interaction, usually exemplified by a clear increase in signal (association), followed by flattening of the curve (either due to reaching equilibrium or saturation). We speculate that this pattern mainly reflects binding to albumin, as the association rate constant of different drugs to various albumin binding sites has been described as very fast. Depending on the employed method, it is in the range of ~2 × 10^5^ M^−1^ s^−1^ for warfarin [84,85] and can surpass this by orders of magnitude, e.g., for copper(II), hematin, and Iophenoxate [86]. Except for the high binding to albumin, the in vitro affinities of our compounds are in a similar range to the currently clinically trialed cyclic peptide WL12 [30]. This peptide showed an affinity of 0.79 nM (DOTAGA-WL12) in our real-time assay, which is close to a previously reported value of 3 nM for [^64^Cu]-NOTA-WL12 using SPR [68].

In contrast to the in vitro evaluation, in vivo behavior of both first and second series of compounds labeled with ^64^Cu was less favorable. PET/CT was performed in mice bearing both PD-L1 overexpressing and mock construct PC3 tumors. IHC confirmed extensive PD-L1 staining in PD-L1 overexpressing and an almost complete absence in mock construct tumors, similar to what has been reported for CHO cells [27,81]. In comparison to WL12 [27,81], our tracer candidates showed a relatively slow tumor uptake and clearance for the first compound series ([^64^Cu]Cu-**42a**–**c** and [^64^Cu]Cu-**43a**–**c**), with low SUV_max_ values in the first two hours, continuously increasing up to 24 h post injection. In order to match the slow pharmacokinetics, ^64^Cu with its longer physical half-life was used as a PET nuclide instead of initially planned ^68^Ga. While tumor uptake was slightly faster for the second compound series, it was still found to be slow and apparently not finished by 24 h. The gradual loss of contrast between PD-L1 and mock tumors, especially after 24 h found for most compounds except [^64^Cu]Cu-**42c** and [^64^Cu]Cu-**43a**–**c**, is not fully understood. It could either indicate nonspecific binding (although unlikely, given the early contrast for several of our compounds) or metabolization. It could also be speculated that this represents the slow accumulation of the albumin–ligand complex over time. In any case, this has not been observed for other PD-L1 ligands, e.g., WL12. 

Blocking experiments performed with 100 nmol of unlabeled compound showed a moderate blocking effect for **43b**, confirming a certain degree of specificity. To achieve a more pronounced blocking effect, a much higher excess of nonradioactive compound would probably have been required, but was not feasible synthetically. Again, the albumin binding likely affected the availability for PD-L1 negatively, explaining the low blocking effect by the unlabeled compound.

Interestingly, slightly increased blood circulation time can be assumed for most compounds, especially when compared to compound [^64^Cu]Cu-**42a** and [^64^Cu]Cu-**42b**. This is apparent from the PET images and, at least for second-series compounds, could be attributed again to albumin binding capacity [83], as recently shown for other ligands in vivo [87].

Clearance of all compounds was primarily via the hepatobiliary excretion route, possibly further enhanced by transchelation of copper by liver proteins in rodents [88]. This is in contrast to the primarily renal clearance found for peptides [25,27,29,81,89] and small molecules [34,35], possibly influenced by the still relatively high lipophilicity of our compounds. 

The reasons for the in vivo behavior observed for both first and second series radioligands is not completely clear, but likely are a combination of albumin binding capacity, longer blood circulation time, and lipophilicity. While affinity certainly plays a role, the difference observed in K_D_ of our compounds does not apparently correlate with in vivo behavior. Other factors influencing this might be related to binding mode, internalization, or even pH in the tumor, with the latter being an already described factor [90].

## 4. Conclusions

In summary, we have developed novel PD-L1 ligands for labeling with ^68^Ga and ^64^Cu based on two PD-L1 small-molecule inhibitors reported in the patent literature. The structural design was founded on a PD-L1-inhibitor cocrystal structure. For accessing the radiolabeling precursors, a new convergent synthetic strategy was developed, and water solubility was increased by attaching sulfonic acid moieties and a piperazine linker. In a saturation binding assay in the presence of BSA, binding affinities below 100 nM were achieved for radioligands bearing the dimethylbiphenyl core and the piperazinylpropyl linker. However, due to extensive albumin binding properties of our compounds, the binding affinities toward PD-L1 are likely masked. Real-time radioligand binding in the absence of BSA indeed revealed affinities in a low, single digit nanomolar range for most of the reported tracer candidates. All radiolabeled compounds were stable in human serum and liver microsomes and showed moderate to low tumor uptake in small animal PET/CT. They exhibited hepatobiliary clearance, and some compounds exhibited a long circulation time, likely due to serum albumin binding of the sulfonates. Optimization of the binding affinities and the pharmacokinetic profile by increasing the hydrophilicity of these radioligands and replacement of DOTA by NODAGA for higher complex stability are currently ongoing.

## 5. Materials and Methods

### 5.1. Organic Synthesis

#### 5.1.1. General Remarks

All manipulations requiring exclusion of oxygen and moisture were carried out in heat-gun dried flasks under argon gas atmosphere using the Schlenk technique. All chemicals and solvents were purchased from Sigma-Aldrich Laborchemikalien GmbH (Saint Louis, MO, USA), abcr GmbH (Karlsruhe, Germany), and Acros Organics and were used without further purification. Deuterated solvents were used from Deutero GmbH. The dry solvents dimethylformamide, dichloromethane, methanol, and tetrahydrofuran were purchased from Sigma-Aldrich Laborchemikalien GmbH in Sure/Seal™ bottles. For thin-layer chromatography (TLC) analysis, Merck (Rahway, NJ, USA) precoated plates (silica gel 60 F_254_, Art 5715, 0.25 mm) were used and were visualized with UV light or KMNO_4_ stain. Attenuated Total Reflectance Fourier Transform Infrared (ATR-FTIR) spectra were recorded with a Thermo Scientific (Waltham, MA, USA) Nicolet iS5 and the intensity of bands was described with weak (w), medium (m), strong (s), and broad signals (br). All synthesized compounds were characterized by NMR spectroscopy using an Agilent DD2-400 MHz NMR or Agilent DD2-600 MHz NMR spectrometer with ProbeOne. Chemical shifts of ^1^H and ^13^C signals were reported in parts per million using TMS as internal standard at 25 °C. Spectra were calibrated using the respective solvent signal. High-resolution mass spectra (HRMS) were obtained on a TOF (Q-TOF MS) using electrospray ionization: Agilent 1260 Infinity II HPLC (Santa Clara, CA, USA; pump G7111B, autosampler G7129A, column oven G7116N, UV detector G7717C, eluent acetonitrile/water acidified with 0.1% formic acid, bypass mode) coupled to UHD Accurate Mass Q-TOF LC MS G6538A. Analytical HPLC was performed on VWR Hitachi (Tokyo, Japan) with acetonitrile/water (0.1% TFA each) as mobile phase on an Agilent C18 column (Agilent Zorbax 300SB-C18, 100 mm × 4.6 mm). Preparative and semipreparative reversed HPLC separations were performed on the Knauer Azura (Berlin, Germany) with acetonitrile/water (0.1% TFA each) as mobile phase on Phenomenex (Torrance, CA, USA) Synergi hydro-RP 4 μm 80 Å, 21.2 × 250 mm and Zorbax SB C-18 5 μm 80 Å, 9.4 × 250 mm, respectively. All compounds were >95% pure by HPLC.

#### 5.1.2. Synthetic Procedures

Synthetic procedures including characterization are provided in the supplementary information (Supplementary Figures S1–S233).

### 5.2. Visualization of PD-L1 Cocrystals

The X-ray cocrystal structure of PD-L1 with BMS-1166 was taken from the Protein Data Bank (PDB code: 6R3K). Molecular graphic manipulation and visualization were performed with PyMOL 2.5.0 and Discovery Studio Visualizer 4.0.

### 5.3. Radiochemistry

For the radiolabeling experiments, 1 mM stock solution in ultrapure water of all nine radiotracers were produced. Each experiment, radiolabeling optimization, in vitro assay, or in vivo experiment was conducted with 8 nmol of the respective ligand for all three radionuclides ^64^Cu, ^68^Ga and ^177^Lu. 

#### 5.3.1. Gallium-68

^68^Ga was obtained by eluting a ^68^Ge/^68^Ga generator (iThemba Labs, Somerset West, South Africa) with 1 M HCl providing between 1000 and 1500 MBq of ^68^Ga in a volume of approx. 300 µL ^68^Ga. Depending on the used amount of ^68^Ga and the elution efficiency, the reaction was performed in volumes between 100 and 300 µL of a 1 M HEPES solution to adjust the pH of 4.

#### 5.3.2. Copper-64

^64^Cu was produced by the nuclear reaction ^64^Ni(p,n) → ^64^Cu in-house with a cyclotron, which is described in detail elsewhere [91]. ^64^Cu was obtained in a solution of 0.1–0.01 M HCl and radiolabeling was performed in approx. 200 µL of a 1 M HEPES-solution, which was previously adjusted to pH 4 with 1 M HCl. 

#### 5.3.3. Lutetium-177

^177^Lu was purchased at ITM GmbH (Garching, Munich, Germany), which was produced by ^176^Yb(n,γ)^177^Yb → ^177^Lu nuclear reaction. Aliquots of the 0.04 M HCl solution were drawn and reacted with the corresponding ligand in approx. 200 µL of a 1 M HEPES-solution which was prior adjusted to pH 4 with 1 M HCl. 

#### 5.3.4. Radiolabeling Procedure

The radiolabeling procedure was optimized and applied to all radionuclides and radioligands. A mixture of the ligand in the HEPES solution for the radionuclide (vide supra) was prepared in 1.5 mL Protein LoBind^®^ Eppendorf Tubes (Hamburg, Germany). The solution of the corresponding radionuclide was added and then the tube was incubated at 300 rpm at 95 °C for 15 min. After cooling down for 3 min, a small aliquot was placed on iTLC-SG chromatography paper (stationary phase). A 0.1 M citrate solution pH 4 (adjusted with 1 M NaOH) was used as the mobile phase. The chromatography paper was scanned using a radioisotope thin layer analyzer (Rita Star, Elysia-Raytest GmbH, Straubenhardt, Germany). Labeling efficiency was evaluated by the proportion of unbound radiometal (*R*_f_ = 0) and radio–metal complex (*R*_f_ = 0.8) by integration of the respective areas. For all combinations of radionuclides and radioligands, labeling efficiencies of >95% were obtained.

### 5.4. Log D_7.4_ Determination

*n*-Octanol/water distribution coefficients (log *D*_7.4_) were determined using the shake-flask method and performed in triplicate. Twenty µL of the reaction mixture was added to a 1.5 mL Eppendorf Tube containing 580 µL of 1× PBS (pH 7.4) and 600 µL *n*-octanol. The two-phase system was shaken at room temperature with 1500 rpm for 5 min. After centrifugation, an aliquot of each phase was withdrawn, and the count rates were measured in a γ-counter (ISOMED2160 sodium iodide crystal detector). The average was calculated, corrected for background activity, and the log *D*_7.4_ value was calculated according to the formula:

$$\log D_{7.4}=\log\left(\frac{A_{n-octanol}}{A_{PBS\;}}\right).$$

### 5.5. Stability Studies in Different Buffer Systems

The corresponding radiotracer was labeled with approx. 50 MBq of ^177^Lu in 200 µL HEPES solution (pH 4) as described above. After successful labeling, the solution was transferred in 500 µL of 1 M HEPES solution (pH 4) or 500 µL of 1× PBS solution (pH 7), respectively, and incubated at room temperature for up to 7 d. A 50 µL aliquot was analyzed by radio-HPLC (PerkinElmer, Waltham, MA, USA, system E) after 1 h, 24 h, 48 h, 72 h, and 7 d of incubation. Evaluation and graphical plotting were performed with OriginPro^®^ 9.0.

#### 5.5.1. Stability Studies in Human Serum

##### By Radio-TLC

The corresponding radiotracer was labeled with approx. 50 MBq of ^177^Lu in 200 µL HEPES solution (pH 4) as described above. After successful labeling, the reaction mixture was diluted 1:10 with human serum. The solution was incubated at 37 °C with shaking at 500 rpm. After 1 h, 24 h, 48 h, 72 h, and 7 d, a 1 µL aliquot was analyzed by radio-TLC as described above. Evaluation and graphical plotting were performed with OriginPro^®^ 9.0.

##### By Precipitation and Radio-HPLC

For investigation by radio-HPLC, a chloroform–methanol precipitation was carried out at each time point [92]. An aliquot (100 µL) of the serum-containing incubation reaction was mixed with 400 µL of MeOH. Then, 100 µL of chloroform were added and the sample was vortexed well. For phase separation, 300 µL of ultrapure water were added, and the sample was vortexed vigorously and centrifuged for 2 min at 14,000× *g*. The upper, aqueous layer was carefully removed and collected. To the lower chloroform phase and the interphase containing the precipitated proteins, 400 µL of MeOH was added. The sample was mixed and centrifuged again for 3 min at 14,000× *g* to pellet the proteins. The supernatant was removed, combined with the supernatant obtained in the previous step, and again centrifugated for 3 min at 14,000× *g* to remove remaining proteins. Afterward, the clear supernatant was analyzed by radio-HPLC (system E). The peak areas were normalized by measuring the activity with the gamma counter (HIDEX Deutschland Vertrieb GmbH, Mainz, Germany) at the corresponding time point. Evaluation and graphical plotting were performed with OriginPro^®^ 9.0.

##### Radiotracer Stability in the Presence of Liver Microsomes

The stability assessment using human liver microsomes was performed as described recently with slight modifications [93]. All microsomal incubations had a final volume of 250 µL, and the final concentration of each component was provided in brackets. At first, the corresponding ligand was labeled with ^177^Lu as described above, but with a total volume of 50 µL, a ligand concentration of 10 mM and a total activity of approx. 100 MBq. 2.5 µL of the labeled solution (1 µM ligand concentration) were preincubated at 37 °C for 3 min at 500 rpm in 210 µL of PBS-buffer pH 7.0 containing 12.5 µL (1 mg/mL) human liver microsomes (Gibco™, Waltham, MA, USA, 50 donors, pooled). Then, 25 µL (2 mM in PBS) of preincubated NADPH was added to initiate the reaction, and the mixture was shaken gently. To terminate the reaction after designated time intervals (0, 2, 5, 10, 15, 20, 30, 40, 50, and 60 min), 20 µL aliquots were added to 80 µL of cold acetonitrile (−20 °C). The samples were shaken vigorously, cooled for 4 min on ice, and centrifuged at 4 °C for 10 min at 14,000× *g*. Ninety µL of the supernatants were analyzed by radio-HPLC using system E.

Testosterone (20 µM) was incubated similarly to the protocol described above as positive control. Its conversion was confirmed by HPLC analyses with UV detection.

### 5.6. Serum Binding Studies

The corresponding ligand was labeled with approx. 50 MBq of ^177^Lu in 200 µL HEPES solution (pH 4) as described above. After successful labeling**,** 40 µL were mixed with 500 µL of human serum or 500 µL of 1 M HEPES solution (control), respectively. The samples were incubated at 37 °C with 500 rpm and were analyzed by size-exclusion chromatography (Jasco Germany GmbH, Pfungstadt, System F) and native discontinuous polyacrylamide gel electrophoresis (PAGE). 

For size-exclusion chromatography, 20 µL aliquots were injected into system F and the obtained peak areas of serum-bound ligands and unbound ligands were used to calculate the percentage of radioligand bound to serum proteins. Evaluation and graphical plotting were performed with OriginPro^®^ 9.0.

For nonreducing, nondenaturing separation of samples by native PAGE, gels with acrylamide concentrations of 10% in the resolving and 5% in the stacking gel were prepared. For 10 mL of the resolving gel, 4.1 mL of ultrapure water, 3.3 mL of 30% acrylamide/bis-acrylamide solution, 2.5 mL 1.5 M TRIS-buffer pH 8.8, 4 µL of *N*,*N*,*N’*,*N’*-tetramethylethylenediamine (TEMED), and 100 µL of 10% ammonium peroxodisulfate (APS) were used. For the preparation of the stacking gel, 6.9 mL of ultrapure water, 1.7 mL of 30% acrylamide/bis-acrylamide solution, 1.25 mL of TRIS-buffer pH 6.8, 10 µL of TEMED, and 100 µL 10% APS were used. 10 µL aliquots of the serum-containing samples were mixed with 10 µL of native sample buffer (Bio-Rad Laboratories, Hercules, CA, USA) and 2 µL of each sample were loaded into each well of the gel. The native PAGE was run at room temperature and 80 V until the dye front reached the resolving gel and then increased up to 160 V. After electrophoresis, the gel was washed for 1 min in ultrapure water and imaged using the phosphorimager Amersham Typhoon 5 Scanner (Cytiva Europe GmbH, Freiburg, Germany).

### 5.7. Cell Lines and Cell Culture

PC3 cells were transduced using a lentiviral vector to stably overexpress PD-L1 (PC3-PD-L1) along with cells transduced with a control plasmid (PC3-mock).

Following transduction, cells were cultured in RPMI-1640 medium (#R0883, Sigma Aldrich, Germany), supplemented with (*v*/*v*) 10% fetal bovine serum (#S0615), 1% penicillin/streptomycin (#15140-122, Gibco, Waltham, MA, USA), 1% nonessential amino acids, and 1% alanine/glutamine (#M7145 and #G8541, respectively; Sigma Aldrich) under normoxic (5% CO_2_; 37 °C) conditions. Cells were passaged upon reaching ~90% confluency.

For radioligand binding studies, cells were trypsinized (0.05% Trypsin-EDTA, Gibco, Waltham, MA, USA) and counted using a CASY1 cell counter (Model TT, Schaerfe System, Reutlingen, Germany). Cells were subsequently diluted to ~160,000 cells/mL in medium and seeded to 48 well plates (Falcon Multiwell #353078, ThermoFisher, Karlsruhe, Germany) for at least 2 days for endpoint saturation studies. For real-time radioligand binding, between 40,000 and 500,000 cells/mL were seeded into one side of petri dishes (3 mL, Nunclon, # 150350, ThermoFisher Germany) ~24 h before the experiments.

### 5.8. Saturation Binding Studies

To determine the dissociation constant (K_D_) and number of binding sites (B_max_) of new PD-L1 small-molecule ligands, saturation binding was performed in at least 3 independent experiments. PC3 cells stably transduced with human PD-L1, cultured under conditions described above, were brought to room temperature followed by cooling on ice (each 15 min). The medium was removed and replaced with 200 µL assay buffer [PBS + 2.5% (*w*/*v*) bovine serum albumin (BSA; #1ETA, Carl Roth, Karlsruhe, Germany)] for total binding (TB) conditions or assay buffer containing 300 µM of **1** (in DMSO, resulting in 0.03% *v*/*v* [48], synthesized according to Supplementary Scheme S1) for assessment of nonspecific binding (NSB). BSA was found to drastically reduce nonspecific binding to the polystyrene material of the 48 well plates of some radiotracer candidates.

After 15 min preincubation for TB/NSB, 200 µL of each eight serial 1:1 dilutions (500 to 3.91 nM) of the respective radioligand were added (in triplicate). Cells were incubated for 90 min on ice, followed by rapid removal of incubation medium and washing in ice-cold assay buffer (3 × ~1 min). Cells were then lysed with 500 µL 0.1 M NaOH + 1%SDS. Radioactivity (counts per minute, CPM) of 400 µL of the lysate was measured in a gamma counter (Perkin Elmer Wizard 1480) using the nuclide-specific energy window. Additionally, we determined the binding to polystyrene in 400 µL of lysate and activity of stock solutions (50 µL). All counts were decay corrected to a reference time (end of radiolabeling). As BSA artificially increases protein content, an additional plate was subjected to the same conditions (preincubation, incubation, and washing) using PBS without BSA for each experiment. Protein content of the lysates (24 or 48 wells) was determined by BCA assay and a mean value (µg/mL) used for this particular dataset (plate). From CPM measurements, final values (pmol/mg protein) were calculated using mean protein content and molar activity. 

Processed data were then subjected to nonlinear iterative curve fitting (GraphPad Prism 9) to yield B_max_ (in pmol/mg protein) and K_D_ (in nM).

### 5.9. Real-Time Radioligand Binding Studies

For binding kinetics (association rate constant k_a_ and dissociation rate constant k_d_) and dissociation constant (K_D_), a real-time assay system (LigandTracer Yellow, Ridgeview Instruments AB, Uppsala, Sweden) was employed. In this system, the petri dish containing the cells in medium (3 mL) was located on one side, while radioactivity was detected on the opposite side. The dish rotated on an inclined base, which allowed continuously measuring two or more alternating parts of the dish for bound radioligand (cells) against background (no cells). Binding was performed at room temperature (using CO_2_-independent medium, Gibco #18045088, ThermoFisher Germany) with or without BSA (2.5%). Association was determined through incubation with two concentrations (10 and 40 nM, 90 min each) of our ^64^Cu-labeled PD-L1 small molecules, binding to PC3 PD-L1 overexpressing cells. This was followed by replacement with fresh medium and measurement of dissociation for at least 2 h. This approach provided reliable kinetic measurements as previously shown [94]. Data were acquired in decay-corrected counts per second (CPS). Binding data were then evaluated using TraceDrawer (1.9.2, Ridgeview Instruments AB, Uppsala, Sweden). Traces were imported, potential spikes (>100% sudden increases in CPS over previous datapoint) removed, and each trace was normalized to its own baseline (=0%) and highest value (=100%). The single experiment in the presence of 2.5% BSA was processed in the same way and analyzed for the same parameters, however without replicates.

### 5.10. PD-L1 Immunostaining

In order to confirm PD-L1 expression in grown tumors (see below), random samples (not subjected to PET) were taken after animals approached endpoints. Prior to this, positive (human placenta) and negative controls (omission of primary/secondary antibodies) were performed in a separate experiment, confirming antibody specificity and staining conditions. 

Tumors were excised and immersion-fixed in 4% paraformaldehyde for 48 h, followed by transfer to PBS (+0.05% sodium azide; *w*/*v*). Tissue was then processed, paraffin-embedded, and 4 µm thick sections cut and placed on SuperFrost+ slides. On the day of immunostaining, sections were dewaxed in RotiHistol (Carl Roth, Karlsruhe, Germany), rehydrated in a descending series of ethanol (100, 96, 85, 70, 50% (*v*/*v*), H_2_O), and antigen retrieval performed [10 mM citrate buffer (pH 6.0), in a steam bath]. Tissue was permeabilized and endogenous peroxidases quenched with 3% (*v*/*v*) H_2_O_2_ in Tris-buffered saline [with 0.1% Tween 20 (TBS-T], followed by blocking using 10% (*w*/*v*) fetal bovine serum in TBS-T. Sections were then incubated with the primary antibody [monoclonal α-human PD-L1, Rabbit IgG #E1L3N Cell Signaling (Danvers, MA, USA), 874 µg/mL] at 1:20,000 overnight at 4 °C in a humidified chamber. Primary antibody binding was detected subsequently using a 2-step polymer detection kit (SuperVision 2 HRP-Polymer, DCS Innovative Diagnostik-System, Hamburg, Germany) with 3,3′-diaminobenzidine (DAB) as chromogen, according to the manufacturer’s protocol. Sections were then counterstained with Mayer’s hematoxylin, coverslipped, and imaged at 10× on an AXIO Imager A1 microscope (Carl Zeiss Microscopy, Oberkochen, Germany).

### 5.11. Animals, Biodistribution and PET Imaging

Male athymic NMRI-nude mice (Rj:NMRI-Foxn1nu, Janvier Labs, Le Genest-Saint-Isle, France) between 8 and 22 weeks of age were used. Under general anesthesia, [~10% desflurane (Baxter, Deerfield, IL, USA) in 30 vol% oxygen + air] and warming, animals were subcutaneously injected with 3–5 × 10^6^ PC3 PD-L1 and PC3 mock cells [in 50 µL PBS + 50 µL Matrigel (Corning, Glendale, CA, USA)] into the right and left thigh, respectively. Tumor growth was monitored three times a week by caliper measurements and mice with tumor sizes above 7 mm were included in the experiments. 

For PET studies, general anesthesia was induced as described above. PET and X-ray computed tomography (CT) were performed in a dedicated small animal nanoScan PET/CT scanner, with 4 animals imaged simultaneously (Mediso, Budapest, Hungary). CT images were employed for attenuation correction and anatomical referencing. Animals received three individual scans corresponding to 0–2 h (dynamic), 4.5 h, and 24 h (static) post injection.

PD-L1 radiotracer candidates (in 300 µL sterile 0.9% NaCl/HEPES buffer, pH 6–7, between 7 and 12 MBq, Molar activities > 12 GBq/µmol)) were delivered over 30 sec i.v. (lateral tail vein) and PET acquisition started simultaneously.

Three-dimensional list mode data were binned using the 400–600 keV energy window and sorted into 36 time frames (15 × 10 s, 5 × 30 s, 5 × 60 s, 4 × 300 s, 3 × 600 s, 3 × 1200 s). Time frames were reconstructed using the Tera-Tomo^TM^ 3D algorithm applying a voxel size of 0.4 mm and corrections for decay, scatter, and attenuation. Images were postprocessed and analyzed using Rover (ABX GmbH, Radeberg, Germany) and displayed as maximum intensity projection (MIPs) at indicated time points and scaling.

Three-dimensional volumes of interest were delineated applying fixed thresholding at 35% of the measured maximum intensity. Standardized uptake values (SUV = [MBq detected activity/mL tissue]/[MBq injected activity/g body weight], mL/g) were determined in selected volumes of interest, among these PD-L1 and mock tumors and metabolizing organs.

For radiotracer biodistribution studies, tumor-bearing animals were i.v. injected with approximately 2 MBq of ^64^Cu-labeled compound 4 via the lateral tail vein. At three timepoints (1, 4 and 24 h p.i.), animals were euthanized under general anesthesia, followed by blood (heart puncture) and urine collection. All major organs were dissected, weighed, and radioactivity determined in a gamma counter (Perkin Elmer Wizard 1480), along with radioactive standards.

### 5.12. Data and Statistical Analysis

Data are presented as mean ± standard deviation. All statistical procedures were performed using GraphPad Prism, version 9.0 (GraphPad Software Inc., San Diego, CA, USA).

## 6. Patents

F.K., K.K. and S.S. are inventors of the European patent application EP21212444 for biphenyl-based PD-L1-targeting agents for imaging and therapy in nuclear medicine, which was submitted on 6 December 2021. No other potential conflicts of interest relevant to this article exist. 

## Figures and Tables

**Table 1:** Summary of in vitro data for ^64^Cu-Labeled PD-L1 Radioligands and their respective LogD_7.4_ values.

Compound	K_D_ (nM) *^a^*	B_max_ (pmol/mg) *^a^*	log *D*_7.4_ *^b^*
[^64^Cu]Cu-**42a**	585 ± 53.0	11.7 ± 0.77	−2.73 ± 0.04
[^64^Cu]Cu-**42b**	487 ± 58.6	8.91 ± 0.63	−3.03 ± 0.05
[^64^Cu]Cu-**42c**	351 ± 8.31	5.85 ± 1.72	−3.47 ± 0.10
[^64^Cu]Cu-**43a**	123 ± 17.3	13.5 ± 1.38	−2.75 ± 0.04
[^64^Cu]Cu-**43b**	59.9 ± 6.05	5.43 ± 0.63	−3.14 ± 0.02
[^64^Cu]Cu-**43c**	71.3 ± 13.2	7.04 ± 0.63	−3.50 ± 0.01
[^64^Cu]Cu-**56a**	128 ± 17.2	20.9 ± 2.82	−2.99 ± 0.02
[^64^Cu]Cu-**56b**	135 ± 3.37	8.28 ± 0.18	−3.48 ± 0.03
[^64^Cu]Cu-**56c**	300 ± 26.8	49.6 ± 1.78	−2.99 ± 0.02
[^64^Cu]Cu-DOTAGA-WL12 [27]	50.2 ± 7.2	56.7 ± 3.87	−2.57 ± 0.02

*^a^* Affinity (equilibrium dissociation constant K_D_) and maximum number of binding sites (B_max_) as determined under identical conditions in a saturation binding assay (addition of 2.5% BSA) using viable and genetically modified PD-L1-positive PC3 cells. Data are mean ± (SD), derived from at least three independent experiments (each in triplicate) *^b^* Data are mean ± (SD), derived from three separate shake flask experiments.

## Data Availability

All data are shown in the study and in the Supplementary Material.

## Supplementary Materials

The following supplementary information can be downloaded at: https://www.mdpi.com/article/10.3390/cancers15092638/s1. Reference [95] is cited in the supplementary materials. Copies of ^1^H and ^13^C NMR, IR and HR-MS spectra as well as HPLC chromatograms of intermediate and final compounds. Radio-HPLC chromatograms, stability assay and K_D_ measurement result. Micro-PET images of all final radioligands. Scheme S1: Synthesis of PD-L1 inhibitor **1** (BMS-1166) with new synthetic strategy. Figure S1: ^1^H NMR spectrum (CDCl_3_, 600 MHz, 298 K) of compound **6**. Figure S2: ^13^C NMR spectrum (CDCl_3_, 151 MHz, 298 K) of compound **6**. Figure S3: ^1^H NMR spectrum (CDCl_3_, 400 MHz, 298 K) of compound **10**. Figure S4: ^13^C NMR spectrum (CDCl_3_, 101 MHz, 298 K) of compound **10**. Figure S5: ^1^H NMR spectrum (DMSO-d_6_, 400 MHz, 298 K) of compound **11**. Figure S6: ^13^C NMR spectrum (DMSO-d_6_, 101 MHz, 298 K) of compound **11**. Figure S7: ^1^H NMR spectrum (CDCl_3_, 600 MHz, 298 K) of compound **17**. Figure S8: ^13^C NMR spectrum (CDCl_3_, 151 MHz, 298 K) of compound **17**. Figure S9: ^1^H NMR spectrum (CDCl_3_, 400 MHz, 298 K) of compound **21**. Figure S10: ^13^C NMR spectrum (CDCl_3_, 101 MHz, 298 K) of compound **21**. Figure S11: ^1^H NMR spectrum (DMSO-d_6_, 400 MHz, 298 K) of compound **22**. Figure S12: ^13^C NMR spectrum (DMSO-d_6_, 101 MHz, 298 K) of compound **22**. Figure S13: ^1^H NMR spectrum (CDCl_3_, 400 MHz, 298 K) of compound **28a**. Figure S14: ^13^C NMR spectrum (CDCl_3_, 101 MHz, 298 K) of compound **28a**. Figure S15: ^1^H NMR spectrum (CDCl_3_, 400 MHz, 298 K) of compound **29a**. Figure S16: ^13^C NMR spectrum (CDCl_3_, 101 MHz, 298 K) of compound **29a**. Figure S17: ^1^H NMR spectrum (methanol-d_4_, 400 MHz, 298 K) of compound **30a**. Figure S18: ^13^C NMR spectrum (methanol-d_4_, 101 MHz, 298 K) of compound **30a**. Figure S19: ^1^H NMR spectrum (DMSO-d_6_, 400 MHz, 298 K) of compound **33b**. Figure S20: ^13^C NMR spectrum (DMSO-d_6_, 101 MHz, 298 K) of compound **33b**. Figure S21: ^1^H NMR spectrum (CDCl_3_, 600 MHz, 298 K) of compound **26**. Figure S22: ^13^C NMR spectrum (CDCl_3_, 151 MHz, 298 K) of compound **26**. Figure S23: ^1^H NMR spectrum (CDCl_3_, 600 MHz, 298 K) of compound **27**. Figure S24: ^13^C NMR spectrum (CDCl_3_, 151 MHz, 298 K) of compound **27**. Figure S25: ^1^H NMR spectrum (CDCl_3_, 600 MHz, 298 K) of compound **28b**. Figure S26: ^13^C NMR spectrum (CDCl_3_, 151 MHz, 298 K) of compound **28b**. Figure S27: ^1^H NMR spectrum (CDCl_3_, 600 MHz, 298 K) of compound **29b**. Figure S29: ^1^H NMR spectrum (methanol-d_4_, 400 MHz, 298 K) of compound **30b**. Figure S30: ^13^C NMR spectrum (methanol-d_4_, 101 MHz, 298 K) of compound **30b**. Figure S31: ^1^H NMR spectrum (DMF-d_7_, 400 MHz, 298 K) of compound **33b**. Figure S32: ^13^C NMR spectrum (DMF-d_7_, 101 MHz, 298 K) of compound **33b**. Figure S33: ^1^H NMR spectrum (CDCl_3_, 400 MHz, 298 K) of compound **S-5**. Figure S34: ^13^C NMR spectrum (CDCl_3_, 101 MHz, 298 K) of compound **S-5**. Figure S35: ^1^H NMR spectrum (CDCl_3_, 400 MHz, 298 K) of compound **35**. Figure S36: ^13^C NMR spectrum (CDCl_3_, 101 MHz, 298 K) of compound **35**. Figure S36: ^13^C NMR spectrum (CDCl_3_, 101 MHz, 298 K) of compound **35**. Figure S37: ^1^H NMR spectrum (CDCl_3_, 400 MHz, 298 K) of compound **S-6**. Figure S38: ^13^C NMR spectrum (CDCl_3_, 101 MHz, 298 K) of compound **S-6**. Figure S39: ^1^H NMR spectrum (methanol-d_4_, 400 MHz, 298 K) of compound **S-7**. Figure S40: ^13^C NMR spectrum (methanol-d_4_, 101 MHz, 298 K) of compound **S-7**. Figure S41: ^1^H NMR spectrum (methanol-d_4_, 400 MHz, 298 K) of compound **39**. Figure S42: ^13^C NMR spectrum (methanol-d_4_, 101 MHz, 298 K) of compound **39**. Figure S43: ^1^H NMR spectrum (DMSO-d_6_, 400 MHz, 298 K) of compound **40a**. Figure S44: ^13^C NMR spectrum (DMSO-d_6_, 101 MHz, 298 K) of compound **40a**. Figure S45: ^1^H NMR spectrum (DMSO-d_6_, 400 MHz, 298 K) of compound **40b**. Figure S46: ^13^C NMR spectrum (DMSO-d_6_, 101 MHz, 298 K) of compound **40b**. Figure S47: ^1^H NMR spectrum (DMSO-d_6_, 400 MHz, 298 K) of compound **40c**. Figure S48: ^13^C NMR spectrum (DMSO-d_6_, 101 MHz, 298 K) of compound **40c**. Figure S49: ^1^H NMR spectrum (DMSO-d_6_, 400 MHz, 298 K) of compound **41a**. Figure S50: ^13^C NMR spectrum (DMSO-d_6_, 101 MHz, 298 K) of compound **41a**. Figure S51: ^1^H NMR spectrum (DMSO-d_6_, 400 MHz, 298 K) of compound **41b**. Figure S52: ^13^C NMR spectrum (DMSO-d_6_, 101 MHz, 298 K) of compound **41b**. Figure S53: ^1^H NMR spectrum (DMSO-d_6_, 400 MHz, 298 K) of compound **41c**. Figure S54: ^13^C NMR spectrum (DMSO-d_6_, 101 MHz, 298 K) of compound **41c**. Figure S55: ^1^H NMR spectrum (CDCl_3_, 600 MHz, 298 K) of compound **46a**. Figure S56: ^13^C NMR spectrum (CDCl_3_, 151 MHz, 298 K) of compound **46a**. Figure S57: ^1^H NMR spectrum (DMSO-d_6_, 600 MHz, 298 K) of compound **51a**. Figure S58: ^13^C NMR spectrum (DMSO-d_6_, 151 MHz, 298 K) of compound **51a**. Figure S59: ^1^H NMR spectrum (CDCl_3_, 600 MHz, 298 K) of compound **52a**. Figure S60: ^13^C NMR spectrum (CDCl_3_, 151 MHz, 298 K) of compound **52a**. Figure S61: ^1^H NMR spectrum (methanol-d_4_, 400 MHz, 298 K) of compound **53a**. Figure S62: ^13^C NMR spectrum (methanol-d_4_, 101 MHz, 298 K) of compound **53a**. Figure S63: ^1^H NMR spectrum (DMSO-d_6_, 400 MHz, 298 K) of compound **54a**. Figure S64: ^13^C NMR spectrum (DMSO-d_6_, 101 MHz, 298 K) of compound **54a**. Figure S65: ^1^H NMR spectrum (DMSO-d_6_, 400 MHz, 298 K) of compound **55a**. Figure S66: ^13^C NMR spectrum (DMSO-d_6_, 101 MHz, 298 K) of compound **55a**. Figure S67: ^1^H NMR spectrum (CDCl_3_, 400 MHz, 298 K) of compound **45**. Figure S68: ^13^C NMR spectrum (CDCl_3_, 101 MHz, 298 K) of compound **45**. Figure S69: ^1^H NMR spectrum (CDCl_3_, 400 MHz, 298 K) of compound **46b**. Figure S70: ^13^C NMR spectrum (CDCl_3_, 101 MHz, 298 K) of compound **46b**. Figure S71: ^1^H NMR spectrum (DMSO-d_6_, 400 MHz, 298 K) of compound **51b**. Figure S72: ^13^C NMR spectrum (DMSO-d_6_, 101 MHz, 298 K) of compound **51b**. Figure S73: ^1^H NMR spectrum (CDCl_3_, 400 MHz, 298 K) of compound **52b**. Figure S74: ^13^C NMR spectrum (CDCl_3_, 101 MHz, 298 K) of compound **52b**. Figure S75: ^1^H NMR spectrum (methanol-d_4_, 400 MHz, 298 K) of compound **53b**. Figure S76: ^13^C NMR spectrum (methanol-d_4_, 101 MHz, 298 K) of compound **53b**. Figure S77: ^1^H NMR spectrum (DMSO-d_6_, 400 MHz, 298 K) of compound **54b**. Figure S78: ^13^C NMR spectrum (DMSO-d_6_, 101 MHz, 298 K) of compound **54b**. Figure S79: ^1^H NMR spectrum (DMSO-d_6_, 400 MHz, 298 K) of compound **55a**. Figure S80: ^13^C NMR spectrum (DMSO-d_6_, 101 MHz, 298 K) of compound **55a**. Figure S81: ^1^H NMR spectrum (CDCl_3_, 400 MHz, 298 K) of compound **50**. Figure S82: ^13^C NMR spectrum (CDCl_3_, 101 MHz, 298 K) of compound **50**. Figure S83: ^1^H NMR spectrum (DMSO-d_6_, 400 MHz, 298 K) of compound **51c**. Figure S84: ^13^C NMR spectrum (DMSO-d_6_, 101 MHz, 298 K) of compound **51c**. Figure S85: ^1^H NMR spectrum (CDCl_3_, 400 MHz, 298 K) of compound **52c**. Figure S86: ^13^C NMR spectrum (CDCl_3_, 101 MHz, 298 K) of compound **52c**. Figure S87: ^1^H NMR spectrum (methanol-d_4_, 400 MHz, 298 K) of compound **53c**. Figure S88: ^13^C NMR spectrum (methanol-d_4_, 101 MHz, 298 K) of compound **53c**. Figure S89: ^1^H NMR spectrum (DMSO-d_6_, 600 MHz, 298 K) of compound **54c**. Figure S90: ^13^C NMR spectrum (DMSO-d_6_, 151 MHz, 298 K) of compound **54c**. Figure S91: ^1^H NMR spectrum (DMSO-d_6_, 600 MHz, 298 K) of compound **55c**. Figure S92: ^13^C NMR spectrum (DMSO-d_6_, 151 MHz, 298 K) of compound **55c**. Figure S93: ATR-IR spectrum of compound **6**. Figure S94: ATR-IR spectrum of compound **10**. Figure S95: ATR-IR spectrum of compound **11**. Figure S96: ATR-IR spectrum of compound **17**. Figure S97: ATR-IR spectrum of compound **21**. Figure S98: ATR-IR spectrum of compound **22**. Figure S99: ATR-IR spectrum of compound **28a**. Figure S100: ATR-IR spectrum of compound **29a**. Figure S101: ATR-IR spectrum of compound **30a**. Figure S102: ATR-IR spectrum of compound **33a**. Figure S103: ATR-IR spectrum of compound **26**. Figure S104: ATR-IR spectrum of compound **27**. Figure S105: ATR-IR spectrum of compound **28b**. Figure S106: ATR-IR spectrum of compound **29b**. Figure S107: ATR-IR spectrum of compound **30b**. Figure S108: ATR-IR spectrum of compound **33b**. Figure S109: ATR-IR spectrum of compound **S-5**. Figure S110: ATR-IR spectrum of compound **S-6**. Figure S111: ATR-IR spectrum of compound **S-7**. Figure S112: ATR-IR spectrum of compound **S-8**. Figure S113: ATR-IR spectrum of compound **39**. Figure S114: ATR-IR spectrum of compound **40a**. Figure S115: ATR-IR spectrum of compound **40b**. Figure S116: ATR-IR spectrum of compound **40c**. Figure S117: ATR-IR spectrum of compound **41a**. Figure S118: ATR-IR spectrum of compound **41b**. Figure S119: ATR-IR spectrum of compound **41c**. Figure S120: ATR-IR spectrum of compound **42a**. Figure S121: ATR-IR spectrum of compound **42b**. Figure S122: ATR-IR spectrum of compound **42c**. Figure S123: ATR-IR spectrum of compound **43a**. Figure S124: ATR-IR spectrum of compound **43b**. Figure S125: ATR-IR spectrum of compound **43c**. Figure S126: ATR-IR spectrum of compound **46a**. Figure S127: ATR-IR spectrum of compound **51a**. Figure S128: ATR-IR spectrum of compound **52a**. Figure S129: ATR-IR spectrum of compound **53a**. Figure S130: ATR-IR spectrum of compound **54a**. Figure S131: ATR-IR spectrum of compound **55a**. Figure S132: ATR-IR spectrum of compound **56a**. Figure S133: ATR-IR spectrum of compound **45**. Figure S134: ATR-IR spectrum of compound **46b**. Figure S135: ATR-IR spectrum of compound **51b**. Figure S136: ATR-IR spectrum of compound **52b**. Figure S137: ATR-IR spectrum of compound **53b**. Figure S138: ATR-IR spectrum of compound **54b**. Figure S139: ATR-IR spectrum of compound **55b**. Figure S140: ATR-IR spectrum of compound **56b**. Figure S141: ATR-IR spectrum of compound **50**. Figure S142: ATR-IR spectrum of compound **51c**. Figure S143: ATR-IR spectrum of compound **52c**. Figure S144: ATR-IR spectrum of compound **53c**. Figure S145: ATR-IR spectrum of compound **54c**. Figure S146: ATR-IR spectrum of compound **55c**. Figure S147: ATR-IR spectrum of compound **56c**. Figure S148: HR-MS Spectrum (ESI+) of compound **6**. Figure S149: HR-MS Spectrum (ESI+) of compound **10**. Figure S150: HR-MS Spectrum (ESI+) of compound **11**. Figure S151: HR-MS Spectrum (ES) of compound **17**. Figure S152: HR-MS Spectrum (ES) of compound **21**. Figure S153: HR-MS Spectrum (ES) of compound **22**. Figure S154: HR-MS Spectrum (ES) of compound **28a**. Figure S155: HR-MS Spectrum (EI) of compound **29a**. Figure S156: HR-MS Spectrum (ESI+) of compound **30a**. Figure S157: HR-MS Spectrum (ESI+) of compound **33a**. Figure S158: HR-MS Spectrum (ESI+) of compound **26**. Figure S159: HR-MS Spectrum (EI) of compound **27**. Figure S160: HR-MS Spectrum (EI) of compound **28b**. Figure S161: HR-MS Spectrum (EI) of compound **29b**. Figure S162: HR-MS Spectrum (ESI-) of compound **30b**. Figure S163: HR-MS Spectrum (ESI-) of compound **33b**. Figure S164: HR-MS Spectrum (ESI+) of compound **S-5**. Figure S165: HR-MS Spectrum (EI) of compound **35**. Figure S166: HR-MS Spectrum (ESI+) of compound **S-6**. Figure S167: HR-MS Spectrum (ESI+) of compound **S-7**. Figure S168: HR-MS Spectrum (ESI+) of compound **39**. Figure S169: HR-MS Spectrum (ESI+) of compound **40a**. Figure S170: HR-MS Spectrum (ESI+) of compound **40b**. Figure S171: HR-MS Spectrum (ESI+) of compound **40c**. Figure S172: HR-MS Spectrum (ESI+) of compound **41a**. Figure S173: HR-MS Spectrum (ESI+) of compound **41b**. Figure S174: HR-MS Spectrum (ESI+) of compound **41c**. Figure S175: HR-MS Spectrum (ESI+) of compound **42a**. Figure S176: HR-MS Spectrum (ESI+) of compound **42b**. Figure S177: HR-MS Spectrum (ESI+) of compound **42c**. Figure S178: HR-MS Spectrum (ESI+) of compound **43a**. Figure S179: HR-MS Spectrum (ESI+) of compound **43b**. Figure S180: HR-MS Spectrum (ESI+) of compound **43c**. Figure S181: HR-MS Spectrum (ESI+) of compound **46a**. Figure S182: HR-MS Spectrum (EI) of compound **51a**. Figure S183: HR-MS Spectrum (ESI+) of compound **52a**. Figure S184: HR-MS Spectrum (ESI+) of compound **53a**. Figure S185: HR-MS Spectrum (ESI+) of compound **54a**. Figure S186: HR-MS Spectrum (ESI+) of compound **55a**. Figure S187: HR-MS Spectrum (ESI+) of compound **56a**. Figure S188: HR-MS Spectrum (ESI+) of compound **45**. Figure S189: HR-MS Spectrum (ESI+) of compound **46b**. Figure S190: HR-MS Spectrum (ESI+) of compound **51b**. Figure S191: HR-MS Spectrum (ESI+) of compound **52b**. Figure S192: HR-MS Spectrum (ESI+) of compound **53b**. Figure S193: HR-MS Spectrum (ESI+) of compound **54b**. Figure S194: HR-MS Spectrum (ESI+) of compound **55b**. Figure S195: HR-MS Spectrum (ESI+) of compound **56b**. Figure S196: HR-MS Spectrum (ESI+) of compound **50**. Figure S197: HR-MS Spectrum (ESI+) of compound **51c**. Figure S198: HR-MS Spectrum (ESI+) of compound **52c**. Figure S199: HR-MS Spectrum (ESI+) of compound **53c**. Figure S200: HR-MS Spectrum (ESI+) of compound **54c**. Figure S201: HR-MS Spectrum (ESI+) of compound **55c**. Figure S202: HR-MS Spectrum (ESI+) of compound **56c**. Figure S203: Analytical RP-HPLC chromatogram (System A) of compound **30a**. Figure S204: Analytical RP-HPLC chromatogram (System A) of compound **30b**. Figure S205: Analytical RP-HPLC chromatogram (System A) of compound **33a**. Figure S206: Analytical RP-HPLC chromatogram (System A) of compound **33b**. Figure S207: Analytical RP-HPLC chromatogram (System A) of compound **40a**. Figure S208: Analytical RP-HPLC chromatogram (System A) of compound **40b**. Figure S209: Analytical RP-HPLC chromatogram (System A) of compound **40c**. Figure S210: Analytical RP-HPLC chromatogram (System A) of compound **41a**. Figure S211: Analytical RP-HPLC chromatogram (System A) of compound **41b**. Figure S212: Analytical RP-HPLC chromatogram (System A) of compound **41c**. Figure S213: Analytical RP-HPLC chromatogram (System A) of compound **42a**. Figure S214: Analytical RP-HPLC chromatogram (System A) of compound **42b**. Figure S215: Analytical RP-HPLC chromatogram (System A) of compound **42c**. Figure S216: Analytical RP-HPLC chromatogram (System A) of compound **43a**. Figure S217: Analytical RP-HPLC chromatogram (System A) of compound **43b**. Figure S218: Analytical RP-HPLC chromatogram (System A) of compound **43c**. Figure S219: Analytical RP-HPLC chromatogram (System A) of compound **53a**. Figure S220: Analytical RP-HPLC chromatogram (System A) of compound **54a**. Figure S221: Analytical RP-HPLC chromatogram (System A) of compound **55a**. Figure S222: Analytical RP-HPLC chromatogram (System A) of compound **56a**. Figure S223: Analytical RP-HPLC chromatogram (System A) of compound **53b**. Figure S224: Analytical RP-HPLC chromatogram (System A) of compound **54b**. Figure S225: Analytical RP-HPLC chromatogram (System A) of compound **55b**. Figure S226: Analytical RP-HPLC chromatogram (System A) of compound **56b**. Figure S227: Analytical RP-HPLC chromatogram (System A) of compound **53c**. Figure S228: Analytical RP-HPLC chromatogram (System A) of compound **54c**. Figure S229: Analytical RP-HPLC chromatogram (System A) of compound **55c**. Figure S230: Analytical RP-HPLC chromatogram (System A) of compound **56c**. Figure S231: Radio-HPLC chromatogram of [^64^Cu]Cu-**56a**. Figure S232: Radio-HPLC chromatogram of [^64^Cu]Cu-**56b**. Figure S233: Radio-HPLC chromatogram of [^64^Cu]Cu-**56c**. Figure S234: Radio-HPLC chromatograms (System E) of ^177^Lu-labeled compounds (a) [^177^Lu]Lu-**42c**; (b) [^177^Lu]Lu-**43c**; (c) [^177^Lu]Lu-**56b**; (d) [^177^Lu]Lu-**56c** incubated in 1 M HEPES solution for 1 h (red), 24 h (yellow), 48 h (green), 72 h (blue) and 7 d (purple). Figure S235: Radio-HPLC chromatograms (System E) of ^177^Lu-labeled compounds (a) [^177^Lu]Lu-**42c**; (b) [^177^Lu]Lu-**43c**; (c) [^177^Lu]Lu-**56b**; (d) [^177^Lu]Lu-**56c** incubated in PBS solution for 1 h (red), 24 h (yellow), 48 h (green), 72 h (blue) and 7 d (purple). Figure S236: Radio-HPLC chromatograms (System E) of ^177^Lu-labeled compounds (a) [^177^Lu]Lu-**42c**; (b) [^177^Lu]Lu-**43c**; (c) [^177^Lu]Lu-**56a** (d) [^177^Lu]Lu-**56b**; (e) [^177^Lu]Lu-**56c** after incubation in human serum for 1 h (red), 24 h (yellow), 48 h (green), 72 h (blue) and 7 d (purple) and subsequent methanol-chloroform precipitation. Figure S237: Radio-HPLC chromatograms (size exclusion chromatography, System F) of (a) human serum (detection of UV-lane) and ^177^Lu-labeled compounds (b) [^177^Lu]Lu-**42c**; (c) [^177^Lu]Lu-**43c**; (d) [^177^Lu]Lu-**56a**; (e) [^177^Lu]Lu-**56b**; (f) [^177^Lu]Lu-**56c** from reaction mixture (detection of γ-line). Figure S238: Radio-HPLC chromatograms (size exclusion chromatography, System F) of ^177^Lu-labeled compounds (a) [^177^Lu]Lu-**42c**; (b) [^177^Lu]Lu-**43c**; (c) [^177^Lu]Lu-**56a** (d) [^177^Lu]Lu-**56b**; (e) [^177^Lu]Lu-**56c** after incubation in human serum for 1 h (red), 24 h (yellow), 48 h (green), 72 h (blue) and 7 d (purple). Shown are both duplicates of each analysis. Table S1: Integrated peak areas in percentage of tracers (a) [^177^Lu]Lu-**42c**; (b) [^177^Lu]Lu-**43c**; (c) [^177^Lu]Lu-**56a** (d) [^177^Lu]Lu-**56b**; (e) [^177^Lu]Lu-**56c** bound to human serum proteins of radio-HPLC chromatograms presented in Figure S238. Figure S239: Binding of ^177^Lu-labeled PD-L1 ligands to human serum proteins. Colloidal Coomassie stained native polyacrylamide gels (A) and corresponding autoradiographs (B) showing electrophoretic separation of ^177^Lu-labeled ligands **42c**, **43c**, **56a**, **56b** and **56c** with and without incubation in human serum for 24 h. Incubation of uncomplexed [^177^Lu]Lu^3+^ with human serum was used as control reaction. Figure S240: Non-linear iterative curve fitting of saturation binding experimental data. (A) First series compounds [^64^Cu]Cu-**42a–c** and [^64^Cu]Cu-**43a–c**; (B) Second series compounds [^64^Cu]Cu-**56a–c** and (C) cyclic peptide [^64^Cu]Cu-DOTAGA-WL12. Curves show representative fits for total and nonspecific binding of 3 individual experiments combined, along with 95% confidence levels (dotted lines). Scaling is only comparable within A/B/C. Figure S241: Real-time radioligand binding (trace) of one first, two second series compounds and the cyclic peptide WL12 in the absence(A/C/E/G) and presence (B/D/F/H) of 2.5% bovine serum albumin (BSA). Kinetic parameters (association rate constant k_a_, dissociation rate constant k_d_ and dissociation constant K_D_) are reported in Figure S3E. Figure S242: In vivo distribution (maximum intensity projections) of ^64^Cu-labeled first generation compounds **42a**, **43a** and **42b** at 0–2, 4–5 and 24–25 h post injection (p.i.). SUV scales differ across images. Figure S243: In vivo distribution (maximum intensity projections) of ^64^Cu-labeled first generation compounds **43b**, **42c**, **43c** at 0–2, 4–5 and 24–25 h post injection (p.i.). SUV scales differ across images. Figure S244: In vivo distribution (maximum intensity projections) of ^64^Cu-labeled second generation compounds **56a–c** at 0–2, 4–5 and 24–25 h post injection (p.i.). SUV scales differ across images. Table S2: SUV_max_ values (mean ± S.D., if applicable) of first and second-series compounds derived from PET data in PD-L1 overexpressing and mock tumors at different timepoints post injection (p.i.). Table S3: SUV_max_ values (mean ± S.D., if applicable) of first and second-series compounds with cold compound as blocking substance derived from PET data in PD-L1 overexpressing and mock tumors at different timepoints post injection (p.i.). Figure S245: Immunostaining of random PD-L1 positive and mock tumors confirms target overexpression (PD-L1, A) and absence thereof (mock, B).
