# Supplementary material for: Design and Biological Evaluation of Small-Molecule PET-Tracers for Imaging of Programmed Death Ligand 1
Source: Cancers (Basel). 2023 May 6;15(9):2638. doi: 10.3390/cancers15092638 (PMC10177516; doi:10.3390/cancers15092638)
Supplement: Supplementary file 1 [file cancers-15-02638-s001.zip › cancers-2347868-supplementary.pdf]

Supporting Information

Content

1. Organic synthesis....................S1
2. ^{1}H and ^{13}C NMR spectra of literature unknown compounds S42
3. IR spectra of literature unknown compounds.................... S89
4. HR-MS spectra of literature unknown compounds.................... S109
5. HPLC chromatograms of HPLC purified compounds.................... S131
6. Radio-HPLC chromatograms of labeled radiotracers.................... S142
7. Stability of radiolabeled compounds S144
   8.1 Kinetic stability in buffer solutions.................... S144
   8.2 Proteolytic stability in human serum S145
   8.3 Serum protein binding studies S146
8. In vitro: Saturation binding assays S150
9. In vitro: Real-time radioligand binding.................... S151
10. In vivo: Qualitative PET scans.................... S152
11. References.................... S156

1. Organic synthesis

Reaction schemes of literature known compounds

Compound **1** (BMS-1166) was used as a blocking substance for the saturation binding assays and was synthesized with our newly developed synthetic route. The ^{1}H NMR and ^{13}C NMR spectra were in accordance to the literature.[1]

Scheme S1: Synthesis of PD-L1 inhibitor 1 (BMS-1166) with new synthetic strategy.

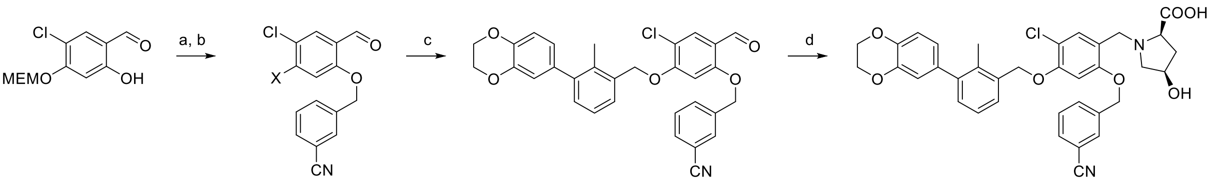

a) 3-cyanobenzyl bromide, K_2CO_3, abs. DMF, rt, 16 h and 95%. (b) TFA:DCM (1:1), 0 °C to rt, 16 h, and 78%. (c) 3-(2,3-dihydro-1,4-benzodioxin-6-yl)-2-methylbenzenemethanol, DEAD, PPh_3, abs. DMF, rt, 16 h and 92%. (d) *cis*-4-hydroxy-D-prolin, $NaBH_3CN$, abs. DMF/MeOH (1:1), 0 °C to rt, 16 h, and 41%.

General Procedures

GP-1: S_N2-Alkylation

The corresponding alcohol (1.0 equiv.) was dissolved in anhydrous DMF under argon atmosphere and potassium carbonate (2.0 equiv.) was added. After stirring the suspension at room temperature for 10 min, the alkyl bromide (1.2-1.5 equiv.) was then added. The reaction mixture was stirred at room temperature for the corresponding time and after completion, the solvent was removed in vacuo. The residue was taken up in an organic solvent and water, the phases were separated, and the aqueous phase extracted with an organic solvent three times. The combined organic phases were dried over anhydrous sodium sulfate and after filtration, the solvent was removed in vacuo. The crude product was purified by flash column chromatography on silica gel using an appropriate eluent.

GP-2: Mitsunobu-Reaction

The phenolic compound (1.0 equiv), the alcohol (1.1 equiv.) and triphenylphosphine (1.3 equiv) were dissolved in anhydrous DMF under argon atmosphere. The reaction solution was cooled to 0 °C and DEAD (1.3 equiv.) was added slowly by syringe. After allowing to warm to room temperature, the reaction solution was stirred for the corresponding time. After completion, all volatiles were removed in vacuo and the residue was purified by flash column chromatography on silica gel using an appropriate eluent.

GP-3: Reductive Amination

The aldehyde (1.0 equiv.) and the amine (3.0-5.0 equiv.) were dissolved in MeOH/DMF (1:1) under argon atmosphere. After stirring for 20 min at room temperature, the reaction solution was cooled to 0 °C and $NaBH_3CN$ (1.5 equiv.) was added. The solution was warmed to room temperature and stirred for the corresponding time. After completion of the reaction,

water and ethyl acetate were added. Phases were separated and the aqueous phase was extracted with ethyl acetate three times. The organic layers were combined, dried over anhydrous sodium sulfate and filtered. After removing the solvent in vacuo, the crude product was purified by flash column chromatography on silica gel using an appropriate eluent.

GP-4: CuAAC-Reaction (Cu(I)-Catalysator system)

The alkyne (1.00 equiv.) and azide (1.50-5.00 equiv.) were dissolved in the corresponding solvent and the resulting solution was degassed with argon for 30 min. TBTA (0.01 equiv.) and $[Cu(MeCN)_4]PF6$ (0.50 equiv.) were added in one portion under argon. The reaction mixture was stirred at room temperature for 16 h. Complete conversion of starting material was verified either by TLC or RP-HPLC. The solvent was removed in vacuo and the crude product was purified either by flash column chromatography on silica gel or by RP-HPLC with subsequent lyophilization.

GP-5: CuAAC-Reaction ($CuSO_4$/sodium ascorbate system)

The alkyne (1.00 equiv.) and azide component (1.00-5.00 equiv.) were dissolved in a 1:1 mixture of H_2O/t-BuOH. $CuSO_4$ (0.30 equiv.), THPTA (0.10 equiv.) and sodium ascorbate (5.00 equiv.) were premixed in H_2O/t-BuOH (1:1) and then added to the reaction solution, which was stirred at room temperature until complete conversion was achieved (monitored by RP-HPLC System A). All volatiles were removed in vacuo, the crude product was purified by RP-HPLC and after lyophilization, the desired triazole was obtained.

GP-6: Amide-Coupling of 2-Aminoethan-1,1-Disulfonic Acid (TBA Salt)

The carboxylic acid (1.0 equiv.), 2-aminoethan-1,1-disulfonic acid (TBA salt) (5.0 equiv.), anhydrous DIPEA (2.0 equiv.) and HBTU (2.2 equiv.) were stirred in anhydrous DMF at room temperature for 40 h. The suspension was filtered over a short plug of celite and DMF was removed under reduced pressure. The crude product was purified by RP-HPLC to yield the product as a TBA salt after lyophilization. Dissolving in water and stirring with DOWEX® 50WX8 at room temperature for 2 h, followed by lyophilization led to the disulfonated product in acid form.

GP-7: DOTA-Conjugation

The secondary amine (1.0 equiv.) was dissolved in anhydrous DMF and the reaction was initialized by the addition of anhydrous DIPEA (5.0 equiv.) and DOTA-*p*-nitrophenylester (2.0 equiv.). The reaction was stirred at room temperature for 40 h and complete consumption

was verified by analytical RP-HPLC (System A). The reaction mixture was then stirred at 80 °C for 4 h to decompose excess of DOTA-*p*-nitrophenylester. The solvents were removed under reduced pressure and the crude product was purified by RP-HPLC and lyophilized to yield the DOTA-conjugated compound.

HPLC-Systems

System A: RP-HPLC, analytical (Agilent Zorbax 300 C-18, 5 µm, 4.6 x 150 mm) with 10-95% acetonitrile (0.1% TFA) in water (0.1% TFA) in a linear gradient over 15 min, 1 mL/min.

System B: RP-HPLC, semi-preparative (Zorbax SB C-18 5 µm 80 Å, 9.4 x 250 mm) with 35-90% acetonitrile (0.1% TFA) in water (0.1% TFA) in a linear gradient over 45 min, 6 mL/min.

System C: RP-HPLC, semi-preparative (Zorbax SB C-18 5 µm 80 Å, 9.4 x 250 mm) with 20-60% acetonitrile (0.1% TFA) in water (0.1% TFA) in a linear gradient over 45 min, 6 mL/min.

System D: hydro-RP-HPLC, preparative (Phenomenex Synergi hydro-RP 4 µm 80 Å, 21.2 x 250 mm) with 25-35% acetonitrile (0.1% TFA) in water (0.1% TFA) in a linear gradient over 60 min, 15 mL/min.

System E: RP-HPLC, analytical (Kintetex® 5 µm Phenyl-Hexyl100 Å) with 5–95% acetonitrile (0.1% TFA) in water (0.1% TFA) in a linear gradient over 10 min, 1 mL/min.

System F: Size-Exclusion-Chromatography (SEC), analytical (Agilent 8 µM PL aquagel-OH 30, 300 x 7.5 mm) with 100% PBS-buffer pH 7 isocratic over 20 min, 1 mL/min

Synthethic Procedures

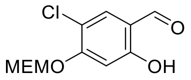

5-Chloro-2-hydroxy-4-((2-methoxyethoxy)methoxy)benzaldehyde (**5**). 5-Chloro-2,4-dihydroxybenzaldehyde (**4**) (4.30 g, 24.7 mmol, 1.00 equiv.) was dissolved in anhydrous THF (80 mL) under argon atmosphere and anhydrous triethylamine (8.81 mL, 49.5 mmol, 2.00 equiv.) was added *via* syringe in one portion. After cooling to 0 °C, MEM chloride (3.96 mL, 34.6 mmol, 1.40 equiv.) was added in small portions. The completion of the reaction was verified by TLC after stirring at room temperature for 16 h under argon. Water (100 mL) was added and the phases were separated. The aqueous phase was extracted with DCM (3 x 100 mL) and the combined organic extracts were dried over anhydrous sodium sulfate, filtered

and concentrated. The residue was purified by flash column chromatography on silica gel (PE:EA, 8:2, R_f = 0.25) to afford **5** (3.87 g, 14.9 mmol, 60%) as colorless crystals. mp = 53 °C. ^{1}H NMR (600 MHz, $CDCl_3$) δ = 11.28 (s, 1H), 9.70 (s, 1H), 7.52 (s, 1H), 6.80 (s, 1H), 5.40 (s, 2H), 3.85–3.87 (m, 2H), 3.55–3.57 (m, 2H), 3.37 ppm (s, 3H). ^{13}C NMR (151 MHz, $CDCl_3$) δ = 194.0, 162.7, 159.7, 134.2, 115.9, 115.0, 103.8, 94.1, 71.5, 68.8, 59.2 ppm. IR (ATR): $\tilde{\nu}$ = 2856 (w), 1643 (s), 1622 (s), 1572 (m), 1489 (s), 1454 (m), 1354 (m), 1320 (m), 1273 (m), 1242 (w), 1223 (w), 1195 (s), 1154 (m), 1136 (m), 1105 (s), 1027 (s), 940 (s), 891 (m), 830 (s), 738 (s), 717 (s), 705 (s), 681 (m), 662 (m), 582 (w), 559 (m), 493 (w), 458 (w), 436 cm^{-1} (w). HRMS (ESI, $[M+Na]^+$) calcd for $C_{11}H_{13}ClO_5Na$, 283.0349; found, 283.0346.

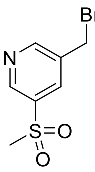

*3-(Bromomethyl)-5-(methylsulfonyl)pyridine (**9**)*. (5-(Methylsulfonyl)pyridin-3-yl)methanol (**8**) (5.00 g, 35.1 mmol, 1.00 equiv.) was dissolved under argon in abs. THF (200 mL) and cooled to 0 °C. Triphenylphosphine (9.67 g, 36.9 mmol, 1.05 equiv.) and tetrabromomethane (12.2 g, 36.90 mmol, 1.05 equiv.) were added and the solution was stirred at 0 °C for 15 min and then for 16 h at room temperature until complete consumption of starting material was observed. Subsequently, the reaction mixture was filtered, water (200 mL) was added to the filtrate, phases were separated and the organic extract was dried over sodium sulfate. After filtration and removal of the solvent, the crude product was purified by flash column chromatography on silica gel (PE:EA, 5:5, R_f = 0.15) to afford 3-(bromomethyl)-5-(methylsulfonyl)pyridine (**9**) (3.73 g, 14.9 mmol, 42%) as beige crystals. mp = 240 °C. ^{1}H NMR (600 MHz, $CDCl_3$) δ = 9.07 (d, ^4J = 2.1 Hz, 1H), 8.90 (d, ^4J = 2.1 Hz, 1H), 8.26 (t, ^4J = 2.1 Hz, 1H), 4.53 (s, 2H), 3.14 ppm (s, 3H). ^{13}C NMR (151 MHz, $CDCl_3$) δ = 154.5, 148.2, 137.2, 135.7, 134.8, 45.0, 27.8 ppm. IR (ATR): $\tilde{\nu}$ = 3024 (w), 1565 (w), 1423 (m), 1295 (s), 1246 (m), 1211 (m), 1160 (m), 1138 (s), 1099 (s), 1021 (w), 960 (m), 915 (w), 860 (w), 766 (m), 707 (m), 683 (m), 638 (w), 573 (m), 544 (m), 525 (s), 441 (w), 423 cm^{-1} (m). HRMS (EI, $[M]^+$) calcd for $C_7H_8BrNO_2S$, 248.9459; found, 248.9467.

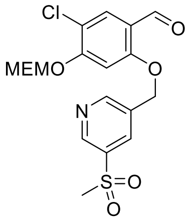

5-Chloro-4-((2-methoxyethoxy)methoxy)-2-((5-(methylsulfonyl)pyridin-3-yl)methoxy)benzaldehyde ***(10)***. 5-Chloro-2-hydroxy-4-((2-methoxyethoxy)methoxy)benzaldehyde (**6**) (2.09 g, 8.01 mmol, 1.00 equiv.) reacted with 3-(bromomethyl)-5-(methylsulfonyl)pyridine (**9**) (2.60 g, 10.4 mmol, 1.30 equiv.) and potassium carbonate (2.21 g, 16.0 mmol, 2.00 equiv.) as a base in abs. DMF (40 mL) according to GP-1. Purification by flash column chromatography on silica gel (EA:MeOH, 100:1.5, R_f = 0.25) provided 5-chloro-4-((2-methoxyethoxy)methoxy)-2-((5-(methylsulfonyl)pyridin-3-yl)methoxy)benzaldehyde (**10**) (2.89 g, 6.72 mmol, 84%) as a yellowish solid. mp = 134 °C. ^{1}H NMR (600 MHz, $CDCl_3$) δ = 10.28 (s, 1H), 9.16 (s, 1H), 8.99 (s, 1H), 8.37 (s, 1H), 7.87 (s, 1H), 7.01 (s, 1H), 5.42 (s, 2H), 5.27 (s, 2H), 3.88-3.90 (m, 2H), 3.57-3.59 (m, 2H), 3.37 (s, 3H), 3.16 ppm (s, 3H). ^{13}C NMR (151 MHz, $CDCl_3$) δ = 186.7, 160.0, 158.9, 153.4, 148.6, 137.4, 134.5, 132.4, 130.7, 120.3, 117.5, 101.0, 94.3, 71.5, 68.5, 67.9, 59.1), 45.0 ppm. IR (ATR): $\tilde{\nu}$ = 2921 (w), 1667 (m), 1594 (m), 1498 (w), 1429 (w), 1398 (w), 1384 (m), 1303 (s), 1258 (m), 1188 (m), 1145 (s), 1117 (s), 1084 (m), 1059 (w), 1013 (m), 984 (m), 967 (s), 894 (w), 867 (m), 851 (m), 824 (m), 768 (s), 722 (m), 701 (m), 670 (m), 642 (w), 590 (w), 557 (m), 535 (s), 516 (m), 458 (w), 427cm^{-1} (w). HRMS (ESI, [2M+Na]$^+$) calcd for $C_{36}H_{40}Cl_2N_2O_{14}S_2$, 881.1190; found, 881.1190.

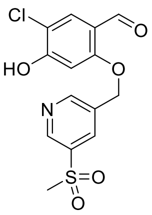

5-Chloro-4-hydroxy-2-((5-(methylsulfonyl)pyridin-3-yl)methoxy)benzaldehyde ***(11)***. MEM-Protected phenol **10** (2.87 g, 6.68 mmol, 1.00 equiv.) was dissolved in abs. dichloromethane (50 mL) under argon and the solution was cooled to 0 °C. After addition of trifluoracetic acid (10 mL), the reaction mixture was stirred at room temperature for 16 h. Ethyl acetate (100 mL) was added and a solid precipitated, which was isolated by filtration. The pale greenish

precipitate turned out to be 5-chloro-4-hydroxy-2-((5-(methylsulfonyl)pyridin-3-yl)methoxy)benzaldehyde (**11**) (0.96 g, 2.81 mmol, 42%), which was used in the next step without further purification. mp = 236 °C (decomposition). ^{1}H NMR (600 MHz, DMSO-d$_6$) δ = 11.63 (s, 1H), 10.10 (s, 1H), 9.09 (d, ^{4}J = 2.0 Hz), 9.07 (d, ^{4}J = 2.0 Hz), 8.48 (d, ^{4}J = 2.0 Hz), 7.67 (s, 1H), 6.81 (s, 1H), 5.38 (s, 2H), 3.37 ppm (s, 3H). ^{13}C NMR (151 MHz, DMSO-d$_6$) δ = 186.5, 160.4, 159.9, 153.3, 147.4, 137.0, 134.3, 132.8, 129.5, 117.9, 113.5, 101.5, 67.1, 43.6 ppm. IR (ATR): $\tilde{\nu}$ = 2915 (w), 1672 (m), 1598 (m), 1520 (w), 1452 (m), 1399 (m), 1368 (w), 1303 (s), 1214 (m), 1191 (s), 1142 (s), 1103 (m), 1029 (m), 974 (m), 902 (w), 845 (w), 816 (m), 765 (m), 725 (m), 699 (m), 665 (w), 641 (w), 556 (m), 535 (s), 458 (m), 429cm^{-1} (w). HRMS (ESI, [M+H]$^{+}$) calcd for $C_{14}H_{13}ClNO_5S$, 342.0197; found, 342.0197.

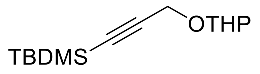

*(3-Bromoprop-1-yn-1-yl)(tert-butyl)dimethylsilane (**14**)*. TBDMS-protected alkyne **13** (13.5 g, 53.1 mmol, 1.00 equiv.) was dissolved in DCM (300 mL), triphenylphosphine bromide (24.6 g, 58.4 mmol, 1.10 equiv.) was added and the reaction mixture was stirred at room temperature for 3 h. The solvent was removed under reduced pressure and the crude product was filtered over a plug of silica gel (PE) to elute (3-bromoprop-1-yn-1-yl)(*tert*-butyl)dimethylsilane (**14**) (8.57 g, 36.8 mmol, 69%) as a yellowish oil. Analytical data are in accordance with literature [2].

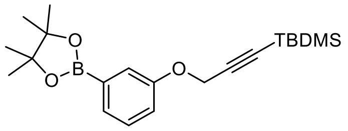

*tert-butyldimethyl(3-(3-(4,4,5,5-tetramethyl-1,3,2-dioxaborolan-2-yl)phenoxy)prop-1-yn-1-yl)silane (**17**)*. 3-(4,4,5,5-Tetramethyl-1,3,2-dioxaborolan-2-yl)phenol (**16**) (4.03 g, 18.3 mmol, 1.00 equiv.) reacted with 3-(TBDMS)propargyl bromide (5.13 g, 22.0 mmol, 1.20 equiv.) and potassium carbonate (3.80 g, 27.5 mmol, 1.50 equiv.) as a base in abs. DMF (50 mL) according to GP-1. Purification by flash column chromatography on silica gel (PE:DCM, 1:1, R_f = 0.4) gave TBDMS-protected alkyne **17** (4.63 g, 12.4 mmol, 68%) as yellow oil. ^{1}H NMR (600 MHz, CDCl$_3$) δ = 7.44-7.42 (m, 2H), 7.29-7.27 (m, 1H), 7.08 (ddd, ^{3}J = 8.2 Hz, ^{4}J = 2.7 Hz, ^{5}J = 1.2 Hz, 1H), 4.71 (s, 2H), 1.34 (s, 12H), 0.91 (s, 9H), 0.11 ppm (s, 6H). ^{13}C NMR (151 MHz, CDCl$_3$) δ = 157.6, 129.0, 128.2, 120.9, 119.2, 101.4, 91.2, 84.0, 57.1, 26.2, 25.1, 16.6, -4.6 ppm. IR (ATR): $\tilde{\nu}$ = 2929 (m), 2857 (m), 1575 (w), 1489 (w), 1428 (m), 1349 (s), 1315 (m), 1251 (w), 1210 (s), 1143 (s), 1069

(w), 1042 (m), 964 (m), 884 (w), 825 (m), 776 (m), 705 (m), 580 cm-1 (w). HRMS (EI, $[M]^+$) calcd for $C_{21}H_{33}BO_3Si$, 372.2292; found, 372.2275.

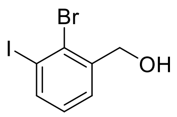

*3-Iodo-2-bromobenzylalcohol (**21**)*. 3-Iodo-2-bromobenzylbromide (**20**) (7.80 g, 20.8 mmol, 1.00 equiv.) was suspended in an 8:2 mixture of DMF/H_2O (250 mL) and the suspension was heated to 80 °C for 16 h. After complete conversion, the mixture was cooled down and DCM (400 mL) and water (200 mL) were added. The phases were separated, the aqueous phase was extracted with DCM (3 x 400 mL), the combined organic phases were dried over sodium sulfate, filtered and concentrated. The residue was purified by flash column chromatography on silica gel (PE:EA, 10:1, R_f = 0.2) to yield 3-iodo-2-bromobenzylalcohol (**21**) (4.89 g, 15.6 mmol, 75%) as colorless crystals. mp = 97 °C. ^{1}H NMR (400 MHz, DMSO-d$_6$) δ = 7.85 (d, ^3J = 7.7 Hz, 1H), 7.50 (d, ^3J = 7.7 Hz, 1H), 7.18 (t, ^3J = 7.7 Hz, 1H), 4.50 ppm (s, 2H). ^{13}C NMR (101 MHz, DMSO-d$_6$) δ = 181.4, 175.9, 166.7, 165.0, 164.9, 141.0, 102.2 ppm. IR (ATR): $\tilde{\nu}$ = 3201 (w), 1530 (w), 1431 (w), 1400 (m), 1431 (m), 1233 (w), 1180 (w), 1133 (m), 1088 (m), 153 (s), 1012 (m), 981 (m), 814 (w), 7662 (s), 685 (s), 616 cm^{-1} (m). HRMS (EI, $[M]^+$) calcd for C_7H_6BrIO 311.8638; found, 311.8647. The analytical data are in accordance with the literature [3].

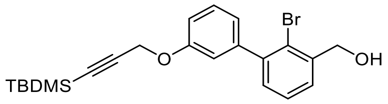

*(2-Bromo-3'-((3-(tert-butyldimethylsilyl)prop-2-yn-1-yl)oxy)-[1,1'-biphenyl]-3-yl)methanol (**22**)*. 3-Iodo-2-bromobenzylalcohol (**21**) (3.22 g, 10.3 mmol, 1.00 equiv.) and TBDMS-protected alkyne **17** (4.41 g, 11.8 mmol, 1.15 equiv.) were dissolved in a 20:0.5:0.1 mixture of toluene/EtOH/H_2O (130 mL) and the resulting solution was degassed with argon for 30 min. The reaction was initiated by the addition of potassium carbonate (2.84 g, 20.6 mmol, 2.00 equiv.) and tetrakis(triphenylphosphine)palladium(0) (0.59 g, 0.51 mmol, 0.05 equiv.). The mixture was stirred at 78 °C for 16 h until all starting material was consumed. It was cooled to room temperature, water (200 mL) and DCM (400 mL) were added, the phases were separated and the aqueous phase was extracted with DCM (3 x 300 mL). Drying over sodium sulfate, filtrating and concentrating under reduced pressure lead to the crude product, which

was purified by flash column chromatography on silica gel (PE:EA, 8:2, R_f = 0.2) to give (2-bromo-3'-((3-(TBDMS)prop-2-yn-1-yl)oxy)-[1,1'-biphenyl]-3-yl)methanol (**22**) (2.79 g, 6,47 mmol, 63%) as a yellowish oil. ^{1}H NMR (400 MHz, DMSO-d$_6$) δ = 7.56 (d, ^3J = 7.6 Hz, 1H), 7.45 (t, ^3J = 7.6 Hz, 1H), 7.36 (t, ^3J = 7.9 Hz, 1H), 7.23-7.21 (m, 1H), 7.02-7.00 (m, 1H), 6.97-6.94 (m, 2H), 5.50 (t, ^3J = 5.1 Hz, 1H), 4.86 (s, 2H), 4.57 (s, 2H), 0.83 (s, 9H), 0.06 ppm (s, 6H). ^{13}C NMR (101 MHz, DMSO-d$_6$) δ = 156.8, 142.2, 141.9, 129.3, 129.0, 127.2, 126.8, 122.3, 115.8, 114.5, 102.0, 90.1, 63.3, 56.2, 25.7, 16.1, –4.9 ppm. IR (ATR): $\tilde{\nu}$ = 3344 (w), 3059 (w), 2952 (m), 2927 (m), 2884 (w), 2856 (m), 2177 (w), 1602 (w), 1580 (m), 1461 (m), 1409 (w), 1362 (w), 1250 (m), 1201 (m), 1076 (w), 1340 (m), 1023 (m), 989 (w), 838 (m), 824 (m), 775 (s), 697 (m), 684 cm^{-1} (m). HRMS (EI, [M]$^+$) calcd for $C_{22}H_{27}BrO_2Si$, 430.0964; found, 430.0975.

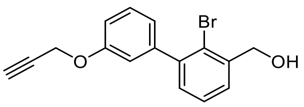

*(2-Bromo-3'-(prop-2-yn-1-yloxy)-[1,1'-biphenyl]-3-yl)methanol (**28a**)*. TBMDS-Protected biaryl **22** (2.56 g, 5.94 mmol, 1.00 equiv.) was dissolved in abs. THF (20 mL), cooled to 0 °C and TBAF (1 M in THF, 6.53 mL, 6.54 mmol, 1.10 equiv.) was added slowly by syringe. The reaction solution was allowed to warm to room temperature and was stirred for 2 h to ensure complete consumption of the starting material. Water (50 mL) and DCM (40 mL) were added, the phases were separated and the aqueous phase was extracted with DCM (3 x 30 mL). The combined organic phases were dried over sodium sulfate, filtered and concentrated under reduced pressure. The crude product was purified by flash column chromatography on silica gel (PE:EA, 7:3, R_f = 0.3) to obtain (2-bromo-3'-(prop-2-yn-1-yloxy)-[1,1'-biphenyl]-3-yl)methanol (**28a**) (1.60 g, 5.04 mmol, 85%) as a colorless solid. mp = 90 °C. ^{1}H NMR (400 MHz, CDCl$_3$) δ = 7.47 (d, ^3J = 7.5 Hz, 1H), 7.35-7-32 (m, 2H), 7.25-7.23 (m, 1H), 7.01-6.97 (m, 3H), 4.80 (s, 2H), 4.71-4.70 (m, 2H), 2.52 ppm (s, 1H). ^{13}C NMR (101 MHz, CDCl$_3$) δ = 157.2, 143.2, 142.8, 140.7, 130.4, 129.2, 127.9, 127.4, 122.9, 122.9, 116.2, 114.3, 78.7, 75.8, 66.0, 56.0 ppm. IR (ATR): $\tilde{\nu}$ = 3279 (s), 3072 (w), 3029 (w), 2929 (w), 2855 (w), 2358 (w), 2116 (w), 1591 (m), 1463 (m), 1452 (m), 1445 (w), 1345 (w), 1318 (w), 1264 (w), 1202 (m), 1176 (w), 1161 (w), 1083 (m), 1060 (m), 1033 (m), 1021 (m), 995 (w), 854 (w), 803 (w), 794 (w), 777 (m), 716 (w), 695 (m), 639 cm^{-1} (m). HRMS (EI, [M]$^+$) calcd for $C_{16}H_{13}BrO_2$, 316.0099; found, 316.0082.

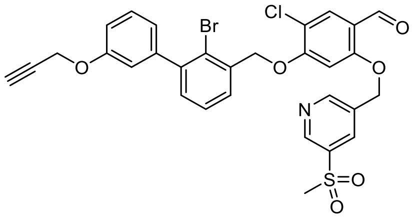

4-((2-Bromo-3'-(prop-2-yn-1-yloxy)-[1,1'-biphenyl]-3-yl)methoxy)-5-chloro-2-((5-methylsulfonyl) pyridin-3-yl)methoxy)benzaldehyde (**29a**). Biaryl **28a** (500 mg, 1.58 mmol, 1.00 equiv.), phenol **11** (647 mg, 1.89 mmol, 1.20 equiv.) and triphenylphosphine (538 mg, 2.05 mmol, 1.30 equiv.) were dissolved in abs. DMF (5 mL) and reacted with DEAD (322 µL, 2.05 mmol, 1.30 equiv.) according to GP-2. Purification by flash column chromatography on silica gel (DCM:EA, 9:1, R_f = 0.25) yielded compound **29a** (808 mg, 1.26 mmol, 80%) as a colorless solid. mp = 145 °C. ^{1}H NMR (400 MHz, DMSO-d$_6$) δ = 10.23 (s, 1H), 9.09 (d, 2H), 8.52 (s, 1H), 7.76 (s, 1H), 7.70 (d, ^{3}J = 7.5 Hz, 1H), 7.54 (t, ^{3}J = 7.6 Hz, 1H), 7.43-7.39 (m, 2H), 7.29 (s, 1H), 7.06-7.00 (m, 3H), 5.57 (s, 2H), 5.46 (s, 2H), 4.85 (d, ^{4}J = 2.0 Hz, 2H), 3.58 (t, ^{4}J = 2.0 Hz, 1H), 3.37 ppm (s, 3H). ^{13}C NMR (101 MHz, DMSO-d$_6$) δ = 186.8, 160.7, 159.3, 156.8, 153.3, 147.4, 142.8, 142.0, 137.0, 135.6, 134.3, 132.7, 131.4, 129.4, 129.2, 128.9, 127.8, 123.1, 122.5, 122.3, 118.9, 115.8, 114.9, 114.2, 100.5, 79.2, 78.4, 71.5, 67.8, 55.5, 43.6 ppm. IR (ATR): ṽ = 3239 (m), 3040 (w), 2991 (m), 2917 (w), 1747 (m), 1693 (s), 1676 (m), 1595 (m), 1530 (s), 1481 (w), 1449 (w), 1367 (w), 1319 (w), 1284 (m), 1233 (s), 1206 (w), 1174 (m), 1144 (m), 1063 (m), 1021 (m), 960 (w), 900 (w), 791 (w), 756 (w), 643 (w), 593 cm^{-1} (w). HRMS (ESI, [M+Na]$^+$) calcd for $C_{30}H_{23}BrClNO_6SNa$, 662.0010; found, 662.0018.

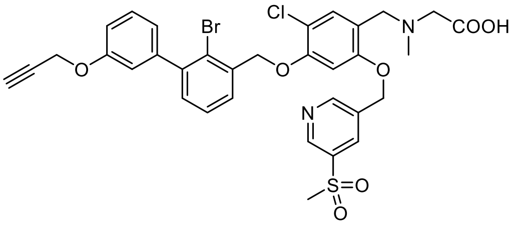

N-(4-((2-Bromo-3'-(prop-2-yn-1-yloxy)-[1,1'-biphenyl]-3-yl)methoxy)-5-chloro-2-((5-(methylsulfonyl) pyridin-3-yl)methoxy)benzyl)-N-methylglycine (**30a**). Aldehyde **29a** (70.0 mg, 109 µmol, 1.00 equiv.), sarcosine (34.1 mg, 382 µmol, 3.50 equiv.) and sodium cyanoborohydride (10.3 mg, 164 µmol, 1.50 equiv.) were reacted in a 1:1 mixture of abs. MeOH/DMF (2 mL) and subsequently worked up according to GP-3. After flash column chromatography on silica gel (MeCN:MeOH:Et_3N, 100:20:1, R_f = 0.2) compound **30a** (44.0 mg,

61.6 µmol, 56%) was obtained as a colorless solid. mp = 175°C. R_t = 11.98 min (System A), purity: 95.4%. ^{1}H NMR (400 MHz, DMSO-d_6) δ = 9.07 (s, 1H), 9.03 (s, 1H), 8.46 (s, 1H), 7.68 (d, ^{3}J = 6.8 Hz, 1H), 7.52 (t, ^{3}J = 7.6 Hz, 1H), 7.45-7.38 (m, 3H), 7.13 (s, 1H), 7.05-6.99 (m, 3H), 5.41 (s, 2H), 5.32 (s, 2H), 4.84 (d, ^{4}J = 1.8 Hz, 2H), 3.75 (s, 3H), 3.58 (s, 1H), 3.21 (s, 2H), 2.34 ppm (s, 3H). ^{13}C NMR (101 MHz, DMSO-d_6) δ = 170.6, 156.8, 155.9, 153.5, 153.0, 147.2, 142.7, 142.0, 136.9, 136.4, 134.2, 133.3, 131.3, 131.1, 129.2, 129.0, 127.7, 122.8, 122.3, 119.4, 115.8, 114.1, 113.3, 100.8, 79.2, 78.4, 71.0, 67.3, 57.5, 55.5, 53.1, 43.7, 41.4 ppm. IR (ATR): ṽ = 3226 (w), 3047 (w), 2925 (w), 1606 (m), 1578 (w), 1506 (m), 1463 (w), 1404 (w), 1366 (w), 1302 (s), 1202 (m), 1149 (m), 1109 (w), 1042 (w), 996 (w), 977 (w), 803 (w), 774 (m), 699 cm^{-1} (w). HRMS (ESI, [M+H]$^{+}$) calcd for $C_{33}H_{31}BrClN_2O_7S$, 713.0719; found, 713.0724.

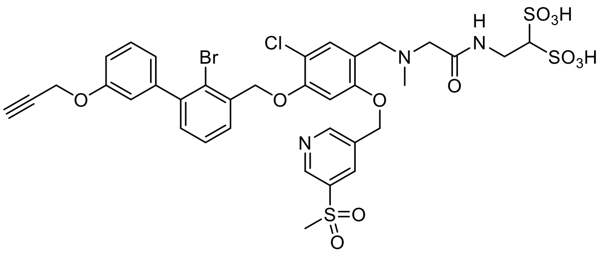

2-(2-((4-((2-bromo-3'-(prop-2-yn-1-yloxy)-[1,1'-biphenyl]-3-yl)methoxy)-5-chloro-2-((5-(methylsulfonyl)pyridin-3-yl)methoxy)benzyl)(methyl)amino)acetamido)ethane-1,1-disulfonic acid (**33a**). Carboxylic acid **30a** (369 mg, 517 µmol, 1.00 equiv.) and 2-aminoethan-1,1-disulfonic acid (TBA salt) (**32**) (1.01 g, 2.58 mmol, 5.00 equiv.) was dissolved in abs. DMF (5 mL) followed by addition of of DIPEA (180 µL, 1.03 mol, 2.00 equiv.) and HBTU (431 mg, 1.14 mmol, 2.20 equiv.). After completion the reaction was subsequently worked up according to GP-6. After purification by preparative RP-HPLC (System D, Rt = 32 min) and desalting, alkyne **33a** (334 mg, 307 µmol, 60%) was obtained as an off-white powder. mp = 195–198 °C (decomposition). R_t = 12.42 min (System A), purity: 100%. ^{1}H NMR (400 MHz, DMSO-d_6) δ = 9.61 (br, 1H), 9.17 (s, 1H), 9.11 (s, 1H), 8.57 (s, 1H), 8.14 (t, 3J = 5.0 Hz, 1H), 7.71-7.69 (m, 1H), 7.66 (s, 1H), 7.53 (t, ^{3}J = 7.6 Hz, 1H), 7.43-7.39 (m, 2H), 7.22 (s, 1H), 7.06-7.00 (m, 3H), 5.51 (s, 2H), 5.38 (s, 2H), 4.84 (d, ^{4}J = 2.3 Hz, 2H), 3.93-3.89 (m, 2H), 3.71-3.64 (m, 2H), 3.59-3.54 (m, 2H), 3.39 (s, 3H), 2.72 ppm (s, 3H). ^{13}C NMR (101 MHz, DMSO-d_6) δ = 163.5, 156.9, 156.8, 155.6, 152.4, 146.5, 142.7, 142.0, 137.2, 136.1, 135.5, 134.0, 133.4, 131.2, 129.4, 129.2, 127.8, 123.1, 122.3, 115.8, 114.1, 113.4, 111.8, 111.4, 109.5, 100.2, 79.2, 78.4, 74.1, 71.2, 67.4, 56.0, 5.55, 52.7, 43.6, 40.6 ppm. IR (ATR): ṽ = 3288 (w), 3053 (w), 2924

(w), 2162 (w), 1682 (m), 1606 (m), 1580 (w), 1506 (w), 1462 (w), 1407 (w), 1307 (m), 1201 (s), 1174 (s), 1154 (s), 1063 (m), 1019 (m), 932 (w), 778 (w), 699 (w), 674 (w), 590 cm^{-1} (w). HRMS (ESI, $[M+H]^+$) calcd for $C_{35}H_{36}BrClN_3O_{12}S_3$, 900.0328; found, 900.0317.

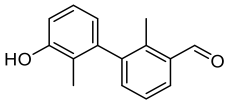

*3'-Hydroxy-2,2'-dimethyl-[1,1'-biphenyl]-3-carbaldehyde (**26**).* A solution of 3-bromo-2-methylphenol (3.93 g, 21.0 mmol, 1.00 equiv.) and 2-methyl-3-(4,4,5,5-tetramethyl-1,3,2-dioxaborolan-2-yl)benzaldehyde (**25**) (5.69 g, 23.1 mmol, 1.10 equiv.) in a 7:1 mixture of 1,4-dioxane/water (300 mL) was degassed with argon for 30 min and after addition of potassium carbonate (8.71 g, 63.0 mmol, 3.00 equiv.) and tetrakis(triphenylphosphine)palladium(0) (1.21 g, 1.05 mmol, 0.05 equiv.) in one portion, the reaction mixture was stirred under argon at 90 °C for 16 h. After complete conversion, the solvent was removed *in vacuo*, dichloromethane (400 mL) and water (350 mL) were added, the phases were separated, and the aqueous phase was extracted with dichloromethane (3 x 400 mL). The combined organic extracts were dried over anhydrous sodium sulfate, filtered and after removing the solvent *in vacuo*, the crude product was purified by flash column chromatography on silica gel (PE:EA, 10:1, R_f = 0.2) to afford 3’-hydroxy-2,2’-dimethyl-[1,1’-biphenyl]-3-carbaldehyde (**26**) (4.30 g, 19.0 mmol, 90%) as an off-white solid. mp = 140 °C. ^{1}H NMR (400 MHz, $CDCl_3$) δ = 10.38 (s, 1H), 7.84 (dd, ^4J = 1.8, ^3J = 7.4 Hz, 1H), 7.35–7.42 (m, 2H), 7.13 (t, ^3J = 7.8 Hz, 1H), 6.84 (d, ^4J = 7.8 Hz, 1H), 6.70 (d, ^4J = 7.4 Hz, 1H), 5.07 (s, 1H), 2.39 (s, 3H), 1.94 ppm (s, 3H). ^{13}C NMR (101 MHz, $CDCl_3$) δ = 193.3, 154.1, 143.4, 142.1, 138.8, 135.1, 134.5, 131.2, 126.6, 126.0, 122.5, 122.1, 114.3, 15.8, 12.7 ppm. IR (ATR): $\tilde{\nu}$ = 3332 (w), 2922 (w), 1681 (s), 1579 (m), 1449 (m), 1409 (w), 1380 (w), 1335 (w), 1307 (m), 1278 (s), 1213 (m), 1178 (w), 1144 (w), 1070 (m), 993 (w), 902 (m), 876 (w), 859 (w), 813 (w), 785 (s), 757 (m), 720 (s), 670 (m), 509 (w), 455 (w), 411 cm^{-1} (w). HRMS (ESI, $[M–H]^-$) calcd for $C_{14}H_{11}O_2$, 226.0994; found, 225.0920.

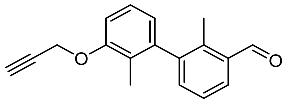

*2,2'-Dimethyl-3'-(prop-2-yn-1-yloxy)-[1,1'-biphenyl]-3-carbaldehyde (**27**).* 3’-Hydroxy-2,2’-dimethyl-[1,1’-biphenyl]-3-carbaldehyde (**26**) (2.00 g, 8.83 mmol, 1.00 equiv.) reacted with propargyl bromide (80 wt. % in toluene, 1.48 mL, 13.3 mmol, 1.50 equiv.) and potassium

carbonate (2.44 g, 17.7 mmol, 2.00 equiv.) as a base in abs. DMF (20 mL) according to GP-1. Purification by flash column chromatography on silica gel (PE:EA, 8:2, R_f = 0.4) gave 2,2'-dimethyl-3'-(prop-2-yn-1-yloxy)-[1,1'-biphenyl]-3-carbaldehyde (**27**) (2.13 g, 8.06 mmol, 91%) as a colorless oil. ^{1}H NMR (600 MHz, $CDCl_3$) δ = 10.37 (s, 1H), 7.84 (d, ^3J = 6.5 Hz, 1H), 7.40 (t, ^3J = 7.5 Hz, 1H), 7.36 (d, ^3J = 6.5 Hz, 1H), 7.22 (t, ^3J = 8.1 Hz, 1H), 7.01 (d, ^3J = 8.1 Hz, 1H), 6.77 (d, ^3J = 7.5 Hz, 1H), 4.77 (d, ^4J = 2.4 Hz, 2H), 2.55 (t, ^4J = 2.4 Hz, 1H), 2.37 (s, 3H), 1.93 ppm (s, 3H). ^{13}C NMR (151 MHz, $CDCl_3$) δ = 193.2, 156.1, 143.4, 141.9, 138.7, 135.1, 131.1, 126.3, 126.0, 125.7, 122.8, 110.9, 79.0, 75.5, 56.3, 15.8, 13.1 ppm. IR (ATR): $\tilde{\nu}$ = 3276 (m), 2924 (w), 2120 (w), 1678 (s), 1578 (m), 1451 (s), 1379 (w), 1306 (m), 1268 (m), 1241 (s), 1228 (m), 1200 (w), 1178 (m), 1139 (m), 1066 (m), 1007 (s), 926 (m), 868 (w), 788 (s), 724 (s), 695 (s), 646 (s), 595 (w), 565 (w), 502 (w), 486cm^{-1} (w). HRMS (EI, $[M]^+$): calcd for $C_{17}H_{14}O_2$, 264.1150; found, 264.1133.

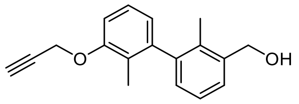

*(2,2'-Dimethyl-3'-(prop-2-yn-1-yloxy)-[1,1'-biphenyl]-3-yl)methanol (**28b**).* 2,2'-Dimethyl-3'-(prop-2-yn-1-yloxy)-[1,1'-biphenyl]-3-carbaldehyde (**27**) (2.45 g, 9.27 mmol, 1.00 equiv.) was dissolved in an 1:1 mixture of abs. DCM/MeOH (20 mL) and was cooled down to 0 °C. Sodium borohydride (0.53 g, 13.9 mmol, 1.50 equiv.) was added in small portions and after complete addition, the reaction mixture was allowed to stir at room temperature for 3 h. After complete conversion, water (50 mL) and ethyl acetate (50 mL) were added. The phases were separated, the aqueous phase was extracted with ethyl acetate (3 x 30 mL), the combined organic phases were dried over sodium sulfate, filtered and concentrated under reduced pressure. The crude product was purified by flash column chromatography on silica gel (PE:EA, 8:2, R_f = 0.3) to obtain (2,2'-dimethyl-3'-(prop-2-yn-1-yloxy)-[1,1'-biphenyl]-3-yl)methanol (**28b**) (2.20 g, 8.26 mmol, 91%) as a colorless oil. ^{1}H NMR (600 MHz, $CDCl_3$) δ = 7.39 (d, ^3J = 7.5 Hz, 1H), 7.25 (t, ^3J = 7.5 Hz, 1H), 7.20 (t, ^3J = 8.0 Hz, 1H), 7.08 (d, ^3J = 7.5 Hz, 1H), 6.98 (d, ^3J = 8.0 Hz, 1H), 6.79 (d, ^3J = 7.8 Hz, 1H), 4.76-4.77 (m, 4H), 2.54 (t, ^4J = 2.5 Hz, 1H), 2.05 (s, 3H), 1.93 ppm (s, 3H). ^{13}C NMR (151 MHz, $CDCl_3$) δ = 156.0, 143.3, 142.2, 139.0, 134.2, 129.2, 126.7, 126.0, 125.7, 125.6, 122.9, 110.5, 79.2, 75.4, 64.2, 56.3, 15.4, 13.1 ppm. IR (ATR): $\tilde{\nu}$ = 3286 (m), 2920 (m), 1676 (w), 1576 (m), 1445 (m), 1372 (m), 1310 (w), 1256 (m), 1233 (w), 1180 (m), 1143

(m), 1085 (s), 1009 (s), 925 (w), 892 (w), 784 (s), 720 (m), 650 (m), 541 cm^{-1} (w). HRMS (ESI, $[M+Na]^+$) calcd for $C_{17}H_{16}O_2Na$, 289.1204; found, 289.1204.

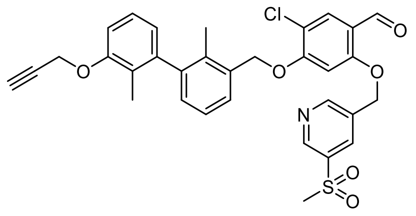

*5-Chloro-4-((2,2′-dimethyl-3′-(prop-2-yn-1-yloxy)-[1,1′-biphenyl]-3-yl)methoxy)-2-((5-(methylsulfonyl)pyridin-3-yl)me-thoxy)benzaldehyde (**29b**). (2,2′-Dimethyl-3′-(prop-2-yn-1-yloxy)-[1,1′-biphenyl]-3-yl)methanol* (**28b**) (300 mg, 878 µmol, 1.00 equiv.), phenol **11** (257 mg, 966 µmol, 1.10 equiv.) and triphenylphosphine (299 mg, 1.14 mmol, 1.30 equiv.) were dissolved in abs. DMF (3 mL) and reacted with DEAD (179 µL, 1.14 mmol, 1.30 equiv.) according to GP-2. Purification by flash column chromatography on silica gel (PE:EA, 4:6, $R_f = 0.2$) gave compound **29b** (444 mg, 752 µmol, 86%) as a colorless solid. mp = 188 °C. ^1H NMR (600 MHz, $CDCl_3$) δ = 10.26 (s, 1H), 9.18 (d, ^4J = 2.1 Hz), 9.98 (d, ^4J = 2.1 Hz), 8.38 (d, ^4J = 2.1 Hz), 7.91 (s, 1H), 7.46 (d, ^3J = 7.0 Hz, 1H), 7.28 (t, ^3J = 7.7 Hz, 1H), 7.22 (t, ^3J = 7.7 Hz, 1H), 7.16-7.17 (m, 1H), 7.00 (d, ^3J = 7.7 Hz, 1H), 6.80 (d, ^3J = 7.0 Hz, 1H), 6.69 (s, 1H), 5.26 (s, 2H), 5.26 (s, 2H), 4.77 (d, ^4J = 2.4 Hz, 2H), 3.16 (s, 3H), 2.54 (t, ^4J = 2.4 Hz, 1H), 2.09 ppm (s, 3H). ^{13}C NMR (151 MHz, $CDCl_3$) δ = 186.5, 160.2, 160.1, 156.0, 153.3, 148.7, 142.8, 142.6, 137.4, 134.8, 134.5, 133.3, 132.3, 130.9, 130.3, 127.6, 126.1, 125.9, 125.6, 122.8, 119.5, 117.6, 110.7, 98.8, 79.1, 75.5, 70.7, 68.0, 56.3, 45.0, 15.9, 13.1 ppm. IR (ATR): ṽ = 3270 (m), 2924 (w), 1662 (m), 1592 (s), 1498 (w), 1457 (m), 1436 (w), 1413 (m), 1378 (m), 1306 (s), 1274 (s), 1249 (s), 1233 (m), 1214 (w), 1177 (m), 1136 (s), 1107 (m), 1093 (w), 1025 (s), 959 (m), 906 (w), 871 (m), 817 (w), 801 (w), 768 (m), 752 (w), 717 (m), 698 (s), 698 (m), 650 (m), 592 (w), 550 (m), 529 (s), 505 (w), 432 cm^{-1} (w). HRMS (ESI, $[M+H]^+$) calcd for $C_{32}H_{29}ClNO_6S$, 590.1404; found, 590.1399.

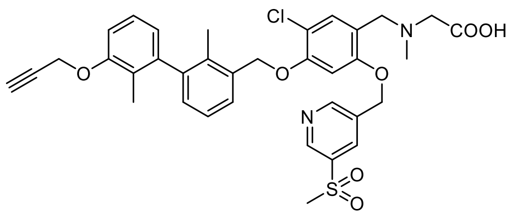

N-(5-Chloro-4-((2,2′-dimethyl-3′-(prop-2-yn-1-yloxy)-[1,1′-biphenyl]-3-yl)methoxy)-2-((5-methylsulfonyl)pyridin-3-yl)methoxy)benzyl)-N-methylglycine (**30b**). Aldehyde (**29b**) (50.0 mg,

84.7 mmol, 1.00 equiv.), sarcosine (14.6 mg, 339 µmol, 4.00 equiv.) and sodium cyanoborohydride (8.0 mg, 127 µmol, 1.50 equiv.) were reacted in a 1:1 mixture of abs. DMF/MeOH (2 mL) and subsequently worked up according to GP-3. After flash column chromatography on silica gel (MeCN:MeOH:Et_3N, 100:20:1, R_f = 0.2) compound **30b** (21.2 mg, 32.3 µmol, 38%) was obtained as a colorless solid. mp = 103 °C. R_t = 12.10 min (System A), purity: 99.6%. ^{1}H NMR (400 MHz, methanol-d_4) δ = 9.11 (s, 1H), 9.03 (s, 1H), 8.56 (s, 1H), 7.56 (s, 1H), 7.47 (d, ^3J = 7.6 Hz, 1H), 7.27-7.19 (m, 2H), 7.13-7.09 (m, 2H), 7.04 (d, ^3J = 8.3 Hz, 1H), 6.74 (d, ^3J = 7.6 Hz, 1H), 5.45 (s, 2H), 5.32 (s, 2H), 4.80 (s, 2H), 4.41 (s, 2H), 3.83 (s, 2H), 3.27 (s, 3H), 2.94 (s, 1H), 2.84 (s, 3H), 2.09 (s, 3H), 1.89 ppm (s, 3H). ^{13}C NMR (101 MHz, methanol-d_4) δ = 168.9, 158.3, 158.3, 157.3, 154.5, 149.0, 144.2, 143.7, 139.0, 136.6, 136.3, 135.7, 135.0, 130.9, 129.1, 127.1, 126.6, 126.3, 123.5, 116.7, 112.1, 112.0, 11.4, 101.1, 80.1, 76.5, 71.6, 68.9, 57.7, 57.0, 55.4, 44.5, 41.8, 15.9, 13.1 ppm. IR (ATR): ṽ = 3285 (w), 3012 (w), 2922 (w), 1732 (w), 1673 (w), 1634 (w), 1605 (m), 1575 (m), 1505 (w), 1455 (w), 1406 (w), 1380 (w), 1303 (m), 1269 (w), 1255 (w), 1234 (m), 1198 (m), 1170 (m), 1143 (s), 1089 (w), 1077 (w), 1014 (m), 965 (w), 891 (w), 798 (w), 784 (w), 766 (m), 719 (m), 694 cm^{-1} (w). HRMS (ESI, $[M-H]^-$) calcd for $C_{35}H_{34}ClN_2O_7S$, 661.1781; found, 661.1783.

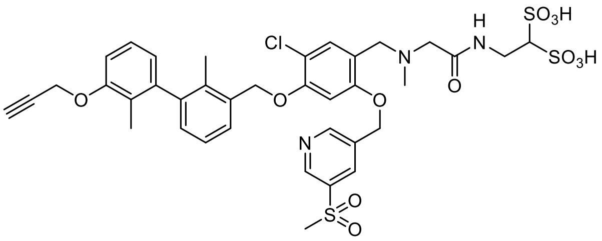

2-(2-((5-chloro-4-((2,2'-dimethyl-3'-(prop-2-yn-1-yloxy)-[1,1'-biphenyl]-3-yl)methoxy)-2-((5-(methylsulfonyl)pyridin-3-yl)methoxy)benzyl)(methyl)amino)acetamido)ethane-1,1-disulfonic acid (**33b**). Carboxylic acid (**30b**) (120 mg, 185 µmol, 1.00 equiv.) reacted with 2-aminoethane-1,1-disulfonic acid (TBA salt) (**32**) (361 mg, 924 µmol, 5.00 equiv.) in presence of DIPEA (64.5 µL, 370 µmol, 2.00 equiv.) and HTBU (154 mg, 407 µmol, 2.20 equiv.) in abs. DMF (2 mL) according to GP-6. Purification was performed by preparative RP-HPLC (System D, R_t = 25 min) and after cation exchange and lyophilization, bis(sulfonated) product **33b** (58.0 mg, 68.2 µmol, 37%) was obtained as a pale-brownish solid. mp = 180–185 °C (decomposition). R_t = 10.79 min (System A), purity: 90.3%. ^{1}H NMR (400 MHz, DMF-d_7) δ = 9.66 (br, 1H), 9.49 (s, 1H), 9.27 (s, 1H), 8.86 (s, 1H), 8.26 (s, 1H), 7.81 (s, 1H), 7.66 (d, ^3J = 7.5 Hz, 1H), 7.47 (s, 1H), 7.36-7.27 (m, 2H), 7.16-7.14 (m, 2H), 6.80 (d, ^3J = 7.5 Hz, 1H), 5.70 (s, 2H), 5.47 (s, 2H), 4.96 (s,

2H), 4.67-4.62 (m, 2H), 4.05-4.04 (m, 2H), 3.90 (s, 1H), 3.58 (t, ^4J = 2.4 Hz, 1H), 3.53 (s, 3H), 3.03 (s, 3H), 2.14 (s, 3H), 1.92 ppm (s, 3H). ^{13}C NMR (101 MHz, DMF-d$_7$) δ = 164.6, 157.6, 156.7, 156.2, 151.2, 143.2, 142.3, 138.6, 138.2, 135.3, 134.6, 129.8, 128.4, 126.5, 125.9, 124.9, 122.6, 114.4, 112.0, 111.1, 109.8, 100.6, 79.8, 77.5, 7.46, 70.4, 67.9, 57.0, 56.3, 53.8, 43.8, 41.1, 39.5, 15.4, 12.7 ppm. IR (ATR): ṽ = 3289 (m), 3055 (w), 2924 (w), 1682 (m), 1606 (w), 1576 (w), 1506 (w), 1456 (m), 1409 (w), 1307 (m), 1234 (s), 1177 (s), 1154 (s), 1090 (w), 1063 (m), 1016 (m), 768 (w), 722 (w), 674 (w), 590 cm^{-1} (w). HRMS (ESI, [M-H]$^-$) calcd for $C_{37}H_{39}ClN_3O_{12}S_3$, 848.1390; found, 848.1397.

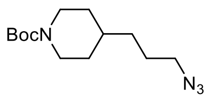

*tert-Butyl 4-(3-azidopropyl)piperidine-1-carboxylate (**58**).* *tert*-Butyl 4-(3-hydroxypropyl)piperidine-1-carboxylate (**57**) (320 mg, 1.32 mmol, 1.00 equiv.) was dissolved in abs. THF (3 mL) and ADMP (450 mg, 1.58 mmol, 1.20 equiv.) and DBU (255 µL, 1.71 mmol, 1.30 equiv.) were added. The reaction mixture was stirred at room temperature for 16 h and after complete conversion, DCM (10 mL) and conc. NH_4Cl solution (10 mL) were added, phases were separated and the aqueous phase was extracted with DCM (3 x 10 mL). The combined organic fractions were dried over sodium sulfate, filtered and concentrated under reduced pressure. Purification by flash column chromatography on silica gel (DCM:EA, 90:10, R_f = 0.3) lead to *tert*-Butyl 4-(3-azidopropyl)piperidine-1-carboxylate (**58**) (300 mg, 1.18 mmol, 85%) as a colorless oil. ^{1}H NMR (400 MHz, $CDCl_3$) δ = 4.10-4.06 (m, 2H), 3.26 (t, ^3J = 6.9 Hz, 2H), 2.71-2.64 (m, 2H), 1.64-1.60 (m, 4H), 1.45 (s, 9H), 1.40-1.39 (m, 1H), 1.34-1.28 (m, 2H), 1.14-1.04 ppm (m, 2H). ^{13}C NMR (101 MHz, $CDCl_3$) δ = 155.0, 79.4, 51.8, 44.1, 35.9, 33.7, 32.2, 28.6, 26.2 ppm. IR (ATR): $\tilde{\nu}$ = 2975 (w), 2928 (m), 2851 (w), 2092 (s), 1688 (s), 1452 (w), 1419 (m), 1364 (m), 1276 (m), 1243 (m), 1161 (s), 1134 (m), 1089 (w), 971 (w), 934 (w), 868 (w), 768 cm^{-1} (w). HRMS (ESI, [M+Na]$^+$) calcd for $C_{13}H_{24}N_4O_2Na$, 291.1791; found, 291.1790.

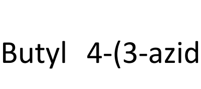

*4-(3-Azidopropyl)piperidine (**35**).* *tert*-Butyl 4-(3-azidopropyl)piperidine-1-carboxylate (**58**) (300 mg, 1.12 mmol, 1.00 equiv.) was dissolved at 0 °C in an 1:1 mixture of DCM/TFA (3 mL) and the mixture was stirred at room temperature for 16 h. The solvents were removed *in*

vacuo to yield 4-(3-azidopropyl)piperidine (**35**) (187 mg, 1.12 mmol, quant.) as a yellowish oil, which was used without further purification. ^{1}H NMR (400 MHz, $CDCl_3$) δ = 5.99 (br, 1H), 3.28-3.20 (m, 4H), 2.74-2.67 (m, 2H), 1.78-1.75 (m, 2H), 1.64-1.57 (m, 2H), 1.45-1.40 (m, 1H), 1.36-1.29 ppm (m, 4H). ^{13}C NMR (400 MHz, $CDCl_3$) δ = 51.7, 45.5, 35.1, 33.7, 31.3, 26.0 ppm. IR (ATR): $\tilde{\nu}$ = 2927 (m), 2854 (m), 2510 (w), 2360 (w), 2092 (s), 1673 (s), 1454 (w), 1256 (w), 1199 (m), 1174 (m), 1127 (m), 831 (w), 798 (w), 720 cm^{-1} (m). HRMS (EI, $[M]^+$) calcd for $C_8H_{16}N_4$, 168.1375; found, 168.1359.

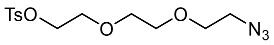

*2-(2-(2-Azidoethoxy)ethoxy)ethyl 4-methylbenzenesulfonate (**59**).* 2-(2-(2-Azidoethoxy)ethoxy)ethan-1-ol (**38**) (230 mg, 1.31 mmol, 1.00 equiv.) was dissovled in abs. DCM (5 mL) under argon. After the addition of tosylchlorid (376 mg, 1.97 mmol, 1.50 equiv.) and pyridine (212 µL, 2.63 mmol, 2.00 equiv.) at 0 °C, the reaction mixture was stirred at room temperature for 16 h. The mixture was diluted with ethyl acetate (50 mL), 1M HCl (50 mL) was added, the phases were separated and the aqueous phase was extracted with ethyl acetate (3 x 40 mL). The combined organic fractions were dried over sodium sulfate, filtered and concentrated under reduced pressure. Purification by flash column chromatography on silica gel (PE:EA, 7:3, R_f = 0.3) yielded 2-(2-(2-azidoethoxy)ethoxy)ethyl 4-methylbenzenesulfonate (**59**) (404 mg, 1.23mmol, 93%) as a colorless oil. ^{1}H NMR (400 MHz, $CDCl_3$) δ = 7.80 (d, ^3J = 8.3 Hz, 2H), 7.34 (d, ^3J = 8.3 Hz, 2H), 4.18-4.15 (m, 2H), 3.71-3.69 (m, 2H), 3.65-3.63 (m, 2H), 3.60 (s, 4H), 3.38-3.35 (m, 2H), 2.45 ppm (s, 3H). ^{13}C NMR (101 MHz, $CDCl_3$) δ = 144.9, 133.2, 130.0, 128.1, 70.9, 70.8, 70.2, 69.4, 68.9, 50.8, 21.8 ppm. IR (ATR): $\tilde{\nu}$ = 2952 (m), 2921 (m), 2854 (w), 2100 (m), 1598 (w), 1454 (w), 1354 (m), 1290 (w), 1189 (m), 1175 (s), 11230 (m), 1096 (m), 1017 (w), 919 (m), 816 (m), 774 (w), 690 (w), 662 (m), 584 (w), 553 cm^{-1} (m). HRMS (ESI, $[M+H]^+$) calcd for $C_{13}H_{20}N_3O_5S$, 352.0943; found, 352.0936.

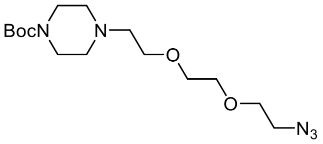

*tert-Butyl 4-(2-(2-(2-azidoethoxy)ethoxy)ethyl)piperazine-1-carboxylate (**60**).* *tert*-Butyl piperazine-1-carboxylate (190 mg, 1.02 mmol, 1.00 equiv.) reacted with 2-(2-(2-azidoethoxy)ethoxy)ethyl 4-methylbenzenesulfonate (**59**) (386 mg, 1.17 mmol, 1.15 equiv.)

and potassium carbonate (282 mg, 2.04 mmol, 2.00 equiv.) as a base in abs. DMF (5 mL) according to GP-1. Purification by flash column chromatography on silica gel (DCM:MeOH, 94:6, R_f = 0.2) provided *tert*-butyl 4-(2-(2-(2-azidoethoxy)ethoxy)ethyl)piperazine-1-carboxylate (**60**) (255 mg, 724 µmol, 73%) as a colorless oil. ^{1}H NMR (400 MHz, methanol-d$_4$) δ = 3.71–3.66 (m, 8H), 3.46–3.39 (m, 6H), 2.66–2.63 (m, 2H), 2.54–2.51 (m, 4H), 1.48 ppm (s, 9H). ^{13}C NMR (101 MHz, methanol-d$_4$) δ = 156.4, 81.2, 71.6, 71.5, 71.1, 69.7, 62.2, 58.7, 54.3, 51.8, 286 ppm. IR (ATR): $\tilde{\nu}$ = 2974 (w), 2927 (m), 2865 (m), 2810 (w), 2100 (m), 1690 (s), 1457 (w), 1418 (m), 1392 (w), 1365 (m), 1289 (m), 1244 (m), 1169 (m), 1119 (s), 1004 (m), 937 (w), 865 (w), 831 (w), 796 cm^{-1} (w). HRMS (ESI, [M+Na]$^+$) calcd for $C_{15}H_{29}N_5O_4Na$, 344.2298; found, 344.2293.

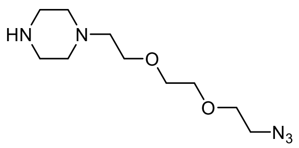

*1-(2-(2-(2-Azidoethoxy)ethoxy)ethyl)piperazine (**39**).* *tert*-Butyl 4-(2-(2-(2-azidoethoxy)ethoxy)ethyl)piperazine-1-carboxylate (**60**) (90.0 mg, 262 µmol, 1.00 equiv.) was dissolved at 0 °C in an 1:1 mixture of DCM/TFA (3 mL) and was stirred at room temperature for 16 h. The solvents were removed *in vacuo* to yield 1-(2-(2-(2-azidoethoxy)ethoxy)ethyl)piperazine (**39**) (63.8 mg, 261 µmol, quant.) as a yellow oil, which was used without further purification. ^{1}H NMR (400 MHz, methanol-d$_4$) δ = 3.85–3.82 (m, 2H), 3.70–3.68 (m, 6H), 3.49–3.46 (m, 4H), 3.43–3.40 (m, 2H), 3.38–3.37 (m, 4H), 3.24–3.21 ppm (m, 2H). ^{13}C NMR (101 MHz, methanol-d$_4$) δ = 71.5, 71.4, 71.0, 67.2, 57.9, 51.8, 50.6, 43.0 ppm. IR (ATR): $\tilde{\nu}$ = 3445 (w), 3015 (w), 2873 (w), 2747 (w), 2487 (w), 2360 (m), 2342 (w), 2110 (m), 1668 (s), 1456 (m), 1423 (m), 1302 (w), 1176 (s), 1121 (s), 1009 (w), 916 (w), 832 (m), 798 (m), 720 (m), 595 cm^{-1} (w). HRMS (ESI, [M+H]$^+$) calcd for $C_{10}H_{22}N_5O_2$, 244.1773; found, 244.1767.

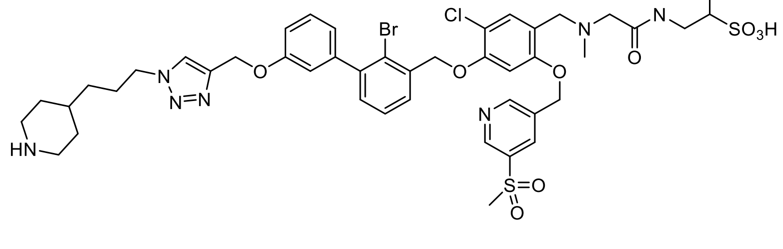

2-(2-((4-((2-Bromo-3'-((1-(3-(piperidin-4-yl)propyl)-1H-1,2,3-triazol-4-yl)methoxy)-[1,1'-biphenyl]-3-yl)methoxy)-5-chloro-2-((5-(methylsulfonyl)pyridin-3-

yl)methoxy)benzyl)(methyl)amino)acetamido)ethane-1,1-disulfonic acid (**40a**). Alkyne **33a** (15.0 mg, 16.4 µmol, 1.00 equiv.) and azide linker **35** (4.1 mg, 24.6 µmol, 1.50 equiv.) were dissolved in DMF (1 mL), reacted in presence of TBTA (0.1 mg, 0.2 µmol, 0.01 equiv.) and $[Cu(MeCN)_4]PF_6$ (2.5 mg, 6.8 µmol, 0.50 equiv.) according to GP-4. Purification by semi-preparative RP-HPLC (System C, R_t = 21 min) yielded compound **40a** (11.5 mg, 10.8 µmol, 66%) as a colorless powder. Mp = 140 °C (decomposition). R_t = 9.42 min (System A), purity: 97.0%. ^{1}H NMR (400 MHz, DMSO-d$_6$) δ = 9.55 (br, 1H), 9.12 (br, 2H), 8.48 (s, 1H), 8.29 (s, 1H), 8.22 (s, 1H), 8.06 (br, 2H), 7.68–7.64 (m, 2H), 7.53–7.49 (m, 1H), 7.41–7.37 (m, 2H), 7.20 (s, 1H), 7.09 (d, ^3J = 8.1 Hz, 1H), 7.04 (s, 1H), 6.96 (d, ^3J = 7.5 Hz, 1H), 5.49–5.48 (m, 2H), 5.38 (s, 2H), 4.37–4.33 (m, 4H), 3.82 (s, 2H), 3.61–3.54 (m), 3.22–3.19 (m, 2H), 2.73 (s, 4H), 1.80–1.79 (m, 2H), 1.69–1.66 (m, 2H), 1.53–1.45 (m, 2H), 1.28–1.23 (m, 2H), 1.14–1.12 ppm. (m, 2H). ^{13}C NMR (101 MHz, DMSO-d$_6$) δ = 163.4, 157.6, 156.9, 155.6, 153.3, 151.2, 147.3, 142.8, 142.6, 142.1, 136.0, 134.4, 134.0, 131.3, 129.6, 129.4, 128.0, 127.7, 125.5, 124.4, 123.3, 121.8, 115.9, 114.3, 113.4, 109.8, 100.2, 73.8, 71.2, 67.5, 61.3, 53.8, 50.6, 49.4, 43.7, 43.3, 41.0, 32.5, 32.1, 28.3, 28.1, 26.7, 25.2 ppm. IR (ATR): ṽ = 3445 (m), 3055 (m), 2928 (m), 2857 (w), 1682 (m), 1605 (w), 1580 (w), 1506 (w), 1456 (w), 1406 (w), 1304 (m), 1200 (s), 1146 (s), 1064 (m), 1021 (m), 799 (w), 779 (w), 719 (w), 700 (w), 652 (w), 596 cm^{-1} (w). HRMS (ESI, $[M+H]^+$) calcd for $C_{43}H_{52}BrClN_7O_{12}S_3$, 1068.1703; found, 1068.1697.

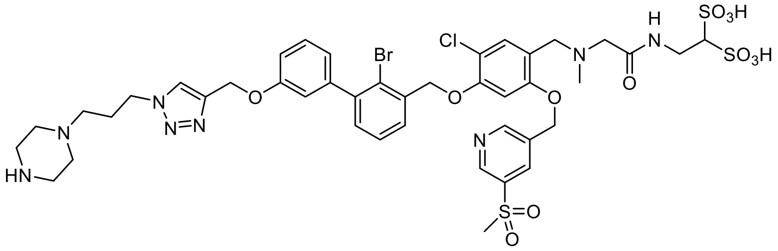

2-(2-((4-((2-Bromo-3'-((1-(3-(piperazin-1-yl)propyl)-1H-1,2,3-triazol-4-yl)methoxy)-[1,1'-biphenyl]-3-yl)methoxy)-5-chloro-2-((5-(methylsulfonyl)pyridin-3-yl)methoxy)benzyl)(methyl)amino)acetamido)ethane-1,1-disulfonic acid (**40b**). Alkyne **33a** (15.0 mg, 16.4 µmol, 1.00 equiv.) and azide linker **37** (3.5 mg, 24.6 µmol, 1.50 equiv.) were dissolved in DMF (1 mL), reacted in presence of TBTA (0.1 mg, 0.2 µmol, 0.01 equiv.) and $[Cu(MeCN)_4]PF_6$ (2.5 mg, 6.8 µmol, 0.50 equiv.) according to GP-4. Purification by semi-preparative RP-HPLC (System C, R_t = 20 min) yielded compound **40b** (5.1 mg, 4.8 µmol, 29%) as a colorless powder. mp = 150–153 °C. R_t = 10.06 min (System A), purity: 100%. ^{1}H NMR

(400 MHz, DMSO-d_6) δ = 9.69 (br, 1H), 9.06–9.05 (m, 2H), 8.45 (s, 1H), 8.25 (s, 1H), 8.06 (s, 1H), 7.67–7.63 (m, 2H), 7.50 (t, ^3J = 7.5 Hz, 2H), 7.41–7.37 (m, 2H), 7.15 (s, 1H), 7.09 (d, ^3J = 8.1 Hz, 1H), 7.04 (s, 1H), 6.95 (d, ^3J = 7.6 Hz, 1H), 5.43 (s, 4H), 5.19 (s, 2H), 4.45 (br, 2H), 4.27 (br, 2H), 3.75–3.37 (m), 2.73 (s, 2H), 2.24 ppm (br, 2H). ^{13}C NMR (101 MHz, DMSO-d_6) δ = 163.6, 158.1, 157.8, 157.5, 156.9, 155.5, 153.2, 147.3, 142.9, 142.6, 142.0, 136.9, 136.1, 134.4, 134.0, 132.8, 131.3, 129.7, 129.5, 127.8, 124.7, 123.4, 121.7, 116.0, 114.2, 113.5, 109.6, 100.3, 73.9, 71.2, 67.4, 61.2, 55.3, 53.8, 52.7, 48.5, 46.9, 43.7, 41.2, 38.7 ppm. IR (ATR): ṽ = 3440 (w), 3020 (w), 1679 (m), 1605 (w), 1575 (w), 1505 (w), 1456 (w), 1409 (w), 1305 (m), 1235 (m), 1199 (s), 1178 (s), 1145 (s), 1063 (m), 1017 (m), 969 (w), 799 (w), 721 (w), 593 cm^{-1} (w). HRMS (ESI, [M+H]$^+$) calcd for $C_{42}H_{52}BrClN_8O_{12}S_3$, 1071.1640; found, 1071.1640.

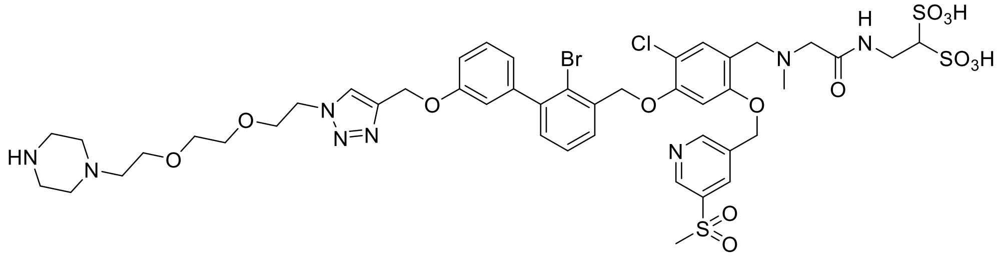

2-(2-((4-((2-Bromo-3'-((1-(2-(2-(2-(piperazin-1-yl)ethoxy)ethoxy)ethyl)-1H-1,2,3-triazol-4-yl)methoxy)-[1,1'-biphenyl]-3-yl)methoxy)-5-chloro-2-((5-(methylsulfonyl)pyridin-3-yl)methoxy)benzyl)(methyl)amino)acetamido)ethane-1,1-disulfonic acid (**40c**). Alkyne **33a** (20.0 mg, 21.9 µmol, 1.00 equiv.) and azide linker **39** (8.0 mg, 32.8 µmol, 1.50 equiv.) were dissolved in DMF (1 mL), reacted in presence of TBTA (0.1 mg, 0.2 µmol, 0.01 equiv.) and [Cu(MeCN)$_4$]PF$_6$ (3.5 mg, 9.4 µmol, 0.50 equiv.) according to GP-4. Purification by semi-preparative RP-HPLC (System C, R_t = 25 min) yielded compound **40c** (17.0 mg, 14.9 µmol, 68%) as a colorless powder. mp = 165–168 °C. R_t = 9.06 min (System A), purity: 95.6%. ^{1}H NMR (400 MHz, DMSO-d_6) δ = 9.67 (br, 1H), 9.07 (s, 2H), 8.47 (s, 1H), 8.22 (s, 1H), 8.07 (br, 1H), 7.67–7.63 (m, 2H), 7.51 (t, ^3J = 7.5 Hz, 1H), 7.42–7.38 (m, 2H), 7.18 (br, 1H), 7.11–7.09 (m, 1H), 7.06 (br, 1H), 6.95 (d, ^3J = 7.5 Hz, 1H), 5.47 (s, 2H), 5.39 (s, 2H), 5.20 (s, 2H), 4.55–4.53 (m, 2H), 4.28 (br, 2H), 3.84–3.37 (m), 2.75 ppm (s, 3H). ^{13}C NMR (101 MHz, DMSO-d_6) δ = 157.5, 156.9, 155.6, 153.3, 147.3, 142.9, 142.5, 142.1, 136.9, 136.0, 134.5, 134.0, 132.8, 131.3, 129.6, 129.4, 127.8, 124.9, 132.4, 121.8, 115.8, 114.3, 113.5, 109.6, 100.2, 73.9, 71.3, 69.3, 69.2, 68.6, 67.5, 64.7, 61.2, 55.7, 52.9, 49.5, 48.6, 43.7, 41.6, 41.5 ppm. IR (ATR): ṽ = 3444 (w), 3023 (w), 2874 (w), 1679 (m), 1605 (w), 1580 (w), 1506 (w), 1461 (w), 1406 (w), 1304 (m), 1199 (s), 1175 (s),

1145 (s), 1063 (m), 1019 (m), 916 (w), 829 (w), 798 (w), 778 (w), 720 (w), 700 (w), 650 (w), 593 cm^{-1} (w). HRMS (ESI, $[M+H]^+$) calcd for $C_{45}H_{57}BrClN_8O_{14}S_3$, 1143.2023; found, 1143.2010.

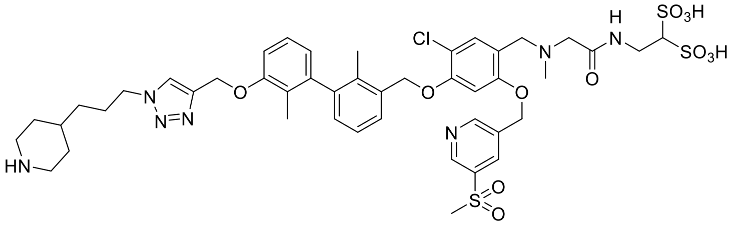

2-(2-((5-Chloro-4-((2,2'-dimethyl-3'-((1-(3-(piperidin-4-yl)propyl)-1H-1,2,3-triazol-4-yl)methoxy)-[1,1'-biphenyl]-3-yl)methoxy)-2-((5-(methylsulfonyl)pyridin-3-yl)methoxy)benzyl)(methyl)amino)acetamido)ethane-1,1-disulfonic acid (**41a**). Alkyne **33b** (26.0 mg, 30.6 µmol, 1.00 equiv.) and azide linker **35** (7.7 mg, 45.9 µmol, 1.50 equiv.) were dissolved in DMF (1 mL), reacted in presence of TBTA (0.1 mg, 0.2 µmol, 0.01 equiv.) and $[Cu(MeCN)_4]PF_6$ (4.3 mg, 11.5 µmol, 0.50 equiv.) according to GP-4. Purification by semi-preparative RP-HPLC (System C, R_t = 20 min) yielded compound **41a** (19.4 mg, 18.6 µmol, 61%) as a colorless powder. mp = 197–200 °C (decomposition). R_t = 9.24 min (System A), purity: 96.2%. ^1H NMR (400 MHz, DMSO-d_6) δ = 9.61 (br, 1H), 9.10 (br, 2H), 8.49 (s, 1H), 8.26–8.22 (m, 2H), 8.05 (br, 2H), 7.61 (s, 1H), 7.50 (d, ^3J = 7.6 Hz, 1H), 7.29–7.21 (m, 2H), 7.15 (d, ^3J = 8.1 Hz, 1H), 7.08 (d, ^3J = 7.7 Hz, 1H), 6.73 (d, ^3J = 7.5 Hz, 1H), 5.49–5.45 (m, 2H), 5.35–5.34 (m, 2H), 5.25–5.16 (m, 2H), 4.38–4.27 (m, 3H), 3.83 (br, 2H), 3.61–3.38 (m), 3.21–3.18 (m, 2H), 2.74–2.73 (m, 4H), 2.02 (s, 3H), 1.83–1.77 (m, 4H), 1.68–1.64 (m, 2H), 1.44 (br, 2H), 1.29–1.23 (m, 3H), 1.12–1.10 ppm (m, 2H). ^{13}C NMR (101 MHz, DMSO-d_6) δ = 163.4, 156.0, 154.2, 153.3, 151.9, 147.3, 143.1, 142.2, 141.6, 134.5, 134.4, 133.9, 129.3, 128.0, 126.3, 125.5, 124.3, 124.1, 121.7, 113.4, 109.6, 100.3, 73.9, 69.9, 67.4, 61.9, 55.7, 52.9, 49.3, 43.7, 43.3, 41.3, 32.4, 32.0, 28.1, 26.7, 15.3, 12.8 ppm. IR (ATR): ṽ = 3451 (w), 3015 (w), 2929 (w),1682 (m), 1606 (w), 1576 (w), 1506 (w), 1456 (m), 1409 (w), 1306 (m), 1199 (s), 1179 (s), 1146 (s), 1091 (w), 1064 (w), 1019 (m), 798 (w), 769 (w), 720 (w), 592 cm^{-1} (w). HRMS (ESI, $[M+H]^+$) calcd for $C_{45}H_{57}ClN_7O_{12}S_3$, 1018.2911; found, 1018.2910.

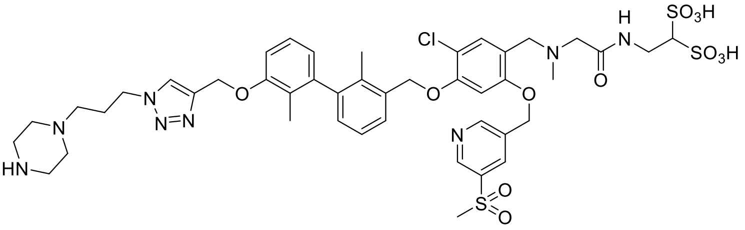

2-(2-((5-Chloro-4-((2,2'-dimethyl-3'-((1-(3-(piperazin-1-yl)propyl)-1H-1,2,3-triazol-4-yl)methoxy)-[1,1'-biphenyl]-3-yl)methoxy)-2-((5-(methylsulfonyl)pyridin-3-yl)methoxy)benzyl)(methyl)amino)acetamido)ethane-1,1-disulfonic acid (**41b**). Alkyne **33b** (15.0 mg, 17.6 µmol, 1.50 equiv.) and azide linker **37** (3.7 mg, 26.5 µmol, 1.50 equiv.) were dissolved in DMF (1 mL), reacted in presence of TBTA (0.1 mg, 0.2 µmol, 0.01 equiv.) and [Cu(MeCN)$_4$]PF$_6$ (2.6 mg, 6.9 µmol, 0.50 equiv.) according to GP-4. Purification by semi-preparative RP-HPLC (System C, R$_t$ = 20 min) yielded compound **41b** (8.1 mg, 7.8 µmol, 45%) as a colorless powder. mp = 155–159 °C. R$_t$ = 9.09 min (System A), purity: 98.8%. ^{1}H NMR (400 MHz, DMSO-d$_6$) δ = 9.66 (br, 1H), 9.09 (br, 2H), 8.47 (s, 1H), 8.27 (s, 1H), 8.12 (s, 1H), 7.59 (s, 1H), 7.50 (d, ^{3}J = 7.3 Hz, 1H), 7.25–7.15 (m, 3H), 7.07 (d, ^{3}J = 7.4 Hz, 1H), 6.72 (d, ^{3}J = 7.4 Hz, 1H), 5.42-5.38 (m, 4H), 5.23–5.16 (m, 2H), 4.48–4.45 (m, 2H), 4.26 (br, 2H), 3.78–3.37 (m), 3.04 (br, 2H), 2.70 (s, 3H), 2.26 (br, 2H), 2.01 (s, 3H), 1.73 ppm (s, 3H). ^{13}C NMR (101 MHz, DMSO-d$_6$) δ = 163.6, 156.8, 156.1, 155.5, 153.2, 147.4, 143.2, 142.2, 141.7, 137.0, 135.0, 134.4, 134.4, 133.8, 132.8, 129.3, 128.3, 126.4, 125.5, 124.3, 124.2, 121.6, 113.6, 110.9, 109.6, 100.5, 73.9, 70.0, 67.4, 61.9, 55.5, 53.7, 52.9, 48.4, 46.9, 43.7, 41.1, 24.3, 15.5, 12.7 ppm. IR (ATR): ṽ = 3443 (w), 3022 (w), 1679 (m), 1606 (w), 1576 (w), 1505 (w), 1456 (w), 1409 (w), 1305 (m), 1236 (m), 1199 (s), 1179 (s), 1145 (s), 1063 (w), 1017 (m), 969 (w), 199 (w), 721 (w), 594 cm^{-1} (w). HRMS (ESI, [M+H]$^+$) calcd for $C_{44}H_{56}ClN_8O_{12}S_3$, 1018.2911; found, 1018.2910.

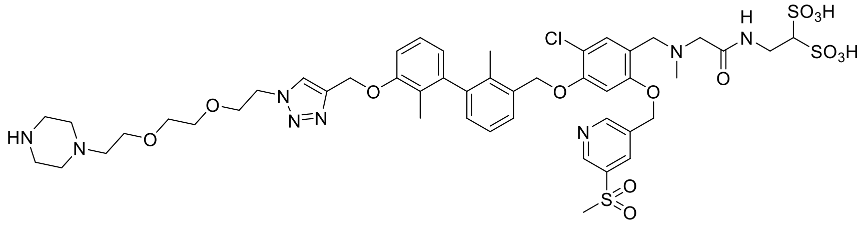

2-(2-((5-Chloro-4-((2,2'-dimethyl-3'-((1-(2-(2-(2-(piperazin-1-yl)ethoxy)ethoxy)ethyl)-1H-1,2,3-triazol-4-yl)methoxy)-[1,1'-biphenyl]-3-yl)methoxy)-2-((5-(methylsulfonyl)pyridin-3-yl)methoxy)benzyl)(methyl)amino)acetamido)ethane-1,1-disulfonic acid (**41c**). Alkyne **33b** (20.0 mg, 23.5 µmol, 1.00 equiv.) and azide linker **39** (8.6 mg, 35.3 µmol, 1.50 equiv.) were dissolved in DMF (1 mL), reacted in presence of TBTA (0.1 mg, 0.2 µmol, 0.01 equiv.) and [Cu(MeCN)$_4$]PF$_6$ (3.6 mg, 9.5 µmol, 0.50 equiv.) according to GP-4. Purification by semi-preparative RP-HPLC (System C, R$_t$ = 22 min) yielded compound **41c** (14.0 mg, 12.8 µmol, 54%) as a colorless powder. mp = 173–176 °C. R$_t$ = 9.17 min (System A), purity: 89.8%. ^{1}H NMR (400 MHz, DMSO-d$_6$) δ = 9.64 (br, 1H), 9.09 (s, 2H), 8.49 (s, 1H), 8.21 (s, 1H), 8.10 (br, 1H), 7.59

(s, 1H), 7.50–7.48 (m, 1H), 7.29–7.17 (m, 3H), 7.09 (d, ^{3}J = 7.5 Hz, 1H), 6.73 (d, ^{3}J = 7.2 Hz, 1H), 5.46 (br, 2H), 5.35 (s, 2H), 5.25–5.17 (m, 2H), 4.56–4.54 (m, 2H), 4.26 (br, 2H), 3.85–3.22 (m), 2.74 (s, 3H), 2.02 (s, 3H), 1.77 (s, 3H). ^{13}C NMR (101 MHz, DMSO-d$_6$) δ = 156.1, 153.3, 147.3, 143.1, 142.2, 141.67, 136.9, 134.5, 132.8, 128.2, 126.4, 125.5, 124.7, 124.2, 121.7, 113.5, 111.0, 109.6, 100.4, 73.9, 69.4, 69.2, 68.7, 67.4, 61.9, 55.8, 53.0, 49.4, 48.6, 43.6, 41.6, 15.4, 12.8 ppm. IR (ATR): ṽ = 3445 (w), 3022 (w), 2874 (w), 1682 (m), 1606 (w), 1576 (w), 1506 (w),1456 (m), 1409 (w), 1305 (m), 1235 (m), 1199 (s), 1178 (s), 1145 (s), 1062 (m), 1016 (m), 916 (w), 828 (w), 799 (w), 767 (w), 720 (m), 649 (w), 594 cm^{-1} (w). HRMS (ESI, [M+H]$^+$) calcd for $C_{47}H_{62}ClN_8O_{14}S_3$, 1093.3231; found, 1093.3225.

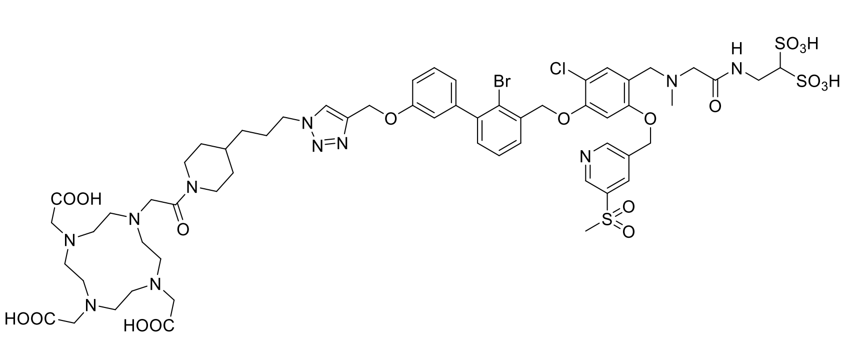

2,2',2''-(10-(2-(4-(3-(4-(((2'-Bromo-3'-((2-chloro-4-(((2-((2,2-disulfoethyl)amino)-2-oxoethyl)(methyl)amino)methyl)-5-((5-(methylsulfonyl)pyridin-3-yl)methoxy)phenoxy)methyl)-[1,1'-biphenyl]-3-yl)oxy)methyl)-1H-1,2,3-triazol-1-yl)propyl)piperidin-1-yl)-2-oxoethyl)-1,4,7,10-tetraazacyclododecane-1,4,7-triyl)triacetic acid (**42a**). Propyl-piperidinyl linker structure **40a** (7.0 mg, 6.5 µmol, 1.00 equiv.), DOTA-*p*-nitrophenylester (**61**) (6.9 mg, 13.1 µmol, 2.00 equiv.) and abs. DIPEA (5.7 µL, 32.7 µmol, 5.00 equiv.) were dissolved in abs. DMF (1 mL) and reacted according to GP-7. Purification by semi-preparative RP-HPLC (System C, R_t = 21 min) and subsequent lyophilization yielded DOTA-conjugate **42a** (3.4 mg, 2.3 µmol, 36%) as a colorless powder. mp = 185 °C (decomposition). R_t = 9.41 min (System A), purity: 99.8%. IR (ATR): ṽ = 3406 (m), 2921 (w), 1651 (m), 1644 (m), 1462 (w), 1434 (w), 1302 (m), 1200 (s), 1145 (s), 1060 (w), 1015 (s), 699 (w), 594 cm^{-1} (w). HRMS (ESI, [M+H]$^+$) calcd for $C_{59}H_{79}BrClN_{11}O_{19}S_3$, 1456.3489; found, 1456.3488.

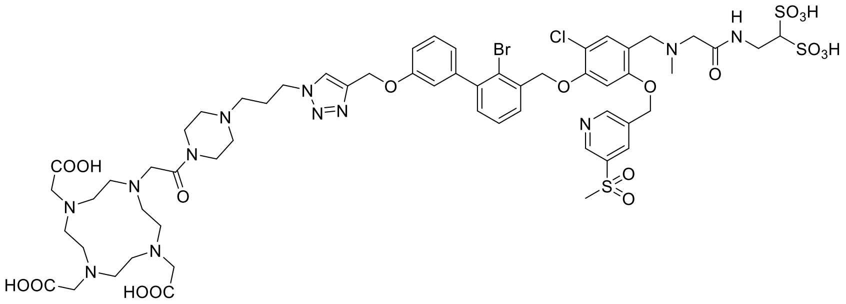

2,2',2''-(10-(2-(4-(3-(4-(((2'-Bromo-3'-((2-chloro-4-(((2-((2,2-disulfoethyl)amino)-2-oxoethyl)(methyl)amino)methyl)-5-((5-(methylsulfonyl)pyridin-3-yl)methoxy)phenoxy)methyl)-[1,1'-biphenyl]-3-yl)oxy)methyl)-1H-1,2,3-triazol-1-yl)propyl)piperazin-1-yl)-2-oxoethyl)-1,4,7,10-tetraazacyclododecane-1,4,7-triyl)triacetic acid (**42b**). Propyl-piperazinyl linker structure **40b** (4.0 mg, 3.7 µmol, 1.00 equiv.), DOTA-*p*-nitrophenylester (**61**) (3.9 mg, 7.5 µmol, 2.00 equiv.) and abs. DIPEA (3.3 µL, 18.7 µmol, 5.00 equiv.) were dissolved in abs. DMF (1 mL) and reacted according to GP-7. Purification by semi-preparative RP-HPLC (System C, R_t = 19 min) and subsequent lyophilization yielded DOTA-conjugate **42b** (4.1 mg, 2.8 µmol, 75%) as a colorless powder. mp = 181–184 °C (decomposition). R_t = 9.37 min (System A), purity: 97.5%. IR (ATR): ṽ = 3434 (w), 1645 (m), 1456 (w), 1304 (w), 1200 (m), 1145 (m), 1089 (w), 1061 (w), 1015 (m), 694 cm^{-1} (w). HRMS (ESI, $[M+H]^+$) calcd for $C_{58}H_{77}BrClN_{12}O_9S_3$, 1457.3462; found, 1457.3477.

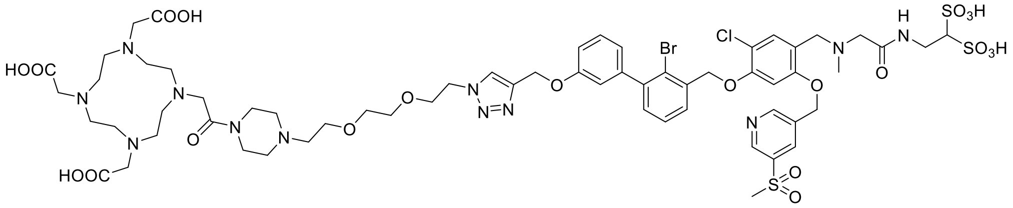

2,2',2''-(10-(2-(4-(2-(2-(2-(4-(((2'-Bromo-3'-((2-chloro-4-(((2-((2,2-disulfoethyl)amino)-2-oxoethyl)(methyl)amino)methyl)-5-((5-(methylsulfonyl)pyridin-3-yl)methoxy)phenoxy)methyl)-[1,1'-biphenyl]-3-yl)oxy)methyl)-1H-1,2,3-triazol-1-yl)ethoxy)ethoxy)ethyl)piperazin-1-yl)-2-oxoethyl)-1,4,7,10-tetraazacyclododecane-1,4,7-triyl)triacetic acid (**42c**). PEG-2-Piperazinyl linker structure **40c** (17.0 mg, 14.9 µmol, 1.00 equiv.), DOTA-nitrophenylester (**61**) (15.6 mg, 29.7 µmol, 2.00 equiv.) and abs. DIPEA (12.9 µL, 74.3 µmol, 5.00 equiv.) were dissolved in abs. DMF (1) and reacted according to GP-7. Purification by semi-preparative RP-HPLC (System C, R_t = 17 min) and subsequent lyophilization yielded DOTA-conjugate **42c** (9.6 mg, 6.3 µmol, 42%) as a colorless powder.

mp = 190 °C (decomposition). R_t = 8.85 min (System A), purity: 100%. IR (ATR): ṽ = 3428 (w), 1668 (s), 1606 (m), 1462 (w), 1403 (w), 1303 (m), 1199 (s), 1145 (s), 1062 (w), 1018 (m), 720 (w), 594 cm^{-1} (w). HRMS (ESI, $[M+H]^+$) calcd for $C_{61}H_{83}BrClN_{12}O_{21}S_3$, 1531.3809; found, 1531.3806.

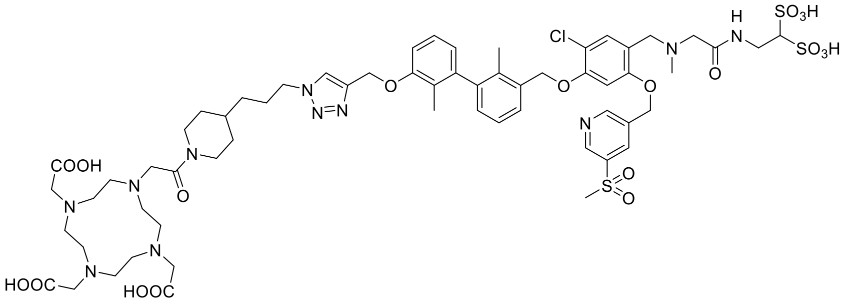

2,2',2''-(10-(2-(4-(3-(4-(((3'-((2-Chloro-4-(((2-((2,2-disulfoethyl)amino)-2-oxoethyl)(methyl)amino)methyl)-5-((5-(methylsulfonyl)pyridin-3-yl)methoxy)phenoxy)methyl)-2,2'-dimethyl-[1,1'-biphenyl]-3-yl)oxy)methyl)-1H-1,2,3-triazol-1-yl)propyl)piperidin-1-yl)-2-oxoethyl)-1,4,7,10-tetraazacyclododecane-1,4,7-triyl)triacetic acid (**43a**). Propyl-piperidinyl linker structure **41a** (8.0 mg, 7.8 µmol, 1.00 equiv.), DOTA-*p*-nitrophenylester (**61**) (8.2 mg, 15.5 µmol, 2.00 equiv.) and abs. DIPEA (6.8 µL, 38.8 µL, 5.00 equiv.) were dissolved in abs. DMF (1 mL) and reacted according to GP-7. Purification by semi-preparative RP-HPLC (System C, R_t = 23 min) and subsequent lyophilization yielded DOTA-conjugate **43a** (4.0 mg, 2.8 mol, 37%) as a colorless powder. mp = 195 °C (decomposition). R_t = 9.57 min (System A), purity: 99.7%. IR (ATR): ṽ = 3406 (m), 2921 (w), 1652 (m), 1462 (w), 1302 (m), 1200 (s), 1145 (s), 1015 (s), 699 (w), 594 cm^{-1} (w). HRMS (ESI, $[(M+2H)/2]^+$) calcd for $C_{61}H_{83}ClN_{11}O_{19}S_3$, 1405.4751; found, 1405.4727.

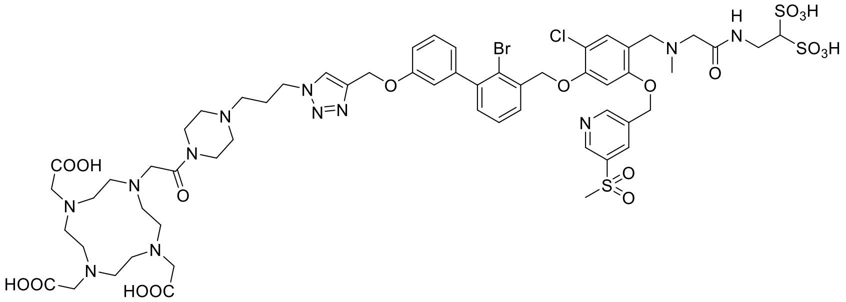

2,2',2''-(10-(2-(4-(3-(4-(((3'-((2-Chloro-4-(((2-((2,2-disulfoethyl)amino)-2-oxoethyl)(methyl)amino)methyl)-5-((5-(methylsulfonyl)pyridin-3-yl)methoxy)phenoxy)methyl)-2,2'-dimethyl-[1,1'-biphenyl]-3-yl)oxy)methyl)-1H-1,2,3-triazol-

1-yl)propyl)piperazin-1-yl)-2-oxoethyl)-1,4,7,10-tetraazacyclododecane-1,4,7-triyl)triacetic acid (**44a**). Propyl-piperazinyl linker structure **41b** (5.0 mg, 4.9 µmol, 1.00 equiv.), DOTA-*p*-nitrophenylester (**61**) (5.2 mg, 9.8 µmol, 2.00 equiv.) and abs. DIPEA (4.3 µL, 24.5 µmol, 5.00 equiv.) were dissolved in abs. DMF (1 mL) and reacted according to GP-7. Purification by semi-preparative RP-HPLC (System C, R_t = 18 min) and subsequent lyophilization yielded DOTA-conjugate **44a** (5.2 mg, 3.7 µmol, 75%) as a colorless powder. mp = 190–193 °C (decomposition). R_t = 8.91 min (System A), purity: 99.0%. IR (ATR): ṽ = 3418 (m), 3011 (w), 1667 (s), 1456 (m), 1385 (m), 1304 (w), 1199 (s), 1145 (s), 1016 (m), 720 (w), 594 cm^{-1} (w). HRMS (ESI, $[M+H]^+$) calcd for $C_{60}H_{82}ClN_{12}O_{19}O_3$, 1405.4665; found, 1405.4650.

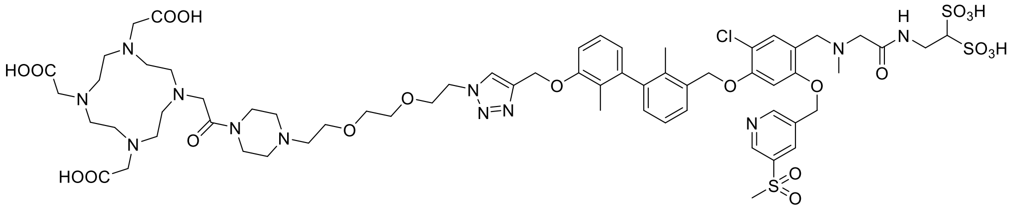

2,2',2''-(10-(2-(4-(2-(2-(2-(4-(((3'-((2-Chloro-4-(((2-((2,2-disulfoethyl)amino)-2-oxoethyl)(methyl)amino)methyl)-5-((5-(methylsulfonyl)pyridin-3-yl)methoxy)phenoxy)methyl)-2,2'-dimethyl-[1,1'-biphenyl]-3-yl)oxy)methyl)-1H-1,2,3-triazol-1-yl)ethoxy)ethoxy)ethyl)piperazin-1-yl)-2-oxoethyl)-1,4,7,10-tetraazacyclododecane-1,4,7-triyl)triacetic acid (**43c**). PEG-2-Piperazinyl linker structure **41c** (14.0 mg, 12.8 µmol, 1.00 equiv.), DOTA-*p*-nitrophenylester (**61**) (13.5 mg, 25.6 µmol, 2.00 equiv.) and abs. DIPEA (11.2 µL, 64.0 µmol, 5.00 equiv.) were dissolved in abs. DMF (1 mL) and reacted according to GP-7. Purification by semi-preparative RP-HPLC (System C, R_t = 16 min) and subsequent lyophilization yielded DOTA-conjugate **43c** (6.2 mg, 4.2 µmol, 33%) as a colorless powder. mp = 195 °C (decomposition). R_t = 9.09 min (System A), purity: 100%. IR (ATR): ṽ = 3419 (w), 1668 (m), 1461 (w), 1403 (w), 1303 (m), 1198 (s), 1145 (s), 1062 (w), 1018 (m), 720 (w), 594 cm^{-1} (w). HRMS (ESI, $[M+H]^+$) calcd for $C_{63}H_{87}ClN_{12}O_{21}S_3$, 1479.5032; found, 1479.5018.

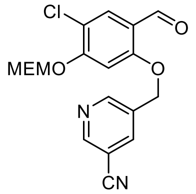

*5-((4-Chloro-2-formyl-5-((2-methoxyethoxy)methoxy)phenoxy)methyl)nicotinonitrile (**46a**)*. 5-Chloro-2-hydroxy-4-((2-methoxyethoxy)methoxy)benzaldehyde (**6**) (644 mg, 2.47 mmol, 1.00 equiv.) reacted with 5-(bromomethyl)nicotinonitrile (730 mg, 3.71 mmol, 1.50 equiv.) and potassium carbonate (683 mg, 4.94 mmol, 2.00 equiv.) as a base in abs. DMF (10 mL) according to GP-1. Purification by flash column chromatography on silica gel (EA, R_f = 0.3) lead to 5-((4-chloro-2-formyl-5-((2-methoxyethoxy)methoxy)phenoxy)methyl)nicotinonitrile (**46a**) (897 mg, 2.38 mmol, 96%) as a yellowish solid. mp = 97 °C. ^{1}H NMR (600 MHz, $CDCl_3$) δ = 10.29 (s, 1H), 8.91 (d, ^4J = 1.9 Hz, 1H), 8.90 (d, ^4J = 1.9 Hz, 1H), 8.11 (s, 1H), 7.87 (s, 1H), 6.98 (s, 1H), 5.42 (s, 2H), 5.23 (s, 2H), 3.88–3.89 (m, 2H), 3.57–3.58 (m, 2H), 3.37 ppm (s, 3H). ^{13}C NMR (151 MHz, $CDCl_3$) δ = 186.6, 159.9, 158.9, 152.4, 152.1, 138.2, 132.1, 130.6, 120.2, 117.5, 116.2, 110.5, 100.9, 94.3, 71.5, 68.5, 67.5, 59.1 ppm. IR (ATR): $\tilde{\nu}$ = 3059 (w), 2920 (w), 2870 (w), 2235 (w), 1676 (s), 1598 (m), 1563 (m), 1493 (m), 1444 (m), 1422 (m), 1373 (m), 1318 (w), 1293 (m), 1185 (s), 1112 (s), 1082 (s), 1054 (m), 1009 (s), 951 (s), 892 (m), 840 (s), 753 (w), 702 (s), 652 (m), 572 (w), 541 (m), 511 (w), 464 (w), 421 cm^{-1} (w). HRMS (ESI, $[M+H]^+$) calcd for $C_{18}H_{18}ClN_2O_5$, 377.0904; found, 377.0899.

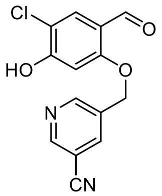

*5-((4-Chloro-2-formyl-5-hydroxyphenoxy)methyl)nicotinonitrile (**51b**)*. MEM-Protected phenol **46a** (870 mg, 2.31 mmol, 1.00 equiv.) was dissolved in abs. dichloromethane (12 mL) under argon atmosphere and the solution was cooled to 0 °C. After addition of trifluoroacetic acid (3 mL), the reaction mixture was stirred at room temperature for 16 h. Ethyl acetate (100 mL) was added to the solution and a solid precipitated, which was isolated by filtration. The pale yellowish precipitate turned out to be 5-((4-chloro-2-formyl-5-hydroxyphenoxy)methyl)nicotinonitrile (**51b**) (498 mg, 1.61 mmol, 70%), which was used in the next step without further purification. mp = 180 °C (decomposition). ^{1}H NMR (600 MHz, DMSO-d_6) δ = 11.58 (s, 1H), 10.16 (s, 1H), 9.03 (d, ^4J = 1.9 Hz, 1H), 9.01 (d, ^4J = 1.9 Hz, 1H), 8.51 (s, 1H), 7.66 (s, 1H), 6.77 (s, 1H), 5.35 ppm (s, 2H). ^{13}C NMR (151 MHz, DMSO-d_6) δ = 186.7, 160.2, 159.8, 152.4, 152.0, 138.9, 132.5, 129.4, 117.9, 116.8, 113.5, 109.1, 101.4, 66.8 ppm. IR (ATR): $\tilde{\nu}$ = 3012 (w), 2241 (w), 1655 (m), 1593 (m), 1568 (m), 1493 (m), 1452 (w), 1433 (m),

1389 (m), 1294 (s), 1198 (m), 1174 (m), 1133 (w), 1057 (m), 1022 (s), 947 (w), 887 (m), 843 (m), 718 (w), 698 (m), 682 (s), 636 (m), 597 (m), 552 (m), 466 (m), 417 cm^{-1} (w). HRMS (EI, $[M]^+$) calcd for C_{14}, 288.0302; found, 288.0286.

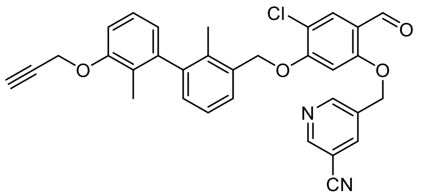

5-((4-Chloro-5-((2,2'-dimethyl-3'-(prop-2-yn-1-yloxy)-[1,1'-biphenyl]-3-yl)-methoxy)-2-formylphenoxy)methyl)nicotinonitrile (**52a**). (2,2'-Dimethyl-3'-(prop-2-yn-1-yloxy)-[1,1'-biphenyl]-3-yl)methanol (**28b**) (110 mg, 381 µmol, 1.00 equiv.), phenol **11** (112 mg, 419 µmol, 1.10 equiv.), triphenylphosphine (130 mg, 495 µmol, 1.30 equiv.) were dissolved in abs. DMF (1.5 mL) and reacted with DEAD (77.7 µL, 495 µmol, 1.30 equiv.) according to GP-2. Purification by flash column chromatography on silica gel (PE:EA, 6:4, R_f = 0.2) gave compound **52a** (149 mg, 278 µmol, 73%) as a colorless solid. mp = 157 °C. ^{1}H NMR (600 MHz, $CDCl_3$) δ = 10.27 (s, 1H), 8.91–8.92 (m, 2H), 8.10 (s, 1H), 7.92 (s, 1H), 7.45 (d, ^3J = 7.6 Hz, 1H), 7.28 (t, ^3J = 7.6 Hz, 1H), 7.22 (t, ^3J = 7.3 Hz, 1H), 7.17 (d, ^3J = 7.5 Hz, 1H), 7.00 (d, ^3J = 8.0 Hz, 1H), 6.80 (d, ^3J = 7.6 Hz, 1H), 6.65 (s, 1H), 5.25 (s, 2H), 5.22 (s, 2H), 4.77 (d, ^4J = 2.3 Hz, 2H), 2.55 (t, ^4J = 2.3 Hz, 1H), 2.09 (s, 3H), 1.93 ppm (s, 3H). ^{13}C NMR (151 MHz, $CDCl_3$) δ = 186.4, 160.1, 160.1, 156.0, 152.5, 151.9, 142.8, 142.6, 138.1, 134.8, 133.3, 132.0, 130.9, 130.3, 127.5, 126.2, 125.9, 125.6, 122.8, 119.5, 117.7, 116.2, 110.7, 110.5, 98.7, 79.1, 75.5, 70.7, 67.7, 56.3, 15.9, 13.1 ppm. IR (ATR): ṽ = 3277 (w), 2918 (w), 2234 (w), 1672 (m), 1597 (s), 1501 (m), 1448 (m), 1410 (m), 1382 (m), 1312 (m), 1271 (s), 1171 (s), 1144 (s), 1076 (m), 1016 (s), 924 (w), 873 (m), 809 (m), 787 (m), 722 (m), 690 (s), 640 (m), 545 (w), 503 (w), 460 cm^{-1} (w). HRMS (MALDI, $[M+H]^+$) calcd for $C_{32}H_{26}ClN_2O_4$, 537.1576; found, 537.1577.

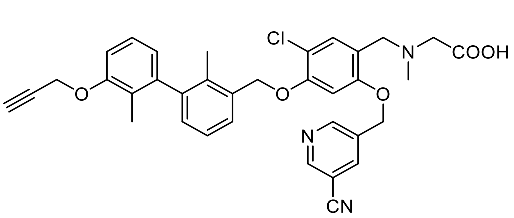

N-(5-Chloro-2-((5-cyanopyridin-3-yl)methoxy)-4-((2,2'-dimethyl-3'-(prop-2-yn-1-yloxy)-[1,1'-biphenyl]-3-yl)methoxy)benzyl)-N-methylglycine (**53a**). Aldehyde **52a** (160 mg, 298 mmol, 1.00 equiv.), sarcosine (133 mg, 1.49 mmol, 5.00 equiv.) and sodium cyanoborohydride

(28.1 mg, 447 µmol, 1.50 equiv.) were reacted in an 1:1 mixture of abs. DMF/MeOH (4 mL) and subsequently worked up according to GP-3. After flash column chromatography on silica gel (MeCN:MeOH:Et_3N, 100:20:1, R_f = 0.2) compound **53a** (107 mg, 175 µmol, 59%) was obtained as a colorless solid. mp = 91 °C. R_t = 12.46 min (System A), purity: 98.6%. ^{1}H NMR (400 MHz, methanol-d_4) δ = 8.97 (s, 1H), 8.92 (s, 1H), 8.41 (s, 1H), 7.57 (s, 1H), 7.46 (d, ^3J = 7.6 Hz, 1H), 7.26–7.18 (m, 2H), 7.03–7.10 (m, 3H), 6.74 (d, ^3J = 7.5 Hz, 1H), 5.38 (s, 2H), 5.30 (s, 2H), 4.76 (s, 2H), 4.34 (s, 2H), 3.57 (s, 2H), 2.94 (t, ^4J = 2.3 Hz, 1H), 2.78 (s, 3H), 2.08 (s, 3H), 1.88 ppm (s, 3H). ^{13}C NMR (101 MHz, methanol-d_4) δ = 170.0, 158.1, 157.9, 157.3, 153.4, 153.1, 144.2, 143.7, 140.7, 137.0, 136.2, 135.7, 135.3, 135.2, 134.7, 134.4, 131.7, 131.5, 130.8, 129.0, 127.1, 126.5, 126.3, 123.5, 117.3, 116.6, 113.0, 112.0, 111.6, 101.1, 80.1, 76.5, 71.5, 68.7, 59.2, 57.0, 55.2, 41.8, 15.8, 13.1 ppm. IR (ATR): ṽ = 1626 (m), 1607 (s), 1575 (s), 1505 (m), 1455 (m), 1380 (s), 1303 (s), 1256 (m), 1166 (s), 1145 (m), 1088 (w), 1015 (m), 897 (w), 785 (w), 721 (w), 699 cm^{-1} (w). HRMS (ESI, $[M+H]^+$) calcd for $C_{35}H_{33}ClN_3O_5$, 610.2103; found, 610.2105.

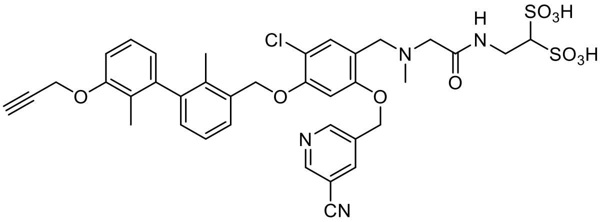

2-(2-((5-Chloro-2-((5-cyanopyridin-3-yl)methoxy)-4-((2,2'-dimethyl-3'-(prop-2-yn-1-yloxy)-[1,1'-biphenyl]-3-yl)methoxy)benzyl)(methyl)amino)acetamido)ethane-1,1-disulfonic acid (**54a**). Carboxylic acid **53a** (100 mg, 164 µmol, 1.00 equiv.) reacted with 2-aminoethane-1,1-disulfonic acid (TBA salt) (**32**) (320 mg, 820 µmol, 5.00 equiv.) in presence of DIPEA (57.3 µL, 328 µmol, 2.00 equiv.) and HTBU (137 mg, 361 µmol, 2.20 equiv.) in abs. DMF (3 mL) according to GP-6. Purification was performed by semi-preparative RP-HPLC (System B, R_t = 9 min) and after cation exchange and lyophilization, compound **54a** (106 mg, 133 µmol, 81%) was obtained as a colorless solid. mp = 170 °C (decomposition). R_t = 10.92 min (System A), purity: 96.8%. ^{1}H NMR (400 MHz, DMSO-d_6) δ = 9.56 (br, 1H), 9.07 (s, 2H), 8.55 (s, 1H), 8.14 (br, 1H), 7.63 (s, 1H), 7.52 (d, ^3J = 7.7 Hz, 1H), 7.22–7.28 (m, 2H), 7.05–7.10 (m, 2H), 6.75 (d, ^3J = 7.5 Hz, 1H), 5.34–5.43 (m, 4H), 4.86 (s, 2H), 4.27–4.36 (m), 3.87–3.91 (m, 2H), 3.70–3.71 (m, 2H), 3.54–3.59 (m, 2H), 2.71 (s, 3H), 2.04 (s, 3H), 1.83 ppm (s, 3H). ^{13}C NMR (101 MHz, DMSO-d_6) δ = 163.4, 156.8, 155.7, 155.4, 152.2, 151.6, 142.3, 141.5, 139.6, 134.6, 134.5, 133.9, 132.8,

129.3, 127.9, 126.2, 125.5, 124.1, 122.1, 116.7, 113.4, 111.1, 110.9, 109.2, 100.2, 79.5, 78.2, 74.0, 69.7, 67.2, 56.2, 55.8, 52.9, 40.7, 15.3, 12.8 ppm. IR (ATR): ṽ = 1679 (m), 1606 (w), 1575 (w), 1456 (w), 1305 (m), 1235 (m), 1199 (s), 1178 (s), 1145 (s), 1063 (w), 1017 (m), 721 (w), 593 cm^{-1} (w). HRMS (ESI, $[M+H]^+$) calcd for $C_{37}H_{38}ClN_4O_{10}S_2$, 797.1713; found, 797.1712.

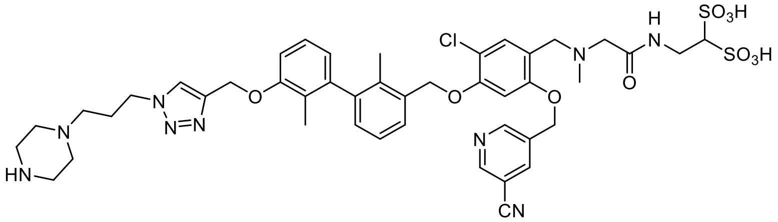

2-(2-((5-Chloro-2-((5-cyanopyridin-3-yl)methoxy)-4-((2,2'-dimethyl-3'-((1-(3-(piperazin-1-yl)propyl)-1H-1,2,3-triazol-4-yl)methoxy)-[1,1'-biphenyl]-3-yl)methoxy)benzyl)(methyl)amino)acetamido)ethane-1,1-disulfonic acid (**55a**). Alkyne **54a** (25.0 mg, 31.4 µmol, 1.00 equiv.) and 1-(3-azidopropyl)piperazine (**37**) (10.6 mg, 62.7 µmol, 2.00 equiv.) were dissolved in DMF (1 mL), reacted in presence of TBTA (0.2 mg, 0.3 µmol, 0.01 equiv.) and $[Cu(MeCN)_4]PF_6$ (5.8 mg, 15.7 µmol, 0.50 equiv.) according to GP-4. Purification by semi-preparative RP-HPLC (System C, R_t = 18 min) yielded compound **55a** (14.0 mg, 14.5 µmol, 46%) as a colorless powder. mp = 150 °C (decomposition). R_t = 9.26 min (System A), purity: 95.2%. ^1H NMR (400 MHz, DMSO-d_6) δ = 9.62 (br, 2H), 9.02 (dd, ^4J = 1.8, ^3J = 11.0 Hz, 2H), 8.48 (s, 1H), 8.27 (s, 1H), 8.15 (br, 1H), 7.58 (s, 1H), 7.50 (d, ^3J = 7.3 Hz, 1H), 7.21–7.27 (m, 2H), 7.14–7.16 (m, 2H), 7.07 (d, ^3J = 7.4 Hz, 1H), 6.72 (d, ^3J = 7.3 Hz, 1H), 5.30–5.39 (m, 4H), 5.16–5.23 (m, 2H), 4.46 (t, ^3J = 6.9 Hz, 2H), 1.92 (br, 2H), 3.78–3.41 (m), 2.70 (s, 3H), 2.24 (br, 2H), 1.99 (s, 3H), 1.71 ppm (br, 3H). ^{13}C NMR (101 MHz, DMSO-d_6) δ = 163.6, 156.6, 156.1, 152.5, 152.1, 146.6, 143.2, 142.2, 141.7, 139.1, 135.1, 134.7, 133.8, 132.6, 129.3, 126.4, 125.4, 124.4, 124.3, 121.6, 116.9, 113.6, 111.0, 109.1, 100.5, 73.9, 70.0, 67.2, 61.9, 53.7, 52.8, 48.5, 46.9, 41.2, 15.5, 12.7 ppm. IR (ATR): ṽ = 1679 (m), 1606 (w), 1575 (w), 1456 (w), 1305 (m), 1199 (s), 1178 (s), 1145 (s), 1063 (w), 1017 (m), 721 (w), 594 cm^{-1} (w). HRMS (ESI, $[M+H]^+$) calcd for $C_{44}H_{53}ClN_9O_{10}S_2$, 966.3040, found, 966.3045.

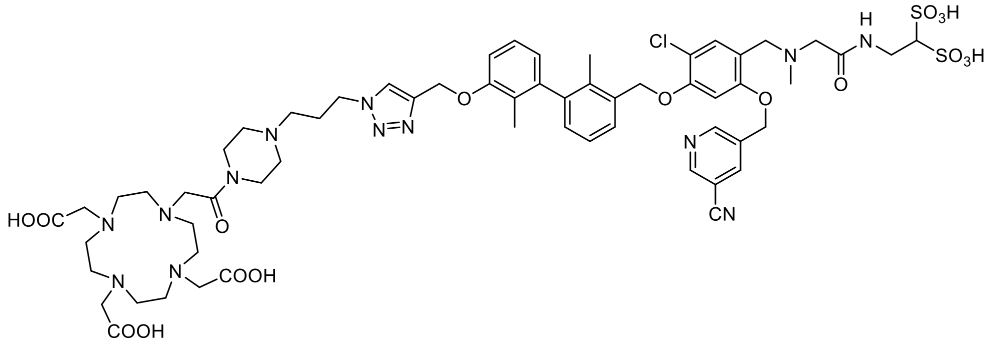

2,2',2''-(10-(2-(4-(3-(4-(((3'-((2-Chloro-5-((5-cyanopyridin-3-yl)methoxy)-4-(((2-((2,2-disulfoethyl)amino)-2-oxoethyl)(methyl)amino)methyl)phenoxy)methyl)-2,2'-dimethyl-[1,1'-biphenyl]-3-yl)oxy)methyl)-1H-1,2,3-triazol-1-yl)propyl)piperazin-1-yl)-2-oxoethyl)-1,4,7,10-tetraazacyclododecane-1,4,7-triyl)triacetic acid (**56a**). Compound **55a** (7.0 mg, 7.2 µmol, 1.00 equiv.), DOTA-*p*-nitrophenylester (**61**) (5.7 mg, 10.9 µmol, 1.50 equiv.) and abs. DIPEA (6.3 µL, 36.2 µmol, 5.00 equiv.) were dissolved in abs. DMF (1 mL) and reacted according to GP-7. Purification by semi-preparative RP-HPLC (System C, R_t = 19 min) and subsequent lyophilization yielded DOTA-conjugate **56a** (6.0 mg, 4.4 µmol, 61%) as a colorless powder. mp = 190 °C (decomposition). R_t = 9.42 min (System A), purity: 96.6%. IR (ATR): ṽ = 3418 (m), 1728 (w), 1668 (s), 1456 (w), 1386 (w), 1198 (s), 1089 (w), 1062 (w), 1015 (m), 720 (w), 594 cm^{-1} (w). HRMS (ESI, $[M+H]^+$) calcd for $C_{60}H_{79}ClN_{13}O_{17}S_2$, 1352.4842; found, 1352.4831.

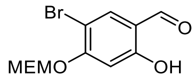

*5-Bromo-2-hydroxy-4-((2-methoxyethoxy)methoxy)benzaldehyde (**45**).* 5-Bromo-2,4-dihydroxybenzaldehyde (**6**) (5.50 g, 25.3 mmol, 1.00 equiv.) was dissolved in abs. THF (100 mL) under argon and abs. DIPEA (8.83 mL, 50.7 mmol, 2.00 equiv.) was added in one portion. After cooling to 0 °C, MEM chloride (4.34 mL, 38.0 mmol, 1.50 equiv.) was added *via* syringe in small portions and the reaction mixture was stirred at room temperature for 16 h under argon. Water (100 mL) was added and the phases were separated. The aqueous phase was extracted with DCM (3 x 100 mL) and the combined organic extracts were dried over sodium sulfate, filtered and concentrated under reduced pressure. The residue was purified by flash column chromatography on silica gel (PE:EA, 7:3, R_f = 0.25) to afford 5-bromo-2-hydroxy-4-((2-methoxyethoxy)methoxy)benzaldehyde (**45**) (3.78 g, 12.4 mmol, 60%) as a yellowish oil. mp = 56 °C. ^{1}H NMR (400 MHz, $CDCl_3$) δ = 11.27 (s, 1H), 9.69 (s, 1H), 7.67 (s, 1H), 6.76 (s, 1H), 5.38 (s, 2H), 3.84–3.86 (m, 2H), 3.57–3.57 ppm (m, 3H). ^{13}C NMR (101 MHz, $CDCl_3$)

δ = 193.9, 163.3, 160.4, 137.5, 116.7, 103.6, 102.8, 94.0, 71.5, 68.8, 59.2 ppm. IR (ATR): $\tilde{\nu}$ = 2906 (m), 2852 (m), 1641 (m), 1619 (s), 1563 (w), 1486 (m), 1454 (w), 1351 (m), 1318 (m), 1270 (m), 1194 (s), 1154 (s), 1103 (s), 1028 (w), 1012 (s), 938 (s), 844 (m), 826 (m), 730 (w), 693 (m), 674 (m), 559 cm^{-1} (w). HRMS (ESI, $[M+Na]^+$) calcd for $C_{11}H_{13}BrO_5Na$, 326.9844; found, 326.9838.

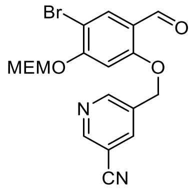

*5-((4-Bromo-2-formyl-5-((2-methoxyethoxy)methoxy)phenoxy)methyl)nicotinonitrile (**46b**)*. 5-Bromo-2-hydroxy-4-((2-methoxyethoxy)methoxy)benzaldehyde (**45**) (1.28 g, 4.20 mmol, 1.00 equiv.) reacted with 5-(bromomethyl)nicotinonitrile (992 mg, 5.03 mmol, 1.20 equiv.) and potassium carbonate (1.16 g, 8.39 mmol, 2.00 equiv.) as a base in abs. DMF (20 mL) according to GP-1. Purification by flash column chromatography on silica gel (EE, R_f = 0.3) lead to 5-((4-bromo-2-formyl-5-((2-methoxyethoxy)methoxy)phenoxy)methyl)nicotinonitrile (**46b**) (1.53 g, 3.63 mmol, 87%) as a yellowish solid. mp = 110 °C. ^1H NMR (400 MHz, $CDCl_3$) δ = 10.21 (s, 1H), 8.89 (d, ^4J = 1.9 Hz, 1H), 8.85 (d, ^4J = 1.6 Hz, 1H), 8.08-8.09 (s, 1H), 7.94 (d, ^4J = 1.0 Hz, 1H), 6.91 (s, 1H), 5.38 (s, 2H), 5.20 (s, 2H), 3.83–3.86 (m, 2H), 3.53–3.55 (m, 2H), 3.33 ppm (s, 3H) ^{13}C NMR (101 MHz, $CDCl_3$) δ = 186.4, 160.4, 159.7, 152.3, 151.9, 138.2, 133.5, 132.0, 120.5, 116.2, 110.2, 105.4, 100.6, 94.2, 71.3, 68.4, 67.3, 59.0 ppm. IR (ATR): $\tilde{\nu}$ = 3059 (w), 2919 (m), 2235 (w), 1691 (m), 1673 (m), 1593 (s), 1557 (m), 1492 (w), 1380 (m), 1371 (s), 1282 (s), 1184 (s), 1117 (m), 1080 (m), 1048 (w), 997 (s), 947 (m), 892 (m), 838 (m), 702 (w), 686 (w), 649 cm^{-1} (w). HRMS (ESI, $[M+Na]^+$) calcd for $C_{18}H_{17}BrN_2O_5Na$, 443.0219; found, 443.0215.

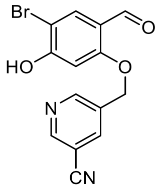

*5-((4-Bromo-2-formyl-5-hydroxyphenoxy)methyl)nicotinonitrile (**51b**)*. MEM-Protected phenol **46b** (760 mg, 1.80 mmol, 1.00 equiv.) was dissolved in abs. dichloromethane (10 mL) under argon atmosphere and the solution was cooled to 0 °C. After addition of trifluoroacetic acid

(5 mL), the reaction mixture was stirred at room temperature for 16 h. Ethyl acetate (100 mL) was added to the solution and a solid precipitated, which was isolated by filtration. The pale yellowish precipitate turned out to be 5-((4-bromo-2-formyl-5-hydroxyphenoxy)methyl)nicotinonitrile (**51b**) (495 mg, 1.49 mmol, 82%), which was used in the next step without further purification. mp = 200 °C (decomposition). ^{1}H NMR (400 MHz, DMSO-d$_6$) δ = 11.62 (s, 1H), 10.14 (s, 1H), 9.02 (s, 2H), 8.51 (s, 1H), 7.80 (s, 1H), 6.75 (s, 1H), 5.32 ppm (s, 2H). ^{13}C NMR (101 MHz, DMSO-d$_6$) δ = 186.6, 160.8, 160.8, 152.3, 152.0, 138.9, 132.6, 132.5, 118.5, 116.8, 109.0, 102.1, 101.1, 66.7 ppm. IR (ATR): $\tilde{\nu}$ = 3258 (w), 3018 (w), 2239 (w), 1655 (s), 1590 (s), 1566 (s), 1448 (m), 1432 (m), 1388 (m), 1292 (s), 1195 (m), 1133 (s), 1054 (w), 1011 (w), 886 (w), 698 (w), 671 cm^{-1}. (m). HRMS (ESI, [M+Na]$^+$) calcd for $C_{14}H_9BrN_2O_3Na$, 331.9897; found, 331.9782.

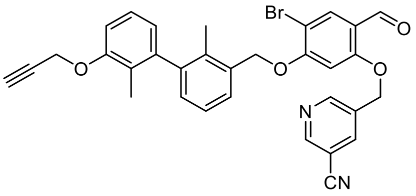

5-((4-Bromo-5-((2,2'-dimethyl-3'-(prop-2-yn-1-yloxy)-[1,1'-biphenyl]-3-yl)-methoxy)-2-formylphenoxy)methyl)nicotinonitrile (**52b**). (2,2'-Dimethyl-3'-(prop-2-yn-1-yloxy)-[1,1'-biphenyl]-3-yl)methanol (**28b**) (150 mg, 563 µmol, 1.00 equiv.), phenol **11** (216 mg, 648 µmol, 1.15 equiv.) and triphenylphosphine (192 mg, 732 µmol, 1.30 equiv.) were dissolved in abs. DMF (3 mL) and reacted with DMEAD (172 mg, 732 µmol, 1.30 equiv.) according to GP-2. Purification by flash column chromatography on silica gel (DCM:EA, 95:5, R_f = 0.2) gave title compound **52b** (185 mg, 318 µmol, 57%) as a colorless solid. mp = 95 °C (decomposition). ^{1}H NMR (400 MHz, CDCl$_3$) δ = 10.25 (s, 1H), 9.02 (br, 2H), 8.09–8.11 (m, 2H), 7.46 (d, ^{3}J = 7.5 Hz, 1H), 7.16–7.28 (m, 3H), 6.99 (d, ^{3}J = 8.2 Hz, 1H), 6.79 (d, ^{3}J = 7.6 Hz, 1H), 6.62 (s, 1H), 5.23–5.25 (m, 4H), 4.77 (s, 2H), 2.54 (s, 1H), 2.09 (s, 3H), 1.93 ppm (s, 3H). ^{13}C NMR (101 MHz, DMSO-d$_6$) δ = 186.3, 161.0, 160.8, 156.0, 152.5, 151.8, 142.8, 142.6, 138.1, 134.7, 134.2, 133.3, 130.3, 127.5, 126.2, 125.9, 125.6, 122.8, 120.1, 116.2, 110.7, 110.2, 105.8, 98.5, 79.1, 75.5, 70.7, 67.7, 56.3, 15.9, 13.1 ppm. IR (ATR): ṽ = 2235 (w), 1669 (m), 1589 (s), 1455 (w), 1409 (m), 1381 (w), 1314 (w), 1271 (s), 1165 (m), 1145 (m), 1086 (w), 1015 cm^{-1} (m). HRMS (ESI, [M+Na]$^+$) calcd for $C_{32}H_{25}BrN_2O_4Na$, 603.0895; found, 603.0896.

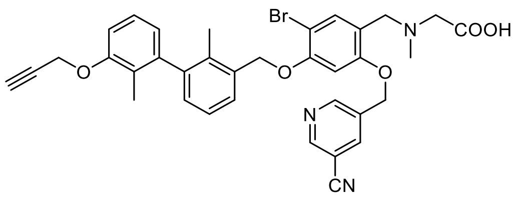

N-(5-Bromo-2-((5-cyanopyridin-3-yl)methoxy)-4-((2,2'-dimethyl-3'-(prop-2-yn-1-yloxy)-[1,1'-biphenyl]-3-yl)methoxy)benzyl)-N-methylglycine (**53b**). Aldeyhde **52b** (180 mg, 310 mmol, 1.00 equiv.), sarcosine (138 mg, 1.55 mmol, 5.00 equiv.) and sodium cyanoborohydride (29.2 mg, 464 µmol, 1.50 equiv.) were reacted in an 1:1 mixture of abs. DMF/MeOH (4 mL) and subsequently worked up according to GP-3. After flash column chromatography on silica gel (MeCN:MeOH:Et_3N, 100:20:1, R_f = 0.2) compound **53b** (131 mg, 200 µmol, 65%) was obtained as a colorless solid. mp = 97–99 °C. R_t = 12.52 min (System A), purity: 96.2%. ^1H NMR (400 MHz, methanol-d$_4$) δ = 8.97 (s, 1H), 8.91 (s, 1H), 8.41 (s, 1H), 7.73 (s, 1H), 7.47 (d, ^3J = 7.6 Hz, 1H), 7.18–7.26 (m, 2H), 7.09 (d, ^3J = 7.6 Hz, 2H), 7.02–7.04 (m, 2H), 6.74 (d, ^3J = 7.5 Hz, 1H), 5.38 (s, 2H), 5.29 (s, 2H), 4.79 (d, ^4J = 1.8 Hz, 2H), 4.36 (s, 2H), 3.59 (s, 2H), 2.94 (t, ^4J = 1.8 Hz, 1H), 2.79 (s, 3H), 2.08 (s, 3H), 1.88 ppm (s, 3H). ^{13}C NMR (101 MHz, methanol-d$_4$) δ = 169.8, 159.0, 158.8, 157.3, 153.4, 153.1, 144.2, 143.6, 140.7, 137.8, 136.2, 135.7, 134.4, 130.8, 128.9, 127.1, 126.5, 126.3, 123.5, 117.3, 133.4, 122.0, 111.6, 104.7, 100.8, 80.1, 76.5, 71.5, 68.7, 59.1, 57.0, 55.2, 41.8, 15.9, 13.2 ppm. IR (ATR): ṽ = 1626 (m), 1061 (s), 1503 (w), 1455 (m), 1380 (m), 1298 (m), 1255 (m), 1161 (s), 1145 (s), 1014 (m), 699 cm^{-1} (w). HRMS (ESI, $[M+H]^+$) calcd for $C_{35}H_{33}BrN_3O_5$, 654.1598; found, 654.1610.

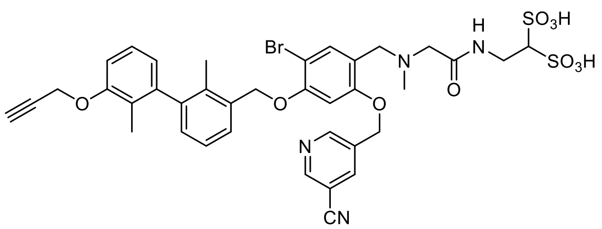

2-(2-((5-Bromo-2-((5-cyanopyridin-3-yl)methoxy)-4-((2,2'-dimethyl-3'-(prop-2-yn-1-yloxy)-[1,1'-biphenyl]-3-yl)methoxy)benzyl)(methyl)amino)acetamido)ethane-1,1-disulfonic acid (**54b**). Carboxylic acid **53b** (120 mg, 209 µmol, 1.00 equiv.) reacted with 2-aminoethane-1,1-disulfonic acid (TBA salt) (**32**) (408 mg, 1.04 mol, 5.00 equiv.) in presence of DIPEA (72.8 µL, 418 µmol, 2.00 equiv.) and HTBU (174 mg, 459 µmol, 2.20 equiv.) in abs. DMF (3 mL) according to GP-6. Purification was performed by semi-preparative RP-HPLC (System B, R_t = 10 min) and after cation exchange and lyophilization, compound **54b** (110 mg, 131 µmol,

63%) was obtained as a colorless solid. mp = 170 °C (decomposition). R_t = 11.06 min (System A), purity: 99.8%. ^{1}H NMR (400 MHz, DMSO-d_6) δ = 9.56 (br, 1H), 9.07 (s, 2H), 8.55 (s, 1H), 8.15 (br, 1H), 7.76 (s, 1H), 7.54 (d, ^3J = 7.4 Hz, 1H), 7.28–7.24 (m, 2H), 7.18 (s, 1H), 7.04–7.10 (m, 2H), 6.75 (d, ^3J = 7.5 Hz, 1H), 5.33–5.47 (m, 4H), 4.82–4.85 (m), 4.26–4.35 (m, 2H), 3.88–3.91 (m, 2H), 3.69–3.72 (m, 2H), 3.54–3.59 (m, 2H), 2.70 (s, 3H), 2.05 (s, 3H), 1.84 ppm (s, 3H). ^{13}C NMR (101 MHz, DMSO-d_6) δ = 163.4, 157.5, 156.6, 155.4, 152.1, 151.6, 142.3, 141.5, 139.6, 136.8, 134.6, 134.5, 132.9, 129.3, 127.8, 126.2, 125.5, 124.1, 122.1, 116.7, 111.8, 110.9, 109.2, 102.1, 100.0, 76.5, 78.2, 74.0, 69.8, 67.1, 56.2, 55.8, 52.9, 40.7, 15.4, 12.8 ppm. IR (ATR): ṽ = 3345 (w), 1677 (m), 1602 (w), 1574 (w), 1437 (w), 1405 (w), 1255 (m), 1205 (s), 1170 (s), 1061 (w), 1013 (m), 755 (w), 585 cm^{-1} (w). HRMS (ESI, $[M+H]^+$) calcd for $C_{37}H_{38}BrN_4O_{10}S_2$, 841.1207; found, 841.1209.

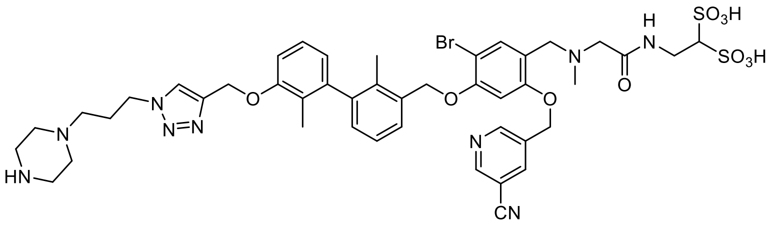

2-(2-((5-Bromo-2-((5-cyanopyridin-3-yl)methoxy)-4-((2,2'-dimethyl-3'-((1-(3-(piperazin-1-yl)propyl)-1H-1,2,3-triazol-4-yl)methoxy)-[1,1'-biphenyl]-3-yl)methoxy)benzyl)(methyl)amino)acetamido)ethane-1,1-disulfonic acid (**55b**). Alkyne **54b** (25.0 mg, 29.7 µmol, 1.00 equiv.) reacted with 1-(3-azidopropyl)piperazine (7.61 mg, 45.0 µmol, 2.00 equiv.) with a premixed catalyst consisting of $CuSO_4$ (1.42 mg, 8.91 µmol, 0.30 equiv.), THPTA (1.68 mg, 3.86 µmol, 0.13 equiv.) and sodium ascorbate (29.4 mg, 149 µmol, 5.00 equiv.) in an 1:1 mixture of H_2O/t-BuOH (2 mL) at room temperature for 5 h according to GP-5. Purification by semi-preparative RP-HPLC (System C, R_t = 21 min) and subsequent lyophilization yielded compound **55b** (19.0 mg, 18.8 µmol, 63%) as a colorless solid. mp = 200 °C (decomposition). R_t = 9.37 min (System A), purity: 97.9%. ^{1}H NMR (400 MHz, DMSO-d_6) δ = 9.81 (br, 2H), 9.26 (s, 1H), 9.23 (s, 1H), 8.70 (s, 1H), 8.49 (s, 1H), 8.37 (br, 1H), 7.94 (s, 1H), 7.73 (br, 1H), 7.43–7.50 (m, 2H), 7.28–7.39 (m, 3H), 6.95 (d, ^3J = 7.3 Hz, 1H), 5.51–5.63 (m, 4H), 5.39–5.46 (m, 2H), 4.69 (t, ^3J = 6.9 Hz, 2H), 4.47 (br), 3.68–3.99 (m), 3.22 (br), 2.93 (s, 3H), 2.73 (s, 3H), 2.22 (s, 3H), 1.90–1.95 ppm (m, 2H). ^{13}C NMR (101 MHz, DMSO-d_6) δ = 163.6, 156.0, 152.4, 152.0, 143.3, 142.2, 141.8, 139.0, 136.7, 135.1, 134.4, 132.5, 129.2, 126.4, 125.4, 124.3, 121.6, 116.9, 111.1, 109.6, 109.1, 102.3, 73.9, 67.1, 61.9,

53.7, 48.4, 46.8, 41.3, 24.1, 15.6, 12.8 ppm. IR (ATR): ṽ = 3445 (w), 3019 (w), 1682 (m), 1606 (w), 1575 (w), 1456 (w), 1305 (m), 1236 (m), 1194 (s), 1179 (s), 1145 (s), 1063 (w), 1018 (m), 721 (w), 594 cm^{-1} (w). HRMS (ESI, $[M+H]^+$) calcd for $C_{44}H_{53}BrN_9O_{10}S_2$, 1010.2540; found, 1010.2533.

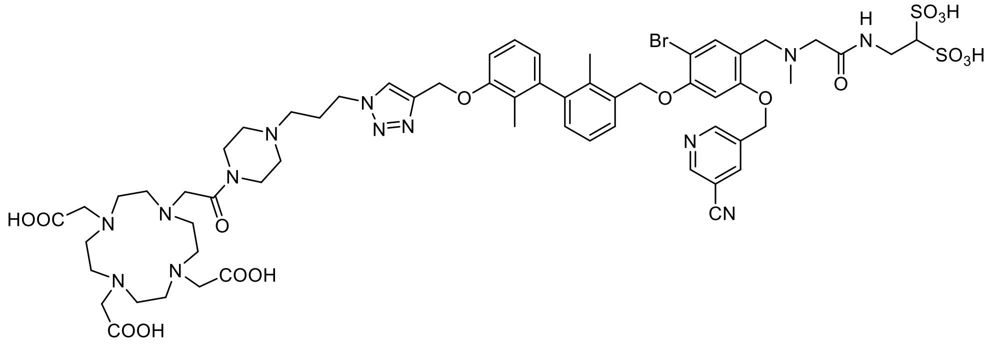

2,2',2''-(10-(2-(4-(3-(4-(((3'-((2-Bromo-5-((5-cyanopyridin-3-yl)methoxy)-4-(((2-((2,2-disulfoethyl)amino)-2-oxoethyl)(methyl)amino)methyl)phenoxy)methyl)-2,2'-dimethyl-[1,1'-biphenyl]-3-yl)oxy)methyl)-1H-1,2,3-triazol-1-yl)propyl)piperazin-1-yl)-2-oxoethyl)-1,4,7,10-tetraazacyclododecane-1,4,7-triyl)triacetic acid (**56b**). Propyl-piperazinyl linker structure **55b** (9.2 mg, 9.1 µmol, 1.00 equiv.), DOTA-*p*-nitrophenylester (**61**) (7.2 mg, 13.7 µmol, 1.50 equiv.) and abs. DIPEA (8.0 µL, 45.5 µmol, 5.00 equiv.) were dissolved in abs. DMF (1 mL) and reacted according to GP-7. Purification by semi-preparative RP-HPLC (System C, R_t = 21 min) and subsequent lyophilization yielded DOTA-conjugate **56b** (6.3 mg, 4.5 µmol, 50%) as a colorless powder. mp = 190 °C (decomposition). R_t = 9.88 min (System A), purity: 100%. IR (ATR): ṽ = 3418 (w), 1673 (s), 1455 (w), 1402 (m), 1199 (s), 1129 (m), 1025 (m), 800 (w), 720 (w), 597 cm^{-1} (w). HRMS (ESI, $[M+H]^+$) calcd for $C_{60}H_{79}Br_{13}O_{17}S_2$, 1396.4336; found, 1396.4327.

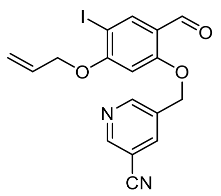

*5-((5-(Allyloxy)-2-formyl-4-iodophenoxy)methyl)nicotinonitrile (**50**)*. 4-(Allyloxy)-2-hydroxy-5-iodobenzaldehyde (**49**) (1.28 g, 4.21 mmol, 1.00 equiv.) reacted with 5-(bromomethyl)nicotinonitrile (995 mg, 5.05 mmol, 1.20 equiv.) and potassium carbonate (3.54 g, 8.42 mmol, 2.00 equiv.) as a base in abs. DMF (5 mL) according to GP-1. Purification by flash column chromatography on silica gel (DCM:EtOH, 90:10, R_f = 0.3) lead to 5-((5-

(allyloxy)-2-formyl-4-iodophenoxy)methyl)nicotinonitrile (**50**) (1.30 g, 3.09 mmol, 74%) as a beige solid. mp = 153 °C. ^{1}H NMR (400 MHz, $CDCl_3$) δ = 10.02 (s, 1H), 8.90–8.92 (m, 2H), 8.25 (s, 1H), 8.10 (s, 1H), 6.42 (s, 1H), 6.00–6.05 (m, 1H), 5.49–5.54 (m, 1H), 5.36–5.39 (m, 1H), 5.22 (s, 2H), 4.68–4.69 ppm (m, 2H). ^{13}C NMR (101 MHz, $CDCl_3$) δ = 186.3, 162.9, 161.8, 152.4, 151.8, 140.3, 138.2, 132.0, 131.5, 120.8, 118.7, 116.2, 110.5, 97.3, 77.8, 70.3, 67.4 ppm. IR (ATR): $\tilde{\nu}$ = 3079 (m), 2233 (m), 1673 (s), 1582 (s), 1567 (s), 1492 (w), 1446 (w), 1412 (m), 1404 (m), 1382 (m), 1312 (m), 1275 (s), 1248 (m), 1201 (m), 1151 (m), 1104 (w), 1052 (w), 1023 (m), 913 (m), 883 (w), 807 (m), 726 (w), 696 (m), 680 cm^{-1} (m). HRMS (ESI, $[M+H]^+$) calcd for $C_{17}H_{14}IN_2O_3$, 421.0044; found, 421.0049.

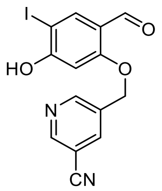

*5-((2-Formyl-5-hydroxy-4-iodophenoxy)methyl)nicotinonitrile (**51c**)*. 5-((5-(Allyloxy)-2-formyl-4-iodophenoxy)methyl)nicotinonitrile (**50**) (1.21 g, 2.87 mmol, 1.00 equiv.) was suspended in MeOH (50 mL) and this solution was degassed with argon for 30 min. Tetrakis(triphenylphosphine)palladium(0) (33.2 mg, 28.7 µmol, 0.01 equiv.) and potassium carbonate (1.19 g, 8.62 mmol, 3.00 equiv.) were added to initialize the reaction, which was stirred under argon at room temperature for 3 h. The solvent was removed under reduced pressure, ethyl acetate (100 mL) and water (100 mL) were added, the phases were separated and the aqueous phase was extracted with ethyl acetate (3 x 50 mL). The combined organic fractions were dried over sodium sulfate, filtered and the solvent was removed *in vacuo*. The crude product was purified on silica gel (EA, R_f = 0.3) to yield 5-((2-formyl-5-hydroxy-4-iodophenoxy)methyl)nicotinonitrile (**51c**) (360 mg, 947 µmol, 33%) as a brownish solid. mp = 160 °C (decomposition). ^{1}H NMR (400 MHz, DMSO-d_6) δ = 11.64 (s, 1H), 10.10 (s, 1H), 9.01–9.02 (m, 2H), 8.51 (s, 1H), 7.98-7.99 (s, 1H), 6.69 (s, 1H), 5.31 ppm (s, 1H). ^{13}C NMR (101 MHz, DMSO-d_6) δ = 186.4, 163.3, 161.7, 152.3, 152.0, 138.9, 138.8, 132.5, 119.3, 116.8, 109.0, 99.9, 75.8, 66.6 ppm. IR (ATR): $\tilde{\nu}$ = 3021 (w), 2235 (m), 1656 (s), 1584 (s), 1562 (s), 1482 (m), 1428 (m), 1411 (m), 1385 (m), 1312 (w), 1285 (s), 1200 (m), 1128 (s), 1051 (m), 1024 (m), 1002 (m), 936 (w), 885 (m), 838 (m), 698 (m), 665 cm^{-1} (m). HRMS (ESI, $[M+H]^+$) calcd for $C_{14}H_{10}IN_2O_3$, 380.9736; found, 380.9732.

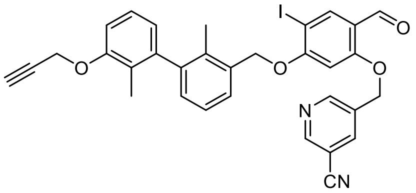

5-((5-((2,2'-Dimethyl-3'-(prop-2-yn-1-yloxy)-[1,1'-biphenyl]-3-yl)methoxy)-2-formyl-4-iodophenoxy)methyl)nicotinonitrile (***52c***). (2,2'-Dimethyl-3'-(prop-2-yn-1-yloxy)-[1,1'-biphenyl]-3-yl)methanol (**28b**) (135 mg, 355 µmol, 1.00 equiv.), phenol **11** (109 mg, 408 µmol, 1.15 equiv.) and triphenylphosphine (121 mg, 462 µmol, 1.30 equiv.) were dissolved in abs. DMF (3 mL) and reacted with DMEAD (108 mg, 462 µmol, 1.30 equiv.) according to GP-2. Purification by flash column chromatography on silica gel (DCM:EA, 95:5, R_f = 0.2) gave title compound **52c** (126 mg, 201 µmol, 57%) as a colorless solid. mp = 142 °C. ^{1}H NMR (400 MHz, $CDCl_3$) δ = 10.21 (s, 1H), 8.91 (s, 2H), 8.29 (s, 1H), 8.12 (s, 1H), 7.48 (d, ^3J = 7.6 Hz, 1H), 7.00–7.27 (m, 3H), 6.99 (d, ^3J = 8.2 Hz, 1H), 6.79 (d, ^3J = 7.6 Hz, 1H), 6.55 (s, 1H), 5.23 (s, 4H), 4.76–4.77 (m, 2H), 2.53–2.55 (m, 1H), 2.09 (s, 3H), 1.94 ppm (s, 3H). ^{13}C NMR (101 MHz, $CDCl_3$) δ = 186.3, 163.1, 161.8, 156.0, 152.5, 151.8, 142.8, 142.6, 140.5, 138.2, 134.7, 133.3, 132.0, 130.2, 127.6, 126.2, 125.8, 125.6, 122.8, 120.9, 116.1, 110.7, 110.6, 97.5, 79.1, 78.0, 75.5, 70.9, 67.5, 56.3, 16.1, 13.1 ppm. IR (ATR): $\tilde{\nu}$ = 1677 (m), 1582 (s), 1453 (m), 1444 (m), 1402 (m), 1382 (m), 1273 (s), 1198 (m), 1167 (m), 1144 (m), 1088 (w), 1077 (w), 1014 (m), 888 (w), 806 (m), 783 (m), 727 (w), 701 (w), 684 cm^{-1} (w). HRMS (ESI, $[M+H]^+$) calcd for $C_{32}H_{26}IN_2O_4$, 629.0932; found, 629.0942.

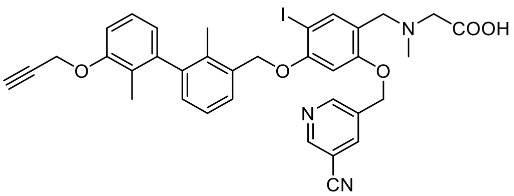

N-(5-Iodo-2-((5-cyanopyridin-3-yl)methoxy)-4-((2,2'-dimethyl-3'-(prop-2-yn-1-yloxy)-[1,1'-biphenyl]-3-yl)methoxy)benzyl)-N-methylglycine (**53c**). Aldehyde **52c** (120 mg, 191 mmol, 1.00 equiv.), sarcosine (85.1 mg, 955 mmol, 5.00 equiv.) and sodium cyanoborohydride (18.0 mg, 287 µmol, 1.50 equiv.) were reacted in a 1:1 mixture of abs. DMF/MeOH (3 mL) and subsequently worked up according to GP-3. After flash column chromatography on silica gel (MeCN:MeOH:Et_3N, 100:20:1, R_f = 0.2) compound **52c** (91.0 mg, 130 µmol, 68%) was obtained as a colorless solid. mp = 75 °C. R_t = 14.25 min (System A), purity: 97.7%. ^{1}H NMR (400 MHz,

methanol-d_4) δ = 8.97 (d, ^4J = 2.1 Hz, 1H), 8.90 (d, ^4J = 2.0 Hz, 1H), 8.40 (t, ^4J = 2.0 Hz, 1H), 7.91 (s, 1H), 7.50 (dd, ^4J = 1.0 Hz, ^3J = 7.6 Hz, 1H), 7.16–7.23 (m, 2H), 7.08 (dd, ^4J = 1.2 Hz, ^3J = 7.6 Hz, 1H), 7.01–7.03 (m, 1H), 6.95 (s, 1H), 6.73 (dd, ^4J = 0.8 Hz, ^3J = 7.5 Hz, 1H), 5.38 (s, 2H), 5.26 (s, 2H), 4.76 (dd, ^4J = 2.4 Hz, ^3J = 9.3 Hz, 2H), 4.34 (s, 2H), 3.59 (s, 2H), 2.94 (t, ^4J = 2.4 Hz, 1H), 2.79 (s, 3H), 2.08 (s, 3H), 1.88 ppm (s, 3H). ^{13}C NMR (101 MHz, methanol-d_4) δ = 170.0, 161.3, 159.9, 157.3, 153.4, 153.1, 144.1, 143.7, 143.5, 140.6, 136.1, 135.7, 134.3, 130.7, 128.9, 127.1, 126.5, 126.3, 123.5, 117.4, 114.4, 111.9, 111.5, 99.7, 80.1, 76.8, 76.6, 71.6, 68.5, 59.1, 57.0, 55.0, 49.9, 41.8, 16.2, 13.3 ppm. IR (ATR): ṽ = 1626 (m), 1597 (w), 1574 (m), 1495 (w), 1455 (m), 1380 (s), 1289 (m), 1255 (m), 1202 (w), 1158 (s), 1145 (m), 1087 (w), 1076 (w), 1014 (m), 784 (w), 721 (w), 699 cm^{-1} (m). HRMS (ESI, [M+H]$^+$) calcd for $C_{35}H_{33}IN_3O_5$, 702.1460; found, 702.1462.

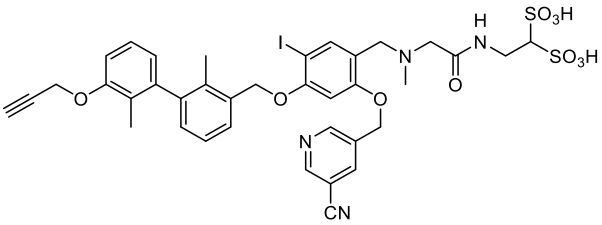

2-(2-((2-((5-Cyanopyridin-3-yl)methoxy)-4-((2,2'-dimethyl-3'-(prop-2-yn-1-yloxy)-[1,1'-biphenyl]-3-yl)methoxy)-5-iodobenzyl)(methyl)amino)acetamido)ethane-1,1-disulfonic acid (**54c**). Carboxylic acid **53c** (85.0 mg, 121 µmol, 1.00 equiv.) reacted with 2-aminoethane-1,1-disulfonic acid (TBA salt) (**37**) (237 mg, 606 mol, 5.00 equiv.) in presence of DIPEA (42.1 µL, 242 µmol, 2.00 equiv.) and HTBU (101 mg, 267 µmol, 2.20 equiv.) in abs. DMF (2 mL) according to GP-5. Purification was performed by semi-preparative RP-HPLC (Agilent Zorbax SB C-18 5 µm 80 Å, 9.4 x 250mm with 30-90% acetonitrile (0.1% TFA) in water (0.1% TFA) in a linear gradient over 45 min, 6 mL/min, R_t = 12 min) and after cation exchange and lyophilization, compound **54c** (60.0 mg, 67.5 µmol, 56%) was obtained as a colorless solid. mp = 150 °C (decomposition). R_t = 11.58 min (System A), purity: 96.1%. ^{1}H NMR (600 MHz, DMSO-d_6) δ = 9.54 (br, 1H), 9.09 (s, 1H), 9.07 (s, 1H), 8.57 (s, 1H), 8.16 (t, ^3J = 5.1 Hz, 1H), 7.92 (s, 1H), 7.58 (d, ^3J = 7.5 Hz, 1H), 7.28 (t, ^3J = 7.6 Hz, 1H), 7.24 (t, ^3J = 7.9 Hz, 1H), 7.09–7.10 (m, 2H), 7.06 (d, ^3J = 8.3 Hz, 1H), 6.75 (d, ^3J = 7.5 Hz, 1H), 5.39–5.44 (m, 2H), 5.25–5.30 (m), 4.85 (d, ^4J = 2.2 Hz, 2H), 4.23–4.32 (m, 2H), 3.86–3.94 (m, 2H), 3.67–3.76 (m, 2H), 3.58–3.60 (m, 2H), 2.69 (s, 3H), 2.06 (s, 3H), 1.85 ppm (s, 3H). ^{13}C NMR (151 MHz, DMSO-d_6) δ = 163.5, 159.1, 158.4, 155.4, 151.9, 151.4, 142.5, 142.4, 141.4, 139.8, 134.6, 134.5, 133.0, 129.2, 127.7, 126.2,

125.4, 124.2, 122.0, 116.6, 112.6, 110.9, 109.3, 99.1, 79.5, 78.1, 76.0, 74.0, 69.8, 67.0, 56.1, 55.9, 52.7, 40.6, 15.6, 12.8 ppm. IR (ATR): ṽ = 3351 (m), 1676 (m), 1589 (w), 1573 (w), 1440 (w), 1403 (w), 1205 (s), 1168 (s), 1064 (w), 1014 (m), 644 (w), 586 cm^{-1} (w). HRMS (ESI, $[M+H]^+$) calcd for $C_{37}H_{38}IN_4O_{10}S_2$, 889.1069; found, 889.1067.

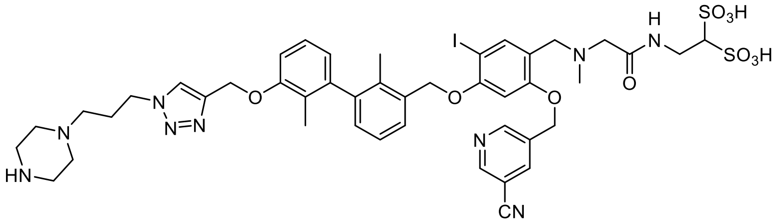

2-(2-((2-((5-Cyanopyridin-3-yl)methoxy)-4-((2,2'-dimethyl-3'-((1-(3-(piperazin-1-yl)propyl)-1H-1,2,3-triazol-4-yl)methoxy)-[1,1'-biphenyl]-3-yl)methoxy)-5-iodobenzyl)(methyl)amino)acetamido)ethane-1,1-disulfonic acid (**55c**). Alkyne **54c** (20.0 mg, 22.5 µmol, 1.00 equiv.) reacted with 1-(3-azidopropyl)piperazine (**37**) (7.62 mg, 45.0 µmol, 2.00 equiv.) with a premixed catalyst consisting of $CuSO_4$ (1.08 mg, 6.75 µmol, 0.30 equiv.), THPTA (1.27 mg, 2.93 µmol, 0.13 equiv.) and sodium ascorbate (22.3 mg, 113 µmol, 5.00 equiv.) in an 1:1 mixture of H_2O/t-BuOH (2 mL) at room temperature for 5 h according to GP-5. Purification by semi-preparative RP-HPLC (System C, R_t = 22 min) and subsequent lyophilization yielded compound **55c** (15.9 mg, 15.0 µmol, 67%) as a colorless solid. mp = 205 °C (decomposition). R_t = 9.83 min (System A), purity: 95.8%. ^1H NMR (600 MHz, DMSO-d_6) δ = 9.57 (br, 2H), 9.02 (dd, ^4J = 1.9 Hz, ^3J = 7.5 Hz, 2H), 8.48 (s, 1H), 8.28 (s, 1H), 8.13 (br, 1H), 7.88 (s, 1H), 7.54 (d, ^3J = 7.5 Hz, 1H), 7.22–7.27 (m, 2H), 7.16 (d, ^3J = 8.3 Hz, 1H), 7.04–7.07 (m, 2H), 6.72 (d, ^3J = 7.4 Hz, 1H), 5.33–5.35 (m, 4H), 5.17–5.22 (m, 2H), 4.46 (t, ^3J = 7.0 Hz, 2H), 4.26 (br, 2H), 3.42-3.80 (m), 2.69 (s, 3H), 2.26 (br, 2H), 2.02 (s, 3H), 1.76 ppm (s, 3H). ^{13}C NMR (151 MHz, DMSO-d_6) δ = 163.6, 159.0, 158.3, 158.0, 156.1, 152.4, 152.0, 143.1, 142.4, 142.2, 141.7, 139.0, 134.8, 134.5, 132.5, 129.2, 128.0, 126.3, 125.4, 124.4, 124.2, 121.6, 116.9, 112.4, 110.9, 99.2, 76.0, 73.9, 70.0, 67.0, 61.8, 55.8, 53.7, 52.8, 48.4, 46.9, 41.0, 40.6, 38.9, 24.4, 15.7, 12.8 ppm. IR (ATR): ṽ = 1679 (s), 1606 (w), 1575 (w), 1505 (w), 1305 (m), 1199 (s), 1179 (s), 1145 (s), 1063 (w), 1017 (m), 721 (w), 593 cm^{-1} (w). HRMS (ESI, $[M+H]^+$) calcd for $C_{44}H_{53}IN_9O_{10}S_2$, 1058.2396; found, 1058.2400.

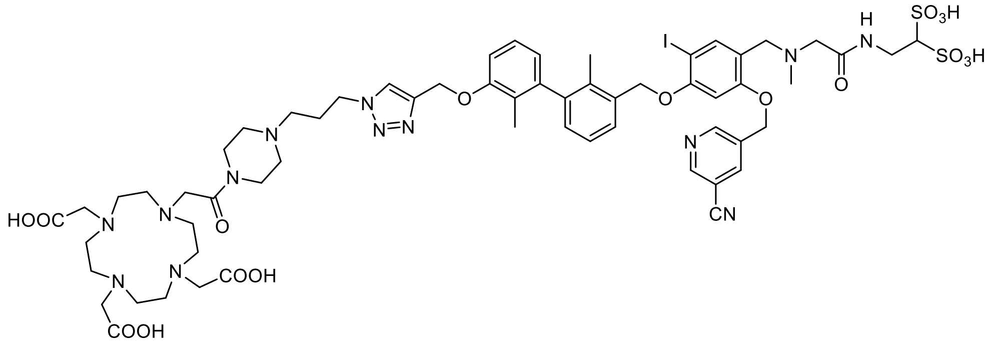

2,2',2''-(10-(2-(4-(3-(4-(((3'-((5-((5-Cyanopyridin-3-yl)methoxy)-4-(((2-((2,2-disulfoethyl)amino)-2-oxoethyl)(methyl)amino)methyl)-2-iodophenoxy)methyl)-2,2'-dimethyl-[1,1'-biphenyl]-3-yl)oxy)methyl)-1H-1,2,3-triazol-1-yl)propyl)piperazin-1-yl)-2-oxoethyl)-1,4,7,10-tetraazacyclododecane-1,4,7-triyl)triacetic acid (**56c**). Propyl-piperazinyl linker structure **55c** (15.0 mg, 14.2 µmol, 1.00 equiv.), DOTA-*p*-nitrophenylester (**61**) (11.2 mg, 21.3 µmol, 1.50 equiv.) and abs. DIPEA (12.4 µL, 70.9 µmol, 5.00 equiv.) were dissolved in abs. DMF (1 mL) and reacted according to GP-7. Purification by semi-preparative RP-HPLC (System C, R_t = 19 min) and subsequent lyophilization yielded DOTA-conjugate **56c** (10.7 mg, 7.4 µmol, 52%) as a colorless powder. mp = 185 °C (decomposition). R_t = 9.91 min (System A), purity: 97.5%. IR (ATR): ṽ = 1668 (s), 1456 (m), 1386 (m), 1197 (s), 1014 cm^{-1} (s). HRMS (ESI, [M+H]$^+$) calcd for $C_{60}H_{79}IN_{13}O_{17}S_2$, 1444.4198; found, 1444.4191.

2. ^{1}H and ^{13}C NMR spectra of literature unknown compounds

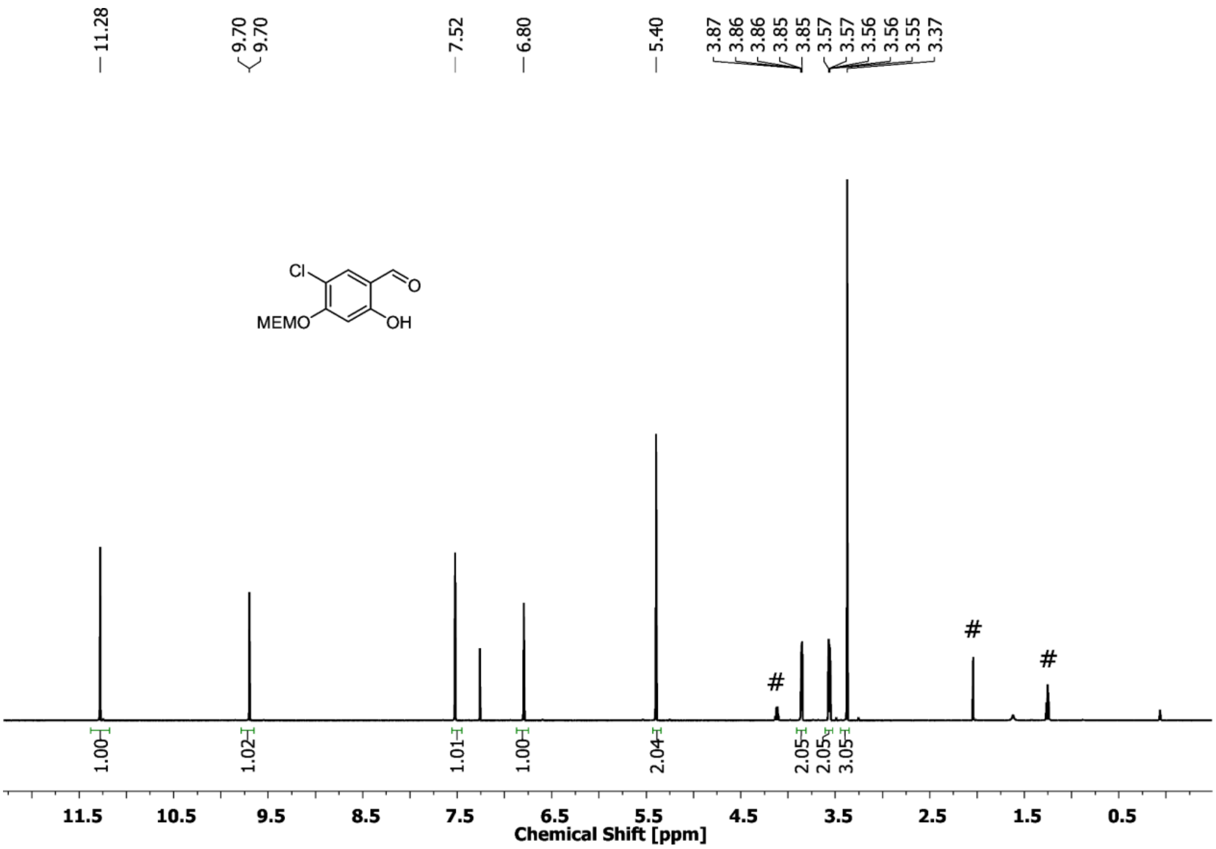

Figure S1: ^{1}H NMR spectrum ($CDCl_3$, 600 MHz, 298 K) of compound 6.

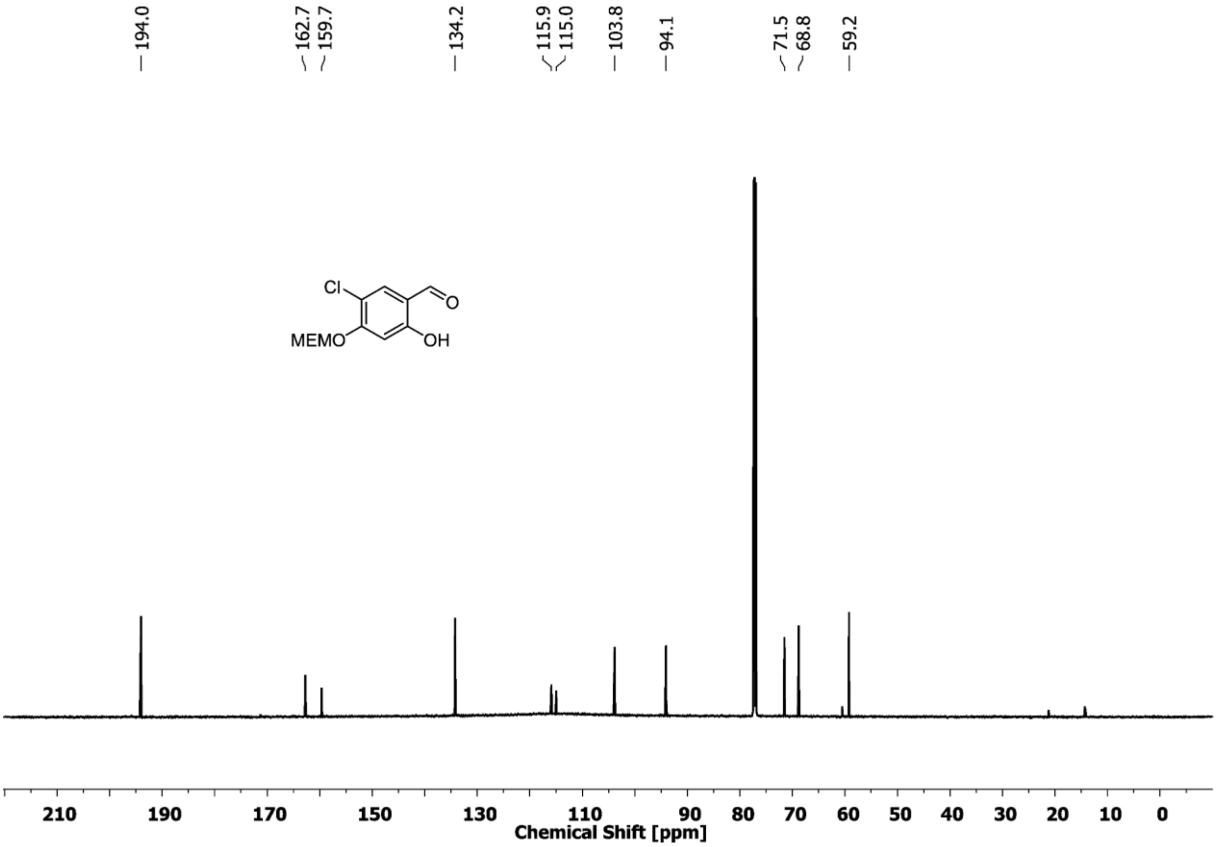

Figure S2: ^{13}C NMR spectrum ($CDCl_3$, 151 MHz, 298 K) of compound 6.

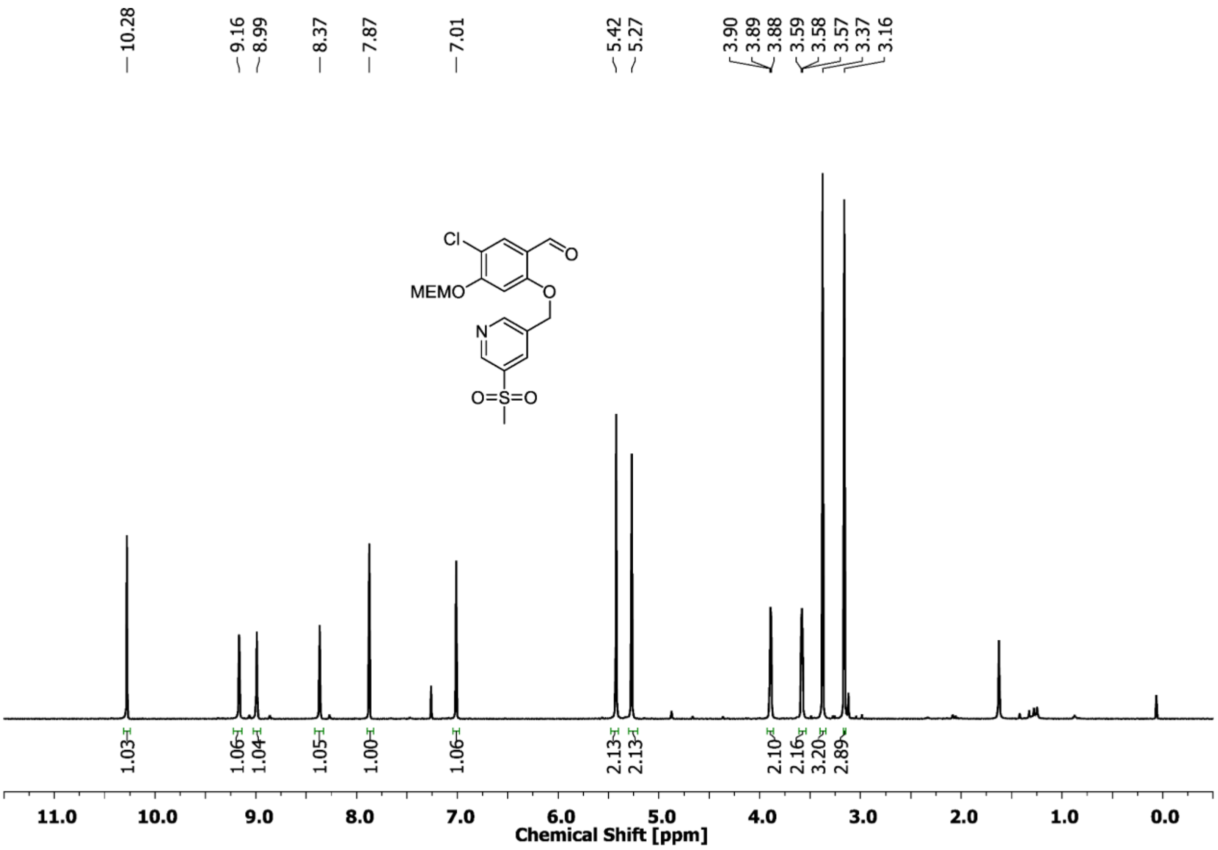

Figure S3: ^{1}H NMR spectrum ($CDCl_3$, 400 MHz, 298 K) of compound 10.

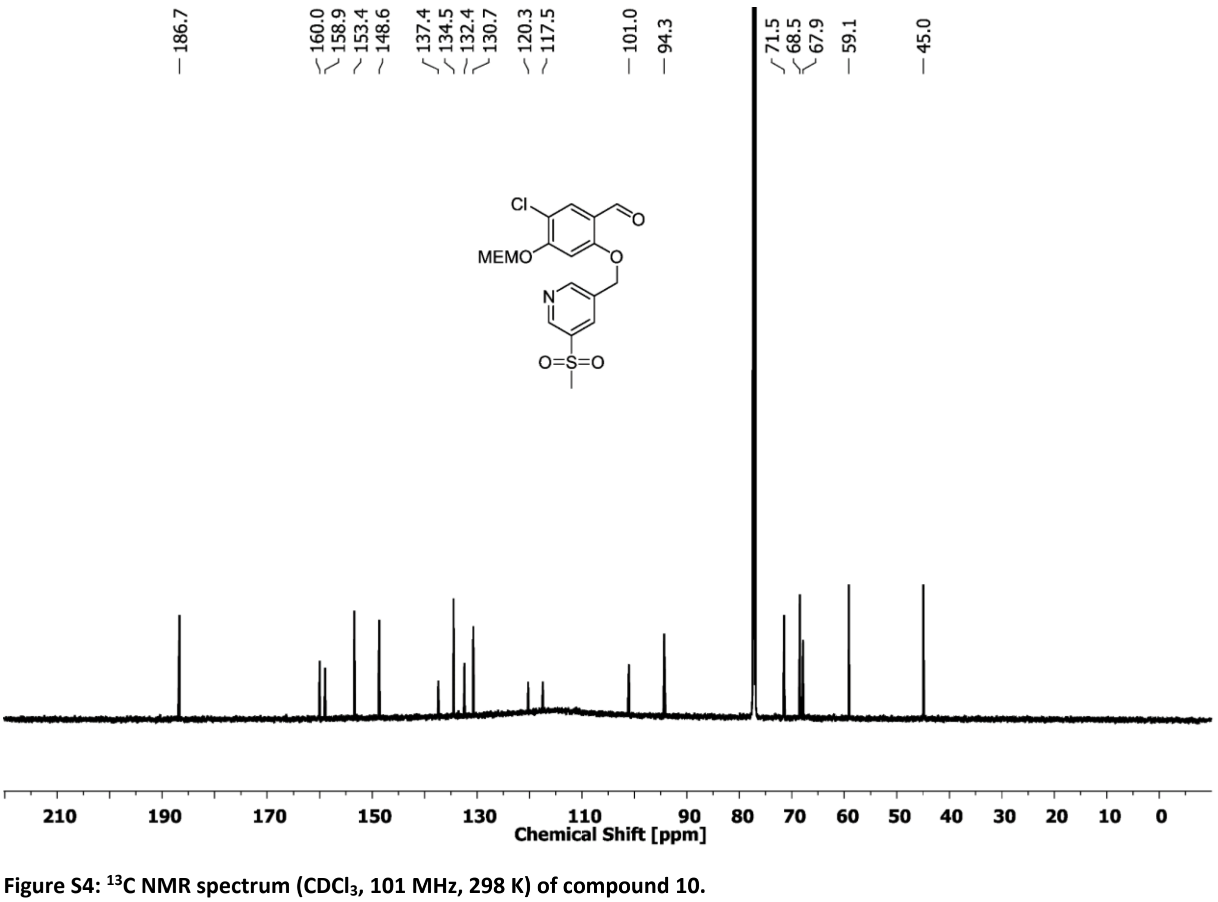

Figure S4: ^{13}C NMR spectrum ($CDCl_3$, 101 MHz, 298 K) of compound 10.

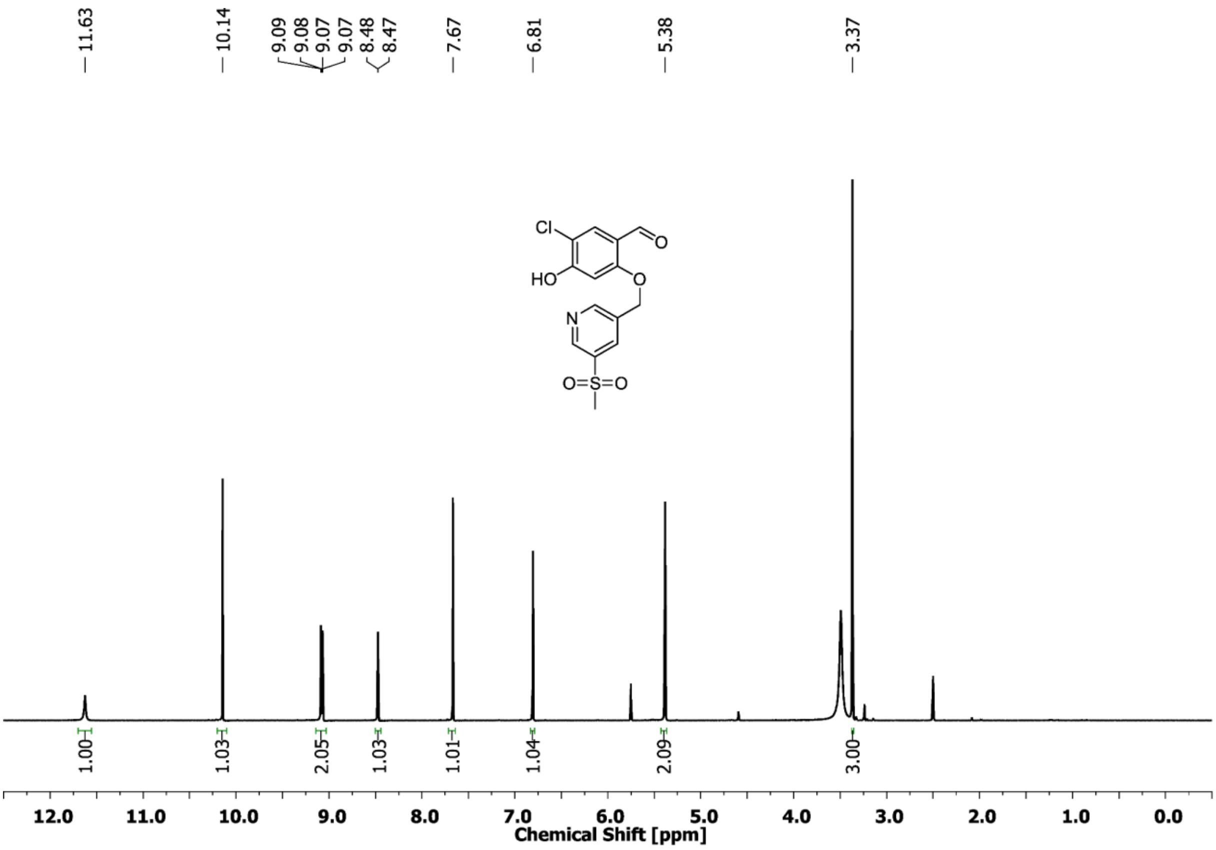

Figure S5: ^{1}H NMR spectrum (DMSO-d_6, 400 MHz, 298 K) of compound 11.

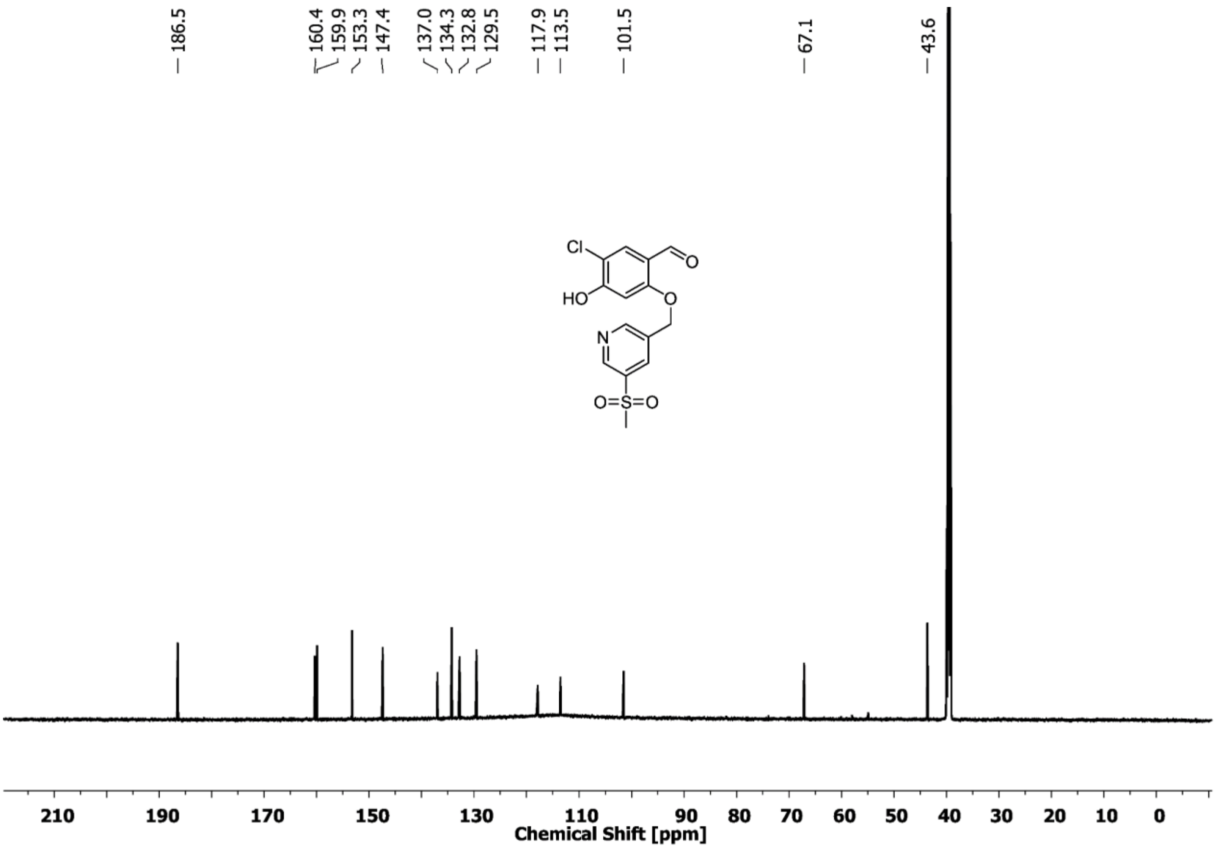

Figure S6: ^{13}C NMR spectrum (DMSO-d_6, 101 MHz, 298 K) of compound 11.

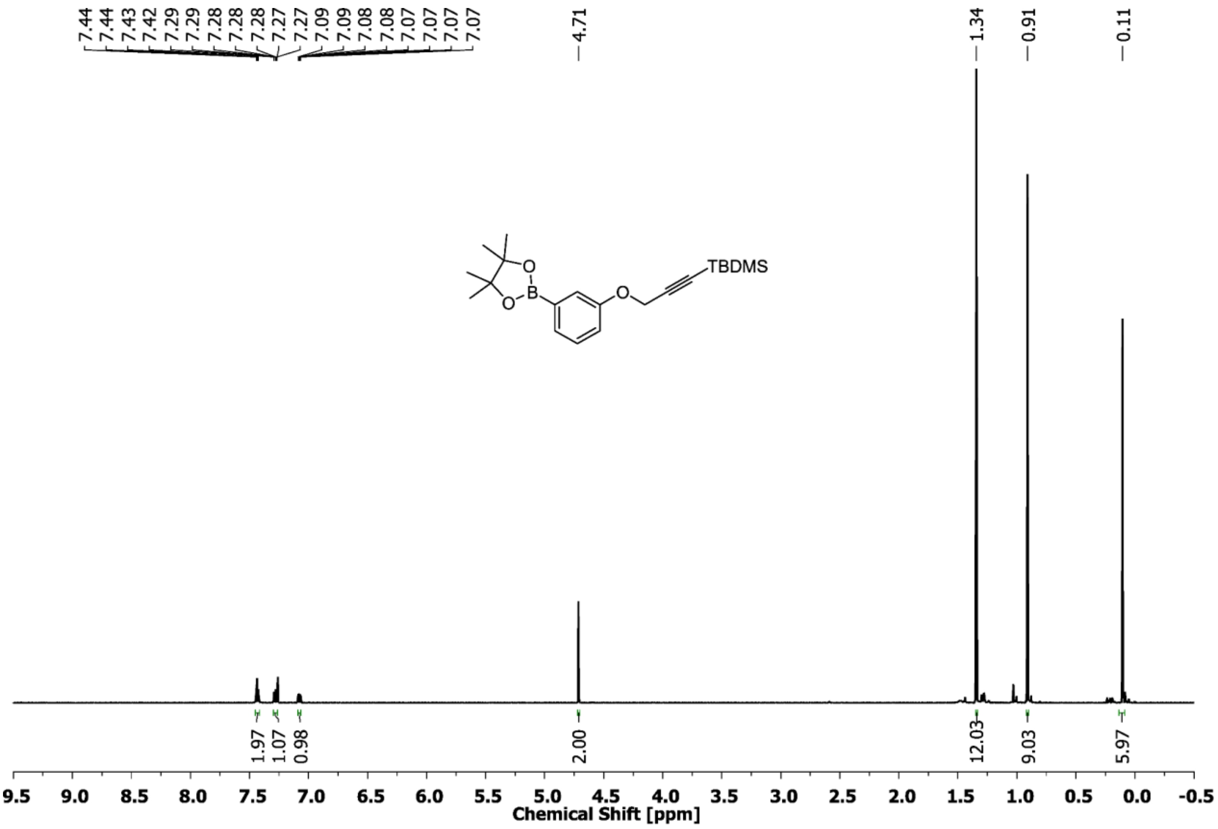

Figure S7: ^{1}H NMR spectrum ($CDCl_3$, 600 MHz, 298 K) of compound 17.

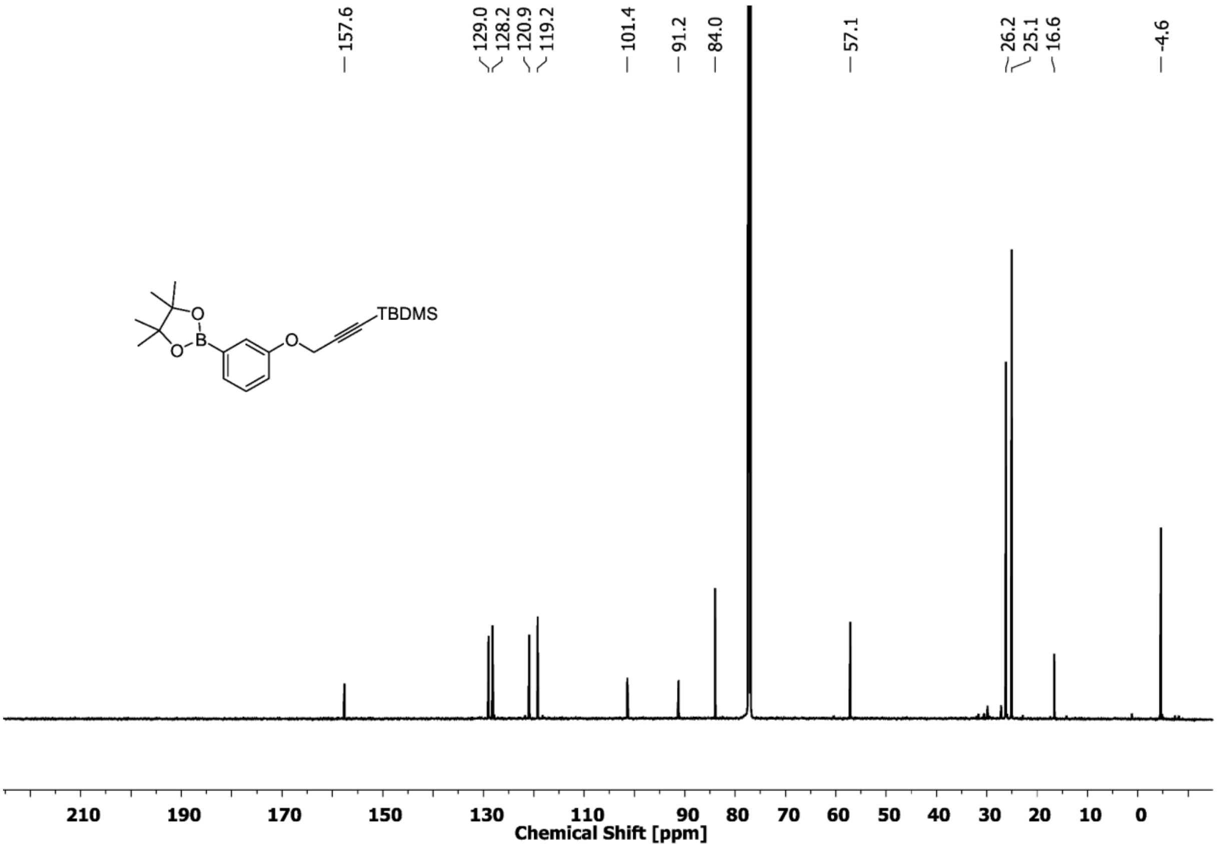

Figure S8: ^{13}C NMR spectrum ($CDCl_3$, 151 MHz, 298 K) of compound 17.

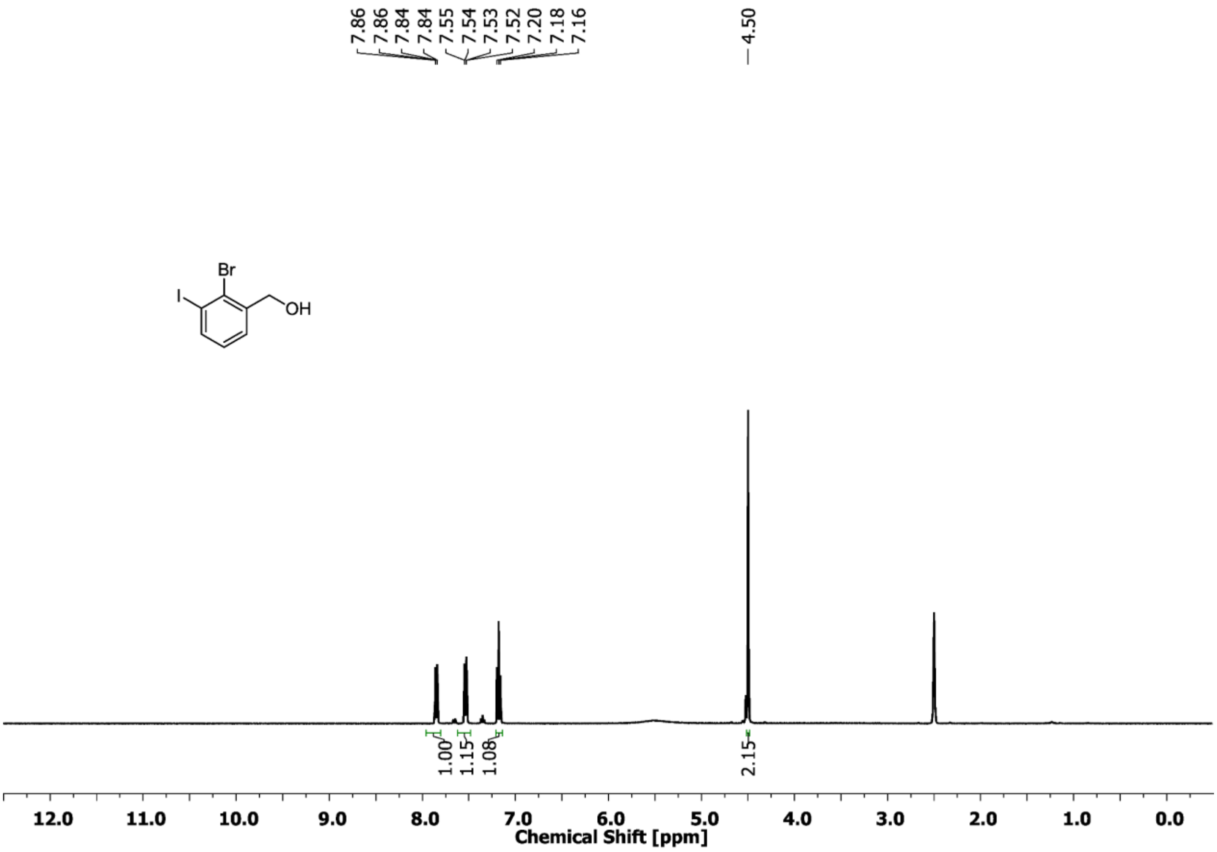

Figure S9: ^{1}H NMR spectrum ($CDCl_3$, 400 MHz, 298 K) of compound 21.

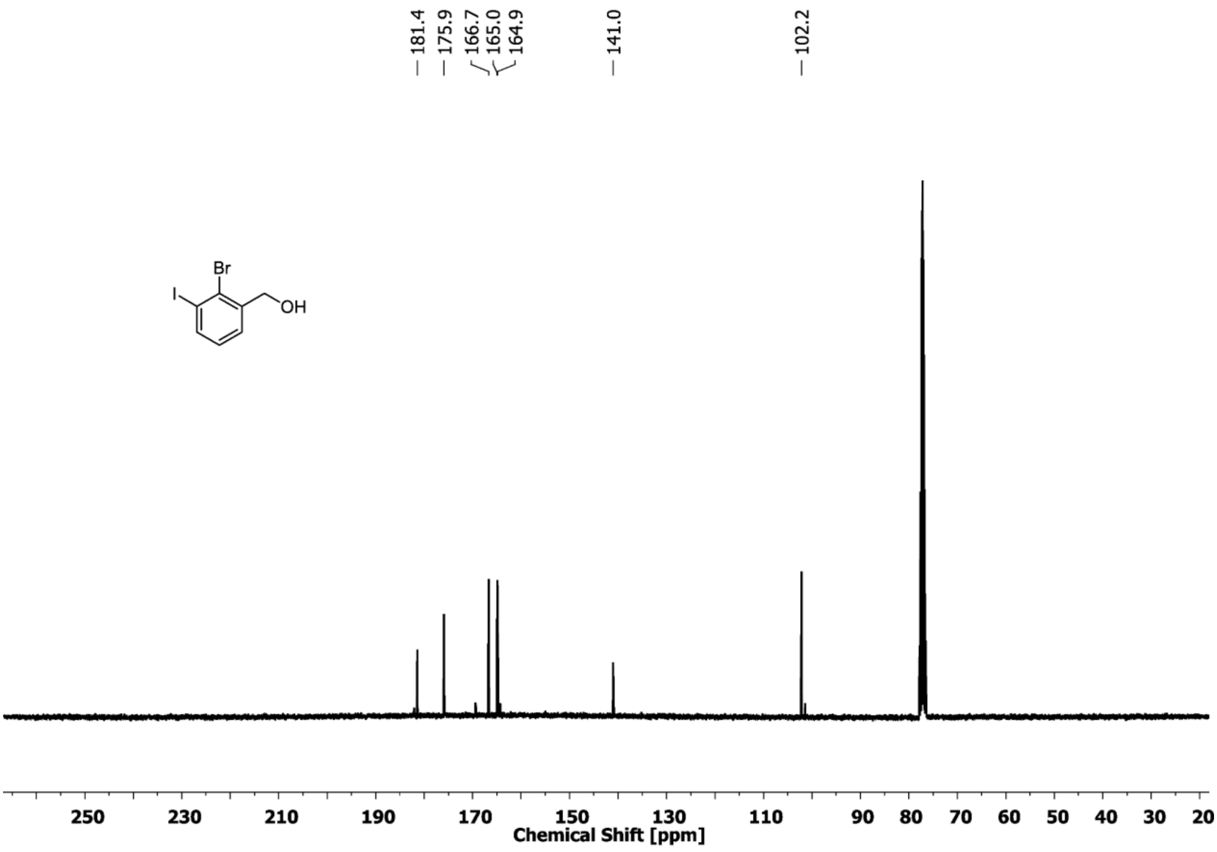

Figure S10: ^{13}C NMR spectrum ($CDCl_3$, 101 MHz, 298 K) of compound 21.

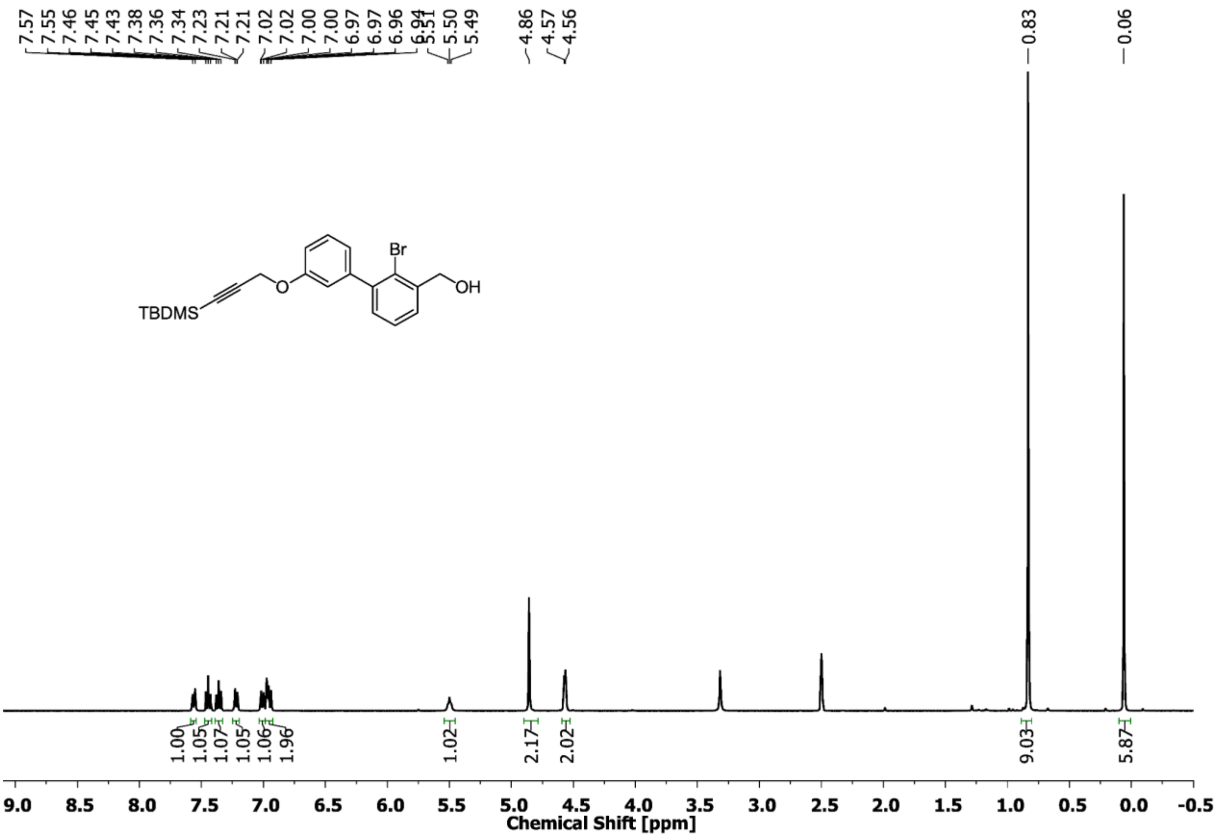

Figure S11: ^{1}H NMR spectrum (DMSO-d_6, 400 MHz, 298 K) of compound 22.

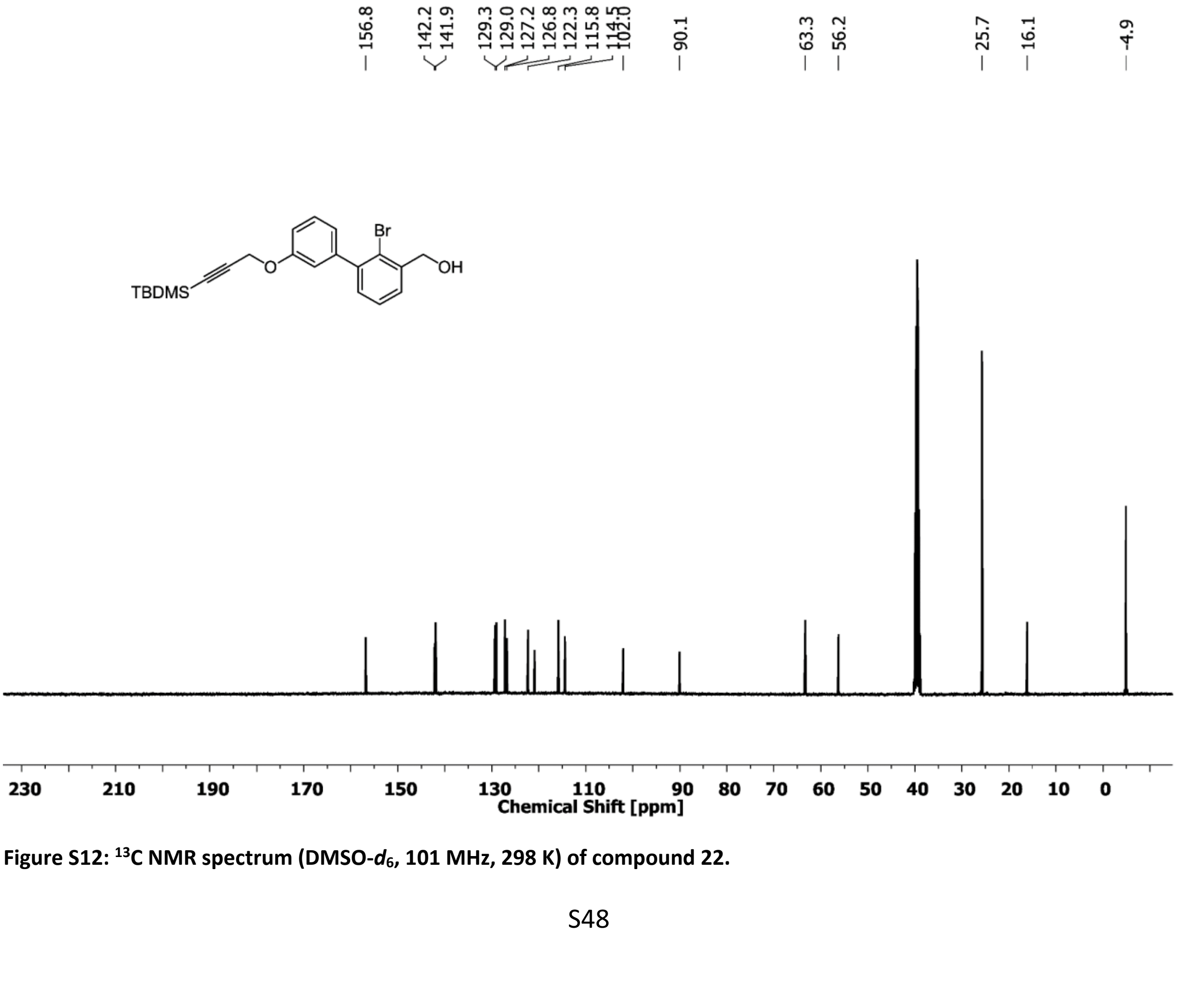

Figure S12: ^{13}C NMR spectrum (DMSO-d_6, 101 MHz, 298 K) of compound 22.

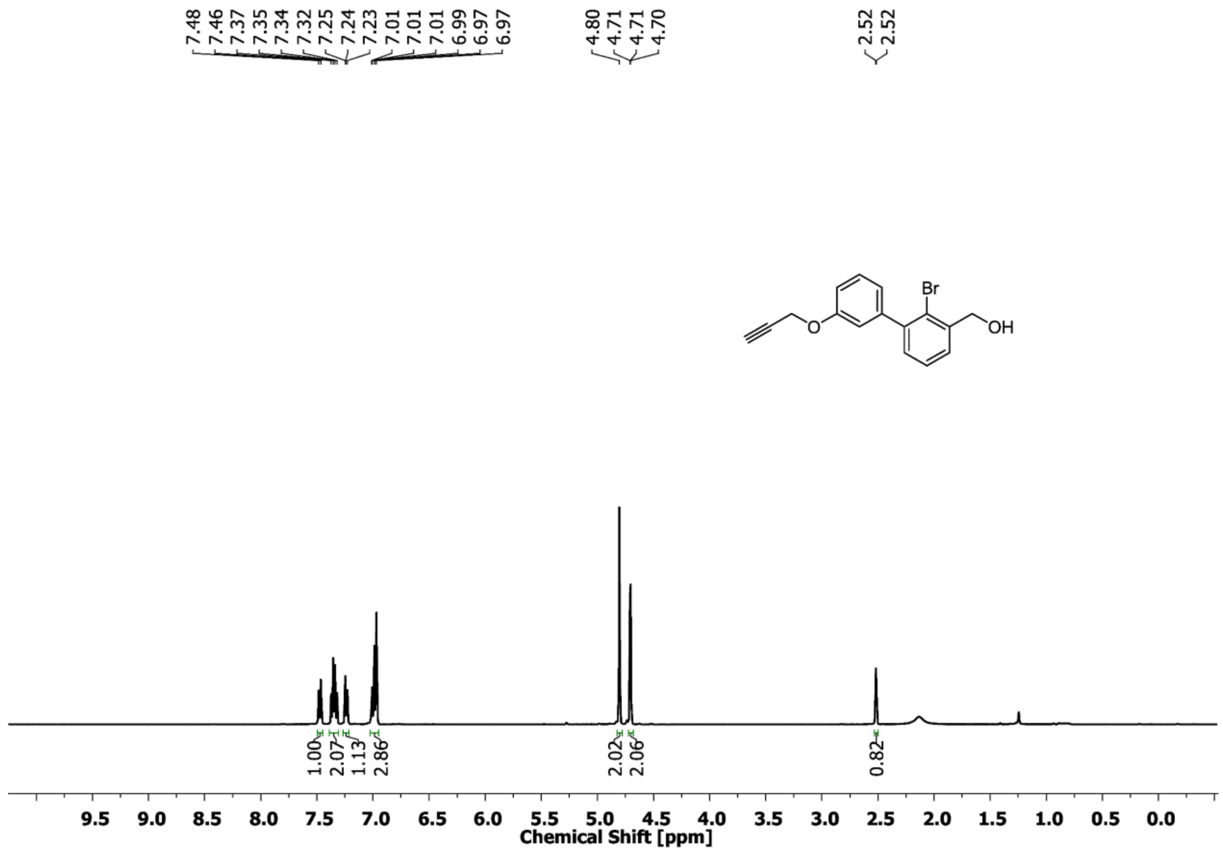

Figure S13: ^{1}H NMR spectrum ($CDCl_3$, 400 MHz, 298 K) of compound 28a.

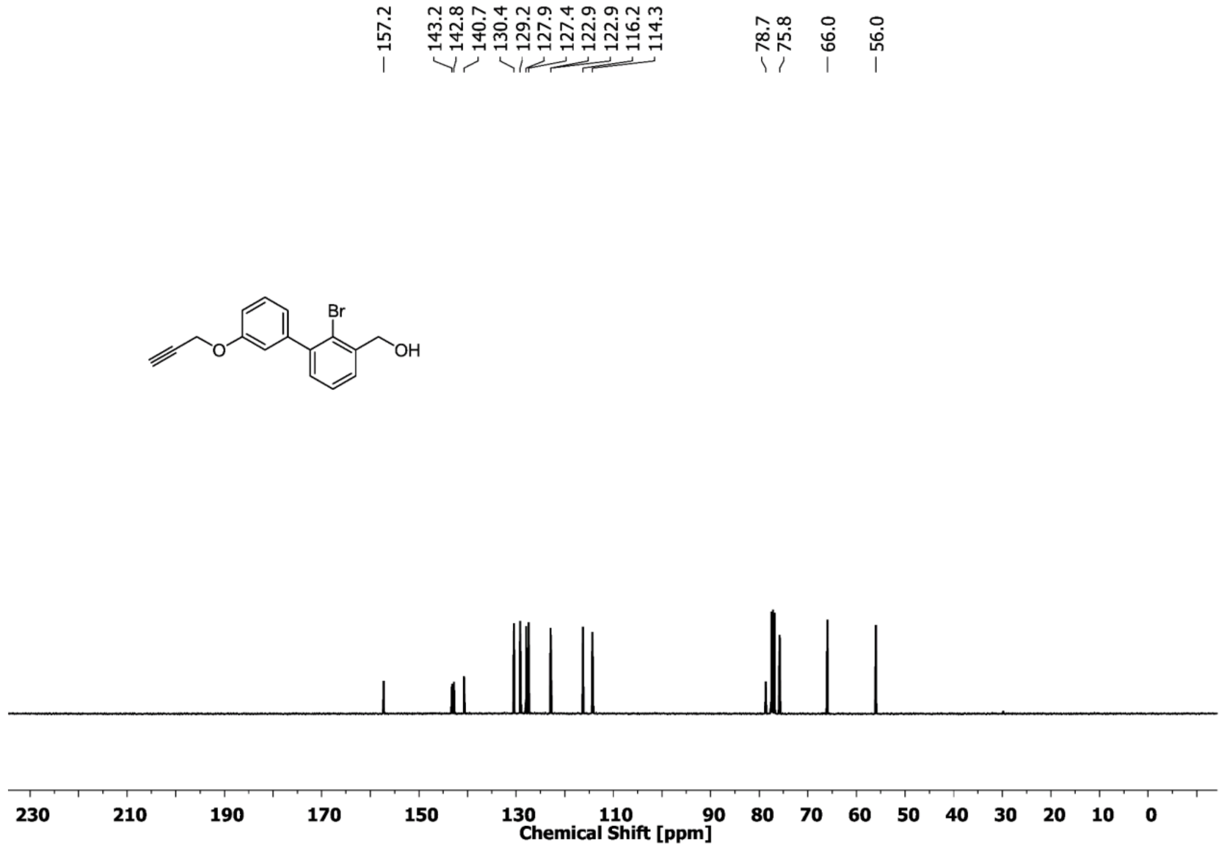

Figure S14: ^{13}C NMR spectrum ($CDCl_3$, 101 MHz, 298 K) of compound 28a.

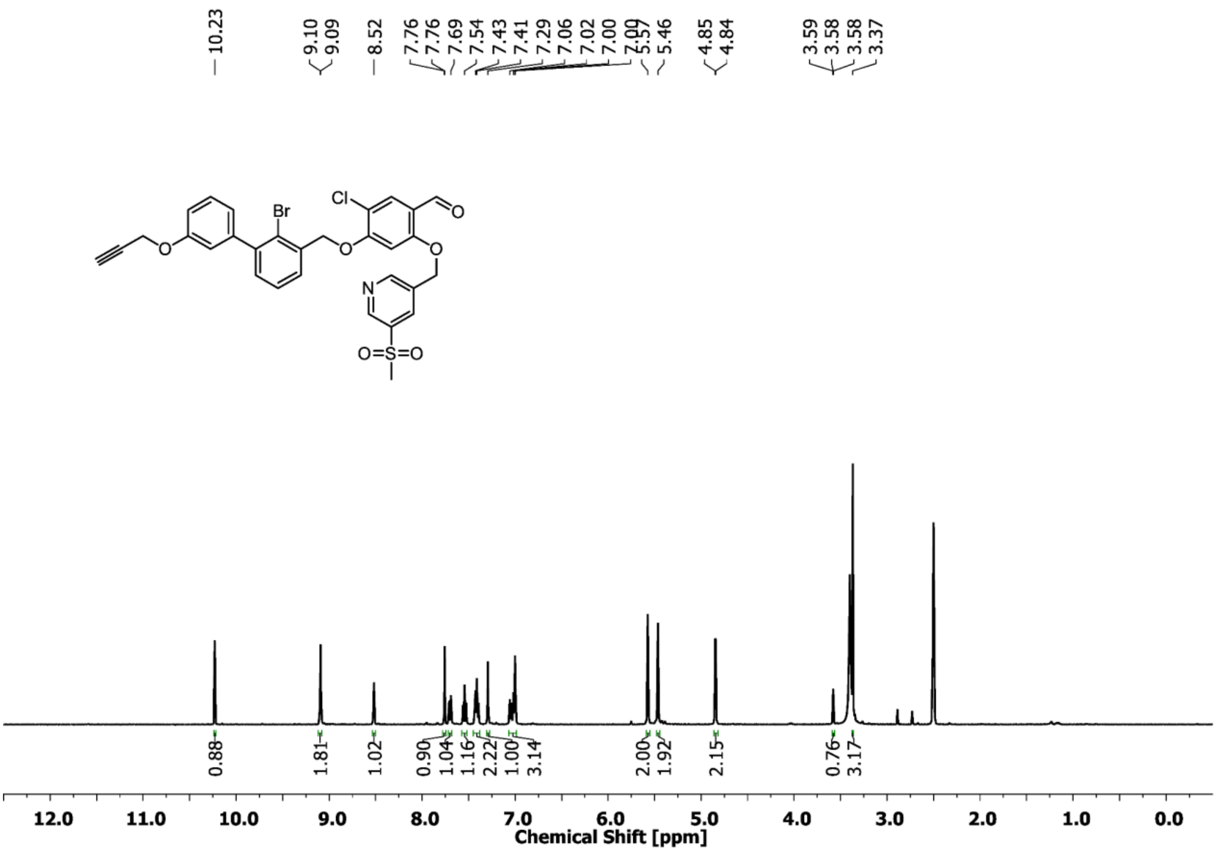

Figure S15: ^{1}H NMR spectrum ($CDCl_3$, 400 MHz, 298 K) of compound 29a.

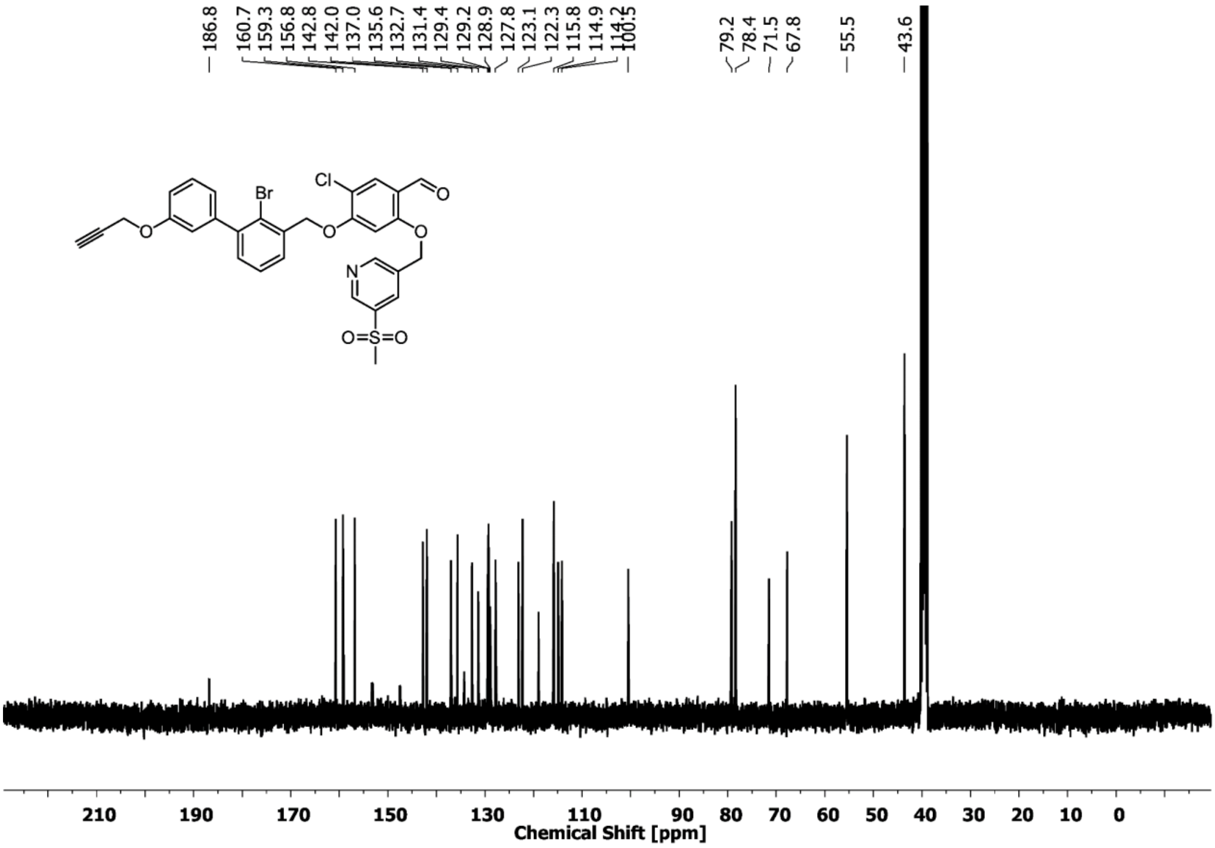

Figure S16: ^{13}C NMR spectrum ($CDCl_3$, 101 MHz, 298 K) of compound 29a.

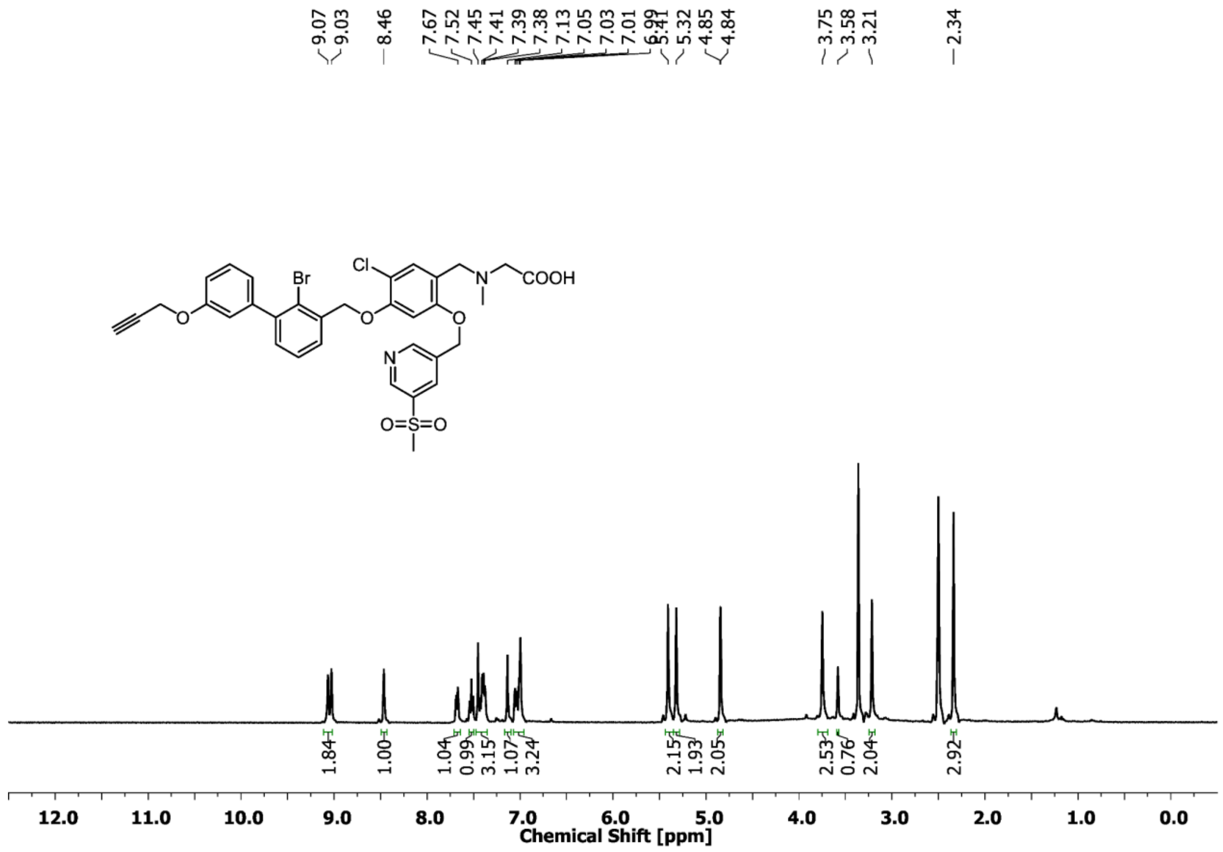

Figure S17: ^{1}H NMR spectrum (methanol-d_4, 400 MHz, 298 K) of compound 30a.

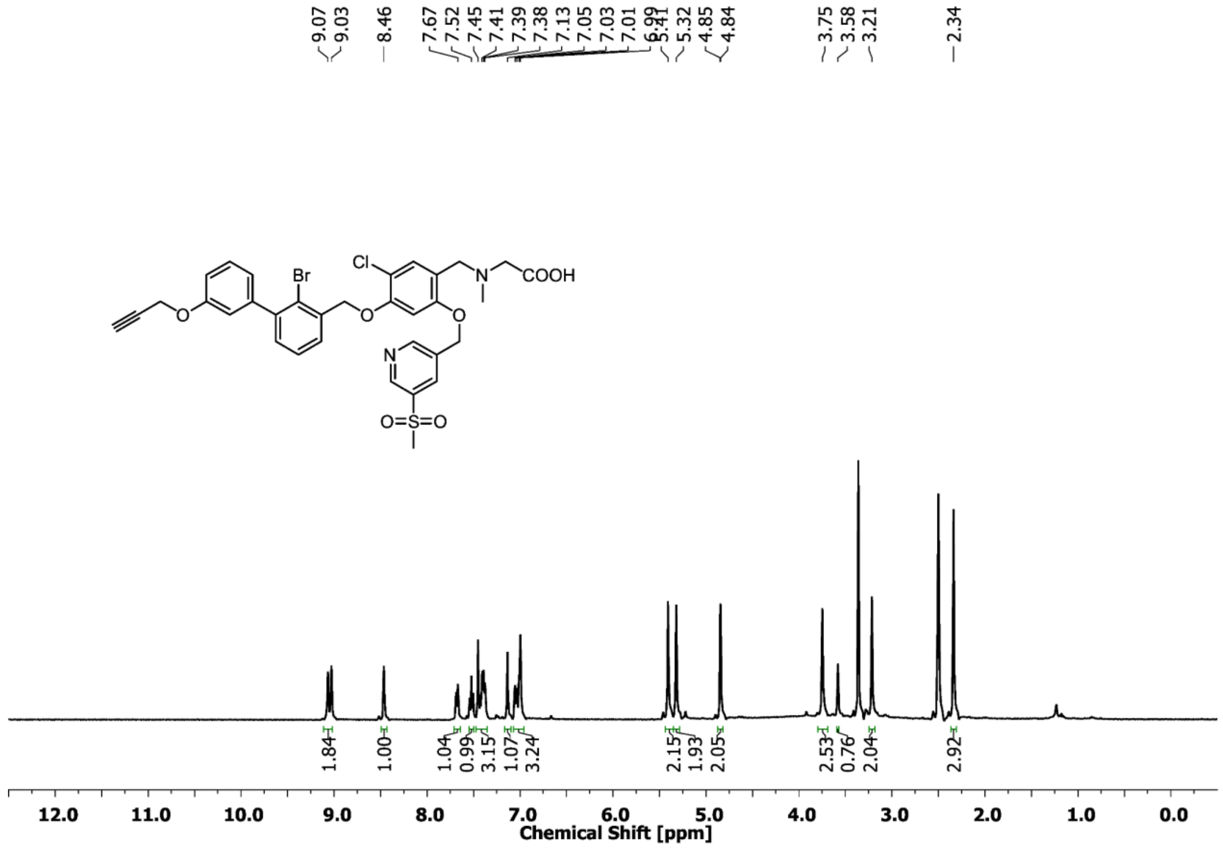

Figure S18: ^{13}C NMR spectrum (methanol-d_4, 101 MHz, 298 K) of compound 30a.

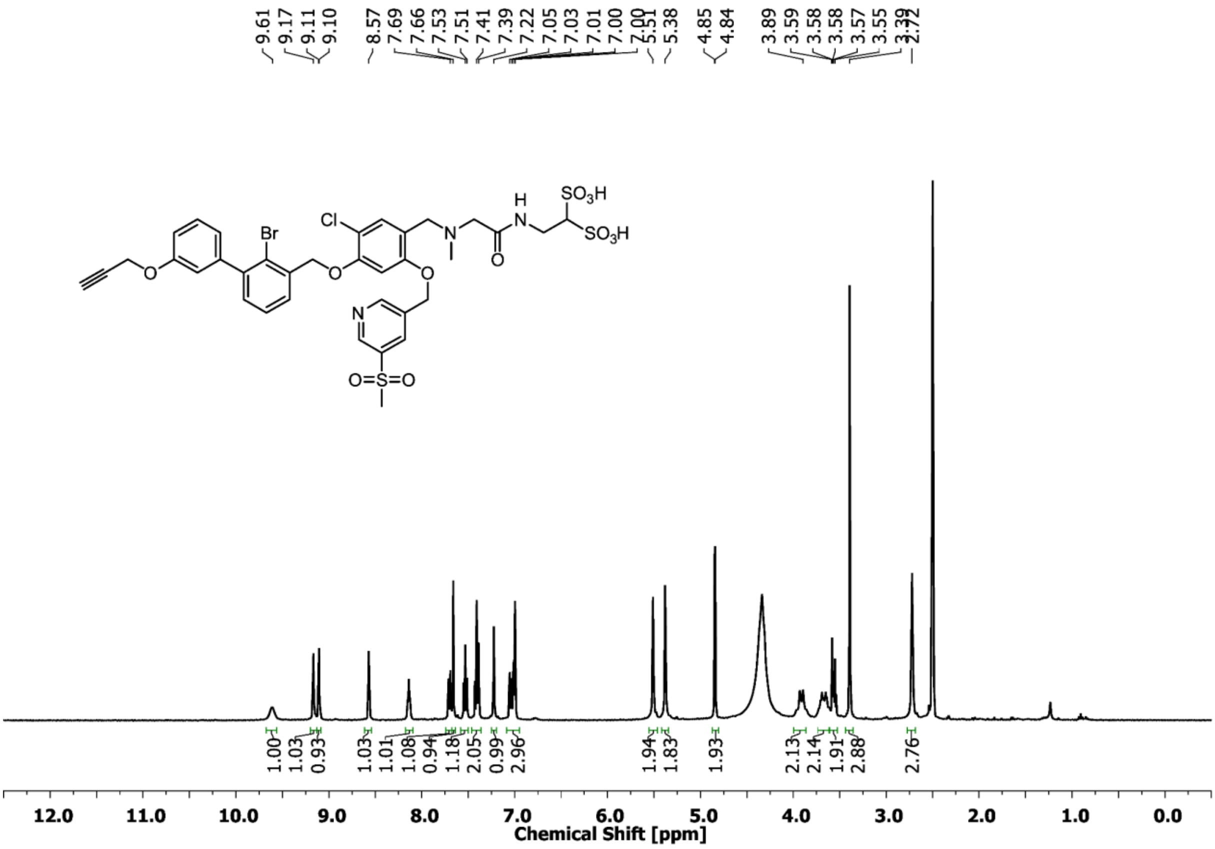

Figure S19: ^{1}H NMR spectrum (DMSO-d_6, 400 MHz, 298 K) of compound 33b.

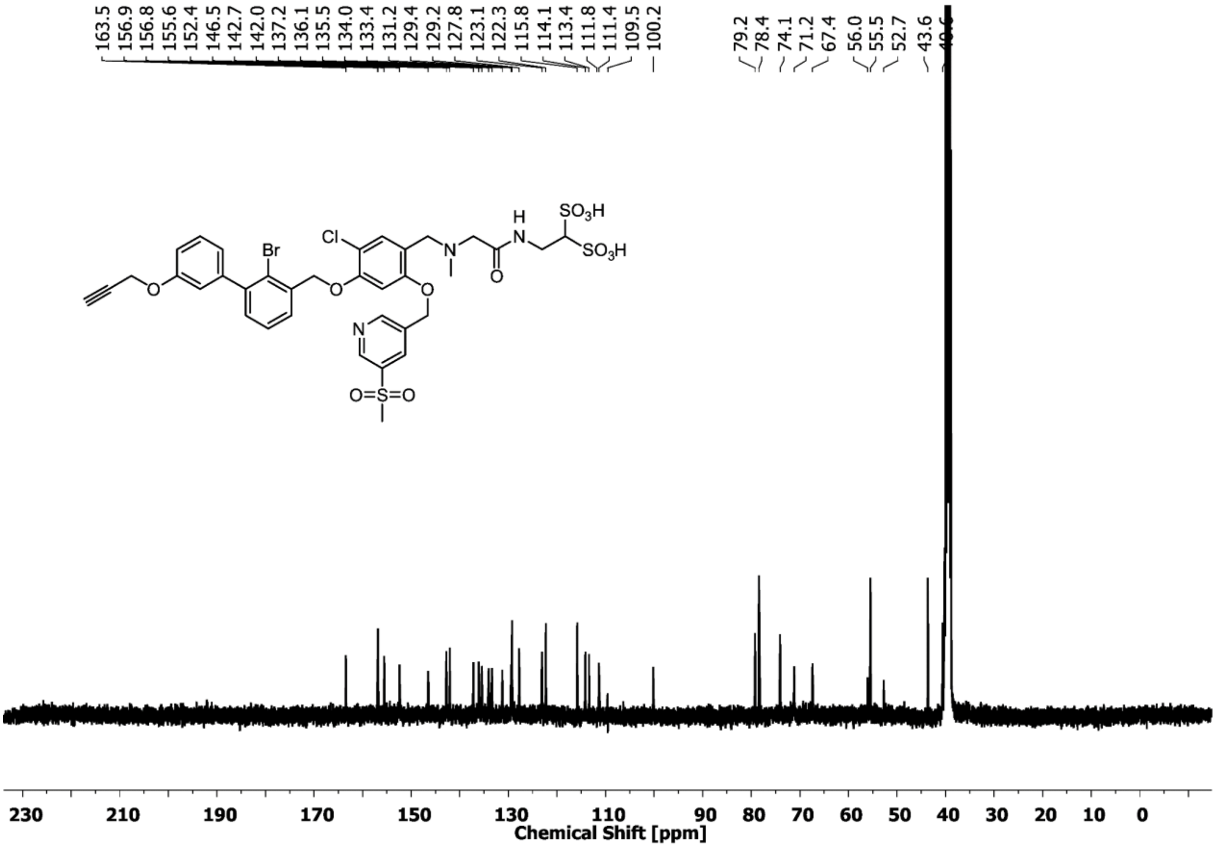

Figure S20: ^{13}C NMR spectrum (DMSO-d_6, 101 MHz, 298 K) of compound 33b.

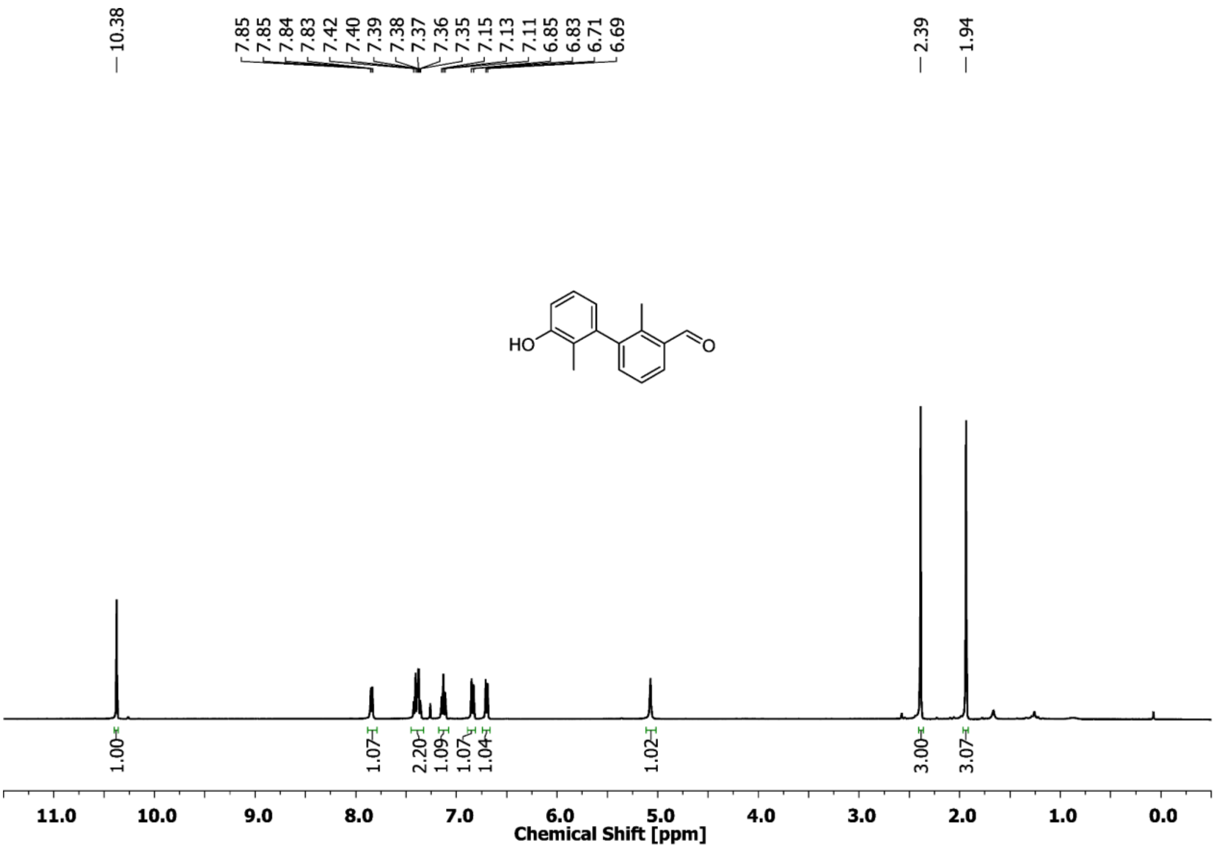

Figure S21: ^{1}H NMR spectrum ($CDCl_3$, 600 MHz, 298 K) of compound 26.

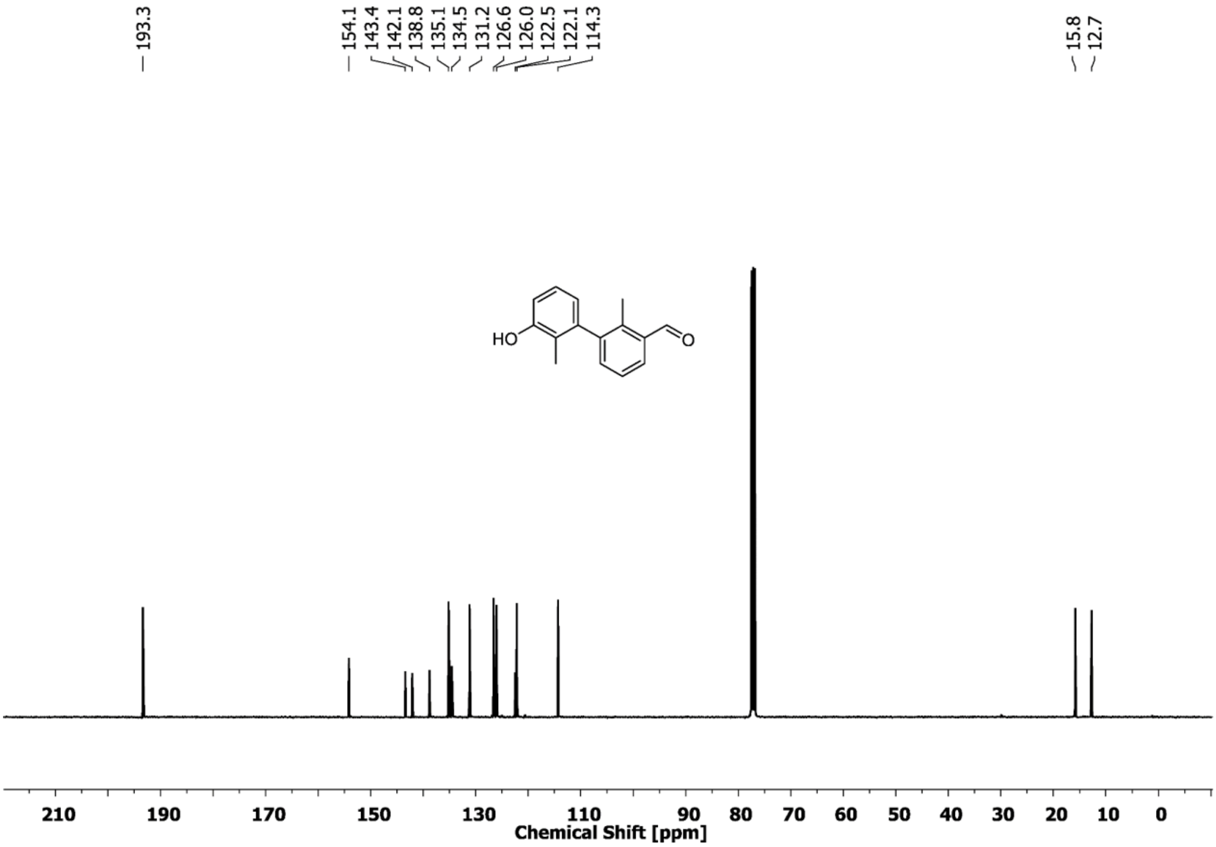

Figure S22: ^{13}C NMR spectrum ($CDCl_3$, 151 MHz, 298 K) of compound 26.

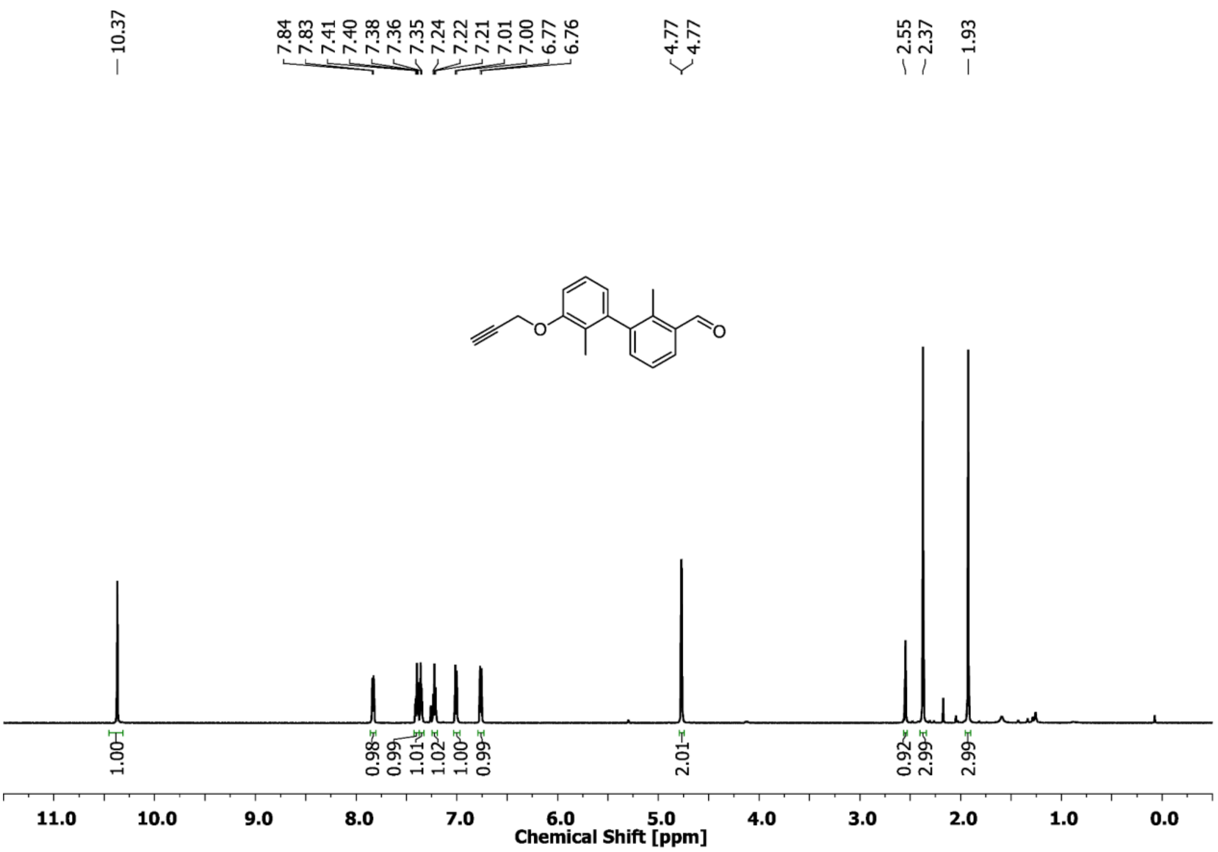

Figure S23: ^{1}H NMR spectrum (CDCl$_3$, 600 MHz, 298 K) of compound 27.

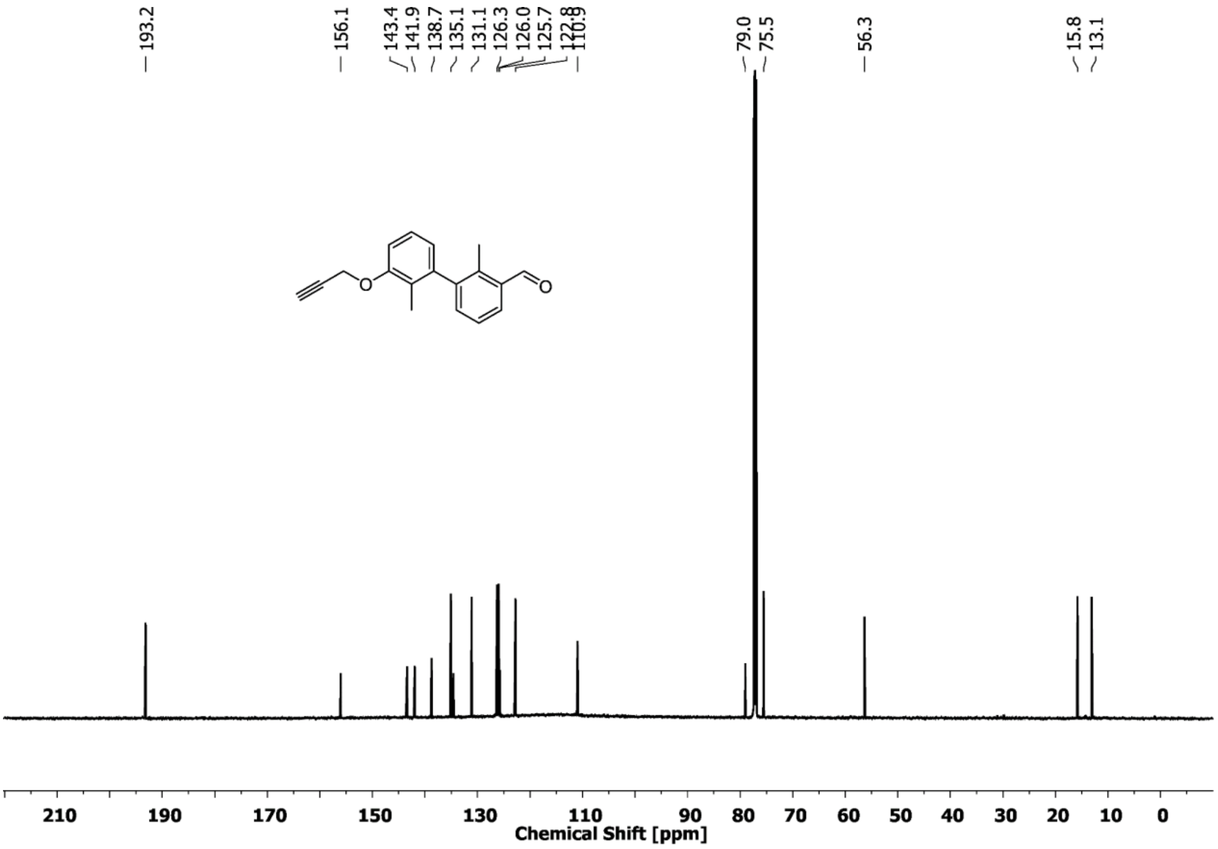

Figure S24: ^{13}C NMR spectrum (CDCl$_3$, 151 MHz, 298 K) of compound 27.

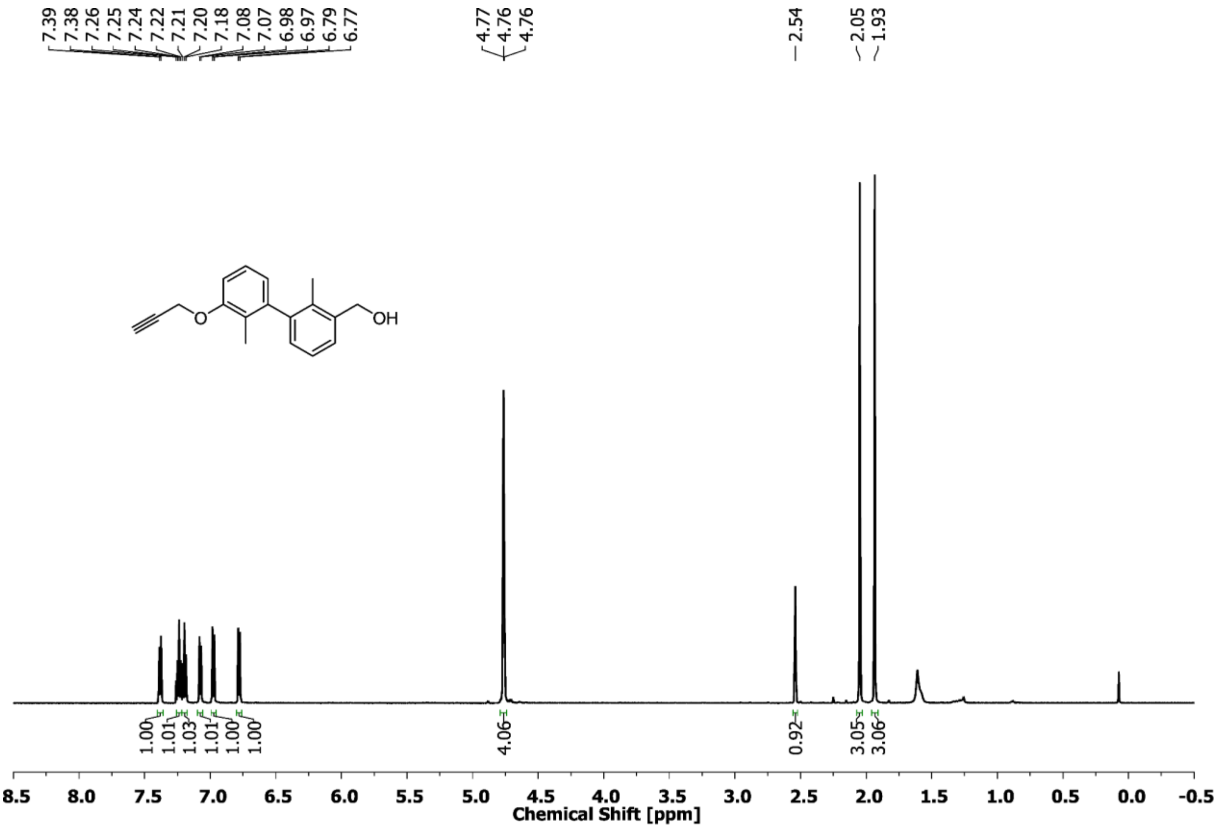

Figure S25: ^{1}H NMR spectrum ($CDCl_3$, 600 MHz, 298 K) of compound 28b.

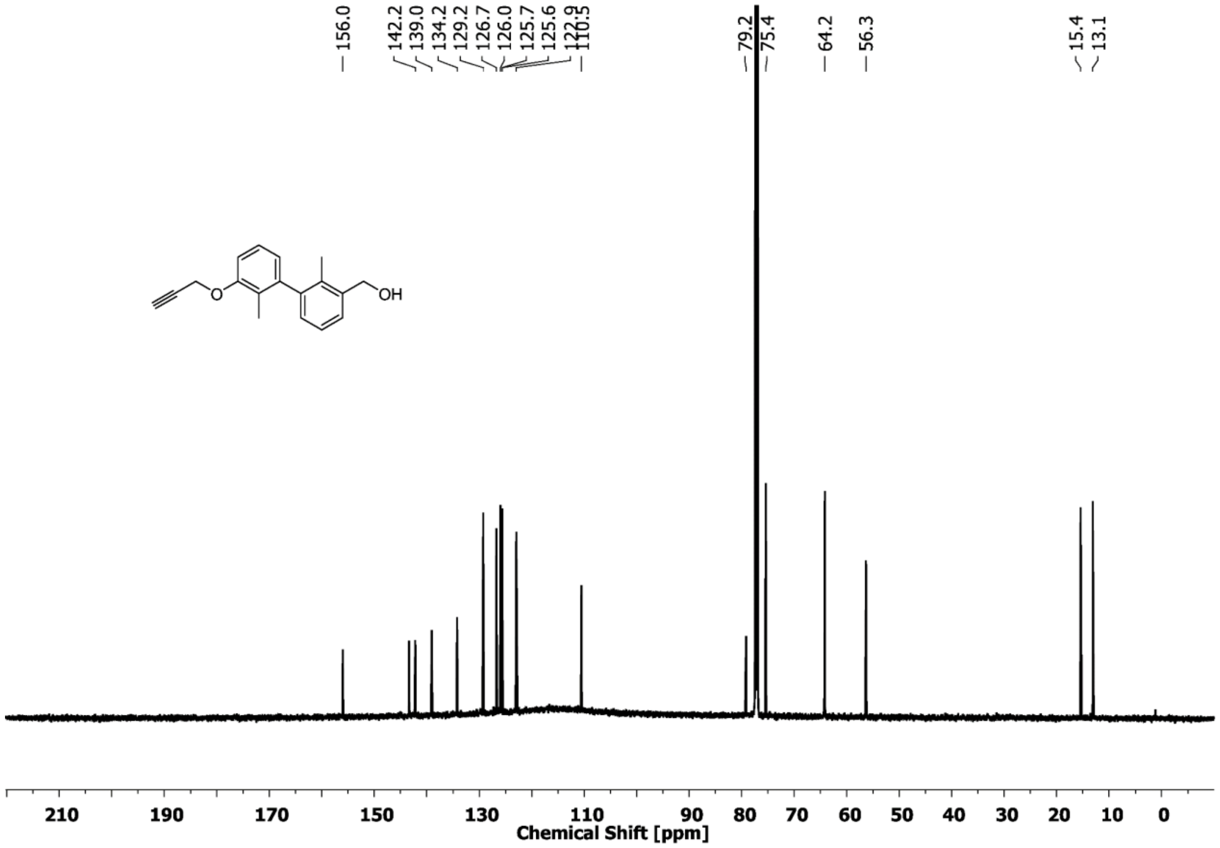

Figure S26: ^{13}C NMR spectrum ($CDCl_3$, 151 MHz, 298 K) of compound 28b.

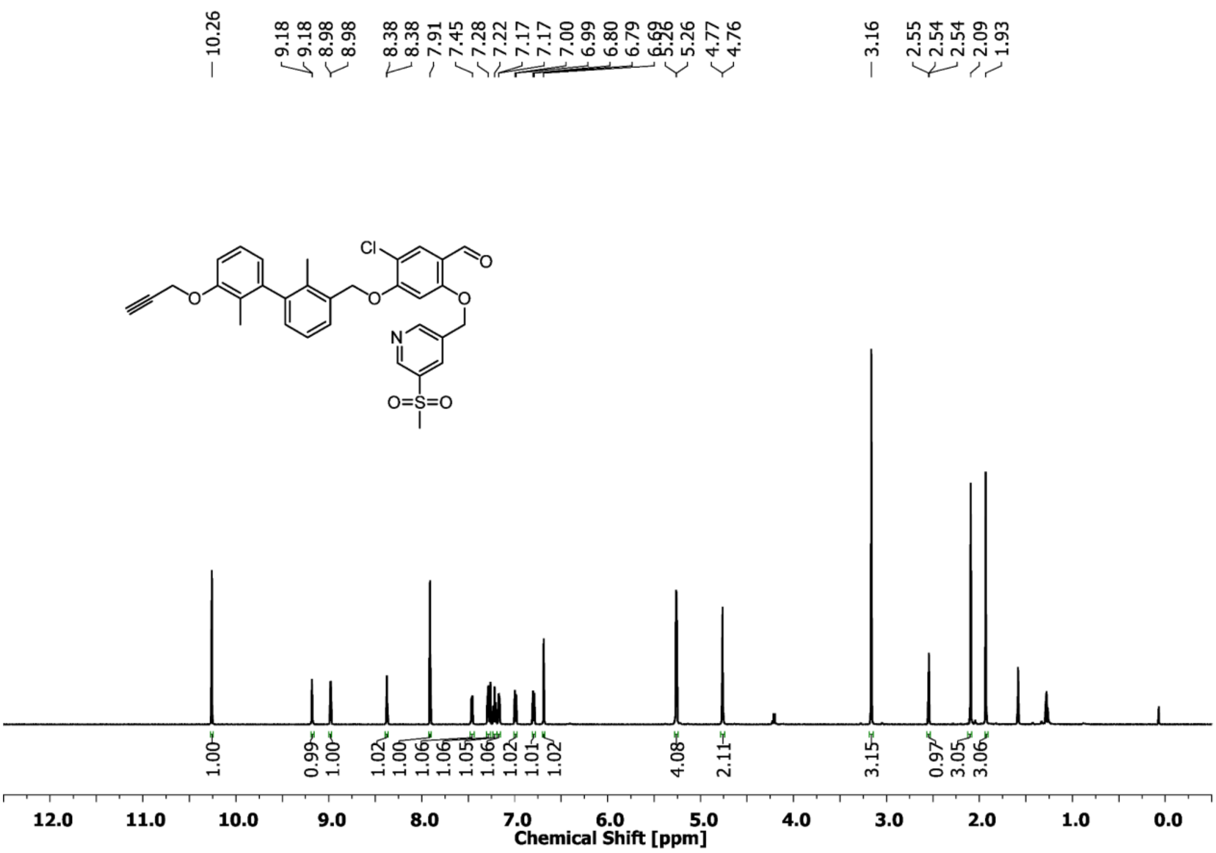

Figure S27: ^{1}H NMR spectrum ($CDCl_3$, 600 MHz, 298 K) of compound 29b.

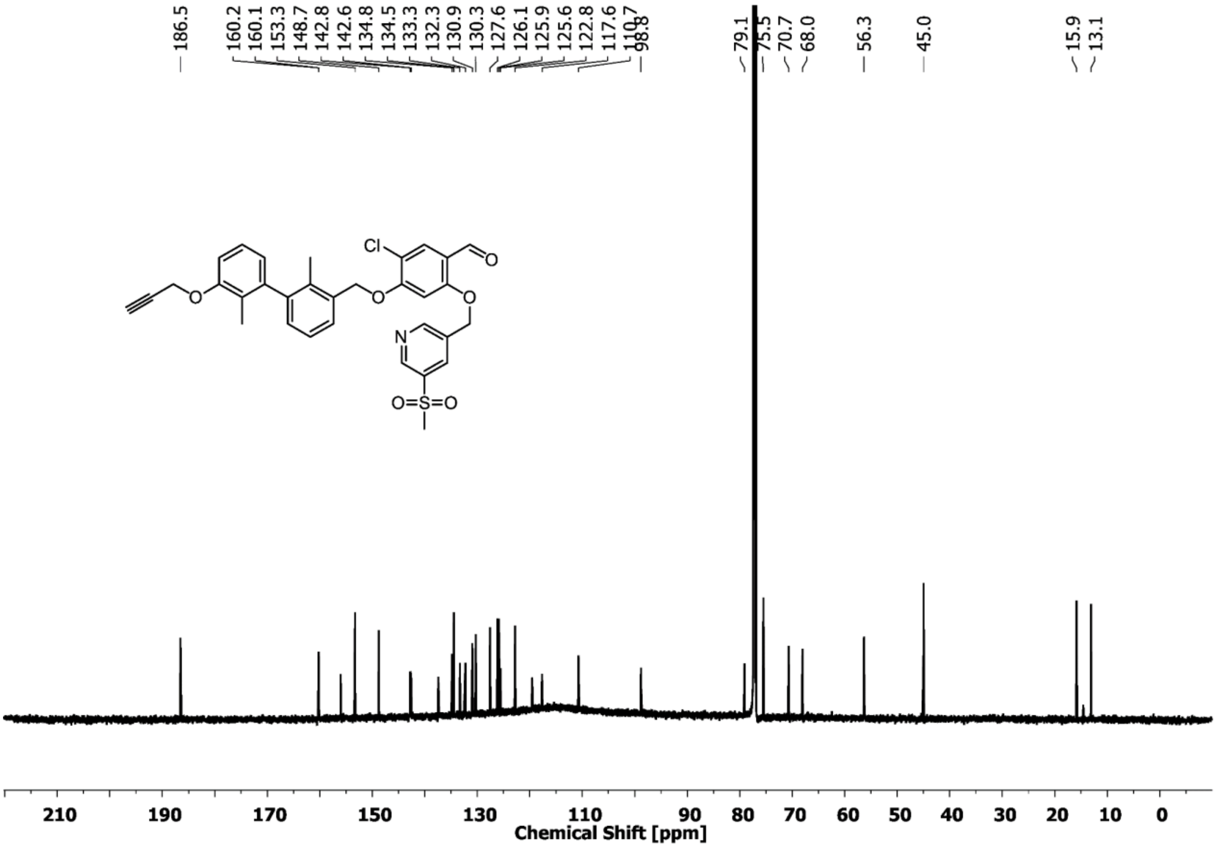

Figure S28: ^{13}C NMR spectrum ($CDCl_3$, 151 MHz, 298 K) of compound 29b.

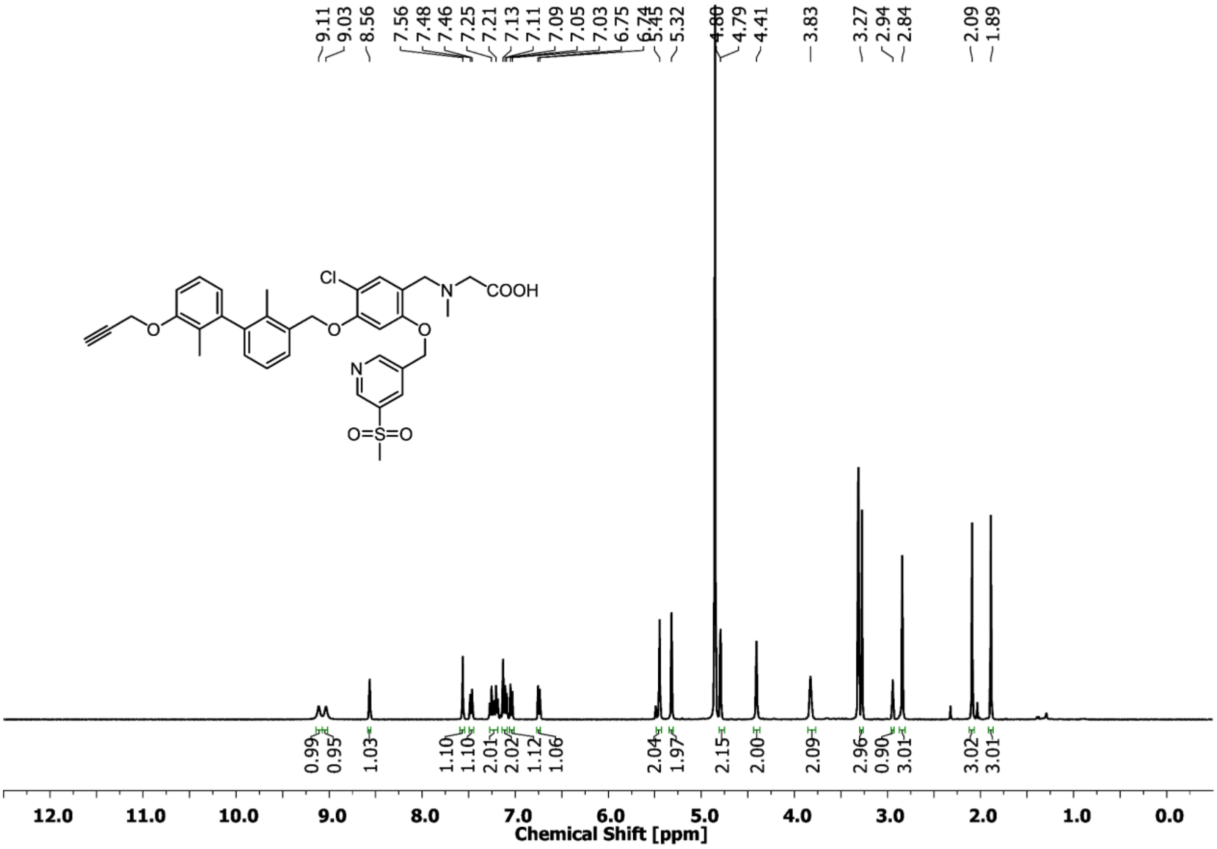

Figure S29: ^{1}H NMR spectrum (methanol-d_4, 400 MHz, 298 K) of compound 30b.

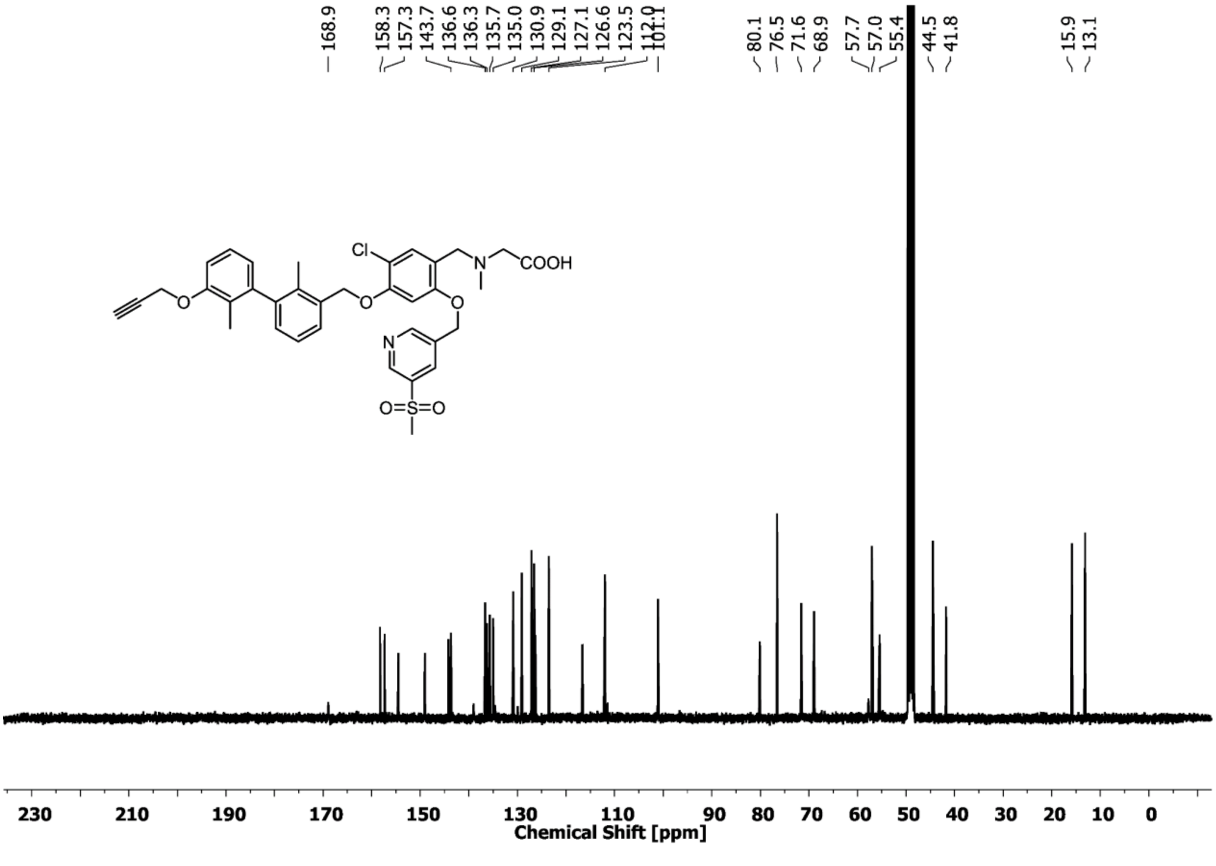

Figure S30: ^{13}C NMR spectrum (methanol-d_4, 101 MHz, 298 K) of compound 30b.

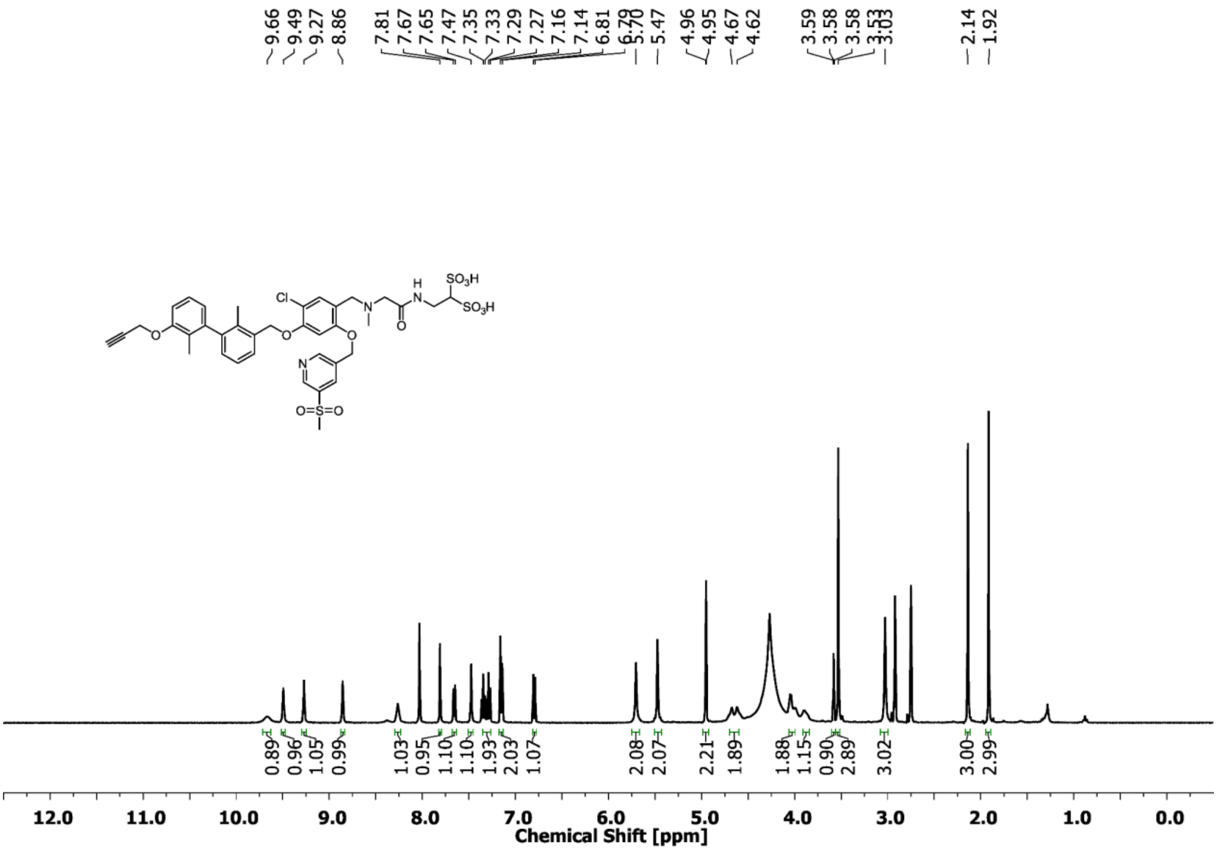

Figure S31: ^{1}H NMR spectrum (DMF-d_7, 400 MHz, 298 K) of compound 33b.

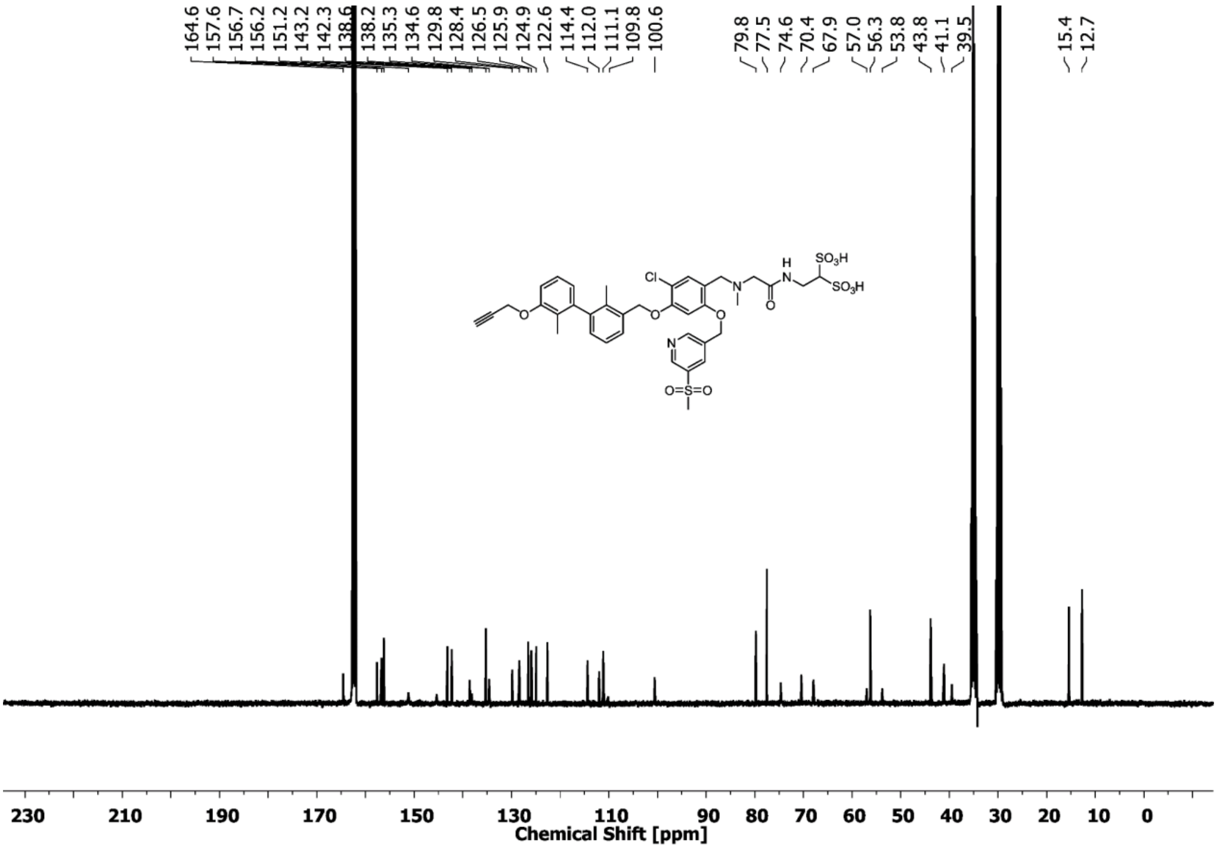

Figure S32: ^{13}C NMR spectrum (DMF-d_7, 101 MHz, 298 K) of compound 33b.

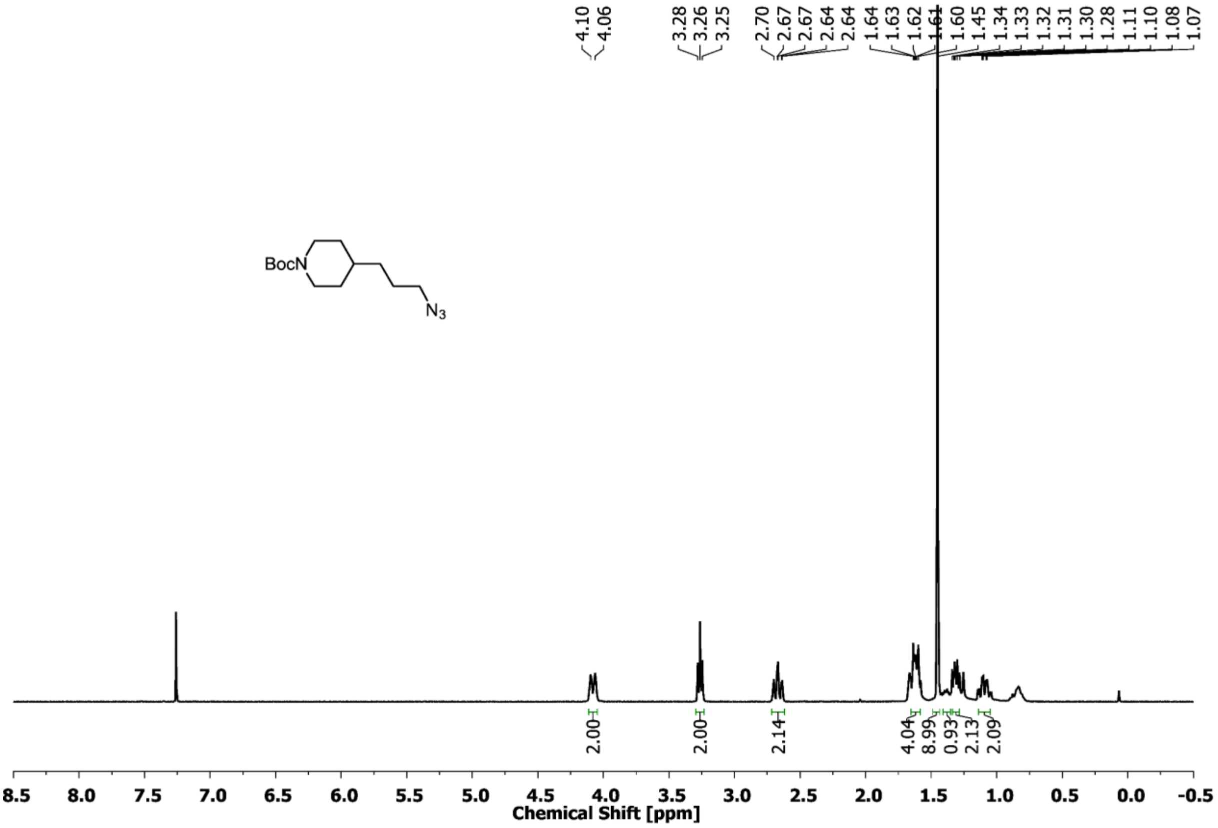

Figure S33: ^{1}H NMR spectrum ($CDCl_3$, 400 MHz, 298 K) of compound S-5.

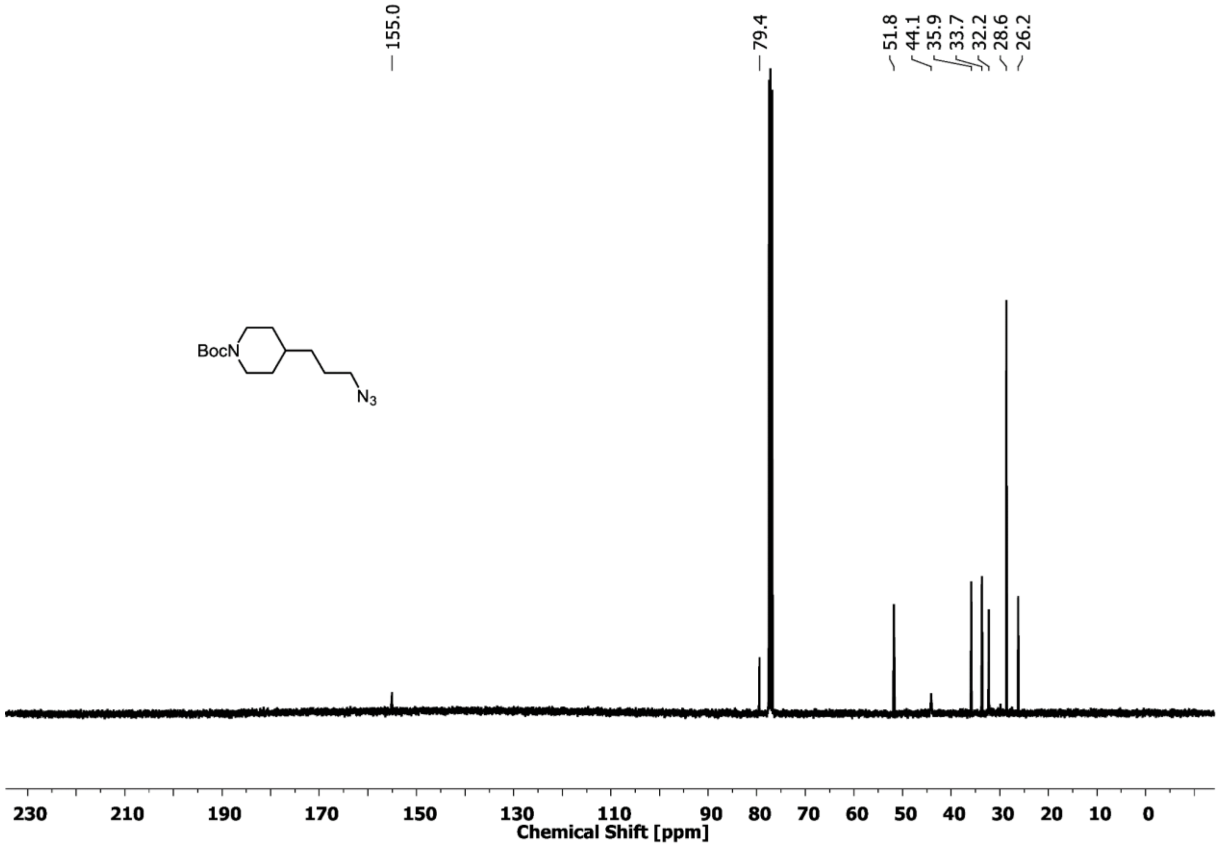

Figure S34: ^{13}C NMR spectrum ($CDCl_3$, 101 MHz, 298 K) of compound S-5.

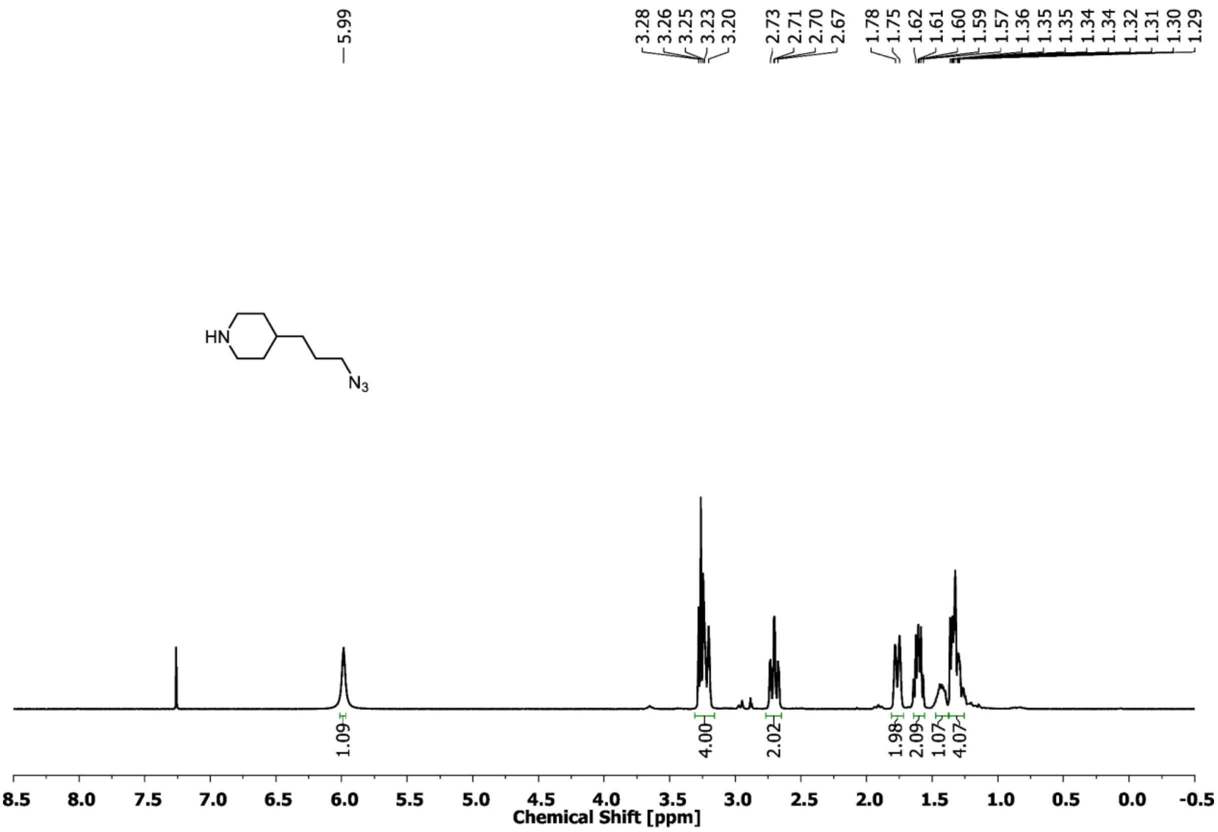

Figure S35: ^{1}H NMR spectrum (CDCl$_3$, 400 MHz, 298 K) of compound 35.

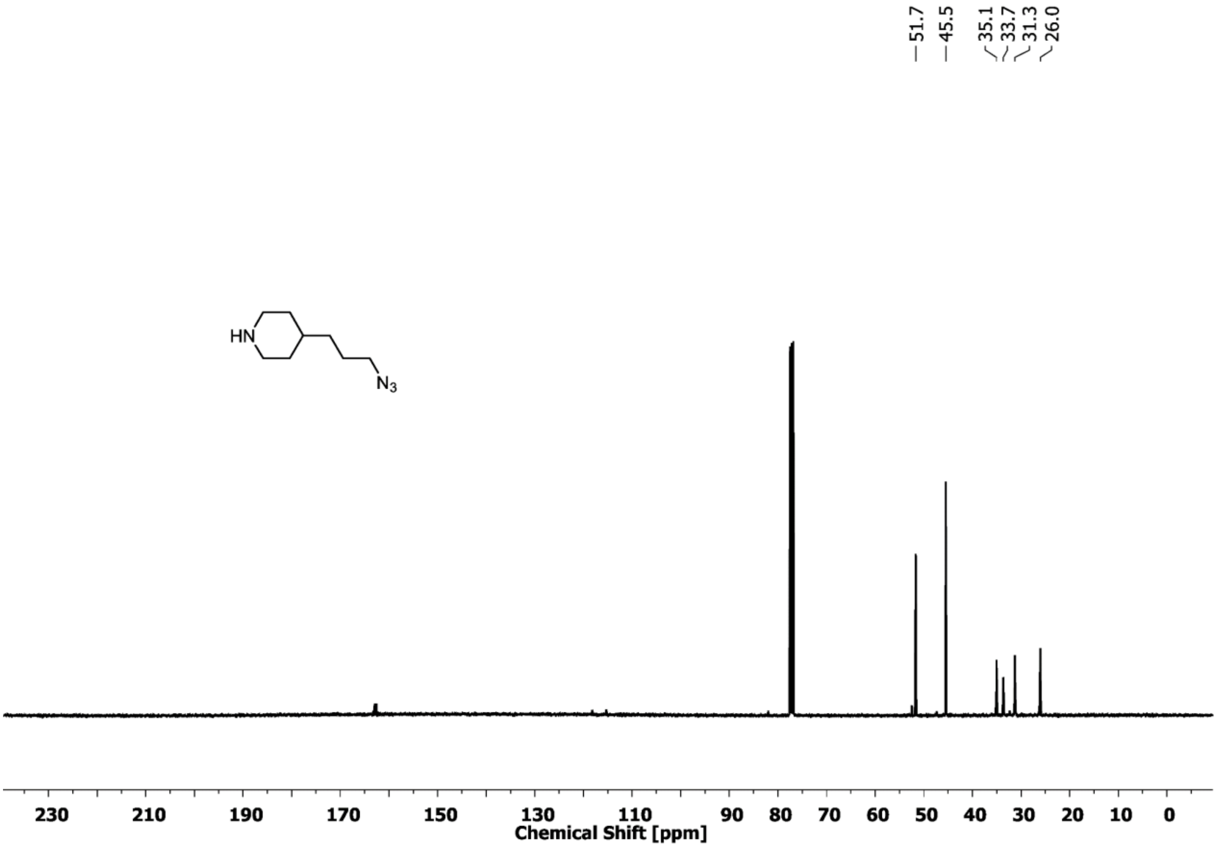

Figure S36: ^{13}C NMR spectrum (CDCl$_3$, 101 MHz, 298 K) of compound 35.

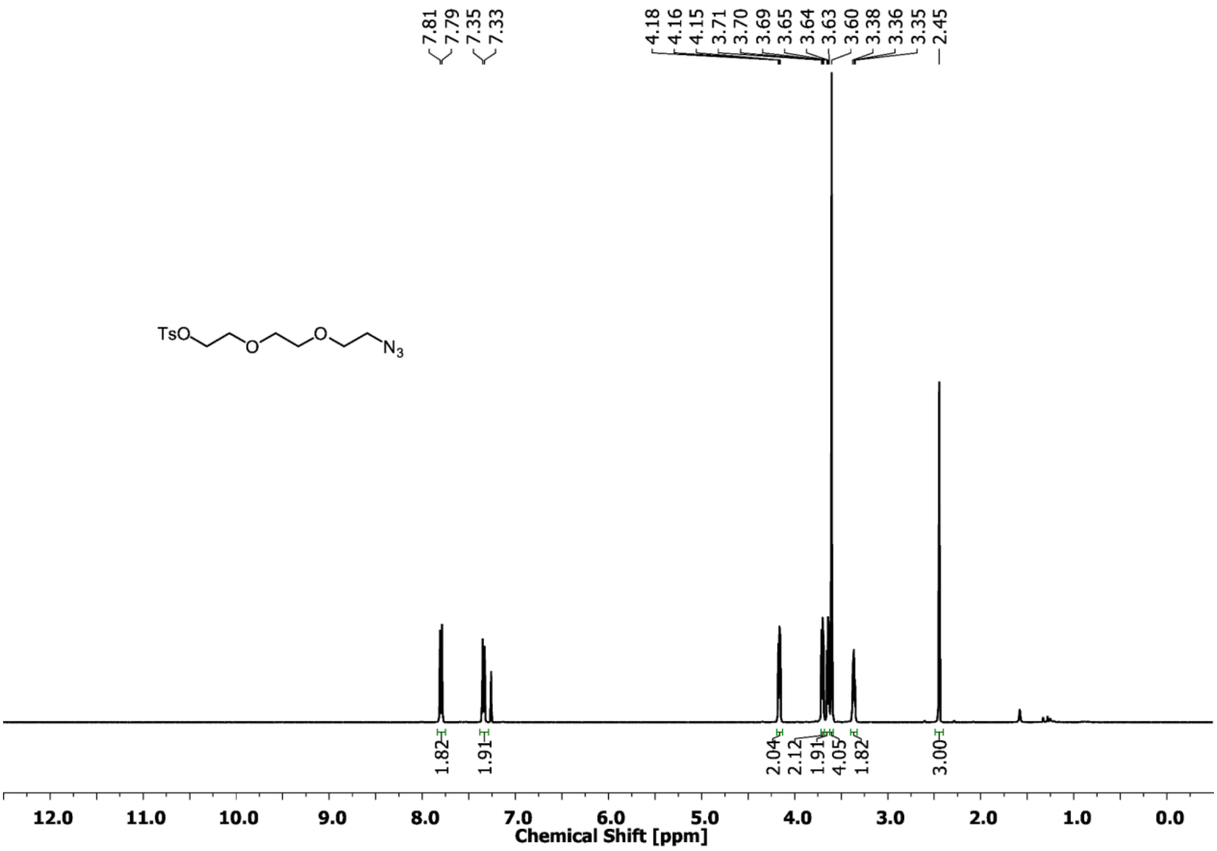

Figure S37: ^{1}H NMR spectrum ($CDCl_3$, 400 MHz, 298 K) of compound S-6.

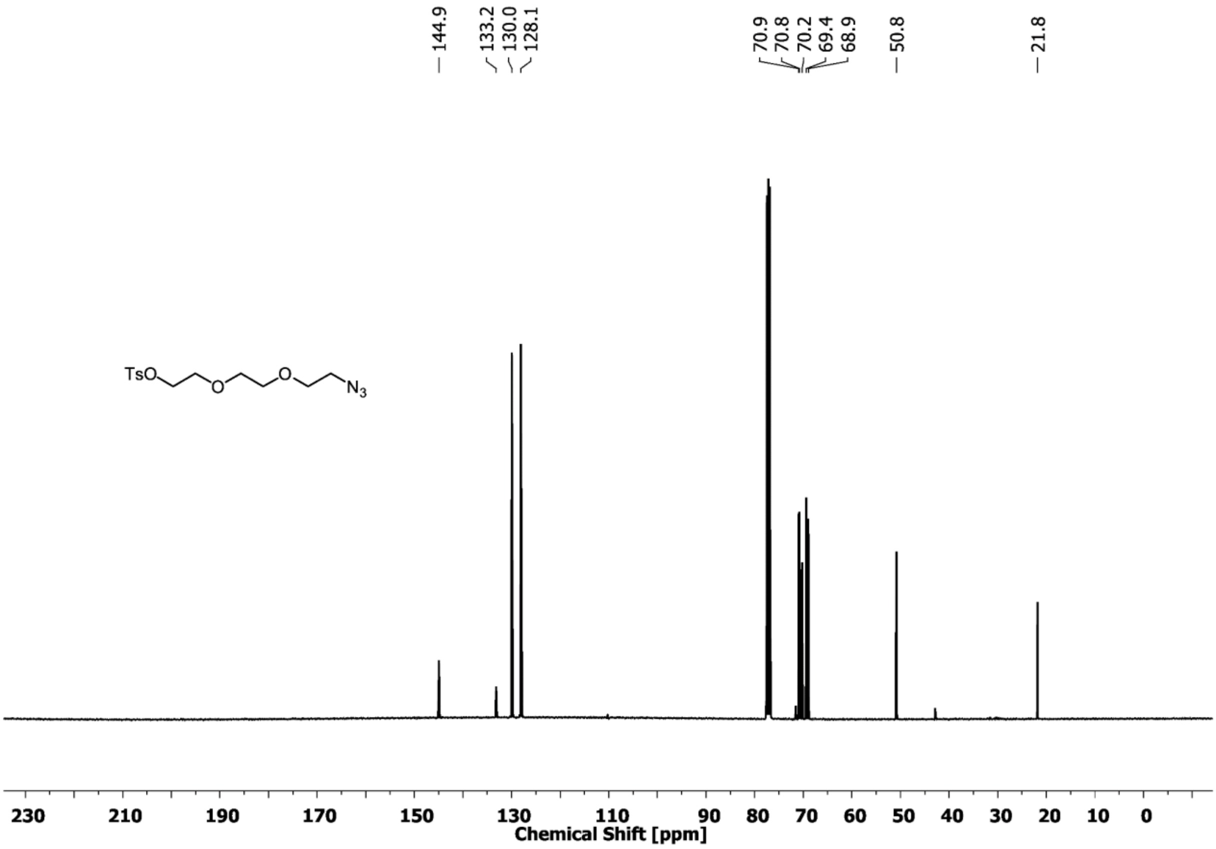

Figure S38: ^{13}C NMR spectrum ($CDCl_3$, 101 MHz, 298 K) of compound S-6.

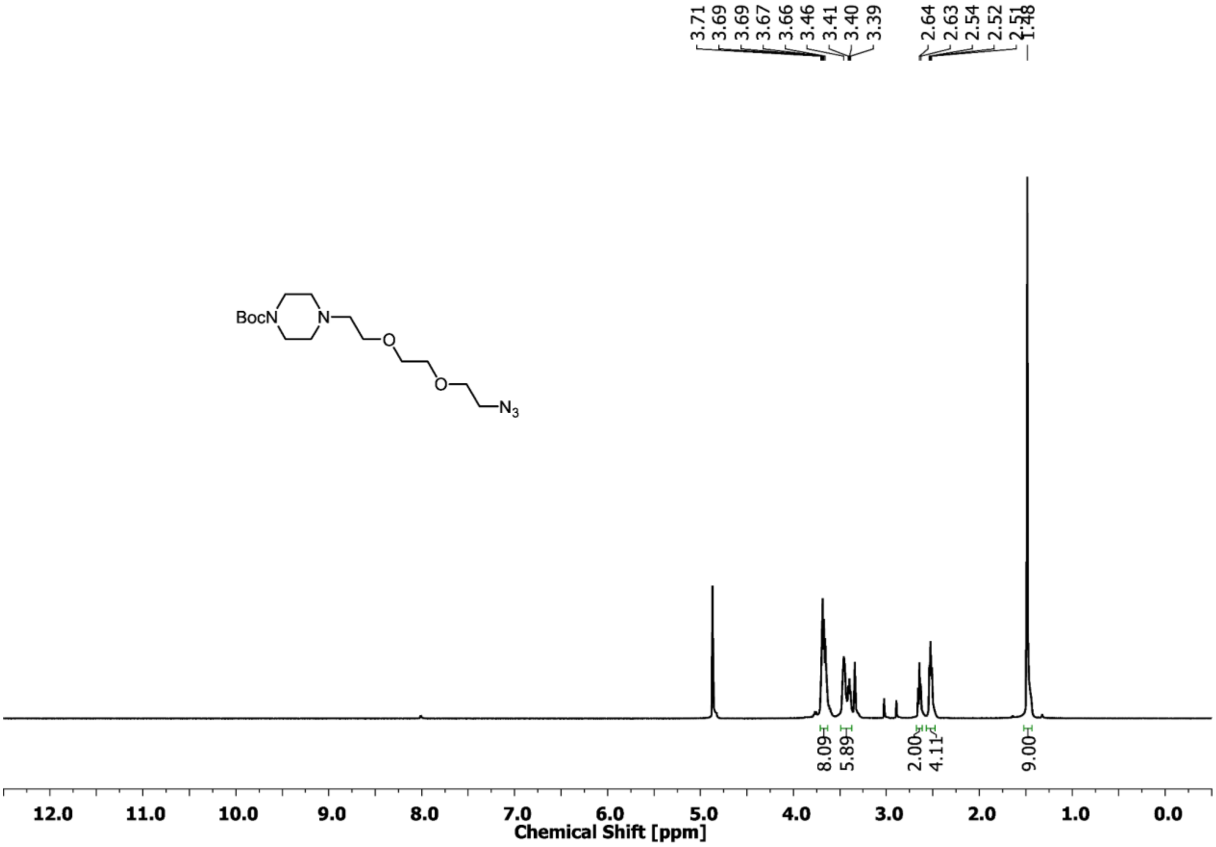

Figure S39: ^{1}H NMR spectrum (methanol-d_4, 400 MHz, 298 K) of compound S-7.

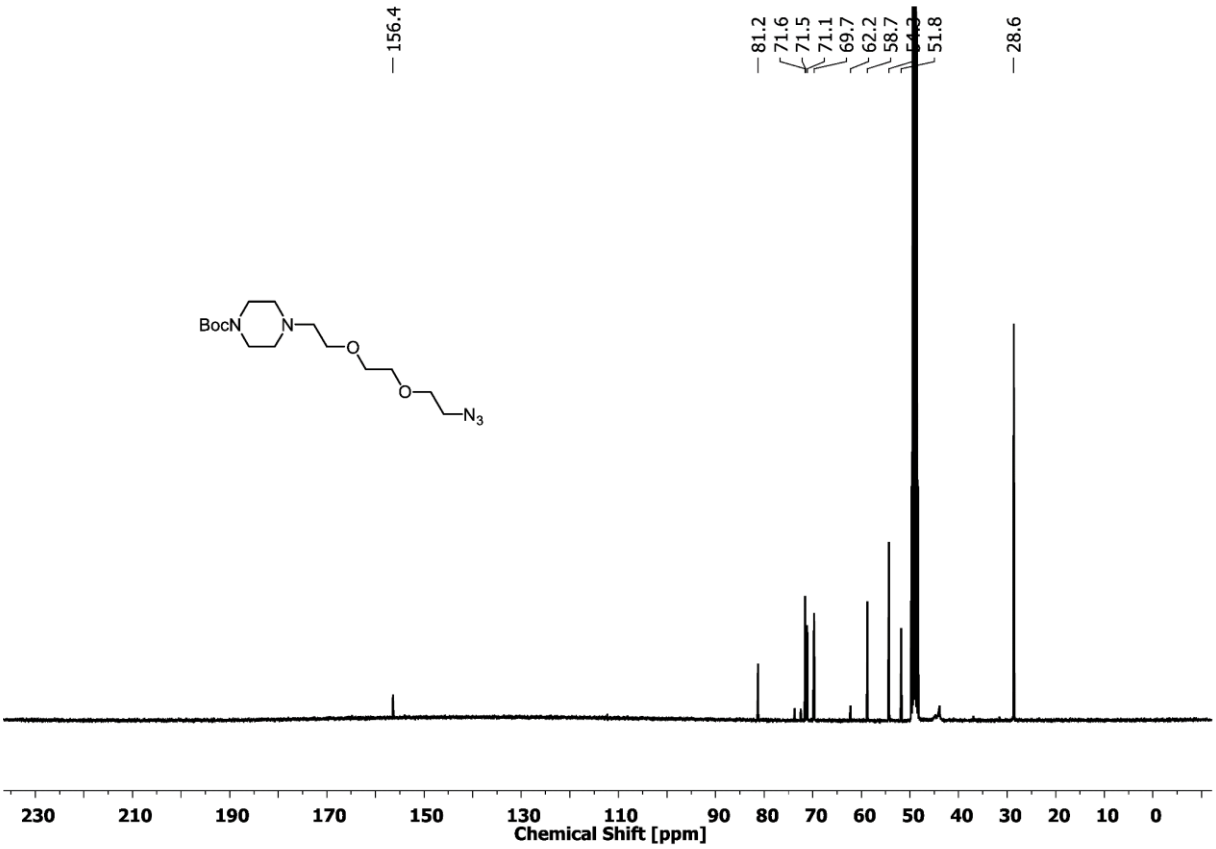

Figure S40: ^{13}C NMR spectrum (methanol-d_4, 101 MHz, 298 K) of compound S-7.

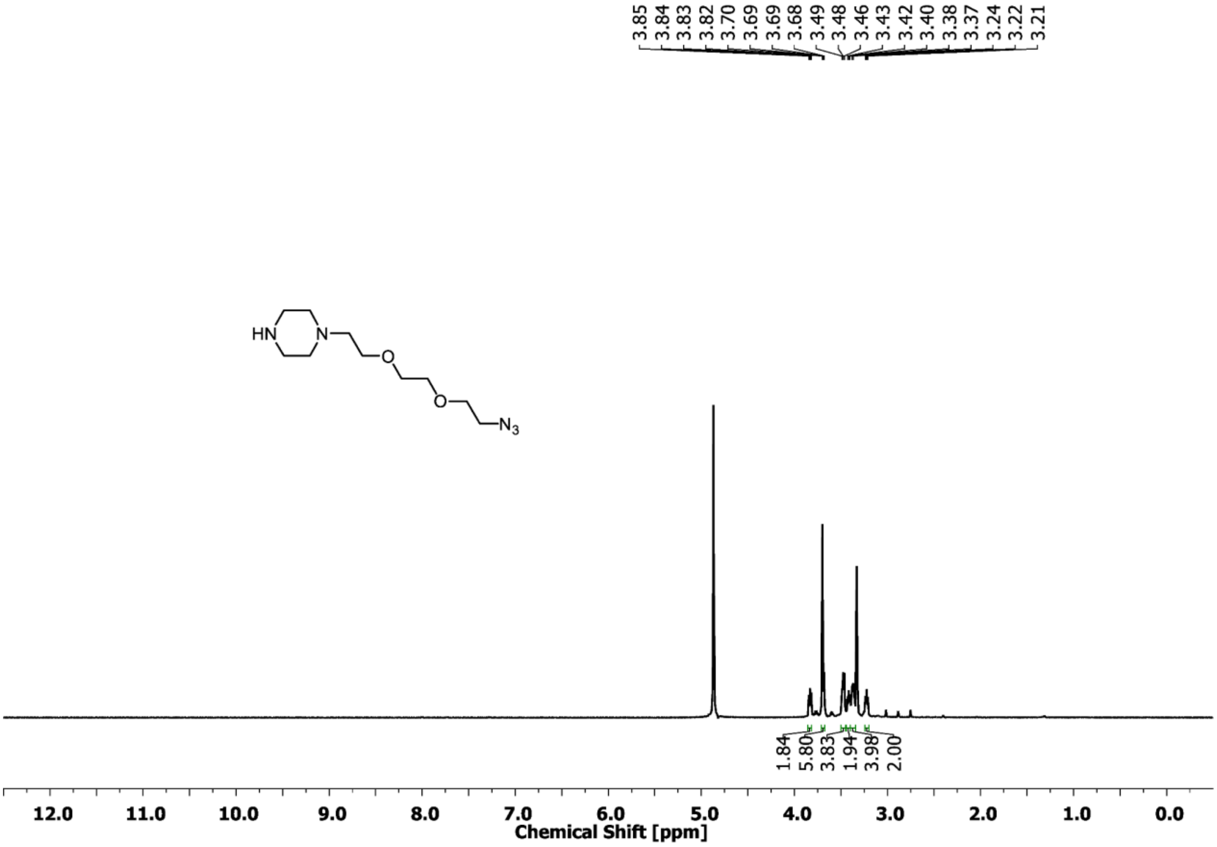

Figure S41: ^{1}H NMR spectrum (methanol-*d*$_4$, 400 MHz, 298 K) of compound 39.

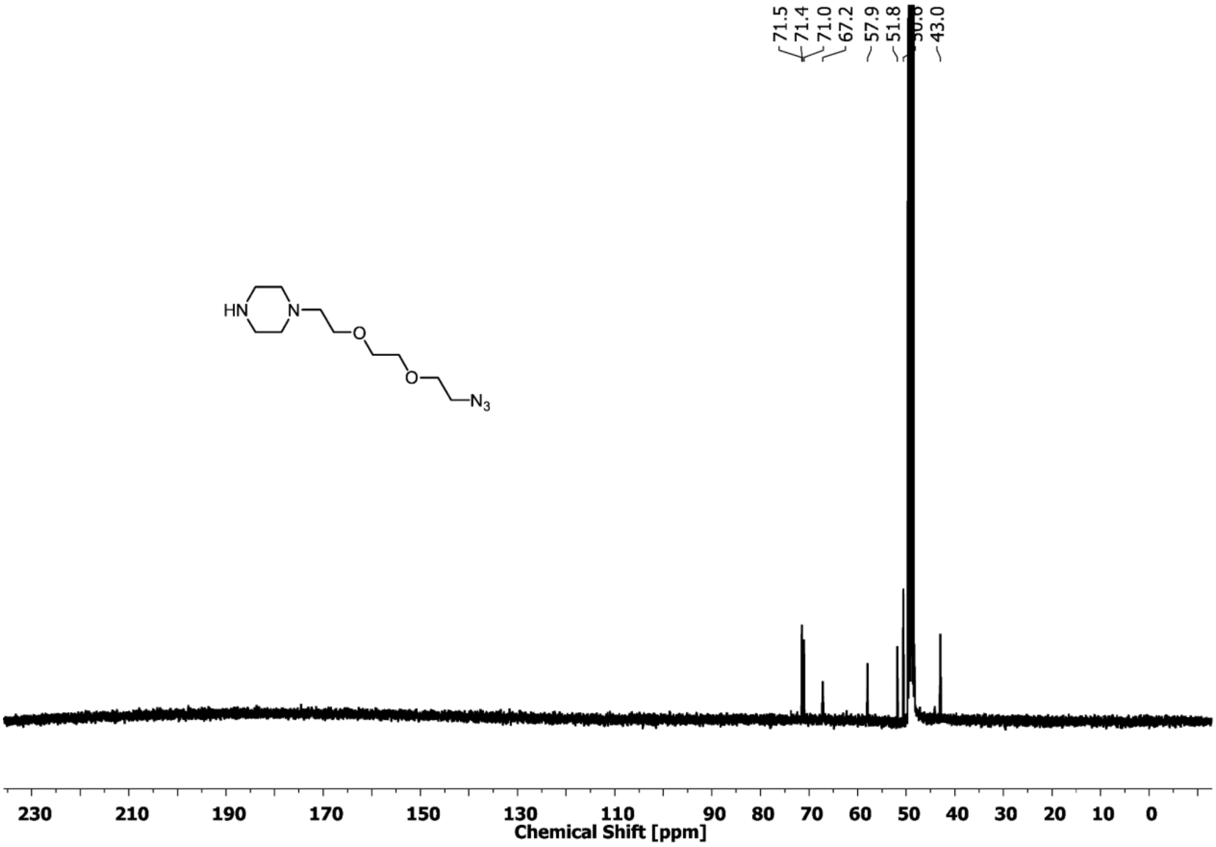

Figure S42: ^{13}C NMR spectrum (methanol-*d*$_4$, 101 MHz, 298 K) of compound 39.

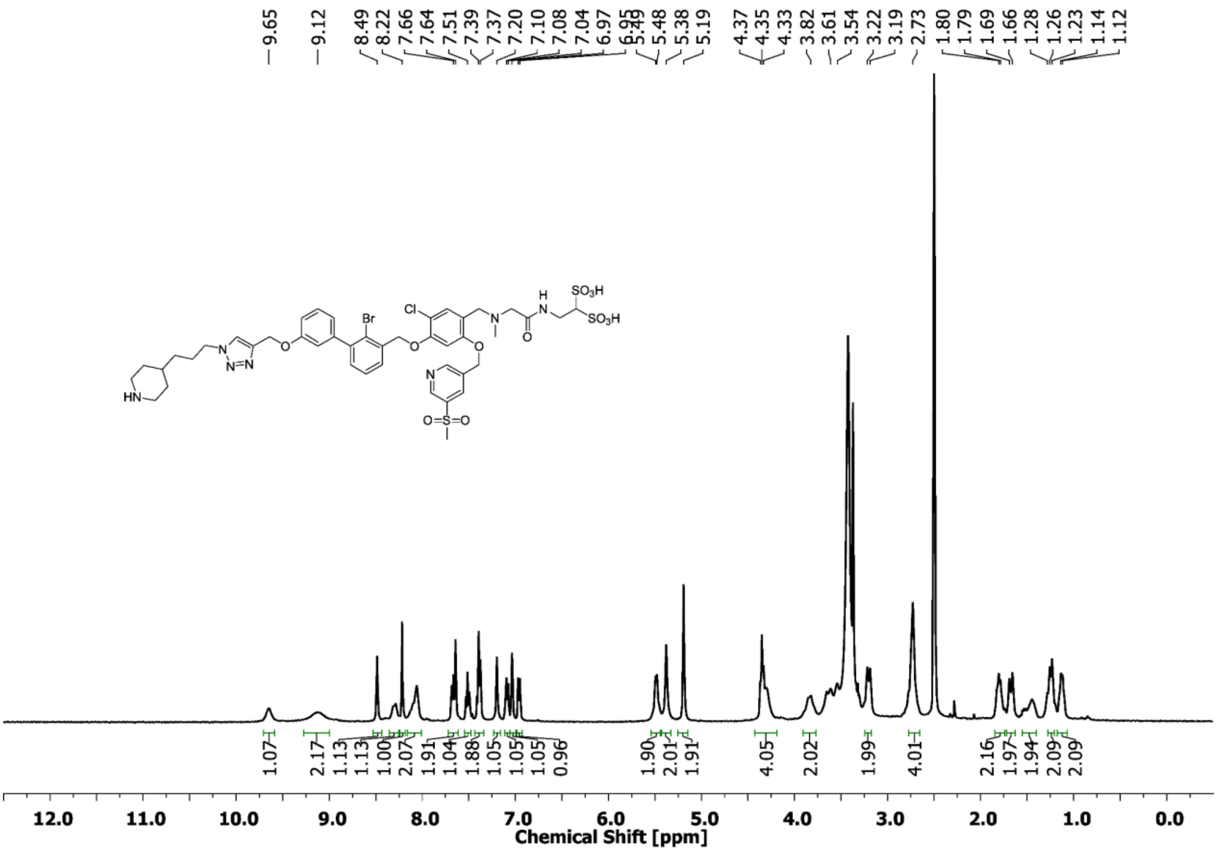

Figure S43: ^{1}H NMR spectrum (DMSO-*d*$_6$, 400 MHz, 298 K) of compound 40a.

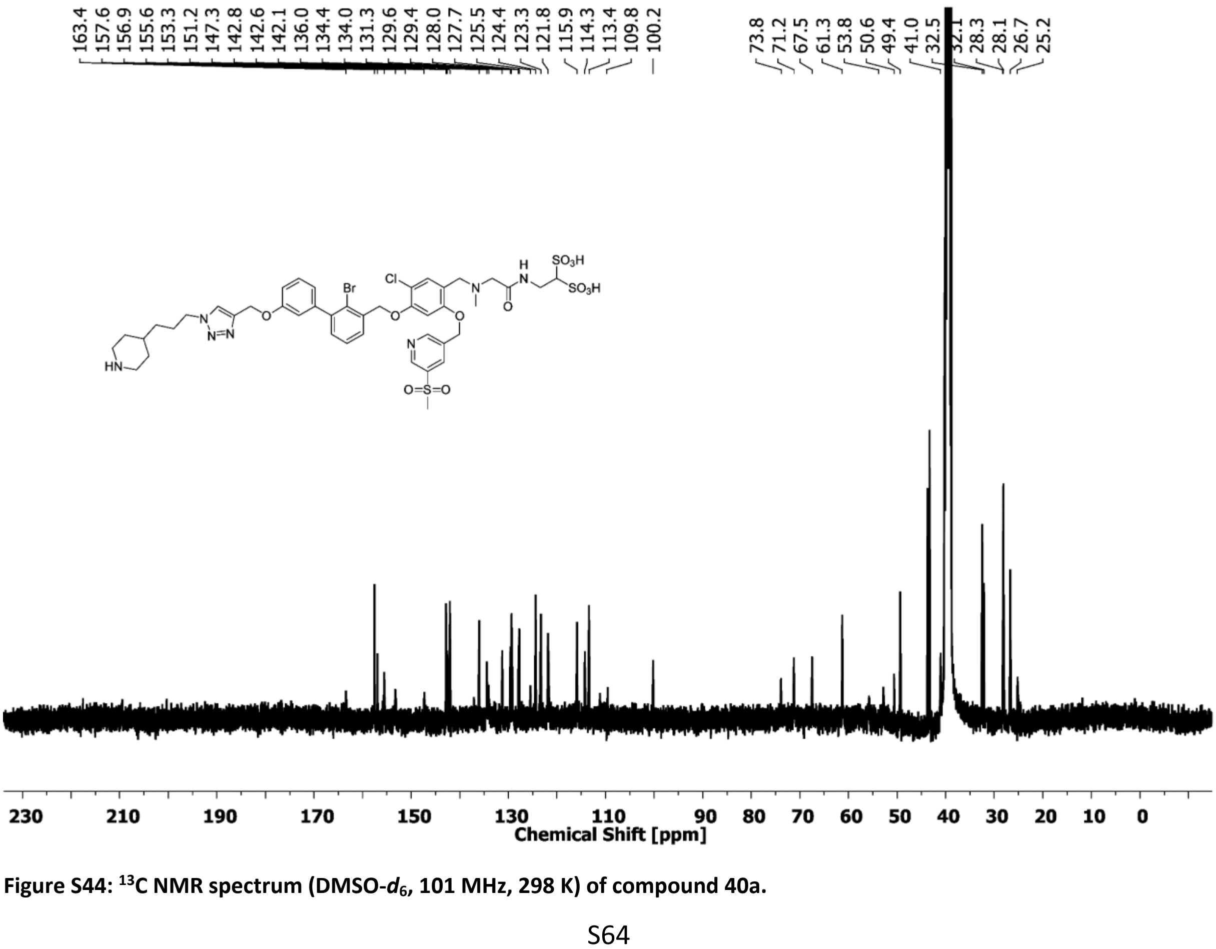

Figure S44: ^{13}C NMR spectrum (DMSO-*d*$_6$, 101 MHz, 298 K) of compound 40a.

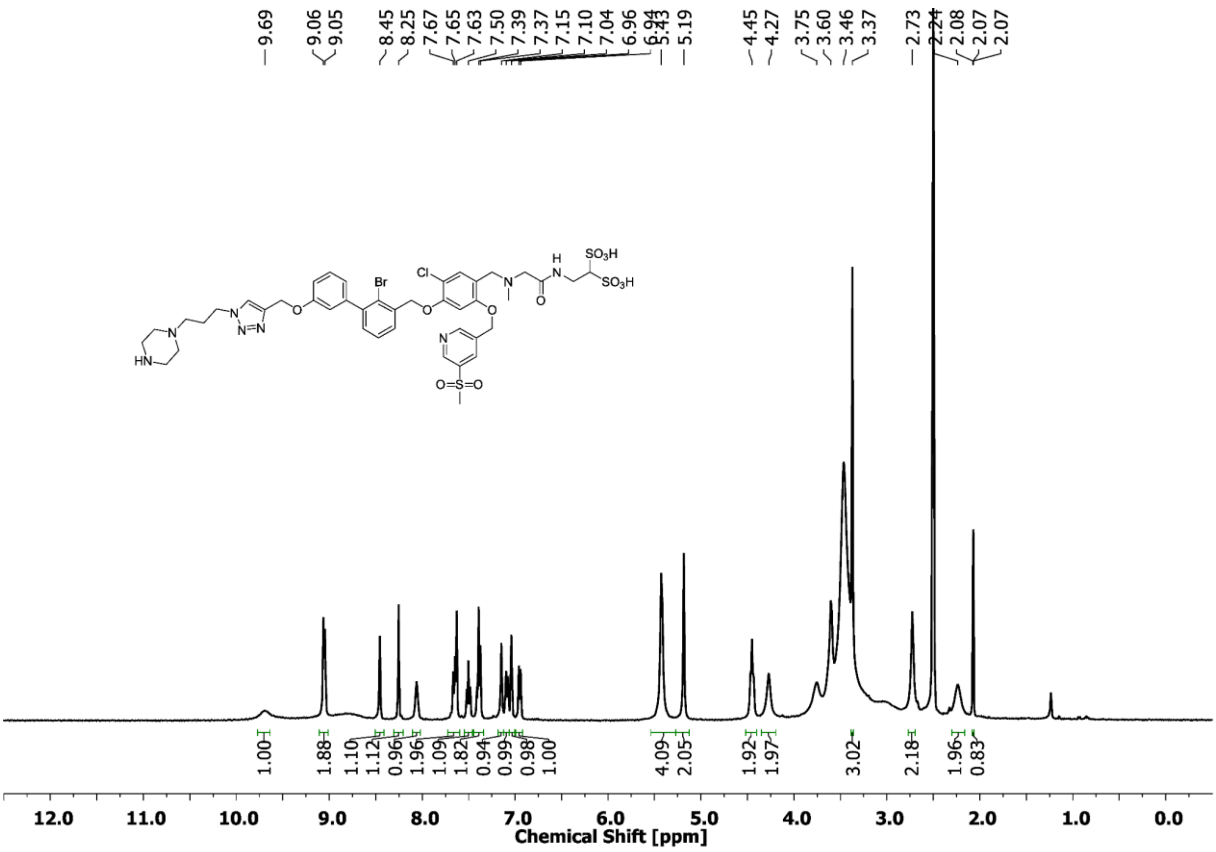

Figure S45: ^{1}H NMR spectrum (DMSO-d_6, 400 MHz, 298 K) of compound 40b.

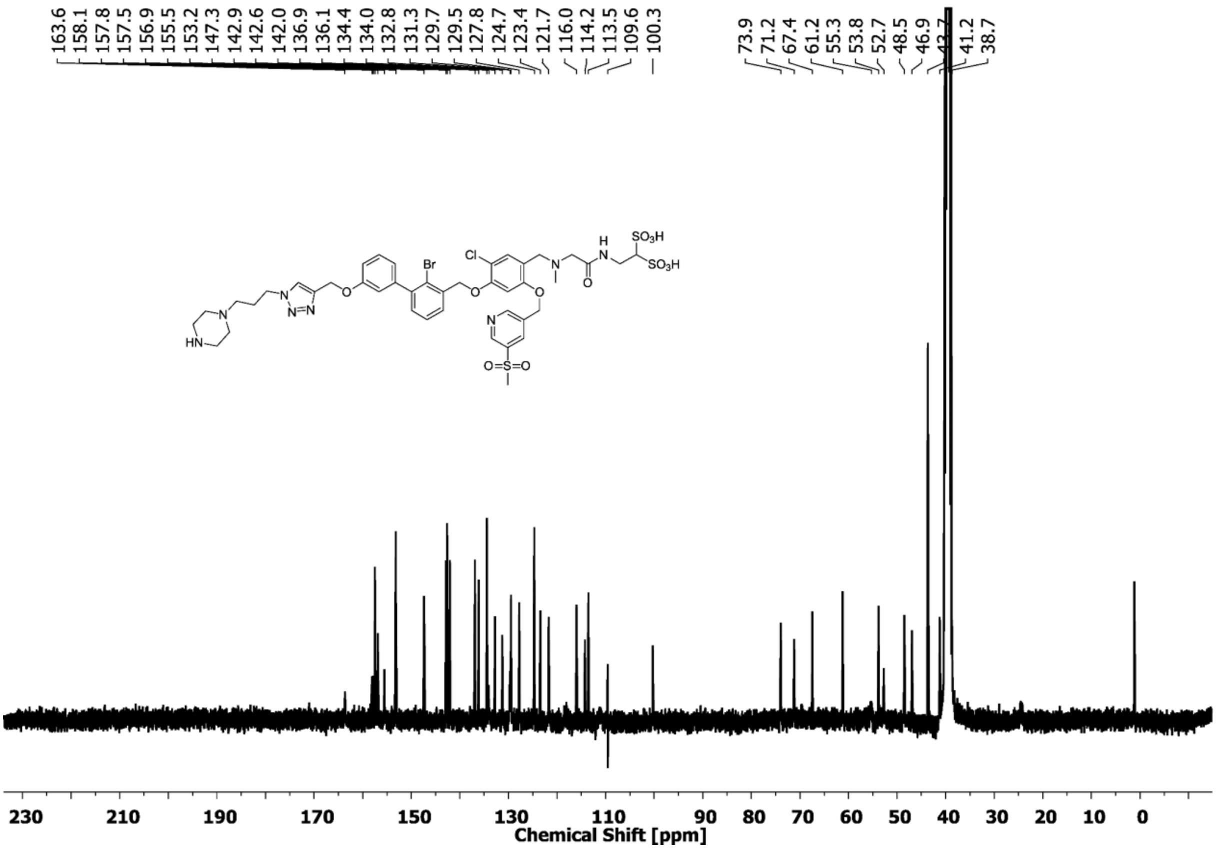

Figure S46: ^{13}C NMR spectrum (DMSO-d_6, 101 MHz, 298 K) of compound 40b.

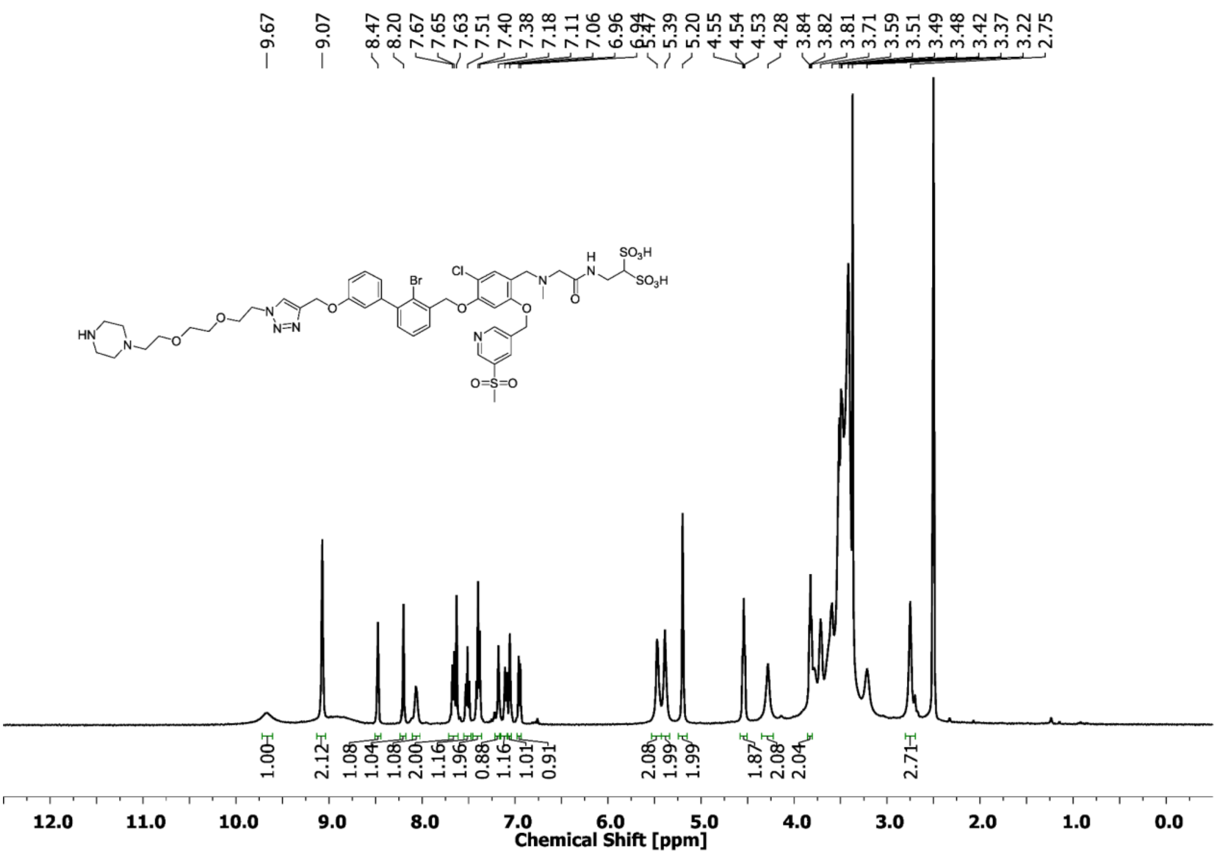

Figure S47: ^{1}H NMR spectrum (DMSO-d_6, 400 MHz, 298 K) of compound 40c.

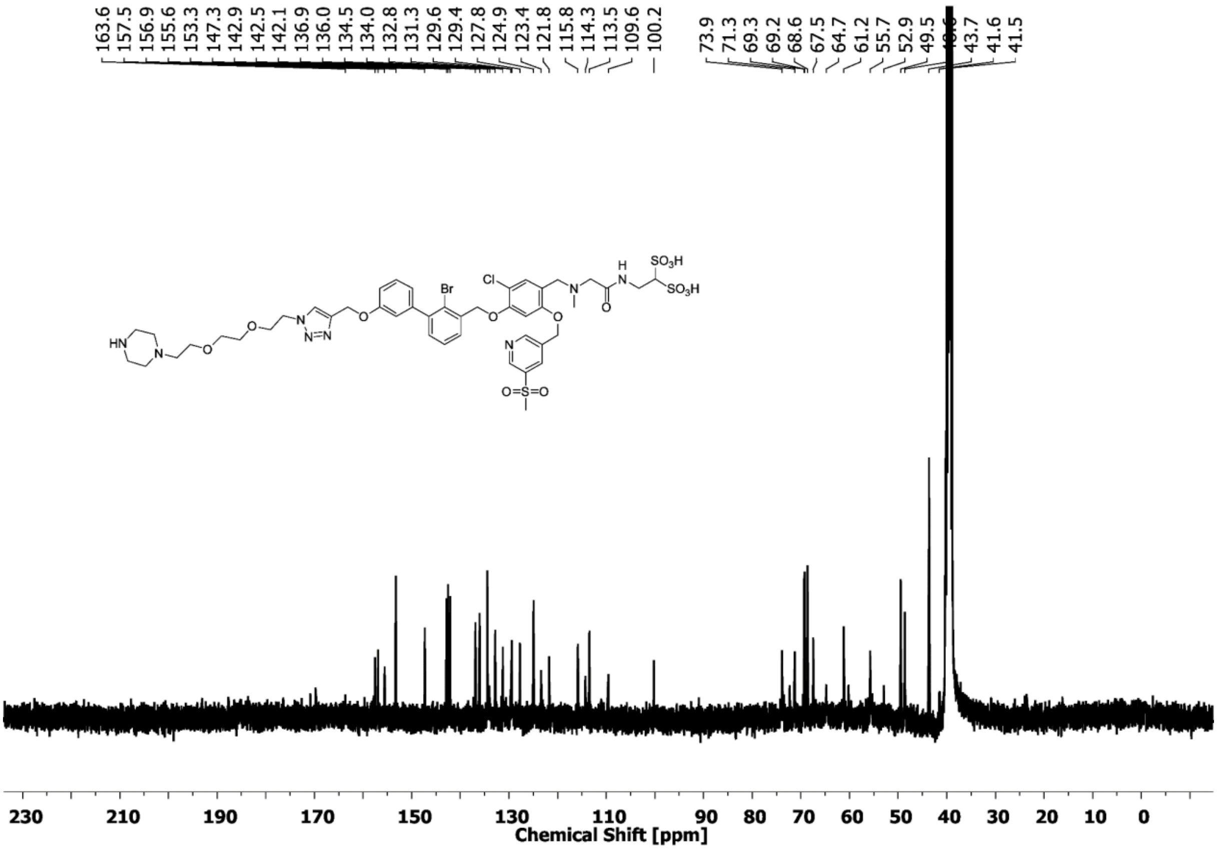

Figure S48: ^{13}C NMR spectrum (DMSO-d_6, 101 MHz, 298 K) of compound 40c.

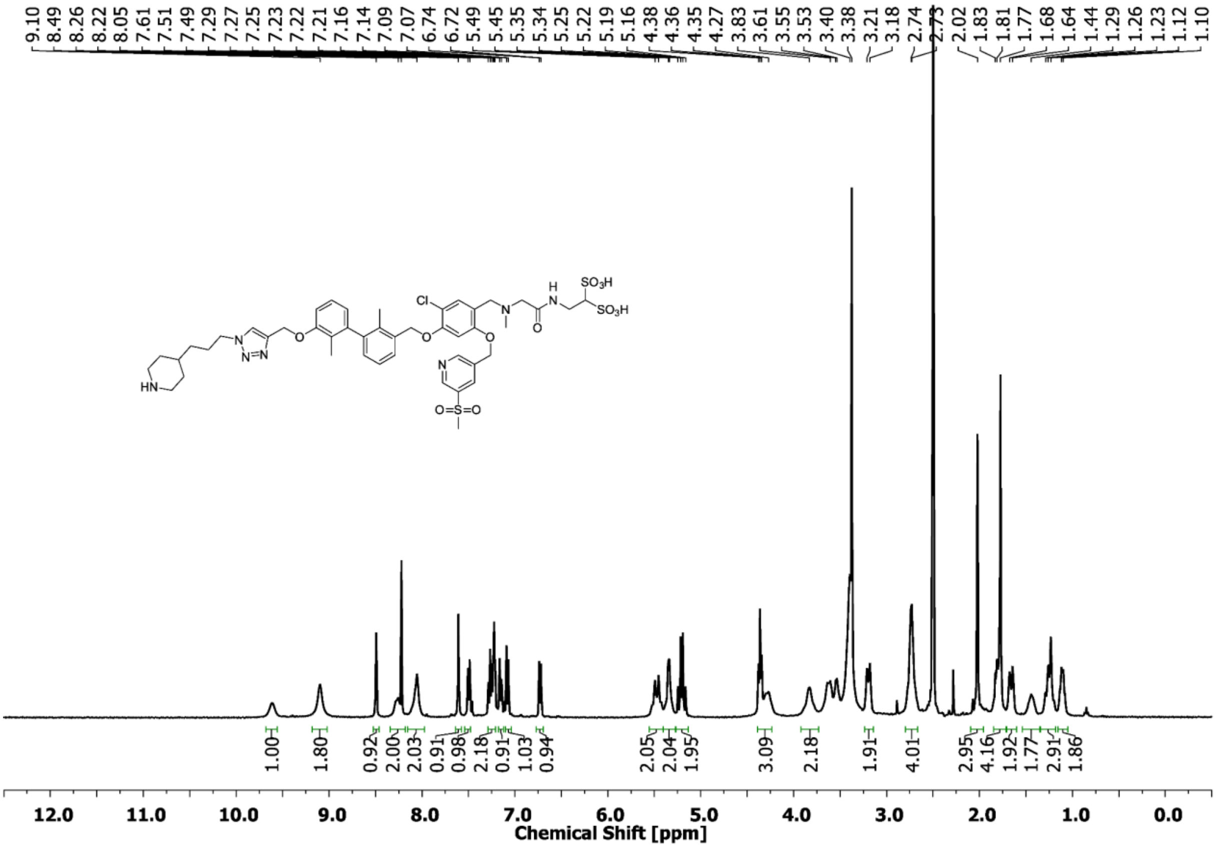

Figure S49: ^{1}H NMR spectrum (DMSO-d_6, 400 MHz, 298 K) of compound 41a.

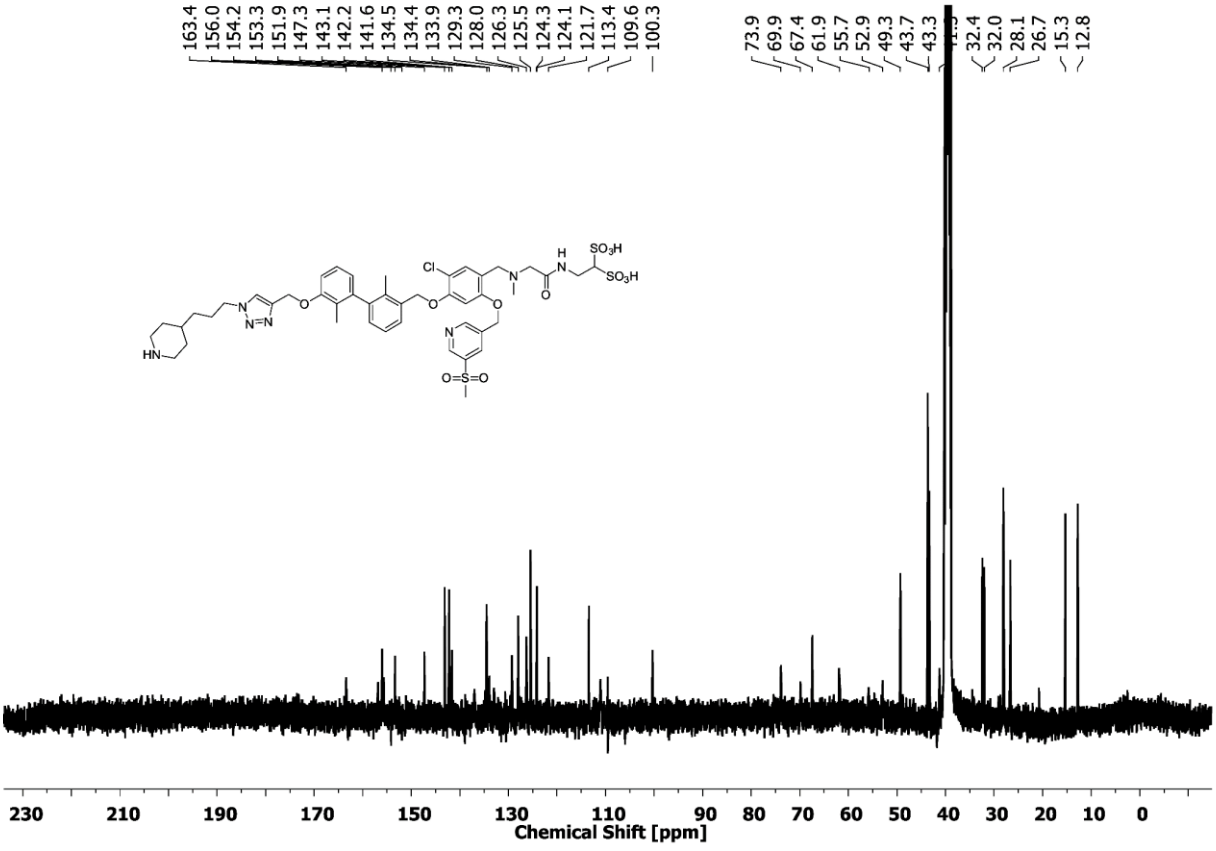

Figure S50: ^{13}C NMR spectrum (DMSO-d_6, 101 MHz, 298 K) of compound 41a.

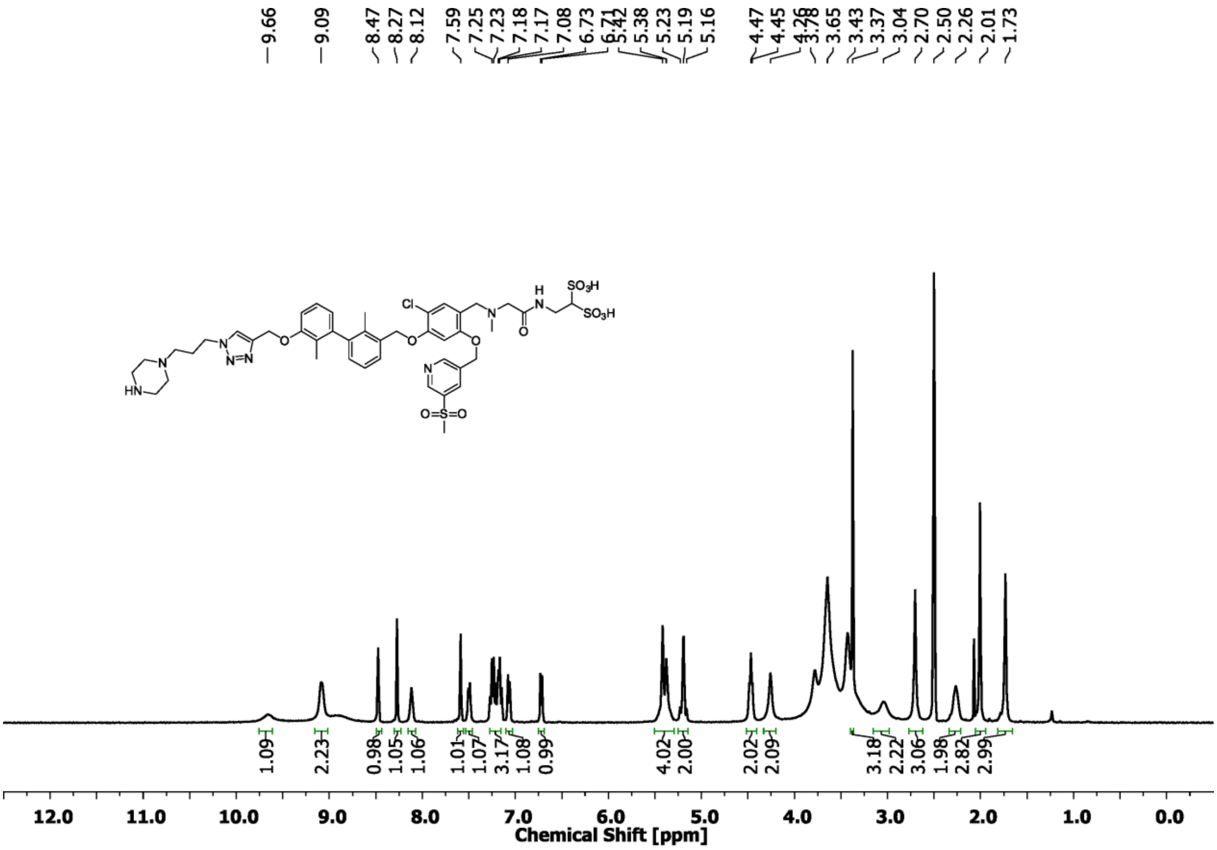

Figure S51: ^{1}H NMR spectrum (DMSO-d_6, 400 MHz, 298 K) of compound 41b.

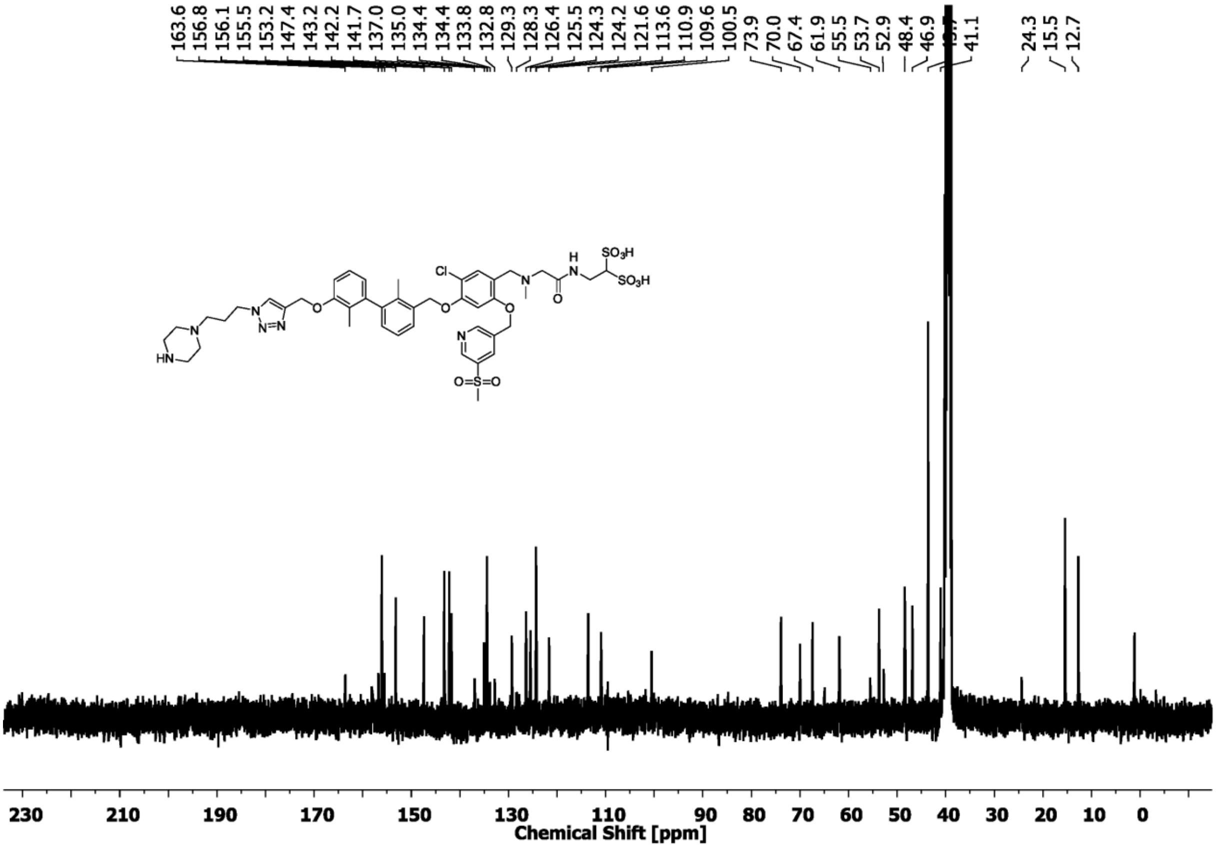

Figure S52: ^{13}C NMR spectrum (DMSO-d_6, 101 MHz, 298 K) of compound 41b.

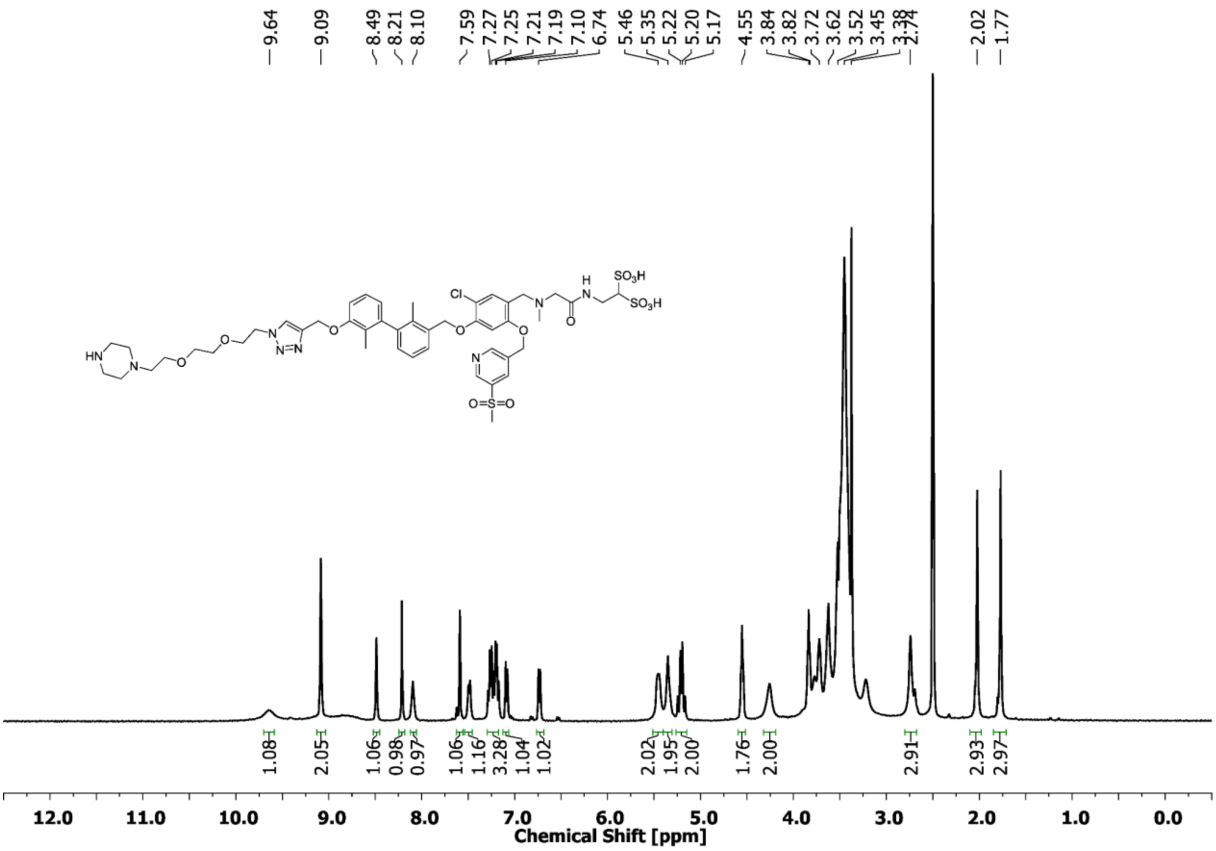

Figure S53: ^{1}H NMR spectrum (DMSO-d_6, 400 MHz, 298 K) of compound 41c.

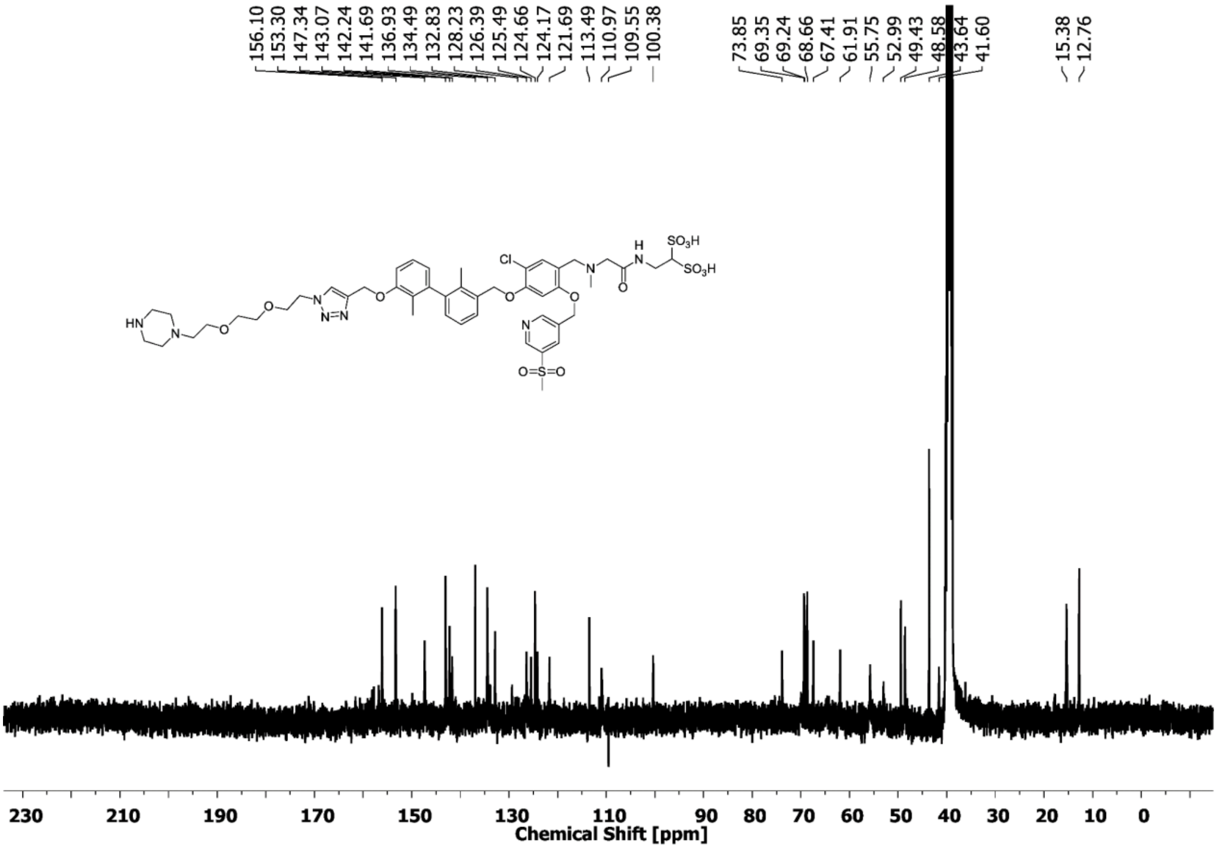

Figure S54: ^{13}C NMR spectrum (DMSO-d_6, 101 MHz, 298 K) of compound 41c.

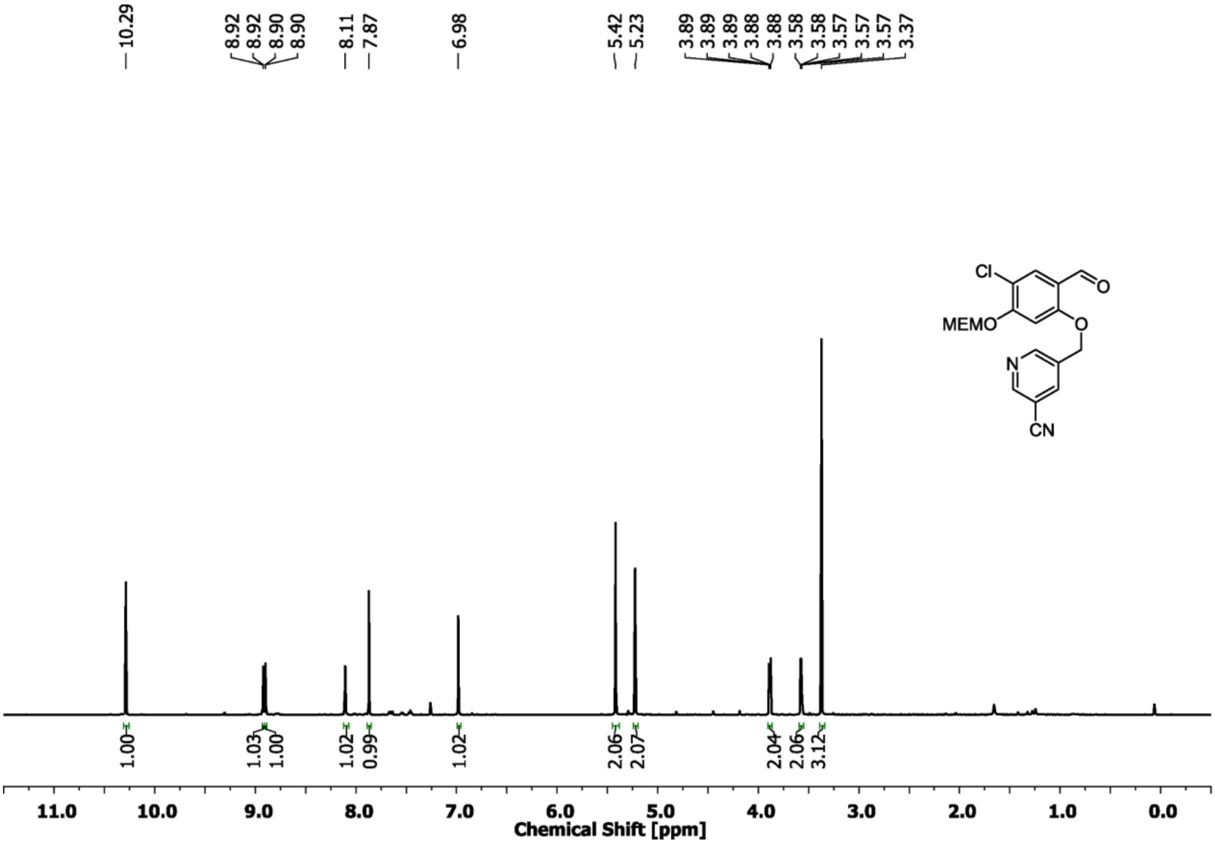

Figure S55: ^{1}H NMR spectrum ($CDCl_3$, 600 MHz, 298 K) of compound 46a.

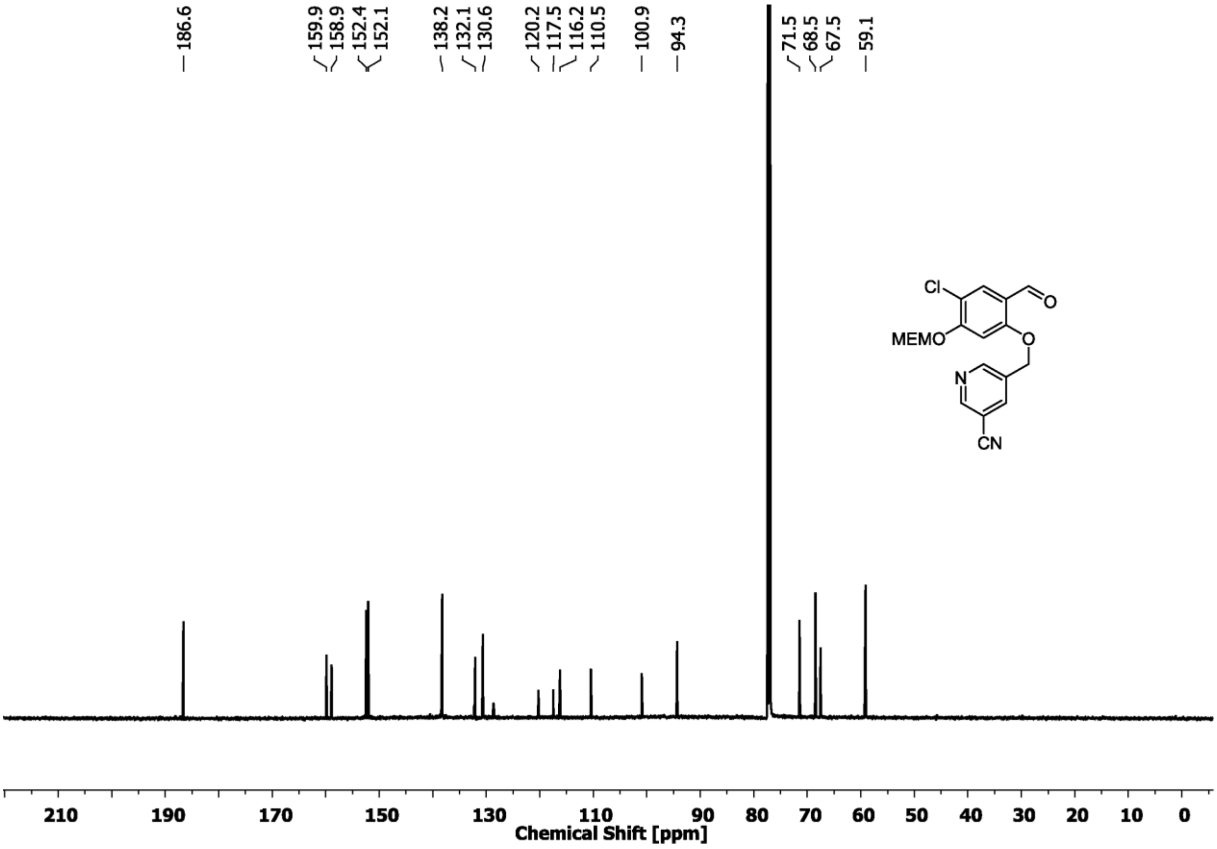

Figure S56: ^{13}C NMR spectrum ($CDCl_3$, 151 MHz, 298 K) of compound 46a.

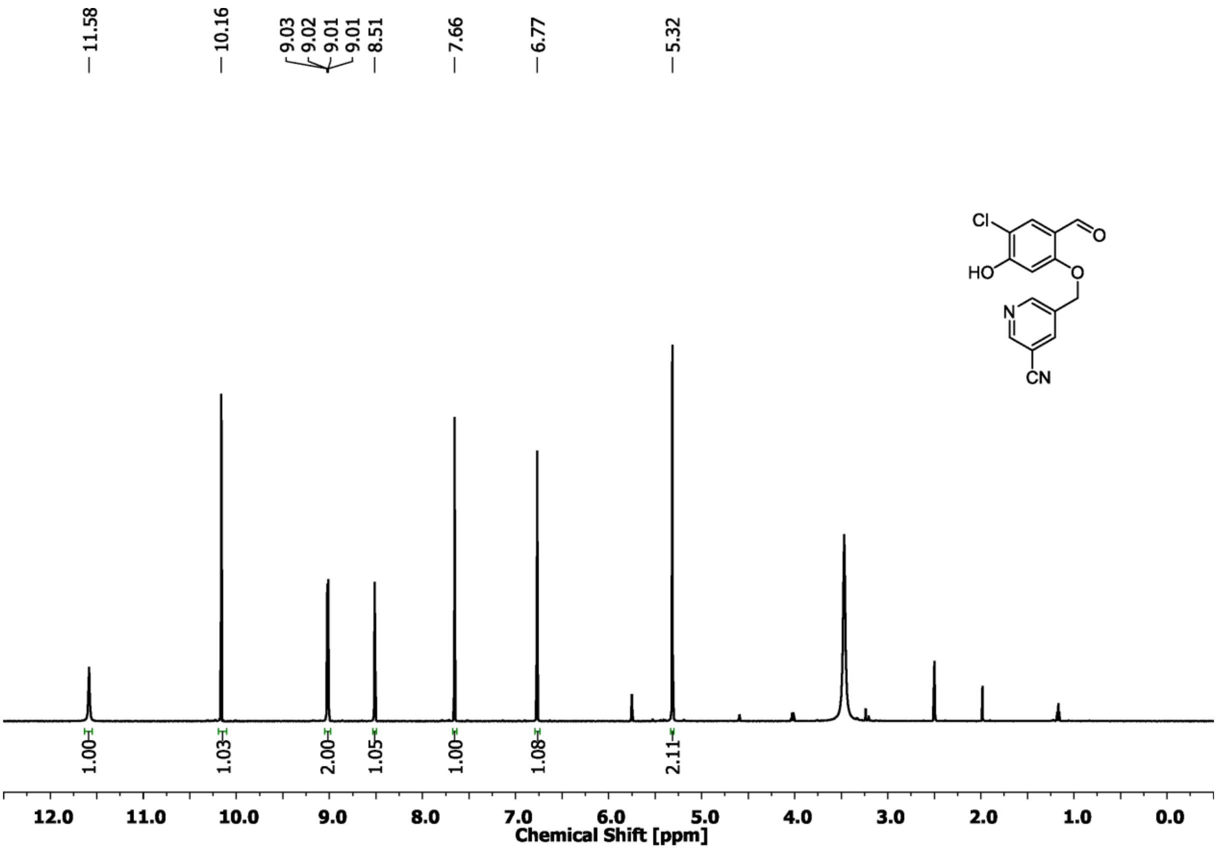

Figure S57: ^{1}H NMR spectrum (DMSO-d_6, 600 MHz, 298 K) of compound 51a.

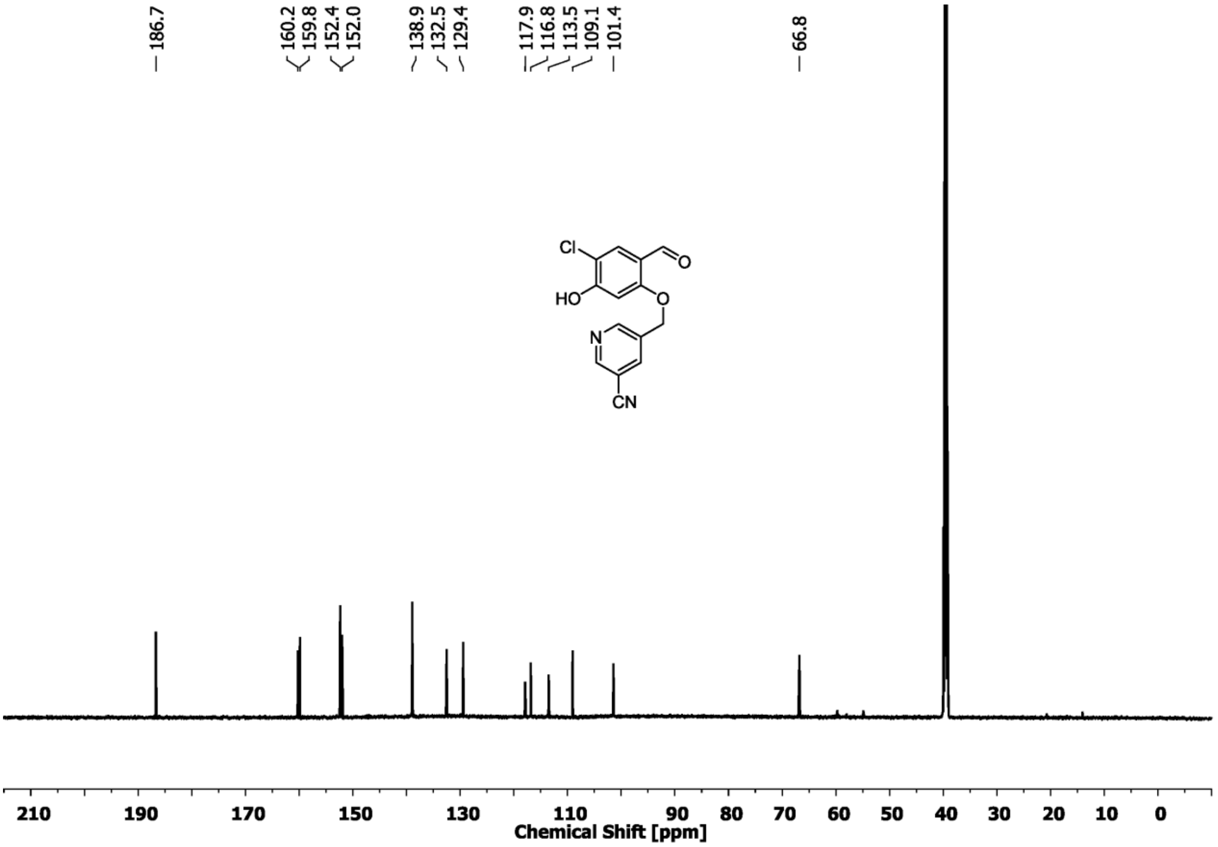

Figure S58: ^{13}C NMR spectrum (DMSO-d_6, 151 MHz, 298 K) of compound 51a.

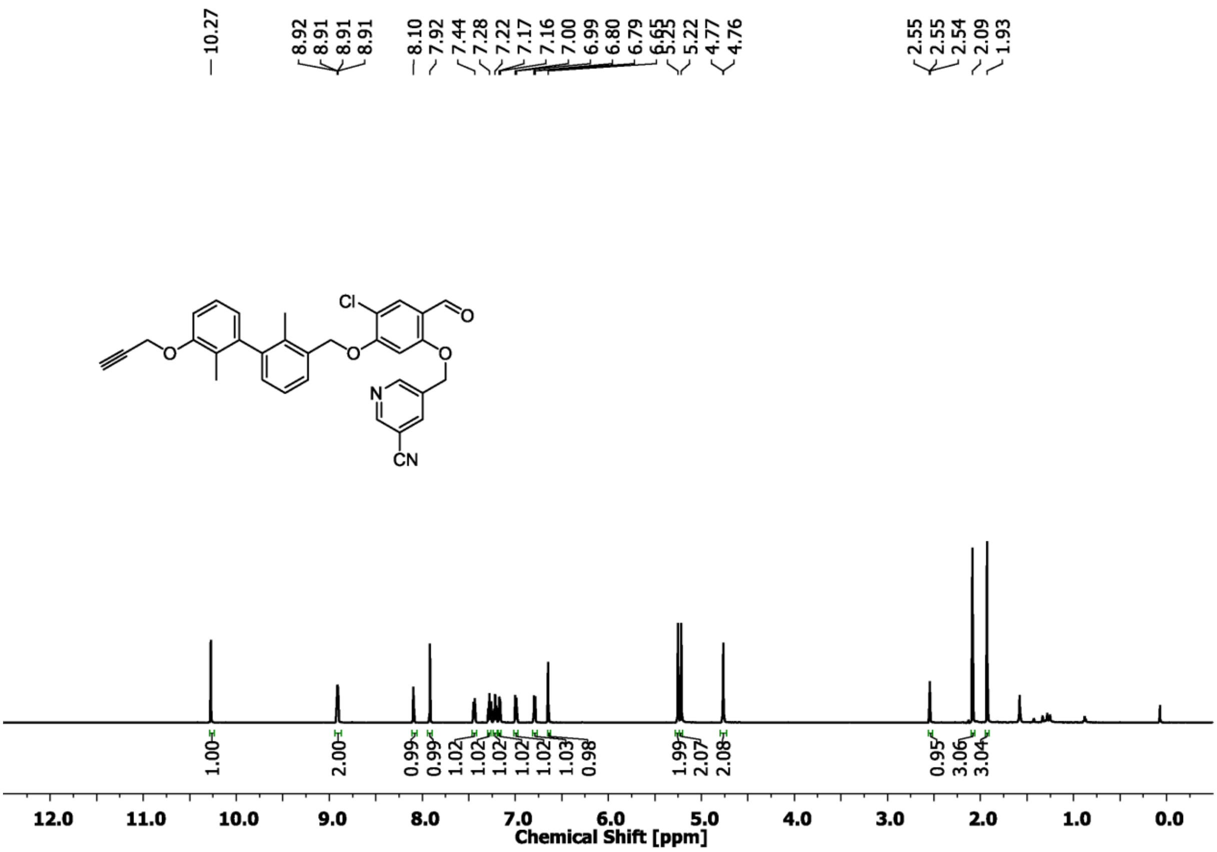

Figure S59: ^{1}H NMR spectrum ($CDCl_3$, 600 MHz, 298 K) of compound 52a.

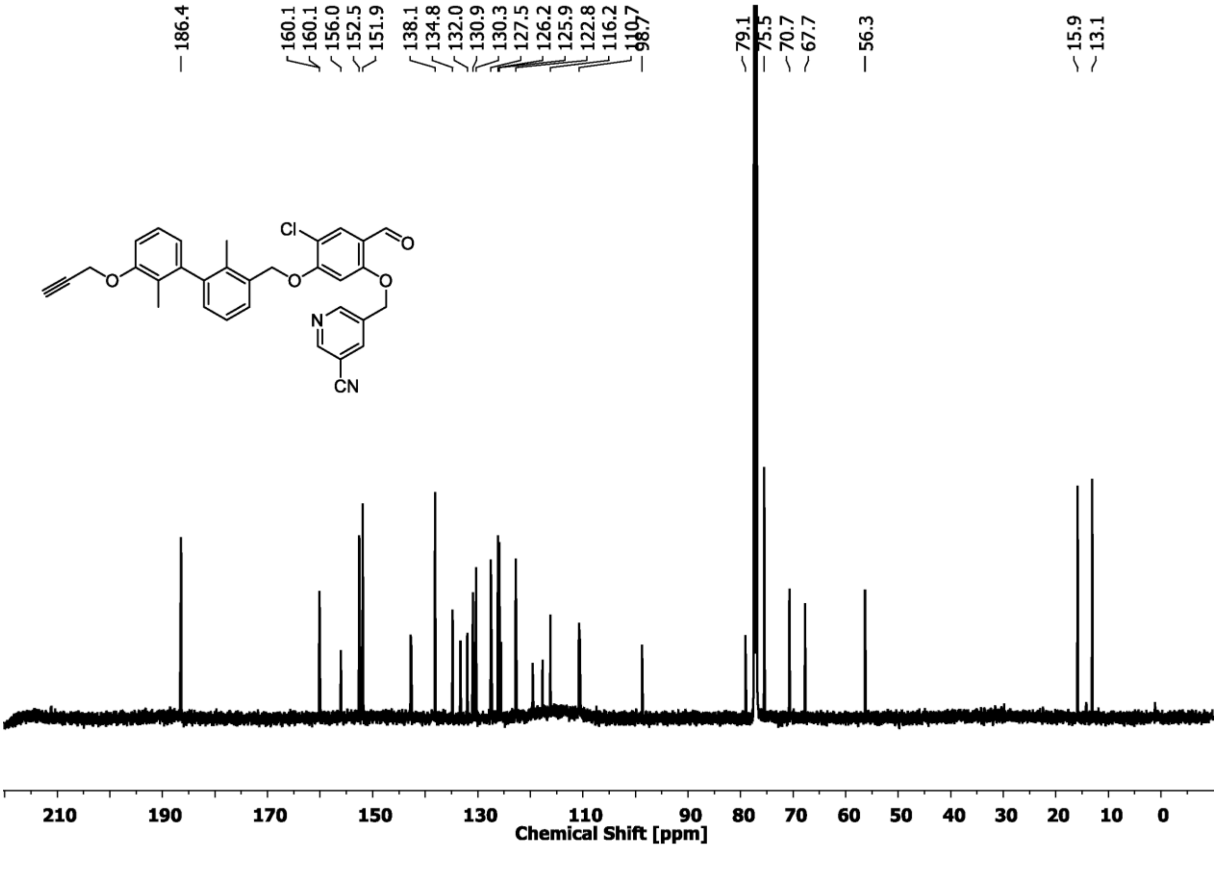

Figure S60: ^{13}C NMR spectrum ($CDCl_3$, 151 MHz, 298 K) of compound 52a.

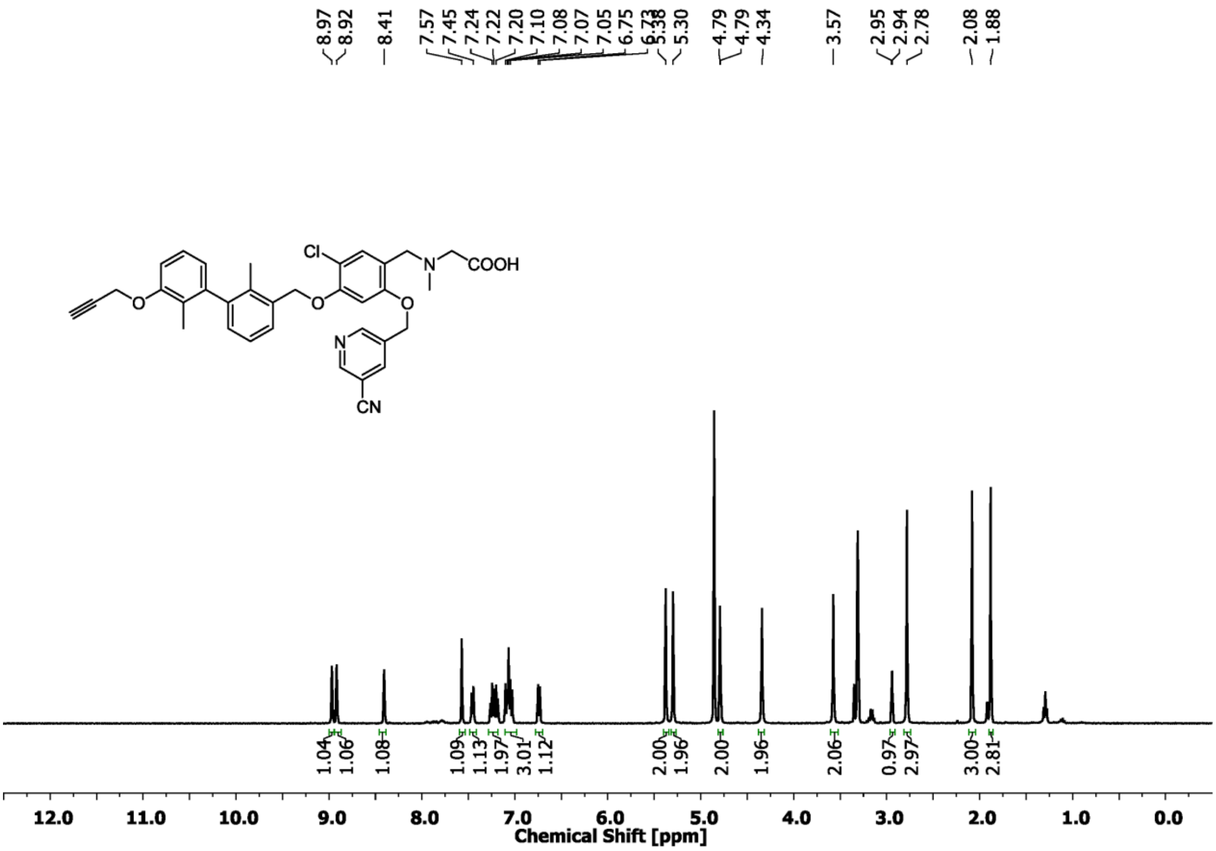

Figure S61: ^{1}H NMR spectrum (methanol-d_4, 400 MHz, 298 K) of compound 53a.

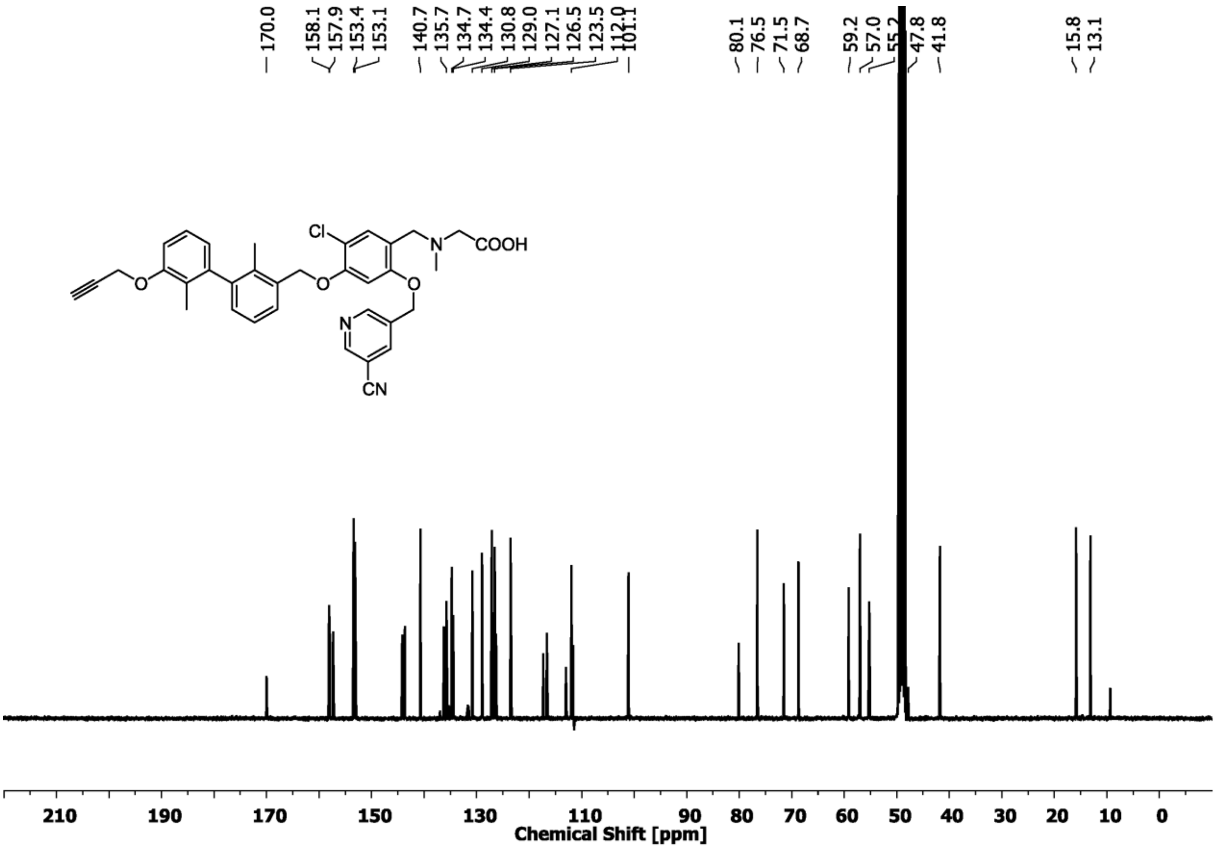

Figure S62: ^{13}C NMR spectrum (methanol-d_4, 101 MHz, 298 K) of compound 53a.

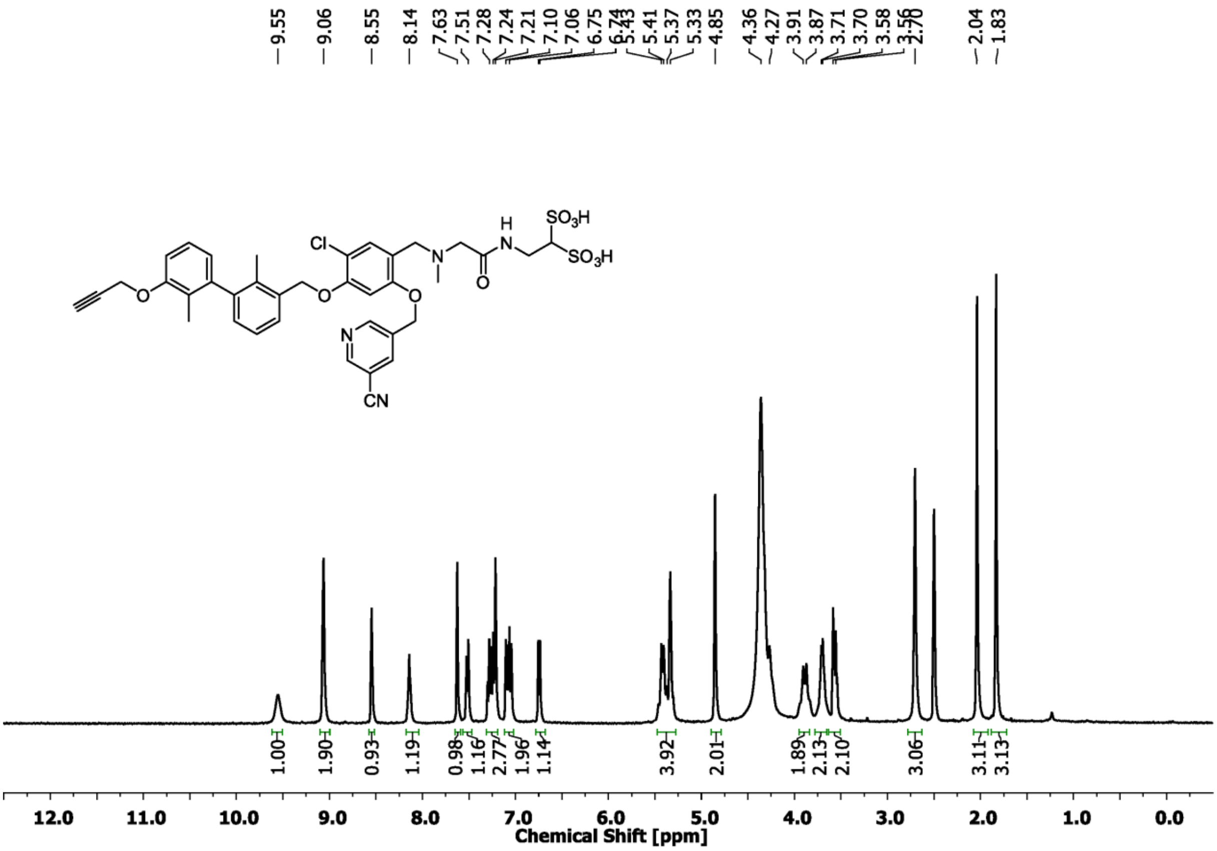

Figure S63: ^{1}H NMR spectrum (DMSO-d_6, 400 MHz, 298 K) of compound 54a.

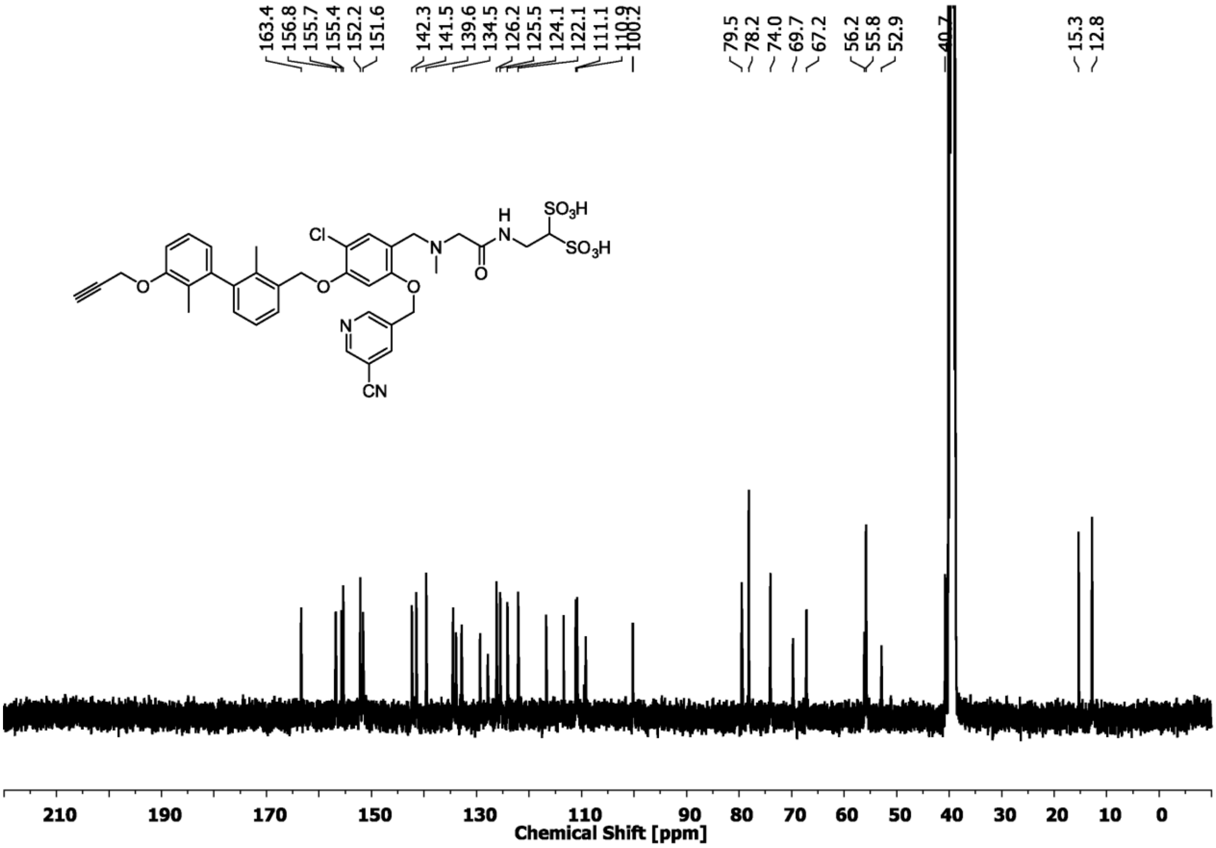

Figure S64: ^{13}C NMR spectrum (DMSO-d_6, 101 MHz, 298 K) of compound 54a.

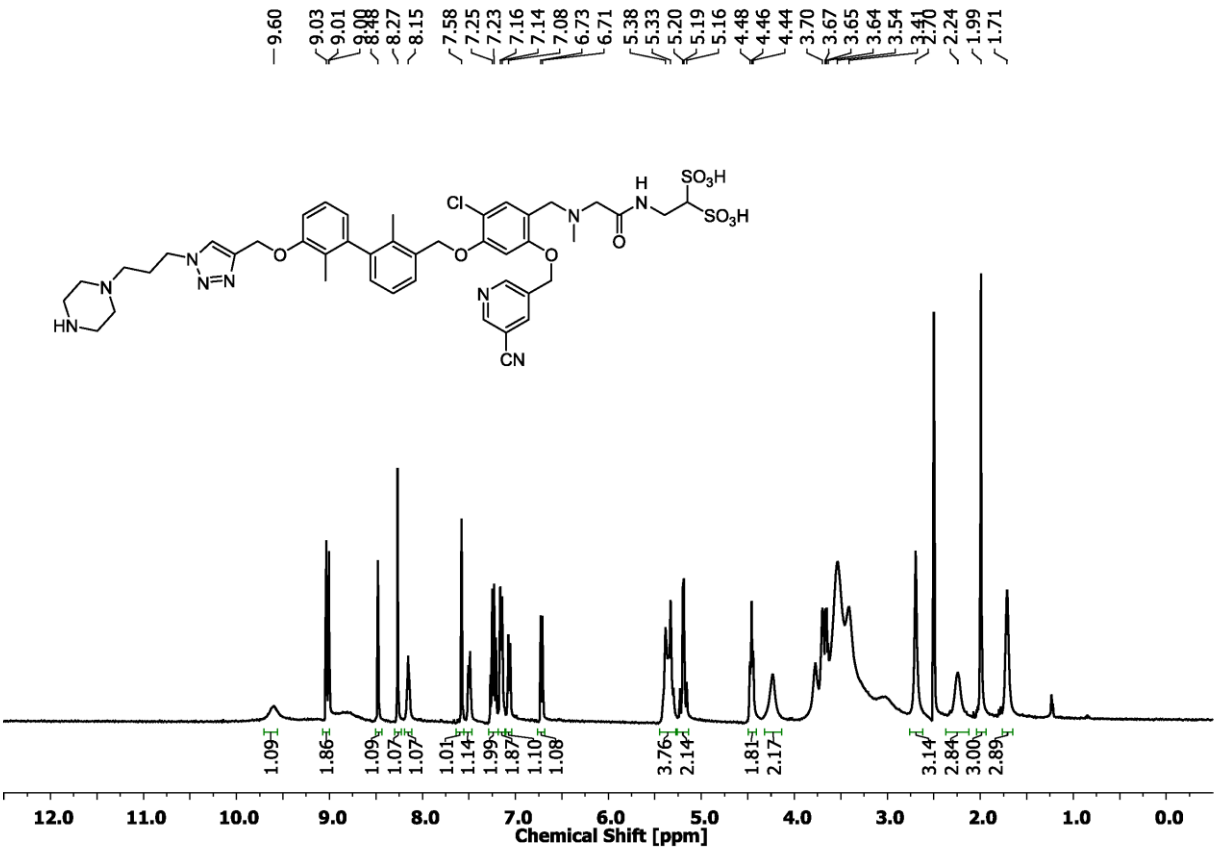

Figure S65: ^{1}H NMR spectrum (DMSO-d_6, 400 MHz, 298 K) of compound 55a.

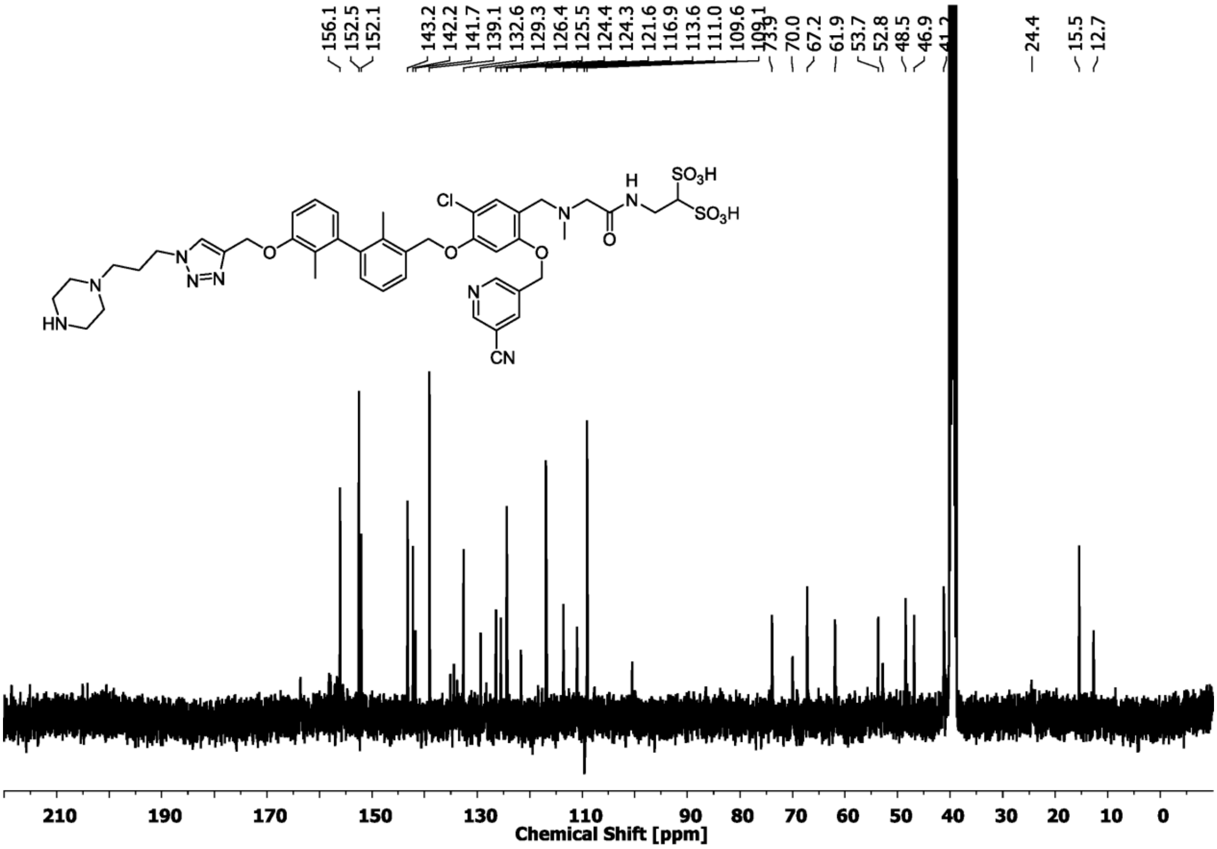

Figure S66: ^{13}C NMR spectrum (DMSO-d_6, 101 MHz, 298 K) of compound 55a.

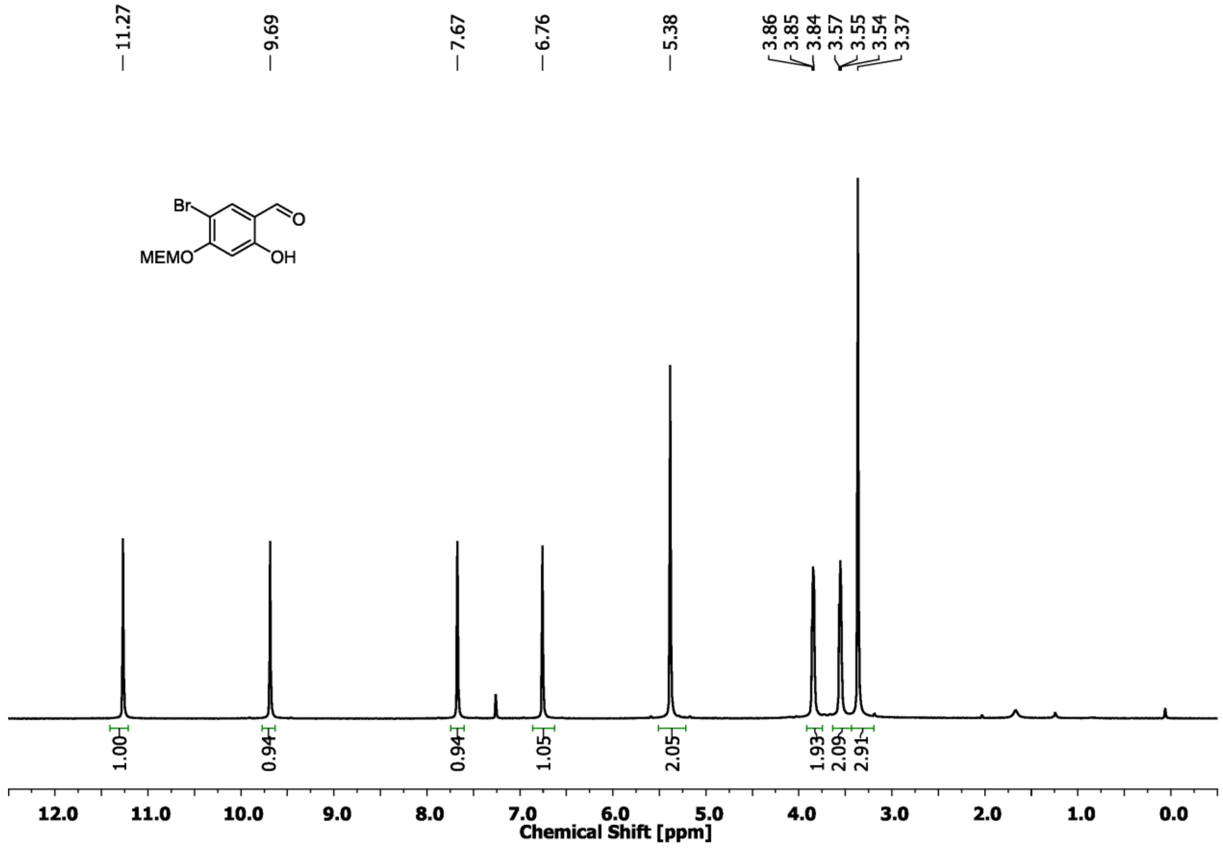

Figure S67: ¹H NMR spectrum ($CDCl_3$, 400 MHz, 298 K) of compound 45.

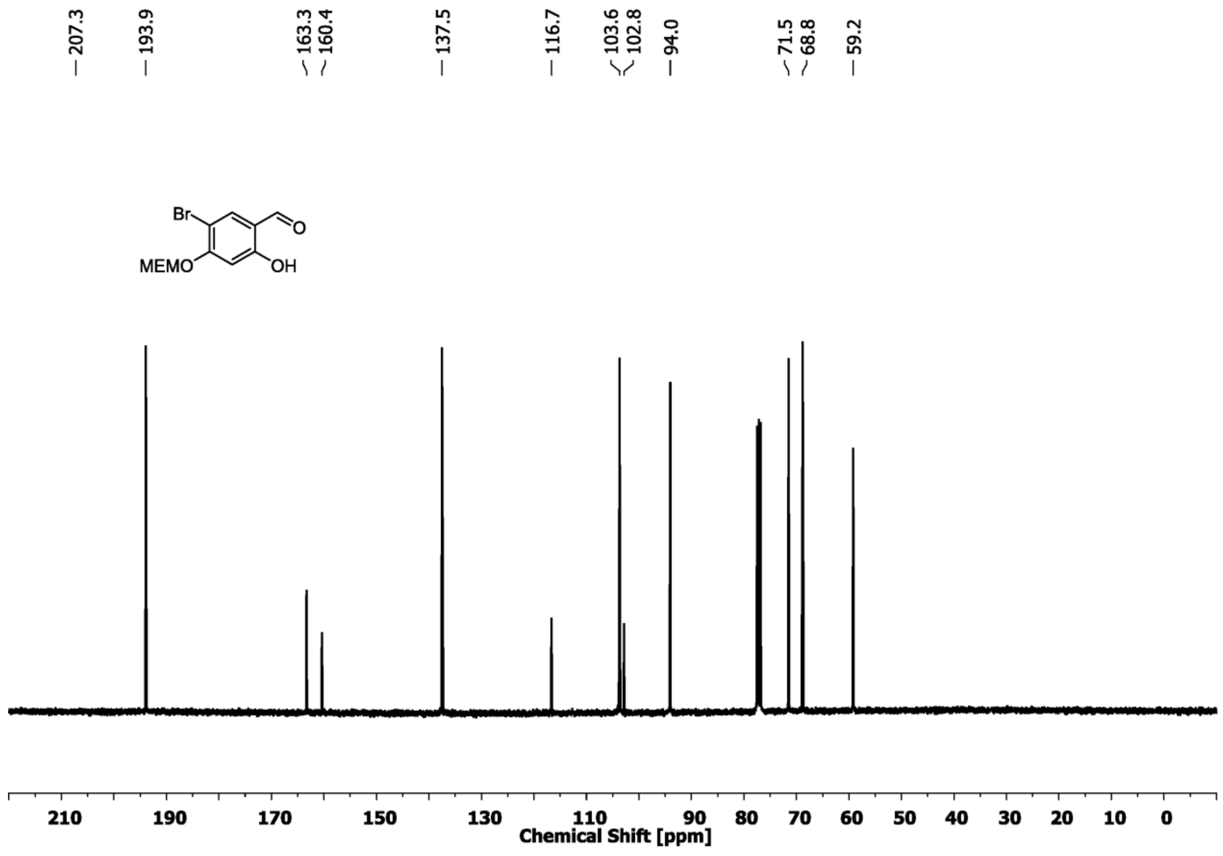

Figure S68: ^{13}C NMR spectrum ($CDCl_3$, 101 MHz, 298 K) of compound 45.

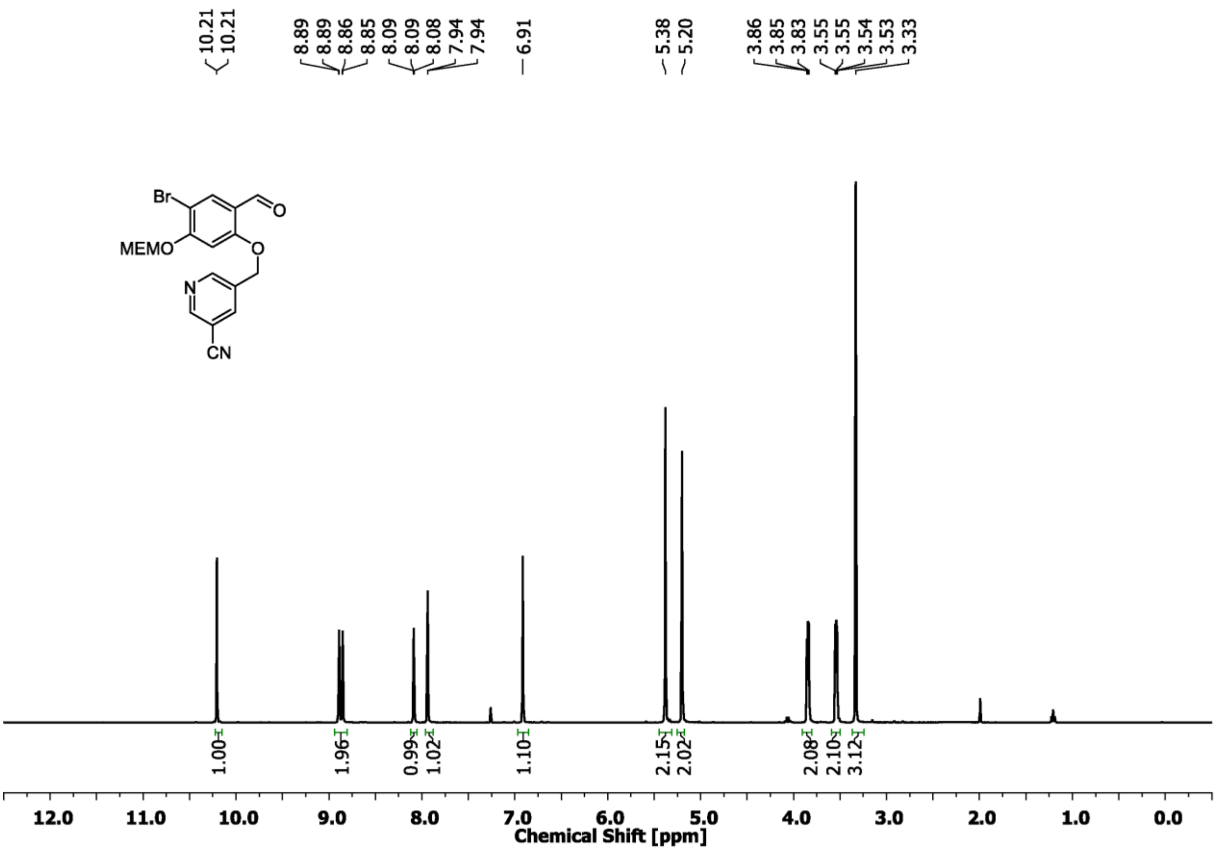

Figure S69: ^{1}H NMR spectrum ($CDCl_3$, 400 MHz, 298 K) of compound 46b.

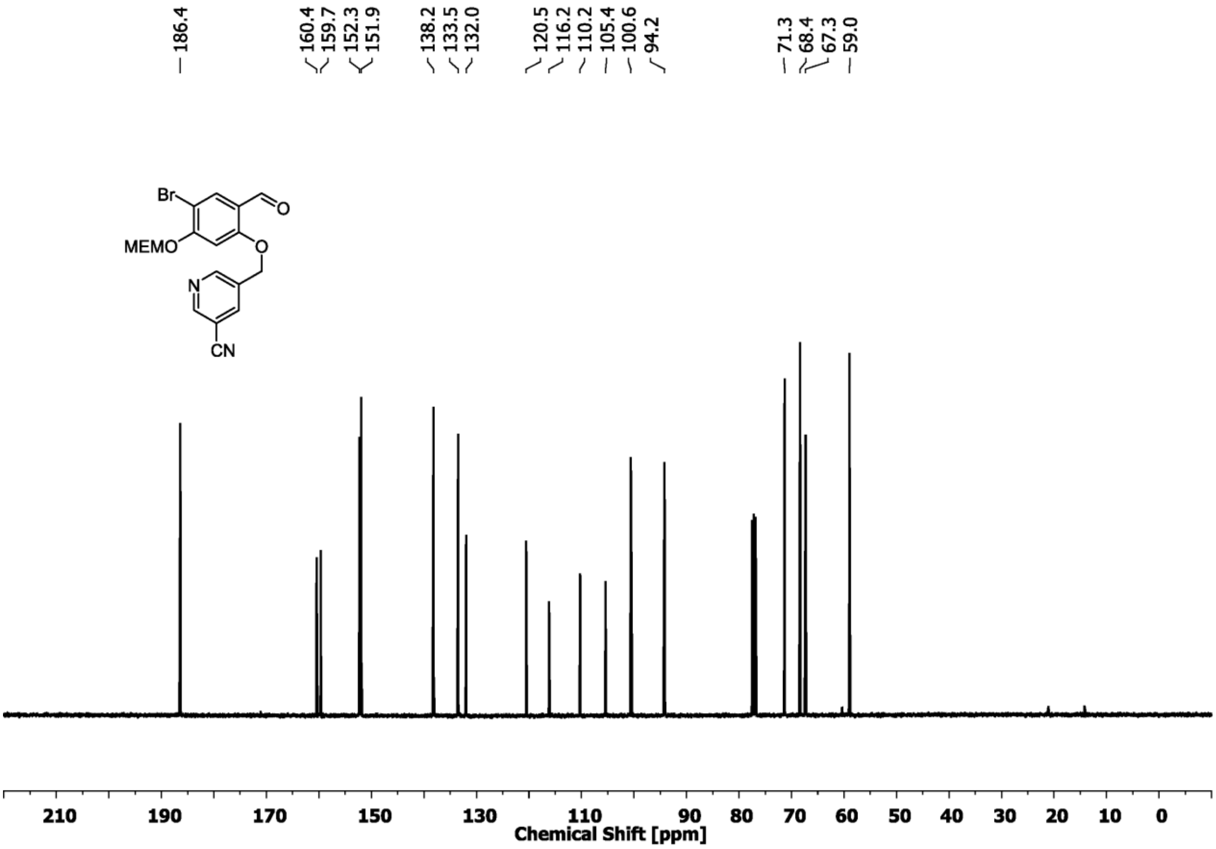

Figure S70: ^{13}C NMR spectrum ($CDCl_3$, 101 MHz, 298 K) of compound 46b.

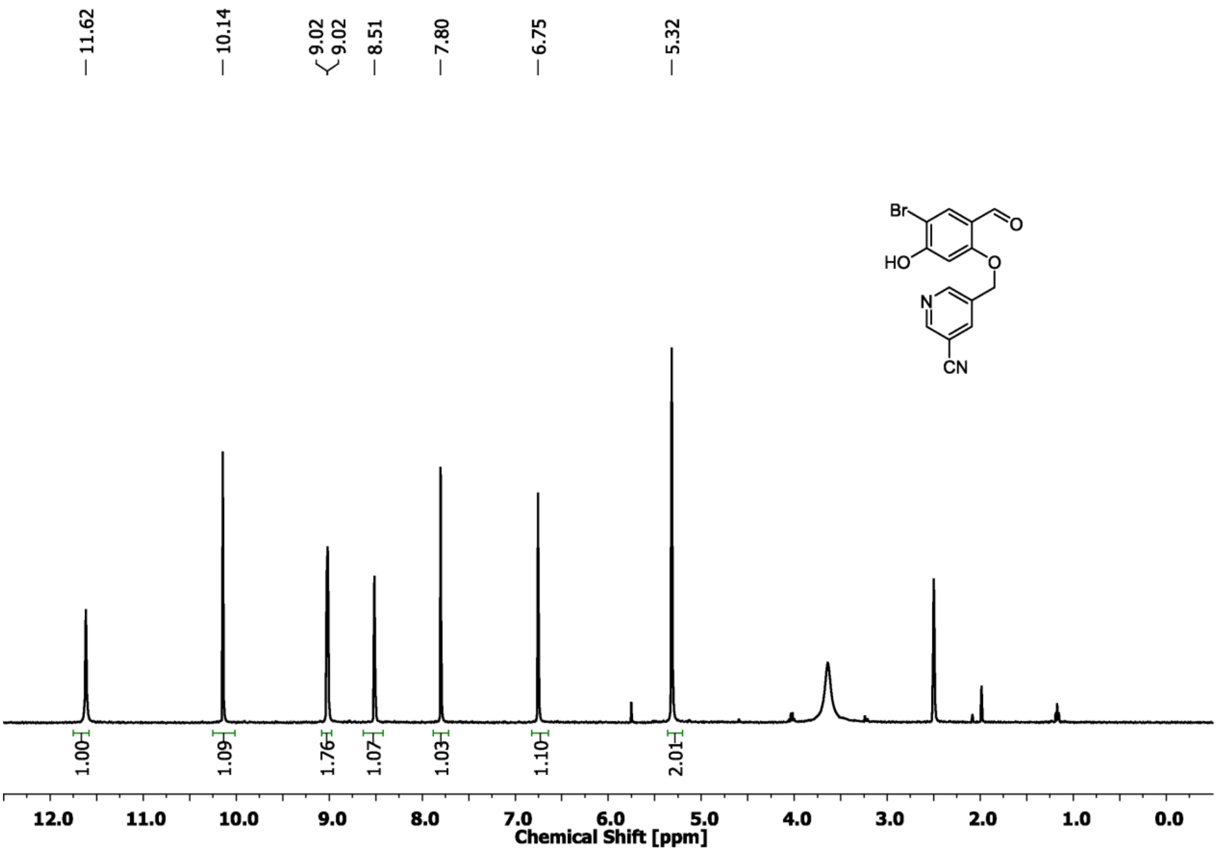

Figure S71: ^{1}H NMR spectrum (DMSO-d_6, 400 MHz, 298 K) of compound 51b.

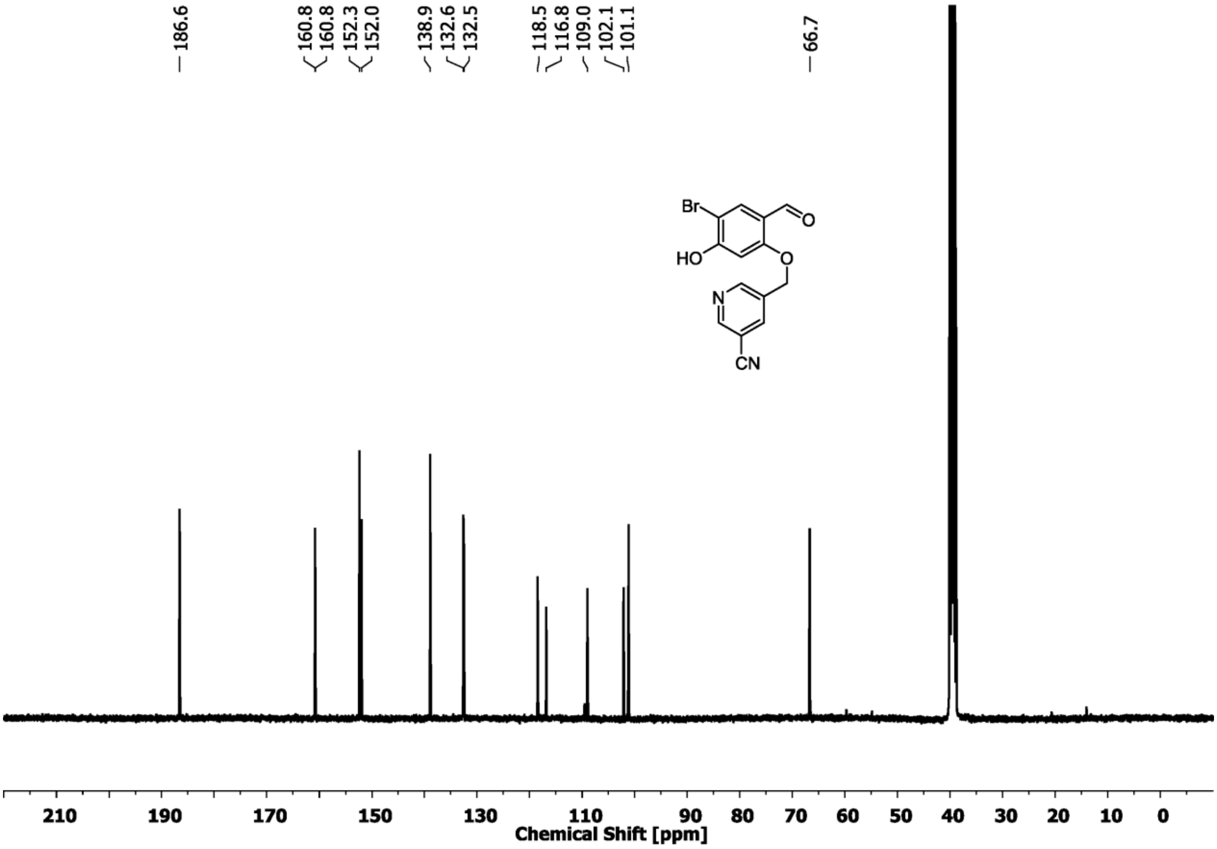

Figure S72: ^{13}C NMR spectrum (DMSO-d_6, 101 MHz, 298 K) of compound 51b.

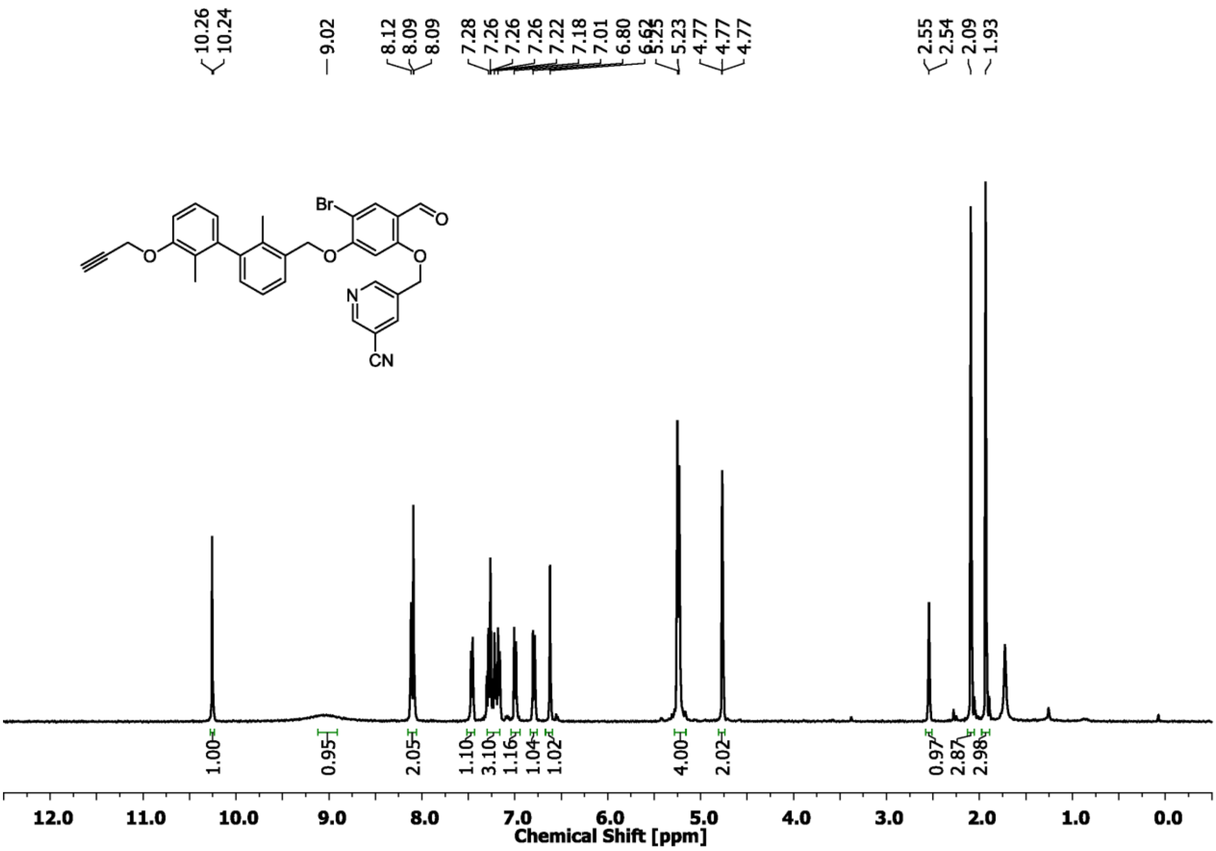

Figure S73: ^{1}H NMR spectrum (CDCl$_3$, 400 MHz, 298 K) of compound 52b.

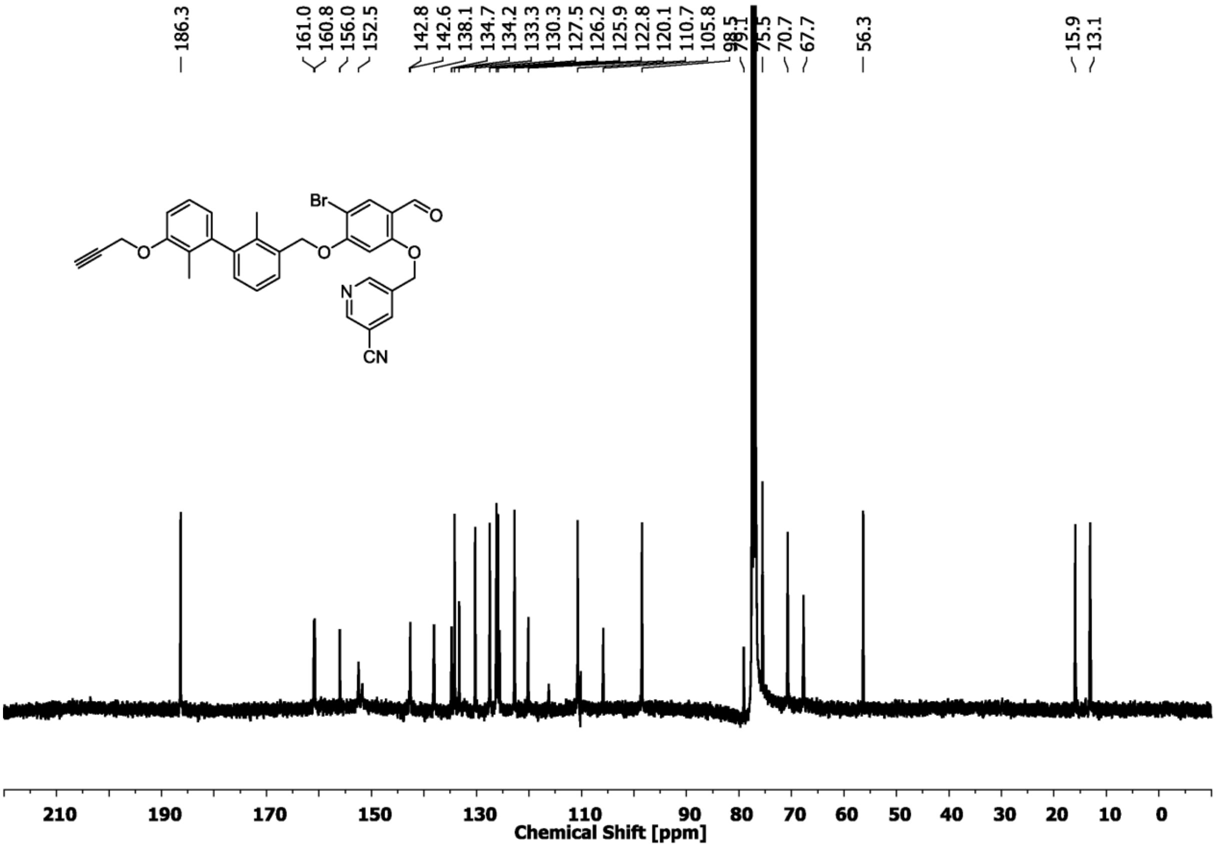

Figure S74: ^{13}C NMR spectrum (CDCl$_3$, 101 MHz, 298 K) of compound 52b.

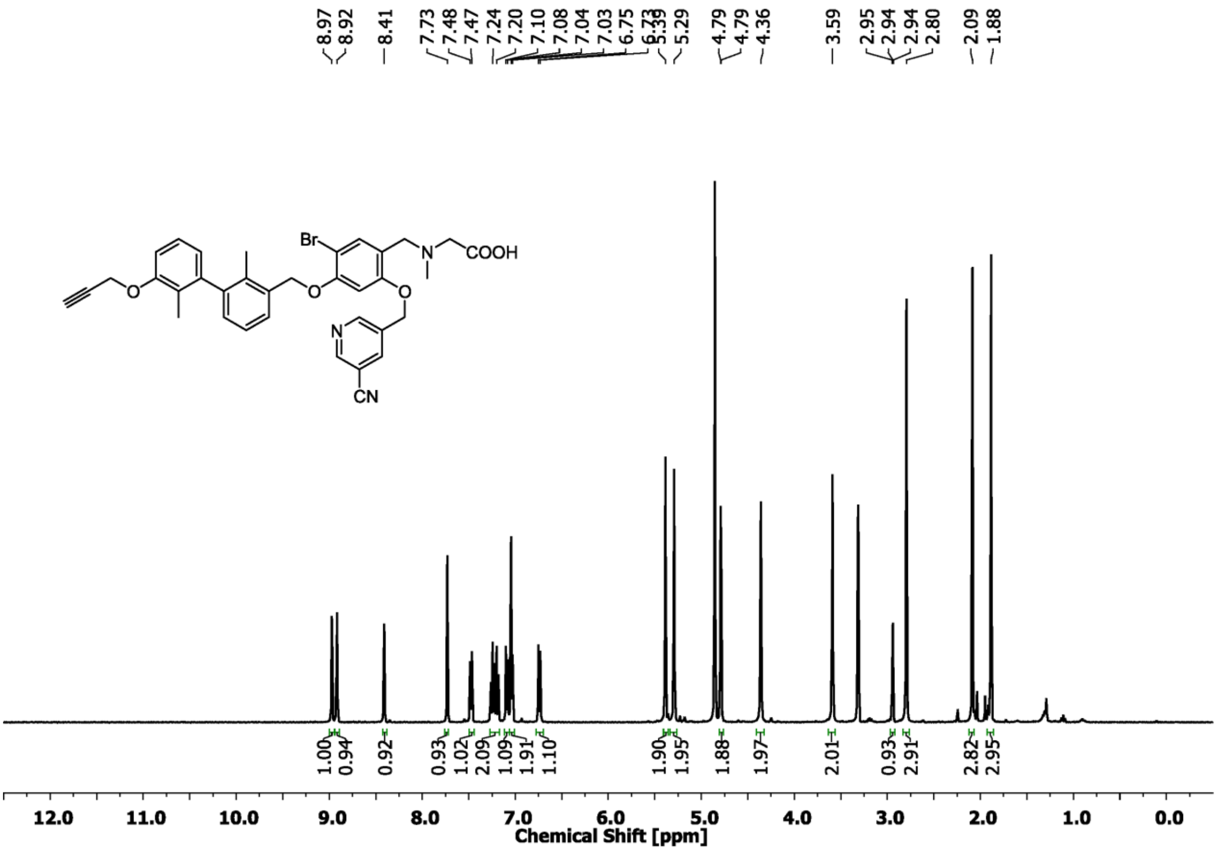

Figure S75: ¹H NMR spectrum (methanol-d_4, 400 MHz, 298 K) of compound 53b.

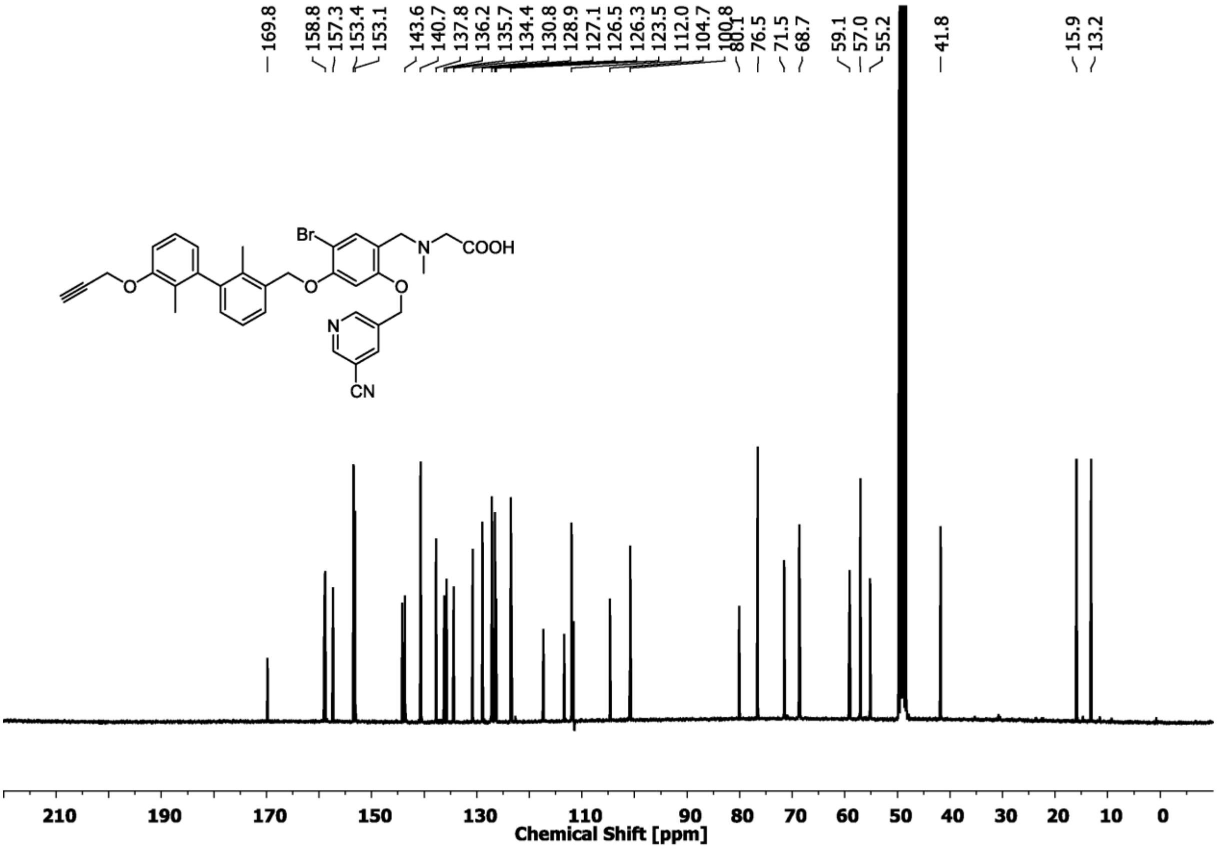

Figure S76: ¹³C NMR spectrum (methanol-d_4, 101 MHz, 298 K) of compound 53b.

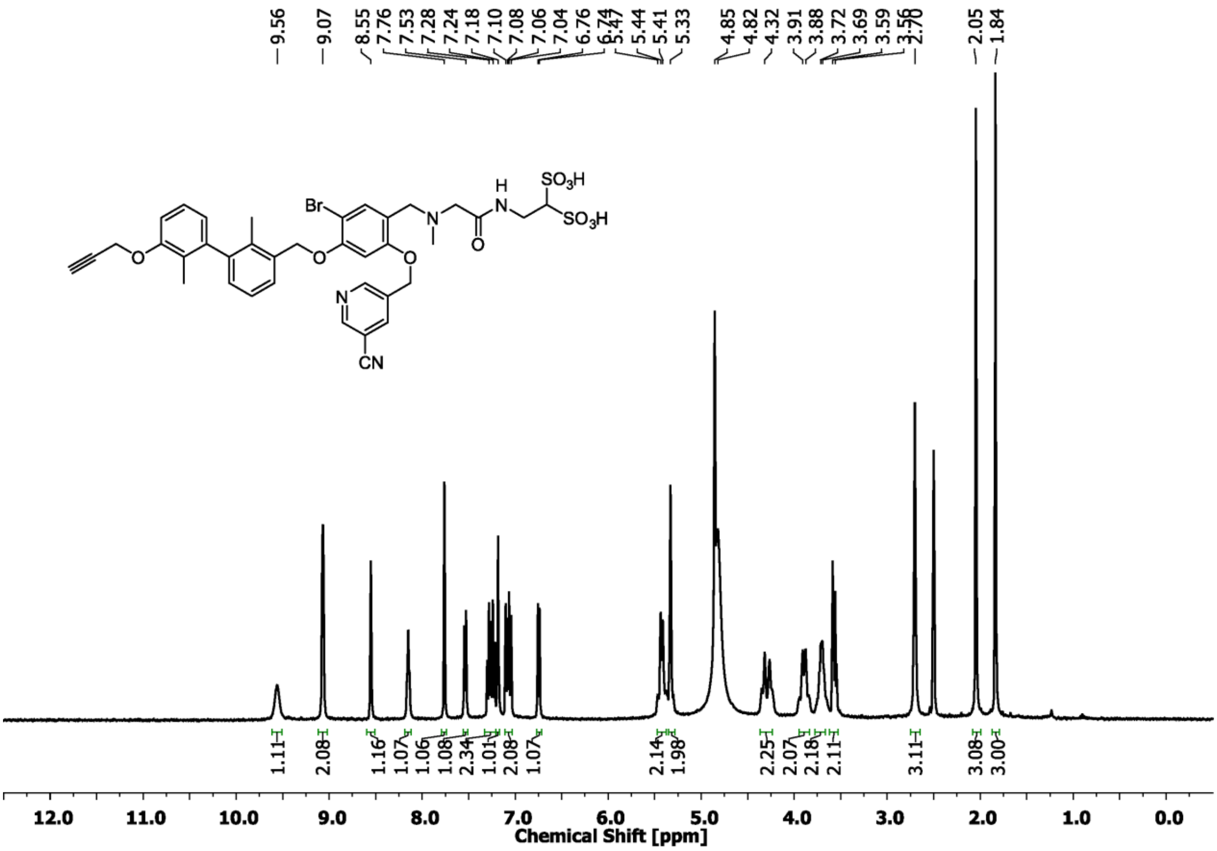

Figure S77: ^{1}H NMR spectrum (DMSO-d_6, 400 MHz, 298 K) of compound 54b.

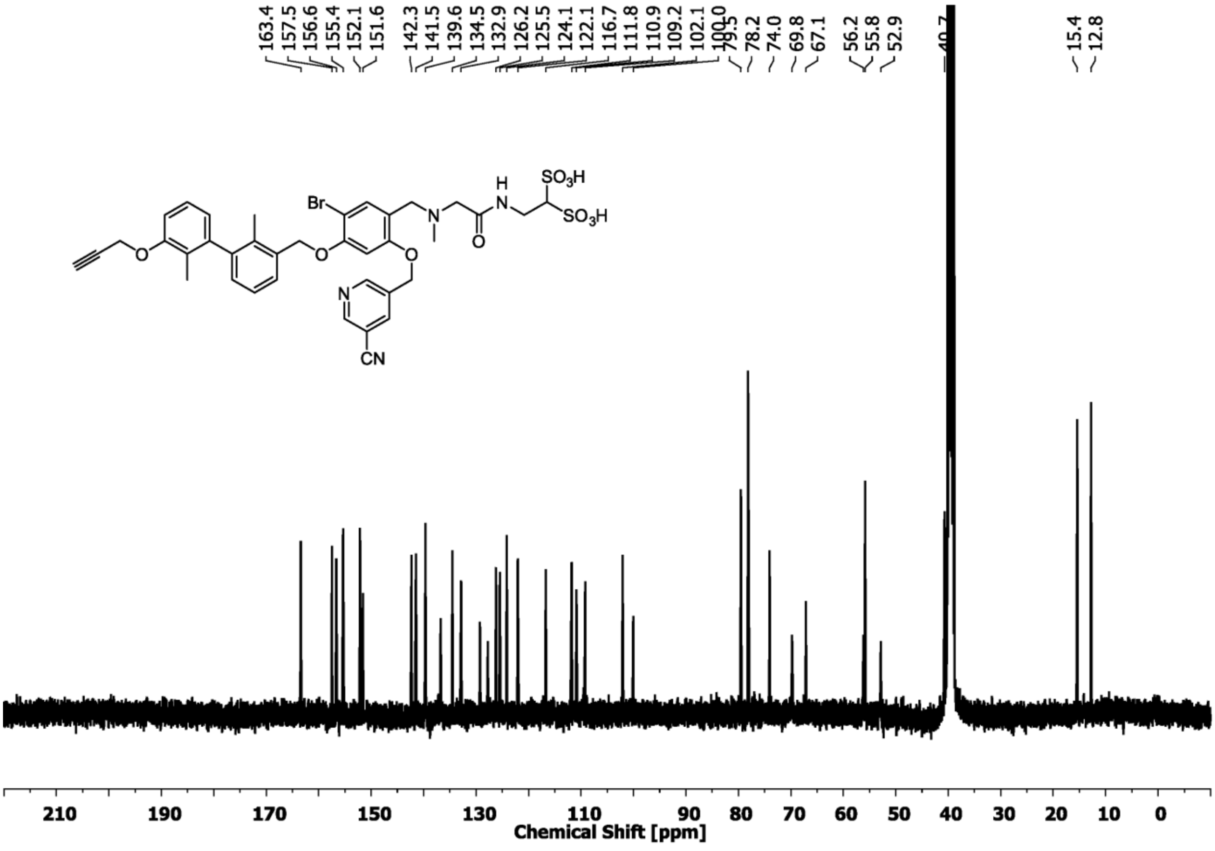

Figure S78: ^{13}C NMR spectrum (DMSO-d_6, 101 MHz, 298 K) of compound 54b.

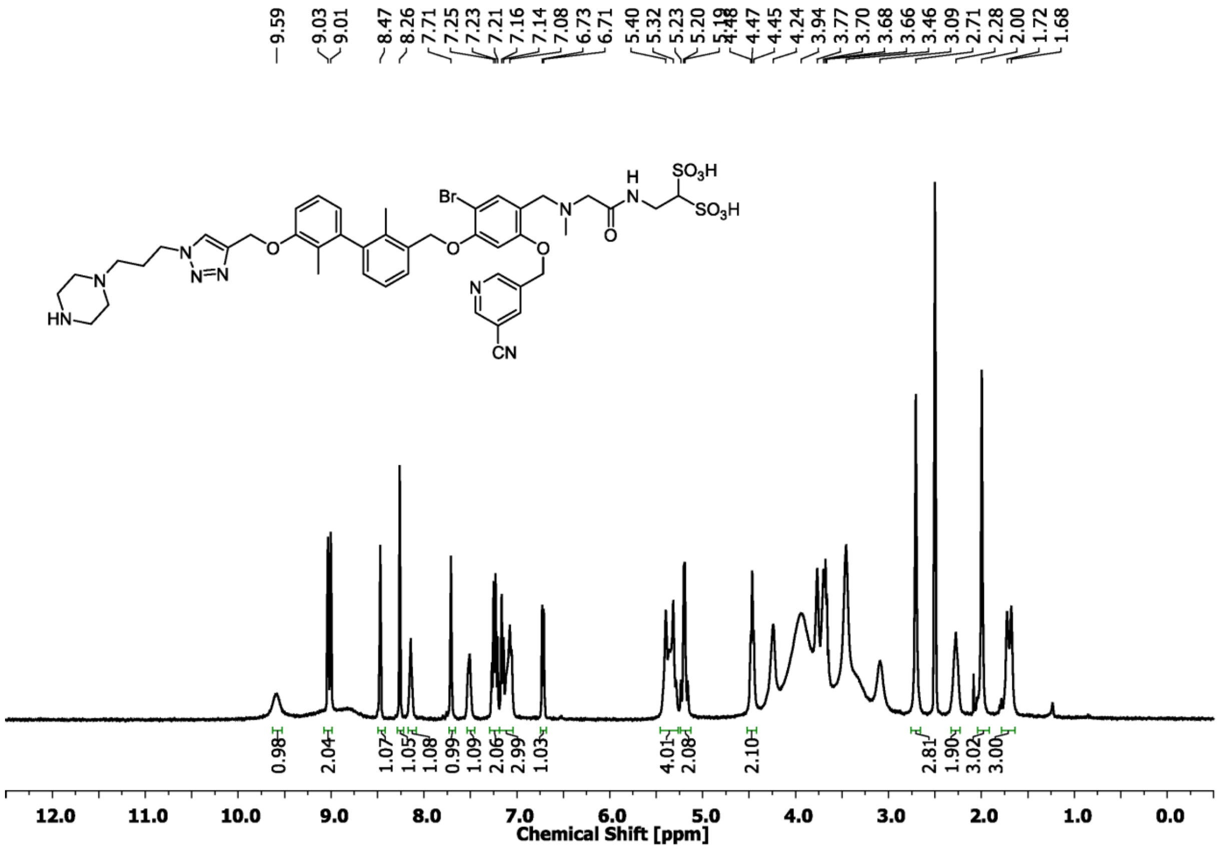

Figure S79: ^{1}H NMR spectrum (DMSO-d_6, 400 MHz, 298 K) of compound 55a.

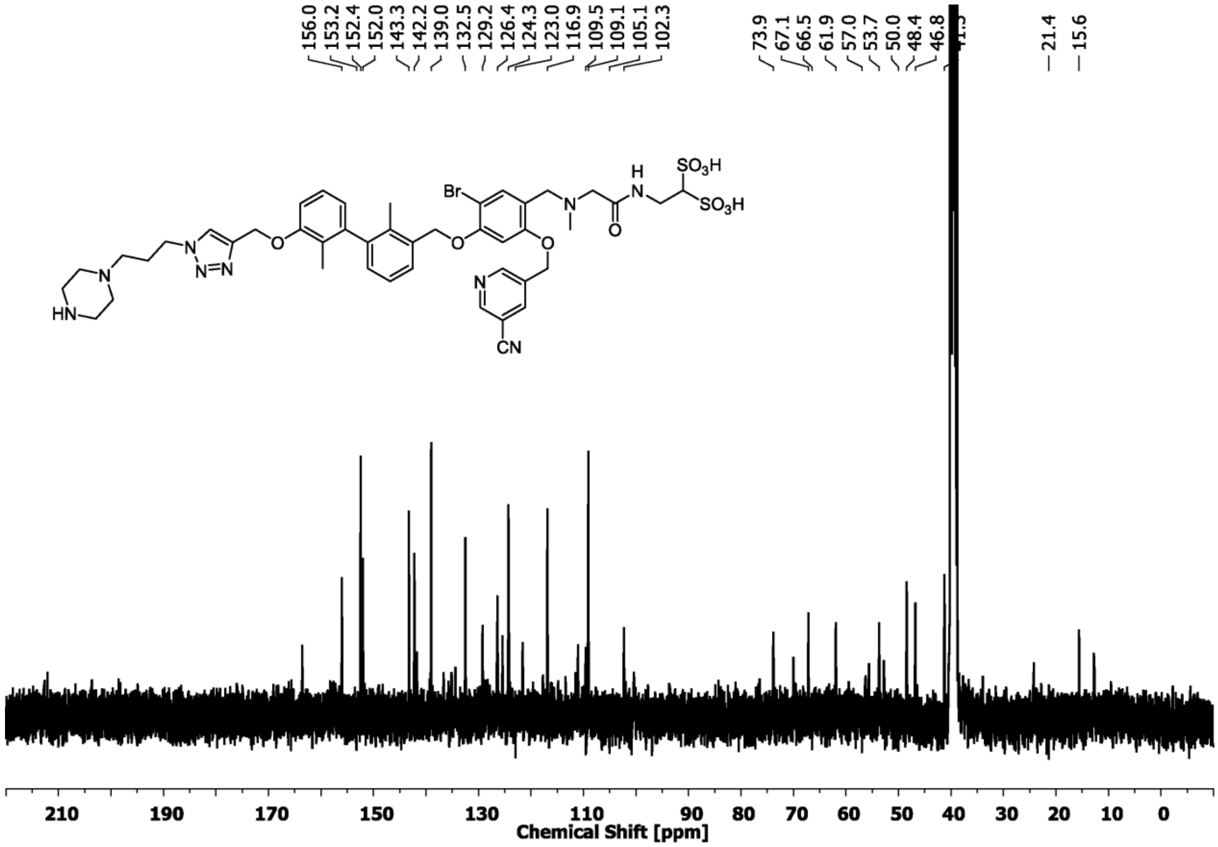

Figure S80: ^{13}C NMR spectrum (DMSO-d_6, 101 MHz, 298 K) of compound 55a.

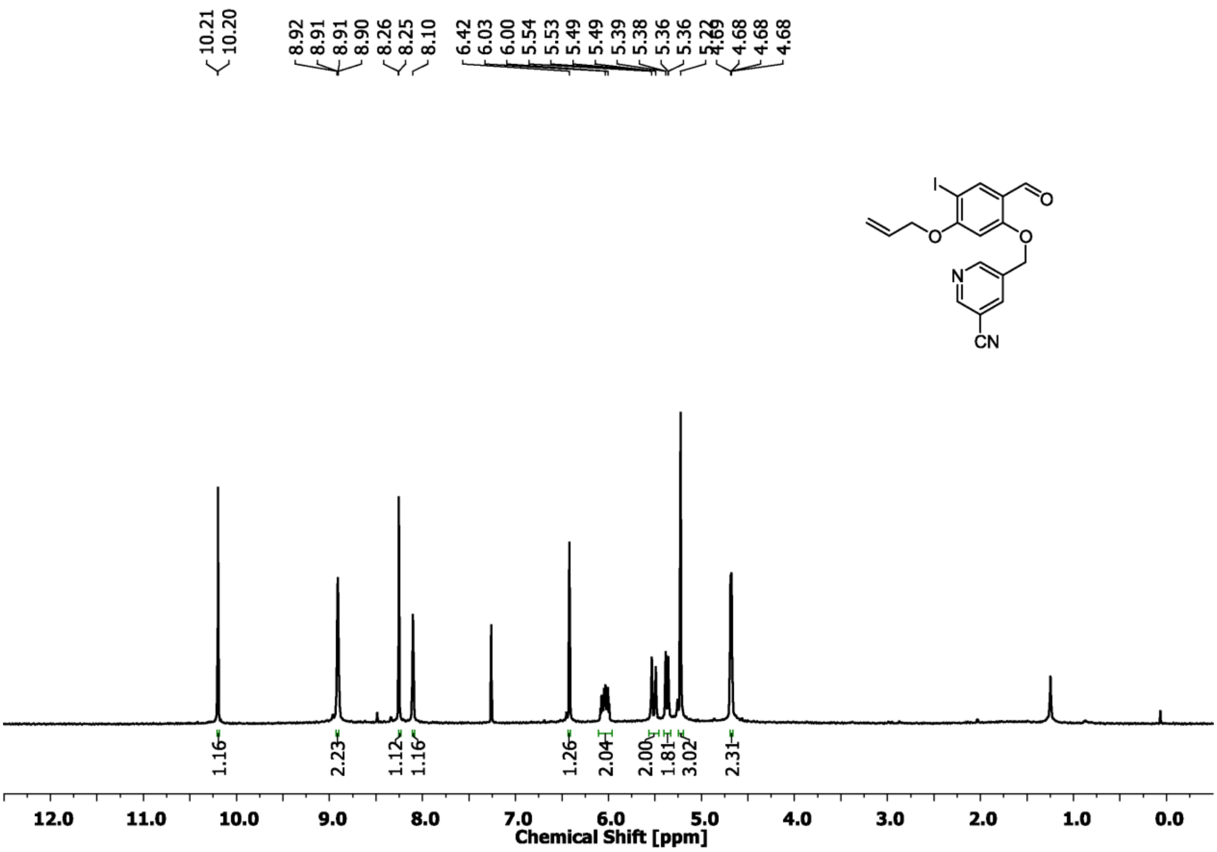

Figure S81: ^{1}H NMR spectrum ($CDCl_3$, 400 MHz, 298 K) of compound 50.

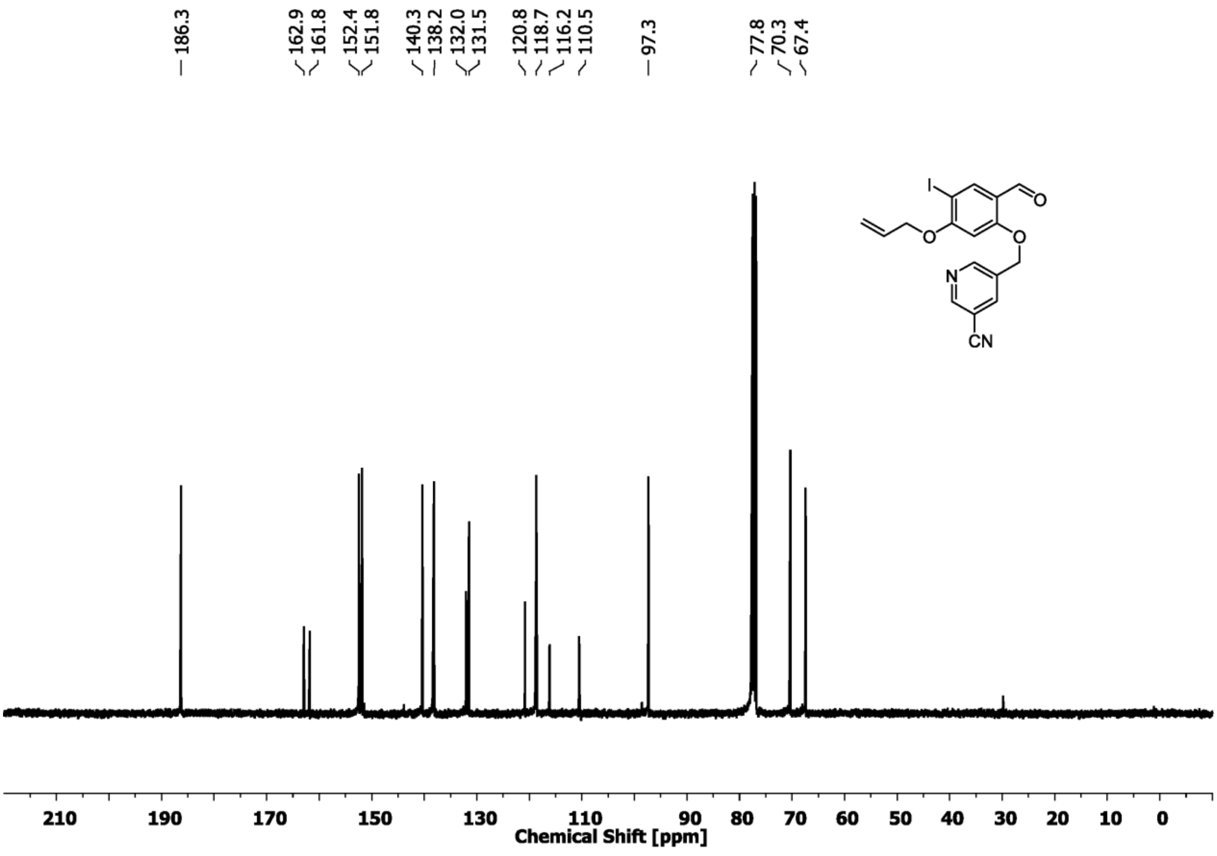

Figure S82: ^{13}C NMR spectrum ($CDCl_3$, 101 MHz, 298 K) of compound 50.

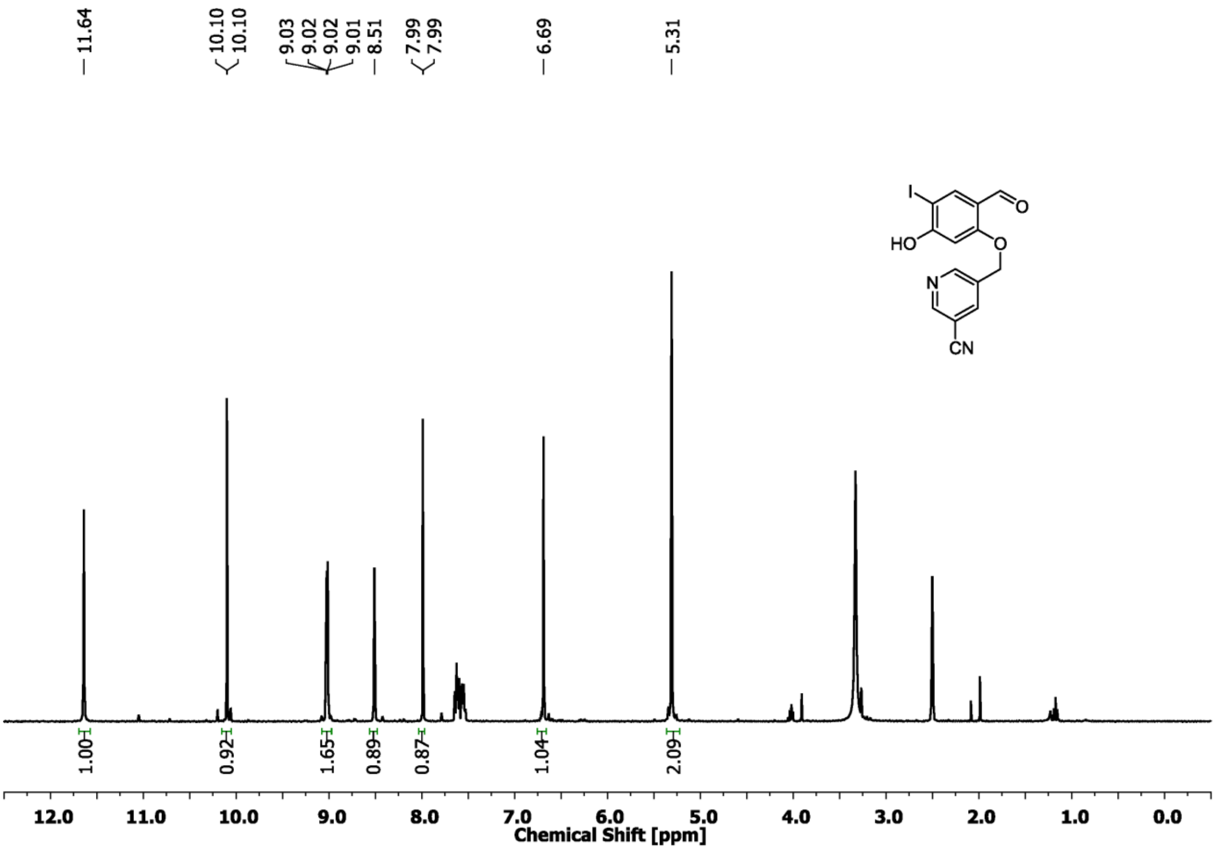

Figure S83: ^{1}H NMR spectrum (DMSO-d_6, 400 MHz, 298 K) of compound 51c.

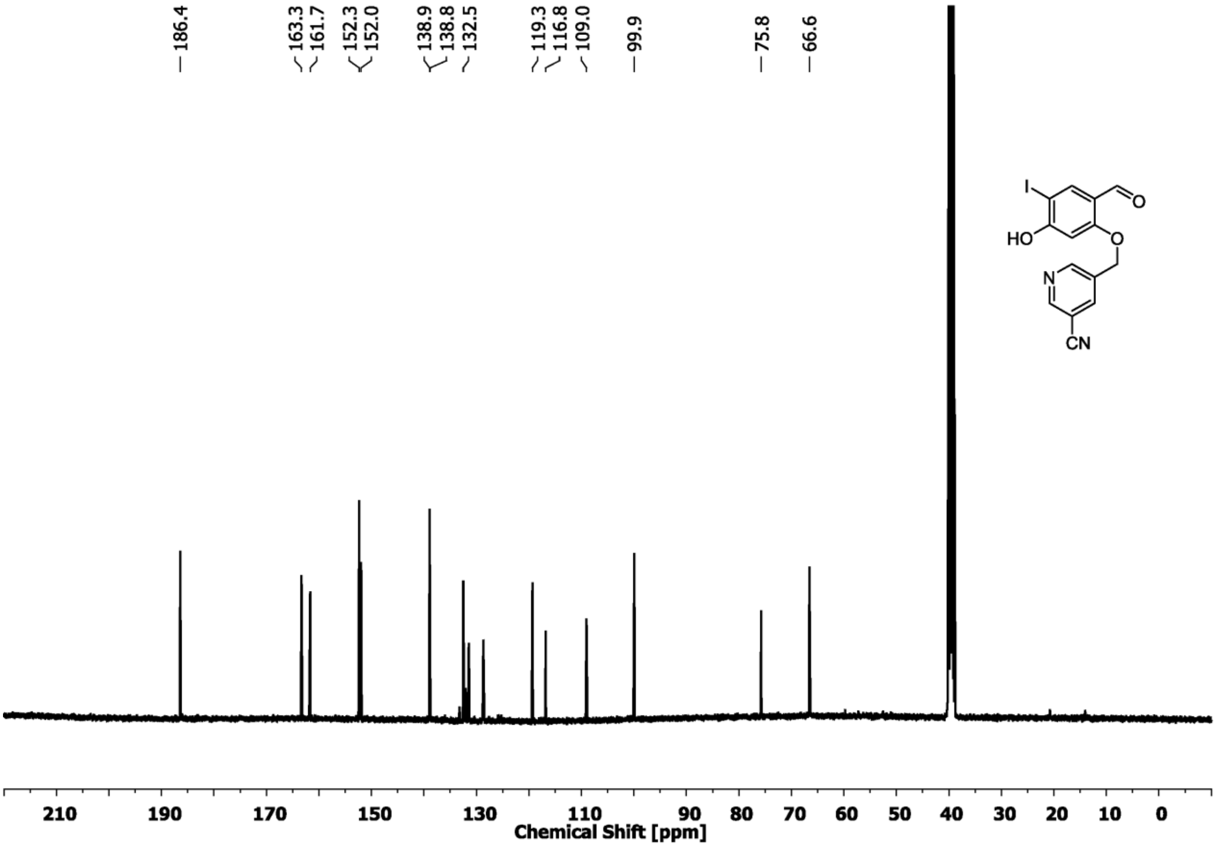

Figure S84: ^{13}C NMR spectrum (DMSO-d_6, 101 MHz, 298 K) of compound 51c.

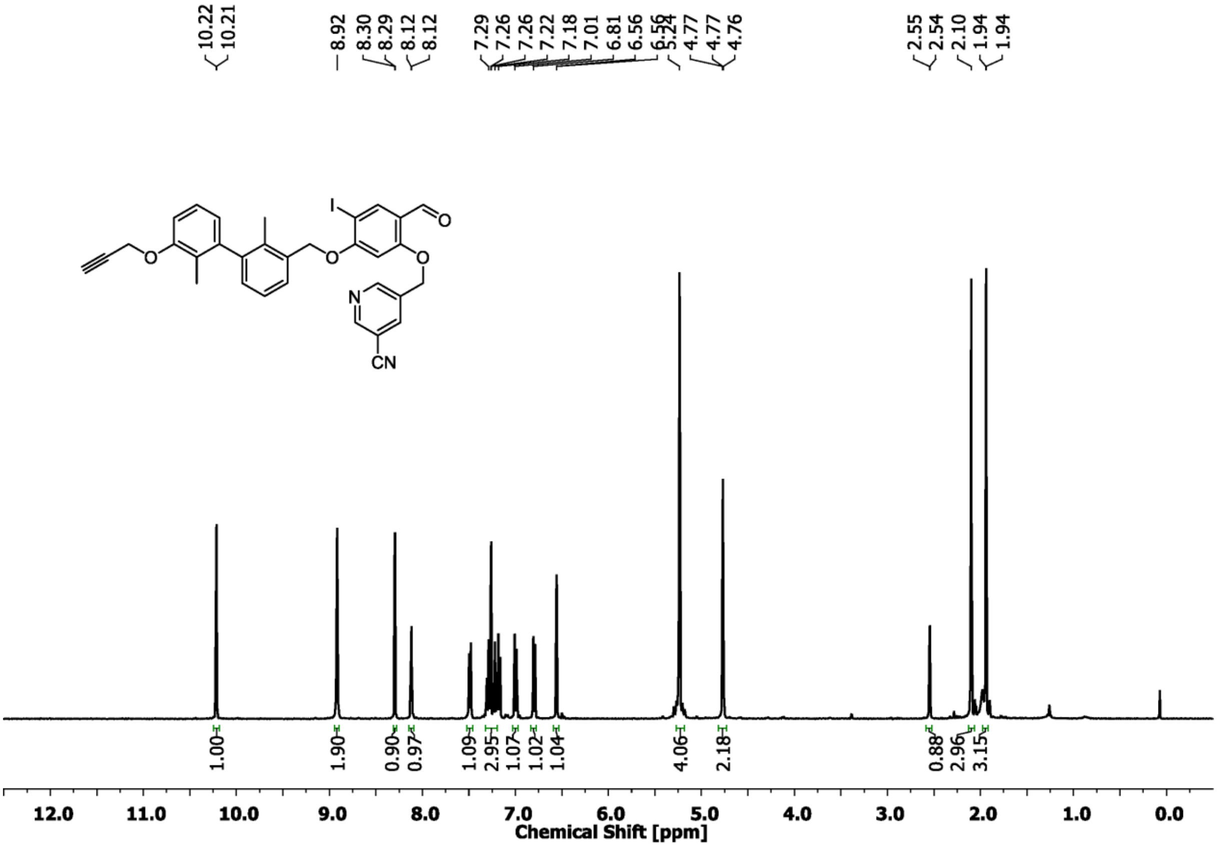

Figure S85: ^{1}H NMR spectrum ($CDCl_3$, 400 MHz, 298 K) of compound 52c.

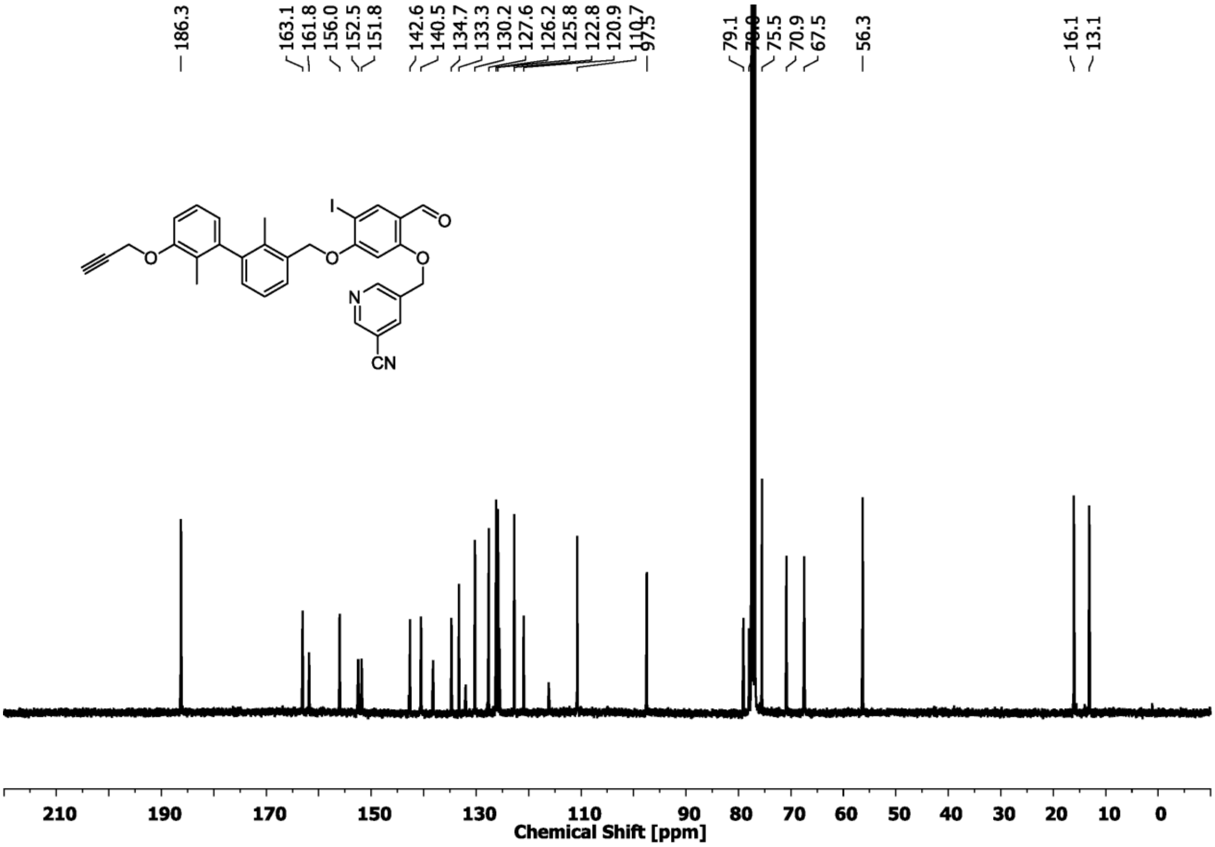

Figure S86: ^{13}C NMR spectrum ($CDCl_3$, 101 MHz, 298 K) of compound 52c.

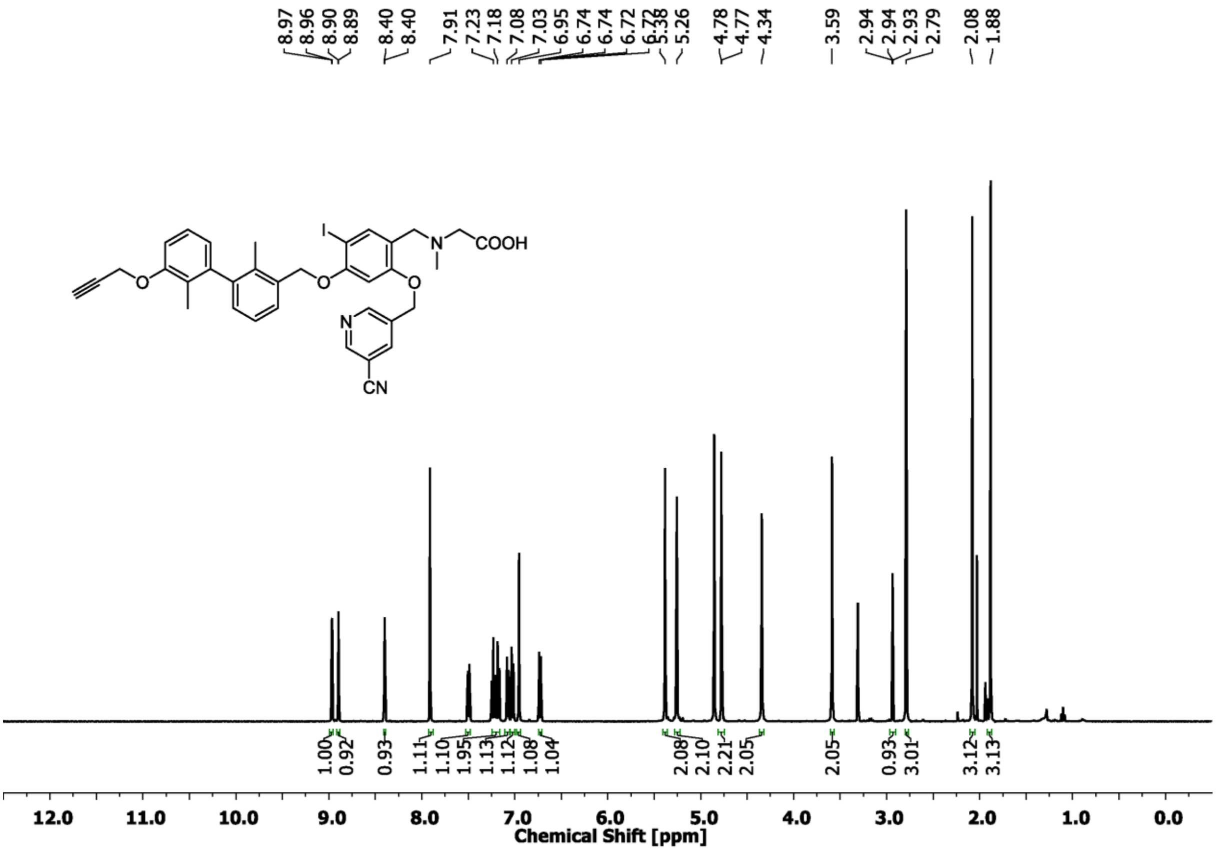

Figure S87: ^{1}H NMR spectrum (methanol-d_4, 400 MHz, 298 K) of compound 53c.

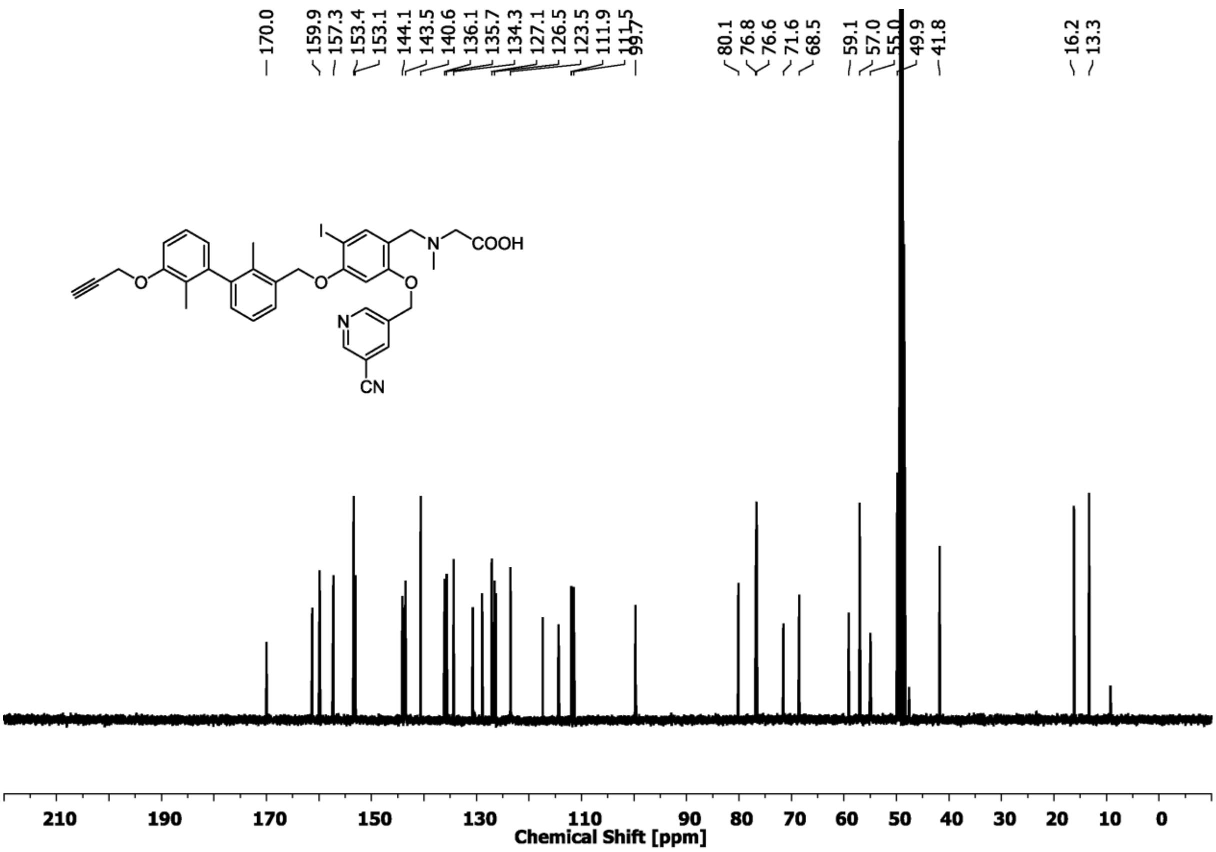

Figure S88: ^{13}C NMR spectrum (methanol-d_4, 101 MHz, 298 K) of compound 53c.

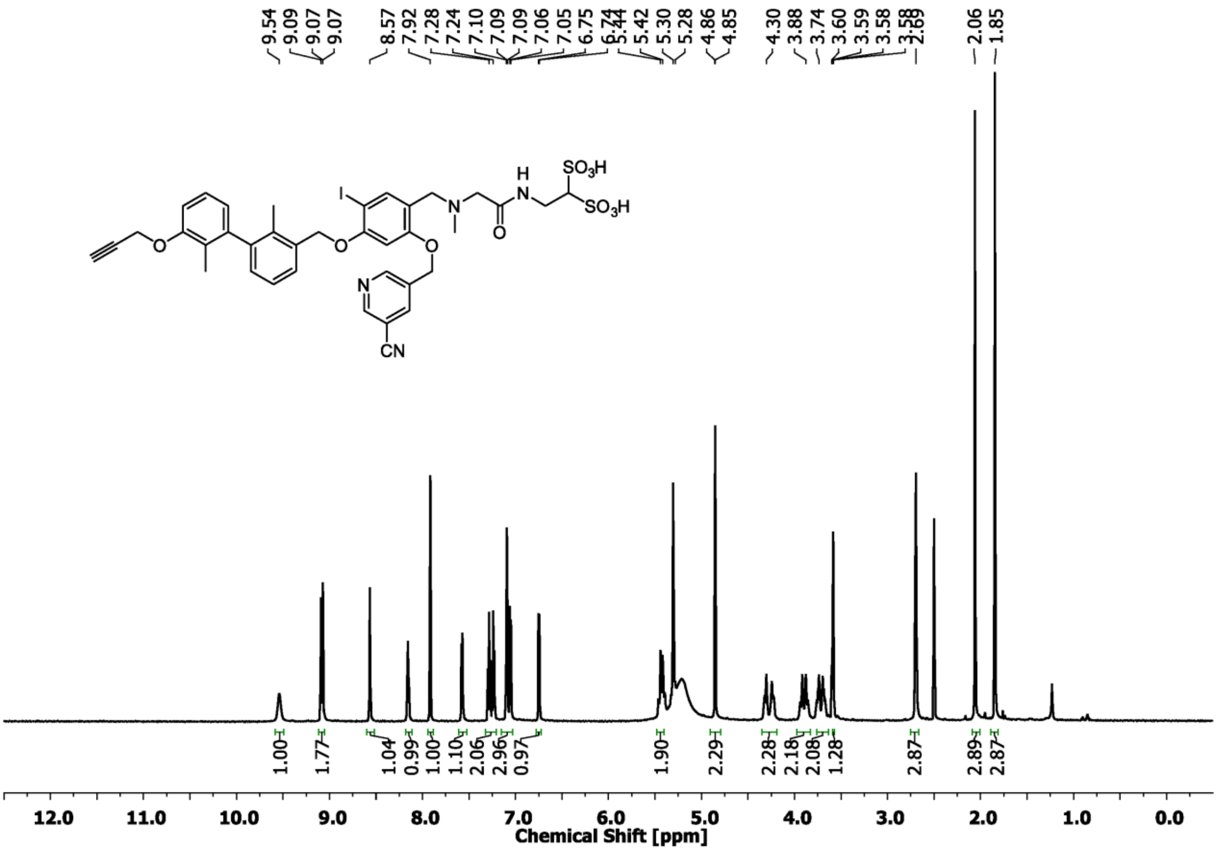

Figure S89: ^{1}H NMR spectrum (DMSO-d_6, 600 MHz, 298 K) of compound 54c.

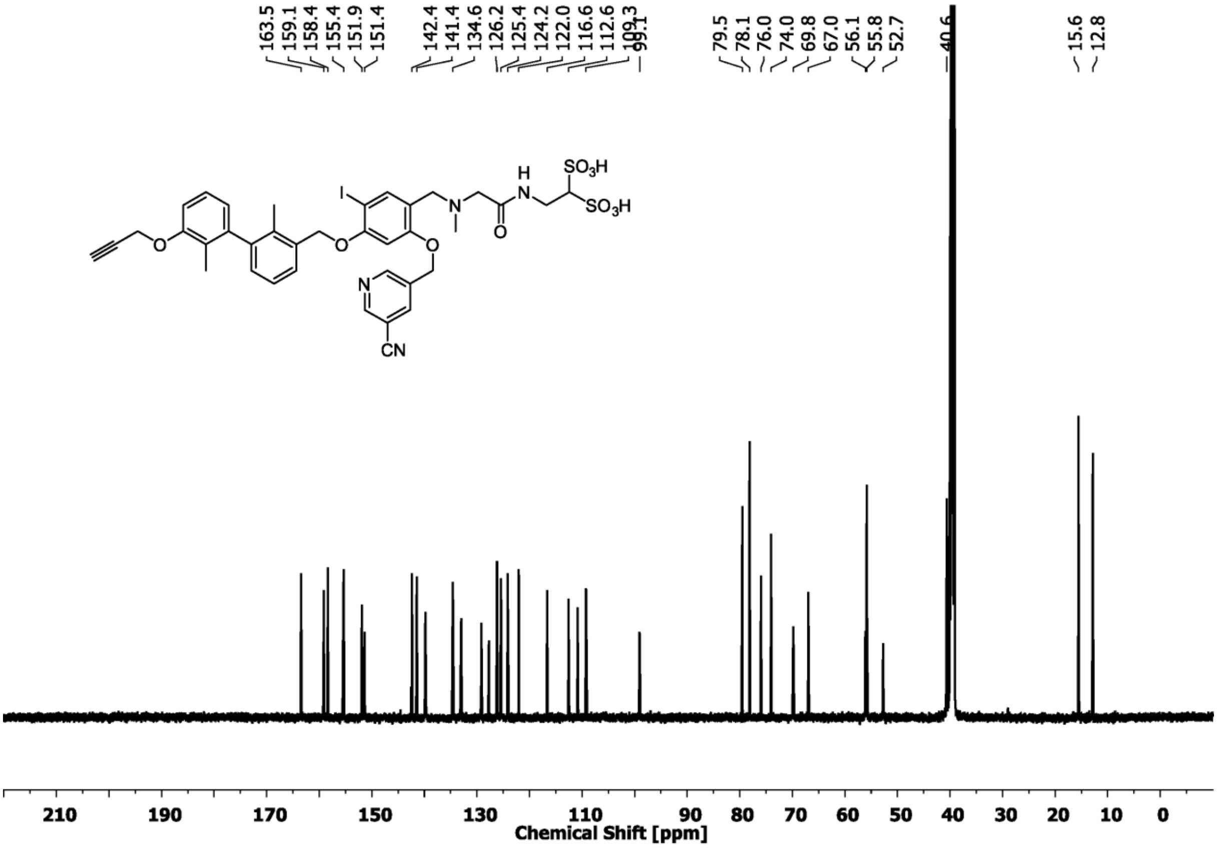

Figure S90: ^{13}C NMR spectrum (DMSO-d_6, 151 MHz, 298 K) of compound 54c.

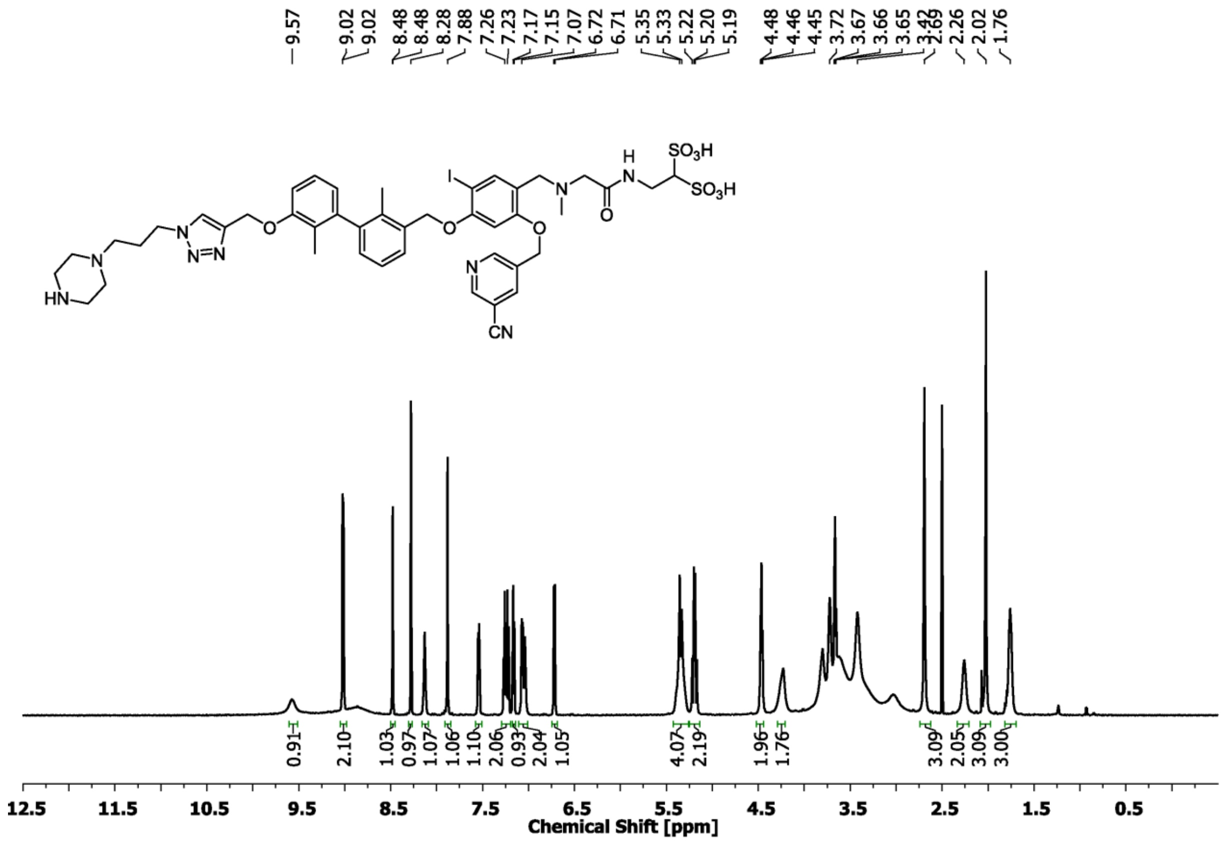

Figure S91: **^{1}H NMR spectrum (DMSO-d_6, 600 MHz, 298 K) of compound 55c.**

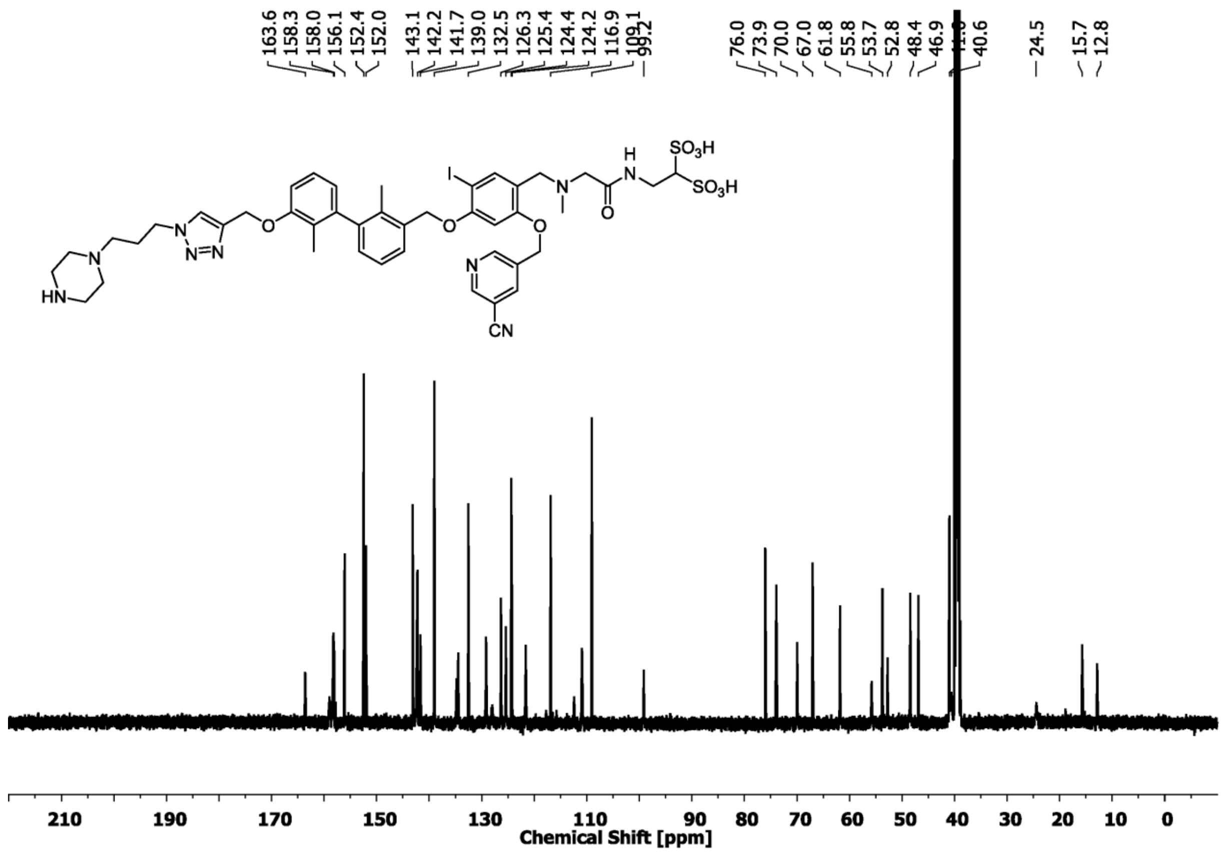

Figure S92: **^{13}C NMR spectrum (DMSO-d_6, 151 MHz, 298 K) of compound 55c.**

3. IR spectra of literature unknown compounds

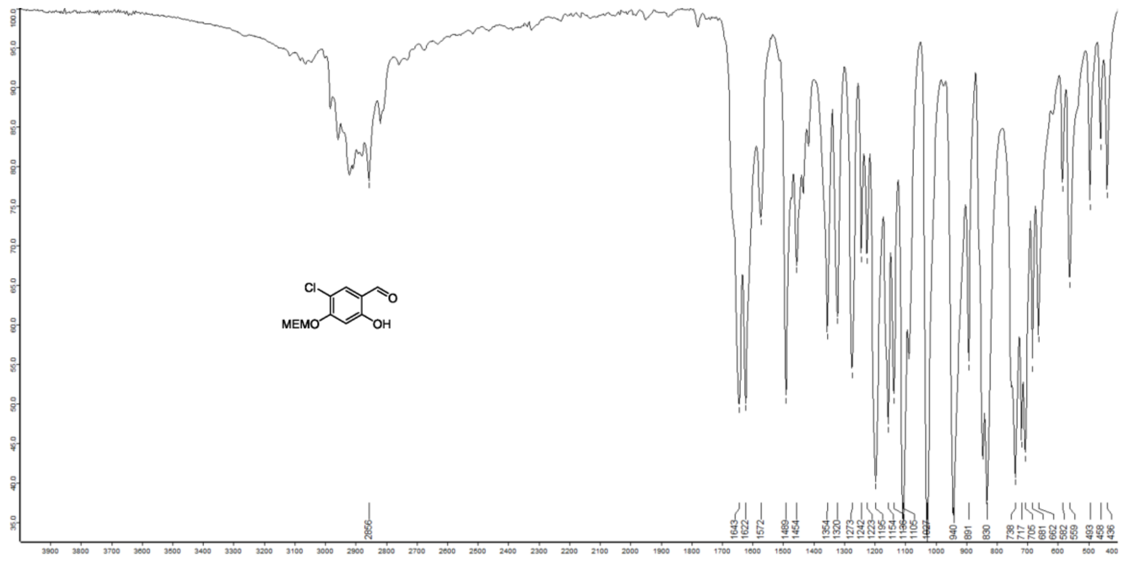

Figure S93: ATR-IR spectrum of compound 6.

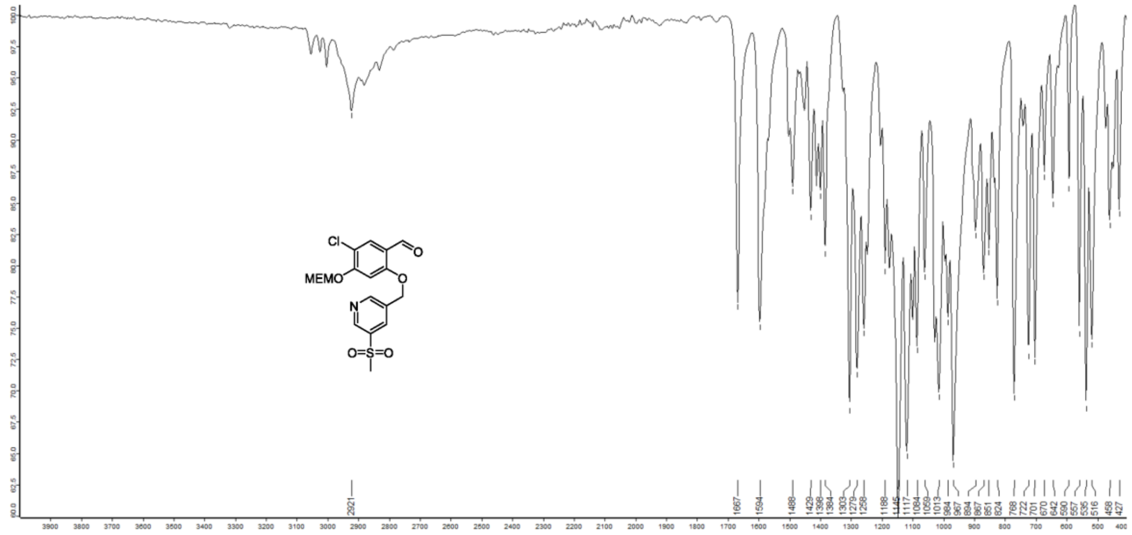

Figure S94: ATR-IR spectrum of compound 10.

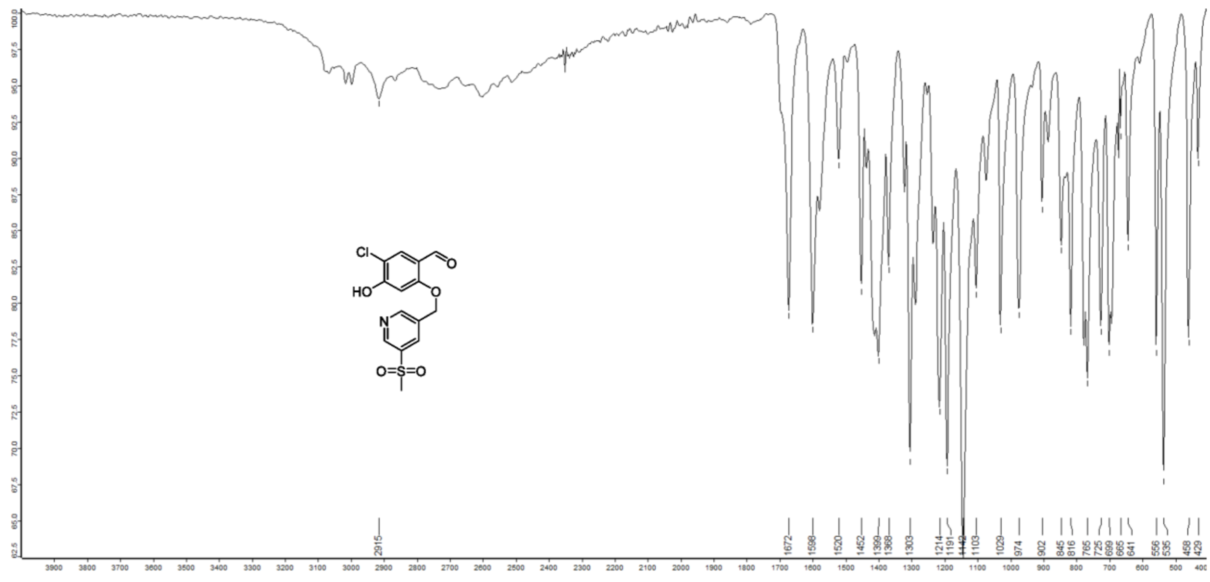

Figure S95: ATR-IR spectrum of compound 11.

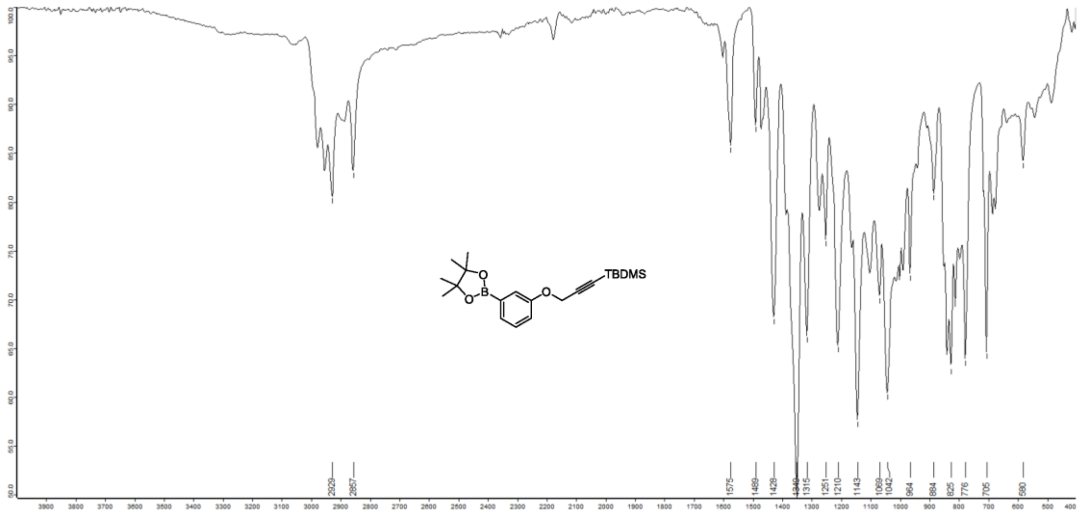

Figure S96: ATR-IR spectrum of compound 17.

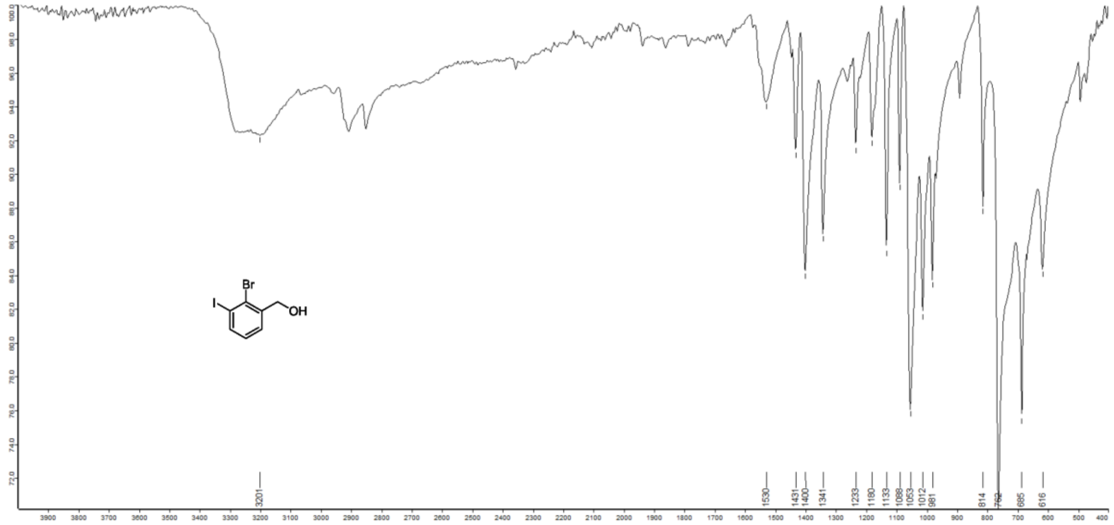

Figure S97: ATR-IR spectrum of compound 21.

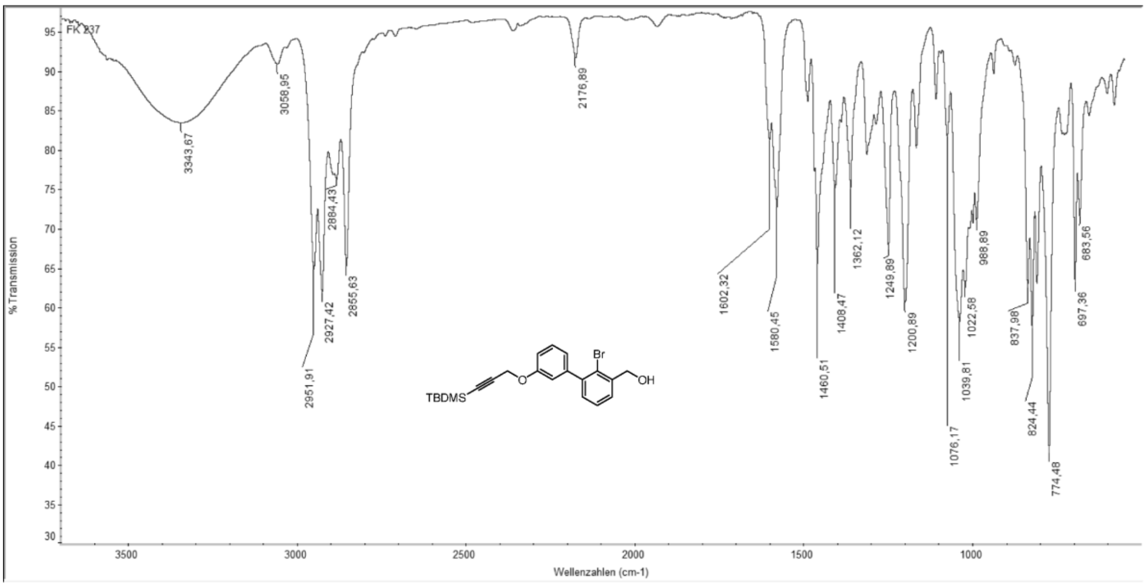

Figure S98: ATR-IR spectrum of compound 22.

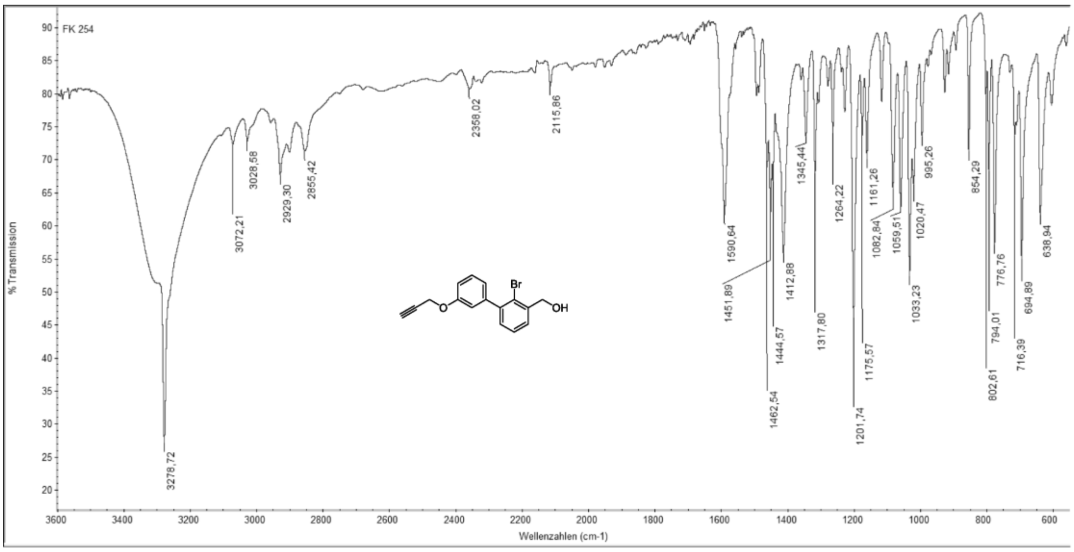

Figure S99: ATR-IR spectrum of compound 28a.

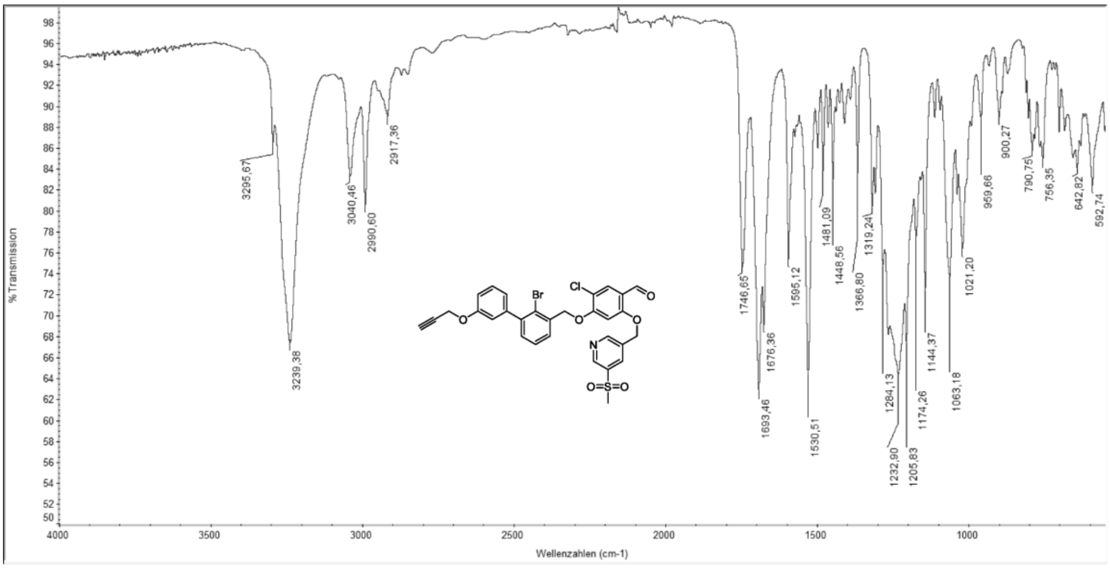

Figure S100: ATR-IR spectrum of compound 29a.

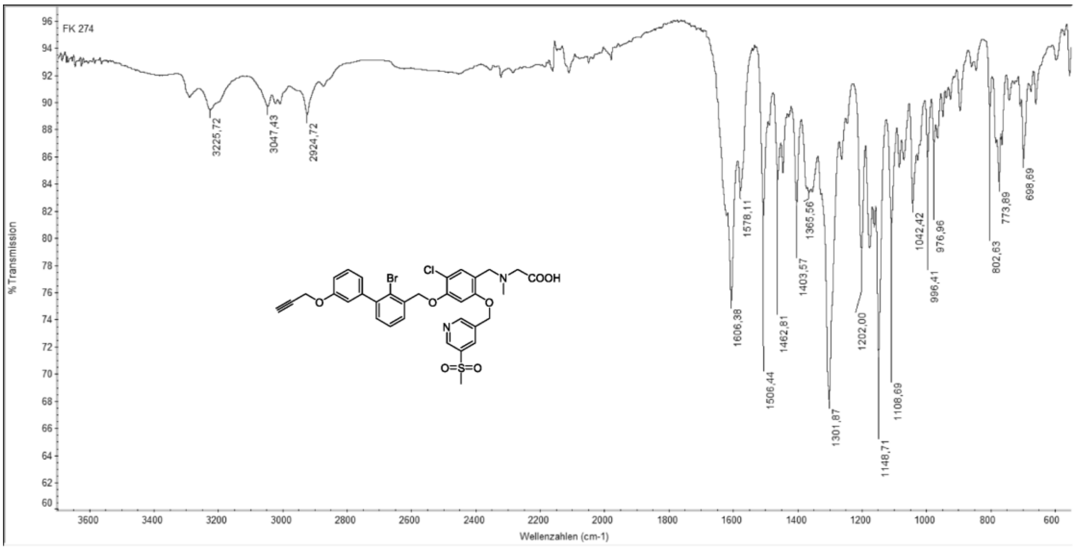

Figure S101: ATR-IR spectrum of compound 30a.

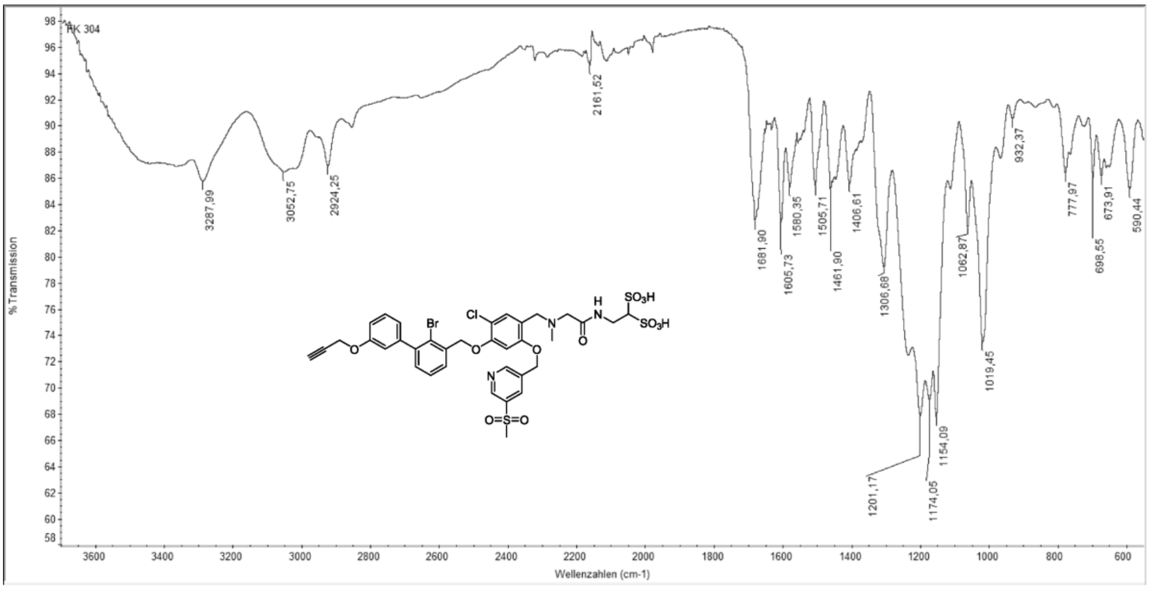

Figure S102: ATR-IR spectrum of compound 33a.

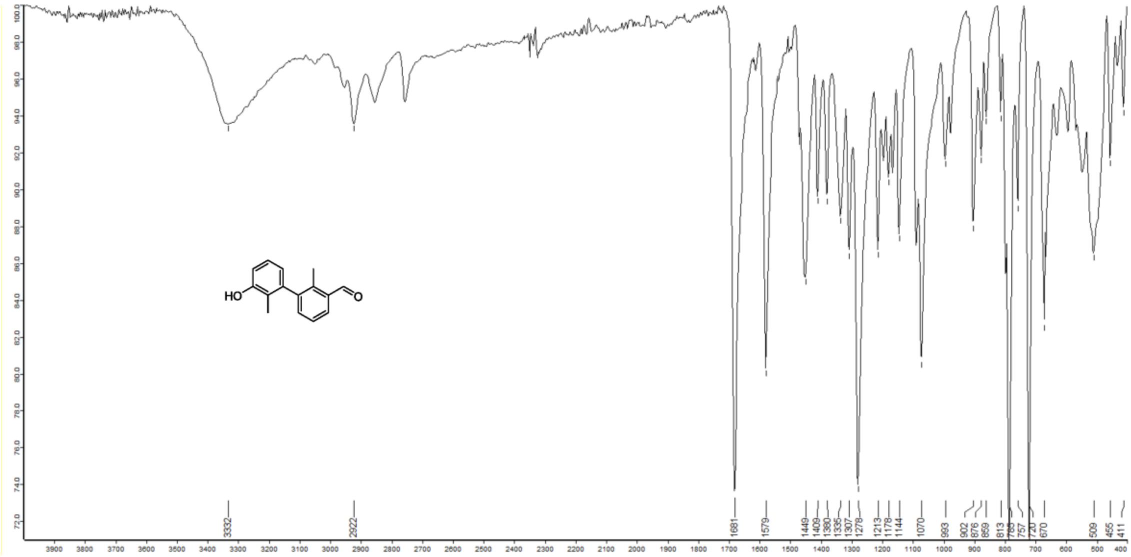

Figure S103: ATR-IR spectrum of compound 26.

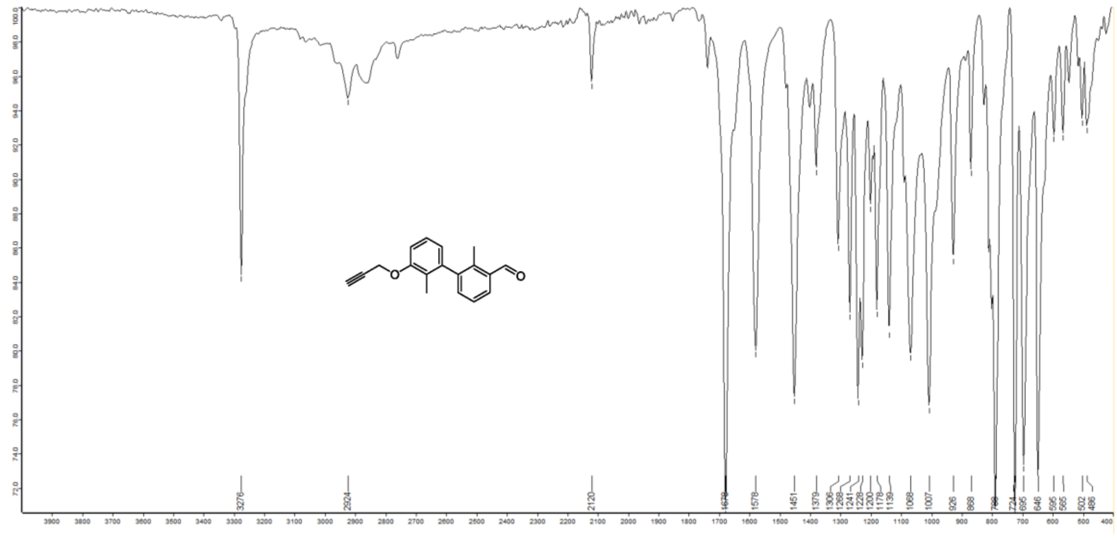

Figure S104: ATR-IR spectrum of compound 27.

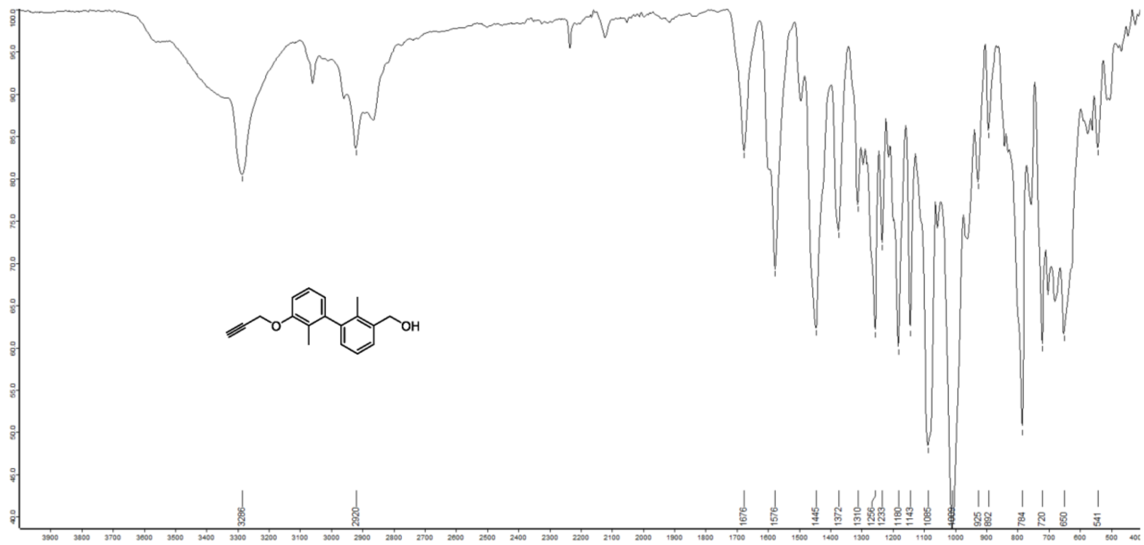

Figure S105: ATR-IR spectrum of compound 28b.

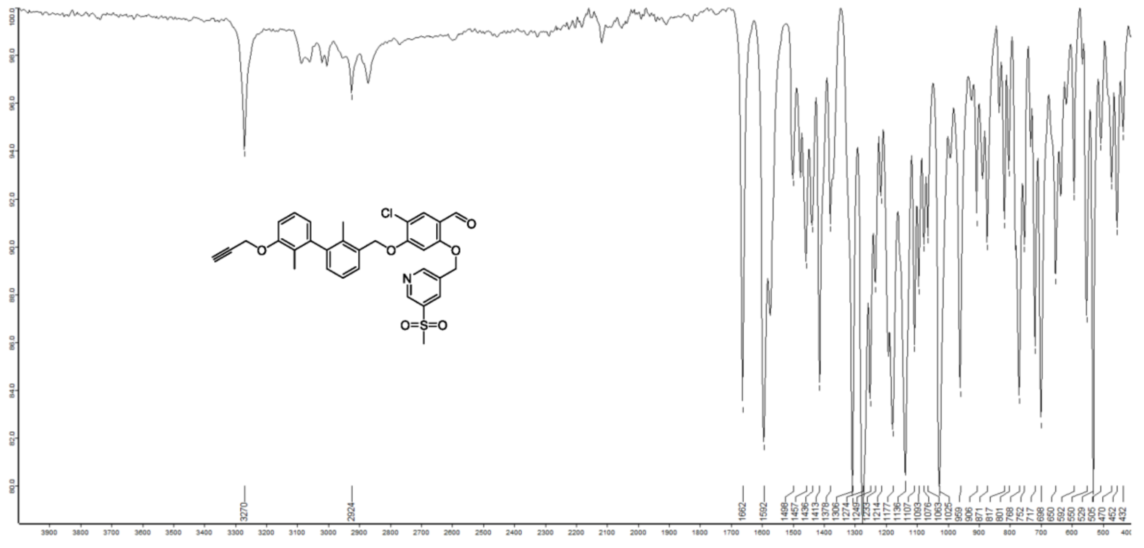

Figure S106: ATR-IR spectrum of compound 29b.

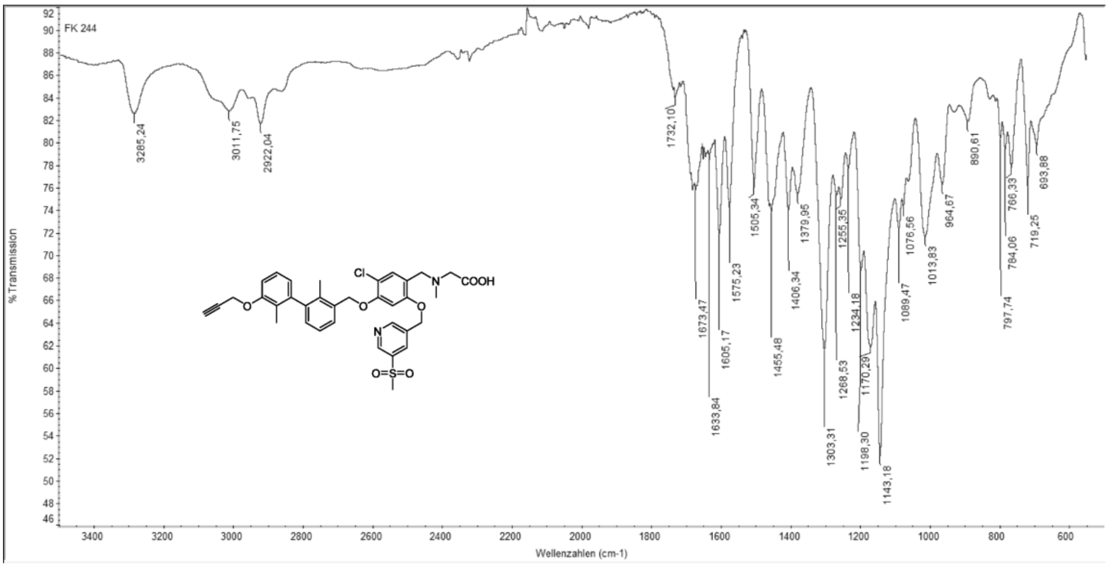

Figure S107: ATR-IR spectrum of compound 30b.

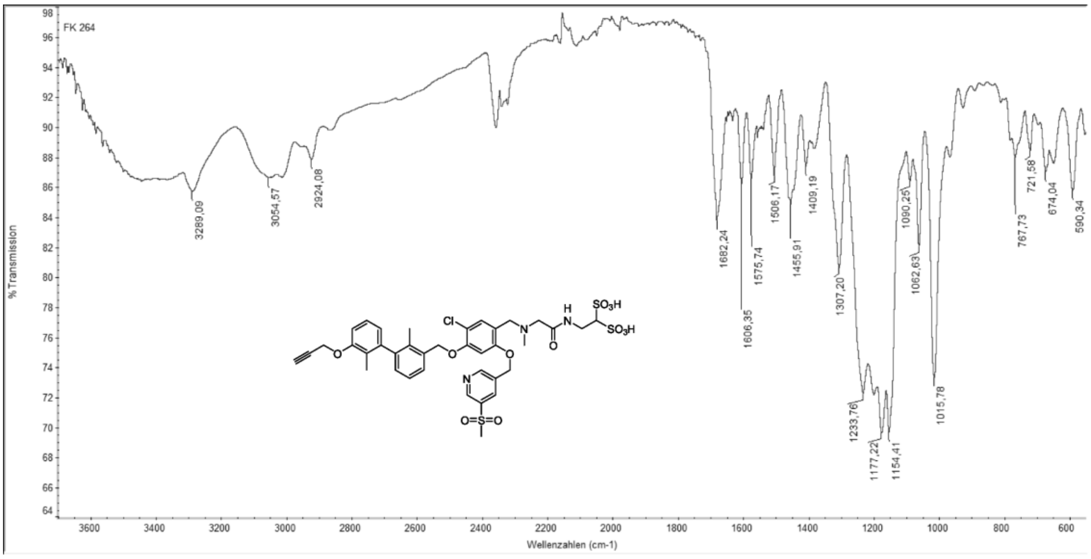

Figure S108: ATR-IR spectrum of compound 33b.

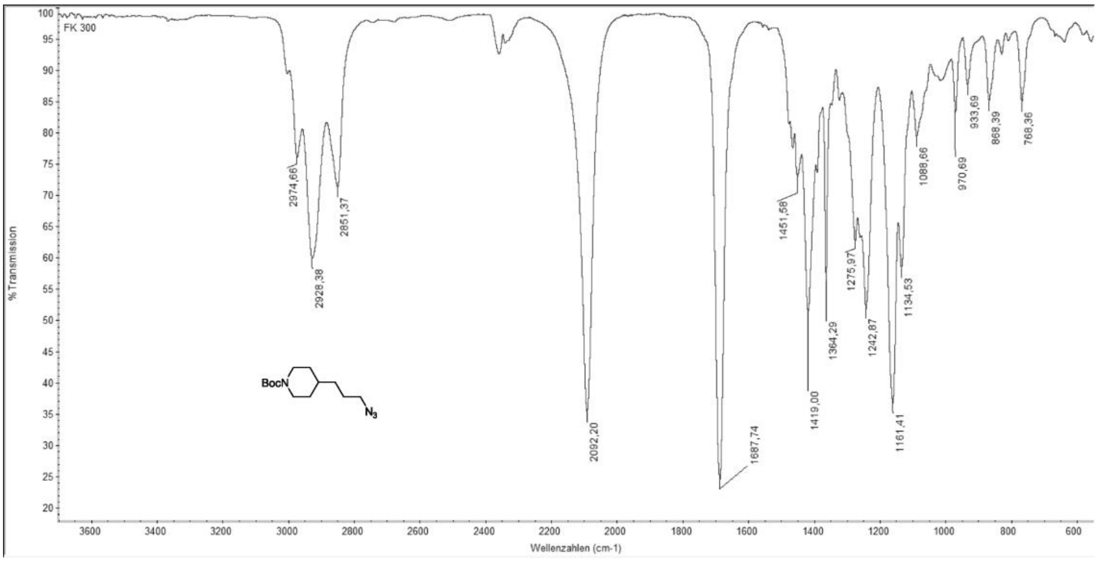

Figure S109: ATR-IR spectrum of compound S-5.

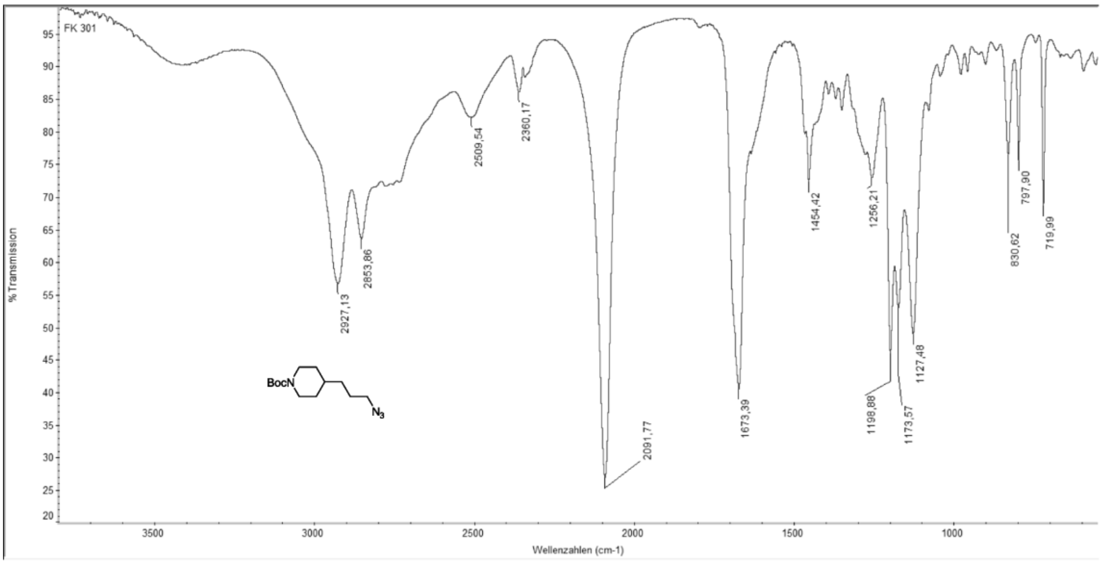

Figure S110: ATR-IR spectrum of compound S-6.

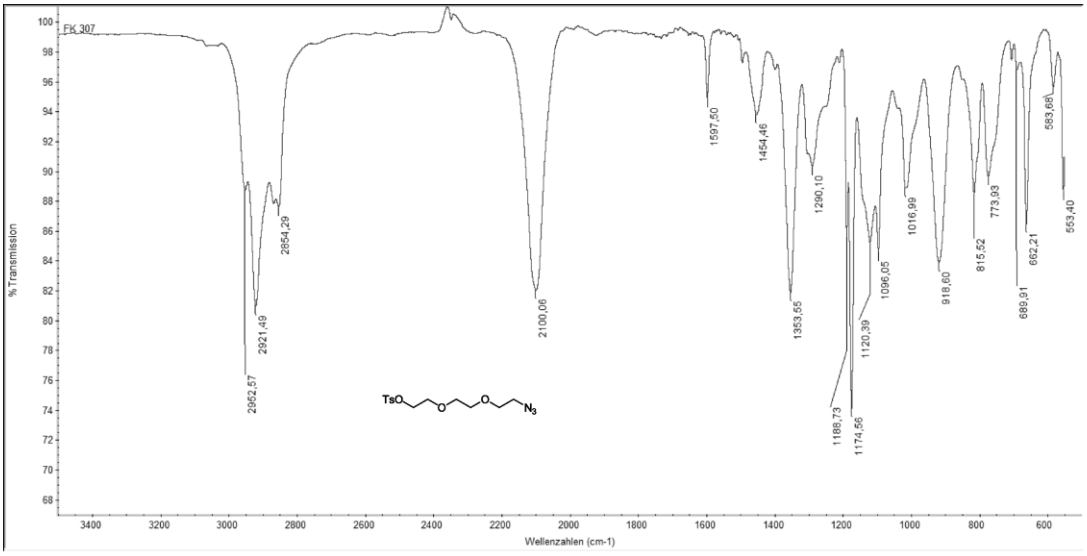

Figure S111: ATR-IR spectrum of compound S-7.

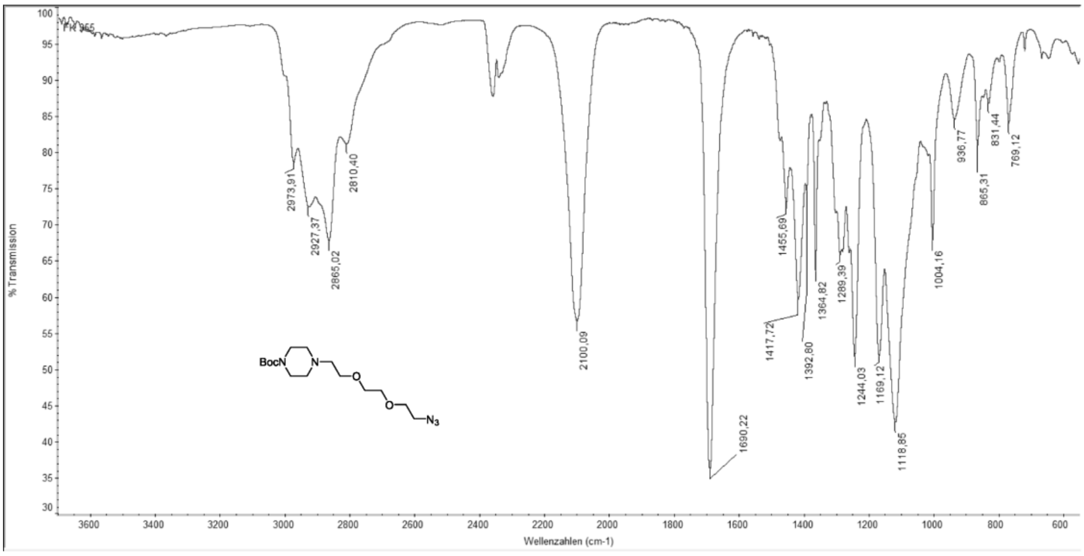

Figure S112: ATR-IR spectrum of compound S-8.

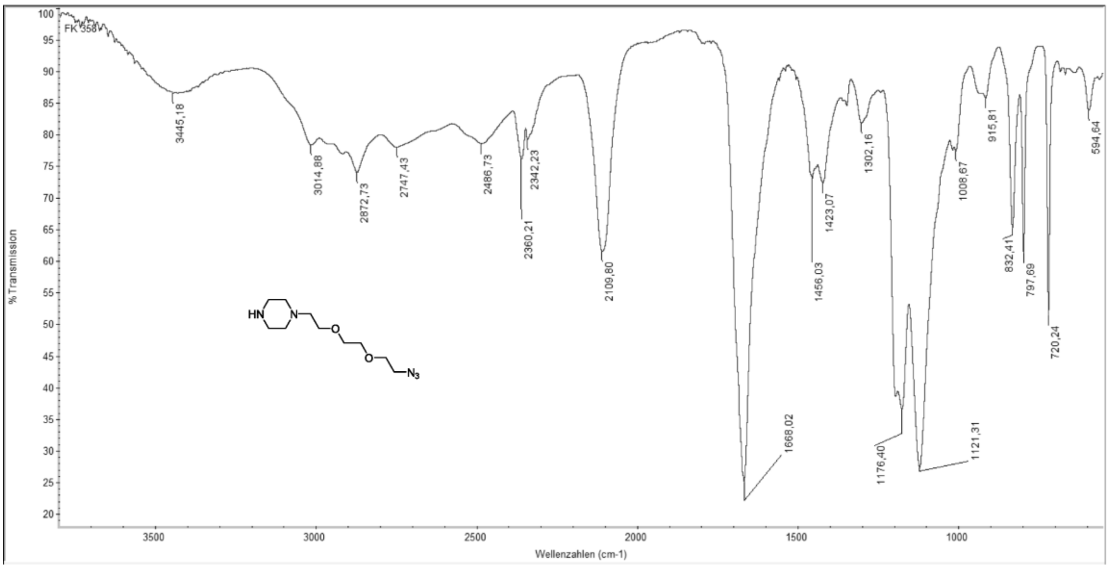

Figure S113: ATR-IR spectrum of compound 39.

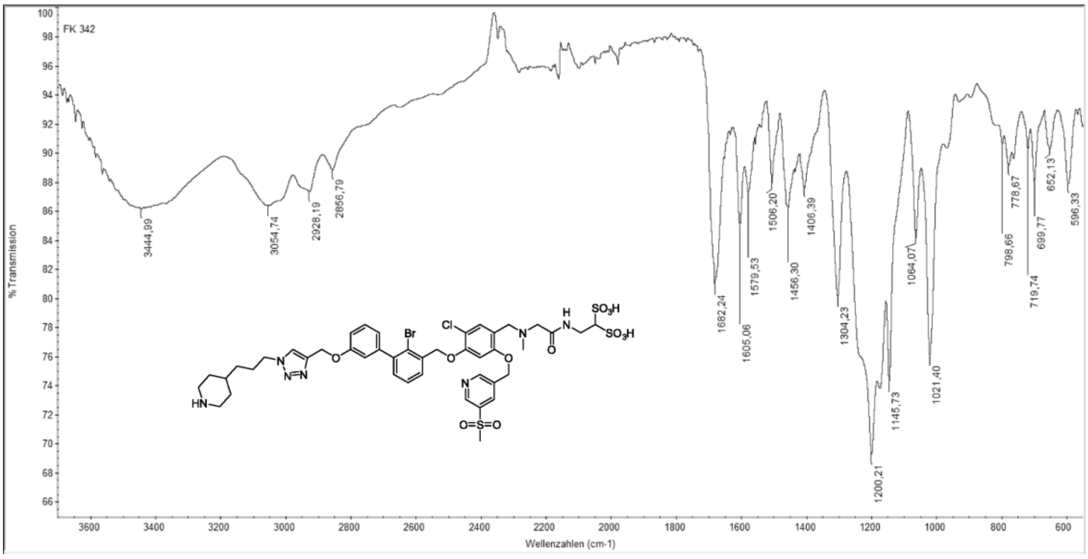

Figure S114: ATR-IR spectrum of compound 40a.

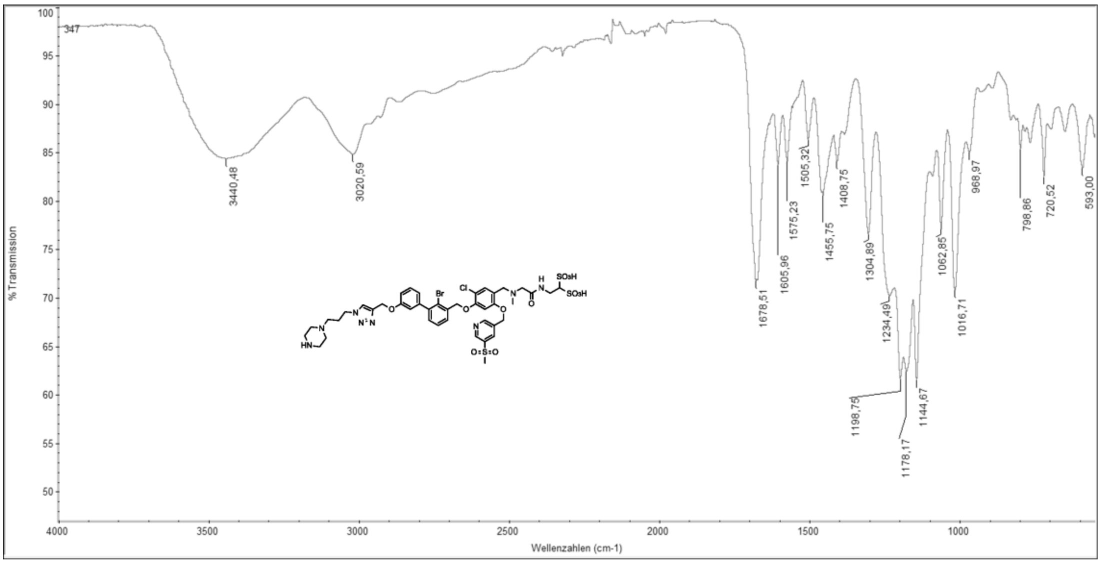

Figure S115: ATR-IR spectrum of compound 40b.

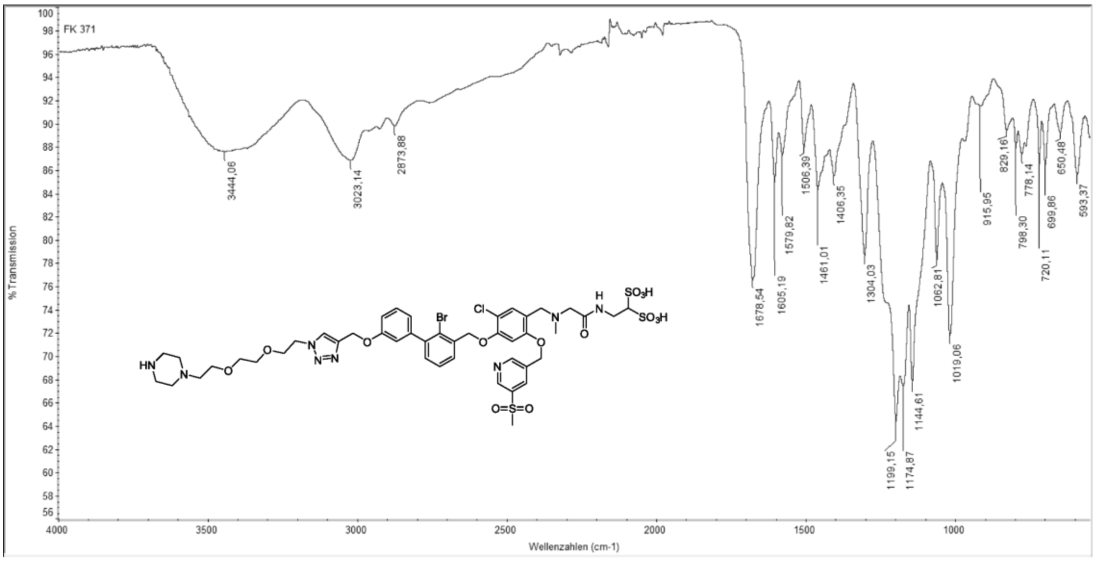

Figure S116: ATR-IR spectrum of compound 40c.

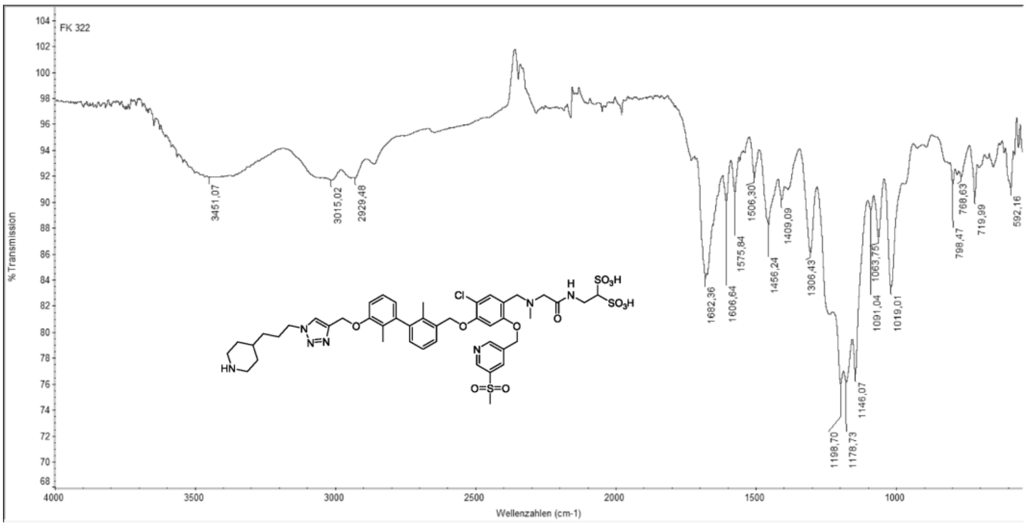

Figure S117: ATR-IR spectrum of compound 41a.

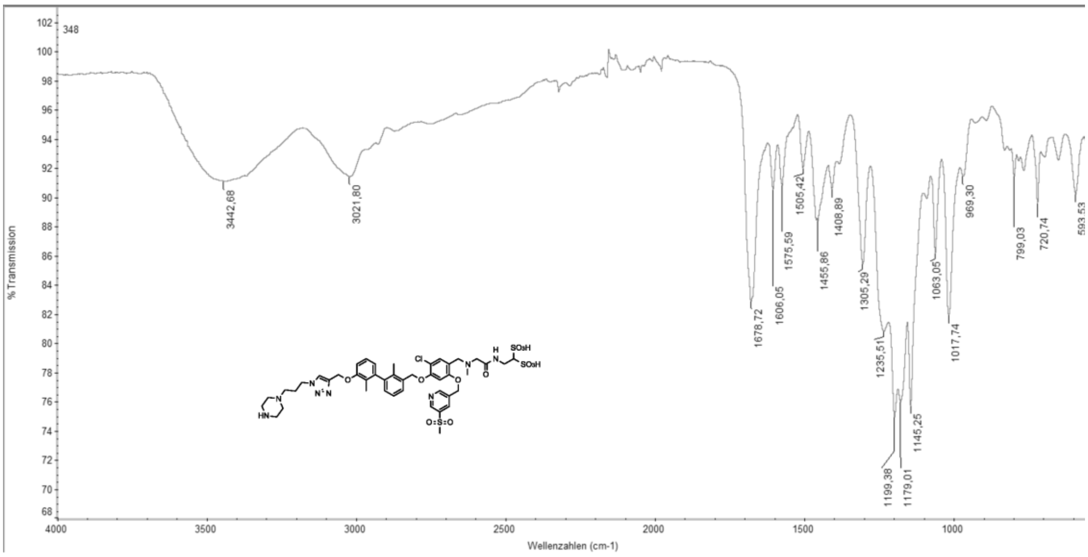

Figure S118: ATR-IR spectrum of compound 41b.

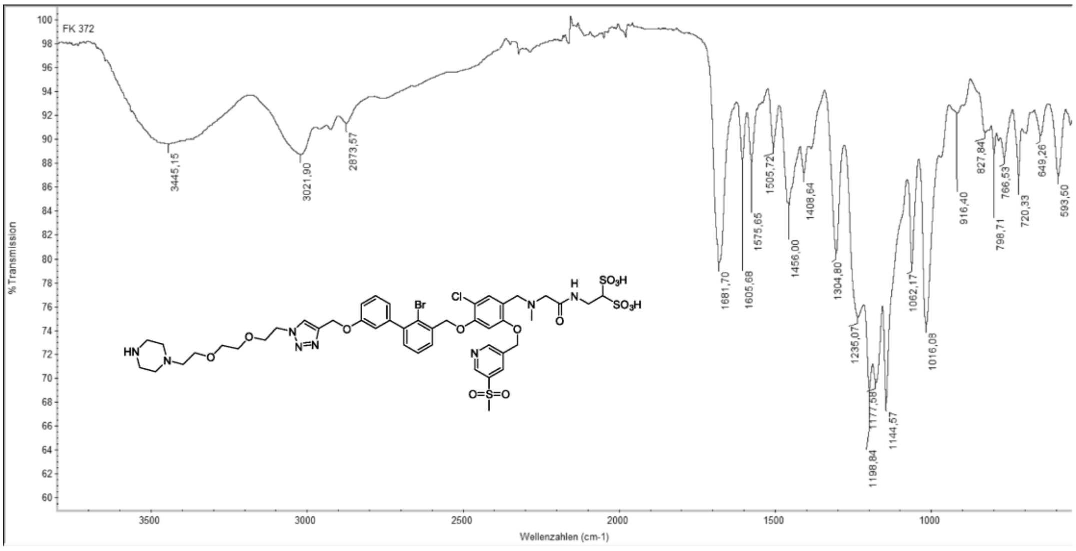

Figure S119: ATR-IR spectrum of compound 41c.

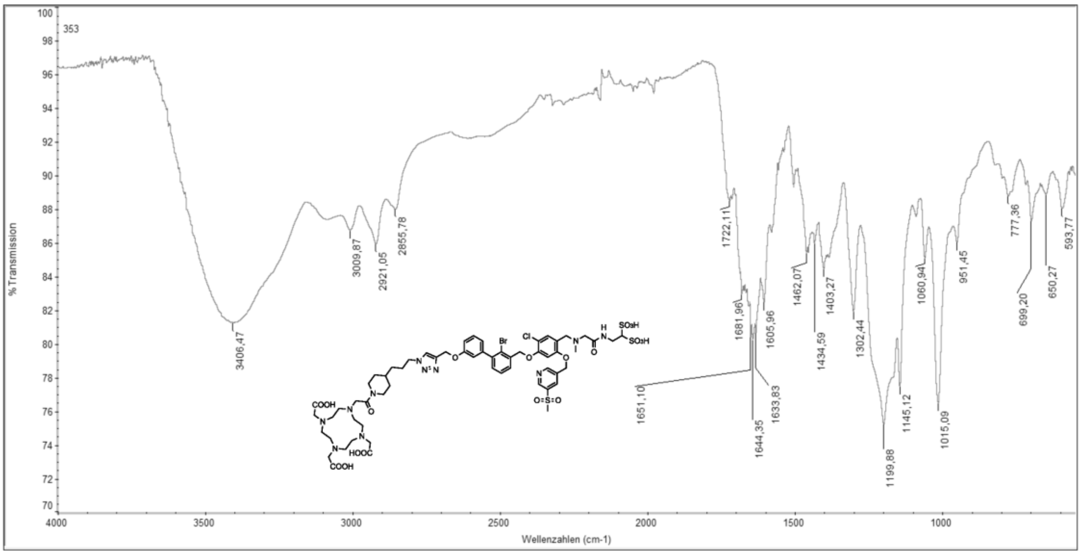

Figure S120: ATR-IR spectrum of compound 42a.

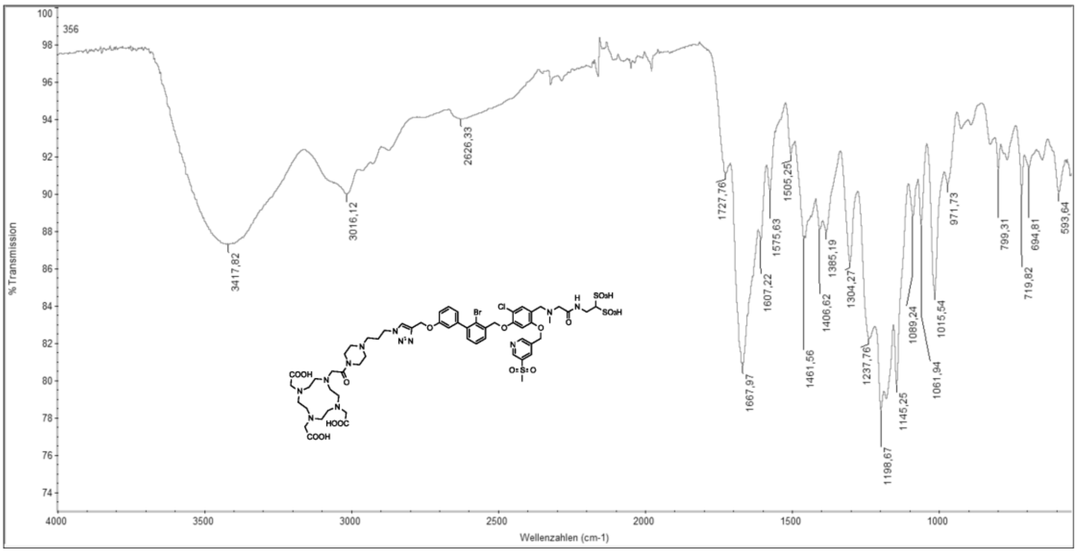

Figure S121: ATR-IR spectrum of compound 42b.

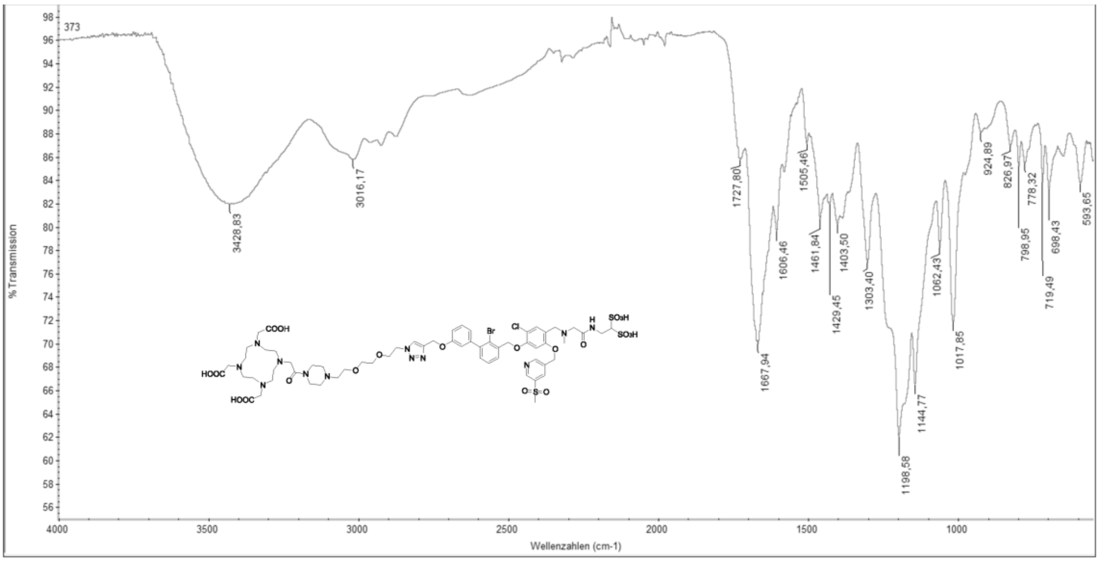

Figure S122: ATR-IR spectrum of compound 42c.

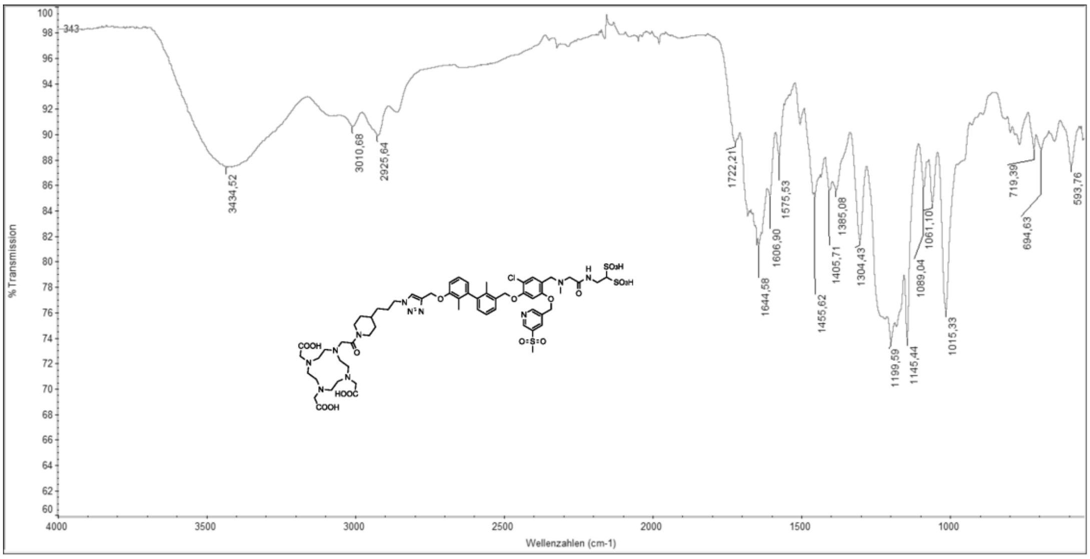

Figure S123: ATR-IR spectrum of compound 43a.

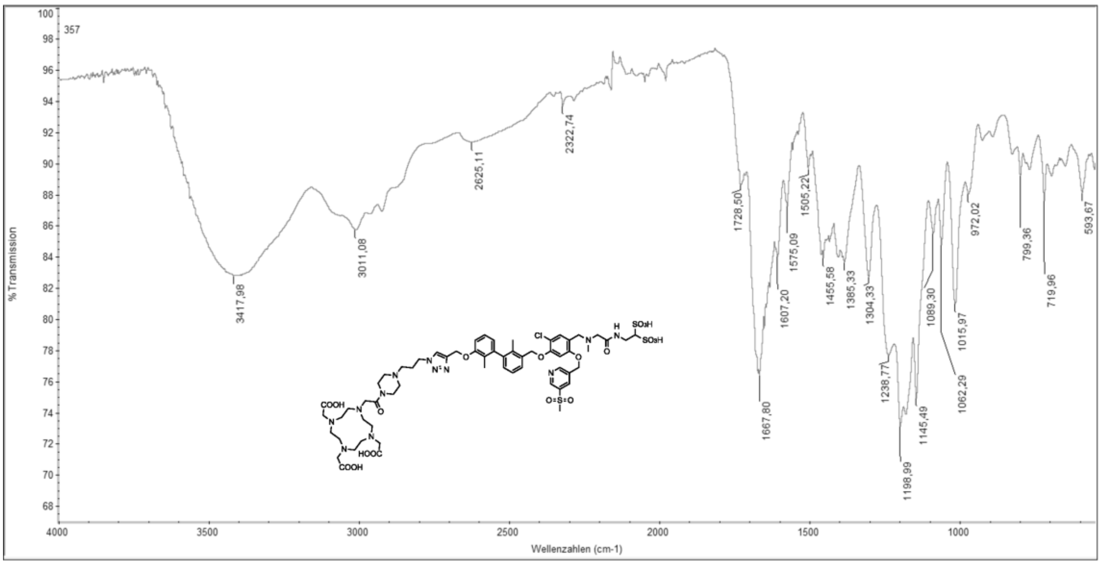

Figure S124: ATR-IR spectrum of compound 43b.

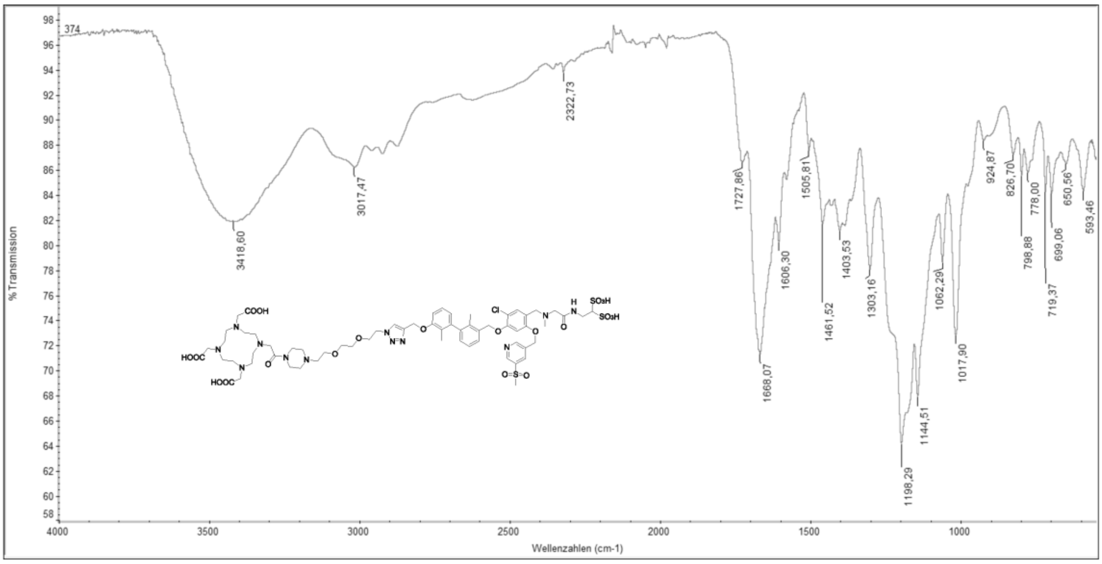

Figure S125: ATR-IR spectrum of compound 43c.

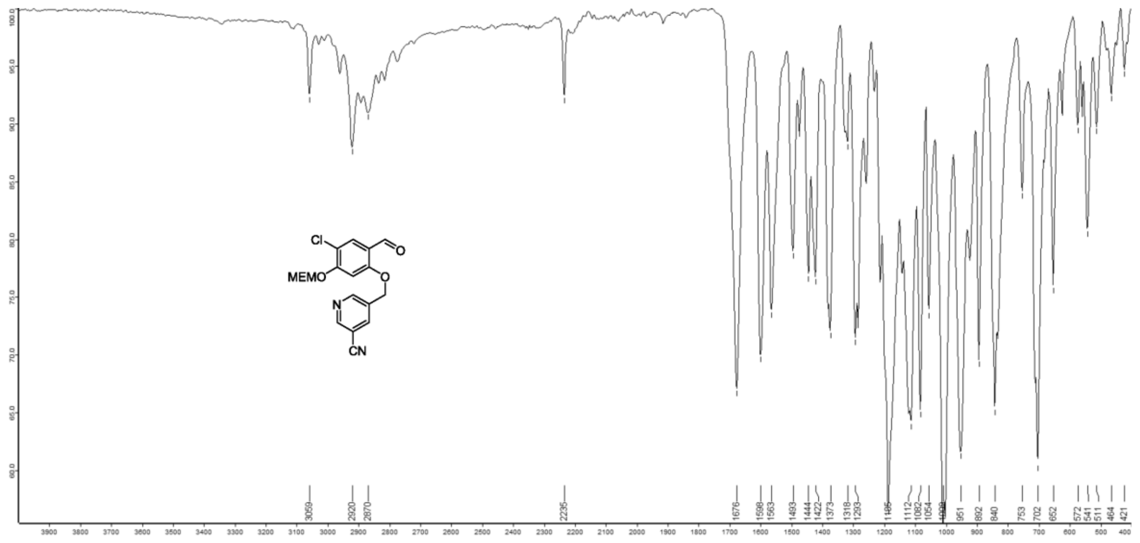

Figure S126: ATR-IR spectrum of compound 46a.

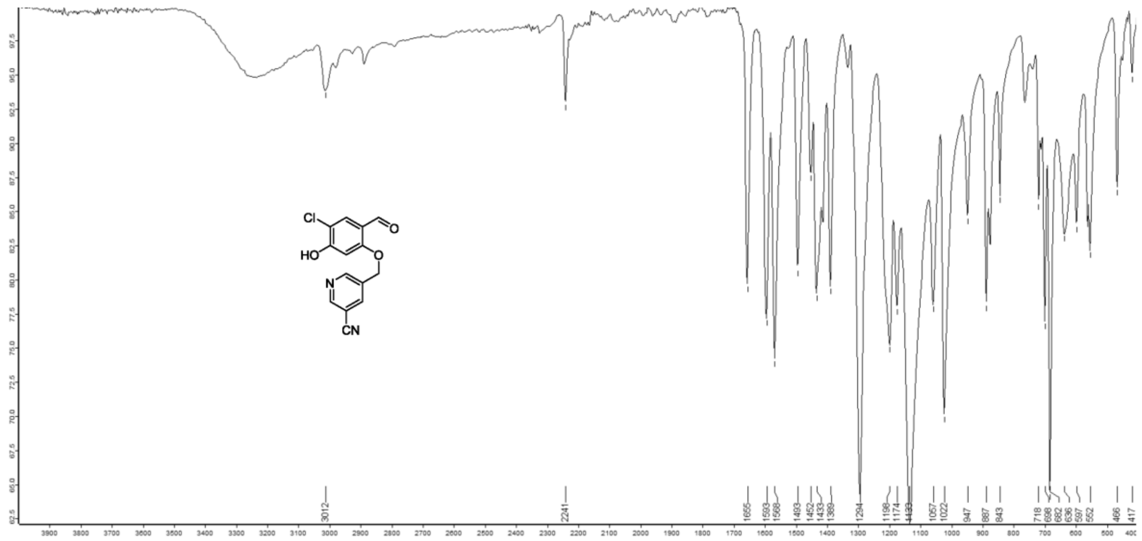

Figure S127: ATR-IR spectrum of compound 51a.

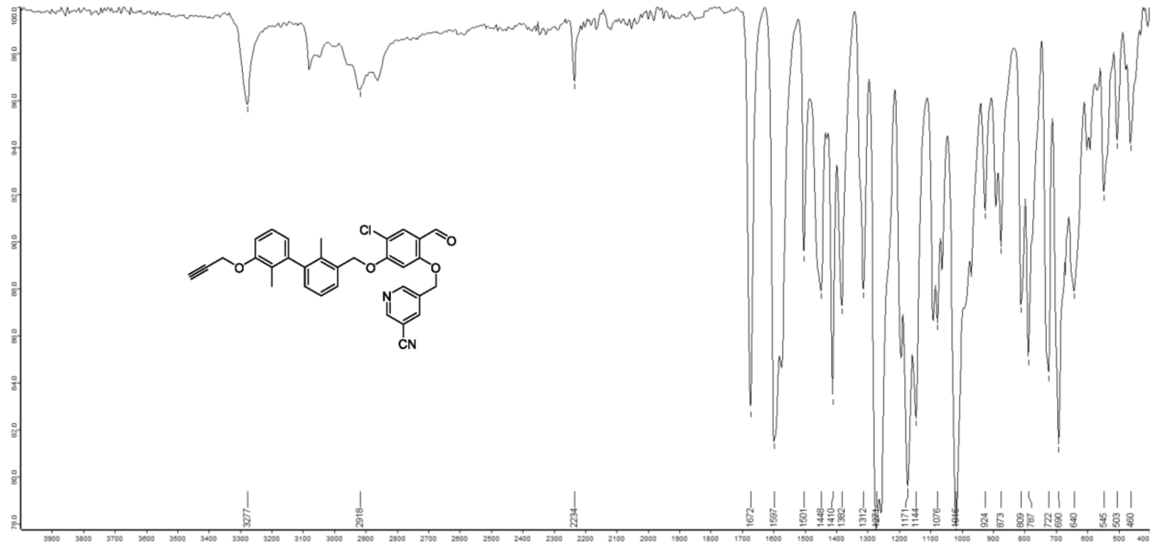

Figure S128: ATR-IR spectrum of compound 52a.

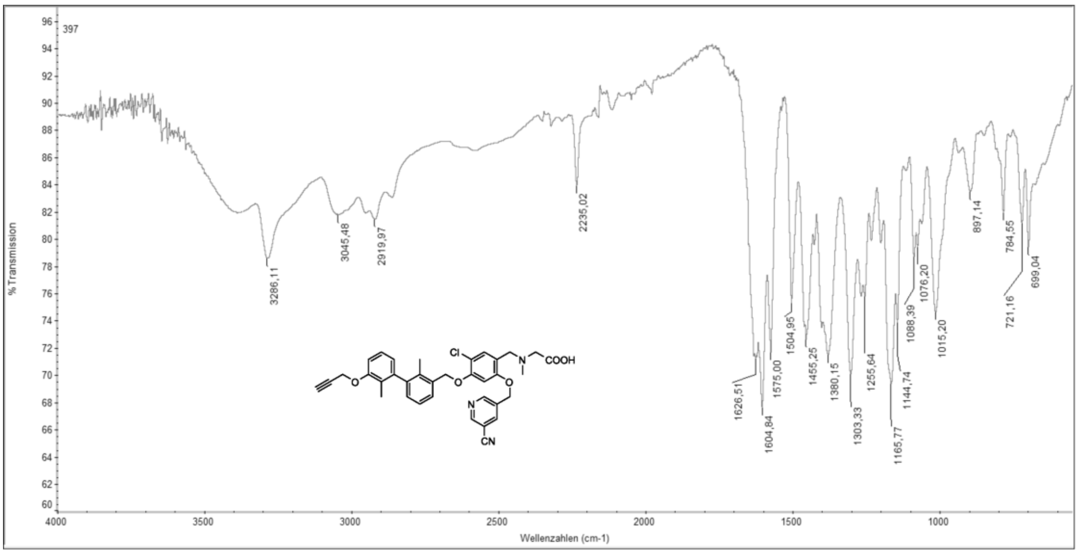

Figure S129: ATR-IR spectrum of compound 53a.

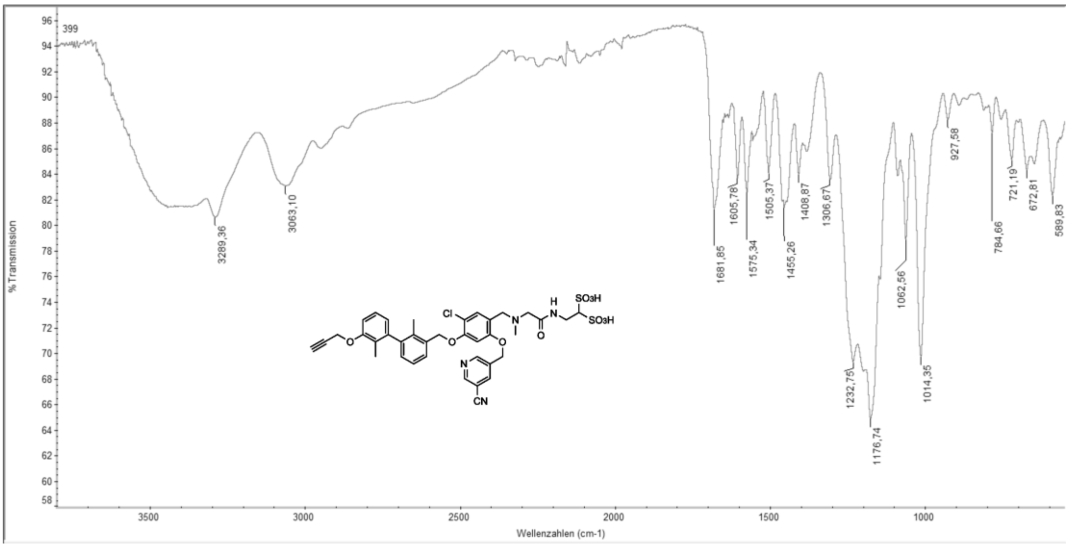

Figure S130: ATR-IR spectrum of compound 54a.

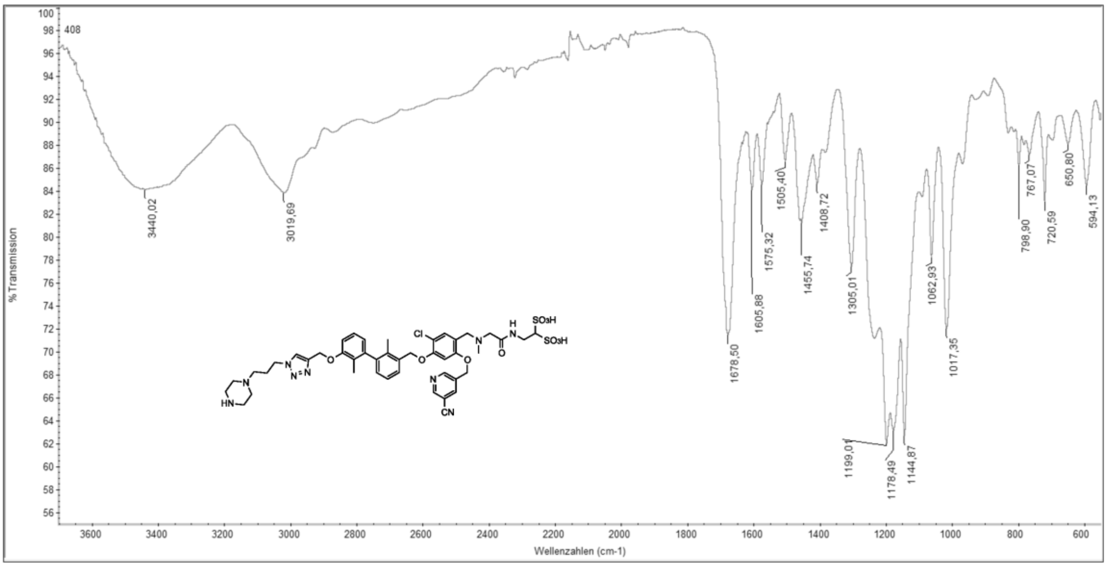

Figure S131: ATR-IR spectrum of compound 55a.

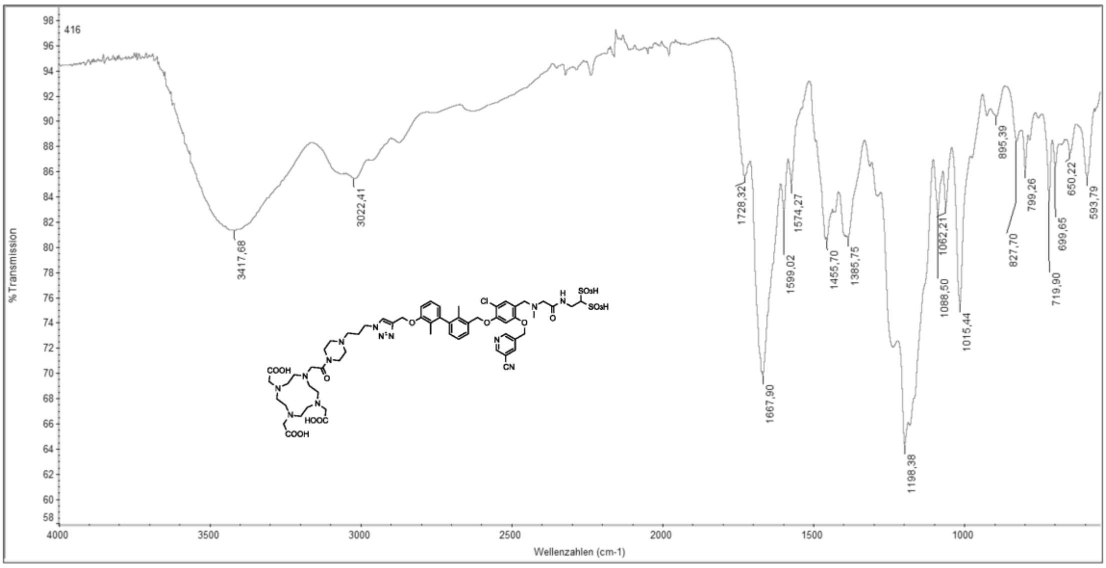

Figure S132: ATR-IR spectrum of compound 56a.

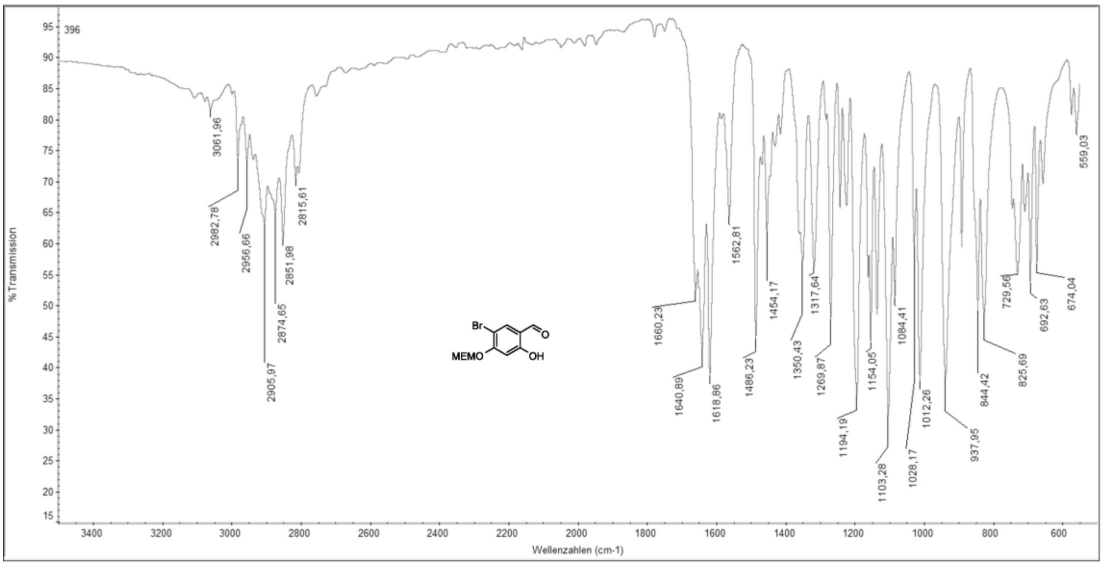

Figure S133: ATR-IR spectrum of compound 45.

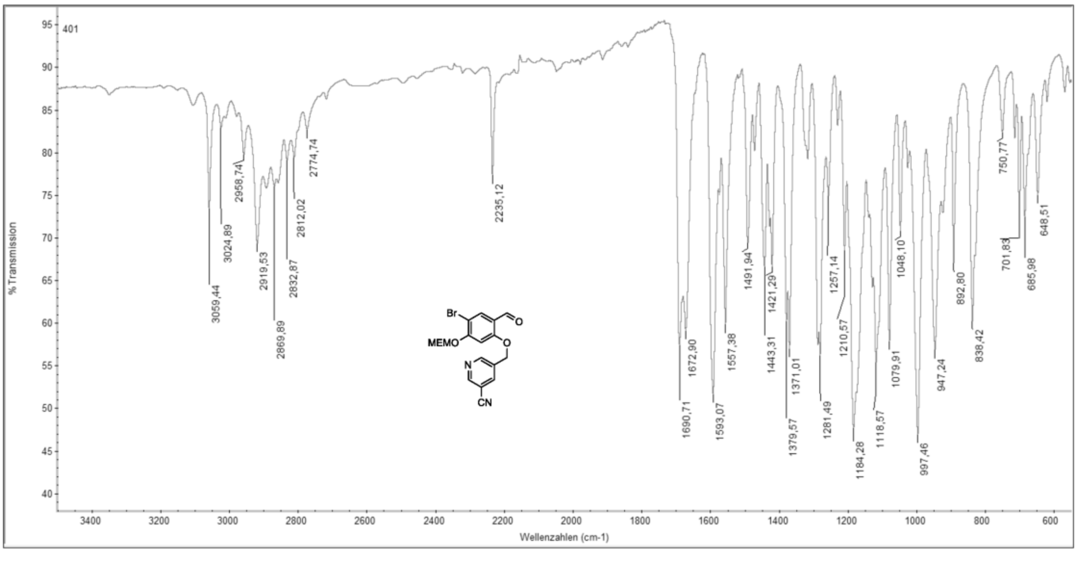

Figure S134: ATR-IR spectrum of compound 46b.

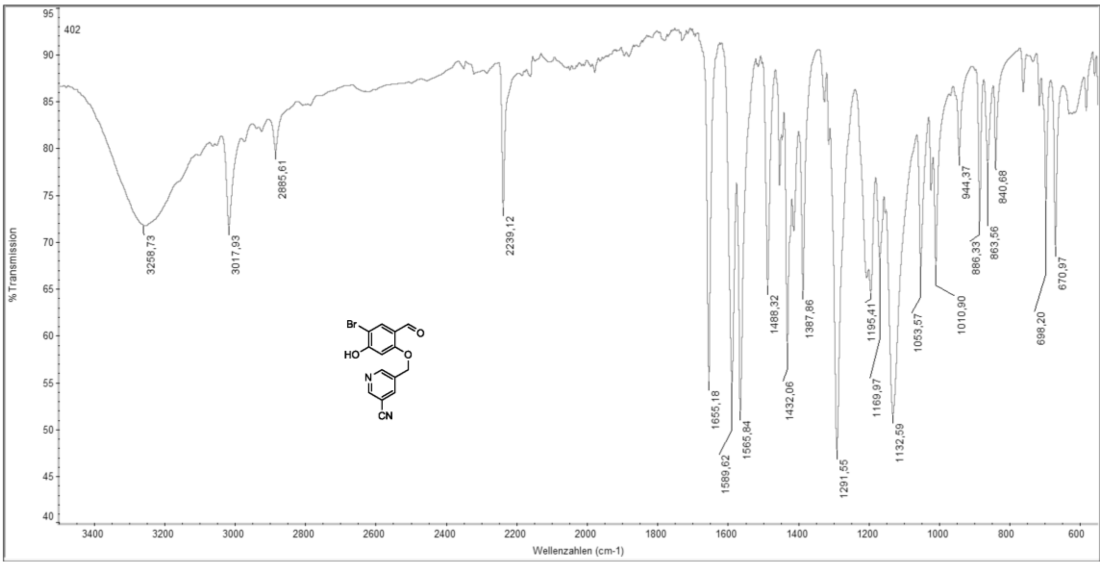

Figure S135: ATR-IR spectrum of compound 51b.

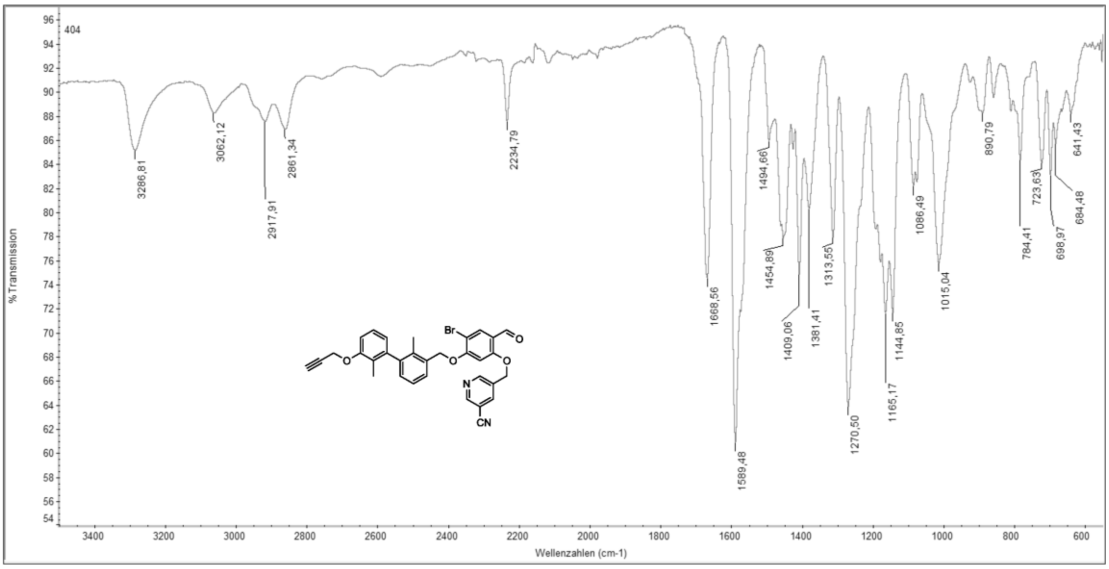

Figure S136: ATR-IR spectrum of compound 52b.

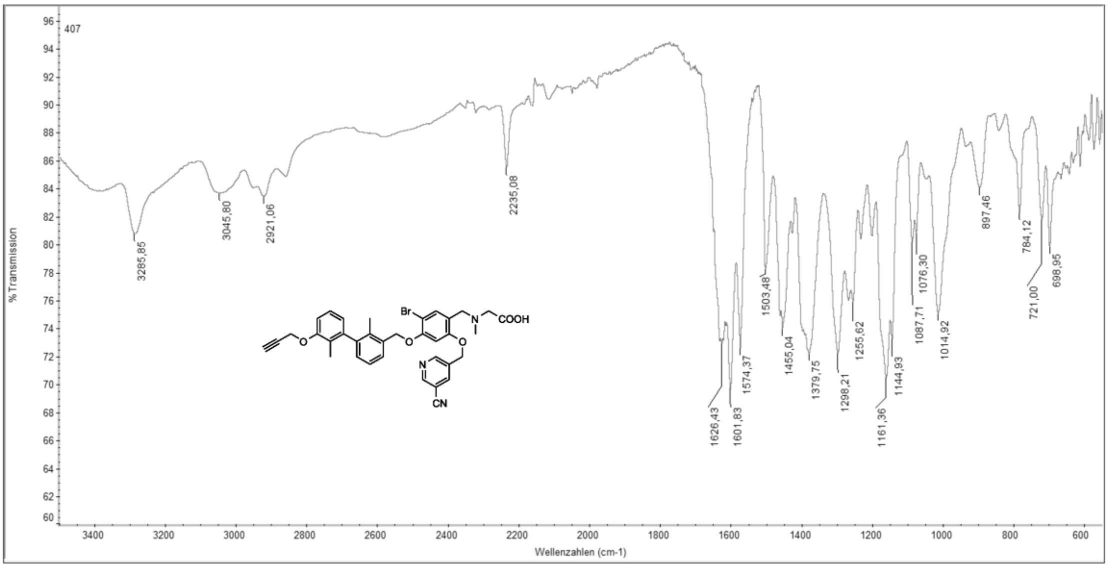

Figure S137: ATR-IR spectrum of compound 53b.

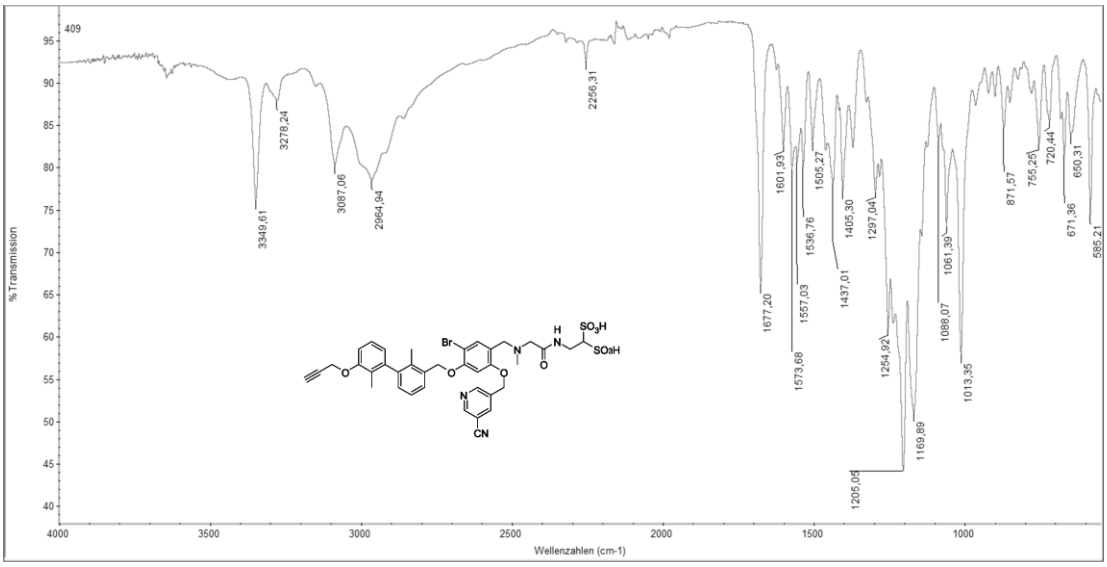

Figure S138: ATR-IR spectrum of compound 54b.

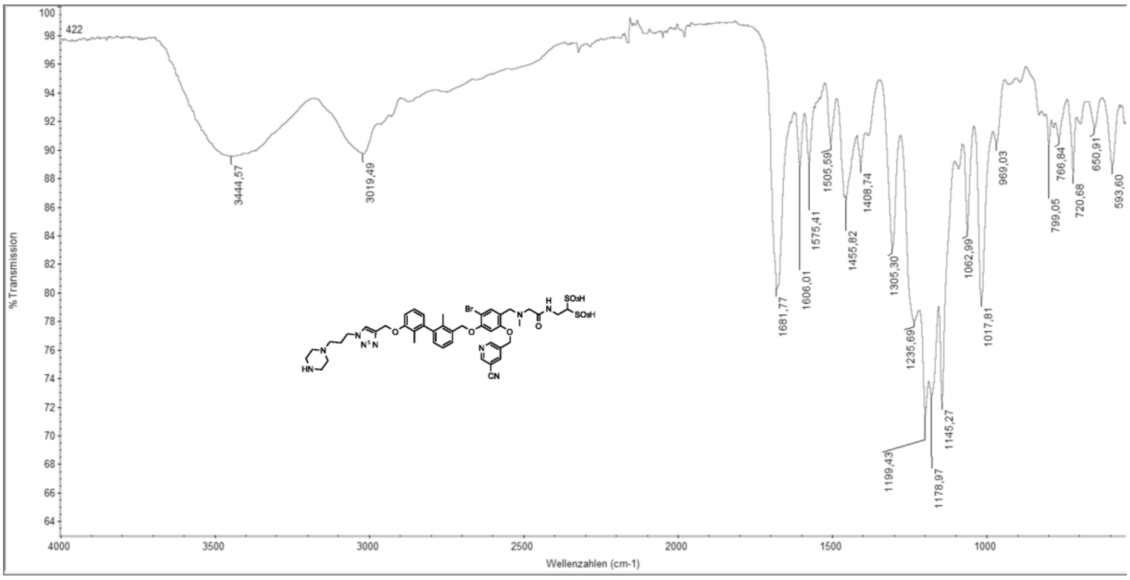

Figure S139: ATR-IR spectrum of compound 55b.

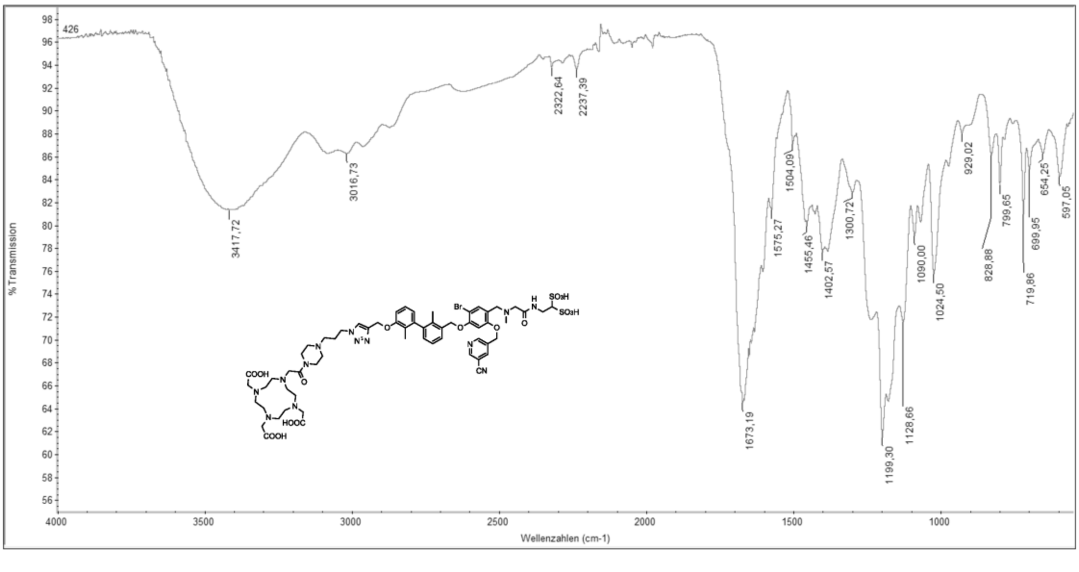

Figure S140: ATR-IR spectrum of compound 56b.

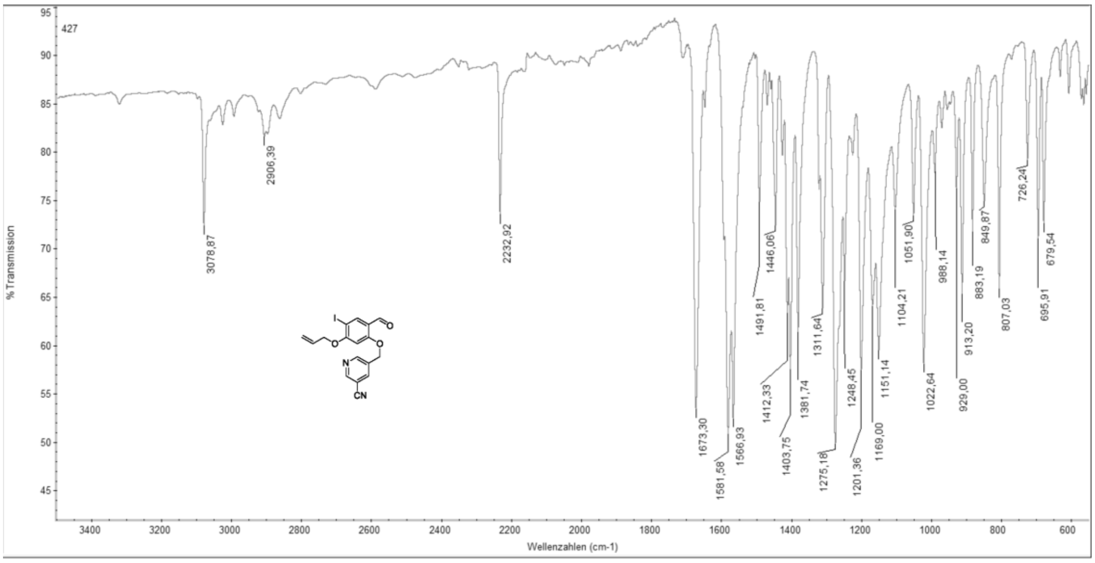

Figure S141: ATR-IR spectrum of compound 50.

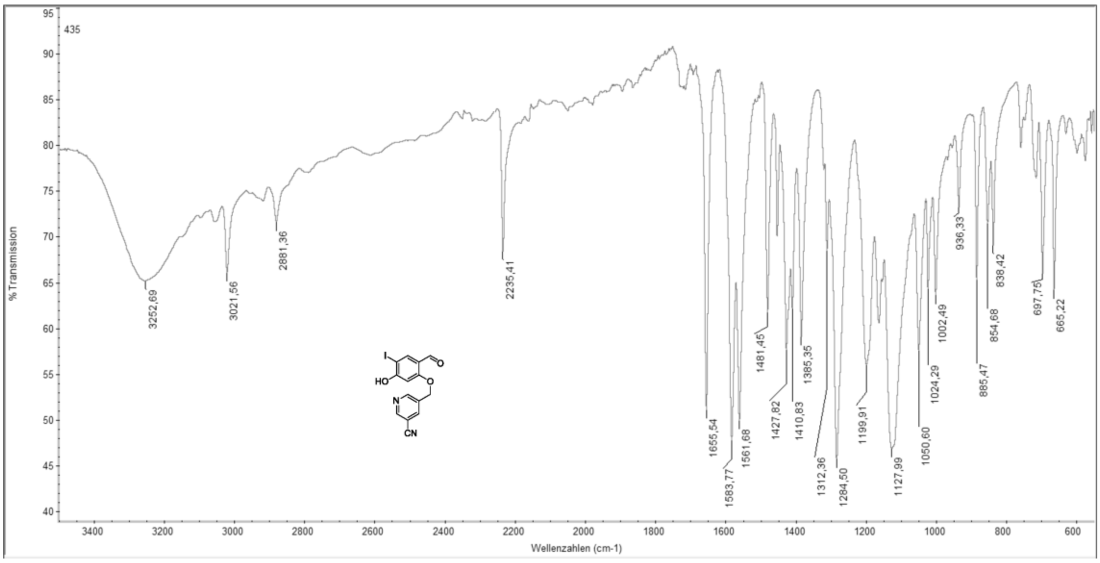

Figure S142: ATR-IR spectrum of compound 51c.

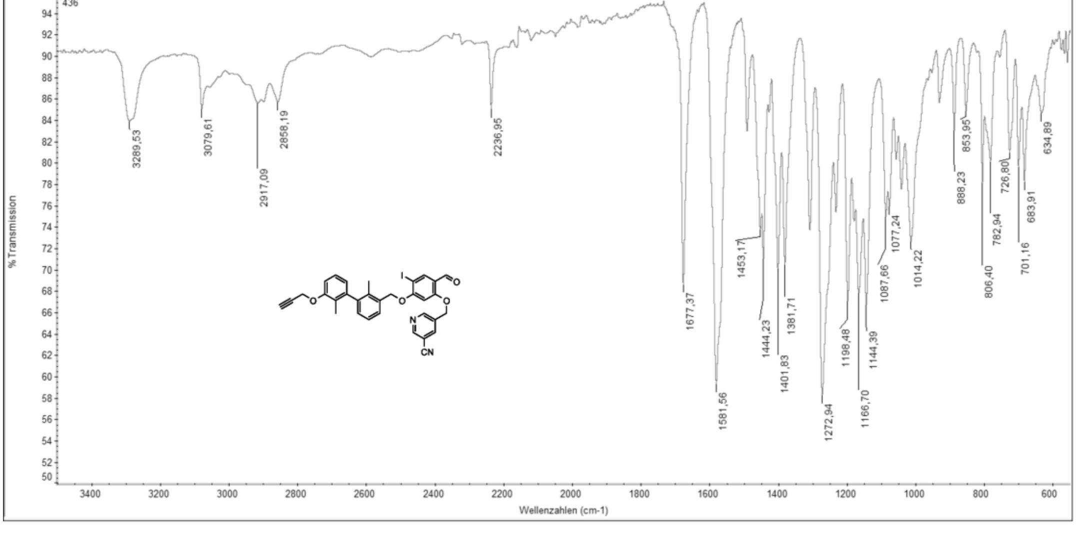

Figure S143: ATR-IR spectrum of compound 52c.

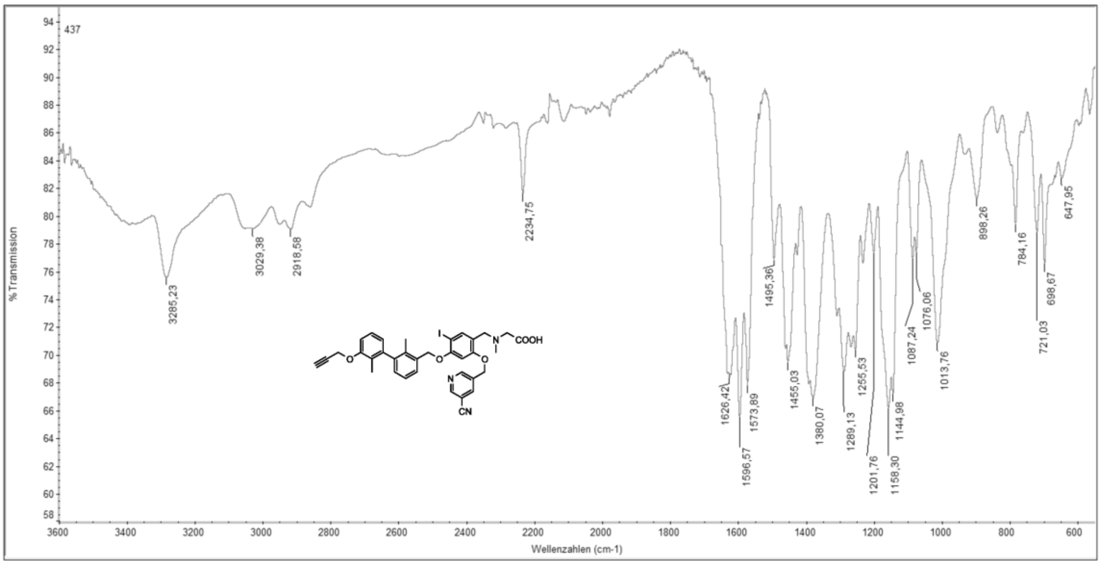

Figure S144: ATR-IR spectrum of compound 53c.

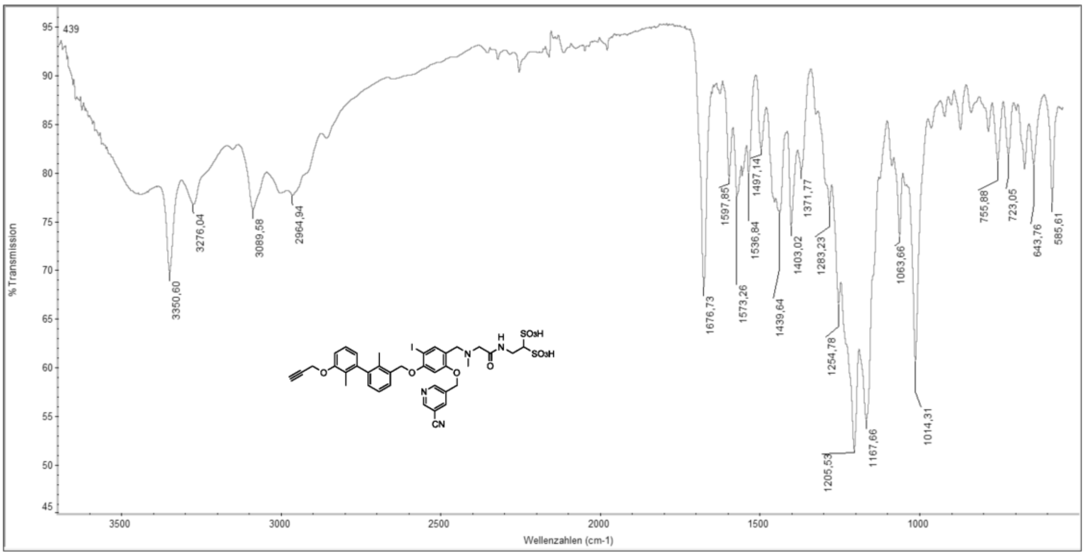

Figure S145: ATR-IR spectrum of compound 54c.

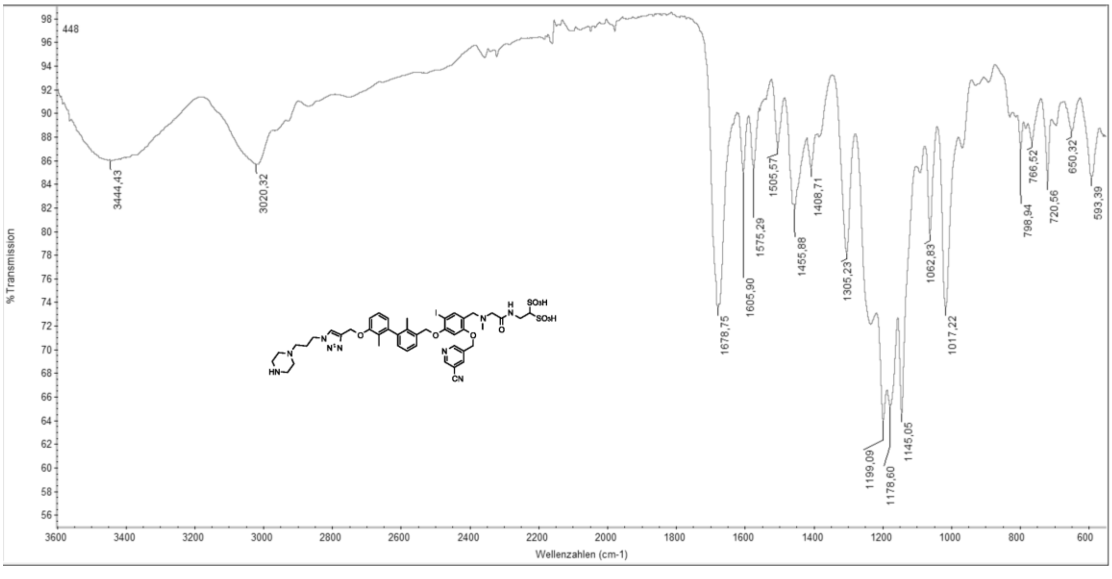

Figure S146: ATR-IR spectrum of compound 55c.

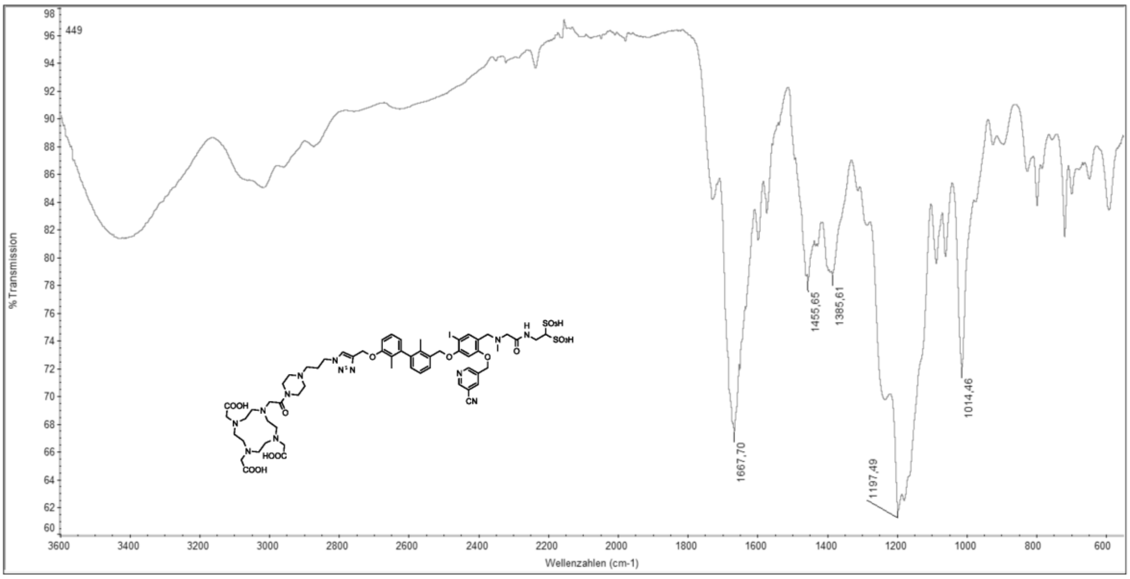

Figure S147: ATR-IR spectrum of compound 56c.

4. HR-MS spectra of literature unknown compounds

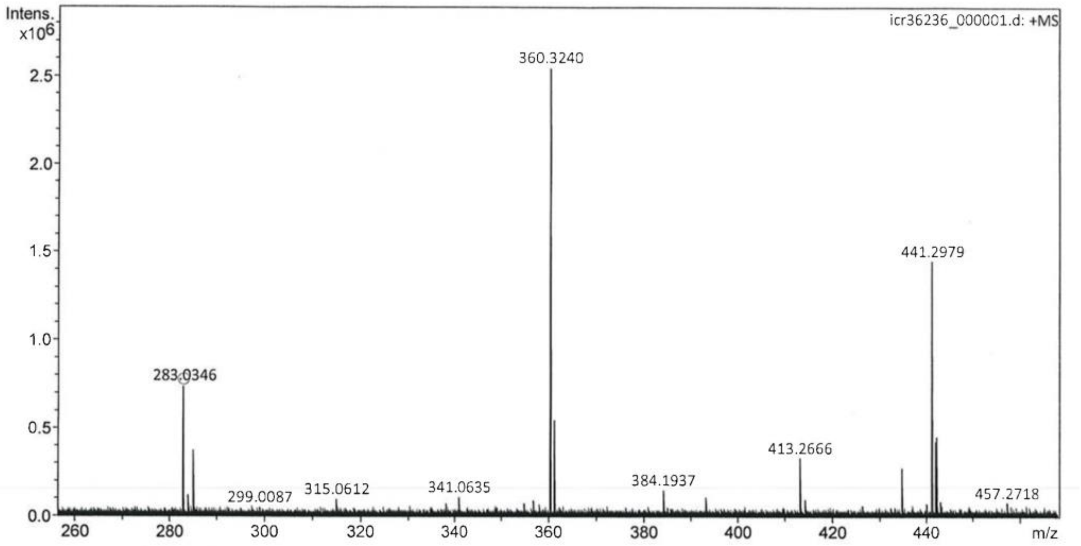

Figure S148: HR-MS Spectrum (ESI+) of compound 6.

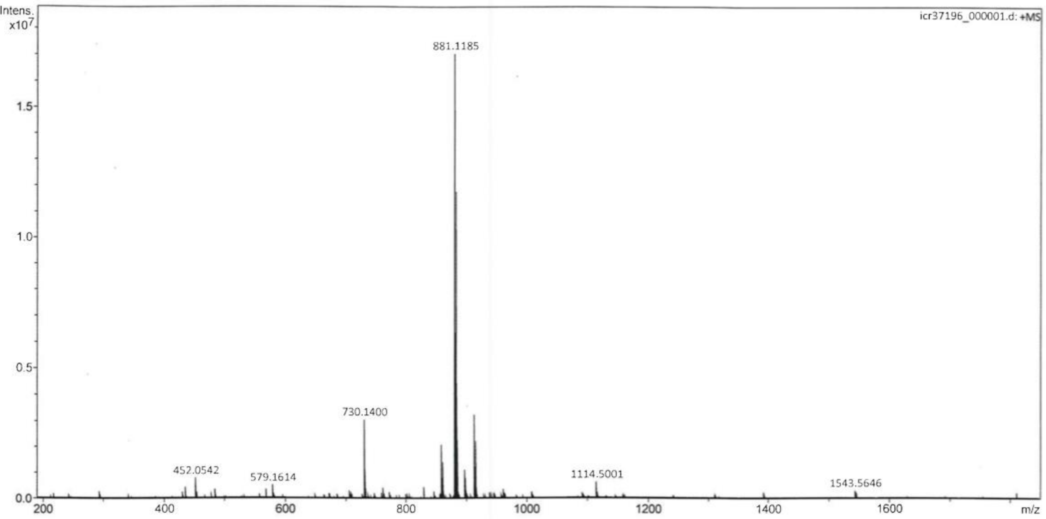

Figure S149: HR-MS Spectrum (ESI+) of compound 10.

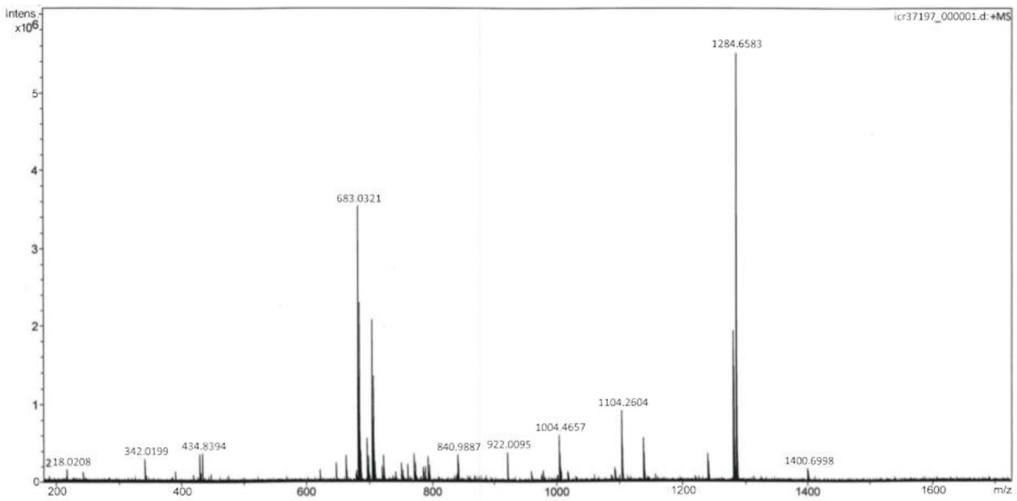

Figure S150: HR-MS Spectrum (ESI+) of compound 11.

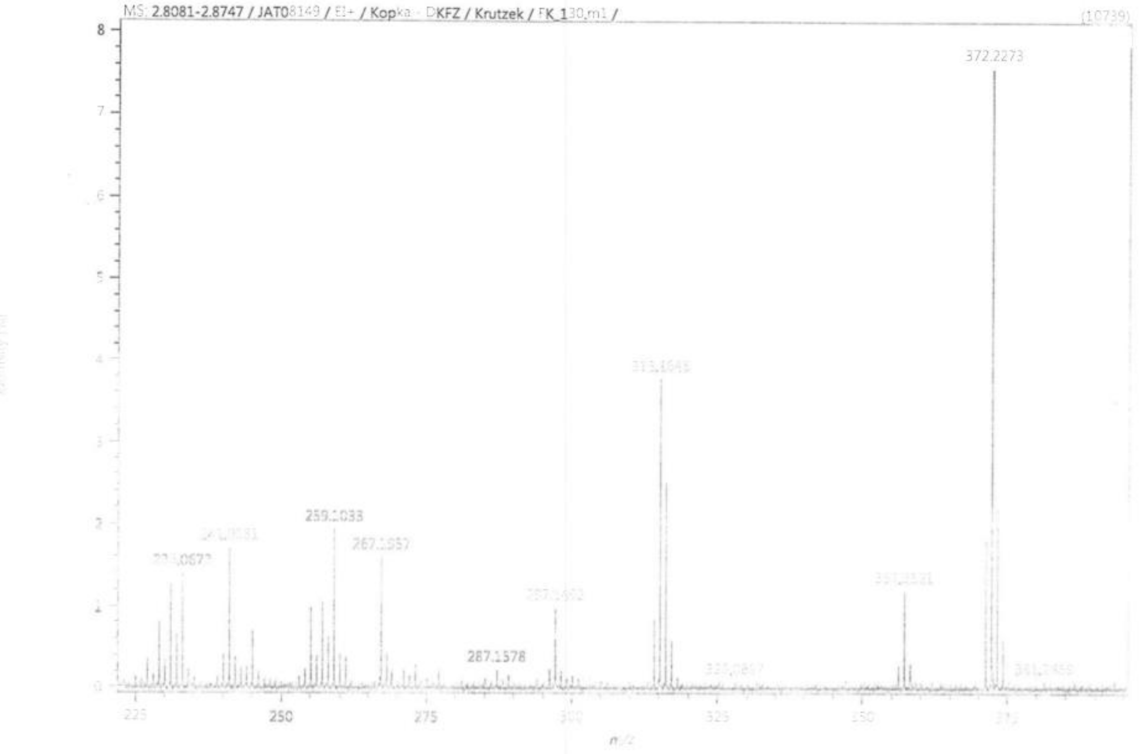

Figure S151: HR-MS Spectrum (ES) of compound 17.

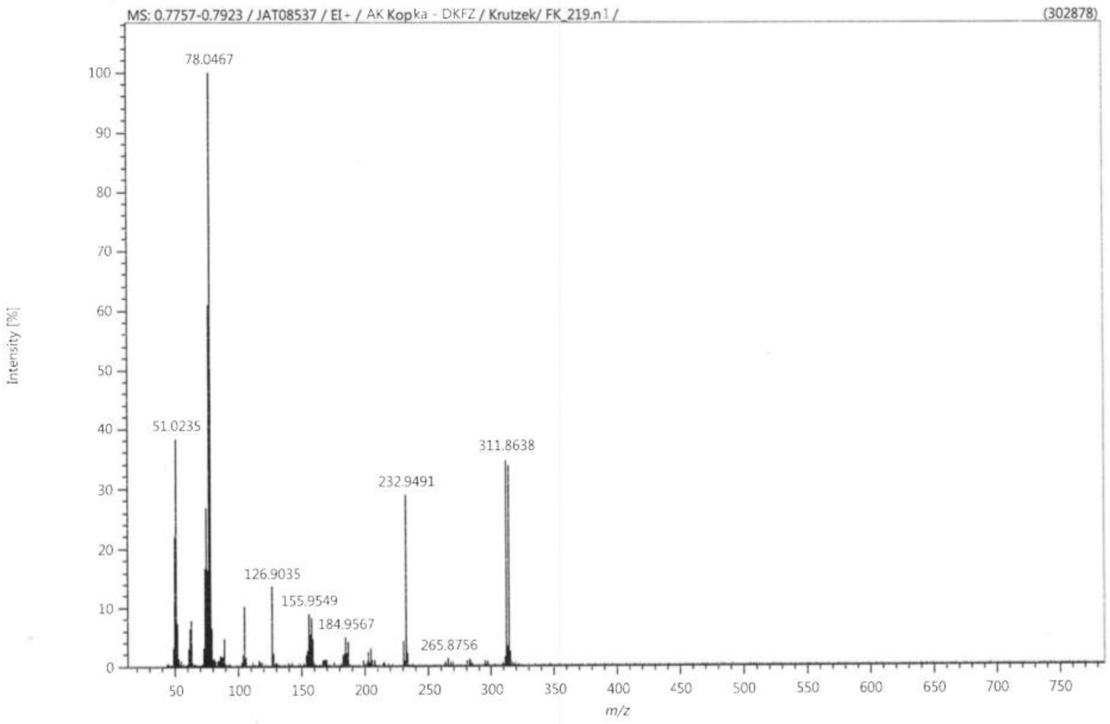

Figure S152: HR-MS Spectrum (ES) of compound 21.

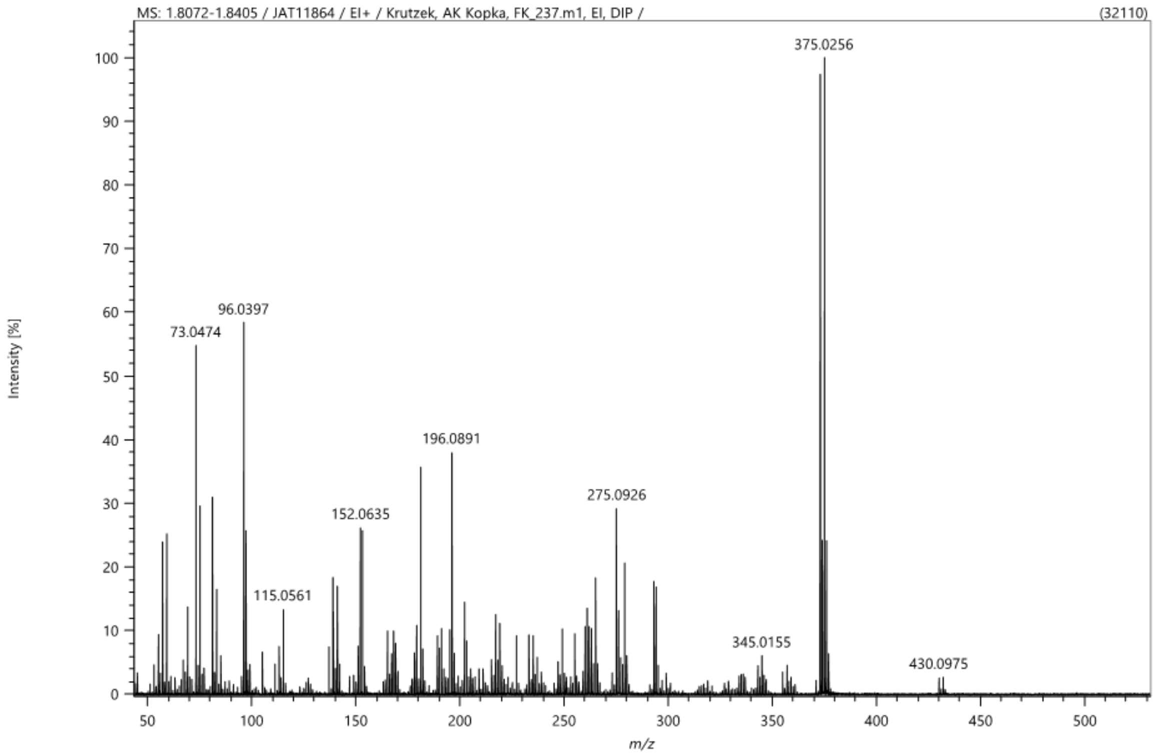

Figure S153: HR-MS Spectrum (ES) of compound 22.

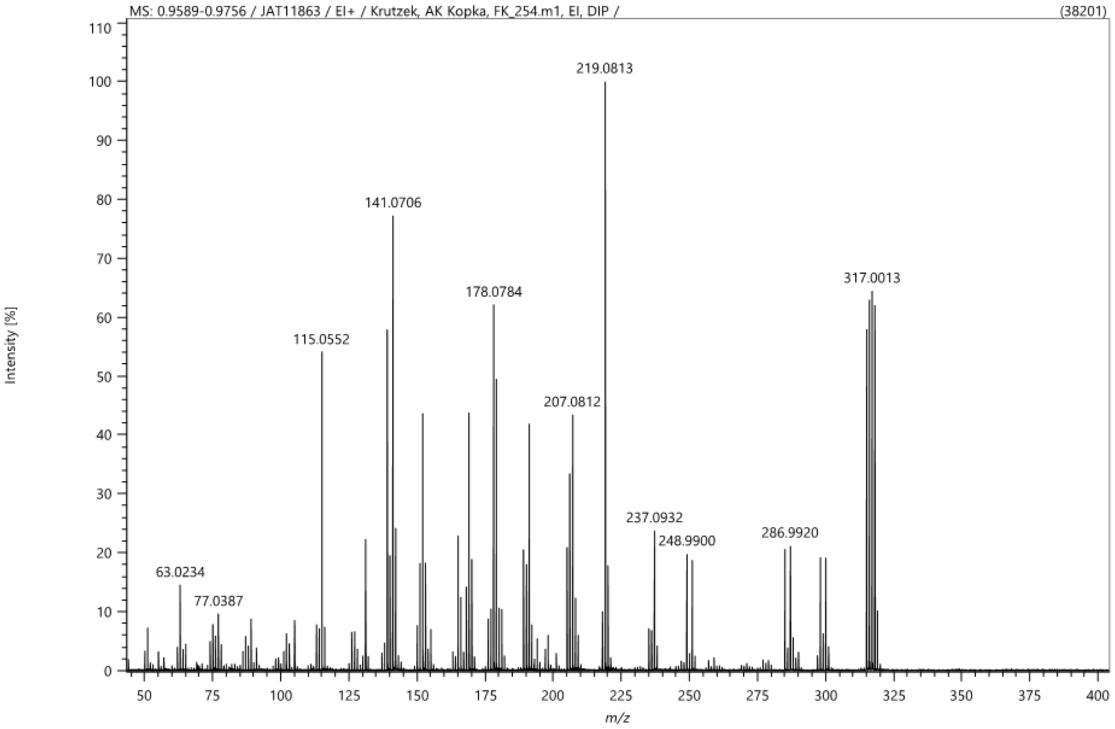

Figure S154: HR-MS Spectrum (ES) of compound 28a.

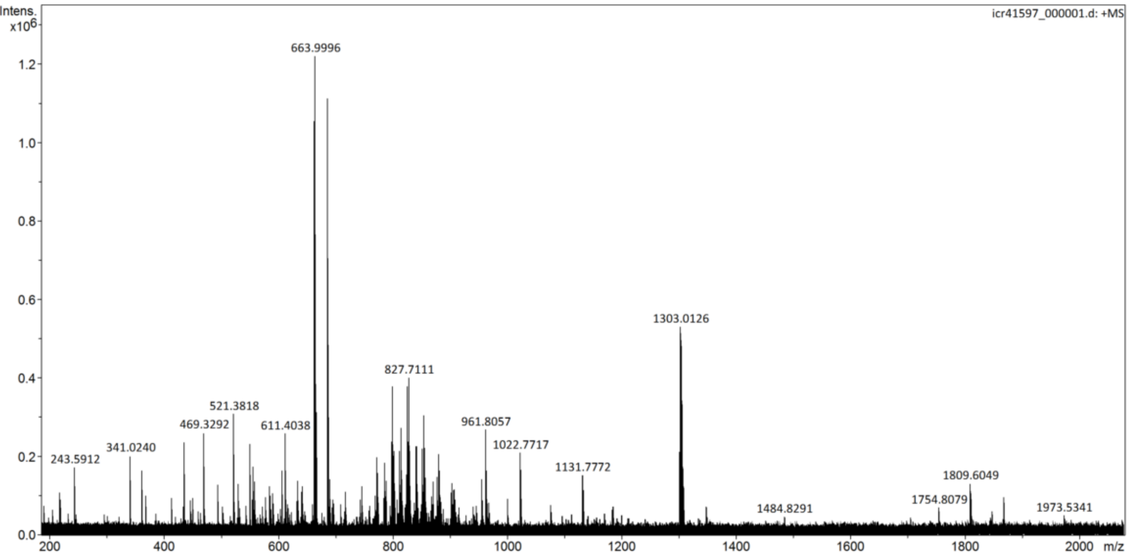

Figure S155: HR-MS Spectrum (EI) of compound 29a.

Fragmentor Voltage	Collision Energy	Ionization Mode
175	0	ESI

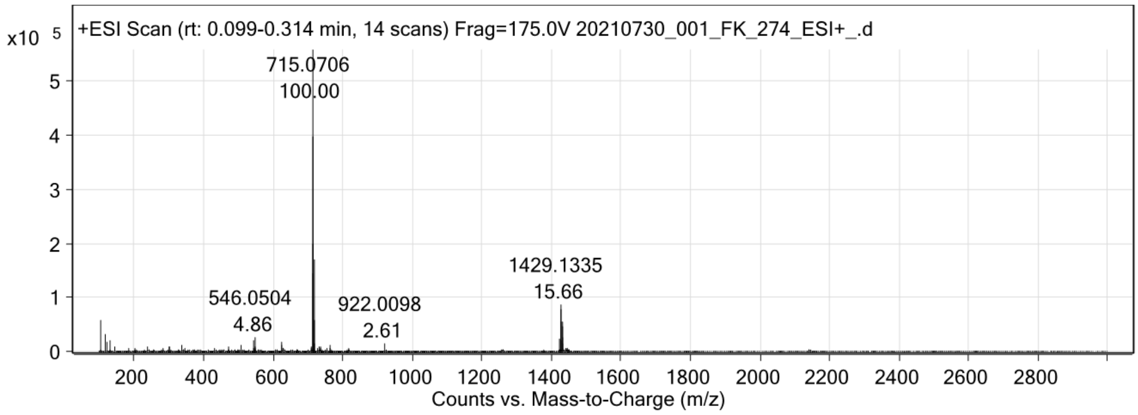

Figure S156: HR-MS Spectrum (ESI+) of compound 30a.

Fragmentor Voltage	Collision Energy	Ionization Mode
175	0	ESI

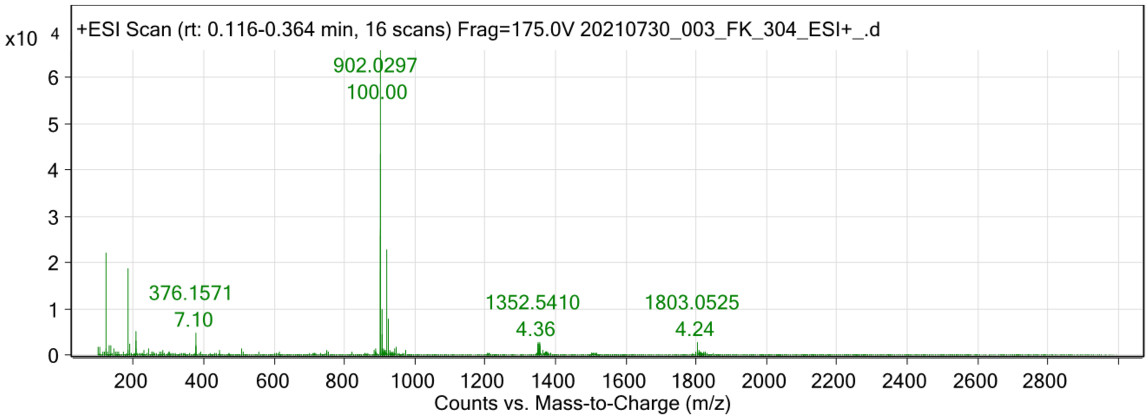

Figure S157: HR-MS Spectrum (ESI+) of compound 33a.

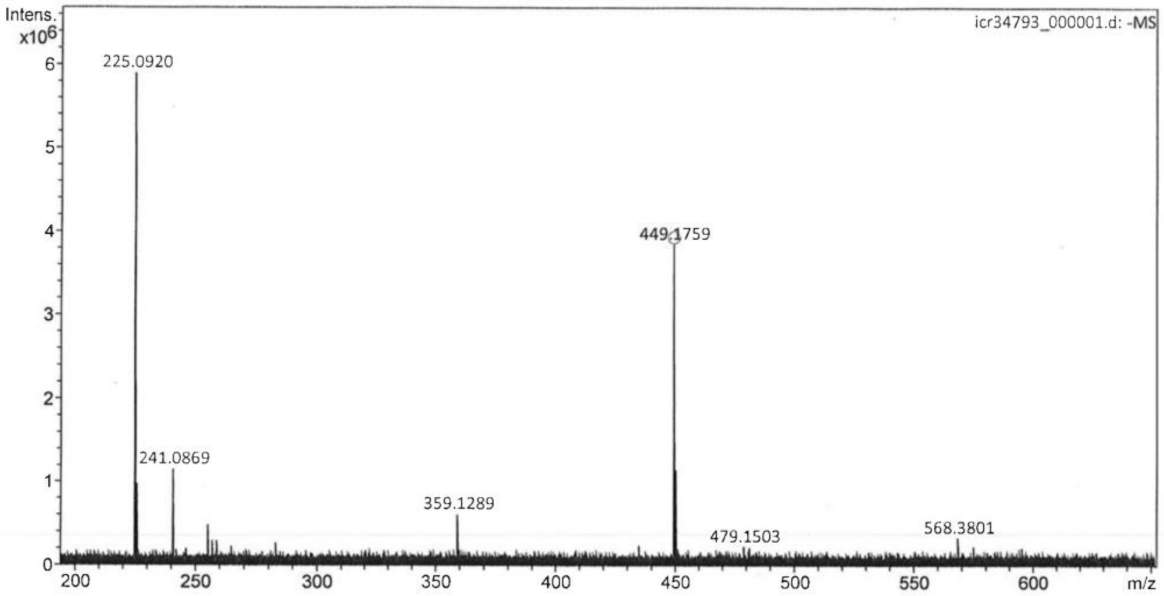

Figure S158: HR-MS Spectrum (ESI+) of compound 26.

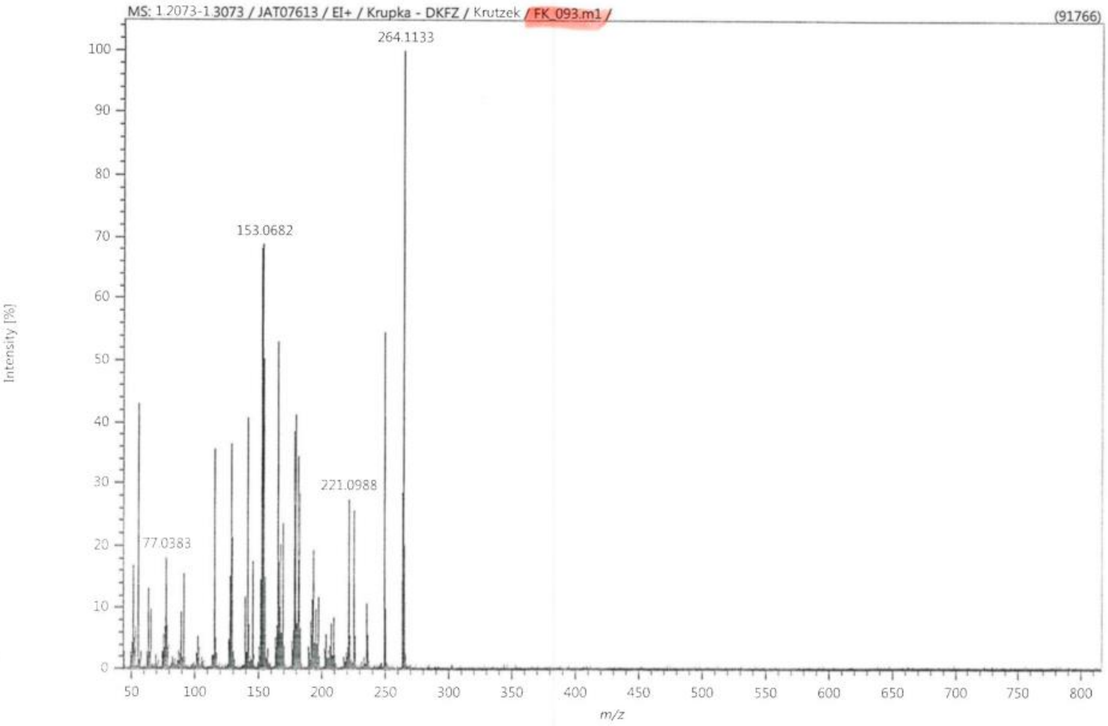

Figure S159: HR-MS Spectrum (EI) of compound 27.

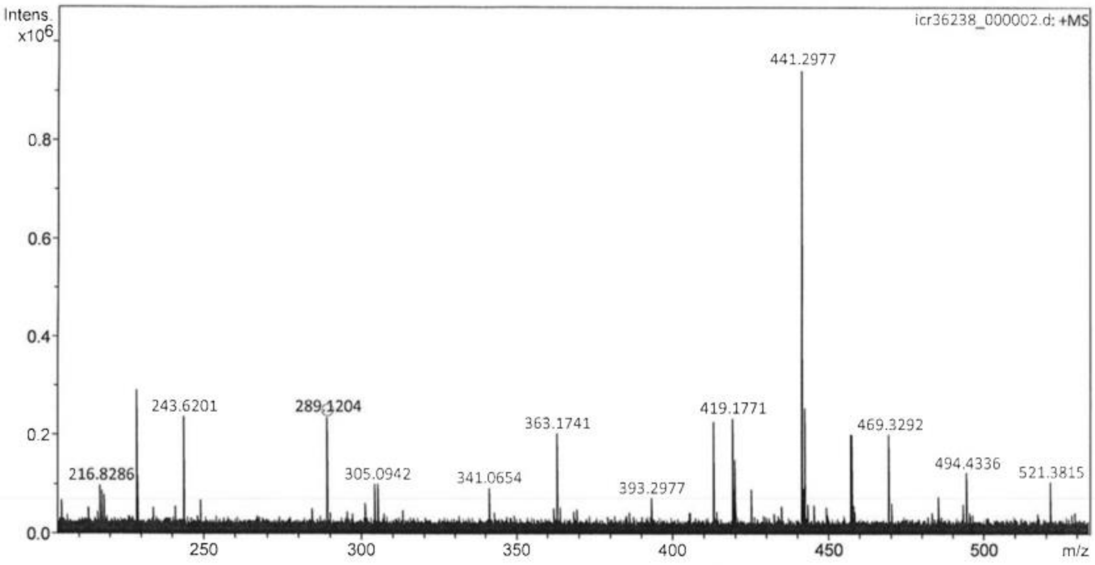

Figure S160: HR-MS Spectrum (EI) of compound 28b.

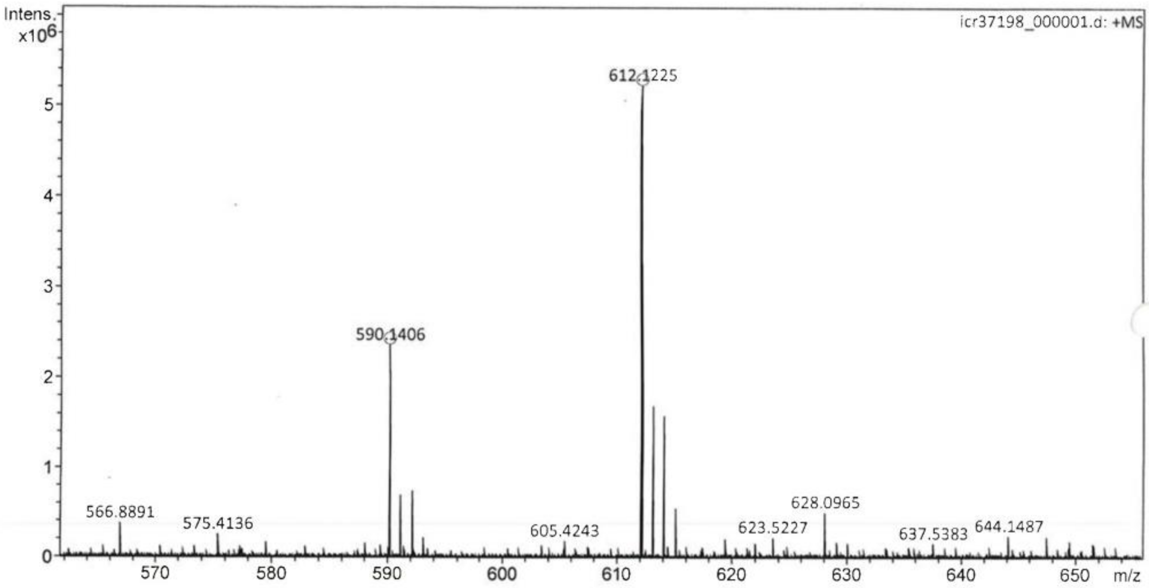

Figure S161: HR-MS Spectrum (EI) of compound 29b.

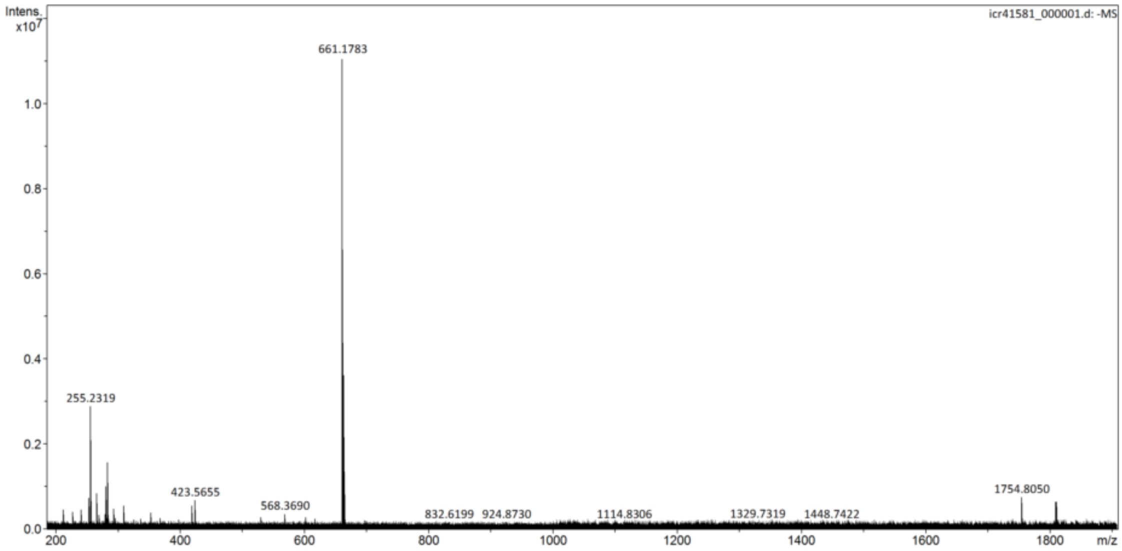

Figure S162: HR-MS Spectrum (ESI-) of compound 30b.

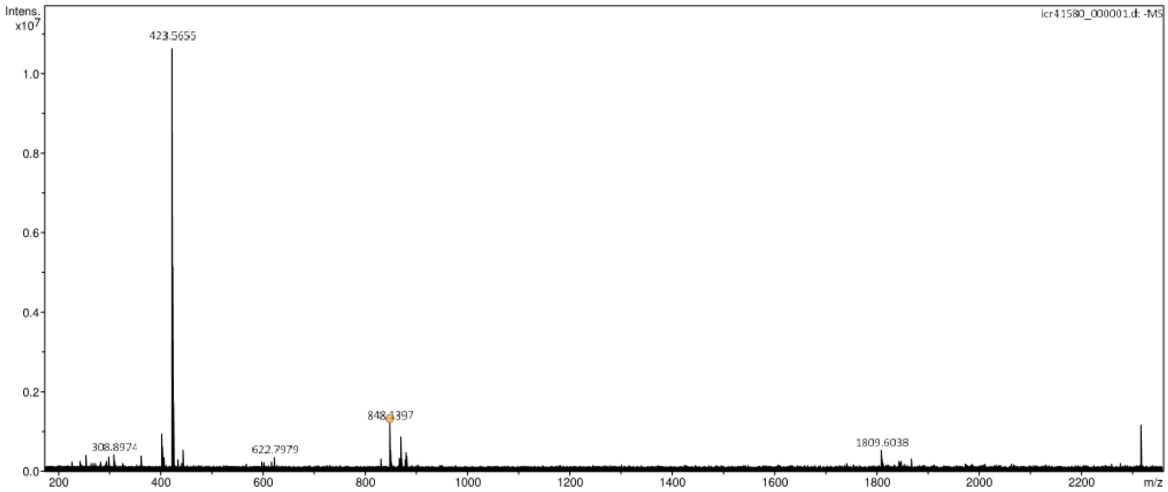

Figure S163: HR-MS Spectrum (ESI-) of compound 33b.

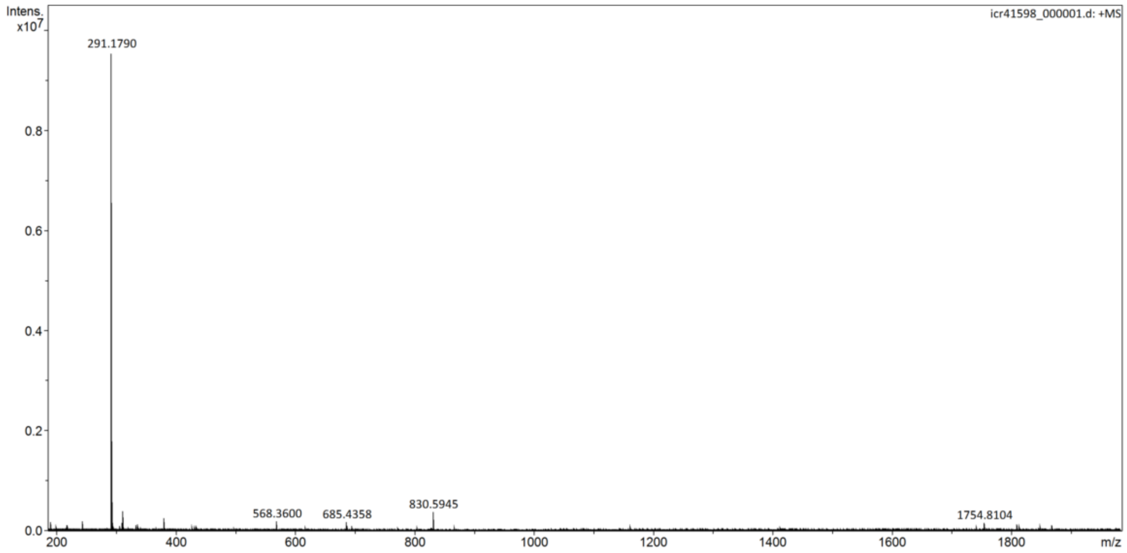

Figure S164: HR-MS Spectrum (ESI+) of compound S-5.

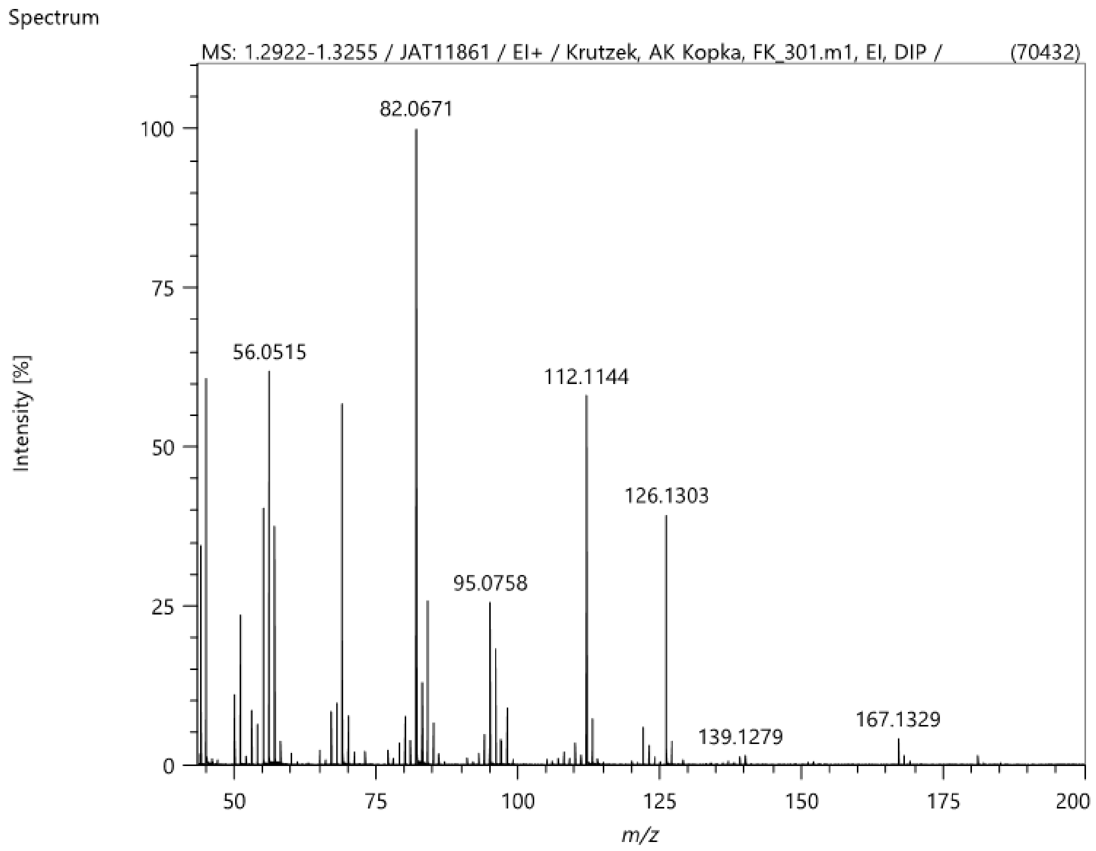

Figure S165: HR-MS Spectrum (EI) of compound 35.

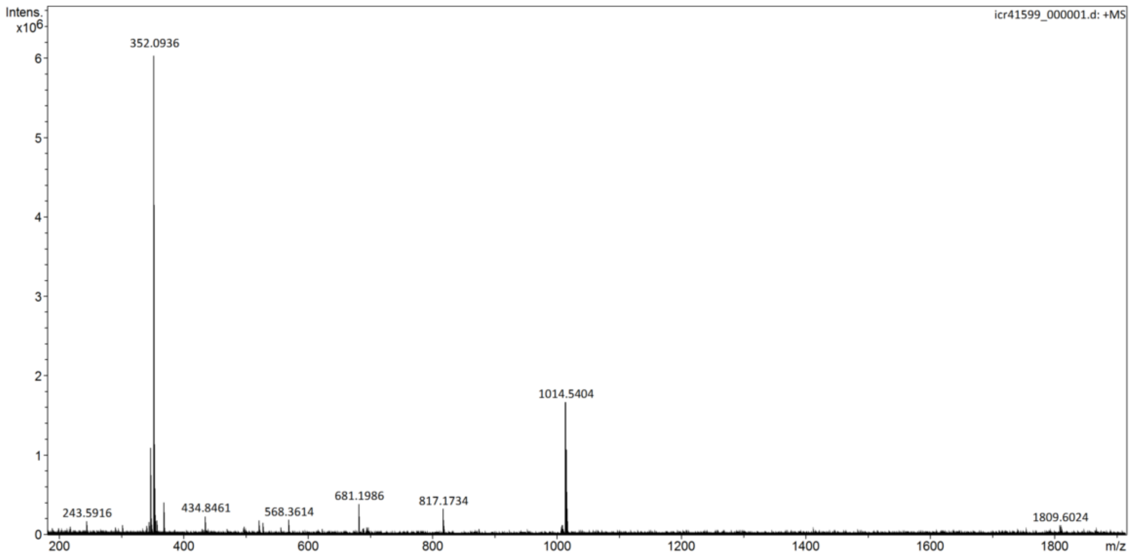

Figure S166: HR-MS Spectrum (ESI+) of compound S-6.

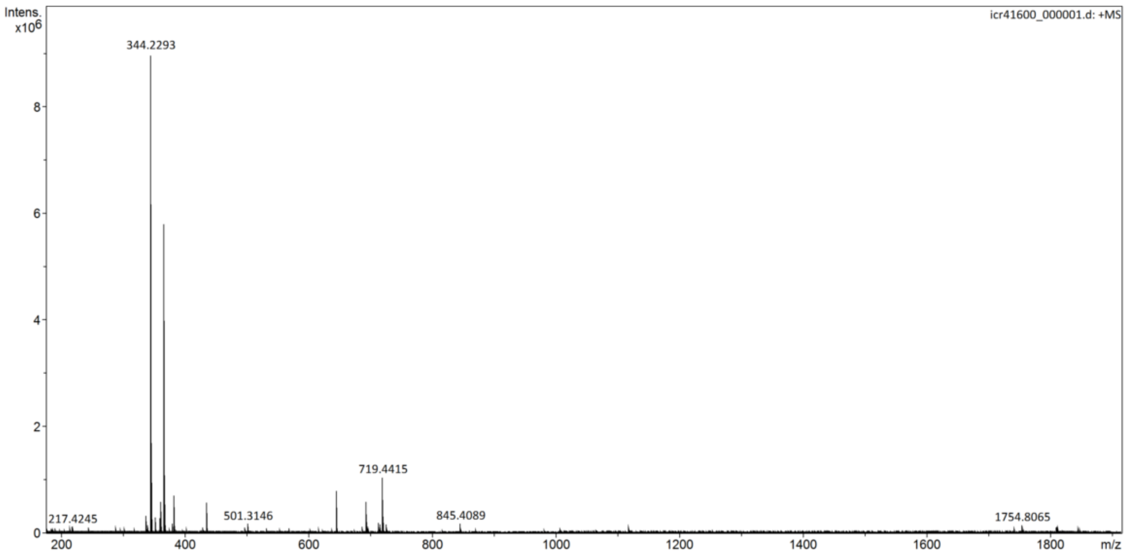

Figure S167: HR-MS Spectrum (ESI+) of compound S-7.

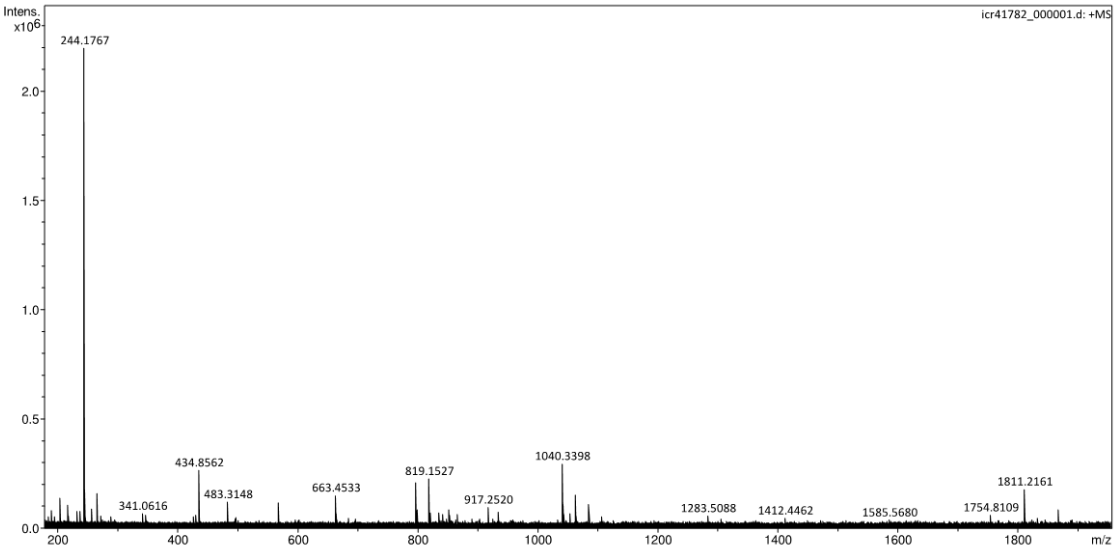

Figure S168: HR-MS Spectrum (ESI+) of compound 39.

Fragmentor Voltage	Collision Energy	Ionization Mode
175	0	ESI

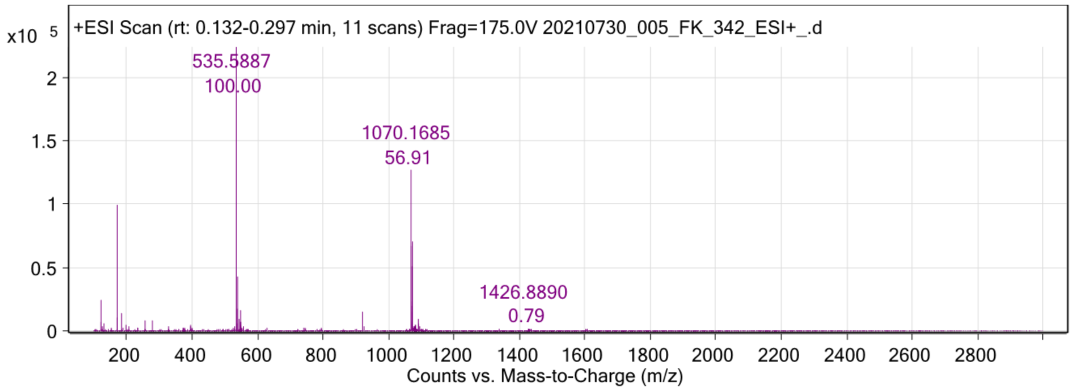

Figure S169: HR-MS Spectrum (ESI+) of compound 40a.

Fragmentor Voltage	Collision Energy	Ionization Mode
175	0	ESI

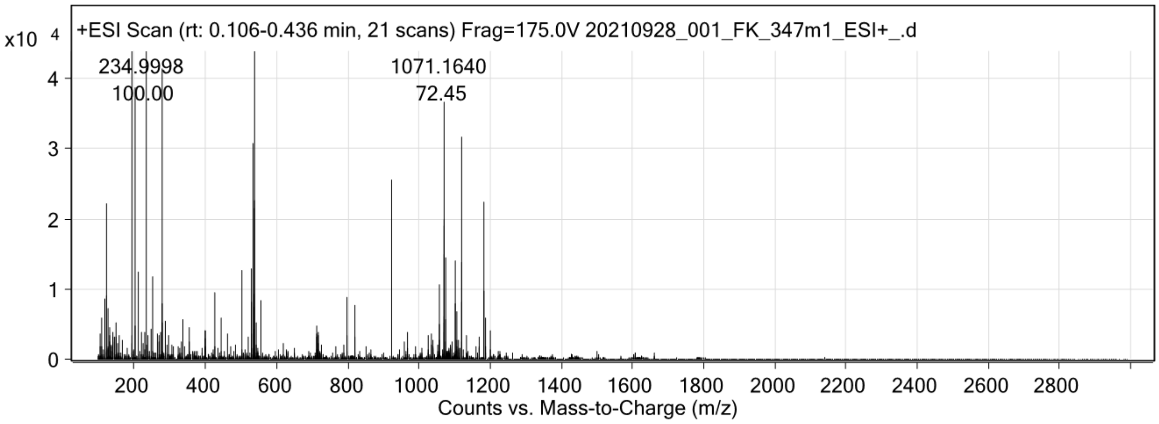

Figure S170: HR-MS Spectrum (ESI+) of compound 40b.

Fragmentor Voltage	Collision Energy	Ionization Mode
175	0	ESI

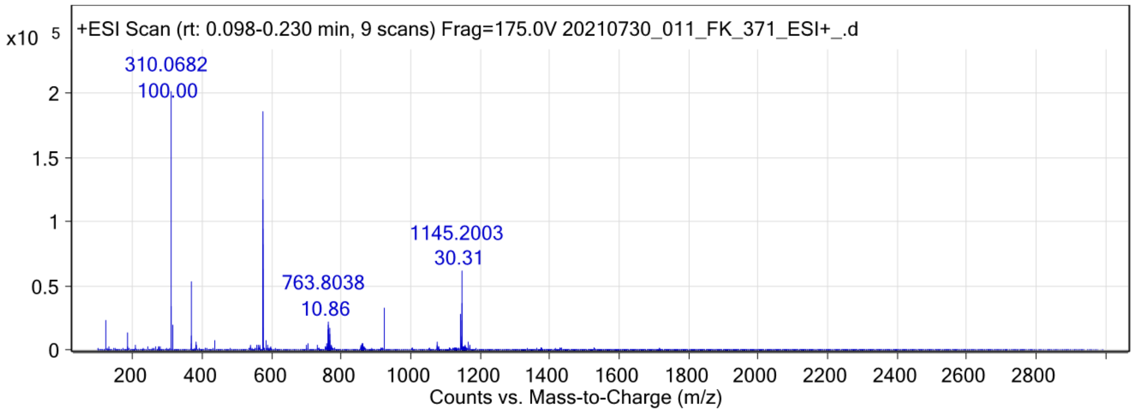

Figure S171: HR-MS Spectrum (ESI+) of compound 40c.

Fragmentor Voltage	Collision Energy	Ionization Mode
175	0	ESI

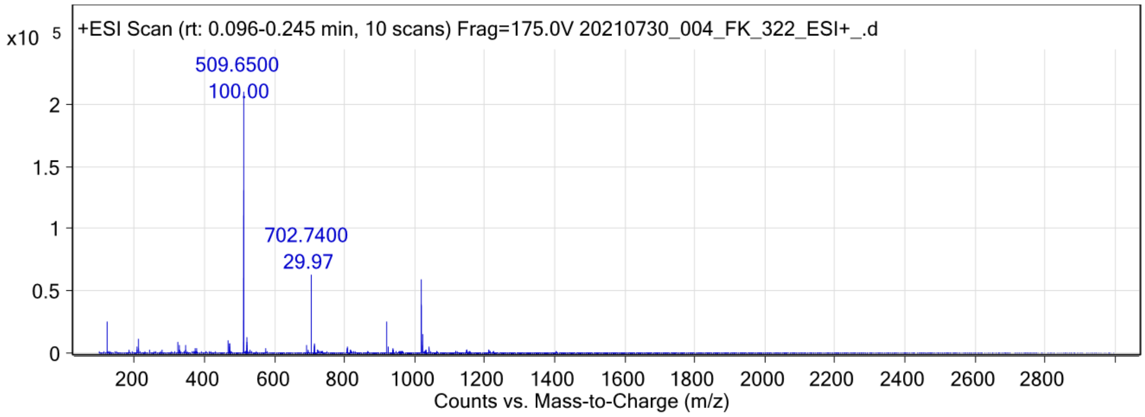

Figure S172: HR-MS Spectrum (ESI+) of compound 41a.

Fragmentor Voltage	Collision Energy	Ionization Mode
175	0	ESI

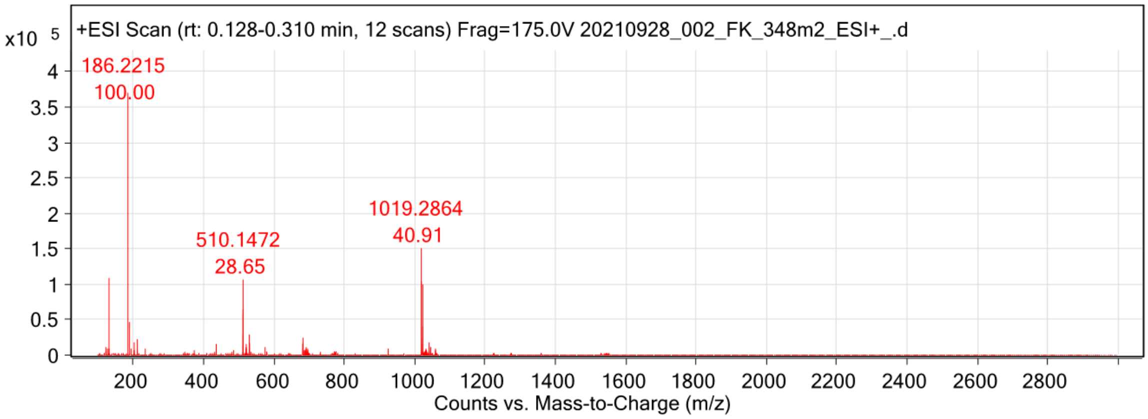

Figure S173: HR-MS Spectrum (ESI+) of compound 41b.

Fragmentor Voltage	Collision Energy	Ionization Mode
175	0	ESI

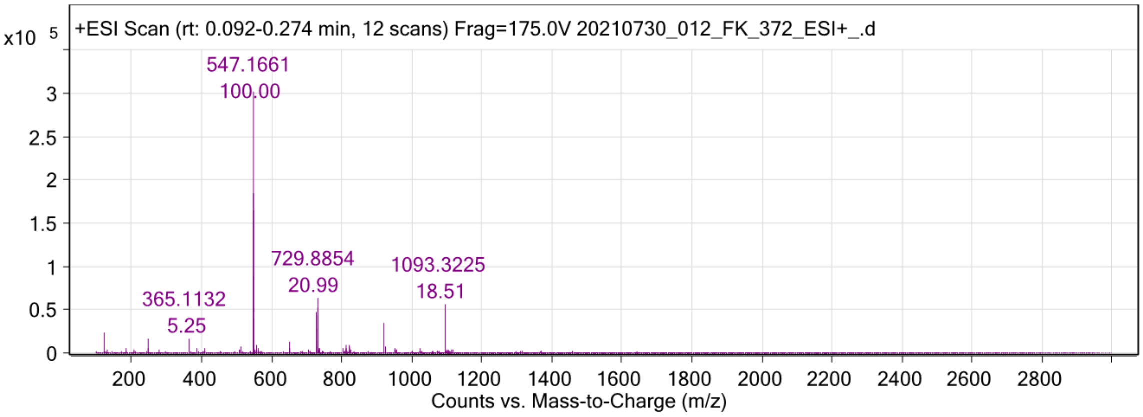

Figure S174: HR-MS Spectrum (ESI+) of compound 41c.

Fragmentor Voltage	Collision Energy	Ionization Mode
175	0	ESI

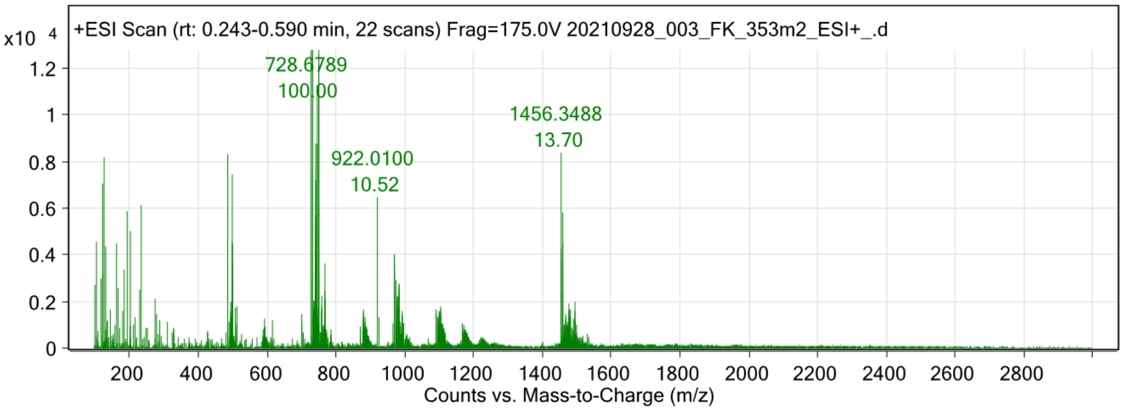

Figure S175: HR-MS Spectrum (ESI+) of compound 42a.

Fragmentor Voltage	Collision Energy	Ionization Mode
175	0	ESI

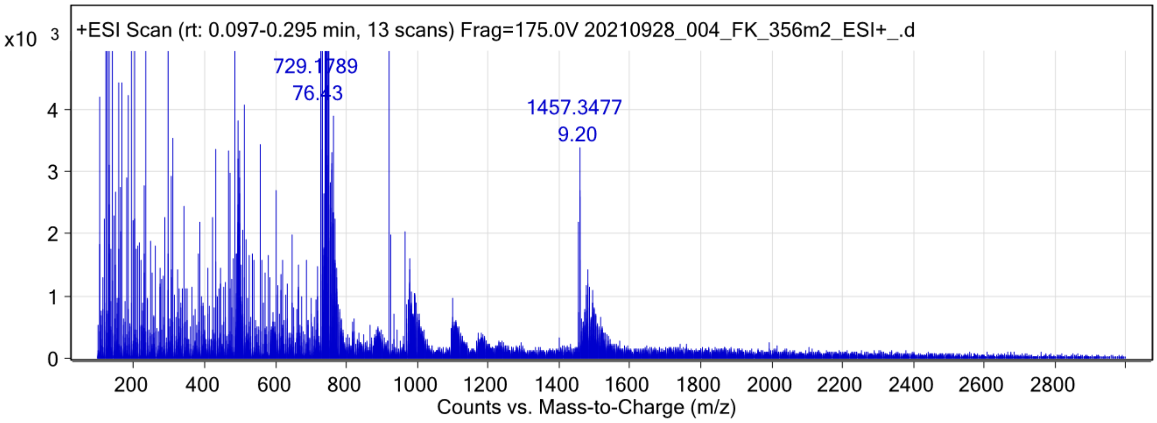

Figure S176: HR-MS Spectrum (ESI+) of compound 42b.

Fragmentor Voltage	Collision Energy	Ionization Mode
175	0	ESI

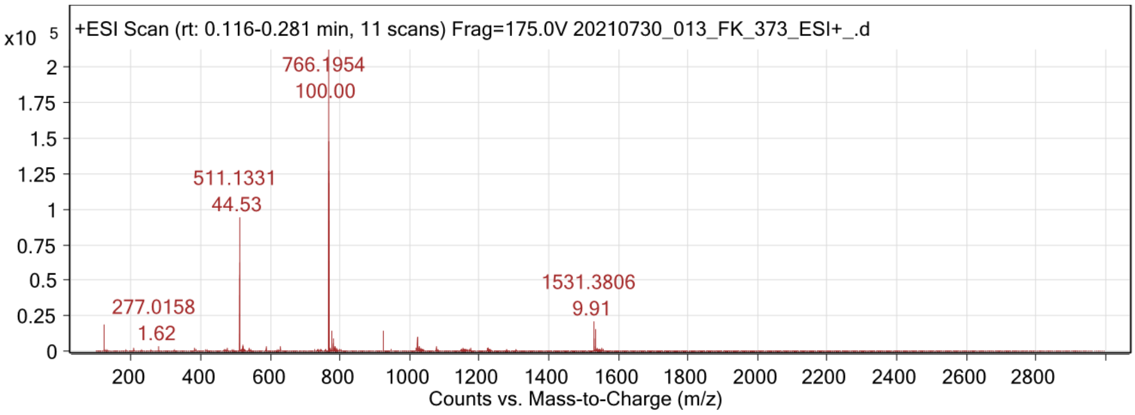

Figure S177: HR-MS Spectrum (ESI+) of compound 42c.

Fragmentor Voltage	Collision Energy	Ionization Mode
175	0	ESI

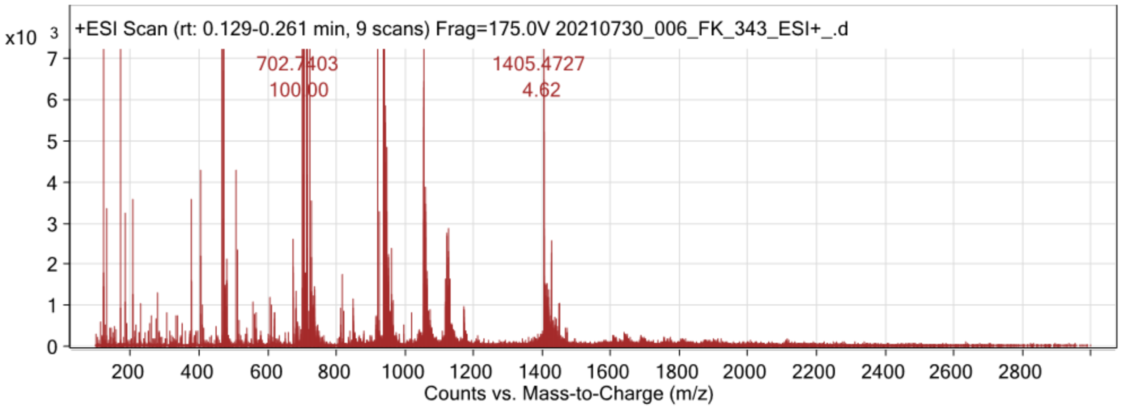

Figure S178: HR-MS Spectrum (ESI+) of compound 43a.

Fragmentor Voltage	Collision Energy	Ionization Mode
175	0	ESI

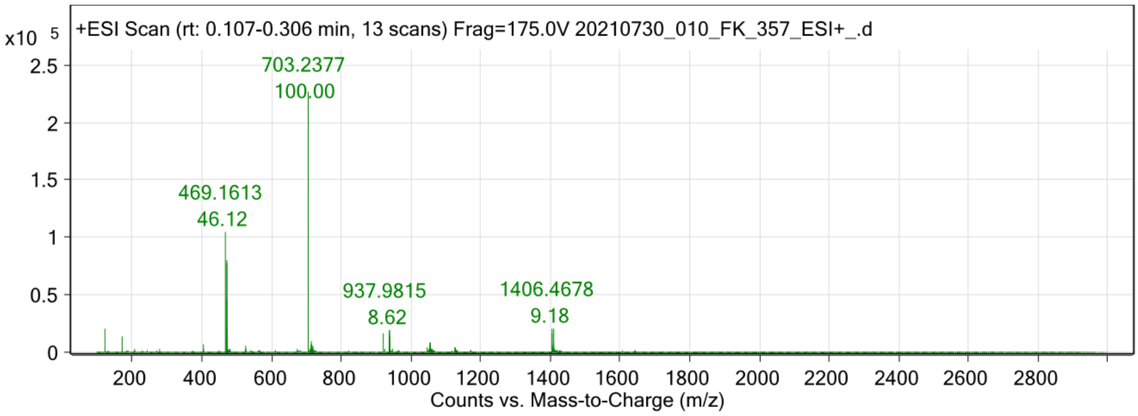

Figure S179: HR-MS Spectrum (ESI+) of compound 43b.

Fragmentor Voltage	Collision Energy	Ionization Mode
175	0	ESI

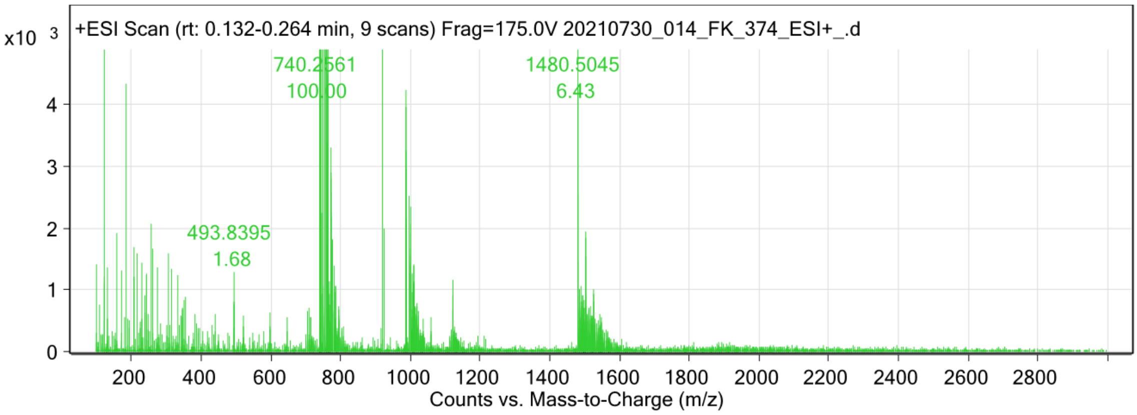

Figure S180: HR-MS Spectrum (ESI+) of compound 43c.

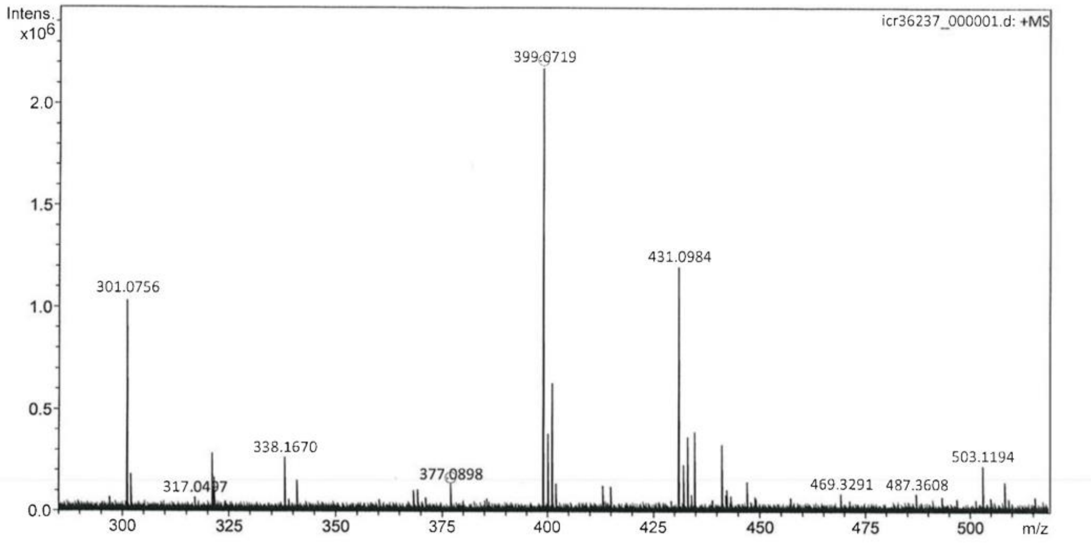

Figure S181: HR-MS Spectrum (ESI+) of compound 46a.

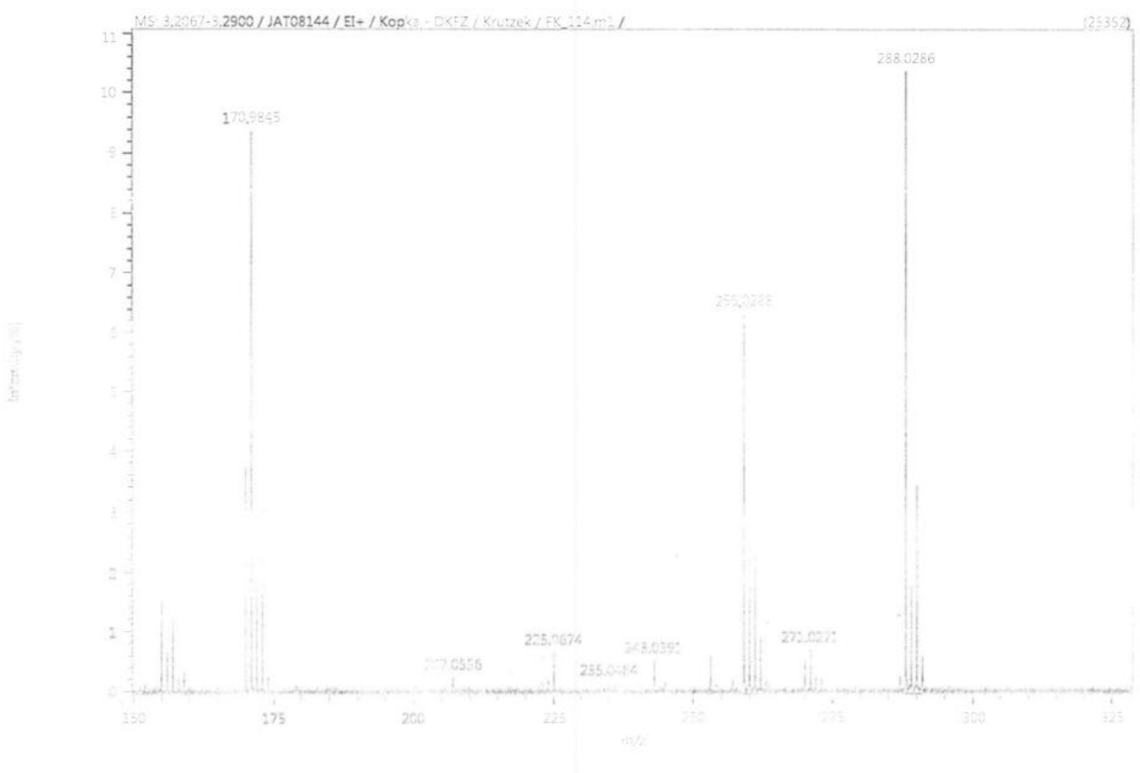

Figure S182: HR-MS Spectrum (EI) of compound 51a.

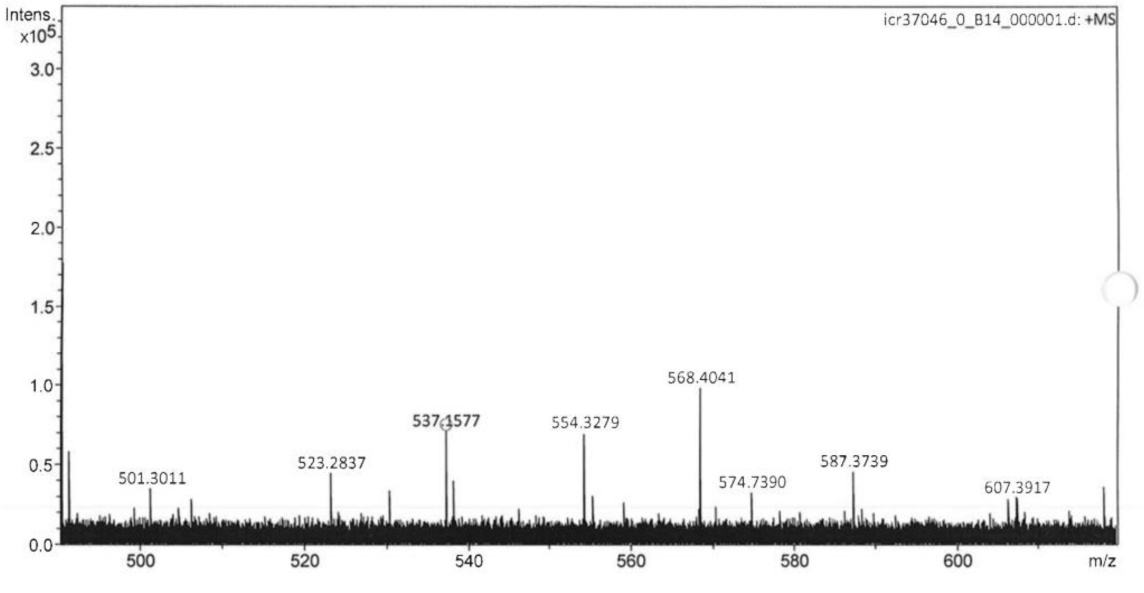

Figure S183: HR-MS Spectrum (ESI+) of compound 52a.

Fragmentor Voltage	Collision Energy	Ionization Mode
175	0	ESI

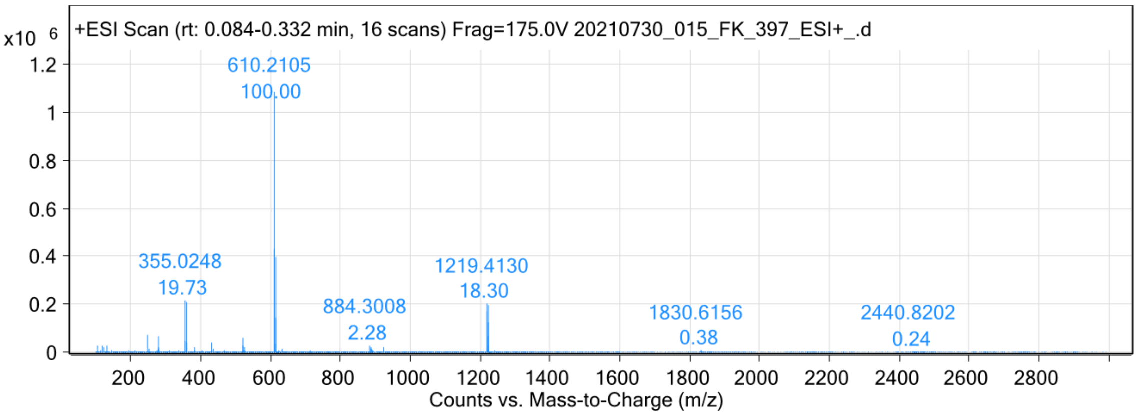

Figure S184: HR-MS Spectrum (ESI+) of compound 53a.

Fragmentor Voltage	Collision Energy	Ionization Mode
175	0	ESI

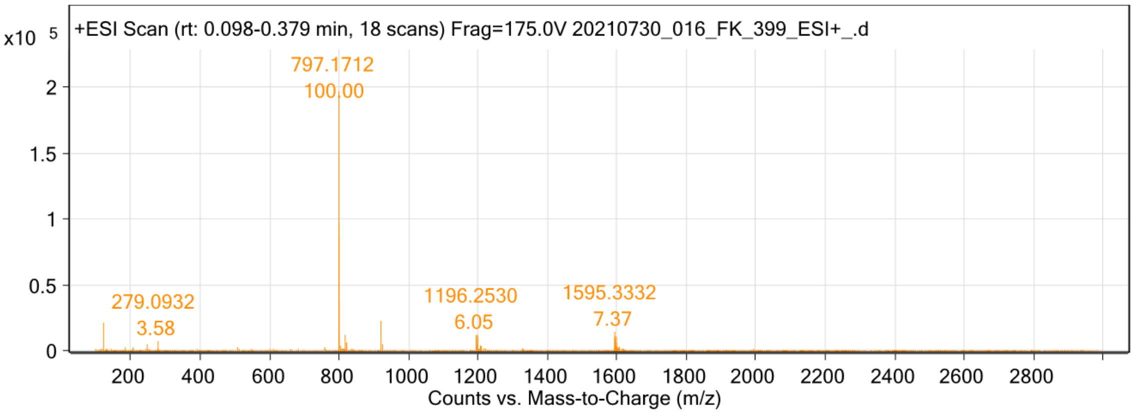

Figure S185: HR-MS Spectrum (ESI+) of compound 54a.

Fragmentor Voltage	Collision Energy	Ionization Mode
175	0	ESI

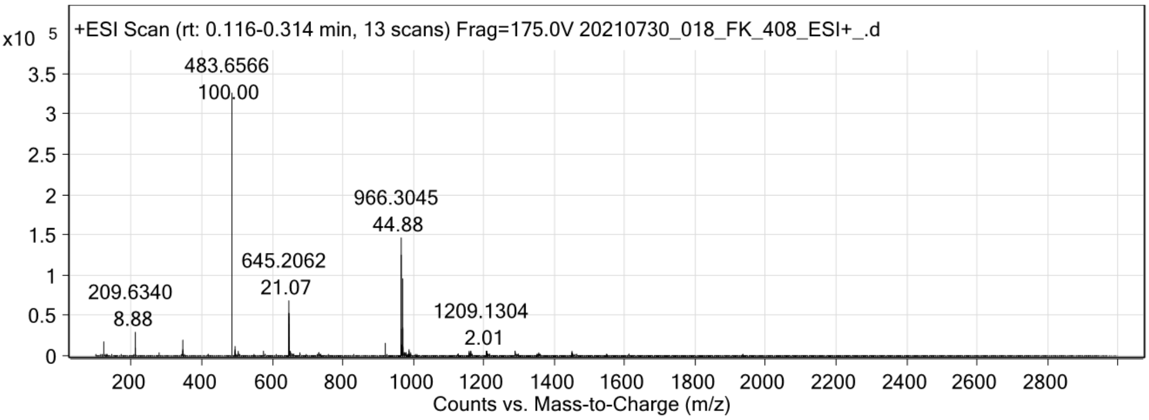

Figure S186: HR-MS Spectrum (ESI+) of compound 55a.

Fragmentor Voltage	Collision Energy	Ionization Mode
175	0	ESI

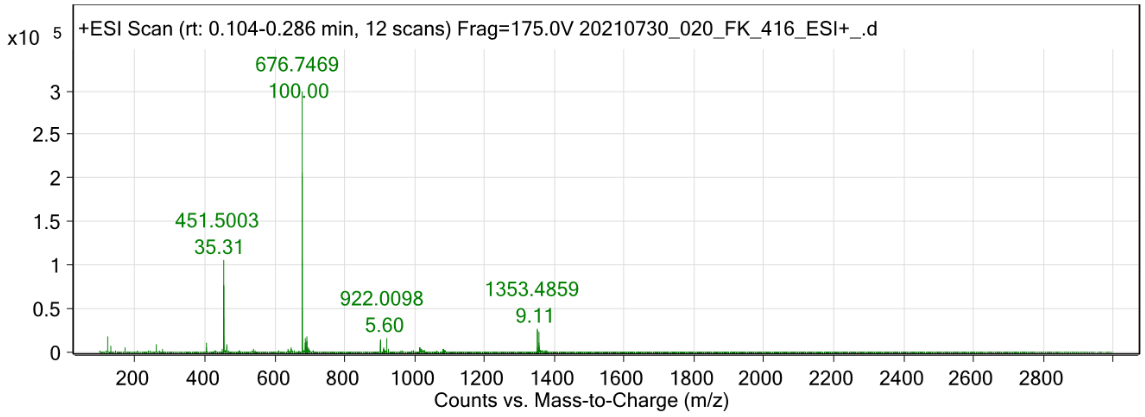

Figure S187: HR-MS Spectrum (ESI+) of compound 56a.

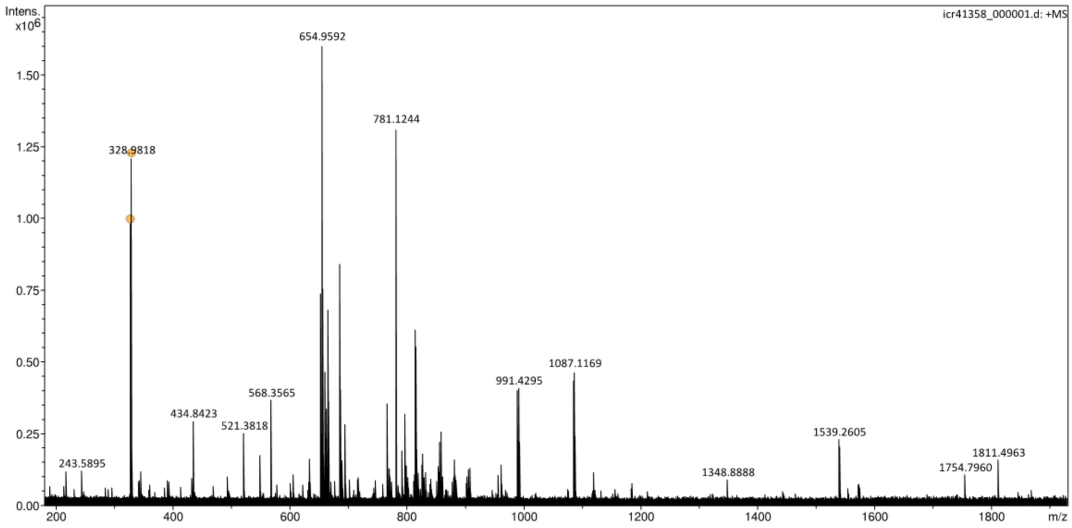

Figure S188: HR-MS Spectrum (ESI+) of compound 45.

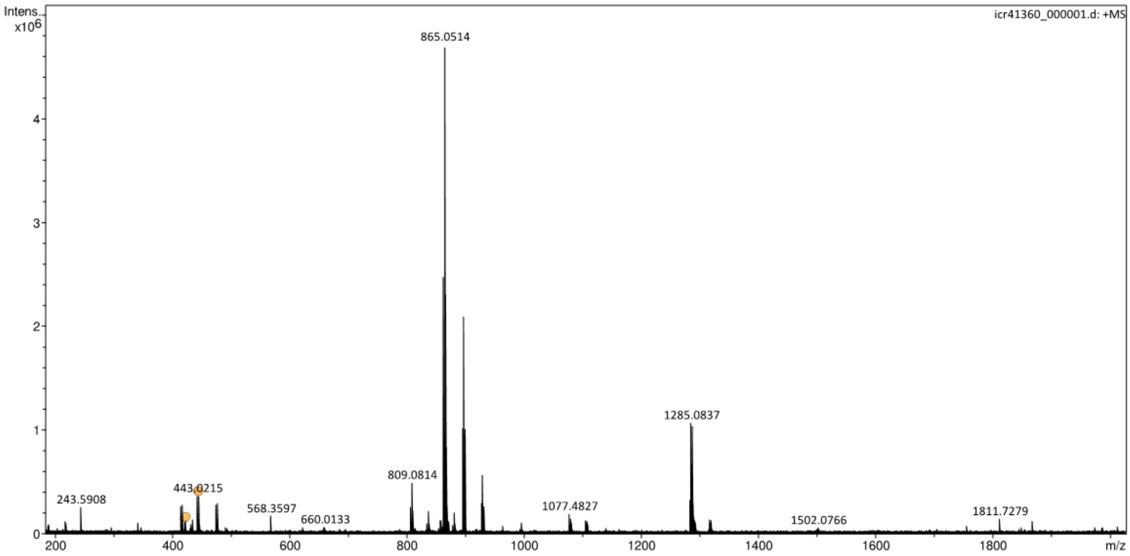

Figure S189: HR-MS Spectrum (ESI+) of compound 46b.

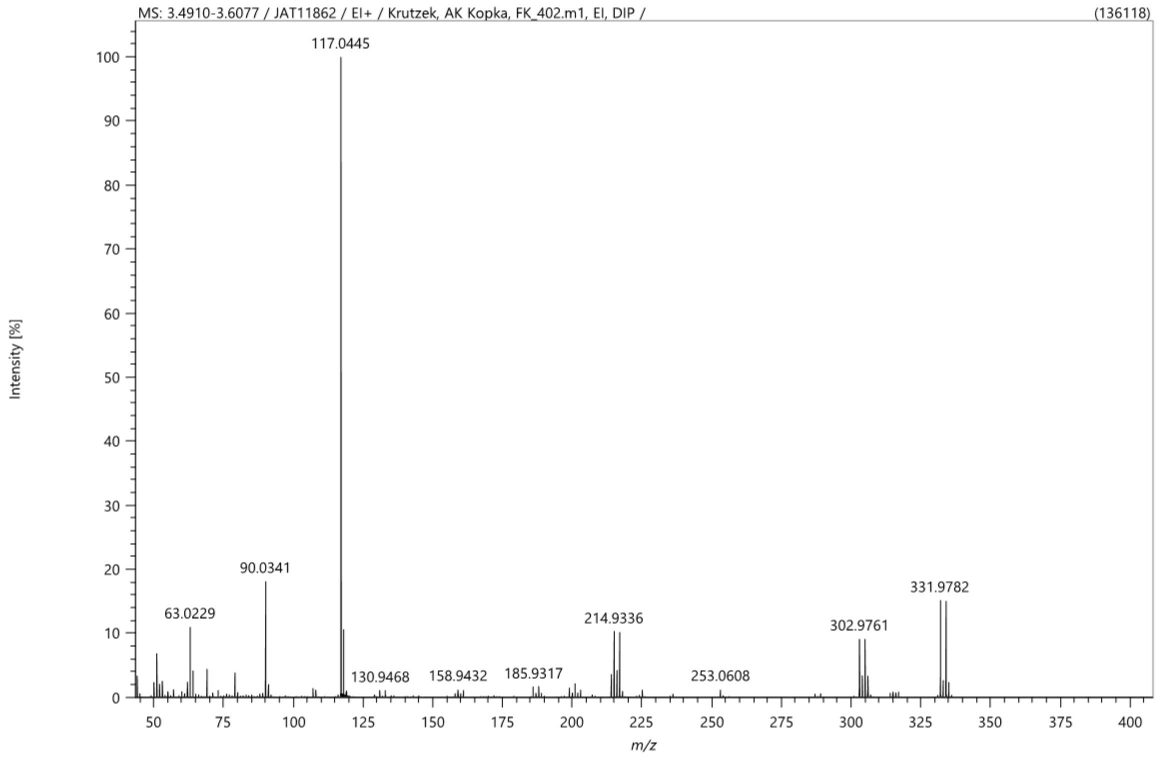

Figure S190: HR-MS Spectrum (ESI+) of compound 51b.

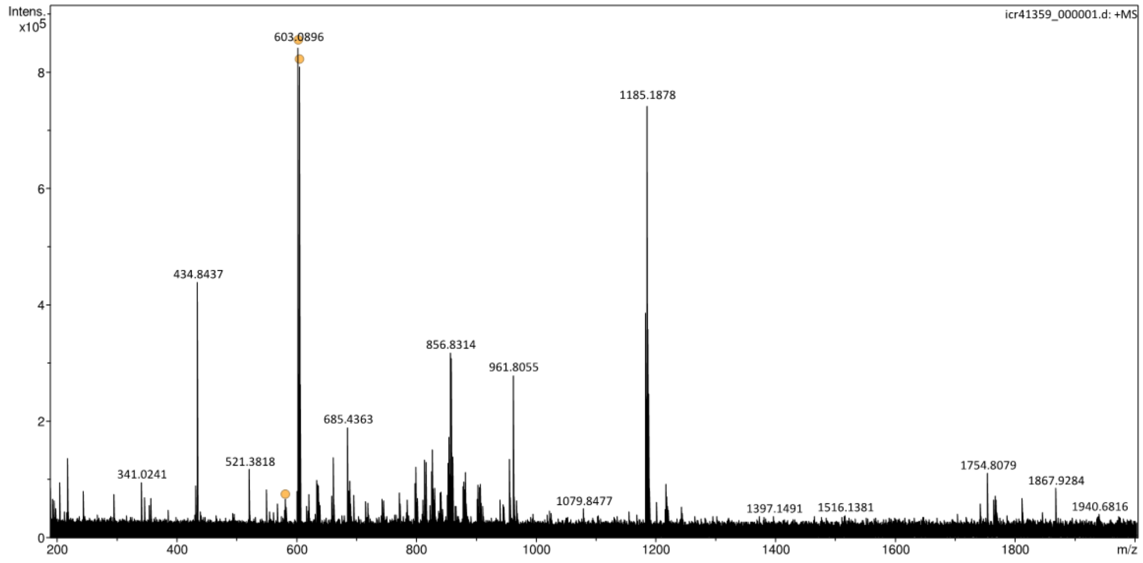

Figure S191: HR-MS Spectrum (ESI+) of compound 52b.

Fragmentor Voltage	Collision Energy	Ionization Mode
175	0	ESI

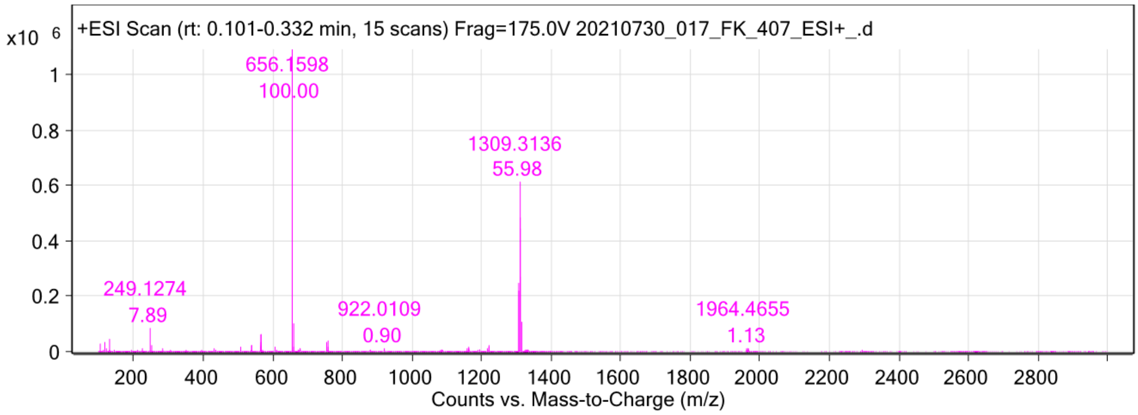

Figure S192: HR-MS Spectrum (ESI+) of compound 53b.

Fragmentor Voltage	Collision Energy	Ionization Mode
175	0	ESI

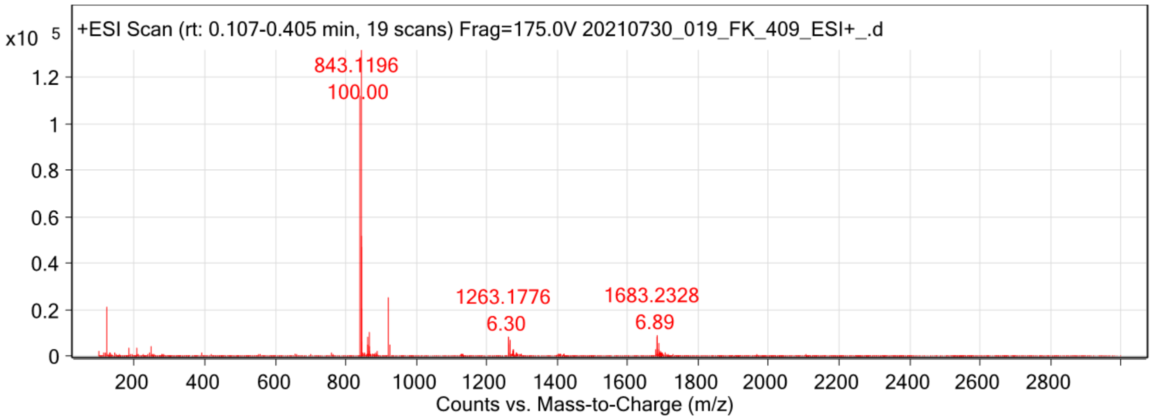

Figure S193: HR-MS Spectrum (ESI+) of compound 54b.

Fragmentor Voltage	Collision Energy	Ionization Mode
175	0	ESI

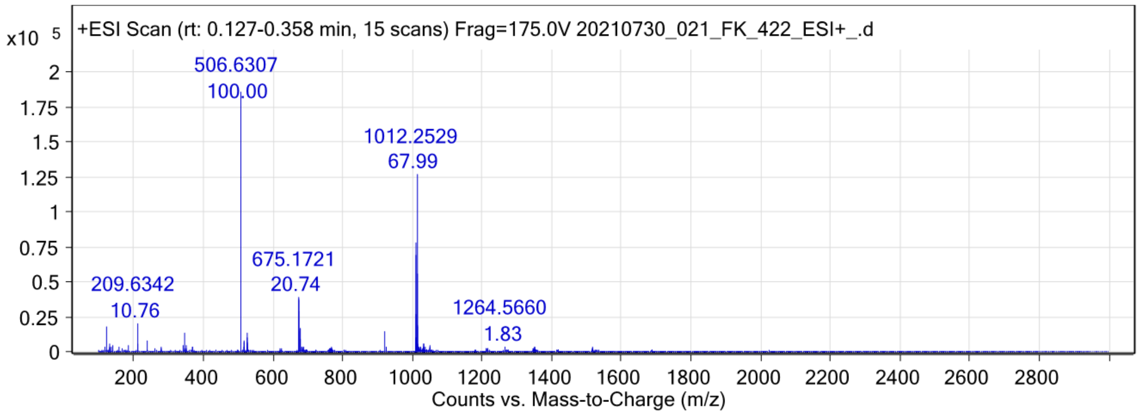

Figure S194: HR-MS Spectrum (ESI+) of compound 55b.

Fragmentor Voltage	Collision Energy	Ionization Mode
175	0	ESI

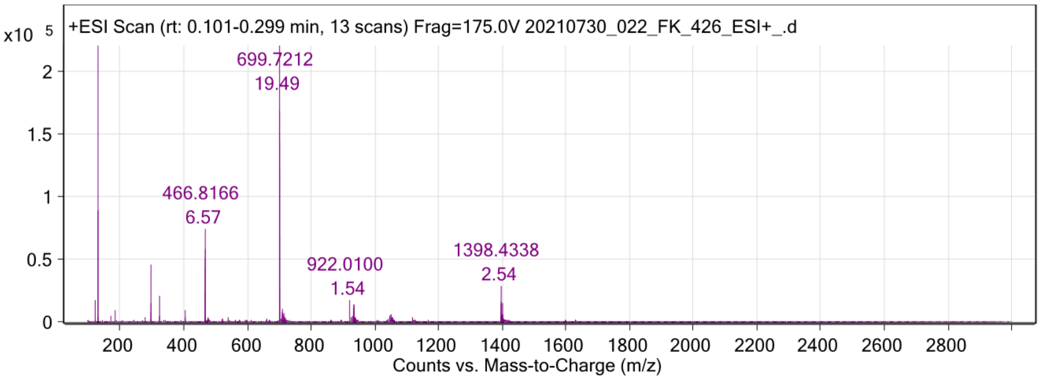

Figure S195: HR-MS Spectrum (ESI+) of compound 56b.

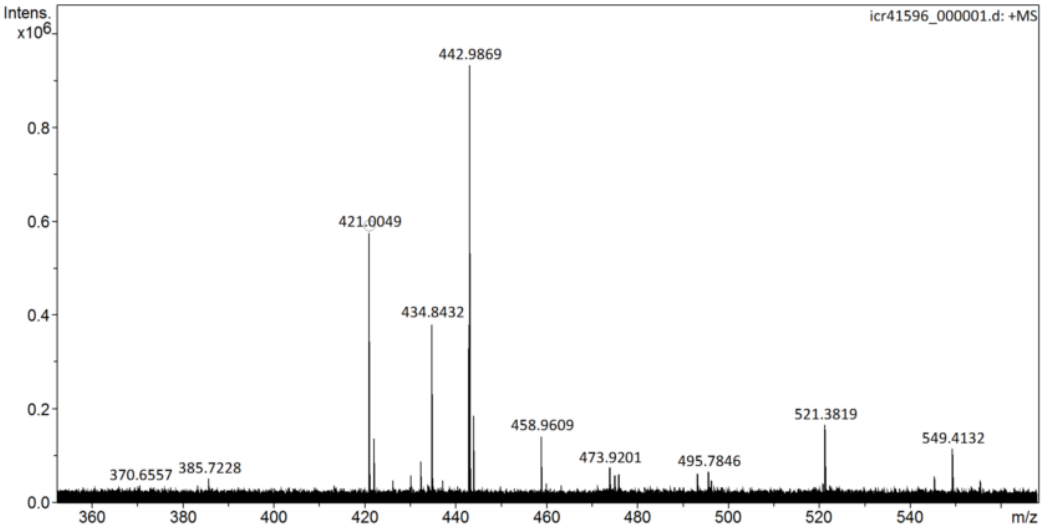

Figure S196: HR-MS Spectrum (ESI+) of compound 50.

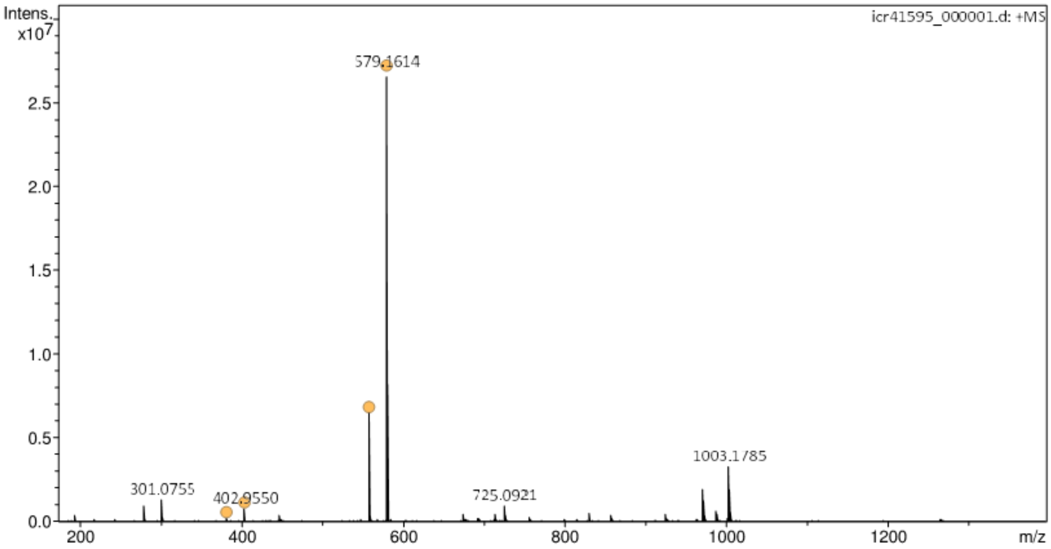

Figure S197: HR-MS Spectrum (ESI+) of compound 51c.

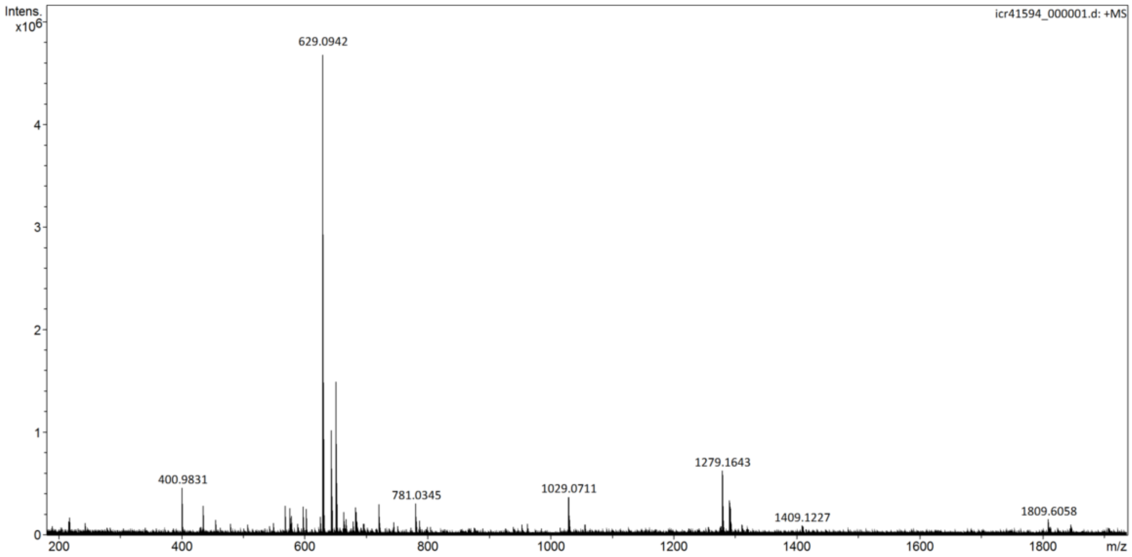

Figure S198: HR-MS Spectrum (ESI+) of compound 52c.

Fragmentor Voltage	Collision Energy	Ionization Mode
175	0	ESI

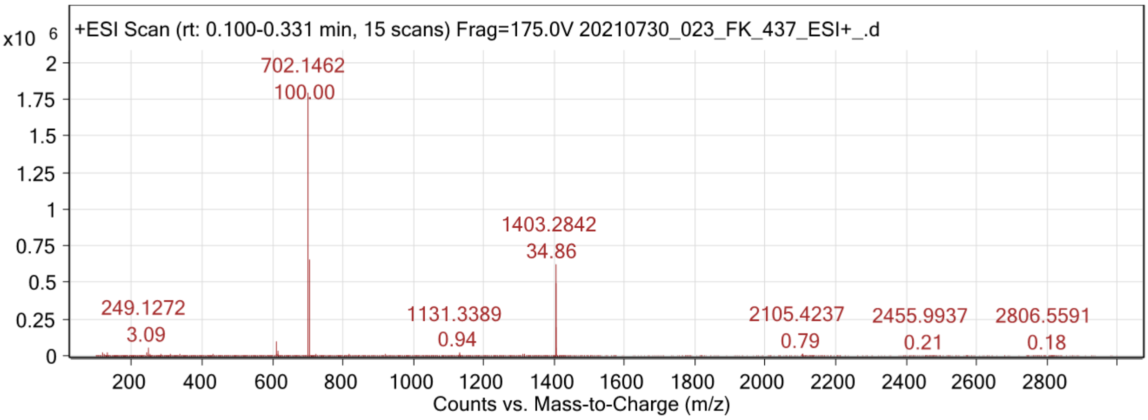

Figure S199: HR-MS Spectrum (ESI+) of compound 53c.

Fragmentor Voltage	Collision Energy	Ionization Mode
175	0	ESI

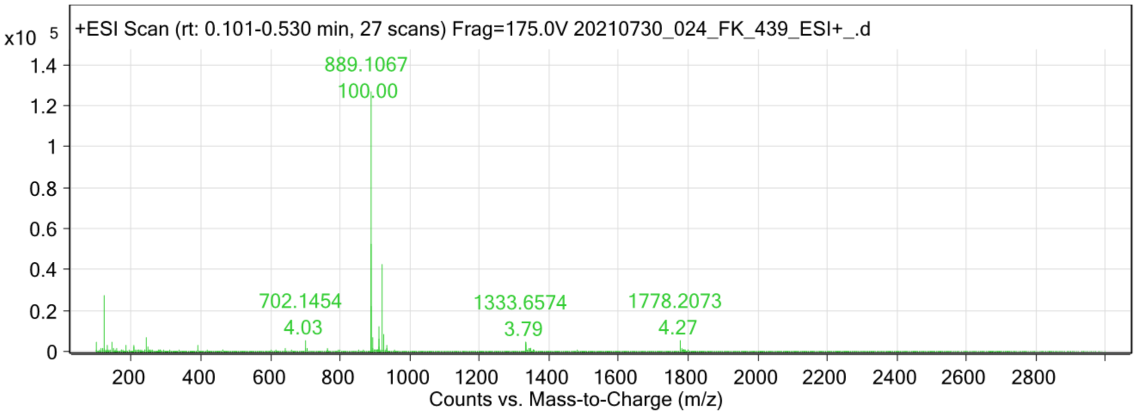

Figure S200: HR-MS Spectrum (ESI+) of compound 54c.

Fragmentor Voltage	Collision Energy	Ionization Mode
175	0	ESI

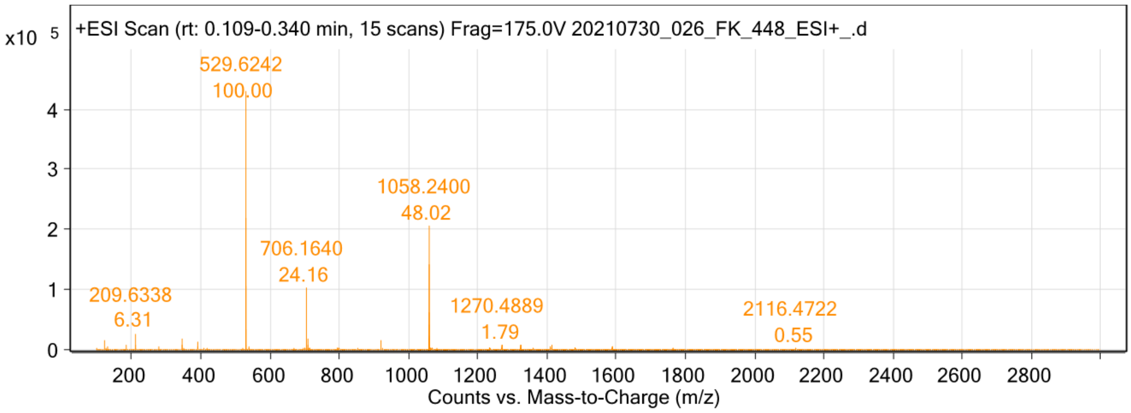

Figure S201: HR-MS Spectrum (ESI+) of compound 55c.

Fragmentor Voltage	Collision Energy	Ionization Mode
175	0	ESI

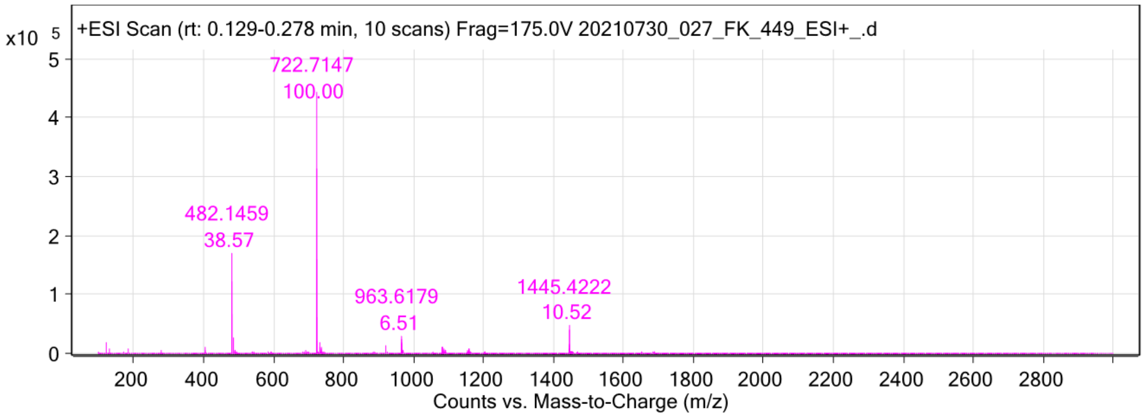

Figure S202: HR-MS Spectrum (ESI+) of compound 56c.

5. HPLC chromatograms of HPLC purified compounds

System A: RP-HPLC, analytical (Agilent Zorbax 300 C-18, 5 µm, 4.6 x 150 mm) with 10-95% acetonitrile (0.1% TFA) in water (0.1% TFA) in a linear gradient over 15 min, 1 mL/min.

System E: RP-HPLC, analytical (Kintetex® 5 µm Phenyl-Hexyl 100 Å) with 5–95% acetonitrile (0.1% TFA) in water (0.1% TFA) in a linear gradient over 10 min, 1 mL/min.

System F: Size-Exclusion-Chromatography (SEC), analytical (Agilent 8 µM PL aquagel-OH 30, 300 x 7.5 mm) with 100% PBS-buffer pH 7 isocratic over 20 min, 1 mL/min.

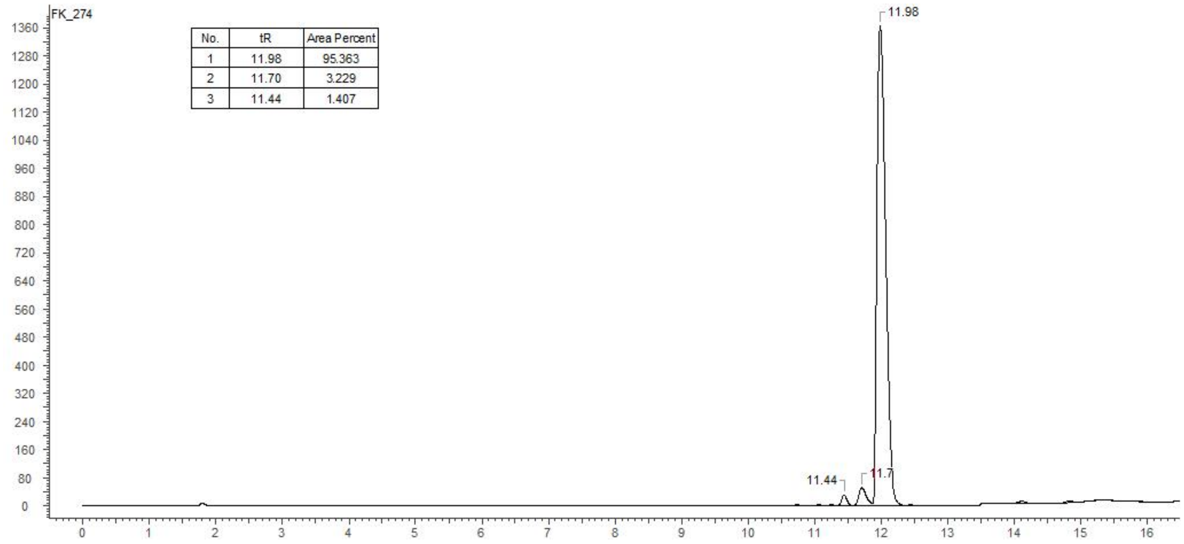

Figure S203: Analytical RP-HPLC chromatogram (System A) of compound 30a.

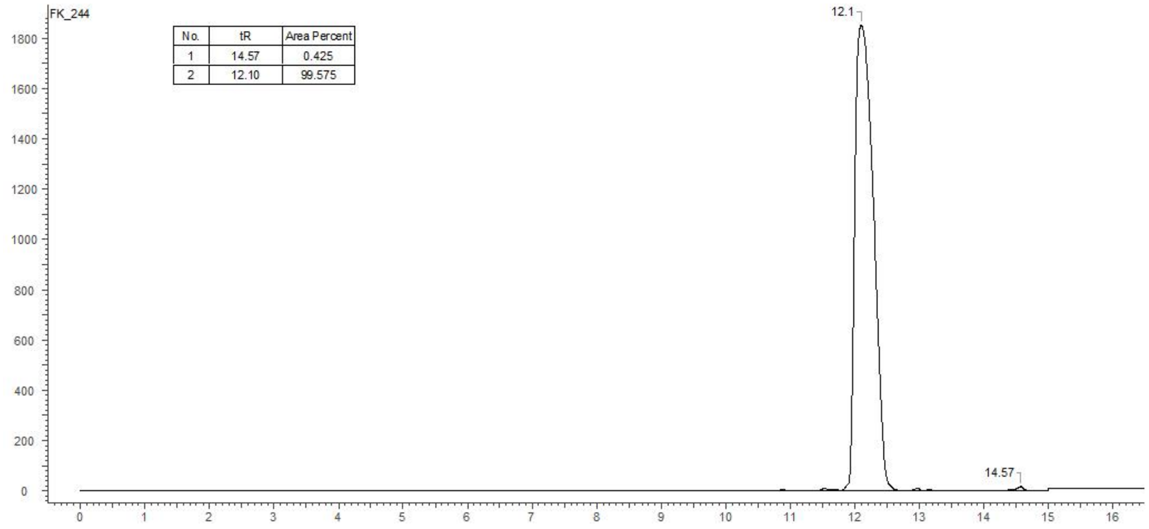

Figure S204: Analytical RP-HPLC chromatogram (System A) of compound 30b.

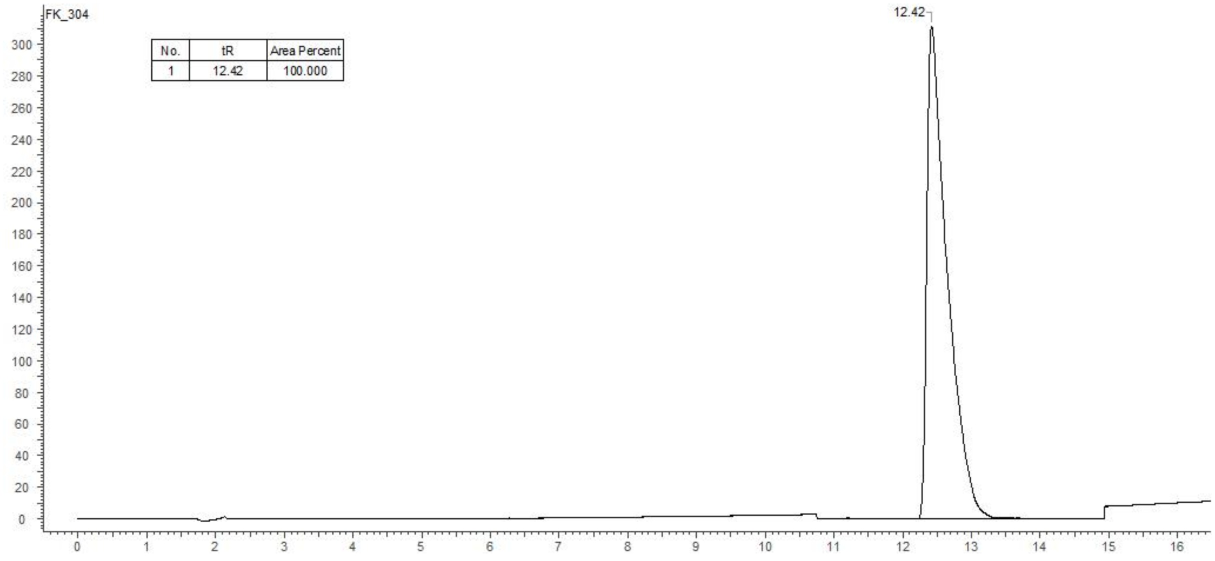

Figure S205: Analytical RP-HPLC chromatogram (System A) of compound 33a.

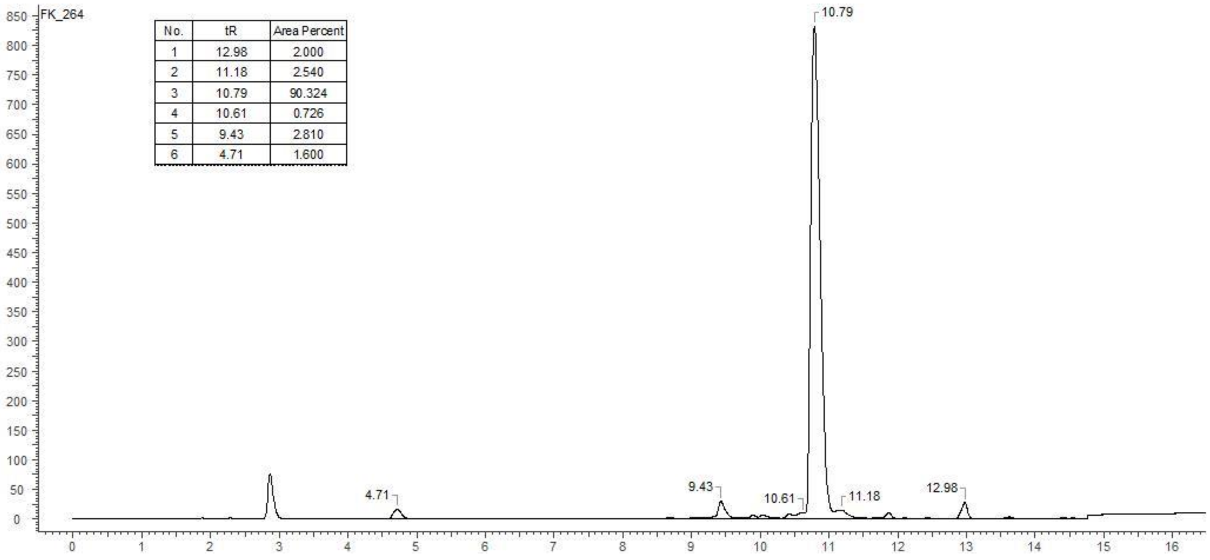

No.	tR	Area Percent
1	12.98	2.000
2	11.18	2.540
3	10.79	90.324
4	10.61	0.726
5	9.43	2.810
6	4.71	1.600

Figure S206: Analytical RP-HPLC chromatogram (System A) of compound 33b.

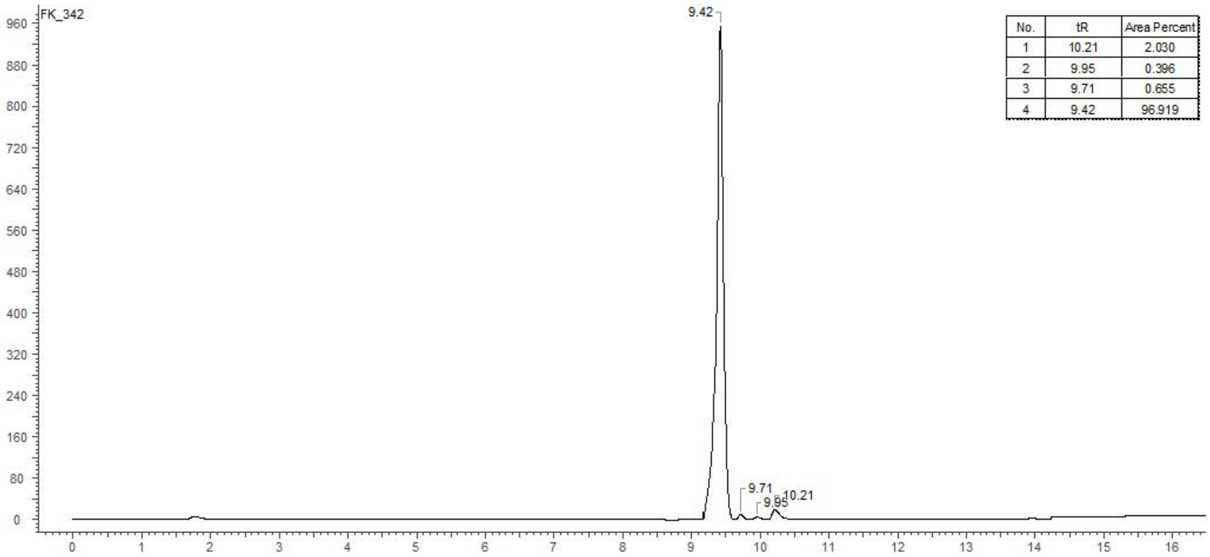

No.	tR	Area Percent
1	10.21	2.030
2	9.95	0.396
3	9.71	0.655
4	9.42	96.919

Figure S207: Analytical RP-HPLC chromatogram (System A) of compound 40a.

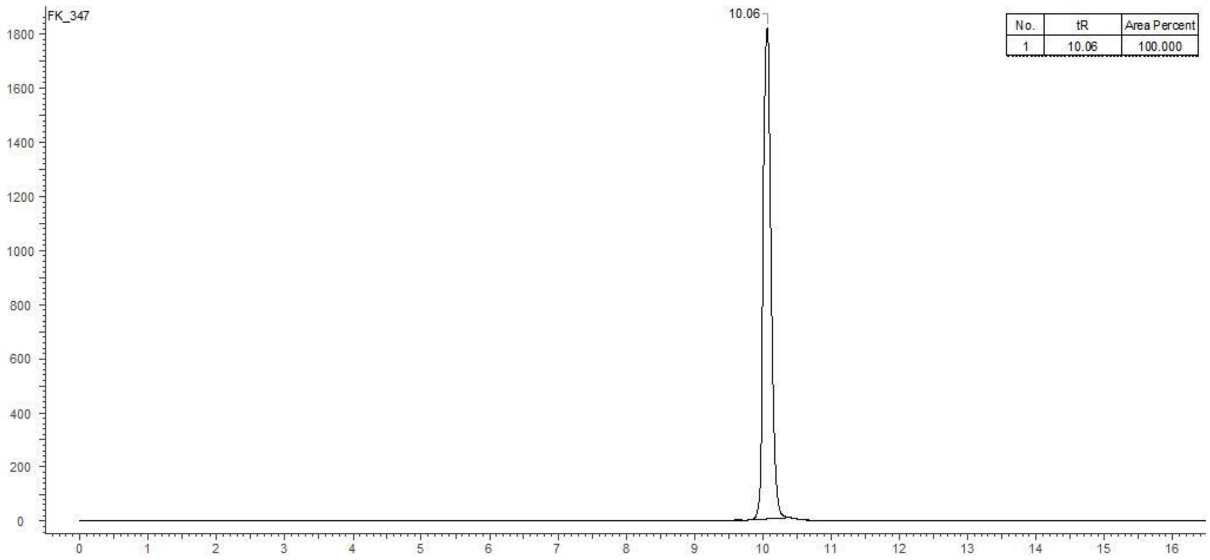

No.	tR	Area Percent
1	10.06	100.000

Figure S208: Analytical RP-HPLC chromatogram (System A) of compound 40b.

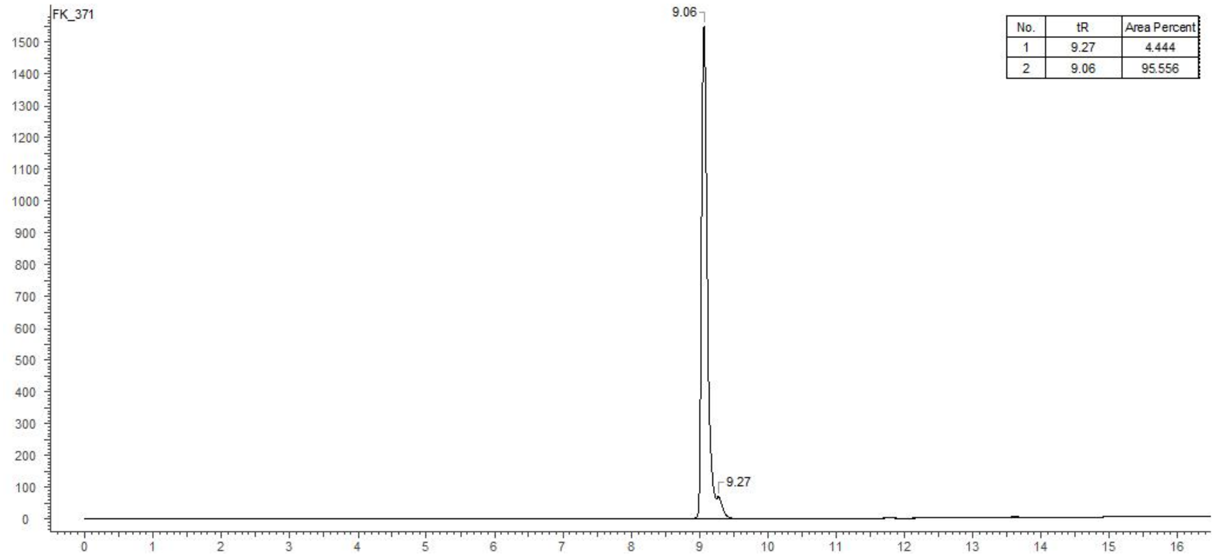

Figure S209: Analytical RP-HPLC chromatogram (System A) of compound 40c.

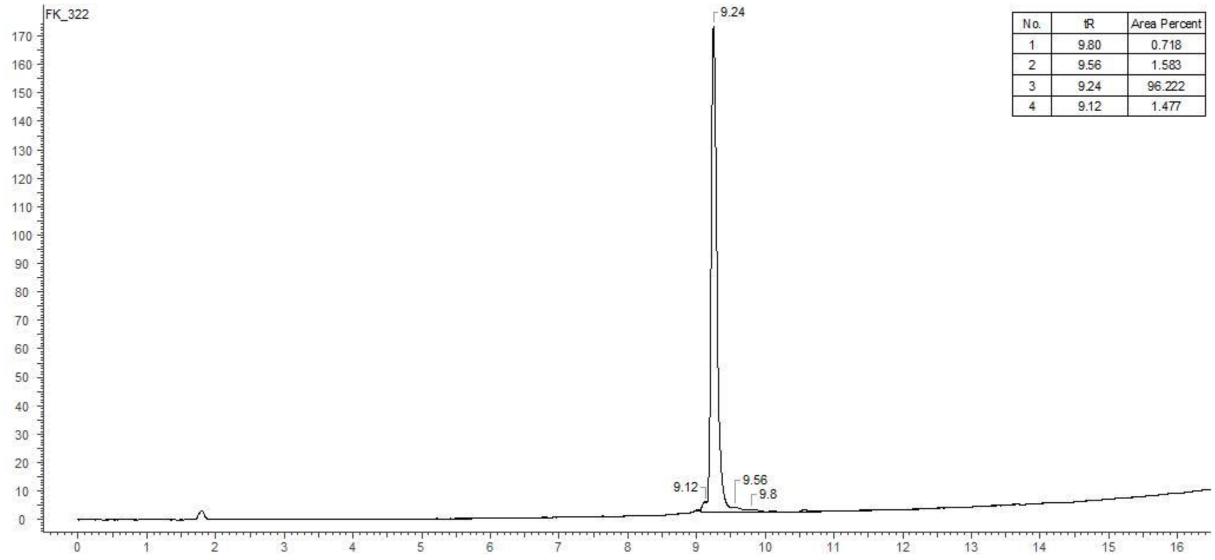

Figure S210: Analytical RP-HPLC chromatogram (System A) of compound 41a.

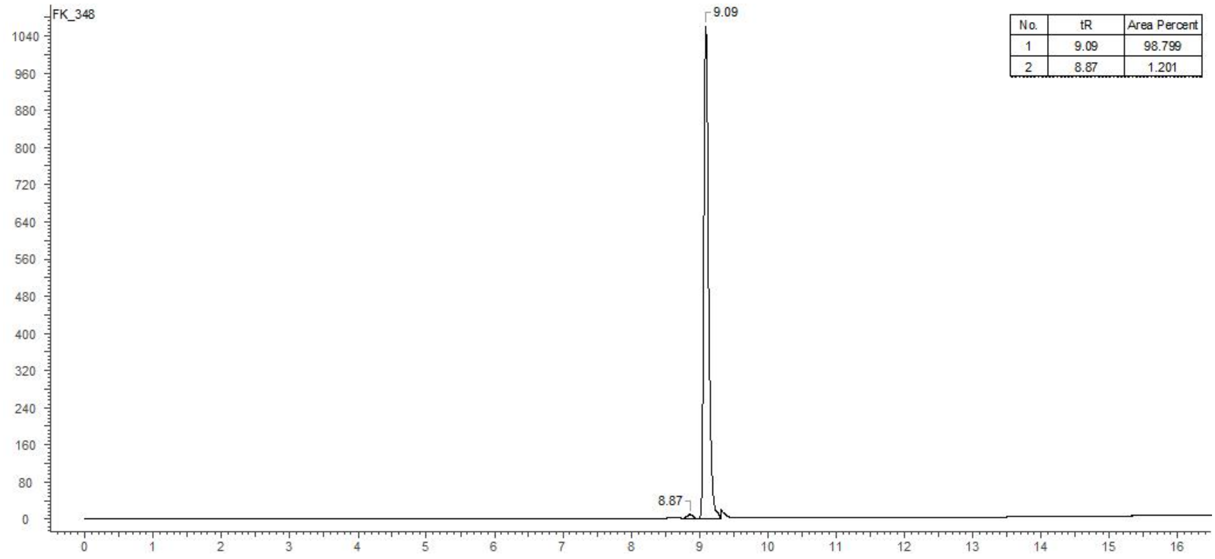

Figure S211: Analytical RP-HPLC chromatogram (System A) of compound 41b.

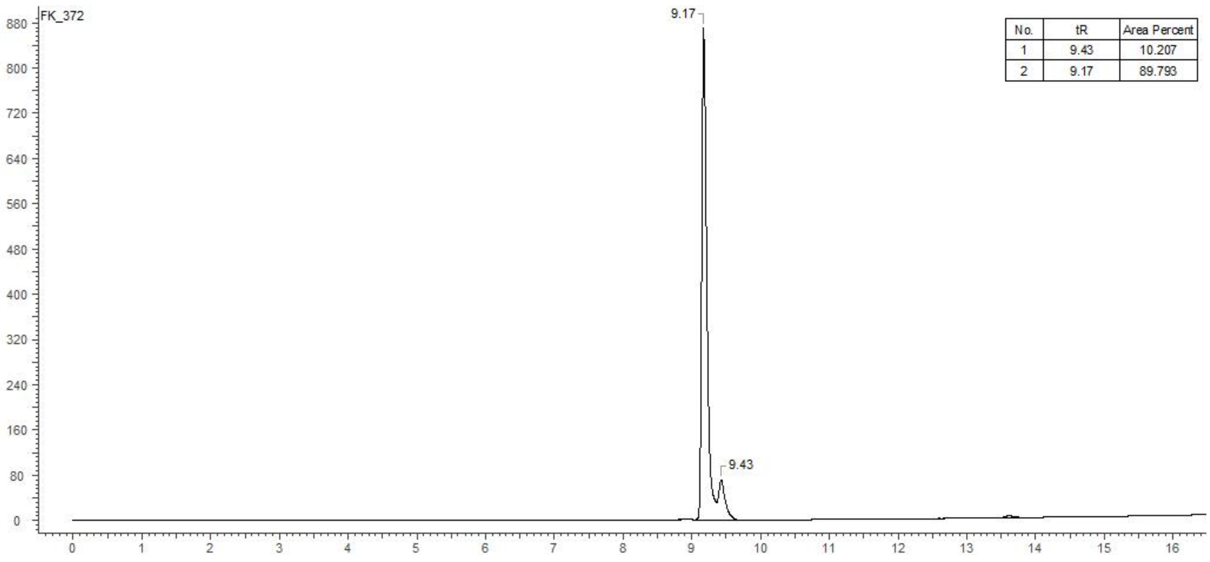

Figure S212: Analytical RP-HPLC chromatogram (System A) of compound 41c.

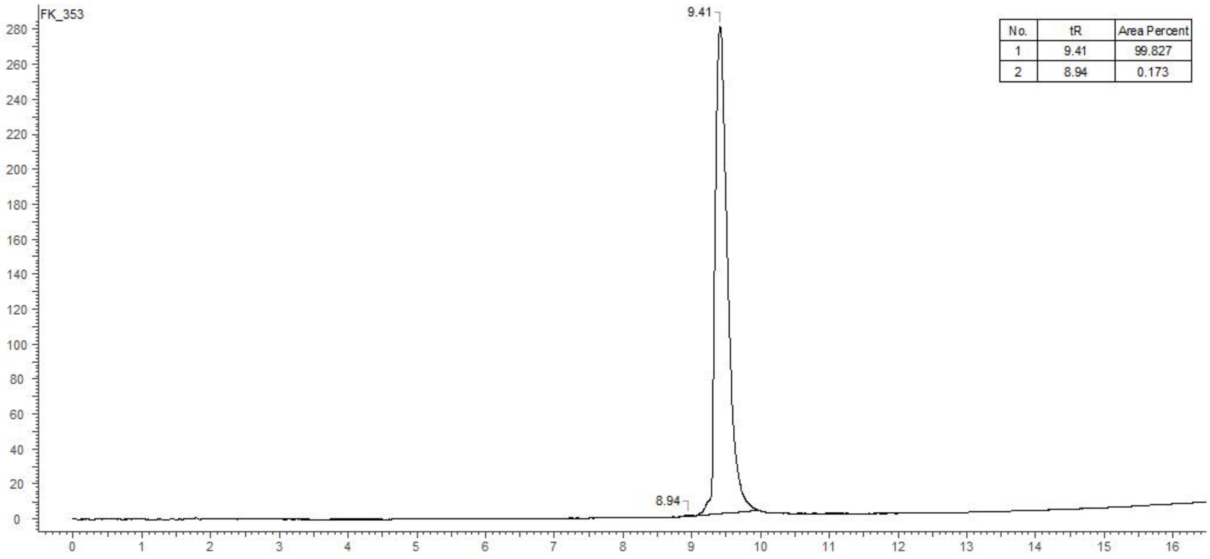

Figure S213: Analytical RP-HPLC chromatogram (System A) of compound 42a.

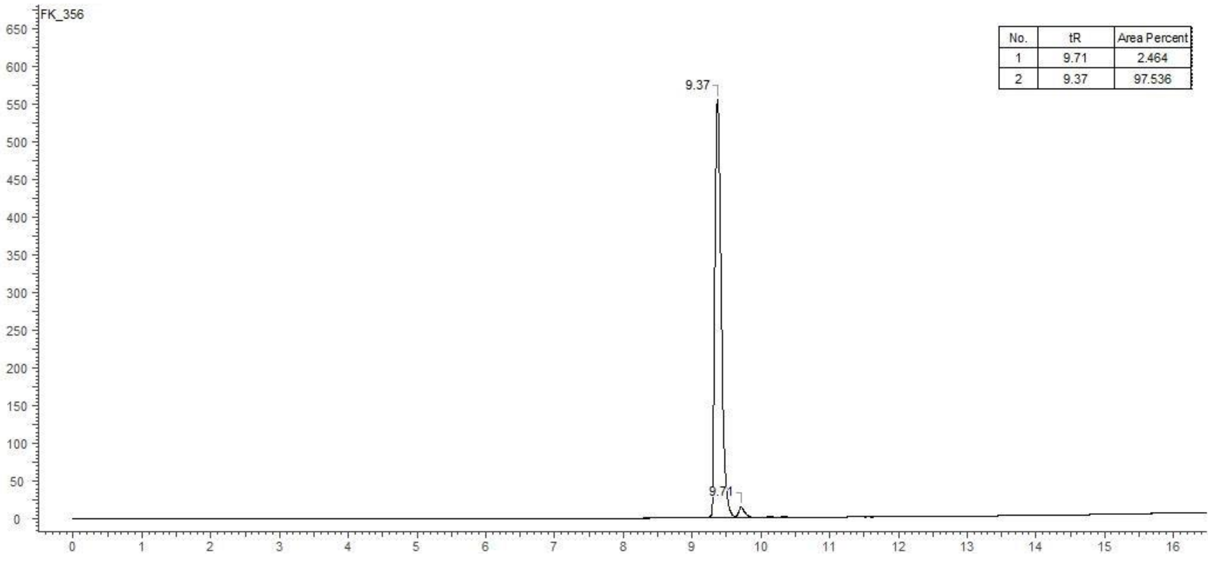

Figure S214: Analytical RP-HPLC chromatogram (System A) of compound 42b.

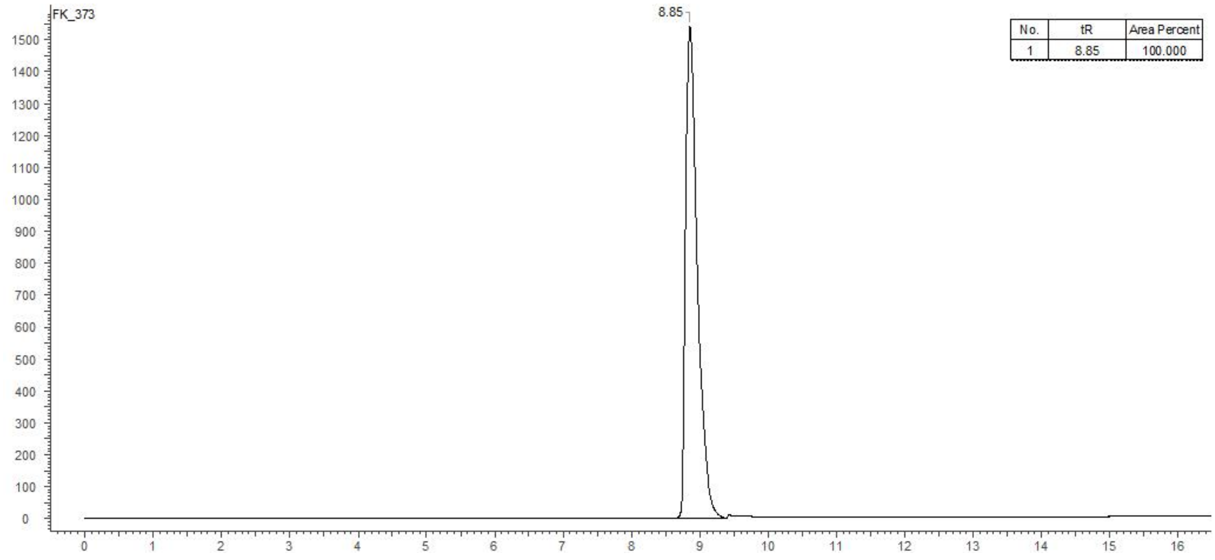

Figure S215: Analytical RP-HPLC chromatogram (System A) of compound 42c.

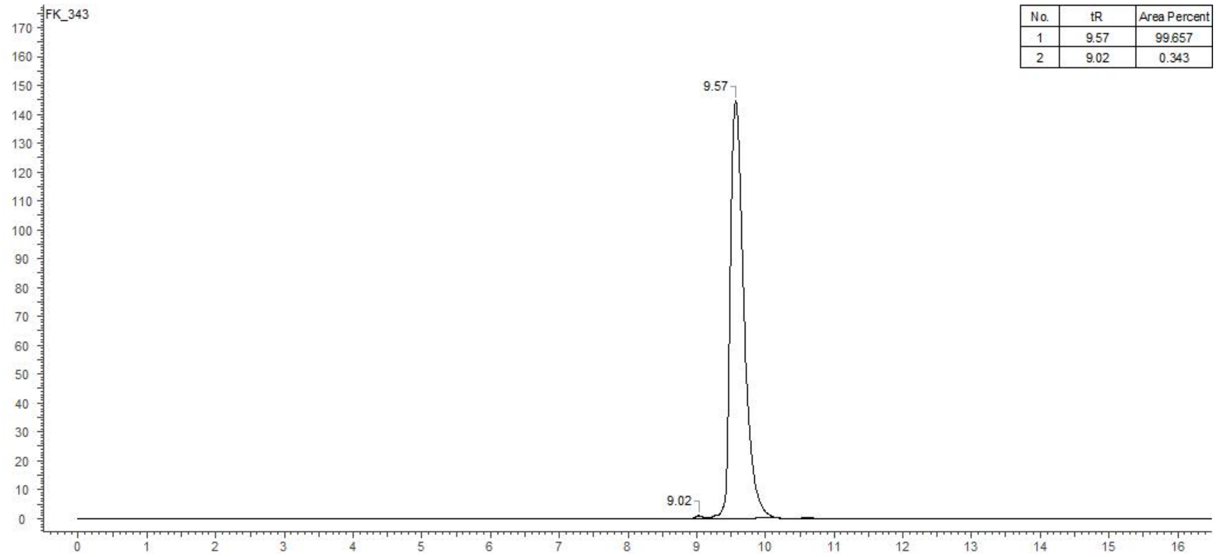

Figure S216: Analytical RP-HPLC chromatogram (System A) of compound 43a.

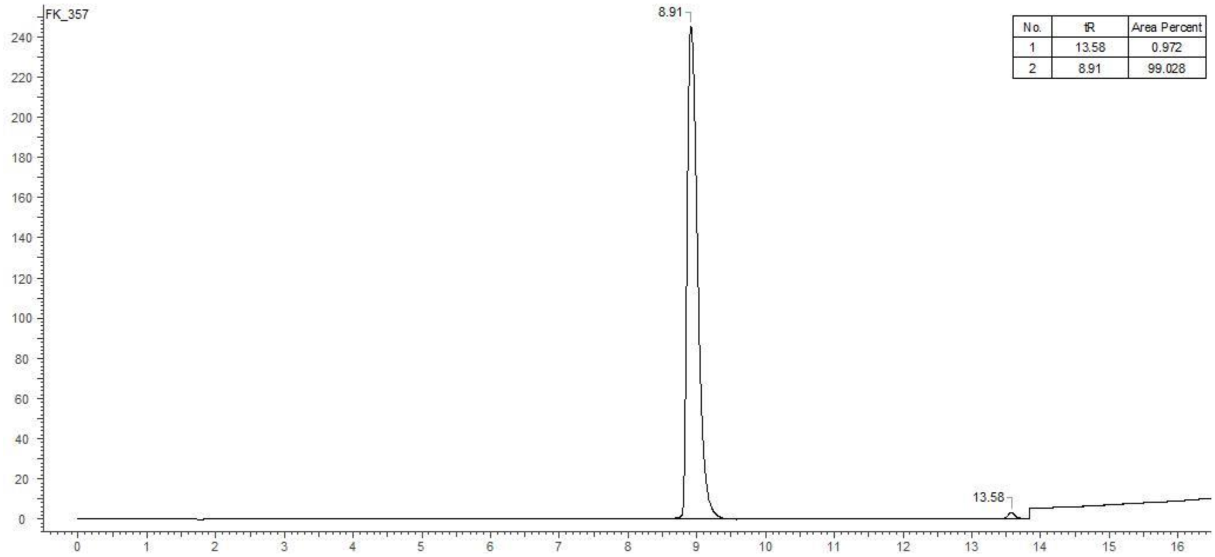

Figure S217: Analytical RP-HPLC chromatogram (System A) of compound 43b.

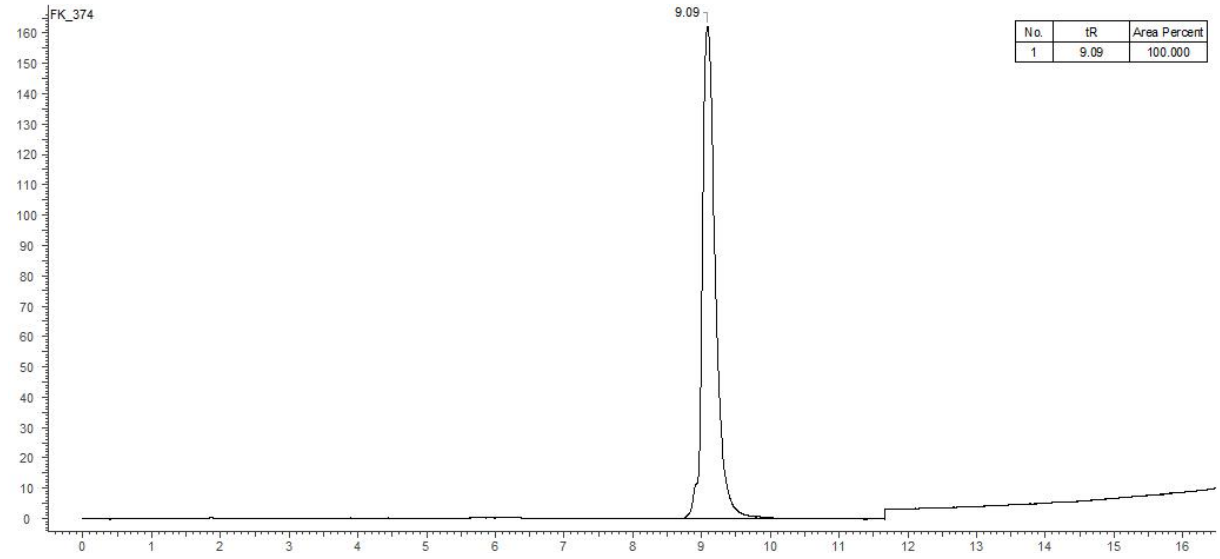

Figure S218: Analytical RP-HPLC chromatogram (System A) of compound 43c.

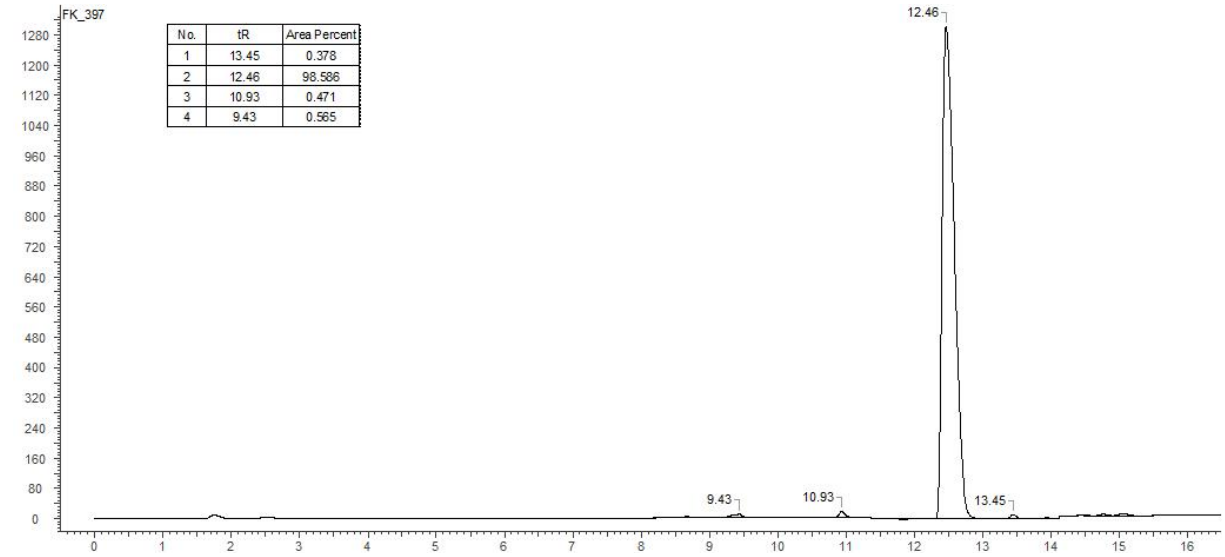

Figure S219: Analytical RP-HPLC chromatogram (System A) of compound 53a.

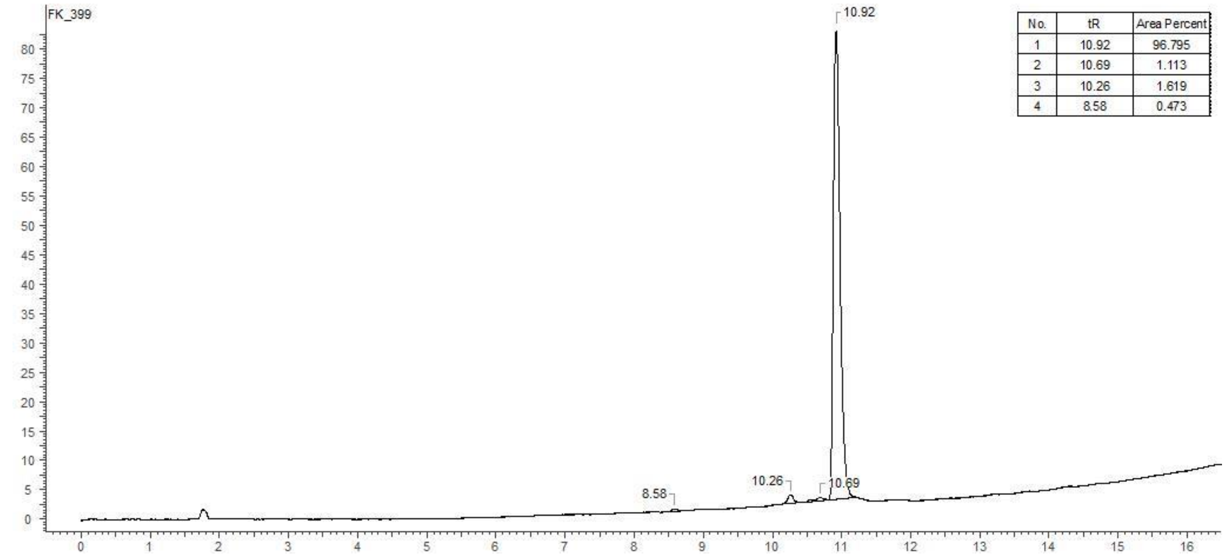

Figure S220: Analytical RP-HPLC chromatogram (System A) of compound 54a.

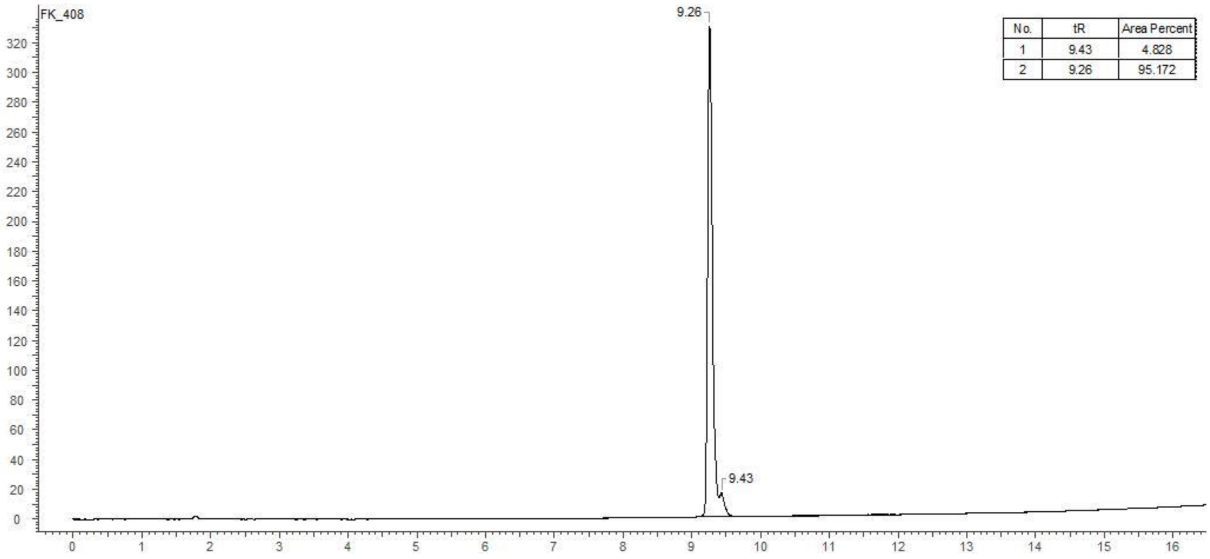

No.	tR	Area Percent
1	9.43	4.828
2	9.26	95.172

Figure S221: Analytical RP-HPLC chromatogram (System A) of compound 55a.

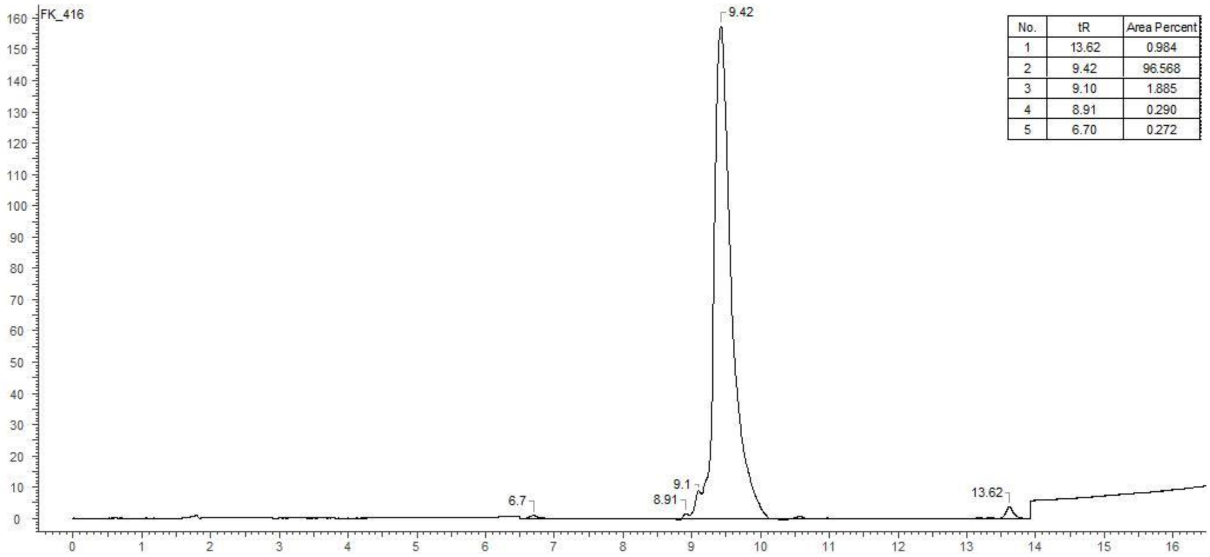

No.	tR	Area Percent
1	13.62	0.984
2	9.42	96.568
3	9.10	1.885
4	8.91	0.290
5	6.70	0.272

Figure S222: Analytical RP-HPLC chromatogram (System A) of compound 56a.

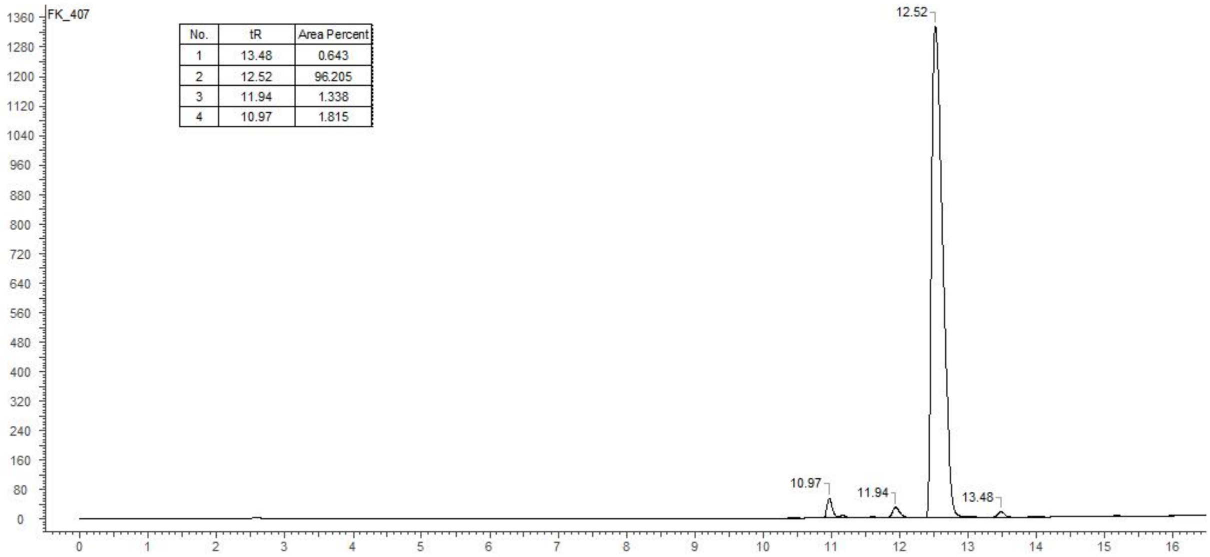

No.	tR	Area Percent
1	13.48	0.643
2	12.52	96.205
3	11.94	1.338
4	10.97	1.815

Figure S223: Analytical RP-HPLC chromatogram (System A) of compound 53b.

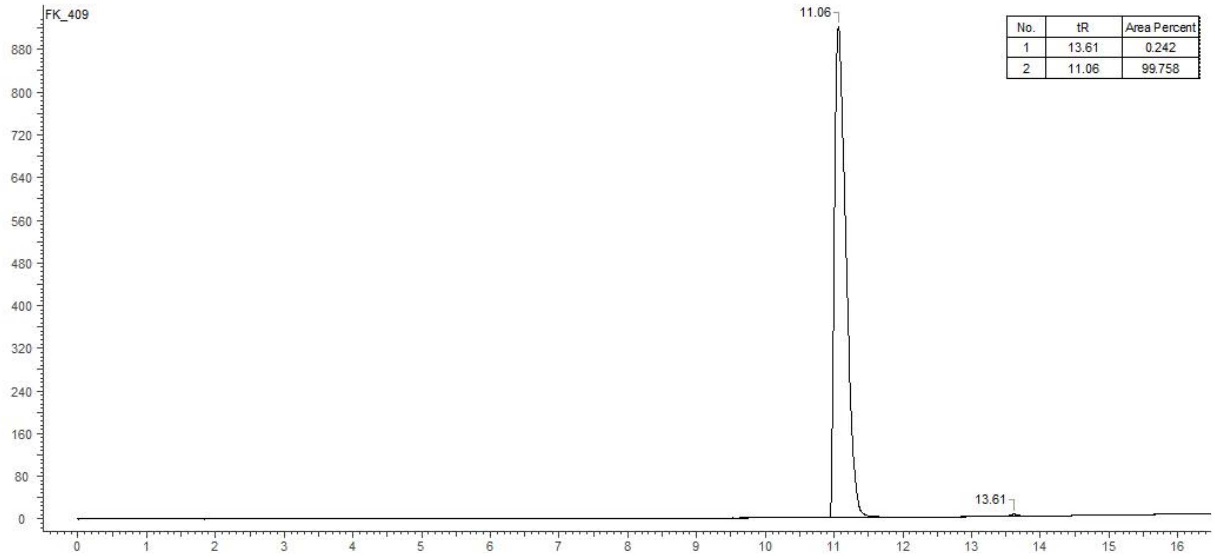

Figure S224: Analytical RP-HPLC chromatogram (System A) of compound 54b.

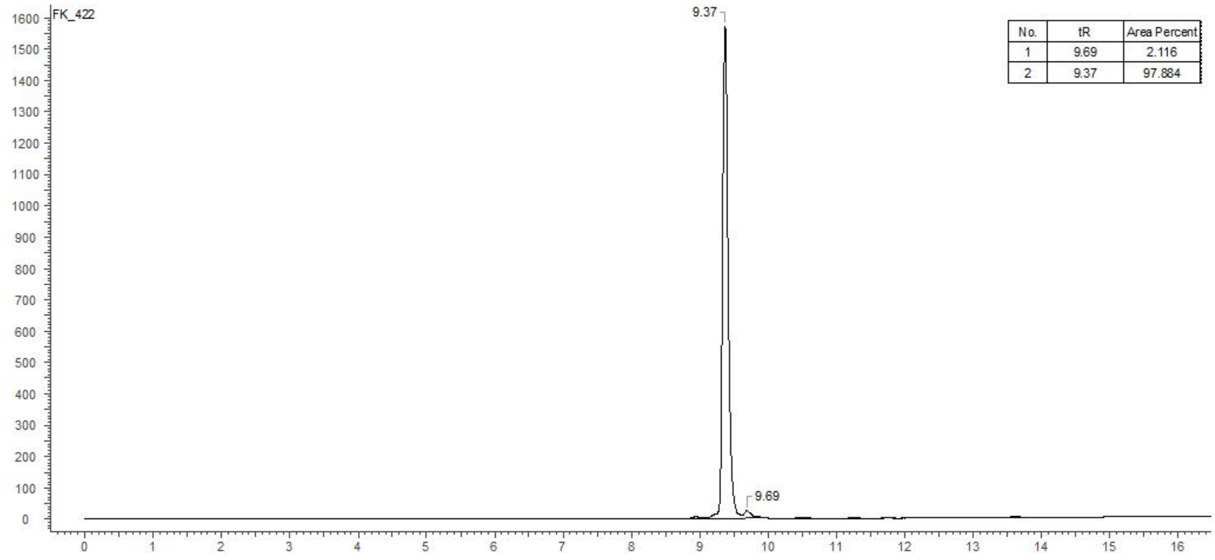

Figure S225: Analytical RP-HPLC chromatogram (System A) of compound 55b.

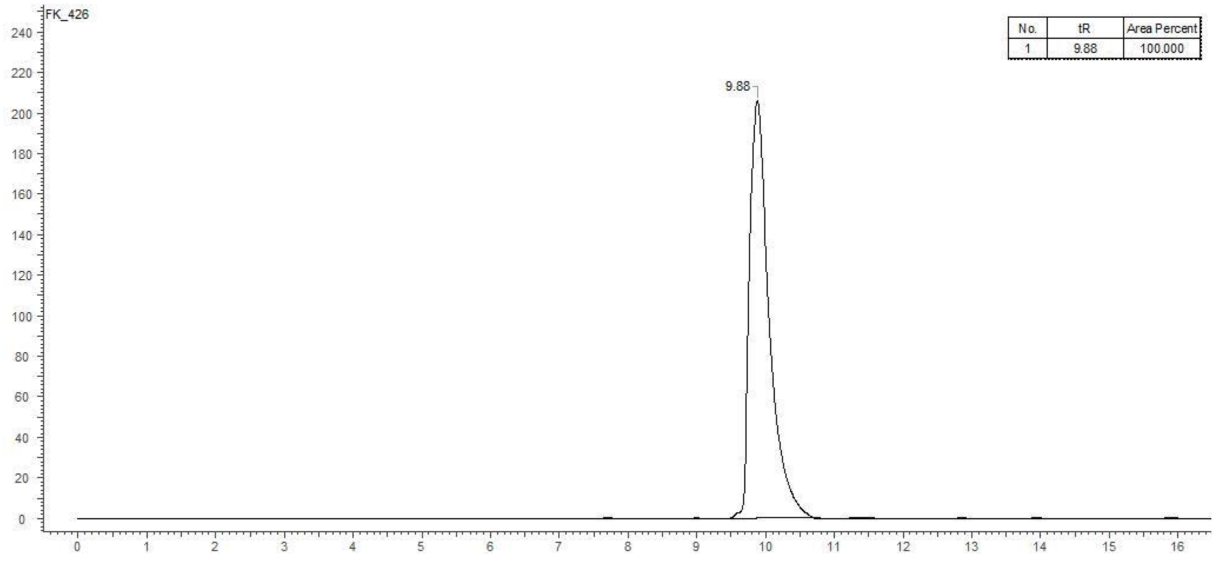

Figure S226: Analytical RP-HPLC chromatogram (System A) of compound 56b.

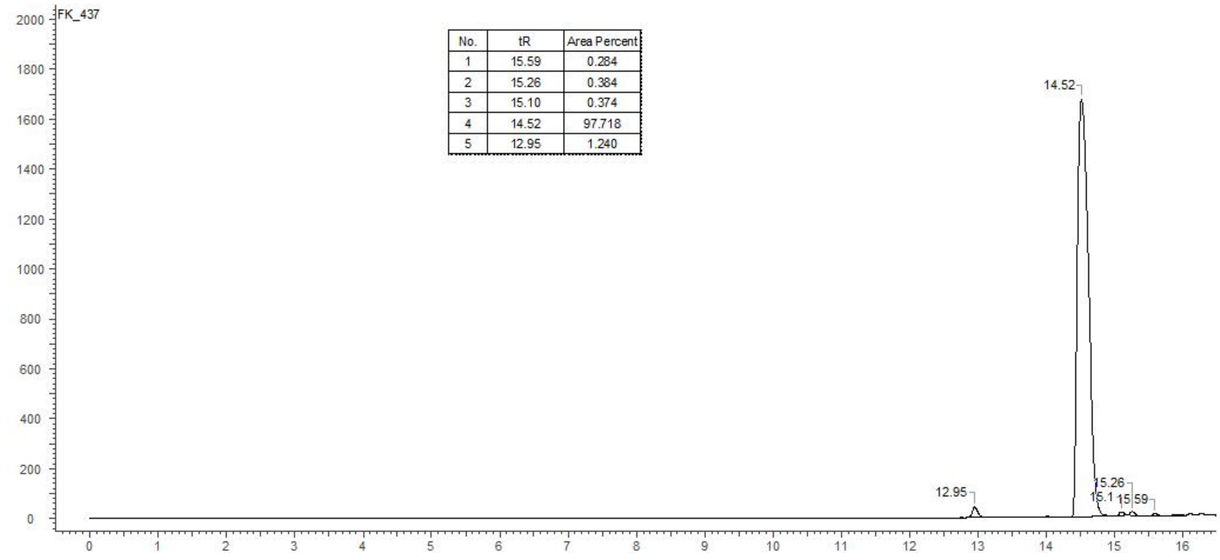

Figure S227: Analytical RP-HPLC chromatogram (System A) of compound 53c.

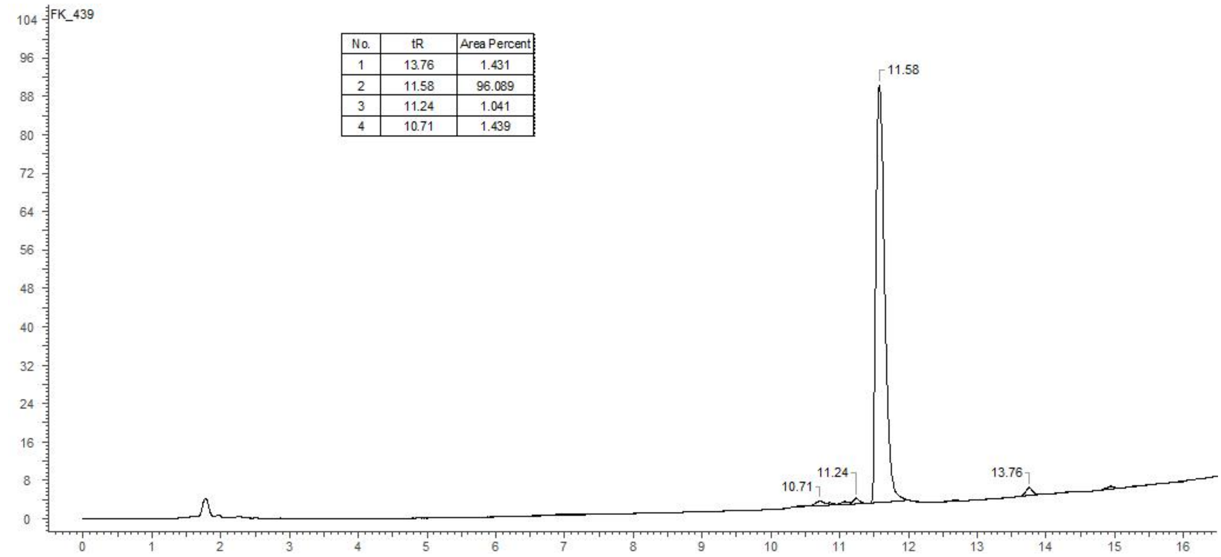

Figure S228: Analytical RP-HPLC chromatogram (System A) of compound 54c.

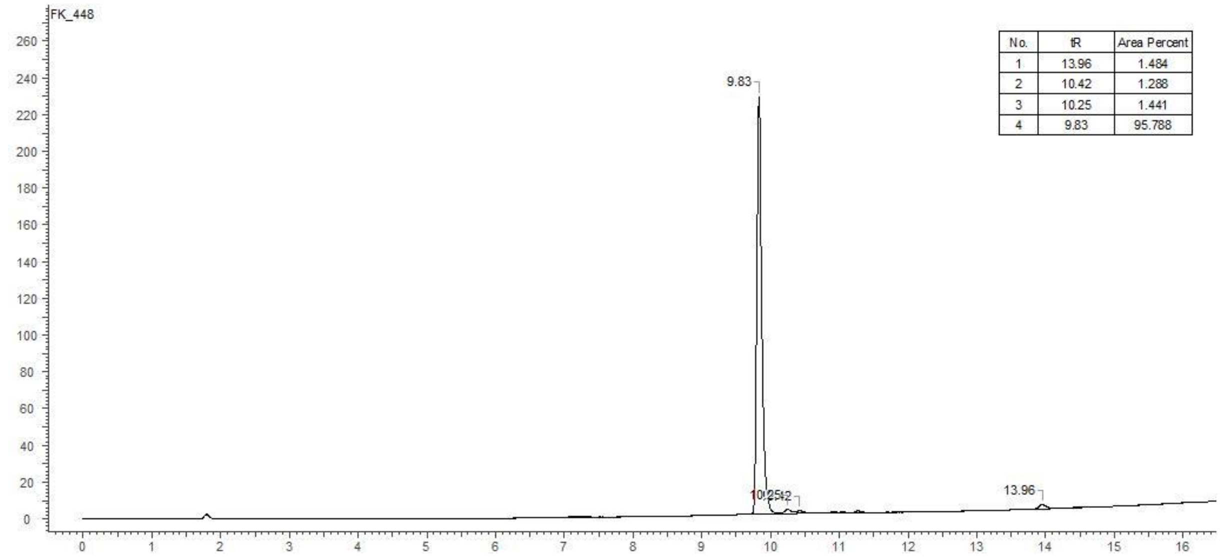

Figure S229: Analytical RP-HPLC chromatogram (System A) of compound 55c.

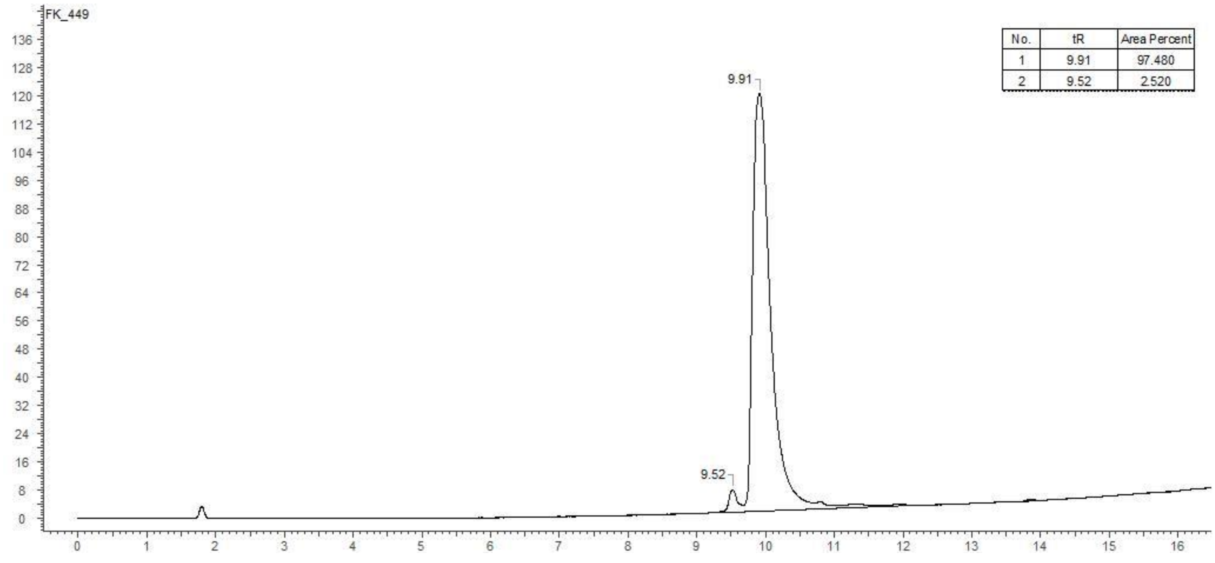

Figure S230: Analytical RP-HPLC chromatogram (System A) of compound 56c.

6. Radio-HPLC chromatograms of labeled radiotracers

Exemplary Radio-HPLC RP-HPLC chromatograms of compounds [^{64}Cu]Cu-**56a**, [^{64}Cu]Cu-**56b** and [^{64}Cu]Cu-**56c**.

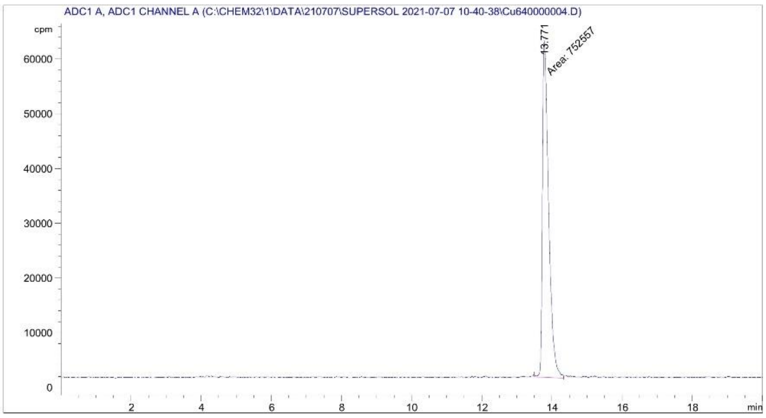


```
=====================================================================
                         Area Percent Report
=====================================================================

Sorted By             :      Signal
Multiplier            :      1.0000
Dilution              :      1.0000
Do not use Multiplier & Dilution Factor with ISTDs


Signal 1: ADC1 A, ADC1 CHANNEL A

Peak RetTime Type  Width     Area      Height     Area
  #   [min]        [min]   [cpm*s]     [cpm]        %
----|-------|----|-------|----------|----------|--------|
   1  13.771 MM    0.2036 7.52557e5  6.15922e4  100.0000

Totals :                  7.52557e5  6.15922e4
```

Figure S231: Radio-HPLC chromatogram of [^{64}Cu]Cu-56a.

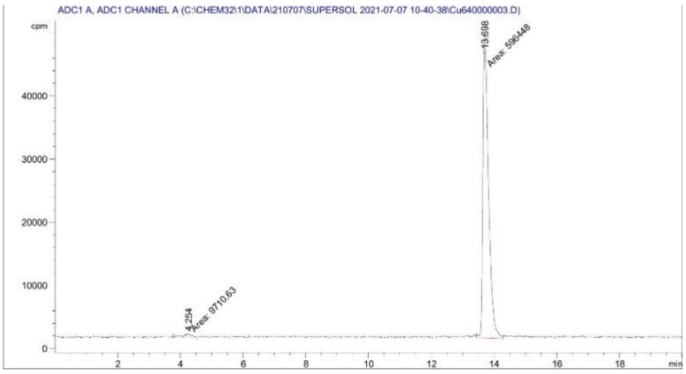


```
=====================================================================
                         Area Percent Report
=====================================================================

Sorted By             :      Signal
Multiplier            :      1.0000
Dilution              :      1.0000
Do not use Multiplier & Dilution Factor with ISTDs


Signal 1: ADC1 A, ADC1 CHANNEL A

Peak RetTime Type  Width     Area      Height     Area
  #   [min]        [min]   [cpm*s]     [cpm]        %
----|-------|----|-------|----------|----------|--------|
   1   4.254 MM    0.3021 9710.63379  535.70636   1.6020
   2  13.698 MM    0.2078 5.96448e5  4.78415e4   98.3980

Totals :                  6.06158e5  4.83772e4
```

Figure S232: Radio-HPLC chromatogram of [^{64}Cu]Cu-56b.

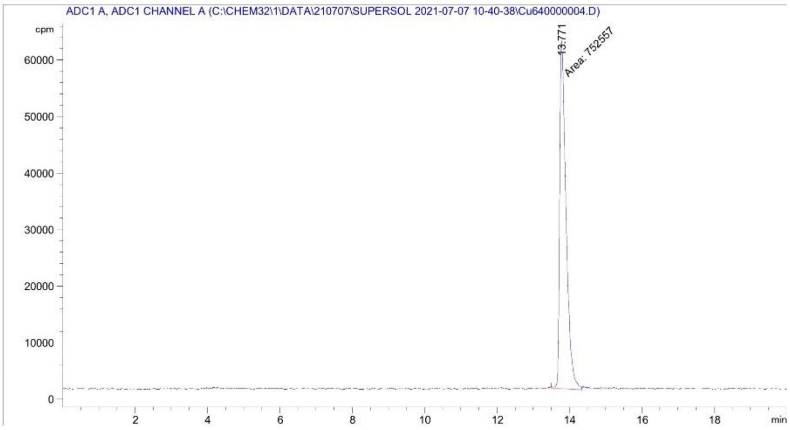


```
=====================================================================
                         Area Percent Report
=====================================================================

Sorted By             :      Signal
Multiplier            :      1.0000
Dilution              :      1.0000
Do not use Multiplier & Dilution Factor with ISTDs


Signal 1: ADC1 A, ADC1 CHANNEL A

Peak RetTime Type  Width     Area      Height     Area
  #   [min]        [min]   [cpm*s]     [cpm]        %
----|-------|----|-------|----------|----------|--------|
   1  13.771 MM    0.2036 7.52557e5  6.15922e4  100.0000

Totals :                  7.52557e5  6.15922e4
```

Figure S233: Radio-HPLC chromatogram of [^{64}Cu]Cu-56c.

7. Stability of radiolabeled compounds

8.1 Kinetic stability in buffer solutions

a)

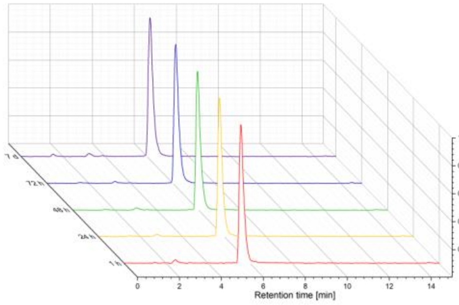

b)

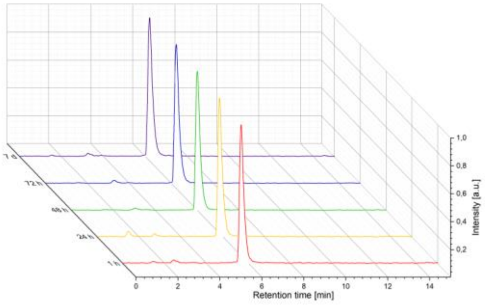

c)

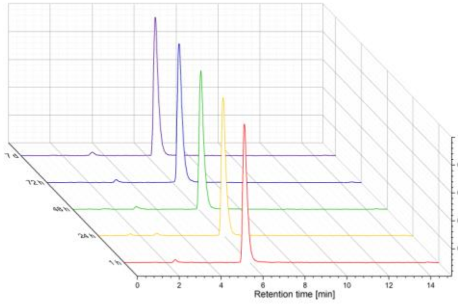

d)

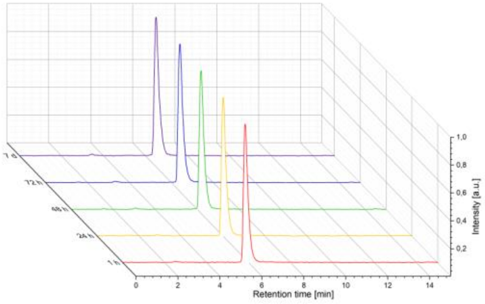

Figure S234: Radio-HPLC chromatograms (System E) of ^{177}Lu-labeled compounds a) [^{177}Lu]Lu-42c; b) [^{177}Lu]Lu-43c; c) [^{177}Lu]Lu-56b; d) [^{177}Lu]Lu-56c incubated in 1 M HEPES solution for 1 h (red), 24 h (yellow), 48 h (green), 72 h (blue) and 7 d (purple).

a)

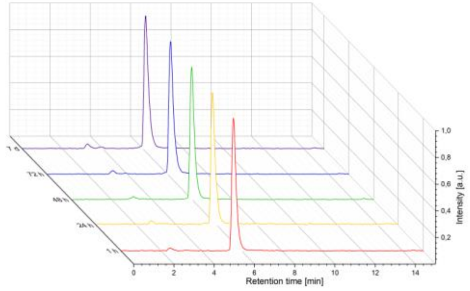

b)

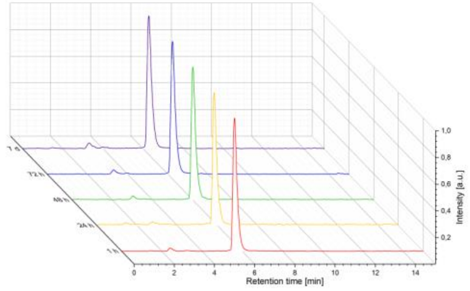

c)

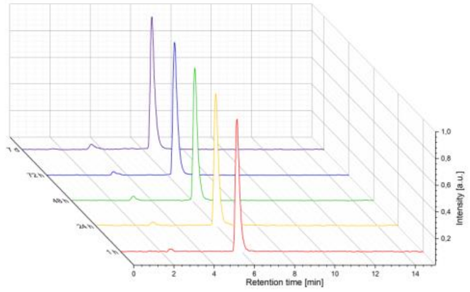

d)

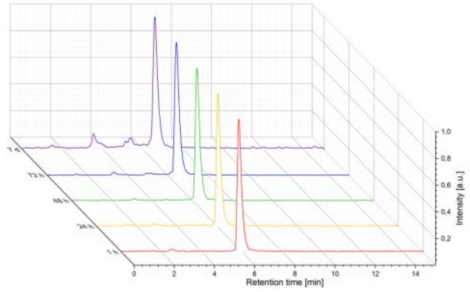

Figure S235: Radio-HPLC chromatograms (System E) of ^{177}Lu-labeled compounds a) [^{177}Lu]Lu-42c; b) [^{177}Lu]Lu-43c; c) [^{177}Lu]Lu-56b; d) [^{177}Lu]Lu-56c incubated in PBS solution for 1 h (red), 24 h (yellow), 48 h (green), 72 h (blue) and 7 d (purple).

8.2 Proteolytic stability in human serum

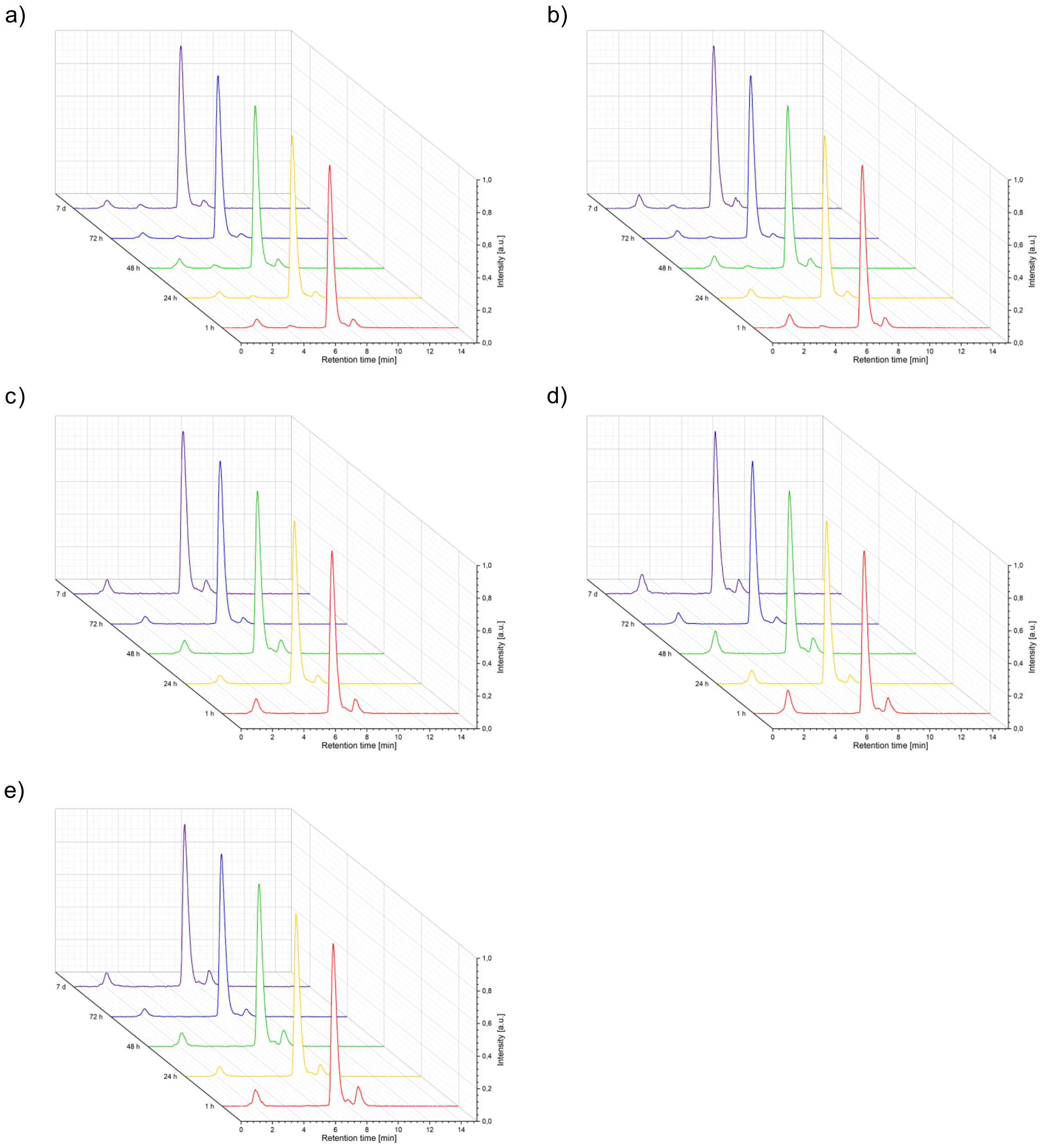

Figure S236: Radio-HPLC chromatograms (System E) of ^{177}Lu-labeled compounds a) [^{177}Lu]Lu-42c; b) [^{177}Lu]Lu-43c; c) [^{177}Lu]Lu-56a d) [^{177}Lu]Lu-56b; e) [^{177}Lu]Lu-56c after incubation in human serum for 1 h (red), 24 h (yellow), 48 h (green), 72 h (blue) and 7 d (purple) and subsequent methanol-chloroform precipitation.

8.3 Serum protein binding studies

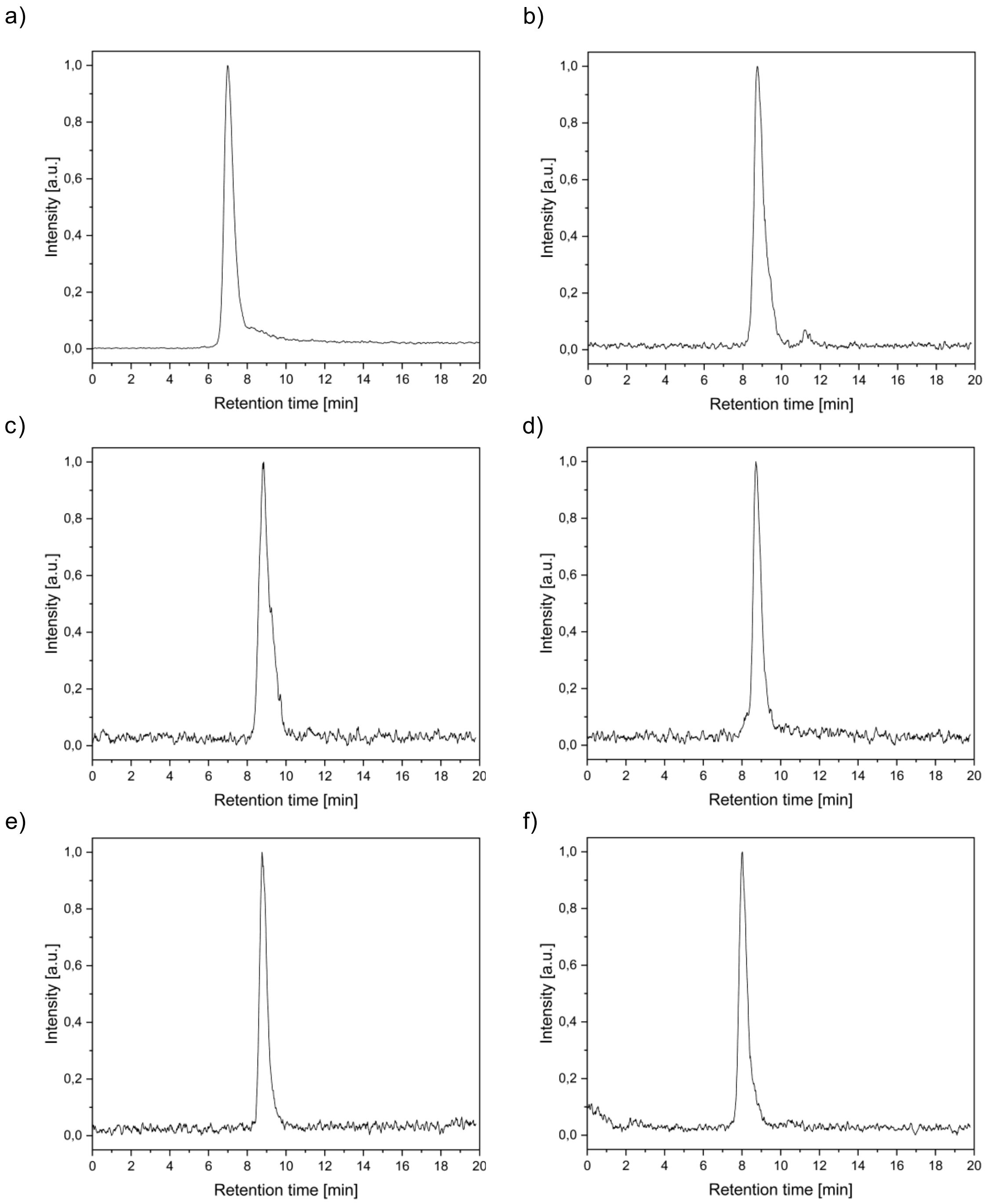

Figure S237: Radio-HPLC chromatograms (size exclusion chromatography, System F) of a) human serum (detection of UV-lane) and ^{177}Lu-labeled compounds b) [^{177}Lu]Lu-42c; c) [^{177}Lu]Lu-43c; d) [^{177}Lu]Lu-56a; e) [^{177}Lu]Lu-56b; f) [^{177}Lu]Lu-56c from reaction mixture (detection of γ-line).

a)

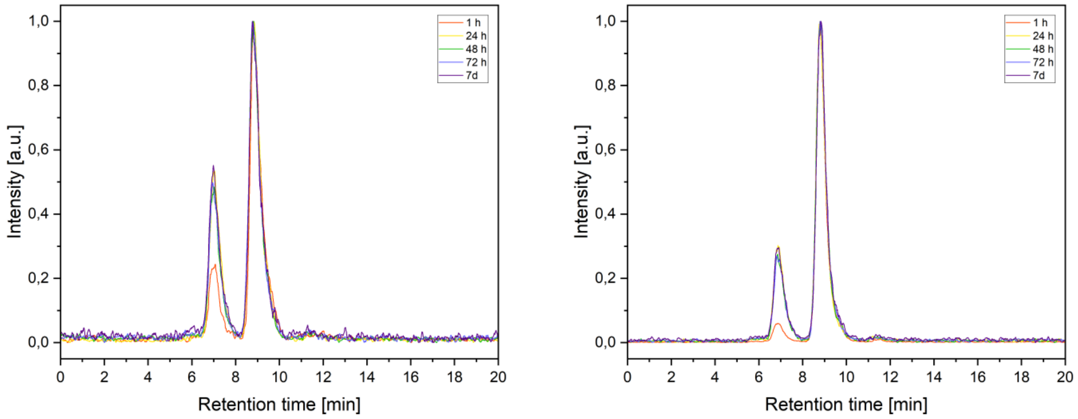
1 h
24 h
48 h
72 h
7d
Intensity [a.u.]
Retention time [min]

b)

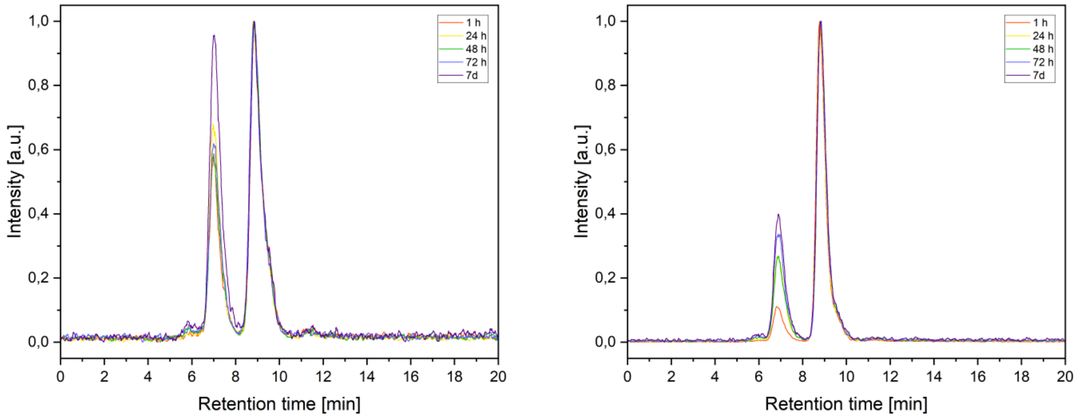
1 h
24 h
48 h
72 h
7d
Intensity [a.u.]
Retention time [min]

c)

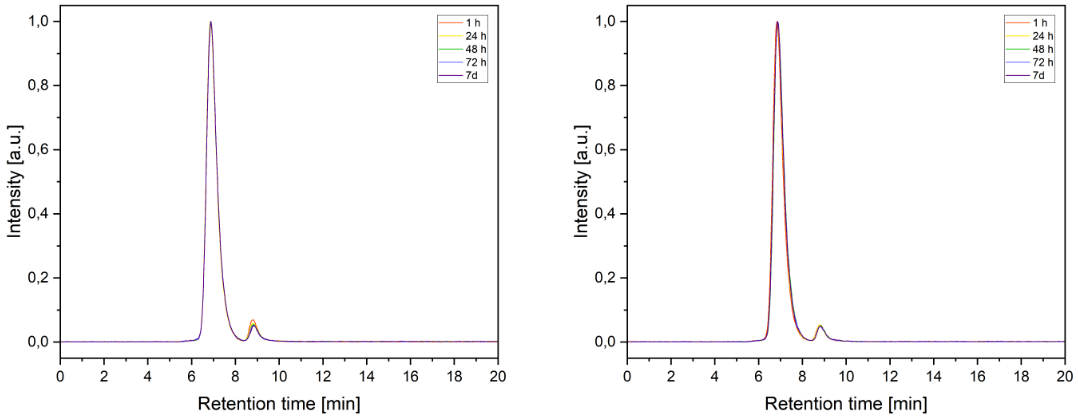
1 h
24 h
48 h
72 h
7d
Intensity [a.u.]
Retention time [min]

d)

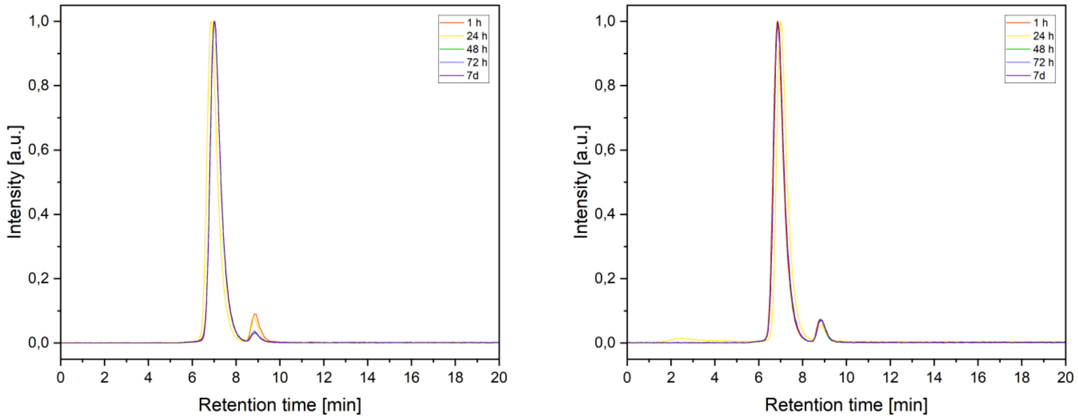

e)

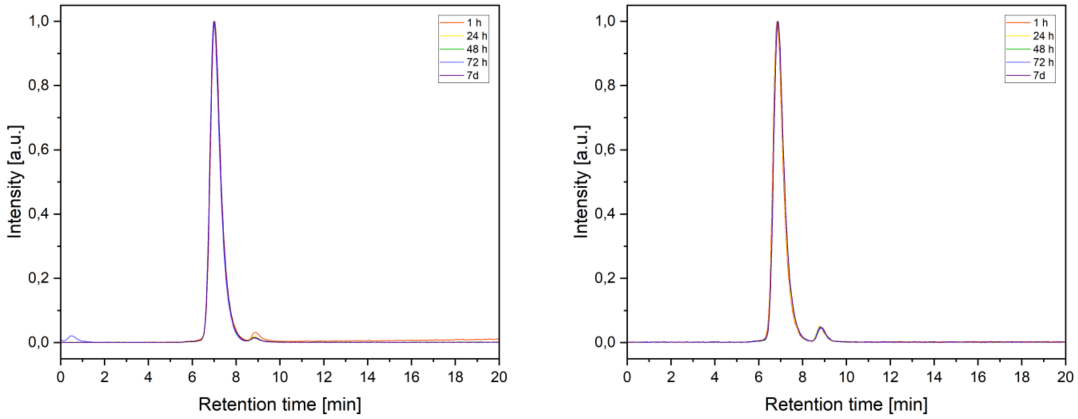

Figure S238: Radio-HPLC chromatograms (size exclusion chromatography, System F) of ^{177}Lu-labeled compounds a) [^{177}Lu]Lu-42c; b) [^{177}Lu]Lu-43c; c) [^{177}Lu]Lu-56a d) [^{177}Lu]Lu-56b; e) [^{177}Lu]Lu-56c after incubation in human serum for 1 h (red), 24 h (yellow), 48 h (green), 72 h (blue) and 7 d (purple). Shown are both duplicates of each analysis.

Table S1: Integrated peak areas in percentage of tracers a) [^{177}Lu]Lu-42c; b) [^{177}Lu]Lu-43c; c) [^{177}Lu]Lu-56a d) [^{177}Lu]Lu-56b; e) [^{177}Lu]Lu-56c bound to human serum proteins of radio-HPLC chromatograms presented in Figure S238.

	**1 h**		**24 h**		**48 h**		**72 h**		**7 d**	
	#1	*#2*	*#1*	*#2*	*#1*	*#2*	*#1*	*#2*	*#1*	*#2*
[^{177}Lu]Lu-**42c**	16.7	5.89	32.5	24.3	30	21.3	30.5	22.1	34.2	21.9
**mean ± SD**	11.3 ± 7.64		28.4 ± 5.80		25.7 ± 6.15		26.3 ± 5.94		28.1 ± 8.70	
[^{177}Lu]Lu-**43c**	32.1	10.1	37.5	22.8	32.8	22.0	35.0	28.0	48.8	29.9
**mean ± SD**	21.1 ± 15.6		30.2 ± 10.4		27.4 ± 7.64		31.5 ± 4.95		38.9 ± 14.0	
[^{177}Lu]Lu-**56a**	95.0	96.4	96.2	96.4	96.4	96.5	96.5	96.7	96.7	96.8
**mean ± SD**	95.7 ± 0.99		96.3 ± 0.14		96.5 ± 0.07		96.6 ± 0.14		96.8 ± 0.07	
[^{177}Lu]Lu-**56b**	94.2	95.0	97.1	94.6	95.0	97.8	97.8	95.1	98.1	95.1
**mean ± SD**	94.6 ± 0.57		95.9 ± 1.77		96.4 ± 1.98		96.5 ± 1.91		96.6 ± 2.12	
[^{177}Lu]Lu-**56c**	98.1	97.1	96.6	95.9	99.1	96.6	99.0	99.4	99.2	96.9
**mean ± SD**	97.6 ± 0.71		96.3 ± 0.49		97.9 ± 1.77		99.2 ± 0.28		98.1 ± 1.62	

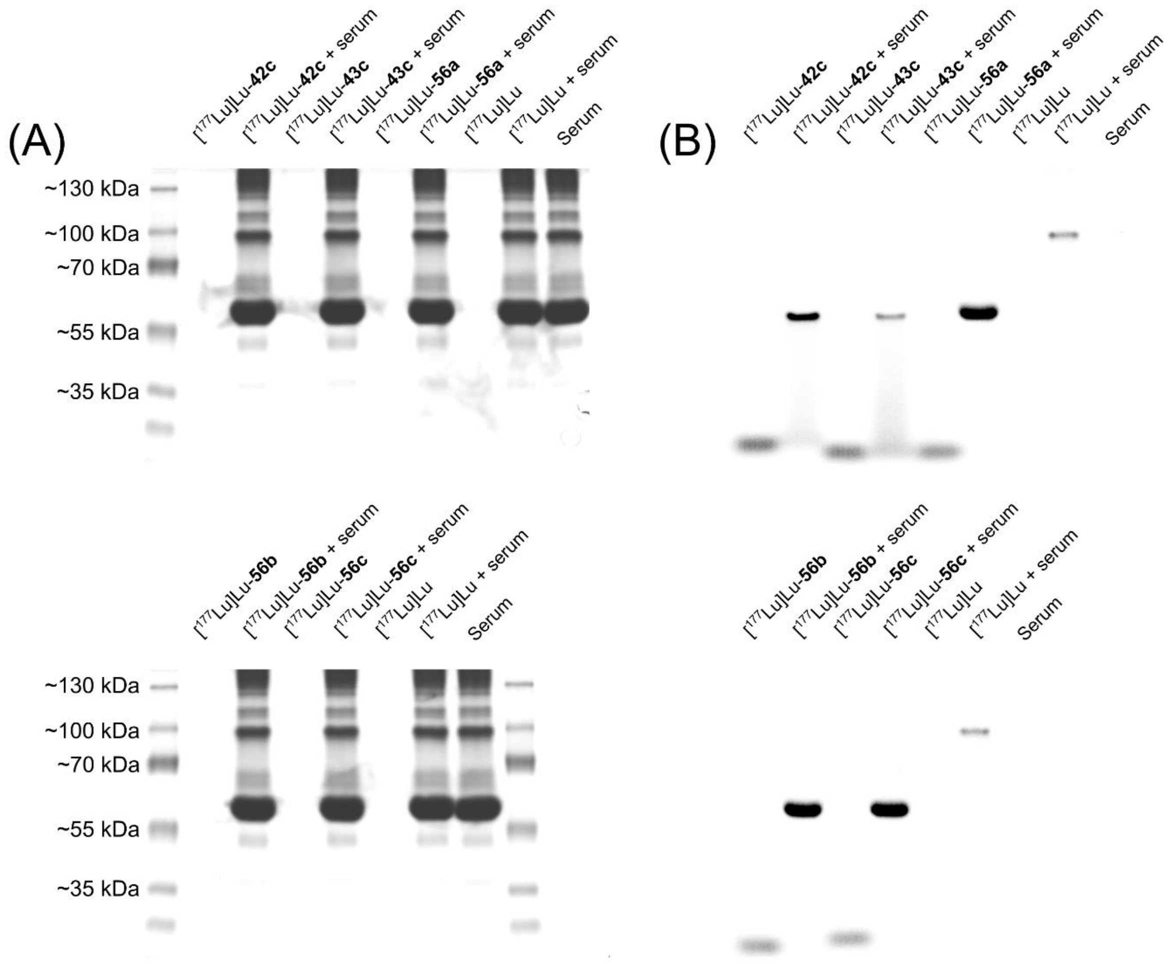

Figure S239: Binding of ^{177}Lu-labeled PD-L1 ligands to human serum proteins. Colloidal Coomassie stained native polyacrylamide gels (A) and corresponding autoradiographs (B) showing electrophoretic separation of ^{177}Lu-labeled ligands 42c, 43c, 56a, 56b and 56c with and without incubation in human serum for 24 h. Incubation of uncomplexed [^{177}Lu]Lu^{3+} with human serum was used as control reaction.

8. In vitro: Saturation binding assays

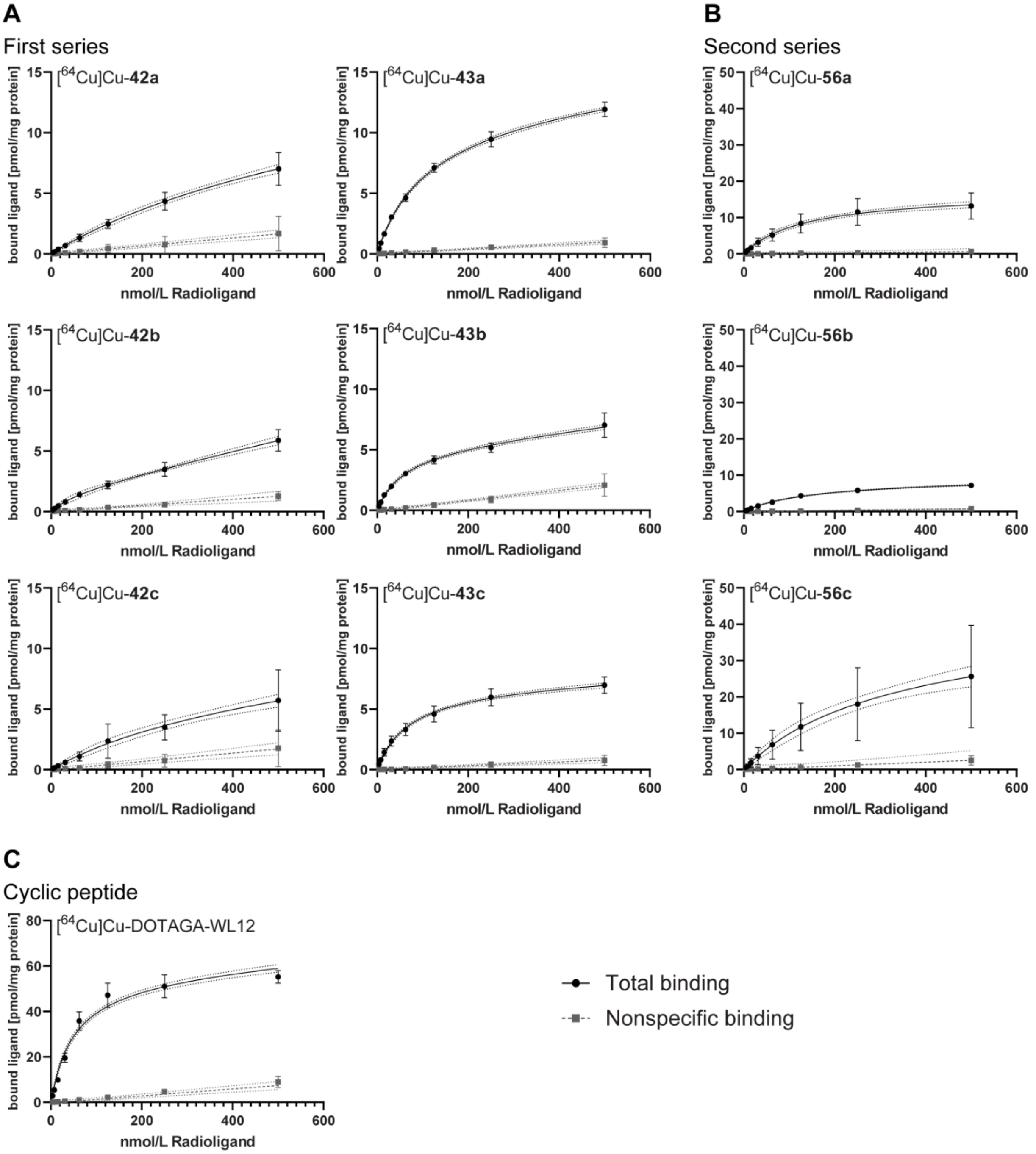

Figure S240: Non-linear iterative curve fitting of saturation binding experimental data. A) First series compounds [^{64}Cu]Cu-42a-c and [^{64}Cu]Cu-43a-c; B) Second series compounds [^{64}Cu]Cu-56a-c and C) cyclic peptide [^{64}Cu]Cu-DOTAGA-WL12. Curves show representative fits for total and nonspecific binding of 3 individual experiments combined, along with 95% confidence levels (dotted lines). Scaling is only comparable within A/B/C.

9. In vitro: Real-time radioligand binding

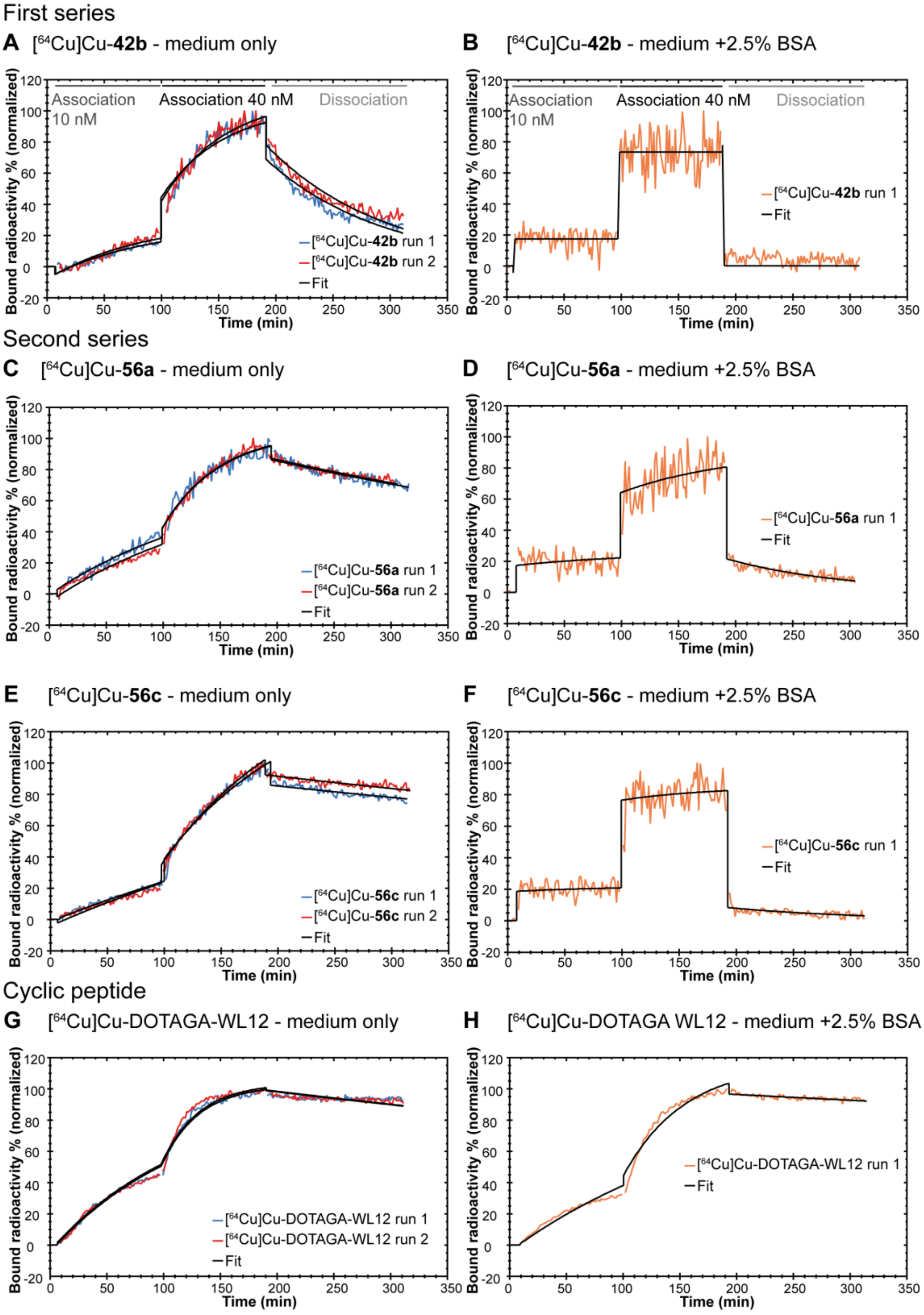

Figure S241: Real-time radioligand binding (trace) of one first, two second series compounds and the cyclic peptide WL12 in the absence(A/C/E/G) and presence (B/D/F/H) of 2.5% bovine serum albumin (BSA). Kinetic parameters (association rate constant k_a, dissociation rate constant k_d and dissociation constant K_D) are reported in Figure 3E.

10. In vivo: Qualitative PET scans

First series

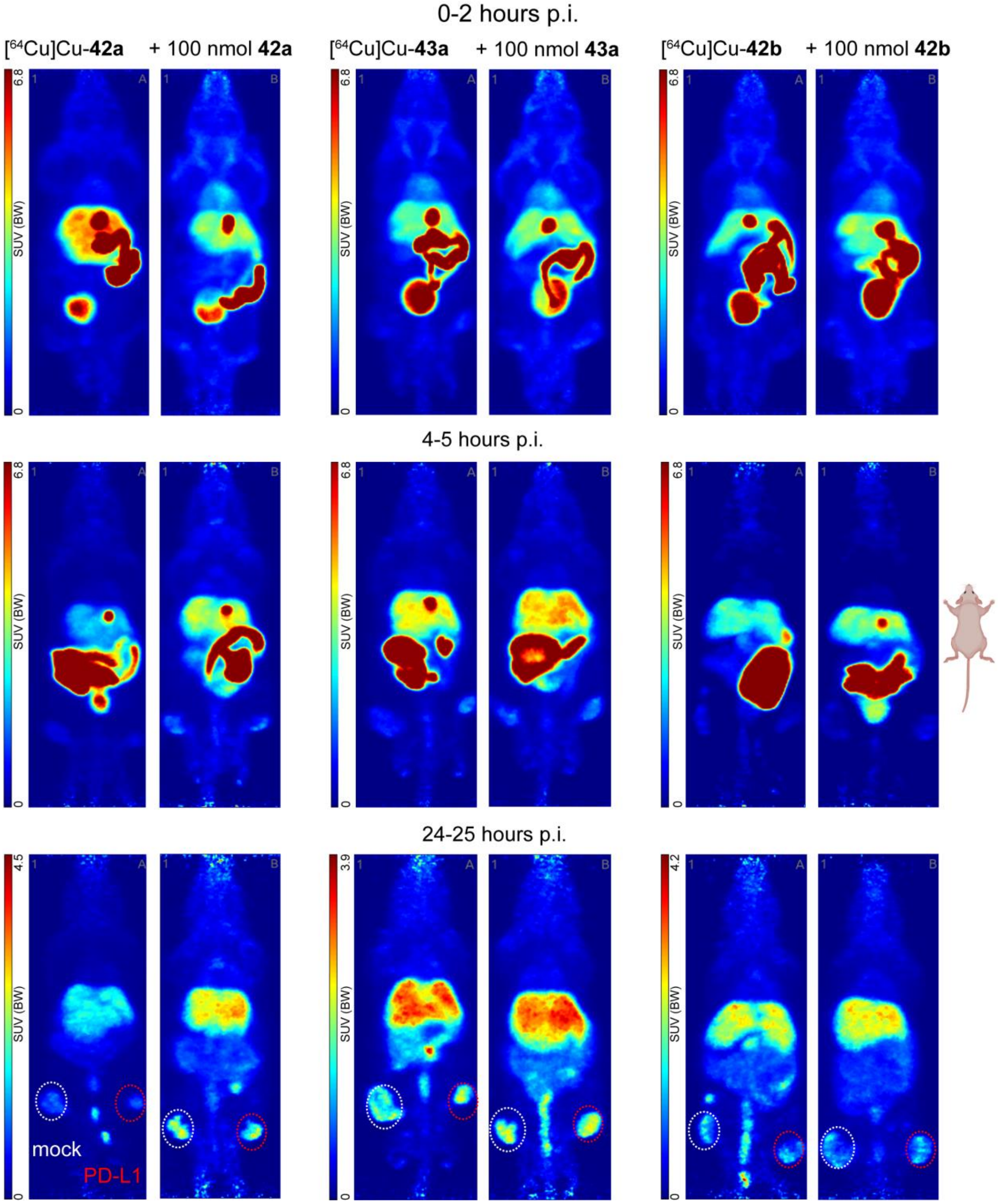

Figure S242: ***In vivo*** **distribution (maximum intensity projections) of ^{64}Cu-labeled first generation compounds 42a, 43a and 42b at 0-2, 4-5 and 24-25 h post injection (p.i.). SUV scales differ across images.**

First series

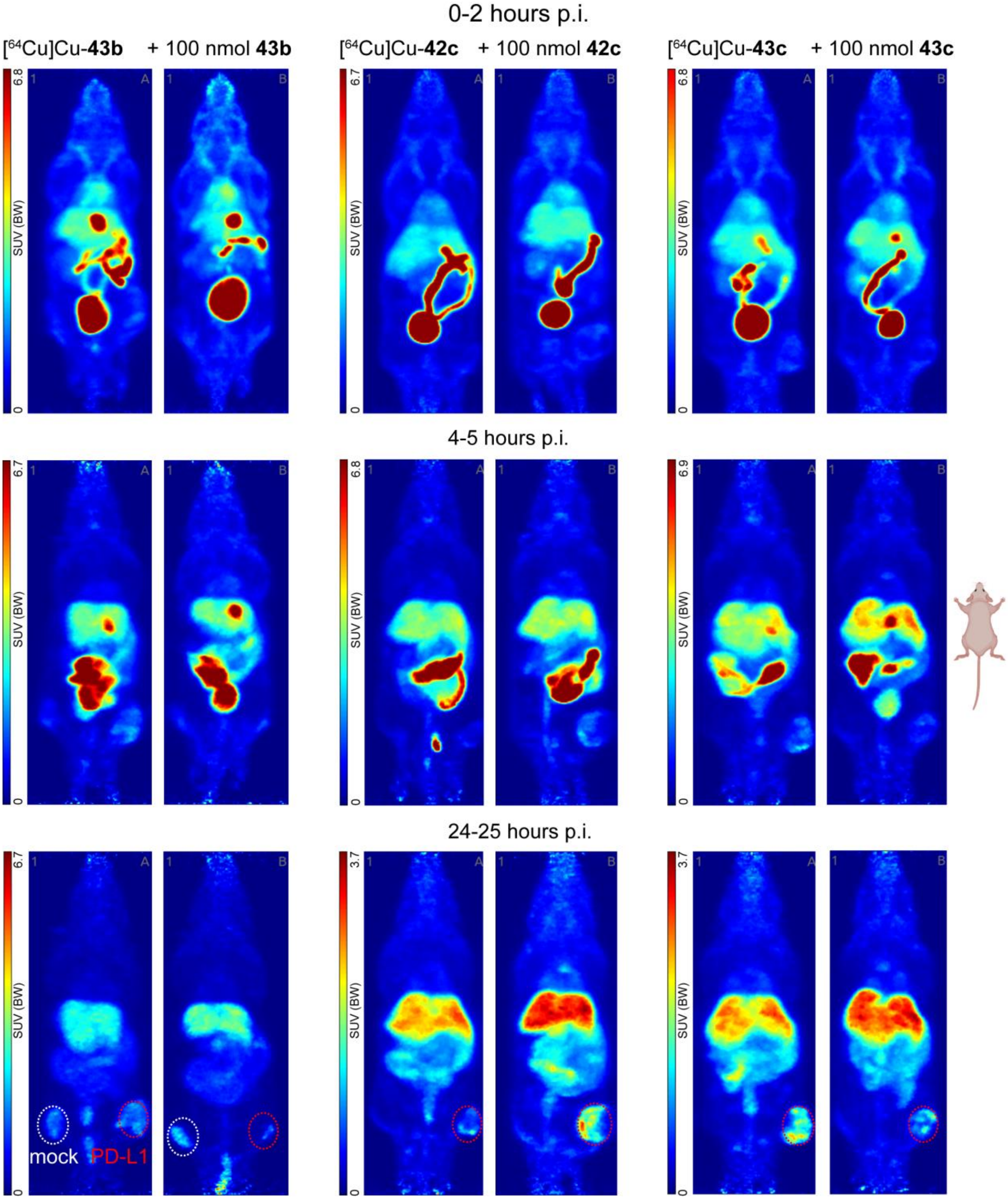

Figure S243: ***In vivo*** **distribution (maximum intensity projections) of ^{64}Cu-labeled first generation compounds 43b, 42c, 43c at 0-2, 4-5 and 24-25 h post injection (p.i.). SUV scales differ across images.**

Second series

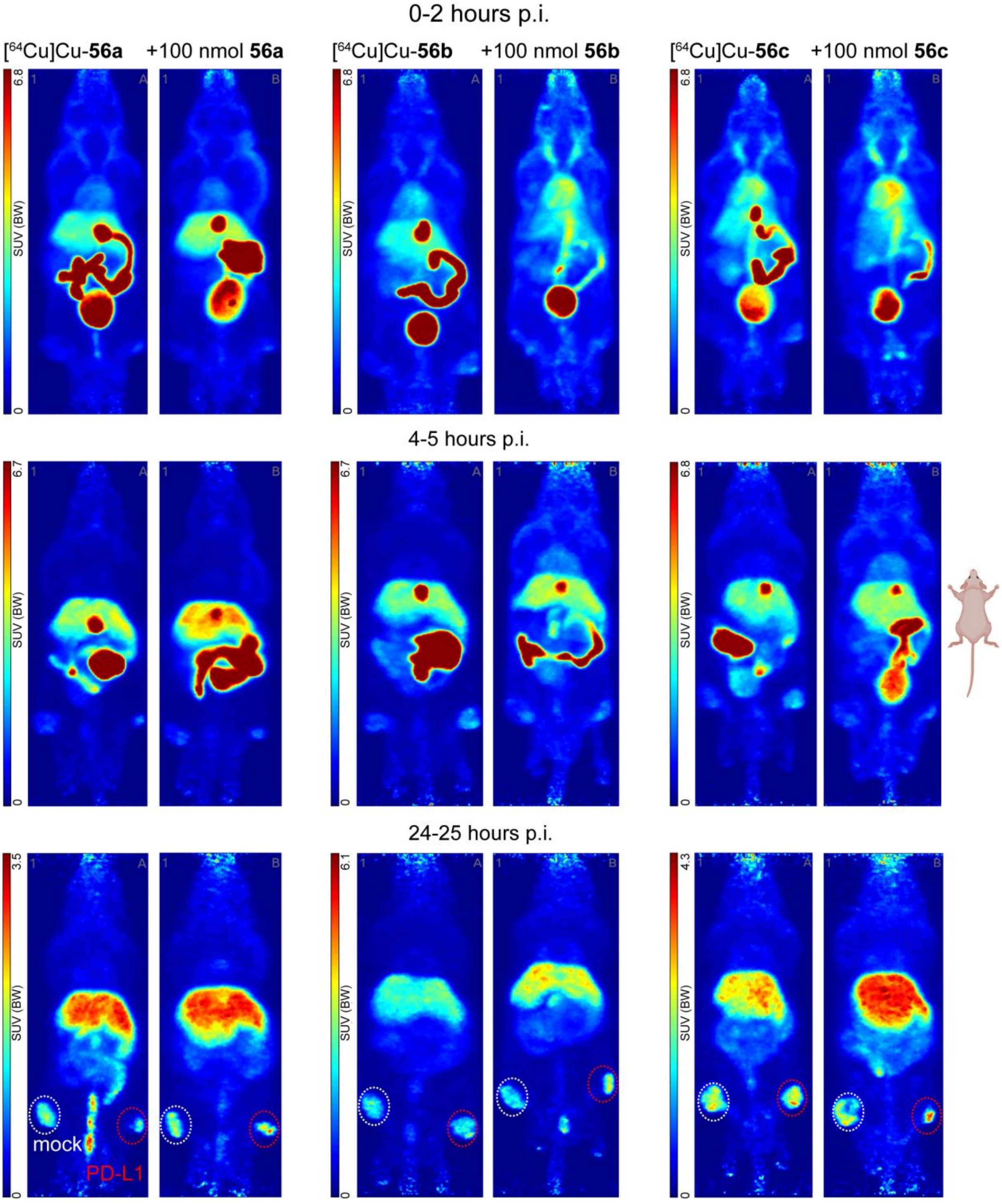

Figure S244: ***In vivo*** **distribution (maximum intensity projections) of ^{64}Cu-labeled second generation compounds 56a-c at 0-2, 4-5 and 24-25 h post injection (p.i.). SUV scales differ across images.**

Table S2: SUV$_{max}$ values (mean ± S.D., if applicable) of first and second-series compounds derived from PET data in PD-L1 overexpressing and mock tumors at different timepoints post injection (p.i.).

Compound (# animals)		1-2 hours p.i.	4-5 hours p.i.	24-25 hours p.i.
		SUV max	SUV max	SUV max
[^{64}Cu]Cu-**42a** (n=1)	hPD-L1	0.45	0.69	1.15
	Mock	0.58	0.67	1.19
[^{64}Cu]Cu-**43a** (n=1)	hPD-L1	1.58	2.13	2.64
	Mock	0.94	1.30	2.50
[^{64}Cu]Cu-**42b** (n=1)	hPD-L1	0.77	1.97	1.81
	Mock	0.78	1.56	2.28
[^{64}Cu]Cu-**43b** (n=1)	hPD-L1	1.91	2.18	2.35
	Mock	1.62	1.31	2.28
[^{64}Cu]Cu-**42c** (n=2)	hPD-L1	1.47 (0.16)	2.38 (0.35)	2.63 (0.99)
	Mock	0.92 (0.04)	1.01 (0.41)	0.71 (0.25)
[^{64}Cu]Cu-**43c** (n=2)	hPD-L1	1.85 (0.30)	2.32 (0.79)	2.41 (0.45)
	Mock	0.98 (0.21)	0.69 (0.18)	0.42 (0.22)
[^{64}Cu]Cu-**56a** (n=1)	hPD-L1	1.84	1.97	2.01
	Mock	1.05	1.56	2.07
[^{64}Cu]Cu-**56b** (n=3)	hPD-L1	2.44 (0.70)	2.58 (0.21)	3.43 (0.47)
	Mock	1.42 (0.17)	1.54 (0.37)	3.04 (0.73)
[^{64}Cu]Cu-**56c** (n=2)	hPD-L1	2.36 (0.43)	3.05 (0.23)	3.52 (0.44)
	Mock	1.20 (0.02)	1.42 (0.28)	2.26 (1.46)

Table S3: SUV$_{max}$ values (mean ± S.D., if applicable) of first and second-series compounds with cold compound as blocking substance derived from PET data in PD-L1 overexpressing and mock tumors at different timepoints post injection (p.i.).

Compound (# animals)		1-2 hours p.i.	4-5 hours p.i.	24-25 hours p.i.
		SUV max	SUV max	SUV max
[^{64}Cu]Cu-**56a** (n=1)	hPD-L1	1.84	1.97	2.01
	Mock	1.05	1.56	2.07
[^{64}Cu]Cu-**56a** +100 nmol 56a (n=1)	hPD-L1	1.80	1.82	3.11
	Mock	0.95	1.55	2.23
[^{64}Cu]Cu-**56b** (n=3)	hPD-L1	2.44 (0.70)	2.58 (0.21)	3.43 (0.47)
	Mock	1.42 (0.17)	1.54 (0.37)	3.04 (0.73)
[^{64}Cu]Cu-**56b** +100-500 nmol 56b (n=3)	hPD-L1	2.21 (0.29)	2.83 (0.56)	4.25 (0.29)
	Mock	1.96 (0.50)	1.89 (0.09)	3.25 (0.18)
[^{64}Cu]Cu-**56c** (n=2)	hPD-L1	2.36 (0.43)	3.05 (0.23)	3.52 (0.44)
	Mock	1.20 (0.02)	1.42 (0.28)	2.26 (1.46)
[^{64}Cu]Cu-**56c** +100 nmol 56c (n=2)	hPD-L1	1.94 (0.71)	2.57 (0.91)	3.97 (0.19)
	Mock	1.79 (0.24)	1.69 (0.15)	3.44 (0.35)

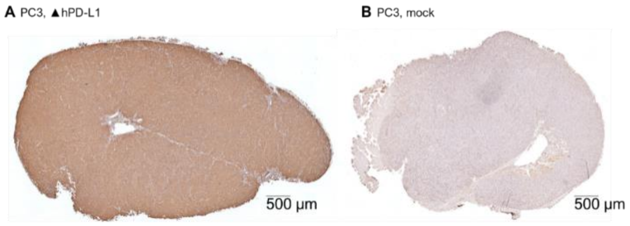

Figure S245: Immunostaining of random PD-L1 positive and mock tumors confirms target overexpression (PD-L1, A)) and absence thereof (mock, B).

11. References

(1) Yeung, K.-S.; Connolly, T. P.; Frennesson, D. B.; Grant-Young, K. A.; Hewawasam, P.; Langley, D. R.; Meng, Z.; Mull, E.; Parcella, K. E.; Saulnier, M. G.; et al. Compounds Useful as Immunomodulators. Patent WO 2017/066227 A1, April 20, 2017.
(2) Wünsch, S.; Breit, B. Probing o-Diphenylphosphanyl Benzoate (o-DPPB)-Directed C=C Bond Formation: Total Synthesis of Dictyostatin. *Chem. Eur. J.* **2015**, *21*, 2358-2363. DOI: 10.1002/chem.201406252.
(3) Rombouts, F.; Peschiulli, A. Preparation of Macrocyclic Indole Compounds as MCL-1 Inhibitor. Patent WO/2020/254471, June 18, 2020.
